# Reagent-controlled regiodivergent ring expansions of steroids

**DOI:** 10.1038/s41467-018-03248-2

**Published:** 2018-03-05

**Authors:** Manwika Charaschanya, Jeffrey Aubé

**Affiliations:** 1 Department of Medicinal Chemistry, University of Kansas, Lawrence, KS 66045 USA; 2 Division of Chemical Biology and Medicinal Chemistry, UNC Eshelman School of Pharmacy, University of North Carolina, Chapel Hill, NC 27599-7363 USA

## Abstract

Ring expansion provides a powerful way of introducing a heteroatom substituent into a carbocyclic framework. However, such reactions are often limited by the tendency of a given substrate to afford only one of the two rearrangement products or fail to achieve high selectivity at all. These limitations are particularly acute when seeking to carry out late-stage functionalization of natural products as starting points in drug discovery. In this work, we present a stereoelectronically controlled ring expansion sequence towards selective and flexible access to complementary ring systems derived from common steroidal substrates. Chemical diversification of the reaction intermediate affords over 100 isomerically pure analogs with spatial and functional diversity. This regiodivergent rearrangement, and the concept of using chiral reagents to affect regiocontrol in chiral natural products, should be broadly applicable to late-stage natural product diversification programs.

## Abstract (web summary)

Late-stage diversification of natural products is an important starting point for drug discovery. Here, the authors use chiral reagents to perform the regiocontrolled ring expansion of steroid precursors and achieve more than 100 isomerically pure analogs with spatial and functional diversity.

## Introduction

**Fig. 1:**
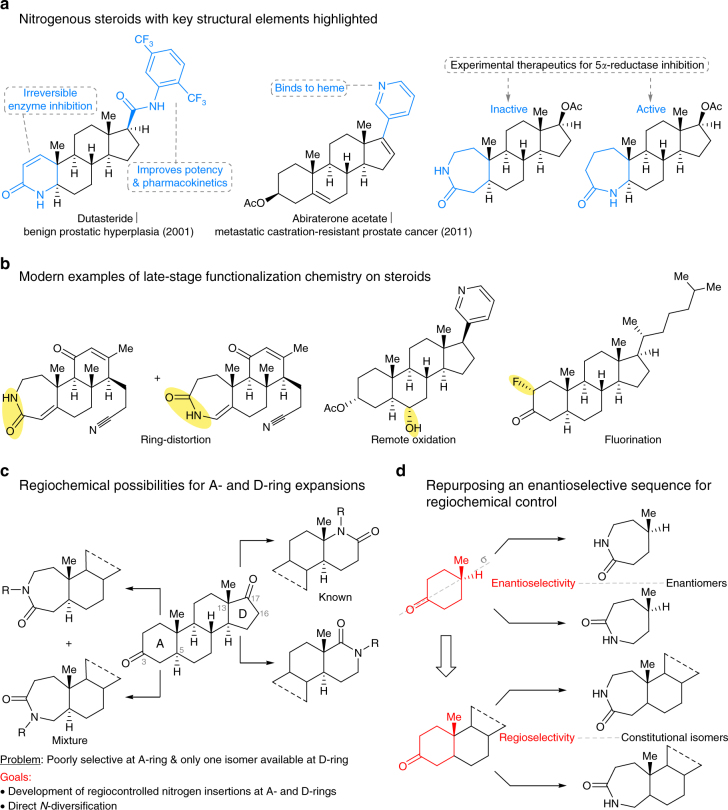
Rationale of project and challenges in steroid library construction. **a** Examples of nitrogen-containing steroidal drugs; the blue-colored bonds represent A- and D-ring sites essential for biological activity. The site of substitution often has a profound effect on biological activity^41^. **b** Representative contemporary approaches to late-stage functionalization of steroids^9, 18, 19^. **c** Regiochemical challenges accompanying ring expansion reactions of 3- and 17-oxosteroids. **d** The relationship between enantiotopic group migration selectivity in a C-4 substituted cyclohexanone and regioselectivity in a 3-oxosteroid

Natural products represent valuable starting points for drug discovery, with late-stage diversification emerging as an attractive approach for generating new bioactive agents from complex scaffolds^1–6^. Despite many recent advances, a challenge common to late-stage natural product functionalization remains: complex structures often have profound biases that dictate the isomeric outcomes of reactions carried out on them. Numerous therapeutic agents and chemical probes are based on steroid skeleta, with new examples continuing to appear in the clinic. The nitrogenous steroids dutasteride^7^ and abiraterone acetate^8^ (Fig. 1a) are emblematic of this contemporary interest, but relatively few approaches to *N*-containing steroid collections have been reported in recent years^9–11^. In addition, we note that steroids figure prominently in classic^12–15^ and contemporary approaches^9,16–19^ to late-stage diversification (Fig. 1b).

We focused on the A- and D-ring expansions because the biological activities of steroids often depend on the structures of these rings. The two limiting cases for regiochemical control are shown in Fig. 1c, with the nitrogen ring expansion of a 17-oxosteroid representing a classic case of unidirectional migration in an α-substituted ketone. In the D-ring, this corresponds to migration of the highly substituted C-13 carbon vs. migration of the C-16 carbon methylene group. The standard outcome of a classic Beckmann or Schmidt rearrangement—which have been previously carried out on steroids^20–22^—is the exclusive migration of C-13, whereas the migration of C-16 is unknown except through multistep sequences^22^.

The problem of A-ring rearrangement is even less straightforward because the two methylene groups attached to the 3-oxosteroid are similarly substituted; i.e., this ketone is only non-symmetrical at a site distal from the actual point of chemical reactivity. Indeed, reported ring expansion reactions at this position are poorly selective^23,24^. An attractive solution is evident if one conceptually embeds an achiral cyclohexanone into the chiral context of the A-ring steroid (Fig. 1d), which renders the two potential migrating methylene groups that were enantiotopic in the monocyclic case, diastereotopic. Only a few examples of using an asymmetric reaction to effect regiocontrol are known^25–28^ and none have been deployed in late-stage functionalization.

In this paper, we describe a global approach to this problem that uses stereochemically controlled ring expansions to effect regiochemical control in a complex molecular setting, thus permitting the directed introduction of a nitrogen-containing group in settings where no regiochemical control was previously possible or overcoming strong substrate bias to afford previously inaccessible constitutional isomers. We expect that this approach should be applicable to regiochemical variation in other classes of natural products or other complex structures.

## Results

### Modifications of A-ring steroids

**Fig. 2:**
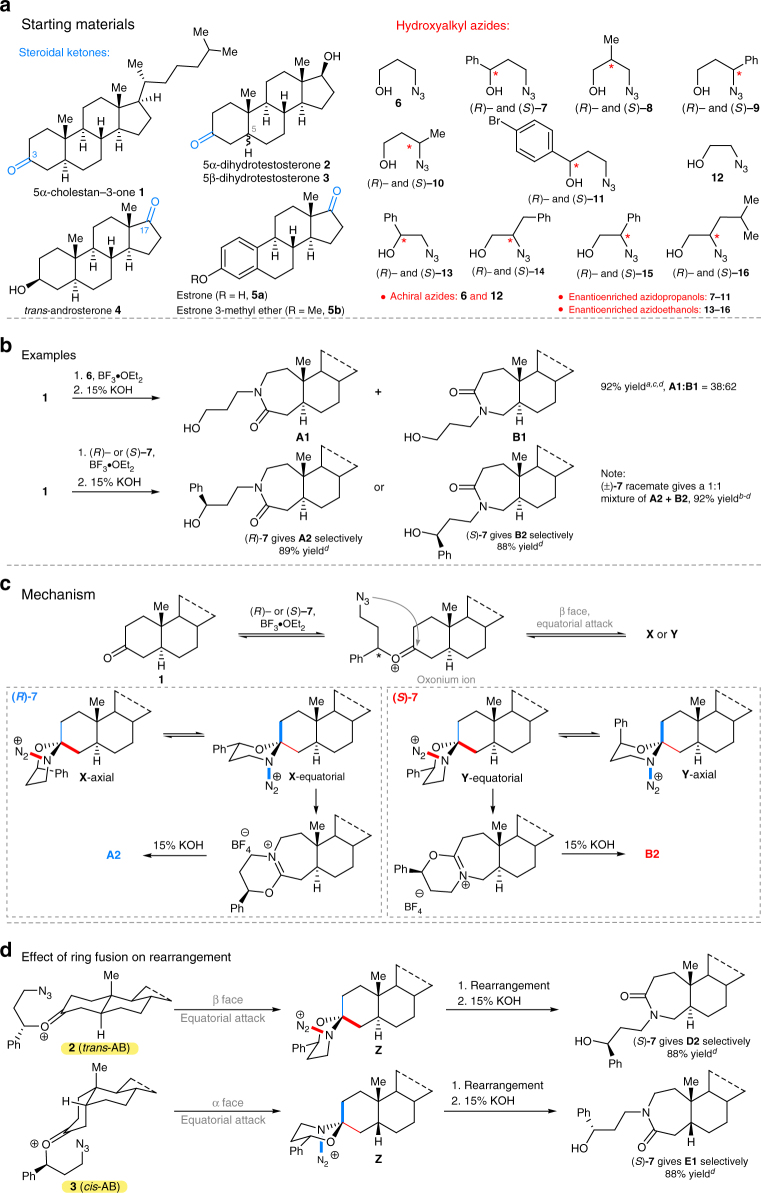
Regioselective ring expansions of 3-oxosteroids. **a** Steroids and hydroxyalkyl azides used in this study. The blue ketones indicate modification sites. **b** Ring expansions obtained by treating **1** with 3-azidopropanol **6**, 3-azido-1-phenylpropanol (*R*)-**7** and (*S*)-**7**. **c** Mechanistic rationale for ring expansion chemistry differentiating between C-2 (blue bond) and C-4 (red bond) migration; key antiperiplanar relationship between the migrating group and the N_2_^+^ leaving group is indicated by bold bonds. **d** Dependence of migration outcome on C-5 stereochemistry. ^*a*^Inseparable mixture by normal phase chromatography. ^*b*^Separable mixture by normal phase chromatography, but for this control experiment the isomers were not separated. ^*c*^Ratio determined by ^1^H nuclear magnetic resonance (NMR). ^*d*^Isolated yield after chromatography on silica. See Supplementary Methods for full synthetic details. **A** and **B** series are 5α-cholestan-3-one derivatives; **D2** is a 5α-dihydrotestosterone derivative; **E1** is a 5β-dihydrotestosterone derivative; **X** and **Y** are proposed spirocyclic intermediates (including both chair conformers as designated); 15% KOH, 15% aqueous solution of potassium hydroxide; *, signifies site of stereochemistry

In this study, we reacted readily available and biologically relevant 3- and 17-oxosteroids **1**–**5** with hydroxyalkyl azides **6**–**16** (Fig. 2a). We first addressed the problem of A-ring selectivity using a stereocontrolled ring expansion to influence the regioselectivity of the *N*-side chain installation. As expected, the ring expansion of 5α-cholestan-3-one **1** with achiral 3-azidopropanol **6** showed a modest inherent bias for one ring expansion outcome over the other giving **A1** and **B1** as a 38:62 mixture as shown in Fig. 2b (an analogous mixture of **C1** and **D1** derived from **2** are found in Supplementary Table 1). To surmount this, we tested a reagent control approach in which enantiomerically pure (*R*)- or (*S*)-3-azido-1-phenylpropanol, (*R*)-**7** or (*S*)-**7**, were separately reacted with **1**. This afforded spectroscopically pure lactams **A2** and **B2**, respectively, demonstrating high regiochemical control in each case. As expected, the reaction with racemic 3-azido-1-phenylpropanol (±)-**7** gave an equimolar mixture of **A2** and **B2** (Fig. 2b). In this case, each half of the racemate provides a specific regioisomer with high selectivity.

The mechanism involves the initial formation of a set of spirocyclic 1,3-oxazinanes that readily equilibrate prior to bond migration and establishment of product structure (Fig. 2c)^28,29^. Three principles govern the outcome of these reactions. The first is an equatorial attack of azide upon the initial oxonium structure, which establishes the stereogenicity of the spirocyclic carbon. This is, in turn, is followed by preferential reaction through a 1,3-oxazinane conformer with the phenyl group in an equatorial position and the stereoelectronically enforced migration of a carbon antiperiplanar to a departing N_2_^+^ group (which must be axial to permit the necessary arrangement between the migrating and leaving groups). In the reaction of an enantiomerically pure ketone with a chiral reagent, all three factors combine to enforce migration of one possible methylene group over the other, which switches when the opposite enantiomer of the hydroxyalkyl azide is used (i.e., the red vs. the blue bonds in Fig. 2c). Thus, ketones **1**–**3** can be regioselectively converted into either desirable lactam in high yields by the expedient choice of (*R*)- or (*S*)-azide, respectively, in the reaction sequence.

Similar results were obtained using enantioenriched azidopropanols substituted at any of the propyl carbons (hydroxyalkyl azides **8**–**11**; analogs **A3**–**A5**, **B3**–**B5**, **C2–C6**, **D2–D6** in Supplementary Table 1), including *p*-bromophenyl groups suitable for downstream cross-coupling activities. Generally, product selectivity was excellent with some erosion observed in cases previously observed^28^ to lead to less-diastereoselective reactions, such as 2-methyl-3-azidopropanols (*R*)-**8** and (*S*)-**8** and some substituted azidoethanol analogs (**A6**–**A9**, **B6**–**B9**, **C7**, **D7**; structure–selectivity data in Supplementary Table 2).

The C-5 epimeric steroids **2** and **3** lead to opposite nitrogen insertion selectivities to one another because equatorial attack is β in the former case, *trans*-AB ring, but α for compounds containing a *cis*-AB ring fusion. Interestingly, changing one of the three selectivity principles outlined for the mechanism reverses the overall outcome of the regioselectivity. For example in Fig. 2d, using the same chiral reagent (*S*)-**7** reverses the overall directionality of the nitrogen insertion reaction, resulting in opposite steroids **D2** and **E1** (for **3**-derived analogs **E1**, **E2**, **F1**, **F2** see Supplementary Table 1 entries 22–25). In total, 32 new nitrogenous A-ring-expanded steroids were obtained from reacting 3-oxosteroids **1–3** with hydroxyalkyl azides **6**–**14** (see Supplementary Discussion for an expanded mechanistic discussion).

### Modifications of D-ring steroids

**Fig. 3:**
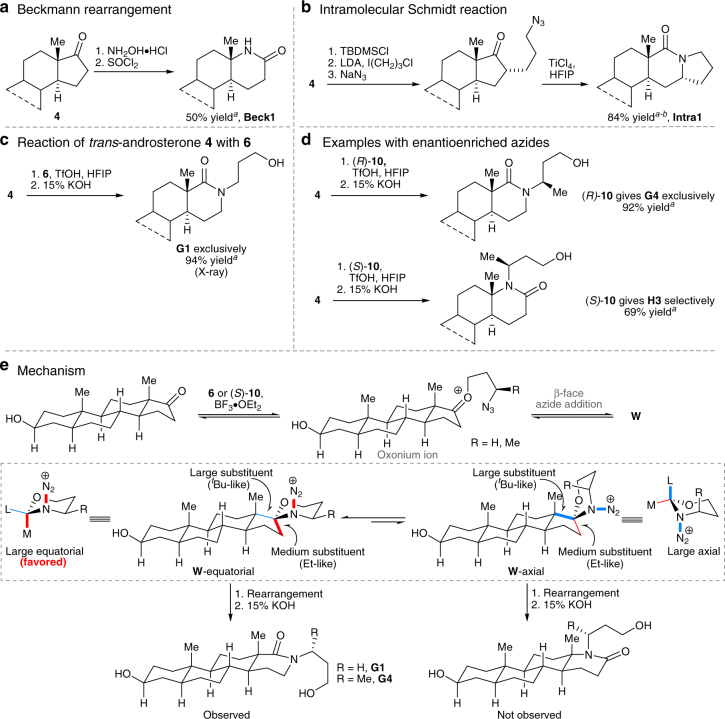
Regioselective ring expansions of 17-oxosteroids. **a** Beckmann reaction of a C-17 ketone affords selective migration of C-13. **b** Forced migration of C-16 using an intramolecular Schmidt reaction. **c** Reaction of a 17-oxosteroid with 3-azidopropanol **6** regioselectively results in C-16 migration. **d** Reactant control of ring expansions of **4** by treatment with 3-azidobutanol (*R*)-**10** and (*S*)-**10**. **e** Mechanism considerations of regiochemical outcomes for the reaction using achiral azide **6** in **c** and chiral reagent (*R*)-**10** in **d**. See Supplementary Methods for full synthetic details. ^*a*^Isolated yield after chromatography on silica. ^*b*^Schmidt reaction with concomitant deprotection of protecting group TBDMS. See Supplementary Methods for full synthetic details. **G** and **H** series are *trans*-androsterone derivatives; **W**, proposed spirocyclic intermediates (two conformers); TBDMS, *tert*-butyldimethylsilyl; LDA, lithium diisopropylamide 1 M in tetrahydrofuran; TfOH, trifluoromethanesulfonic acid; HFIP, hexafluoro-2-propanol; 15% KOH, 15% aqueous solution of potassium hydroxide

We then sought a solution for the problem of D-ring expansion selectivity. Classical nitrogen insertion reactions of 2-substituted ketones typically afford lactams in which the more highly substituted α-group migrates^30.^ In the present case, application of a Beckmann rearrangement via the *E*-oxime derived from **4** nicely afforded lactam **Beck1** in accord with literature precedent (Fig. 3a)^20–22,31^. One surefire way of forcing the alternative C-16 carbon to migrate is shown in Fig. 3b, in which attachment of an azidopropane side chain onto a steroid was followed by an intramolecular Schmidt reaction^30,32,33^ to afford interesting pentacyclic compounds like **Intra1** (3 analogs of this type were made, **Intra1**–**3**; see Supplementary Methods). However, this is a limited solution and a more general way of selectively diversifying this position of the steroidal backbone was pursued.

The regioselectivity of hydroxyalkyl azide insertions into simple 2-substituted cycloalkanones is highly variable and dependent on the nature of the substituent^34,35^. In the present case, treatment of **4** with 3-azidopropanol **6** led to lactam **G1** as the exclusive isomer in excellent yields (Fig. 3c). Owing to the relatively hindered nature of the D-ring ketones, a survey of conditions was first carried out; the best results were obtained by carrying out the reaction in hexafluoroisopropanol^36^ (optimization of reaction conditions shown in Supplementary Table 3). Analysis of the two possible 1,3-oxazinane intermediates suggests that the quaternary C-13 serves as a large substituent while the C-16 methylene is relatively small (Fig. 3e). Migration of C-16 occurs through the preferred oxazinane conformer to afford the observed product **G1** exclusively (structure confirmed by X-ray crystallography; an expanded mechanistic discussion is provided in the Supplementary Discussion).

Despite the apparently high substrate bias of 17-oxosteroids with 3-azidopropanol **6**, it proved possible to completely divert the reaction using chiral azides as reactants (Fig. 3d). When **4** was reacted with (*R*)-**10**, migration of C-16 was exclusively observed, similar to that observed with using achiral azide **6**. Remarkably, the opposite migration occurred when the reaction was carried out using (*S*)-**10**, suggesting that the intrinsic preference of the chiral hydroxyalkyl azide forcibly led to C-13 migration over the natural substrate preference for C-16 migration. In general, hydroxyalkyl azides able to enact high regioselectivity in the A-ring also gave high selectivity in the D-ring (25 cases examined: 2 D-ring substrates × 12 azides give *trans*-androsterone derivatives **G1**–**G8** and **H1**–**H6**, and estrone derivatives **I1**–**I9** and **J1**–**J6**; see Supplementary Tables 4 and 5). The power of the present approach is illustrated by the ability to divert the regiochemistry of these reactions with chiral reagents despite a large substrate preference.

### Diversification of intermediates and traceless ring expansions

**Fig. 4:**
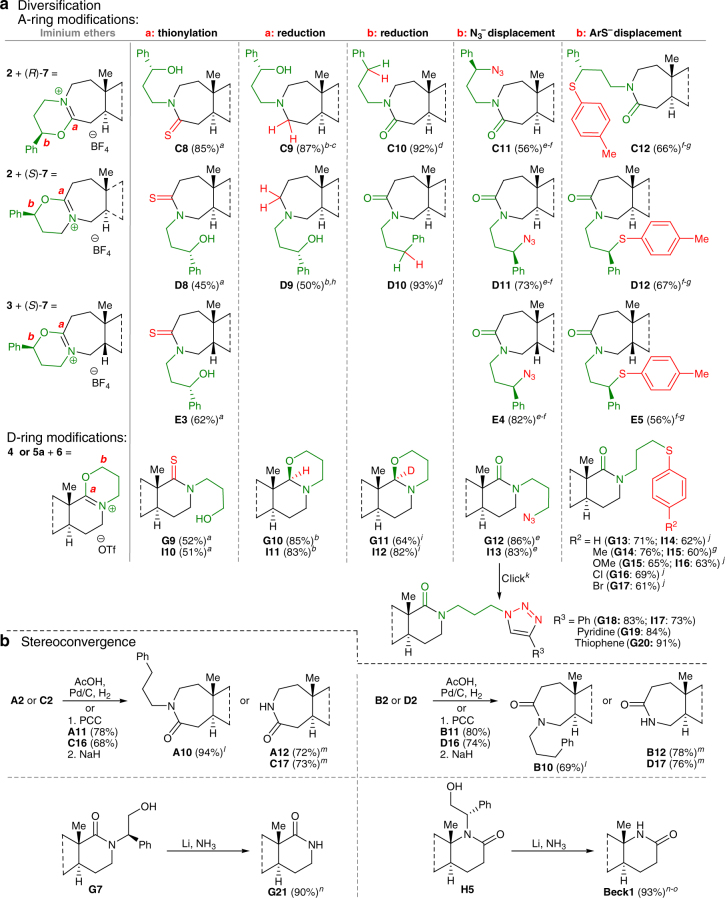
Structural diversification. **a** Diversification of iminium ethers for A-ring and D-ring iminium ether intermediates. Green text highlights the azide component and red text highlights the nucleophile component; positions ***a*** and ***b*** (in red) mark the sites of particular transformations. Yields refer to isolated yields of products after chromatography on silica. **b** Stereoconvergence and synthesis of parent NH lactams. Yields refer to isolated yields of products after chromatography on silica. ^*a*^Na_2_S, THF or DMF, 65 °C. ^*b*^NaBH_4_, MeOH, rt. ^*c*^**C9** was also obtained following LAH reduction of **C2** (51%). ^*d*^Hydrogenation using 10% Pd/C, EtOH. ^*e*^NaN_3_, DMF, 70 °C. ^*f*^Inversion of stereochemistry. ^*g*^4-Methylbenzenethiol, DMF, 75 °C. ^*h*^**D9** was also obtained following LAH reduction of **D2** (58%). ^*i*^NaBD_4_, MeOH, rt. ^*j*^Stock solutions of sodium thiophenoxides, DMF, 75 °C. ^*k*^Substituted acetylene, CuSO_4_•5H_2_O, sodium L-ascorbate, ^*t*^BuOH/H_2_O. ^*l*^Hydrogenation using 10% Pd/C, AcOH, EtOH. ^*m*^Removal of non-benzylic side chain via treatment with base. ^*n*^Removal of benzylic side chain via dissolving metal reduction. ^*o*^Isolated product is **Beck1**. See Supplementary Methods for full synthetic details. **C** and **D** are 5α-dihydrotestosterone derivatives; **E** and **F** are 5β-dihydrotestosterone derivatives; **G** are *trans*-androsterone derivatives; **I** are estrone derivatives. AcOH, acetic acid; PCC, pyridinium chlorochromate; NaH, sodium hydride in 60% mineral oil; Na_2_S, sodium sulfide; THF, tetrahydrofuran; DMF, dimethylformamide; NaBH_4_, sodium borohydride; MeOH, methanol; EtOH, ethanol, LAH, lithium aluminum hydride in 1 M THF; NaN_3_, sodium azide; NaBD_4_, sodium borodeuteride; ^*t*^BuOH, *tert*-butanol; H_2_O, distilled water

The intermediacy of iminium ethers as primary rearranged products permits their ready diversification using experimentally straightforward chemistry (Fig. 4). Iminium ether intermediates, which were not isolated, are ambident electrophiles at positions ***a*** (hydrolysis, thioamide formation, or iminium ion reduction) or ***b*** (S_N_2 azide or thioaryl displacement with clean inversion, or benzylic reduction)^37–40^. Figure 4a illustrates how A-ring iminium ethers were telescoped with a sample set of four nucleophiles (13 out of 22 A-ring analogs are shown, arising from 2 A-ring substrates × 4 azides × 4 nucleophiles; full collection given in Supplementary Table 6). Similar transformations were achieved for D-ring iminium ethers (2 D-ring substrates × 1 azide × 4 nucleophiles, Supplementary Table 7). Interestingly, reduction of D-ring iminium ether with sodium borohydride or sodium borodeuteride stereoselectively led to previously unobserved 1,3-oxazinanes resulting from partial reduction (structure **G10** was confirmed by X-ray crystallography). Secondary diversification was accomplished by converting the D-ring azide-containing side chains to triazoles through alkyne cycloaddition chemistry.

Finally, these technologies provide easy access to the parent lactams of these steroids and examples lacking the stereochemical information used to effect the specific ring expansion reactions. For example, reduction of the iminium ion through path ***a*** was accomplished under very mild conditions compared to those typically employed for amide bond to amine conversions. Conversely, path ***b*** hydrogenation removed the regiodirecting stereocenter, resulting in a traceless and stereoconvergent control process (Fig. 4b). Notably, all four parent constitutional isomers of C-3- and C-17-derived lactams were obtained by removing the *N*-substituents by dissolving metal reduction or oxidation of the side chain hydroxyl group followed by elimination with base (Fig. 4b). Overall, we prepared 114 nitrogenous steroid analogs of which 105 are isomerically pure. All compounds described were prepared in ≥20 mg quantities and ≥90% purity, with most made from the corresponding ketone without isolation of iminium ether intermediates.

## Discussion

We have demonstrated the predictable and selective installation of nitrogen into the steroid backbone in four separate positions using stereoelectronically controlled ring expansion reactions of chiral hydroxyalkyl azides to direct nitrogen insertion wherein there is little innate migration preference (e.g,. 3-oxosteroids) or to even overcome a substantial substrate bias for a given directional result (e.g., 17-oxosteroids). This sort of reagent control enhances the chemist’s ability to rationally plan the placement of new functional groups onto a natural product backbone.

## Methods

### General procedure for the preparation of azasteroids

To a solution of a steroidal ketone **1**–**5** and hydroxyalkyl azide **6**–**16** in anhydrous CH_2_Cl_2_ or HFIP in a nitrogen-flushed vial at room temperature or 0 °C was added BF_3_•OEt_2_ or TfOH dropwise. The vial was capped and the reaction mixture was stirred at room temperature for 16–24 h. The solvent was removed under nitrogen and the residual iminium ion was treated with a nucleophile; either an aqueous solution of 15% KOH, Na_2_S, NaBH_4_, NaN_3_, or NaSAr (DMF stock solutions). The mixture was stirred vigorously at a specified temperature and time. Purification of analogs was carried out by an automated medium-pressure liquid chromatography system on normal phase silica gel columns.

Full experimental details and characterization of data for all new compounds are provided in the Supplementary Methods.

### Data availability

All data associated with these findings are available in the paper or supplementary information, except for X-ray crystallographic data, which has been deposited in the Cambridge Crystallography Database (CCDC 1583534 for **A9**, CCDC 1583535 for **B9**, CCDC 1583536 for **G1**, and CCDC 1583518 for **G10**). All other data are available from the authors upon reasonable request.

## Electronic supplementary material

Supplementary Information
