# Supplementary material for: Reagent-controlled regiodivergent ring expansions of steroids
Source: Nat Commun. 2018 Mar 5;9:934. doi: 10.1038/s41467-018-03248-2 (PMC5838248; doi:10.1038/s41467-018-03248-2)
Supplement: Supplementary file 1 — Supplementary Information [file 41467_2018_3248_MOESM1_ESM.pdf]

Reagent-Controlled Regiodivergent Ring Expansions of Steroids

Charaschanya and Aubé

Supplementary Tables

Supplementary Table 1. Reactions of 3-oxosteroids **1**–**3** with 1,3-hydroxyalkyl azides **6**–**11**.[a]

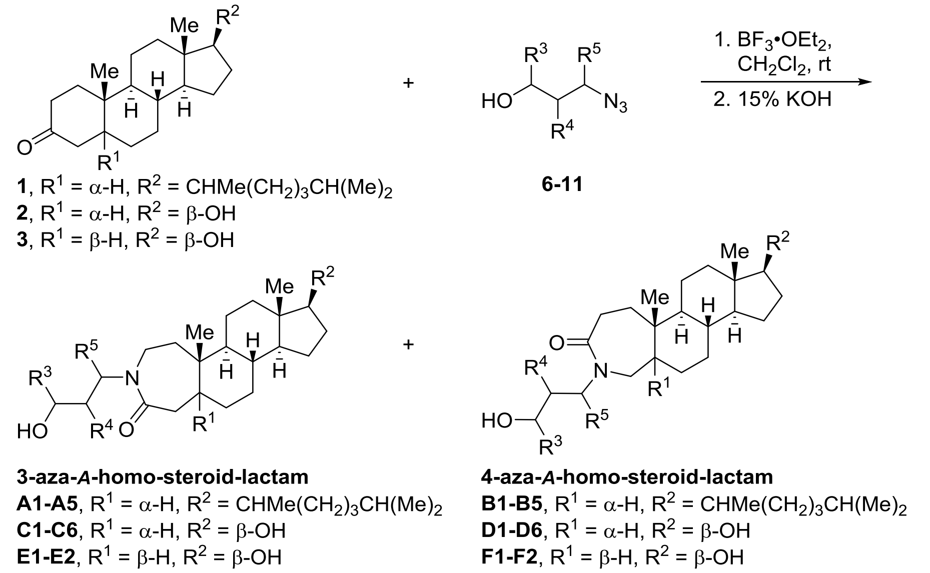

entry	steroid	azide	R^3	R^4	R^5	ratio A:B[b,c]	product(s)	isolated yield (%)[d]
1	**1**	**6**	H	H	H	38:62[e]	**A1** + **B1**	92[f]
2	**1**	(±)-**7**	(±)-Ph	H	H	50:50[e]	**A2** + **B2**	92[f]
3	**1**	(*S*)-**7**	(*S*)-Ph	H	H	5:>95	**B2**	88
4	**1**	(*R*)-**7**	(*R*)-Ph	H	H	>95:5	**A2**	89
5	**1**	(*S*)-**8**	H	(*S*)-Me	H	10:90[e]	**B3**	84
6	**1**	(*R*)-**8**	H	(*R*)-Me	H	87:13[e]	**A3**	71
7	**1**	(*S*)-**9**	H	H	(*S*)-Ph	>95:5	**A4**	88
8	**1**	(*R*)-**9**	H	H	(*R*)-Ph	5:>95	**B4**	87
9	**1**	(*S*)-**10**	H	H	(*S*)-Me	5:>95	**B5**	89
10	**1**	(*R*)-**10**	H	H	(*R*)-Me	>95:5	**A5**	92
11	**2**	**6**	H	H	H	40:60[e]	**C1** + **D1**	95
12	**2**	(*S*)-**7**	(*S*)-Ph	H	H	5:>95	**D2**	88
13	**2**	(*R*)-**7**	(*R*)-Ph	H	H	>95:5	**C2**	84
14	**2**	(*S*)-**11**	(*S*)-(4-Br)Ph	H	H	5:>95	**D3**	86
15	**2**	(*R*)-**11**	(*R*)-(4-Br)Ph	H	H	>95:5	**C3**	83
16	**2**	(*S*)-**8**	H	(*S*)-Me	H	13:87[e]	**D4**	87
17	**2**	(*R*)-**8**	H	(*R*)-Me	H	87:13[e]	**C4**	85
18	**2**	(*S*)-**9**	H	H	(*S*)-Ph	>95:5	**C5**	89
19	**2**	(*R*)-**9**	H	H	(*R*)-Ph	5:>95	**D5**	90
20	**2**	(*S*)-**10**	H	H	(*S*)-Me	5:>95	**D6**	85
21	**2**	(*R*)-**10**	H	H	(*R*)-Me	>95:5	**C6**	85
22	**3**	(*S*)-**7**	(*S*)-Ph	H	H	>95:5	**E1**	89
23	**3**	(*R*)-**7**	(*R*)-Ph	H	H	5:>95	**F1**	83
24	**3**	(*S*)-**9**	H	H	(*S*)-Ph	5:>95	**F2**	87
25	**3**	(*R*)-**9**	H	H	(*R*)-Ph	>95:5	**E2**	90

[a]See Supplementary Methods for reaction protocol. [b]Ratio was determined by ^1H NMR of the crude reaction mixture. [c]Only one regioisomer was observed by ^1H NMR of the crude reaction mixture, unless otherwise noted. [d]Isolated yields of only the major regioisomer, unless otherwise noted. [e]Mixture of regioisomers observed by ^1H NMR of the crude reaction mixture. [f]Isolated yields of a mixture of regioisomers.

Supplementary Table 2. Reactions of 3-oxosteroids **1**–**3** with 1,2-hydroxyalkyl azides **12**–**14**.[a]

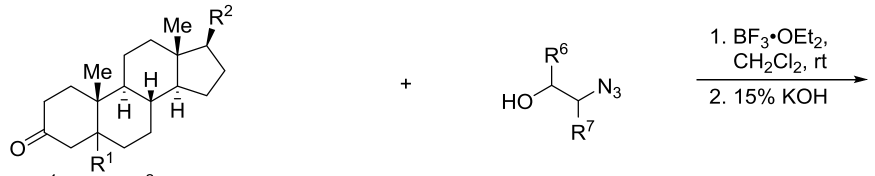

1, R^1 = α-H, R^2 = $CHMe(CH_2)_3CH(Me)_2$
2, R^1 = α-H, R^2 = β-OH
3, R^1 = β-H, R^2 = β-OH

12-14

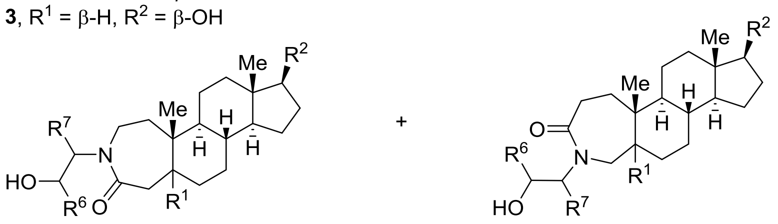

3-aza-*A*-homo-steroid-lactam
A6-A9, R^1 = α-H, R^2 = $CHMe(CH_2)_3CH(Me)_2$
C7, R^1 = α-H, R^2 = β-OH

4-aza-*A*-homo-steroid-lactam
B6-B9, R^1 = α-H, R^2 = $CHMe(CH_2)_3CH(Me)_2$
D7, R^1 = α-H, R^2 = β-OH

entry	steroid	azide	R^6	R^7	ratio[b] A:B or C:D	product(s)	isolated yield (%)
1	**1**	**12**	H	H	37:63[c]	**A6** + **B6**	95[d]
2	**1**	(*S*)-**13**	(*S*)-Ph	H	46:54	**A7** + **B7**	93[d]
3	**1**	(*R*)-**13**	(*R*)-Ph	H	33:67[c]	**A8** + **B8**	88[d,e]
4	**1**	(*S*)-**14**	H	(*S*)-CH_2Ph	ND	**B9**	81[f,g]
5	**1**	(*R*)-**14**	H	(*R*)-CH_2Ph	ND	**A9**	65[f,h]
6	**2**	(*S*)-**14**	H	(*S*)-CH_2Ph	ND	**D7**	76[f,i]
7	**2**	(*R*)-**14**	H	(*R*)-CH_2Ph	ND	**C7**	65[f]

[a]See Supplementary Methods for reaction protocols. [b]Ratio was determined by ^1H NMR of the final product. [c]Mixture of regioisomers observed by ^1H NMR of the crude reaction mixture, unless otherwise noted. [d]Isolated yield of a mixture of regioisomers. [e]Isomeric mixture was separated to give 26% isolated yield of **A8** and 46% isolated yield of **B8**. [f]Isolated yield of the major regioisomer. [g]Isolated 12% yield (<10.0 mg) of the minor regioisomer, however was not further characterized. [h]Isolated 22% yield (<17.0 mg) of minor regioisomer, however was not further characterized. [i]Minor regioisomer observed in the final product. ND = Not Determined.

Supplementary Table 3. Optimization of conditions for the reaction between *trans*-androsterone **4** and 3-azidopropanol **6**.[a]

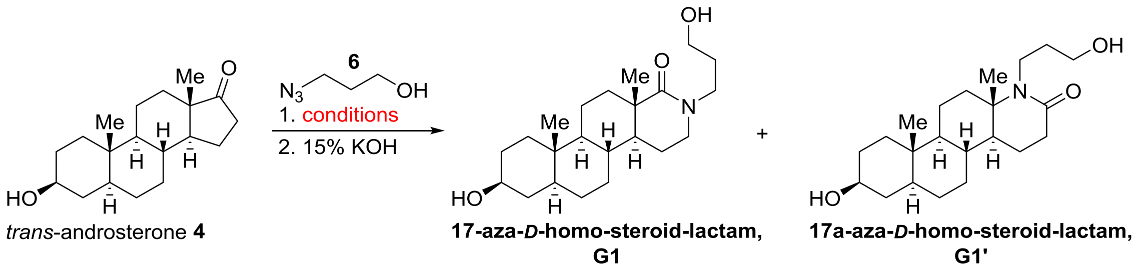

entry	azidopropanol 6 (equiv)	catalyst	catalyst (equiv)	solvent	G1:4[b,c]	isolated yield (%)[d]
1	3.0	BF_3•OEt_2	2.0	CH_2Cl_2	21:79	16
2	3.0	BF_3•OEt_2	5.0[e]	CH_2Cl_2	82:18	80
3	2.0	BF_3•OEt_2	7.0[e]	CH_2Cl_2	89:11	85
4	2.0	BF_3•OEt_2	1.1	$(CF_3)_2CHOH$	78:22	72
5	2.0	H_2SO_4	0.55[f]	$(CF_3)_2CHOH$	86:14	78
6	2.0	CF_3SO_3H	1.1	$(CF_3)_2CHOH$	>98:2[g]	94
7	2.0	CF_3SO_3H	1.1	CF_3CH_2OH	61:39	61
8	2.0	CF_3SO_3H	1.1	CH_2Cl_2	16:84	10

[a]See Supplementary Methods for reaction protocols. [b]Only regioisomer **G1** was observed by ^{1}H NMR of the crude reaction mixture. [c]Product conversion was determined by ^{1}H NMR of the crude reaction mixture. [d]Isolated yields of regioisomer **G1**. [e]Catalyst was added at 0 °C. [f]Generates 1.1 equiv of proton catalyst. [g]Complete conversion of *trans*-androsterone **4** to **G1** was observed by ^{1}H NMR of the crude reaction mixture.

Supplementary Table 4. Reactions of 17-oxosteroids **4** and **5** with 1,3-hydroxyalkyl azides **6**, **8**, and **10**.[a]

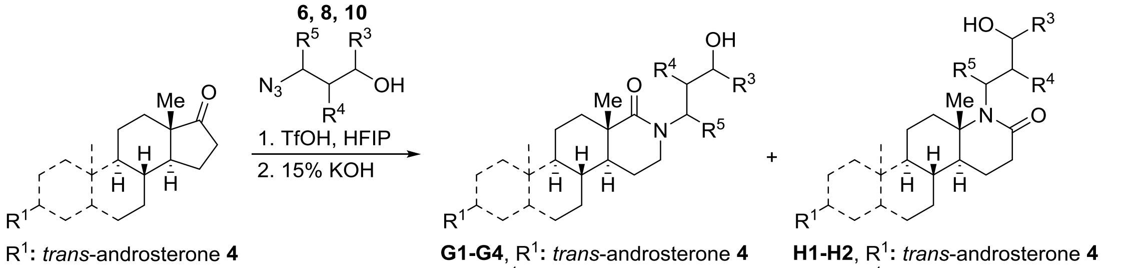

entry	steroid	azide	R^3	R^4	R^5	ratio[b,c] G:H or I:J	product(s)	isolated yield (%)[d]
1	**4**	**6**	H	H	H	>95:5	**G1**	94
2	**4**	(*S*)-**8**	H	(*S*)-Me	H	56:44	**G2** + **H1**	53:35[e]
3	**4**	(*R*)-**8**	H	(*R*)-Me	H	>95:5	**G3**	93
4	**4**	(*S*)-**10**	H	H	(*S*)-Me	>95:5	**H2**	69
5	**4**	(*R*)-**10**	H	H	(*R*)-Me	>95:5	**G4**	92
6	**5a**	**6**	H	H	H	ND	**I1**	91
7	**5a**	(*S*)-**8**	H	(*S*)-Me	H	60:40	**I2** + **J1**	52:34[e,f]
8	**5a**	(*R*)-**8**	H	(*R*)-Me	H	ND	**I3**	85
9	**5a**	(*S*)-**10**	H	H	(*S*)-Me	ND	**J2**	68
10	**5a**	(*R*)-**10**	H	H	(*R*)-Me	ND	**I4**	86
11	**5b**	**6**	H	H	H	ND	**I5**	93

[a]See Supplementary Methods for reaction protocols. [b]Ratio was determined by ^{1}H NMR of the crude reaction mixture. [c]Only one regioisomer was observed by ^{1}H NMR of the crude reaction mixture, unless otherwise noted. [d]Isolated yields of only the major regioisomer, unless otherwise noted. [e]Isolated yields of individual regioisomers. [f]Ratio determined by analytical HPLC of the crude reaction mixture.

Supplementary Table 5. Reactions of 17-oxosteroids **4** and **5** with 1,2-hydroxyalkyl azides **12** and **14**–**16**.[a]

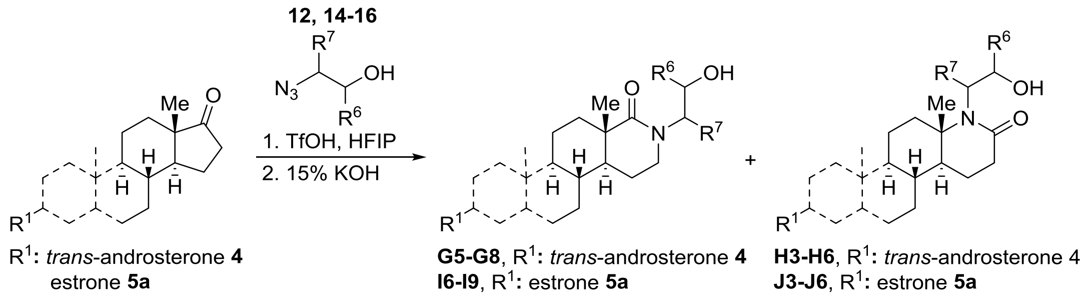

entry	steroid	azide	R^6	R^7	ratio G:H or I:J	product(s)	isolated yield (%)[b]
1	**4**	**12**	H	H	30:70[c]	**G5 + H3**	96[d]
2	**4**	(*S*)-**14**	H	(*S*)-CH_2Ph	ND	**H4**	89
3	**4**	(*R*)-**14**	H	(*R*)-CH_2Ph	ND	**G6**	82[e]
4	**4**	(*S*)-**15**	H	(*S*)-Ph	ND	**H5**	77
5	**4**	(*R*)-**15**	H	(*R*)-Ph	ND	**G7**	51
6	**4**	(*S*)-**16**	H	(*S*)-CH_2CHMe_2	ND	**H6**	86
7	**4**	(*R*)-**16**	H	(*R*)-CH_2CHMe_2	ND	**G8**	83[e]
8	**5a**	**12**	H	H	30:70[f]	**I6 + J3**	94[g]
9	**5a**	(*S*)-**14**	H	(*S*)-CH_2Ph	ND	**J4**	91
10	**5a**	(*R*)-**14**	H	(*R*)-CH_2Ph	ND	**I7**	72[e]
11	**5a**	(*S*)-**15**	H	(*S*)-Ph	ND	**J5**	51
12	**5a**	(*R*)-**15**	H	(*R*)-Ph	ND	**I8**	56[e]
13	**5a**	(*S*)-**16**	H	(*S*)-CH_2CHMe_2	ND	**J6**	86
14	**5a**	(*R*)-**16**	H	(*R*)-CH_2CHMe_2	ND	**I9**	68

[a]See Supplementary Methods for reaction protocols. [b]Isolated yields of only the major regioisomer, unless otherwise noted. [c]Ratio determined by ^{1}H NMR of final compound. [d]Isolated yield of a mixture of regioisomers. [e]Minor regioisomer was observed in ^{1}H NMR of final compound. [f]Ratio determined by analytical HPLC of crude reaction mixture. [g]Isomeric mixture was separated to give 27% isolated yield of **I6** and 52% isolated yield of **J3**.

Supplementary Table 6. Three-component reactions of functionalized 3-azasteroids **C8–C15** and **E3–E5**, and 4-azasteroids **D8–D15** and **F3–F5**.[a]

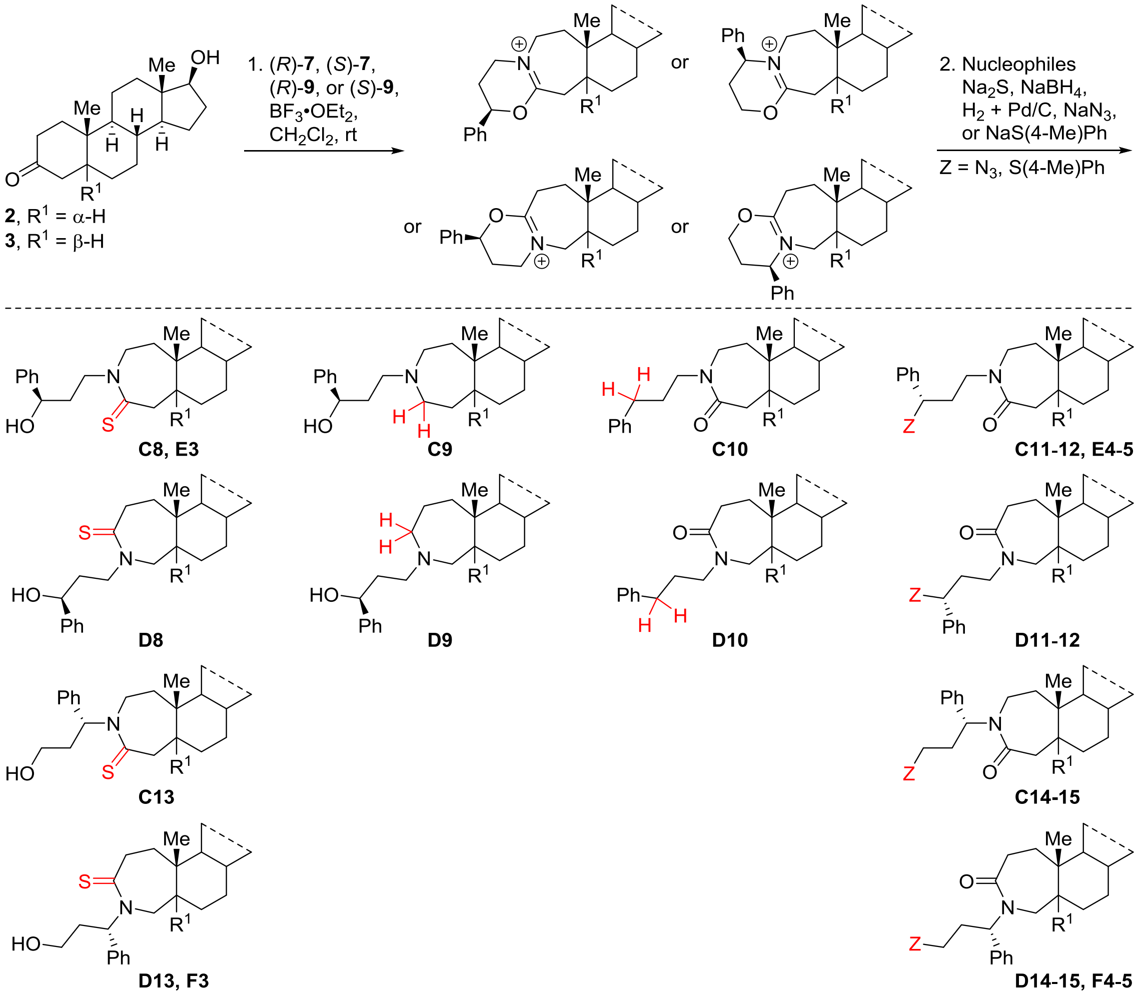

entry	steroid	azide	isolated yield (%)				
						Z	
			S	H		N_3	(4-Me)SPh[b]
1	**2**	(*R*)-**7**	85, **C8**	87, **C9**[c,d]	92, **C10**[e]	56, **C11**[f]	66, **C12**[f]
2	**2**	(*S*)-**7**	45, **D8**	50, **D9**[c,g]	93, **D10**[e]	73, **D11**[f]	67, **D12**[f]
3	**2**	(*R*)-**9**	59, **D13**	-		65, **D14**	83, **D15**
4	**2**	(*S*)-**9**	80, **C13**	-		60, **C14**	65, **C15**
5	**3**	(*S*)-**7**	62, **E3**	-		82, **E4**[f]	56, **E5**[f]
6	**3**	(*S*)-**9**	78, **F3**	-		84, **F4**	76, **F5**

[a]See Supplementary Methods for reaction protocols. [b]0.66 M stock solution of sodium 4-methyl thiophenoxide was prepared immediately prior to use by adding sodium (1.0 equiv) to a solution of 4-methylbenzenethiol (1.1 equiv) in anhydrous DMF (15.0 mL) at 0 ˚C and stirring at room temperature overnight. [c]$NaBH_4$, MeOH. [d]51% isolated yield of **C9** was also obtained from LAH reduction of **C2**. [e]Hydrogenation using 10% Pd/C, EtOH. [f]Inversion of stereochemistry. [g]58% isolated yield of **D9** was also obtained from LAH of **D2**.

Supplementary Table 7. Three-component reactions of functionalized 17-aza-*D*-homo-steroids **G9–G17** and **I10–I16**.[a]

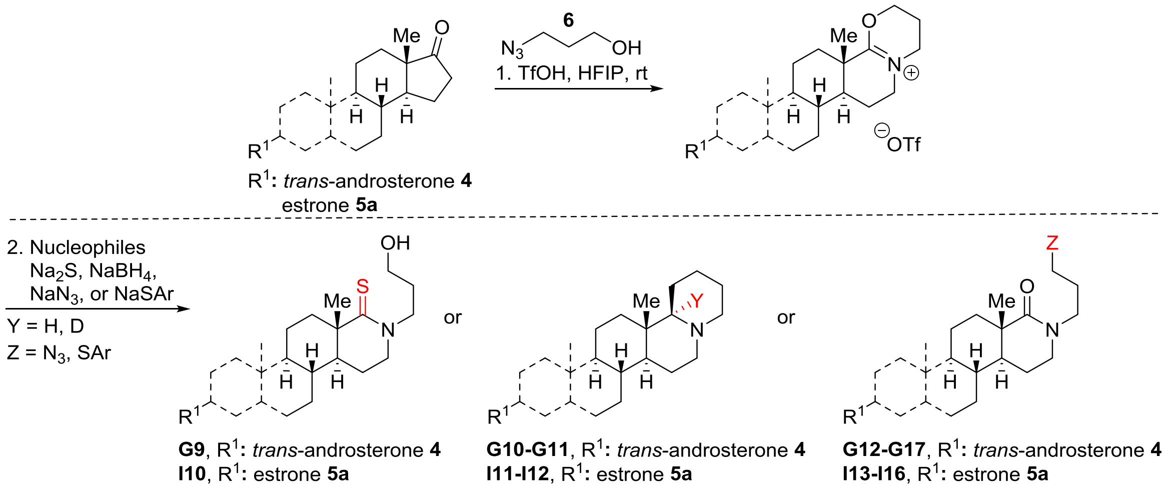

entry	steroid	nucleophile	S, Y or Z	product	isolated yield (%)
1	**4**	Na_2S	S	**G9**	52
2	**4**	$NaBH_4$	H	**G10**	85
3	**4**	$NaBD_4$	D	**G11**	64
4	**4**	NaN_3	N_3	**G12**	86
5	**4**	NaSAr	SC_6H_5[b-c]	**G13**	71
6	**4**	NaSAr	S(4-Me)C_6H_4[b-c]	**G14**	76
7	**4**	NaSAr	S(4-OMe)C_6H_4[b,d]	**G15**	65
8	**4**	NaSAr	S(4-Cl)C_6H_4[b,e]	**G16**	69
9	**4**	NaSAr	S(4-Br)C_6H_4[b,f]	**G17**	61
10	**5a**	Na_2S	S	**I10**	51
11	**5a**	$NaBH_4$	H	**I11**	83
12	**5a**	$NaBH_4$	D	**I12**	82
13	**5a**	NaN_3	N_3	**I13**	82
14	**5a**	NaSAr	SC_6H_5[b-c]	**I14**	62
15	**5a**	NaSAr	S(4-Me)C_6H_4[b-c]	**I15**	60
16	**5a**	NaSAr	S(4-OMe)C_6H_4[b,d]	**I16**	63

[a]See Supplementary Methods for reaction protocols. [b]Stock solutions of sodium thiophenoxides were prepared immediately prior to use by adding sodium (1.0 equiv) to a solution of thiophenols (1.1 equiv) in anhydrous DMF (15.0 mL) at 0 ˚C and stirring at room temperature overnight. [c]0.66 M stock solution. [d]0.67 M stock solution. [e]0.70 M stock solution. [f]0.56 M stock solution.

Supplementary Discussion

General mechanism of Schmidt reactions enabled by hydroxyalkyl azides[2,3]

This reaction has been extensively studied by experimental and computational methods. For general information, see a relevant review[1]. The specific features of this reaction relevant to the present project are noted here for the reader's convenience. Following this description, specific details for selected examples are provided.

The reaction begins with attack of the alcohol group onto the carbonyl reactant, followed by elimination to afford an oxonium ion intermediate. The attached azide attacks the oxonium ion to afford a spirocyclic intermediate, which undergoes migration to provide an iminium ether. This intermediate can be isolated but is usually reacted *in situ* with a nucleophile to provide an *N*-substituted lactam product.

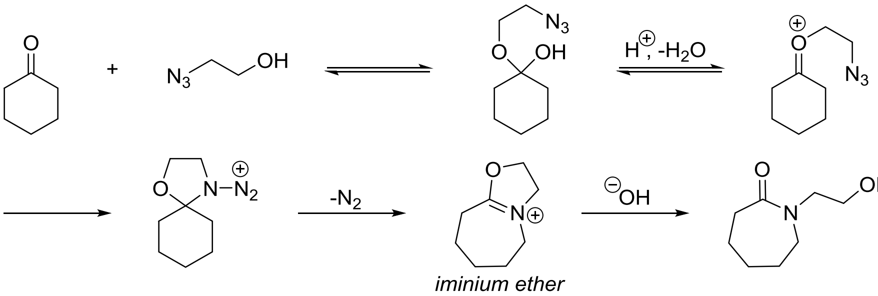

Supplementary Figure 1. General mechanism of ring expansions using hydroxyalkyl azides.

Factors that determine reaction regio- and stereochemistry

Most of the work pertaining to the regiochemical outcome of this reaction was carried out in the context of asymmetric ring expansions of achiral cyclohexanones to diastereoselectively make substituted caprolactams. In brief, the outcomes of these reactions depend on three considerations. They are the direction of azide attack relative to pre-existing substitution on the ketone reactant, selective formation and reaction of the most stable new heterocyclic ring (1,3-oxazinane from 3-azidopropanol or oxazoline from 2-azidoethanol), and antiperiplanar C→N migration to afford the iminium ether product. Importantly, all steps leading to the actual migration are reversible on the reaction time scale and therefore thermodynamically controlled. This is supported by DFT calculations – the barrier for C→N migration is only ca. 2 kcal/mol higher than reversion to azide and oxonium ion[4].

A representative case is shown and annotated below; this discussion will focus on the better-understood reactions of substituted cyclohexanones with 3-azidopropanols, but similar considerations apply to other versions as well. Overall, one can consider this complex process to arise from a combination of three stereochemical and conformational factors.

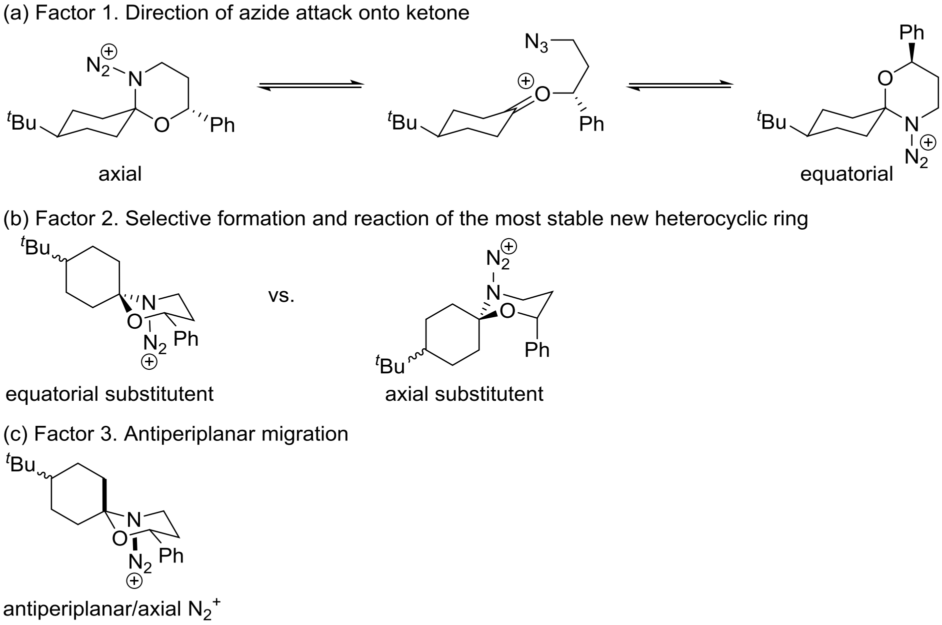

Supplementary Figure 2. The three determinants of stereo- or regioselectivity in ring expansion reactions mediated by chiral hydroxyalkyl azides. **a,** In intermediates derived from six-membered rings, equatorial attack onto the more stable cyclohexanone derivative has been proposed and supported by computation[4]; the stereoselectivity is related to the relative populations of the possible conformations of the starting ketones (typically in chair conformations). Attack onto ketones of other ring sizes depend on steric accessibility (see specific examples below). Practically, since it is not possible to directly observe the spirocyclic intermediate, the direction of attack in non-obvious cases may need to be inferred from analyzing the outcomes in the context of considerations 2 and 3 below (see, for example, discussion of C-17 reactivity below in the SI). **b,** For intermediates derived from monosubstituted 3-azidopropanols, the major products arise from spirocyclic 1,3-oxazinanes containing the carbon substituent in an equatorial orientation. It has been reported[4] that 2-aryl- or 2-alkoxy 3-azidopropanols lead to greater quantities of product arising from spirocyclic 1,3-oxazinanes having the aryl group in an axial position (due to a π–cation interaction between the aryl group and the N_2^+ group, which is now in a potentially 1,3-diaxial relationship), but that is not relevant in this paper since we did not explore any hydroxyalkyl azides of these types in this study. In all cases, the 1,3-oxazinane ring is presumed to adopt the most stable chair-like conformation, recognizing that the two chair forms can interconvert either by reversion to oxonium ion and azide or by ring flip between the two forms. **c,** All known C→N migrations in azido-Schmidt reactions entail concerted antiperiplanar migration to the N_2^+ leaving group[4]. For the spirocyclic intermediates arising from hydroxylalkyl azides, this means that the reaction coordinate necessarily goes through an axial N_2^+ group, since when this group is equatorial, the only antiperiplanar options are a C–O bond (very unlikely to migrate, as it would form a new, weak N–O bond) or a C–C bond that, if broken, would lead to a cation unstabilized by a neighboring oxygen atom. In general, the N_2^+ group is axial in the ground-state conformation as revealed by DFT calculations[4].

Specific cases are discussed on the following pages to illustrate the above principles.

Regioselectivity for 5α-substituted steroids

In this section, we provide one example of a reaction of each of three substituted 3-azidopropanol reagents, in both enantiomeric forms, with 3-oxosteroid **2**.

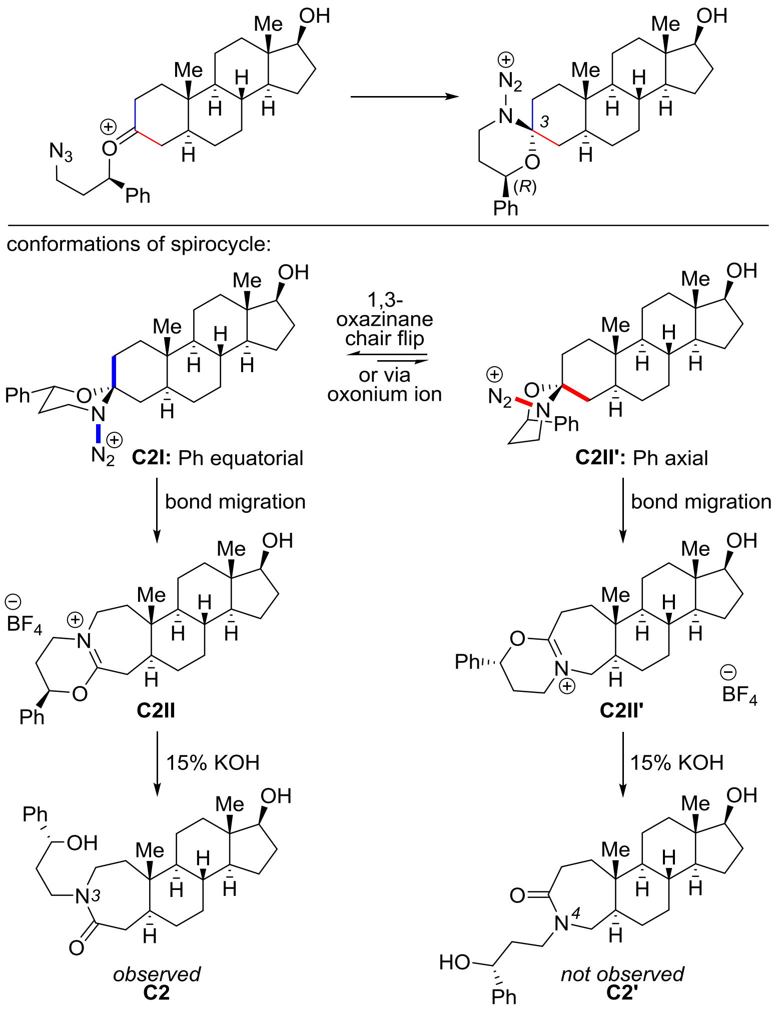

Supplementary Figure 3. Proposed mechanism for the regioselectivity of 3-oxosteroid **2** with (*R*)-3-azido-1-phenylpropanol (*R*)-**7**. In this example, β/equatorial attack by the azide is preferred and the conformation of the newly-formed 1,3-oxazinanne ring is anchored by the 1-phenyl substituent. This leads to migration of the C2 carbon and affords isomer **C2**.

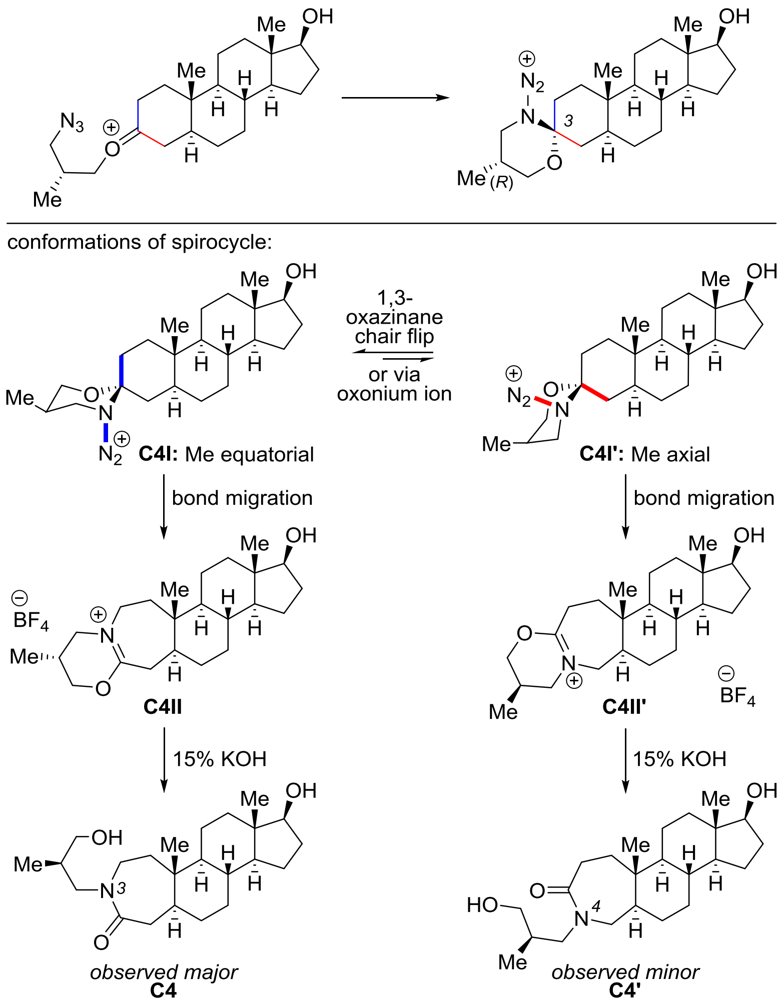

Supplementary Figure 4. Proposed mechanism for the regioselectivity of 3-oxosteroid **2** with (*R*)-3-azido-2-methylpropanol (*R*)-**8**. In this example, β/equatorial attack by the azide is preferred and the conformation of the newly-formed 1,3-oxazinane ring is anchored by the 2-methyl substituent. This leads to migration of the C2 carbon and affords isomer **C4**.

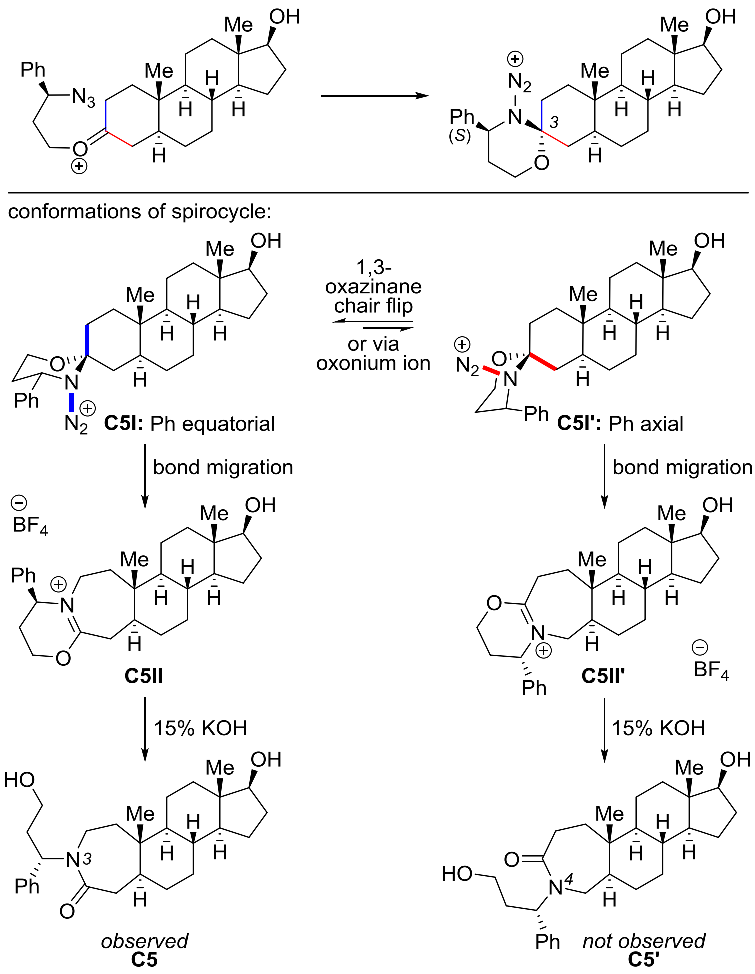

Supplementary Figure 5. Proposed mechanism for the regioselectivity of 3-oxosteroid **2** with (*S*)-3-azido-3-phenylpropanol (*S*)-**9**. In this example, β/equatorial attack by the azide is preferred and the conformation of the newly-formed 1,3-oxazinane ring is anchored by the 3-phenyl substituent. This leads to migration of the C2 carbon and affords isomer **C5**.

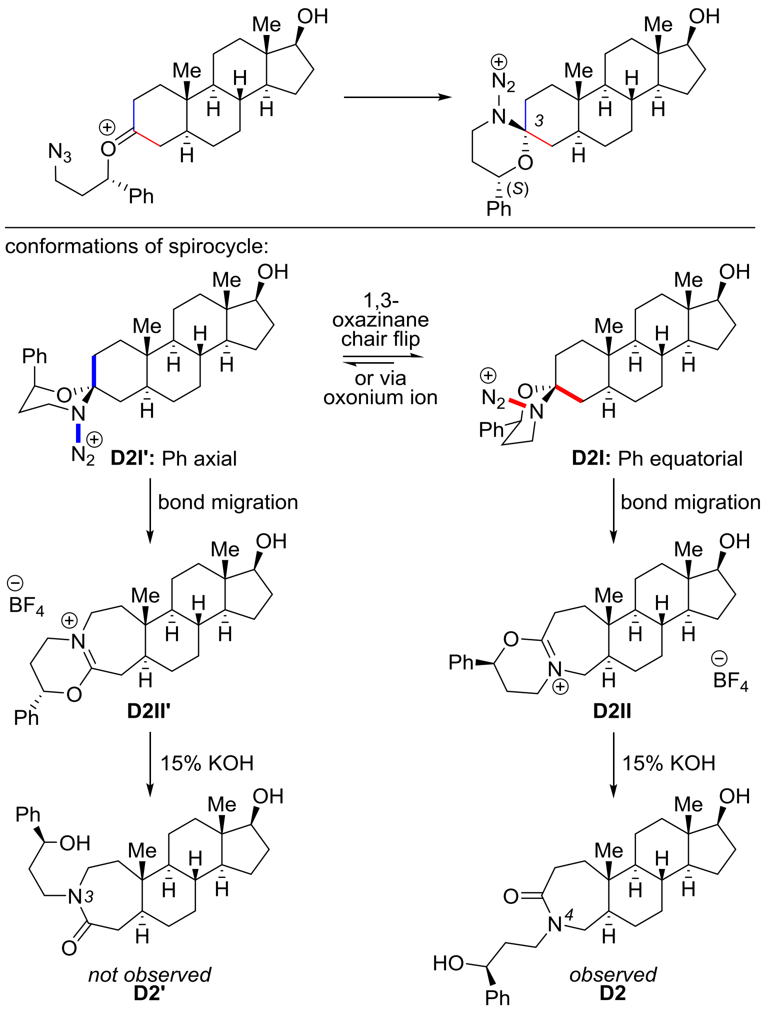

Supplementary Figure 6. Proposed mechanism for the regioselectivity of 3-oxosteroid **2** with (*S*)-3-azido-1-phenylpropanol (*S*)-**7**. In this example, β/equatorial attack by the azide is preferred and the conformation of the newly-formed 1,3-oxazinane ring is anchored by the 1-phenyl substituent. This leads to migration of the C4 carbon and affords isomer **D2**.

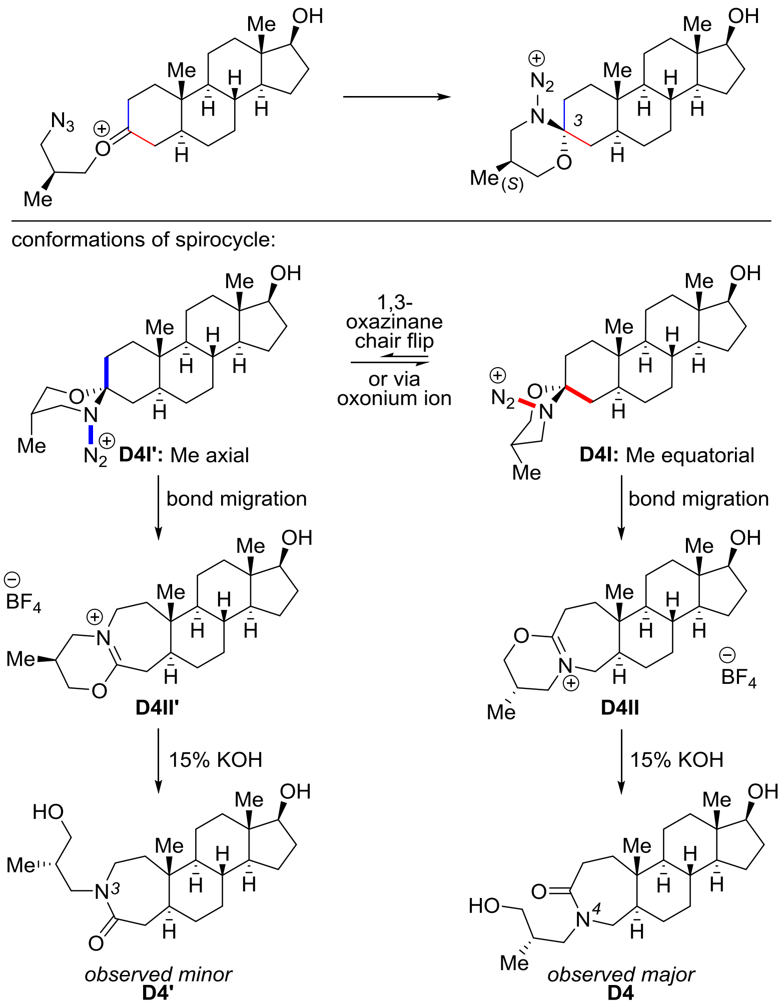

Supplementary Figure 7. Proposed mechanism for the regioselectivity of 3-oxosteroid **2** with (*S*)-3-azido-2-methylpropanol (*S*)-**8**. In this example, β/equatorial attack by the azide is preferred and the conformation of the newly-formed 1,3-oxazinane ring is anchored by the 2-methyl substituent. This leads to migration of the C4 carbon and affords isomer **D4**.

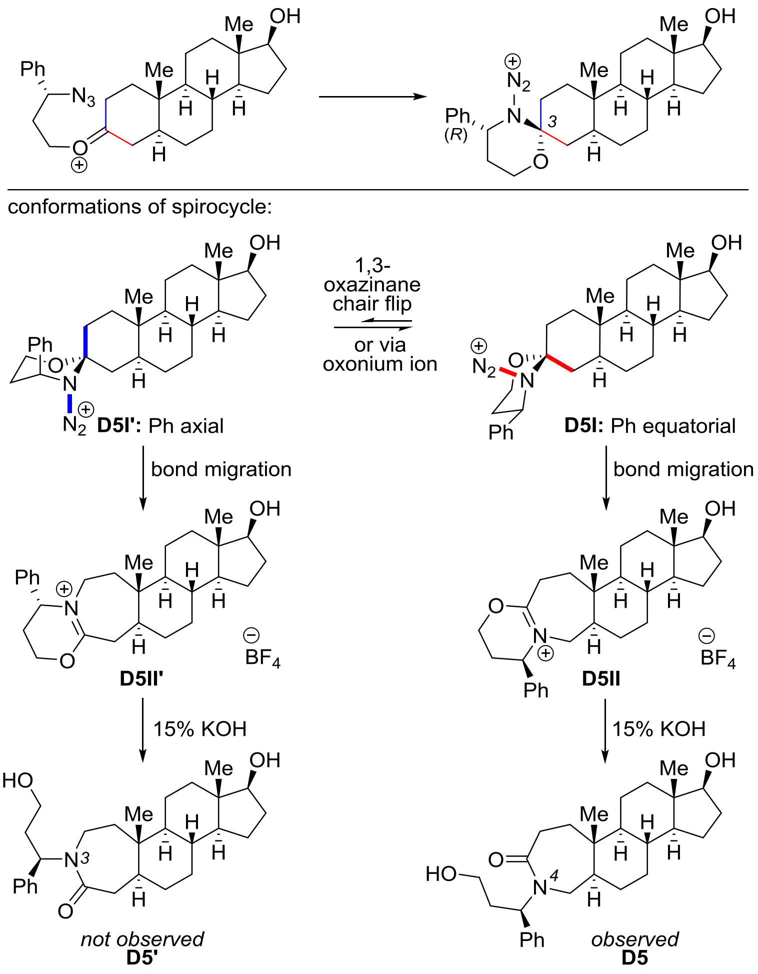

Supplementary Figure 8. Proposed mechanism for the regioselectivity of 3-oxosteroid **2** with (*R*)-3-azido-3-phenylpropanol (*R*)-**9**. In this example, β/equatorial attack by the azide is preferred and the conformation of the newly-formed 1,3-oxazinane ring is anchored by the 3-phenyl substituent. This leads to migration of the C4 carbon and affords isomer **D5**.

Regioselectivity for 5β-substituted steroids

In this section, we provide two examples to show how regiochemistry differs in response to changing the steroid configuration at C5; compare these examples with those in Supplementary Figure 3 and 6.

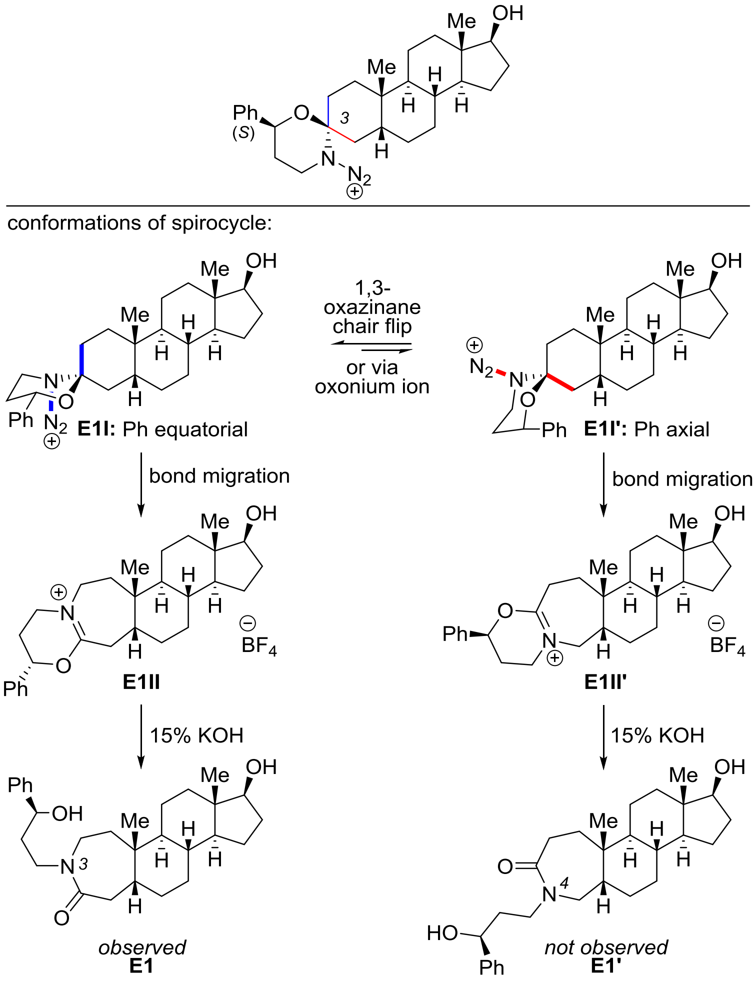

Supplementary Figure 9. Proposed mechanism for the regioselectivity of 3-oxosteroid **3** with (*S*)-3-azido-1-phenylpropanol (*S*)-**7**. In this example, α/equatorial attack by the azide is preferred and the conformation of the newly-formed 1,3-oxazinane ring is anchored by the 1-phenyl substituent. This leads to migration of the C2 carbon and affords isomer **E1**.

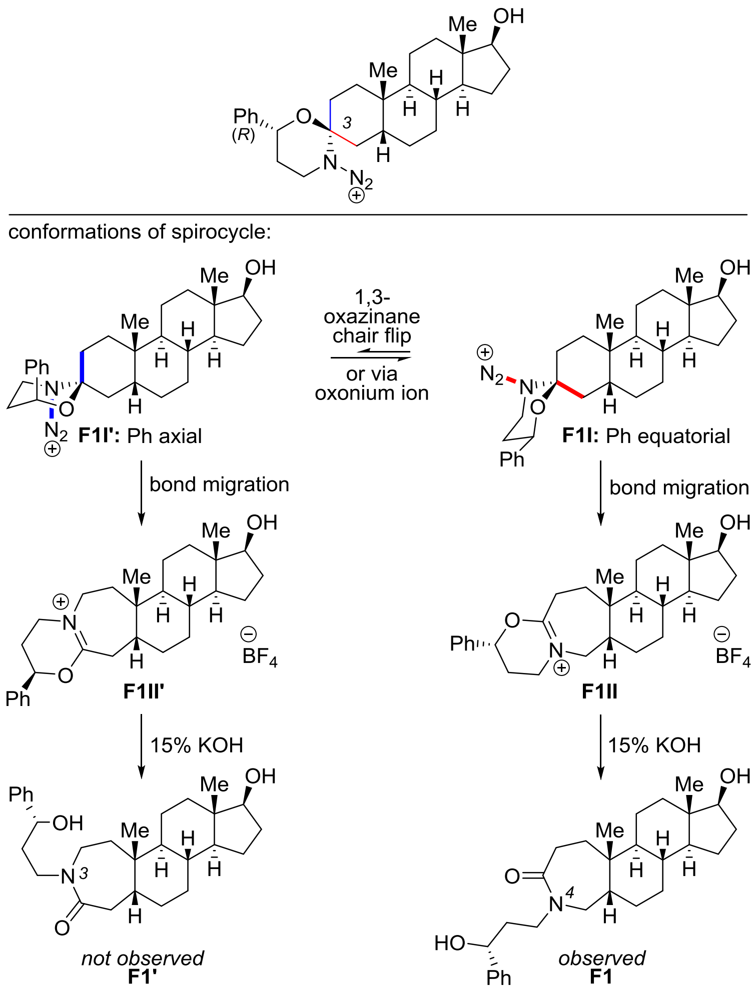

Supplementary Figure 10. Proposed mechanism for the regioselectivity of 3-oxosteroid **3** with (*R*)-3-azido-1-phenylpropanol (*R*)-**7**. In this example, α/equatorial attack by the azide is preferred and the conformation of the newly-formed 1,3-oxazinane ring is anchored by the 1-phenyl substituent. This leads to migration of the C4 carbon and affords isomer **F1**.

Regioselectivity in 17-oxosteroid ring expansions

In this section, we include descriptions of all of the reported approaches to control ring expansions of 17-oxosteroids, namely the Beckmann rearrangement, the intramolecular Schmidt reaction, and the reactions of 17-oxosteroids with 3-azidopropanols (both unsubstituted and substituted). For the last, a detailed description of how we assigned α vs. β azide addition to the ketone has been included.

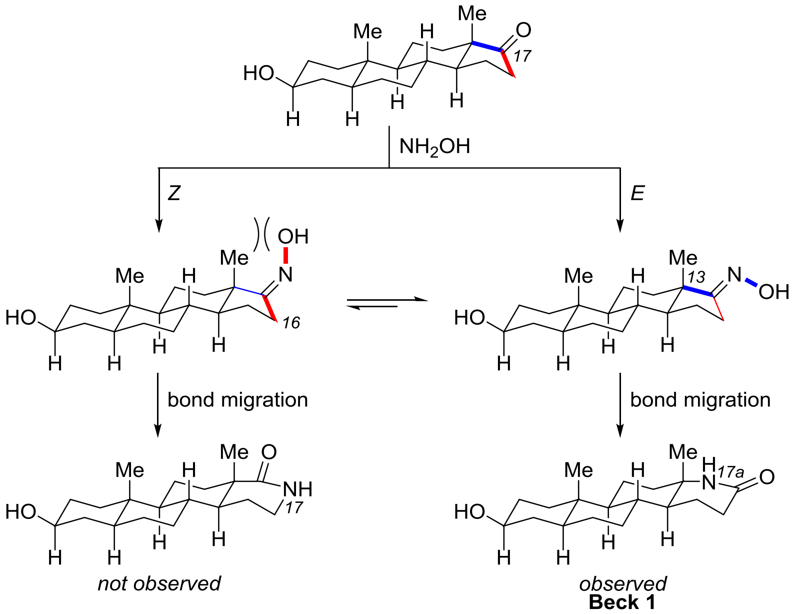

Supplementary Figure 11. Beckmann rearrangement at C-17 in *trans*-androsterone **4**. The mechanism of the Beckmann rearrangement has long been established[5] and shown to occur through selective reaction of the sterically least hindered oxime stereoisomer (here, the E isomer) and migration of the *trans*-antiperiplanar bond as depicted.

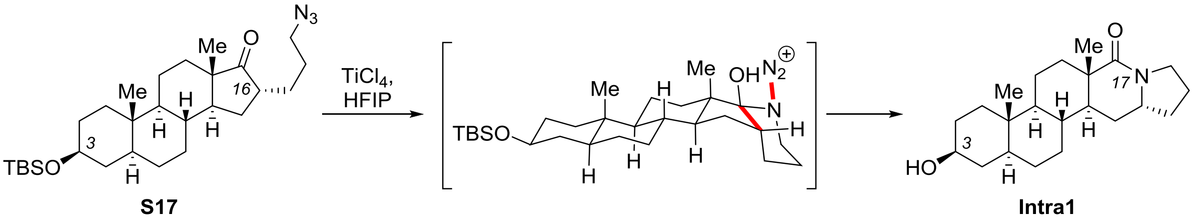

Supplementary Figure 12. Intramolecular Schmidt reaction. The intramolecular Schmidt reaction of a tethered azido ketone entails formation of the most stable ring fusion upon azide addition to the activated (protonated) ketone and subsequent migration of a C–C bond antiperiplanar to the N_2^+ leaving group. In these reactions, the leaving group generally occupies an equatorial position and migration of the ring fusion carbon ensues. Under special conditions, axial N_2^+ is possible, leading to bridged adducts, but these cases are rare[6]. In the case shown, α-addition to C-17 is proposed because *cis* [4.3.0] bicyclic ring systems are generally preferred over the *trans* version, but the same outcome would be predicted from either direction of attack.

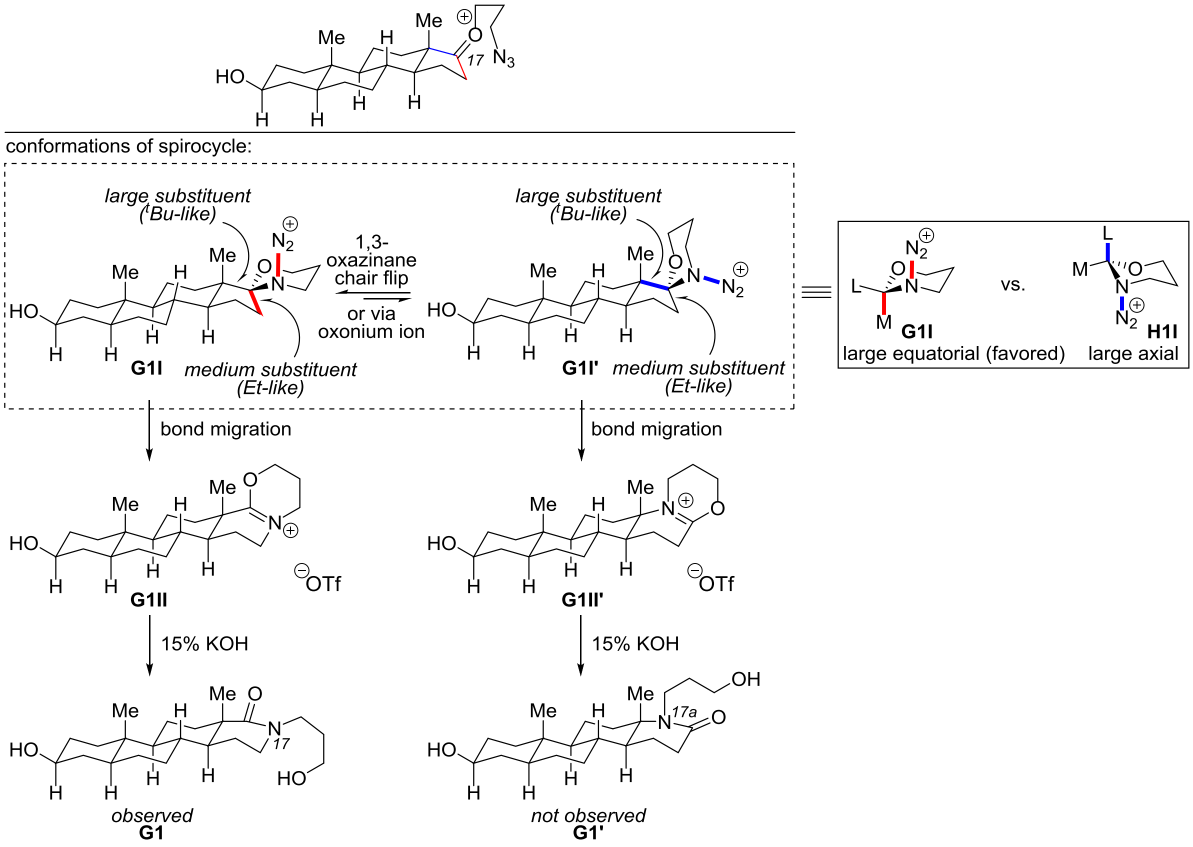

Supplementary Figure 13. Regiochemistry of D-ring expansion reaction with 3-azidopropanol **6**. This reaction may occur from a combination of two stereochemical/conformational considerations, neither of which is obvious based on precedent. These are α vs. β attack onto the C-17 oxonium ion and the formation of a particular 1,3-oxazinane ring chair conformation. All possible transformations are assumed to involve antiperiplanar migration to an axial N_2^+ leaving group as no exceptions to this arrangement are currently known (see general mechanistic comments above).

The newly-formed 1,3-oxazinane ring can exist in two conformations as shown. This spirocyclic center is attached to C16 and C13 of the steroid; the former is ethyl-like and the latter fully substituted, and therefore *tert*-butyl-like. Accordingly, C13 should occupy an equatorial position in preference to the smaller C16 group as shown in the scheme above.

The matter of α vs. β attack is less obvious because in each case described above there is a stable chair conformation that would lead to the observed stereochemistry, as shown:

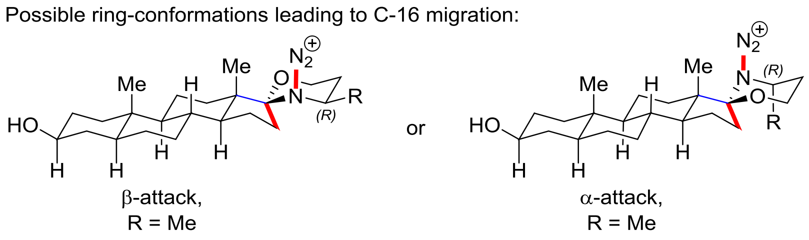

The literature is silent on the preference of intramolecular spirocycle formation in cases like these (as opposed to intermolecular organometallic additions, which prefer α attack away from the C18 methyl group). However, C-16 migration was observed when (*R*)-3-azidobutanol (*R*)-**10** was used in the reaction (Supplementary Figure 14), which is only consistent with β-attack of the azide (in this case, R

= Me in the above diagram). Moreover, the opposite regiochemistry was observed when (*S*)-3-azidobutanol (*S*)-**10** was used in the reaction (Supplementary Figure 15), also only consistent with β attack in that system. For those reasons, we propose that β-attack is uniformly observed in all of the spirocycles formed upon attack at C-17 by these reagents.

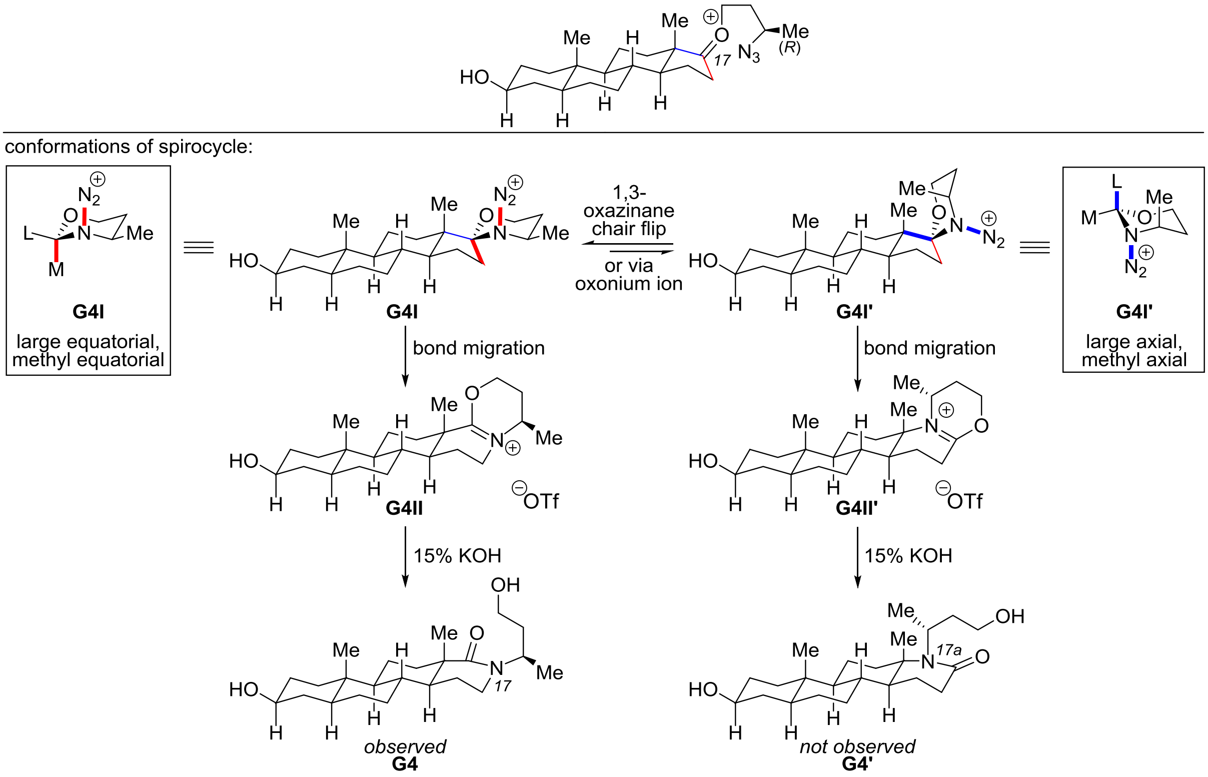

Supplementary Figure 14. Proposed mechanism for regioselectivity of 17-oxosteroid **4** with (*R*)-3-azidobutanol (R)-**10**. In this example, the (*R*)-methyl group and the placement of the large C-18 carbon into an equatorial substituent clearly favor the conformation of the 1,3-oxazinane shown in **G4I** and experimentally was shown to lead to isomer **G4** resulting from migration of C-16. Together, these facts support the assignment of β attack onto the C-17 oxonium ion as shown.

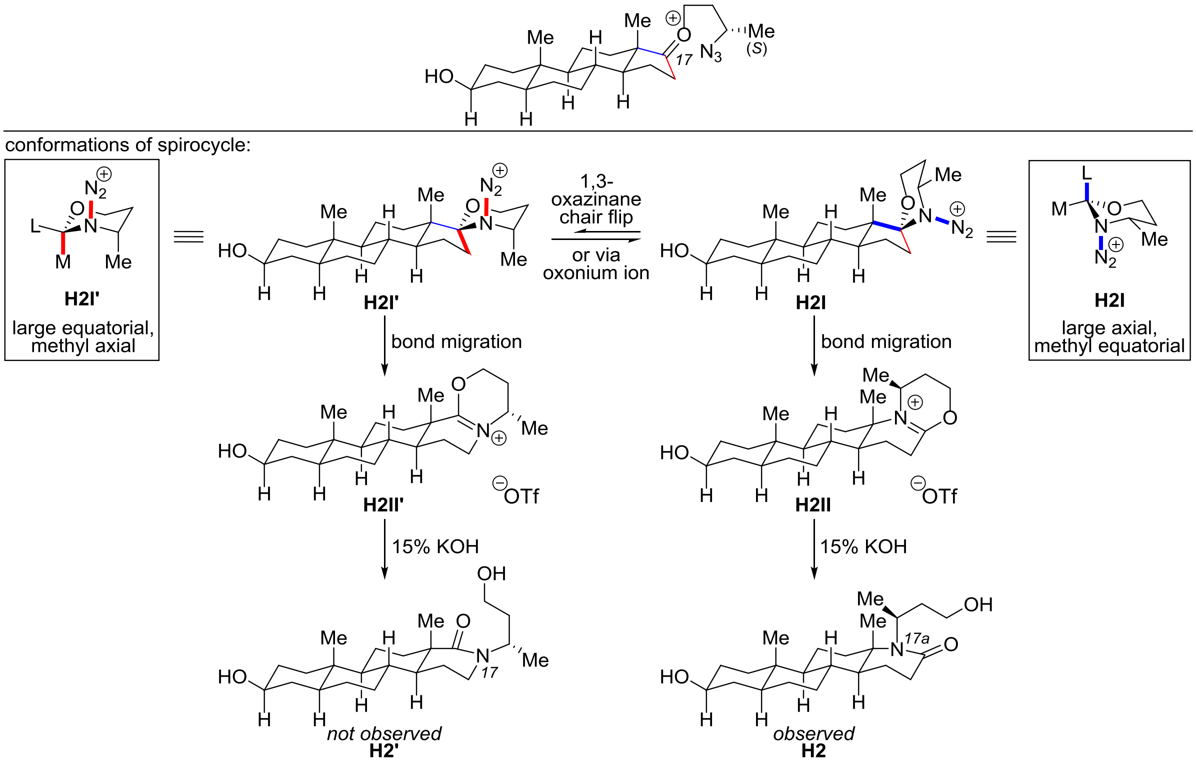

Supplementary Figure 15. Proposed mechanism regioselectivity of 17-oxosteroid **4** with (*S*)-3-azidobutanol (*S*)-**10**. In this example, the (*S*)-methyl group and the placement of the large C-18 carbon into an equatorial substituent clearly favor the conformation of the 1,3-oxazinane shown in **H2I** and experimentally was shown to lead to isomer **H2** resulting from migration of C-18. Together, these facts support the assignment of β attack onto the C-17 oxonium ion as shown.

Supplementary Methods

Caution: Although we have not experienced any untoward events with the compounds mentioned in this paper, azides and their precursors are known explosive hazards and should be used with appropriate safety precautions. Minimally, careful control of temperature and scale should be exercised. We do not recommend distillation of reaction mixtures that may contain residues of azide sources.

Reactions were performed under inert atmosphere (argon or nitrogen). Reactions were carried out in either flame-dried round bottom flasks or glass sample vials (TFE-lined cap). All chemicals were purchased from commercial sources and used without further purification. New containers of BF_3•OEt_2, TfOH, and HFIP were used. Anhydrous CH_2Cl_2, MeOH, DMF and THF were purchased from Sigma-Aldrich and used as received. Thin-layer chromatography (TLC) was performed using commercial glass-backed silica plates (250 µM) with an organic binder. Visualization was accomplished with UV light, Seebach's stain or aqueous $KMnO_4$ stain and heating. Purification was carried out by an automated flash chromatography/medium-pressure liquid chromatography (MPLC) system using normal phase silica gel flash columns (4, 12, 24, 40, or 80 g).

The infrared (IR) spectra were acquired as thin films using a universal ATR sampling accessory either on a PerkinElmer Spectrum One FT-IR spectrometer, Thermo Scientific Nicolet iS5 FT-IR spectrometer, or Bruker Alpha FT-IR spectrometer; the absorption frequencies are reported in cm^{-1}. All nuclear magnetic resonance spectra were recorded on a 400 MHz, 500 MHz with a dual carbon/proton cyroprobe, or 600 MHz with a dual carbon/proton cryoprobe instrument; Varian and Bruker instruments were used. NMR samples were recorded in deuterated chloroform ($CDCl_3$) or deuterated dimethylsulfoxide (DMSO-d_6). Chemical shifts are reported in parts per million (ppm) and referenced to the center line of solvent ($CDCl_3$: δ 7.26 ppm for ^{1}H NMR and 77.16 for ^{13}C NMR; DMSO-d_6 δ 2.50 ppm for ^{1}H NMR and 39.52 for ^{13}C NMR). Coupling constants are given in hertz (Hz). Parameters for 1D NOESY and 2D-NMR sequences include. (1) Varian NOESY1D pulse sequence entails nucleus: 1H, number of scans: 64 or 256, receiver gain: 30 or 46, relaxation delay: 1.0, pulse width: 8.8, 9.2 or 11.0, spectrometer frequency: 399.7, spectral width: 6410.3, lowest frequency: -799.8 or -806.8. (2) Varian HSQCAD pulse sequence entails nucleus: (1H, 13C), number of scans: 4 or 8, receiver gain: 30, relaxation delay: 1.0, pulse width: 8.8 or 9.2, spectrometer frequency: (399.7, 100.5), spectral width: (6410.3, 20100.5), lowest frequency: (-806.8, -1004.7). (3) Bruker HSQCAD pulse sequence entails nucleus: (1H, 13C), number of scans: 2 or 4, receiver gain: 2050 or 3649, relaxation delay: 2.0, pulse width: 16.0, spectrometer frequency: (500.2, 125.8), spectral width: (8012.8, 20836.7), lowest frequency: (-1662.0, -1599.9). (4) Varian gHMBCAD pulse sequence entails nucleus: (1H, 13C), number of scans: 8, 16 or 32, receiver gain: 30, relaxation delay: 1.0, pulse width: 8.8, spectrometer frequency: (399.7, 100.5), spectral width: (6410.3, 24118.2), lowest frequency: (-806.8, -1505.8). (5) Varian gCOSY pulse sequence entails nucleus: (1H, 1H), number of scans: 5, receiver gain: 48, relaxation delay: 1.0, pulse width: 8.8, spectrometer frequency: (399.7, 399.7), spectral width: (3787.9, 3787.9), lowest frequency: (-379.5, -379.5). (6) Bruker cosygpgf pulse sequence entails nucleus: (1H, 1H), number of scans: 1 or 2, receiver gain: 32 or 90, relaxation delay: 1.5, pulse width: 16.0, spectrometer frequency: (500.2, 500.2), spectral width: (6684.5, 6677.7), lowest frequency: (-335, -331.6).

HRMS data were collected using two instruments. (1) Time-of-flight mass spectrometer (TOF) with an electrospray ion source (ESI). (2) Thermo LTQ Fourier transform ion cyclotron resonance (FT-ICR, 7T) with a heated electrospray ion source (HESI), electrospray ion source (ESI), atmospheric-pressure chemical ionization source (APCI), or atmospheric-pressure photoionization (APPI). Purity data were collected using two instruments. (1) Waters Acquity H-class UPLC-PDA detector coupled to the Thermo LTQ Fourier transform ion cyclotron resonance mass spectrometer (FT-ICR, 7T) with a heated electrospray ion source (HESI). Samples were run on analytical Acquity UPLC BEH 2.1 × 50 mm, 1.7 µm, C18 column, and analytical Acquity UPLC HSS T3, 2.1 × 50 mm, 3.18 µm, C18 column, at 40 ˚C with mobile phases A (H_2O + 0.1% formic acid) and B (MeCN + 0.1% formic acid). (2) Agilent 6110 Series LCMS with a UV detector and a single quadrupole mass spectrometer. Samples were run on an

analytical Agilent Eclipse Plus 4.6 × 50 mm, 1.8 µm, C18 column at room temperature with mobile phases A (H_2O + 0.1% acetic acid) and B (MeOH + 0.1% acetic acid). Melting points were determined in open capillary tubes using OptiMelt, an automated melting point apparatus, and are uncorrected.

Spectroscopic data for known compounds described in the paper match with those reported in the literature. ^{1}H NMR and ^{13}C NMR of known compounds are provided in the Supplementary Figures section.

List of known compounds

The following steroid intermediates and substrates: **S1**[7], **1**[8], **2**[9], **3**[10], **S12**[11], **Beck1**[12], **S13**[13], **S15**[11,14], **5b**[15]
The following hydroxyalkyl azides: **6**[16], (*R*)-**7**[17], (*R*)-**8**[17], (*R*)-**9**[17], **12**[16], (*S*)-**14**[18], (*R*)-**14**[18], (*R*)-**15**[19], (*S*)-**16**[18]

Preparation of steroidal ketones (known compounds)

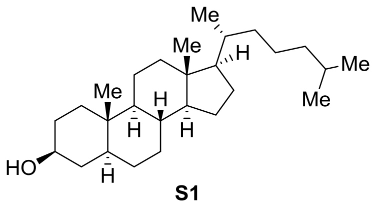

S1

5α-Cholestan-3-ol, S1[7]
To a solution of cholesterol (2.03 g, 5.25 mmol) in anhydrous EtOH (55.0 mL, 0.1 M) was added 10% Pd/C (220 mg, 2.07 mmol, 0.40 equiv) under argon. The reaction mixture was degassed and charged with hydrogen. The reaction mixture was stirred under balloon-pressure hydrogen overnight. The reaction mixture was filtered over Celite and concentrated. Purification was carried out by an automated MPLC system using a 40 g normal phase silica column with gradient elution from 0–10% EtOAc/hexanes to afford **S1** as a white amorphous solid (1.86 g, 4.79 mmol, 91% yield). Characterization data were consistent with reported data.

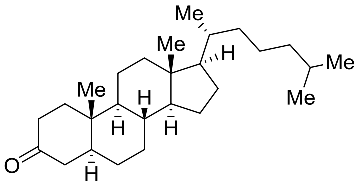

1

5α-Cholestan-3-one, 1[8]
To a solution of **S1** (1.57 g, 4.04 mmol) in anhydrous CH_2Cl_2 (50.0 mL) was added Celite and PCC (1.31 g, 6.07 mmol, 1.5 equiv) at 0 ˚C. The reaction mixture was allowed to room temperature and stirred overnight. The reaction mixture was filtered through a short pad of Celite and concentrated. Purification was carried out by an automated MPLC system using a 40 g normal phase silica column with gradient elution from 0–10% EtOAc/hexanes to afford **1** as a white amorphous solid (1.45 g, 3.75 mmol, 93% yield). Characterization data were consistent with reported data; mp 129–130 ˚C.

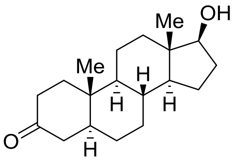

2

5α-Dihydrotestosterone, 2[9]

Following a literature procedure[20], 5α-Dihydrotestosterone **2** was prepared as described. To a solution of testosterone (570 mg, 1.98 mmol) in anhydrous THF (6.0 mL) was condensed liquid NH_3 (~30 mL) at –78 ˚C. To the cooled solution was added pieces of lithium wire (83.0 mg, 12.0 mmol, 6.0 equiv). The blue solution was stirred at –78 ˚C for 20 min, and was allowed to –35 ˚C and stirred for an additional 2 h. The reaction mixture was quenched by adding solid NH_4Cl slowly (until the disappearance of blue). The resulting mixture was diluted with H_2O and EtOAc. The organic layer was washed with brine, dried over anhydrous Na_2SO_4, filtered, and concentrated. Purification was carried out by an automated MPLC system using a 24 g normal phase silica column with gradient elution from 0–30% EtOAc/hexanes to afford **2** as a white amorphous solid (331 mg, 1.14 mmol, 58% yield). Characterization data were consistent with reported data; mp 179–181 ˚C.

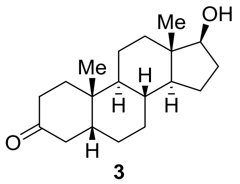

3

5β-Dihydrotestosterone, 3[10]

To a solution of testosterone (2.30 g, 8.00 mmol) in anhydrous THF (80.0 mL, 0.1 M) was added 10% Pd/C (341 mg, 3.20 mmol, 0.40 equiv) under argon. The reaction mixture was degassed and charged with hydrogen. The reaction mixture was stirred under balloon-pressure hydrogen overnight. The reaction mixture was filtered over Celite and concentrated. Purification was carried out by an automated MPLC system using an 80 g normal phase silica column with gradient elution from 0–20% EtOAc/hexanes to afford **3** as a colorless amorphous solid (1.51 g, 5.20 mmol, 65% yield) and **2** as a white amorphous solid (414 mg, 1.43 mmol, 18% yield). Characterization data were consistent with reported data.

Preparation of hydroxyalkyl azides

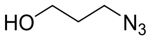

6

3-Azidopropanol, 6[16]

3-Azidopropanol **6** was prepared following a previously published procedure. Characterization data were consistent with reported data.

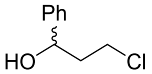

S2

(±)-3-Chloro-1-phenylpropanol, S2[21]

(±)-3-Chloro-1-phenylpropanol **S2** was prepared following a previously published procedure. Characterization data were consistent with reported data.

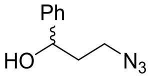

(±)-7

(±)-3-Azido-1-phenylpropanol, (±)-7

Following a literature procedure[17], (±)-3-azido-1-phenylpropanol **(±)-7** was prepared as described. A mixture of **(±)**-3-chloro-1-phenylpropanol **S2** (681 mg, 3.99 mmol), NaN_3 (780 mg, 12.0 mmol, 3.0

equiv), NaI (899 mg, 6.00 mmol, 1.5 equiv) in anhydrous DMF (40.0 mL) was stirred for 24 h at 80 ˚C. The reaction mixture was partitioned between Et_2O and water. The organic layer was washed with brine, dried over anhydrous Na_2SO_4, filtered, and concentrated under a stream of nitrogen. The crude oil was purified by an automated MPLC system using a 24 g normal phase silica column with gradient elution of 0–15% EtOAc/hexanes. Concentration of solvents under nitrogen afforded racemic product as a colorless oil (619 mg, 3.50 mmol, 88% yield). R_f = 0.25 (10% EtOAc/hexanes); IR (neat) 3378, 2089 cm^{-1}; ^1H NMR (400 MHz, $CDCl_3$) δ 7.40–7.28 (m, 5H), 4.85 (m, 1H), 3.54–3.36 (m, 2H), 2.09–1.91 (m, 3H); ^{13}C NMR (101 MHz, $CDCl_3$) δ 144.0, 128.8, 128.1, 125.9, 72.0, 48.5, 38.0. HRMS (TOF, ESI) *m/z*: $[M – N_2 + H]^+$ calcd for $C_9H_{12}NO$ 150.0913, found 150.0898. HPLC: Chiralcel OD-H, Daicel Chemical Industries, Ltd.; 2–40% *i*-PrOH/hexanes over 60 min; flow rate 1.0 mL/min; UV 220 nm; (*S*) t_R = 10.29 min, (*R*) t_R = 11.03 min.

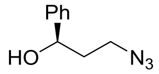

(*R*)-**7**

(*R*)-3-Azido-1-phenylpropanol, (*R*)-7[17]

(*R*)-3-Azido-1-phenylpropanol (*R*)-**7** was prepared following a previously published procedure. Characterization data were consistent with reported data.

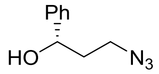

(*S*)-**7**

(*S*)-3-Azido-1-phenylpropanol, (*S*)-7

Following a literature procedure[17], (*S*)-3-azido-1-phenylpropanol (*S*)-**7** was prepared as described. A mixture of (*S*)-3-chloro-1-phenylpropanol (859 mg, 5.03 mmol), NaN_3 (975 mg, 15.0 mmol, 3.0 equiv), NaI (1.12 g, 7.50 mmol, 1.5 equiv) in anhydrous DMF (45.0 mL) was stirred for 24 h at 80 ˚C. The reaction mixture was partitioned between Et_2O and water. The organic layer was washed with brine, dried over anhydrous Na_2SO_4, filtered, and concentrated under a stream of nitrogen. The crude oil was purified by an automated MPLC system using a 24 g normal phase silica column with gradient elution of 0–15% EtOAc/hexanes. Concentration of solvents under nitrogen afforded product as a colorless oil (797 mg, 4.50 mmol, 89% yield) in ≥99.5% ee as determined by analytical HPLC. R_f = 0.25 (10% EtOAc/hexanes); IR (neat) 3373, 2092 cm^{-1}; $[\alpha]_D^{23}$ = –35.9 (*c* 1.09, $CHCl_3$); ^1H NMR (400 MHz, $CDCl_3$) δ 7.40–7.28 (m, 5H), 4.84 (dd, *J* = 8.4, 4.7, 1H), 3.54–3.36 (m, 2H), 2.09–1.91 (m, 3H); ^{13}C NMR (101 MHz, $CDCl_3$) δ 144.0, 128.8, 128.1, 125.9, 72.0, 48.5, 38.0. HRMS (TOF, ESI) *m/z*: $[M – N_2 + H]^+$ calcd for 150.0913, found 150.0895. HPLC: Chiralcel OD-H, Daicel Chemical Industries, Ltd.; 2–40% *i*-PrOH/hexanes over 60 min; flow rate 1.0 mL/min; UV 220 nm; (*S*) t_R = 10.29 min, (*R*) t_R = 11.03 min.

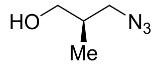

(*R*)-**8**

(*R*)-3-Azido-2-methylpropanol, (*R*)-8[17]

(*R*)-3-Azido-2-methylpropanol (*R*)-**8** was prepared following a previously published procedure at an elevated temperature of 45 ˚C for 24 h. Characterization data were consistent with reported data.

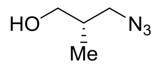

(*S*)-**8**

(*S*)-3-Azido-2-methylpropanol, (*S*)-8

Following a literature procedure, (*S*)-3-azido-2-methylpropanol (*S*)-**8** was prepared as described. A mixture of (*R*)-3-bromo-2-methylpropanol (310 µL, 2.96 mmol) and NaN_3 (975 mg, 15.0 mmol, 5.0 equiv) in anhydrous DMF (8.0 mL) was stirred for 24 h at 45 ˚C. The reaction mixture was partitioned between Et_2O and H_2O. The organic layer was washed with H_2O, brine, dried over anhydrous Na_2SO_4, filtered, and concentrated under a stream of nitrogen. The crude oil was purified by an automated MPLC system using a 24 g normal phase silica column with gradient elution of 0–25% EtOAc/hexanes. Concentration of solvents under nitrogen afforded product as a colorless oil (315 mg, 2.74 mmol, 91% yield). R_f = 0.30 (25% EtOAc/hexanes); IR (neat) 3341, 2092 cm^{-1}; $[\alpha]_D^{23}$ = –5.98 (*c* 1.00, $CHCl_3$); ^1H NMR (400 MHz, $CDCl_3$) δ 3.58 (m, 2H), 3.35 (m, 2H), 1.93 (m, 1H), 1.65 (s, 1H), 0.97 (d, *J* = 6.9, 3H); ^{13}C NMR (101 MHz, $CDCl_3$) δ 65.5, 54.8, 36.0, 14.6. HRMS (TOF, ESI) *m/z*: $[2M - H]^-$ calcd for $C_8H_{17}N_6O_2$ 229.1418, found 229.1414.

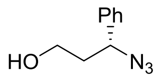

(*R*)-**9**

(*R*)-3-Azido-3-phenylpropanol, (*R*)-9[17]

(*R*)-3-Azido-3-phenylpropanol (*R*)-**9** was prepared following a previously published procedure, in which DPPA and DIAD were used instead of hydrazoic acid and DEAD, respectively. Characterization data were consistent with reported data.

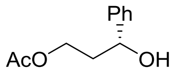

S3

(*R*)-3-Hydroxy-3-phenylpropyl acetate, S3

Following a literature procedure[17], (*R*)-3-hydroxy-3-phenylpropyl acetate **S3** was prepared as described. A mixture of (*R*)-3-chloro-1-phenylpropanol (1.03 g, 6.02 mmol), NaOAc (1.48 g, 18.0 mmol, 3.0 equiv) and NaI (1.08 mg, 7.20 mmol, 1.2 equiv) was heated at 130 ˚C in anhydrous DMF (60.0 mL) for 24 h. The reaction was cooled, and partitioned between Et_2O and water. The organic layer was washed with brine, dried over anhydrous Na_2SO_4, filtered, and concentrated under a stream of nitrogen. The crude oil was purified by an automated MPLC system using a 24 g normal phase silica column with gradient elution of 0–15% EtOAc/hexanes. Concentration of solvents under nitrogen afforded product as a colorless oil (758 mg, 3.90 mmol, 65% yield). R_f = 0.30 (20% EtOAc/hexanes); IR (neat) 3424, 1728 cm^{-1}; $[\alpha]_D^{20}$ = +22.3 (*c* 1.03, $CHCl_3$); ^1H NMR (400 MHz, $CDCl_3$) δ 7.36 (m, 4H), 7.29 (m, 1H), 4.80 (m, 1H), 4.33 (m, 1H), 4.13 (m, 1H), 2.20 (m, 1H), 2.14–1.95 (m, 5H); ^{13}C NMR (101 MHz, $CDCl_3$) δ 171.4, 144.0, 128.7, 127.9, 125.9, 71.4, 61.7, 38.1, 21.1. HRMS (FT-ICR, ESI) *m/z*: $[M + Na]^+$ calcd for $C_{11}H_{14}NaO_3$ 217.0835, found 217.0832.

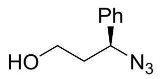

(*S*)-**9**

(*S*)-3-Azido-3-phenylpropanol, (*S*)-9

Following a literature procedure[17], (*S*)-3-azido-3-phenylpropan-1-ol (*S*)-**9** was prepared as described. A solution of (*R*)-3-hydroxy-3-phenylpropyl acetate **S3** (1.07 g, 5.50 mmol) dissolved in anhydrous THF (55.0 mL) was cooled to 0 ˚C. PPh_3 (2.17 g, 8.26 mmol, 1.5 equiv), DPPA (2.00 mL, 9.35 mmol, 1.7 equiv), and DIAD (1.6 mL, 8.25 mmol, 1.5 equiv) were added sequentially. The reaction mixture was warmed to room temperature and stirred for 18 h. The reaction mixture was partitioned between Et_2O and H_2O. The organic layer was washed with H_2O, brine, dried over anhydrous Na_2SO_4, filtered, and concentrated under a stream of nitrogen. The crude oil was purified by an automated MPLC system

using a 24 g normal phase silica column with gradient elution of 0–20% EtOAc/hexanes. Concentration of solvents under nitrogen afforded a colorless impure oil (R_f = 0.73, 20% EtOAc/hexanes). The impure oil was directly hydrolyzed with K_2CO_3 (1.14 g, 8.25 mmol, 1.5 equiv) in anhydrous MeOH (45.0 mL) at room temperature for 24 h. MeOH was removed under a stream of nitrogen, and the crude residue was purified by an automated MPLC system using a 24 g normal phase silica column with gradient elution of 0–20% EtOAc/hexanes. Concentration of solvents under nitrogen afforded product as a colorless oil (815 mg, 4.56 mmol, 83% yield) in 97.0% ee as determined by analytical HPLC. R_f = 0.30 (20% EtOAc/hexanes); IR (neat) 3335, 2094 cm^{-1}; $[\alpha]_D^{23}$ = –168.4 (*c* 1.02, $CHCl_3$); ^{1}H NMR (400 MHz, $CDCl_3$) δ 7.43–7.26 (m, 5H), 4.71 (dd, *J* = 8.8, 5.8 Hz, 1H), 3.82–3.67 (m, 2H), 2.11–1.92 (m, 2H); ^{13}C NMR (101 MHz, $CDCl_3$) δ 139.4, 129.1, 128.6, 127.0, 63.6, 59.8, 38.9. HRMS (TOF, ESI) *m/z*: [M – N_2 + H]$^+$ calcd for $C_9H_{12}NO$ 150.0913, found 150.0900. HPLC: Chiralcel OD-H, Daicel Chemical Industries, Ltd.; 2–40% *i*-PrOH/hexanes over 60 min; flow rate 1.0 mL/min; UV 220 nm; (*R*) t_R = 11.29 min, (*S*) t_R = 12.77 min.

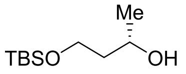

S4

(*S*)-4-((*tert*-Butyldimethylsilyl)oxy)butan-2-ol, S4

Following a literature procedure[22], (*S*)-4-((*tert*-butyldimethylsilyl)oxy)butan-2-ol **S4** was prepared as described. Imidazole (1.36 g, 20.0 mmol, 2.0 equiv) and DMAP (31.0 mg, 25.0 mmol, 2.5 equiv) were added to (*S*)-1,3-butandiol (900 µL, 10.0 mmol) in anhydrous DMF (25.0 mL, 0.4 M). The mixture was cooled to 0 ˚C and TBDMSCl (1.66 g, 11.0 mmol, 1.1 equiv) in anhydrous CH_2Cl_2 (12.0 mL) was added. The solution was stirred at 0 ˚C for 2 h and then stirred at room temperature overnight. The reaction mixture was diluted with H_2O and extracted with Et_2O (4 x 20 mL). The combined organic layers were dried over anhydrous Na_2SO_4, filtered, and concentrated under a stream of nitrogen. The crude oil was purified by an automated MPLC system using a 24 g normal phase silica column with gradient elution of 0–10% EtOAc/hexanes. Concentration of solvents under nitrogen afforded product as a colorless oil (1.82 g, 8.89 mmol, 89% yield). R_f = 0.32 (10% EtOAc/hexanes); IR (neat) 3388 cm^{-1}; $[\alpha]_D^{22}$ = –4.31 (*c* 1.02, $CHCl_3$); ^{1}H NMR (400 MHz, $CDCl_3$) δ 4.03 (m, 1H), 3.89 (m, 1H), 3.81 (m, 1H), 1.65 (m, 2H), 1.19 (d, *J* = 6.24 Hz, 3H), 0.90 (s, 9H), 0.081 (s, 6H); ^{13}C NMR (101 MHz, $CDCl_3$) δ 68.5, 62.9, 40.1, 26.0, 23.5, 18.3, -5.38, -5.43. HRMS (FT-ICR, ESI) *m/z*: [M + Na]$^+$ calcd for $C_{10}H_{24}NaO_2Si$ 227.1438, found 227.1434.

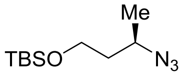

S5

(*R*)-(3-Azidobutoxy)(*tert*-butyl)dimethylsilane, S5

Following a literature procedure[17], (*R*)-(3-azidobutoxy)(*tert*-butyl)dimethylsilane **S5** was prepared as described. A solution of (*S*)-4-((*tert*-butyldimethylsilyl)oxy)butan-2-ol **S4** (1.02 g, 5.00 mmol) in anhydrous THF (50.0 mL) was cooled to 0 ˚C. PPh_3 (1.57 g, 6.00 mmol, 1.2 equiv), DPPA (1.60 mL, 7.42 mmol, 1.48 equiv), and DIAD (1.20 mL, 6.09 mmol, 1.2 equiv) were added sequentially. The reaction mixture was warmed to room temperature and stirred for 18 h. The reaction mixture was partitioned between Et_2O and H_2O. The organic layer was washed with H_2O, brine, dried over anhydrous Na_2SO_4, filtered, and concentrated under nitrogen atmosphere. The crude oil was purified by an automated MPLC system using a 24 g normal phase silica column with gradient elution of 100% hexanes. Concentration of solvents under nitrogen afforded product as a colorless oil (857 mg, 3.74 mmol, 75% yield). R_f = 0.75 (10% EtOAc/hexanes); IR (neat) 2098 cm^{-1}; $[\alpha]_D^{23}$ = –55.8 (*c* 1.00, $CHCl_3$); ^{1}H NMR (400 MHz, $CDCl_3$) δ 3.68 (m, 3H), 1.66 (m, 2H), 1.28 (d, *J* = 6.60 Hz, 3H), 0.90 (s, 9H), 0.062 (s, 3H), 0.057 (s, 3H); ^{13}C NMR (101 MHz, $CDCl_3$) δ 59.7, 54.9, 39.2, 26.1, 19.8, 18.4, -5.26. HRMS (TOF, ESI) *m/z*: [M – N_2 + H]$^+$ calcd for $C_{10}H_{24}NOSi$ 202.1622, found 202.1636.

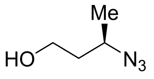

(R)-**10**

(*R*)-3-Azidobutanol, (*R*)-10

Following a literature procedure[17], (R)-3-azidobutanol (R)-**10** was prepared as described. A solution of (R)-(3-azidobutoxy)(*tert*-butyl)dimethylsilane **S5** (573 mg, 2.50 mmol) in anhydrous THF (50.0 mL) was treated with TBAF (1.0 M in THF, 2.75 mL, 2.75 mmol, 1.1 equiv). After stirring at room temperature for 30 min, the reaction was partitioned between Et_2O and H_2O. The organic layer was washed with a saturated solution of NH_4Cl, brine, dried over anhydrous Na_2SO_4, filtered, and concentrated under a stream of nitrogen. The crude oil was purified by an automated MPLC system using a 12 g normal phase silica column with gradient elution of 0–45% EtOAc/hexanes. Concentration of solvents under nitrogen afforded product as a colorless oil (234 mg, 2.03 mmol, 81% yield). R_f = 0.33 (25% EtOAc/hexanes); IR (neat) 3341, 2094 cm^{-1}; $[\alpha]_D^{23}$ = –107.3 (c 1.00, $CHCl_3$); ^{1}H NMR (400 MHz, $CDCl_3$) δ 3.81–3.68 (m, 3H), 1.72 (m, 2H), 1.32 (d, J = 6.56, 3H); ^{13}C NMR (101 MHz, $CDCl_3$) δ 60.0, 55.5, 38.7, 19.7. HRMS (TOF, ESI) *m/z*: $[2M - H]^-$ calcd for $C_8H_{17}N_6O_2$ 229.1418, found 229.1446.

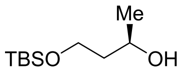

S6

(*R*)-4-((*tert*-Butyldimethylsilyl)oxy)butan-2-ol, S6[22]

(R)-4-((*tert*-Butyldimethylsilyl)oxy)butan-2-ol **S6** was prepared following a previously published procedure. Characterization data were consistent with reported data.

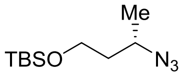

S7

(*S*)-(3-Azidobutoxy)(*tert*-butyl)dimethylsilane, S7

Following a literature procedure[17], (S)-(3-azidobutoxy)(*tert*-butyl)dimethylsilane **S7** was prepared as described. A solution of (R)-4-((*tert*-butyldimethylsilyl)oxy)butan-2-ol **S6** (1.02 g, 5.00 mmol) in anhydrous THF (50.0 mL) was cooled to 0 ˚C. PPh_3 (1.57 g, 6.00 mmol, 1.2 equiv), DPPA (1.60 mL, 7.42 mmol, 1.48 equiv), and DIAD (1.20 mL, 6.09 mmol, 1.21 equiv) were added sequentially. The reaction mixture was warmed to room temperature and stirred for 18 h. The reaction mixture was partitioned between Et_2O and H_2O. The organic layer was washed with H_2O, brine, dried over anhydrous Na_2SO_4, filtered, and concentrated under a stream of nitrogen. The crude oil was purified by an automated MPLC system using a 24 g normal phase silica column with gradient elution of 100% hexanes. Concentration of solvents under nitrogen afforded product as a colorless oil (897 mg, 3.91 mmol, 78% yield). R_f = 0.75 (10% EtOAc/hexanes); IR (neat) 2098 cm^{-1}; $[\alpha]_D^{23}$ = +58.1 (c 1.02, $CHCl_3$); ^{1}H NMR (400 MHz, $CDCl_3$) δ 3.69 (m, 3H), 1.66 (m, 2H), 1.28 (d, J = 6.56, 3H), 0.90 (s, 9H), 0.062 (s, 3H), 0.057 (s, 3H); ^{13}C NMR (101 MHz, $CDCl_3$) δ 59.7, 55.0, 39.3, 26.0, 19.8, 18.4, -5.27. HRMS (TOF, ESI) *m/z*: $[M – N_2 + H]^+$ calcd for $C_{10}H_{24}NOSi$ 202.1622, found 202.1597.

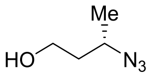

(S)-**10**

(*S*)-3-Azidobutanol, (*S*)-10

Following a literature procedure[17], (S)-3-azidobutanol (S)-**10** was prepared as described. A solution of (S)-(3-azidobutoxy)(*tert*-butyl)dimethylsilane **S7** (520 mg, 2.27 mmol) in anhydrous THF (45.0 mL)

was treated with TBAF (1.0 M in THF, 2.50 mL, 2.50 mmol, 1.1 equiv). After stirring at room temperature for 30 min, the reaction was partitioned between Et_2O and H_2O. The organic layer was washed with a saturated solution of NH_4Cl, brine, dried over anhydrous Na_2SO_4, filtered, and concentrated under a stream of nitrogen. The crude oil was purified by an automated MPLC system using a 12 g normal phase silica column with gradient elution of 0–45% EtOAc/hexanes. Concentration of solvents under nitrogen afforded product as a colorless oil (178 mg, 1.55 mmol, 68% yield). R_f = 0.32 (25% EtOAc/hexanes); IR (neat) 3341, 2094 cm^{-1}; $[\alpha]_D^{23}$ = +91.7 (*c* 1.00, $CHCl_3$); ^1H NMR (400 MHz, $CDCl_3$) δ 3.81–3.74 (m, 3H), 1.71 (m, 2H), 1.32 (d, *J* = 6.56 Hz, 3H); ^{13}C NMR (101 MHz, $CDCl_3$) δ 60.0, 55.5, 38.8, 19.7. HRMS (ESI) *m/z* calcd for $C_4H_{10}N_3O$ [M + H]$^+$ 116.0824, found 116.0485.

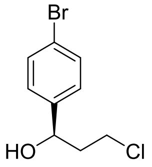

S8

(*R*)-1-(4-Bromophenyl)-3-chloropropanol, S8

Following a literature procedure[23], (*R*)-1-(4-bromophenyl)-3-chloropropanol **S8** was prepared as described. A flame-dried, three-neck 25 mL round bottom equipped with a thermocouple was charged with (*R*)-(+)-2-methyl-CBS-oxazaborolidine solution (1.0 M in toluene, 900 µL, 0.900 mmol, 0.15 equiv). Borane *N,N*-diethylaniline complex (1.60 mL, 9.00 mmol, 1.5 equiv), and was heated to 30 ˚C. 4′-Bromo-3-chloropropiophenone (1.48 g, 6.00 mmol) in anhydrous toluene (4.0 mL) was added dropwise to the reaction mixture using a syringe pump (0.07 mL/min) over 60 min. After ketone addition, the reaction mixture was stirred for an additional 1 h at 30 ˚C. The reaction mixture was allowed to 25 ˚C, and carefully quenched with MeOH (3.0 mL), followed by addition of 1.0 N HCl (5.0 mL) and stirred for 15–20 min. The aqueous layer was extracted with Et_2O (4 x 10 mL). The combined organic layers were washed with 1.0 N HCl (2 x 10 mL), H_2O, brine, dried over anhydrous Na_2SO_4, filtered, and concentrated. The crude oil was purified by an automated MPLC system using a 24 g normal phase silica column with gradient elution of 0–10% EtOAc/hexanes. Concentration of solvents under nitrogen afforded product as a colorless oil (1.25 g, 5.02 mmol, 84% yield) in 92.2% ee as determined by analytical HPLC. R_f = 0.30 (10% EtOAc/hexanes); IR (neat) 3366 cm^{-1}; $[\alpha]_D^{23}$ = +7.25 (*c* 1.00, $CHCl_3$); ^1H NMR (400 MHz, $CDCl_3$) δ 7.50 (m, 2H), 7.25 (m, 2H), 4.94 (m, 1H), 3.74 (m, 1H), 3.55 (m, 1H), 2.20 (m, 1H), 2.05 (m, 1H), 1.95 (br s, 1H); ^{13}C NMR (101 MHz, $CDCl_3$) δ 142.8, 131.9, 127.6, 121.8, 70.8, 41.6, 41.5. HRMS (TOF, ESI) *m/z*: [M + HCOO]$^-$ calcd for $C_{10}H_{11}BrClO_3$ 292.9580, found 292.9571. HPLC: Chiralcel OJ-H, Daicel Chemical Industries, Ltd.; 2–40% *i*-PrOH/hexanes over 60 min; flow rate 1.0 mL/min; UV 220 nm; (*S*) t_R = 11.57 min, (*R*) t_R = 12.89 min.

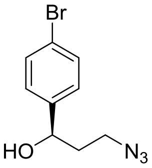

(*R*)-**11**

(*R*)-3-Azido-1-(4-bromophenyl)propanol, (*R*)-11

Following a literature procedure[17], (*R*)-3-azido-1-(4-bromophenyl)propanol (*R*)-**11** was prepared as described. A mixture of (*R*)-1-(4-bromophenyl)-3-chloropropanol **S8** (999 mg, 4.00 mmol), NaN_3 (780 mg, 12.0 mmol, 3.0 equiv), NaI (899 mg, 6.00 mmol, 1.5 equiv) in anhydrous DMF (40.0 mL) was stirred for 24 h at 80 ˚C. The reaction mixture was partitioned between Et_2O and H_2O. The organic layer was washed brine, dried over anhydrous Na_2SO_4, filtered, and concentrated under a stream of nitrogen. The crude oil was purified by an automated MPLC system using a 24 g normal phase silica column with gradient elution of 0–20% EtOAc/hexanes. Concentration of solvents under nitrogen afforded

product as a colorless oil (902 mg, 3.52 mmol, 88% yield) in 92.7% ee as determined by analytical HPLC. R_f = 0.20 (10% EtOAc/hexanes); IR (neat) 3386, 2092 cm^{-1}; $[\alpha]_D^{20}$ = +18.3 (*c* 1.00, $CHCl_3$); ^1H NMR (400 MHz, $CDCl_3$) δ 7.49 (m, 2H), 7.24 (m, 2H), 4.82 (dd, *J* = 8.5, 4.5, 1H), 3.52 (m, 1H), 3.38 (m, 1H), 2.09 (br s, 1H), 2.06–1.84 (m, 2H); ^{13}C NMR (101 MHz, $CDCl_3$) δ 143.0, 131.9, 127.6, 121.8, 76.8, 48.4, 38.0. HRMS (FT-ICR, APPI) *m/z*: $[M - N_2 + H]^+$ calcd for $C_9H_{11}BrNO$ 228.0019, found 228.0019. HPLC: Chiralpak IA, Daicel Chemical Industries, Ltd.; 8% EtOH/hexanes over 60 min; flow rate 1.0 mL/min; UV 220 nm; (*S*) t_R = 11.56 min, (*R*) t_R = 13.74 min.

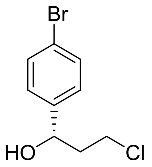

S9

(*S*)-1-(4-Bromophenyl)-3-chloropropanol, S9

Following a literature procedure[23], (*S*)-1-(4-bromophenyl)-3-chloropropanol **S9** was prepared as described. A flame-dried, three-neck 25 mL round bottom equipped with a thermocouple was charged with (*R*)-(+)-2-methyl-CBS-oxazaborolidine solution (1.0 M in toluene, 900 µL, 0.900 mmol, 0.15 equiv). Borane *N,N*-diethylaniline complex (1.60 mL, 9.00 mmol, 1.5 equiv), and was heated to 30 ˚C. 4′-Bromo-3-chloropropiophenone (1.48 g, 6.00 mmol) in anhydrous toluene (4.0 mL) was added dropwise to the reaction mixture using a syringe pump (0.07 mL/min) over 60 min. After ketone addition, the reaction mixture was stirred for an additional 1 h at 30 ˚C. The reaction mixture was allowed to 25 ˚C, and carefully quenched with MeOH (3.0 mL), followed by addition of 1.0 N HCl (5.0 mL) and stirred for 15–20 min. The aqueous layer was extracted with Et_2O (4 x 10 mL). The combined organic layers were washed with 1.0 N HCl (2 x 10 mL), H_2O, brine, dried over anhydrous Na_2SO_4, filtered, and concentrated. The crude oil was purified by an automated MPLC system using a 24 g normal phase silica column with gradient elution of 0–10% EtOAc/hexanes. Concentration of solvents under nitrogen afforded product as a colorless oil (1.20 g, 4.80 mmol, 80% yield) in 93.8% ee as determined by analytical HPLC. R_f = 0.30 (10% EtOAc/hexanes); IR (neat) 3366 cm^{-1}; $[\alpha]_D^{23}$ = –6.00 (*c* 1.00, $CHCl_3$). Characterization data were consistent with reported data.[24] HPLC: Chiralcel OJ-H, Daicel Chemical Industries, Ltd.; 2–40% *i*-PrOH/hexanes over 60 min; flow rate 1.0 mL/min; UV 220 nm; (*S*) t_R = 11.57 min, (*R*) t_R = 12.89 min.

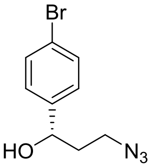

(*S*)-**11**

(*S*)-3-Azido-1-(4-bromophenyl)propanol, (*S*)-11

Following a literature procedure[17], (*S*)-3-azido-1-(4-bromophenyl)propanol (*S*)-**11** was prepared as described. A mixture of (*S*)-1-(4-bromophenyl)-3-chloropropanol **S9** (1.04 g, 4.16 mmol), NaN_3 (780 mg, 12.5 mmol, 3.0 equiv), NaI (890 mg, 6.24 mmol, 1.5 equiv) in anhydrous DMF (40.0 mL) was stirred for 24 h at 80 ˚C. The reaction mixture was partitioned between Et_2O and H_2O. The organic layer was washed with brine, dried over anhydrous Na_2SO_4, filtered, and concentrated under a stream of nitrogen. The crude oil was purified by an automated MPLC system using a 24 g normal phase silica column with gradient elution of 0–20% EtOAc/hexanes. Concentration of solvents under nitrogen afforded product as a colorless oil (1.01 g, 3.97 mmol, 95% yield) in 93.2% ee as determined by analytical HPLC. R_f = 0.20 (10% EtOAc/hexanes); IR (neat) 3385, 2092 cm^{-1}; $[\alpha]_D^{20}$ = –18.1 (*c* 1.00, $CHCl_3$); ^1H NMR (400 MHz, $CDCl_3$) δ 7.49 (m, 2H), 7.24 (m, 2H), 4.81 (dd, *J* = 8.4, 4.5 Hz, 1H), 3.51 (m, 1H), 3.38 (m, 1H), 2.14 (br s, 1H), 2.05–1.83 (m, 2H); ^{13}C NMR (101 MHz, $CDCl_3$) δ 142.9, 131.9,

127.6, 121.8, 71.4, 48.4, 38.0. HRMS (FT-ICR, APPI) *m/z*: [M – N_2 + H]$^+$ calcd for $C_9H_{11}BrNO$ 228.0019, found 228.0019. HPLC: Chiralpak IA, Daicel Chemical Industries, Ltd.; 8% EtOH/hexanes over 60 min; flow rate 1.0 mL/min; UV 220 nm; (*S*) t_R = 11.56 min, (*R*) t_R = 13.74 min.

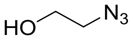

12

2-Azidoethanol, 12[16]
2-Azidoethanol **12** was prepared following a previously published procedure. Characterization data were consistent with reported data.

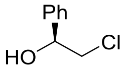

S10

(*S*)-2-Chloro-1-phenylethanol, S10
Following a literature procedure[23], (*S*)-2-chloro-1-phenylethanol **S10** was prepared as described. A flame-dried, three-neck 25 mL round bottom equipped with a thermocouple was charged with (*S*)-(+)-2-methyl-CBS-oxazaborolidine solution (1.0 M in toluene, 1.65 mL, 1.65 mmol, 0.1 equiv). Borane *N*,*N*-diethylaniline complex (3.0 mL, 1.01 mmol, 1.01 equiv), and was heated to 32 ˚C. 2-Chloroacetophenone (2.55 g, 16.5 mmol) in anhydrous toluene (6.0 mL) was added dropwise to the reaction mixture using a syringe pump (0.10 mL/min) over 90 min. After ketone addition, the reaction mixture was stirred for an additional 1 h at 32 ˚C. The reaction mixture was allowed to 25 ˚C, and carefully quenched with MeOH (5.0 mL), followed by addition of 1.0 N HCl (10.0 mL) and stirred for 15–20 min. The aqueous layer was extracted with Et_2O (4 x 15 mL). The combined organic layers were washed with 1.0 N HCl (2 x 10 mL), H_2O, brine, dried over anhydrous Na_2SO_4, filtered, and concentrated. The crude oil was purified by an automated MPLC system using a 24 g normal phase silica column with gradient elution of 0–5% EtOAc/hexanes. Concentration of solvents under nitrogen afforded product as a colorless oil (2.09 g, 13.3 mmol, 81% yield) in 97.8% ee as determined by analytical HPLC. R_f = 0.40 (10% EtOAc/hexanes); IR (neat) 3379 cm^{-1}; $[\alpha]_D^{22}$ = +57.3 (*c* 1.00, $CHCl_3$). Characterization data were consistent with reported data.[25] HPLC: Chiralcel OD-H, Daicel Chemical Industries, Ltd.; 2–40% *i*-PrOH/hexanes over 60 min; flow rate 1.0 mL/min; UV 220 nm; (*S*) t_R = 9.75 min, (*R*) t_R = 10.83 min.

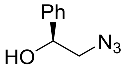

(*S*)-**13**

(*S*)-2-Azido-1-phenylethanol, (*S*)-13
Following a literature procedure[17], (*S*)-2-azido-1-phenylethanol (*S*)-**13** was prepared as described. A mixture of (*S*)-2-chloro-1-phenylethanol **S10** (313 mg, 2.00 mmol), NaN_3 (520 mg, 8.00 mmol, 4.0 equiv), and NaI (599 g, 4.00 mmol, 2.0 equiv) in anhydrous DMF (20.0 mL) was stirred for 30 h at 85 ˚C. The reaction mixture was partitioned between Et_2O and H_2O. The organic layer was washed with brine, dried over anhydrous Na_2SO_4, filtered, and concentrated under a stream of nitrogen. The crude oil was purified by an automated MPLC system using a 24 g normal phase silica column with gradient elution of 0–10% EtOAc/hexanes. Concentration of solvents under nitrogen afforded product as a colorless oil (292 mg, 1.79 mmol, 90% yield) in ≥99.5% ee as determined by analytical HPLC. R_f = 0.25 (10% EtOAc/hexanes); IR (neat) 3396, 2096 cm^{-1}; $[\alpha]_D^{22}$ = +87.9 (*c* 1.03, $CHCl_3$); ^{1}H NMR (400 MHz, $CDCl_3$) δ 7.45–7.29 (m, 5H), 4.89 (m, 1H), 3.47 (m, 2H), 2.32 (d, *J* = 3.2 Hz, 1H)**;** ^{13}C NMR (101 MHz, $CDCl_3$) δ 140.7, 128.9, 128.6, 126.1, 73.6, 58.3. HRMS (TOF, ESI) *m/z*: [M – N_2 + H]$^+$ calcd for $C_8H_{10}NO$ 136.0757, found 136.0719. HPLC: Chiralcel OJ-H, Daicel Chemical Industries, Ltd.; 2% *i*-PrOH/hexanes over 60 min; flow rate 1.0 mL/min; UV 220 nm; (*R*) t_R = 24.41 min, (*S*) t_R = 29.05 min.

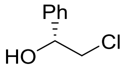

S11

(*R*)-2-Chloro-1-phenylethanol, S11

Following a literature procedure[23], (*R*)-2-chloro-1-phenylethanol **S11** was prepared as described. A flame-dried, three-neck 25 mL round bottom equipped with a thermocouple was charged with (*R*)-(+)-2-methyl-CBS-oxazaborolidine solution (1.0 M in toluene, 1.65 mL, 1.65 mmol, 0.1 equiv). Borane *N*,*N*-diethylaniline complex (3.0 mL, 1.01 mmol, 1.01 equiv), and was heated to 32 ˚C. 2-Chloroacetophenone (2.55 g, 16.5 mmol) in anhydrous toluene (6.0 mL) was added dropwise to the reaction mixture using a syringe pump (0.10 mL/min) over 90 min. After ketone addition, the reaction mixture was stirred for an additional 1 h at 32 ˚C. The reaction mixture was allowed to 25 ˚C, and carefully quenched with MeOH (5.0 mL), followed by addition of 1.0 N HCl (10.0 mL) and stirred for 15–20 min. The aqueous layer was extracted with Et_2O (4 x 15 mL). The combined organic layers were washed with 1.0 N HCl (2 x 10 mL), H_2O, brine, dried over anhydrous Na_2SO_4, filtered, and concentrated. The crude oil was purified by an automated MPLC system using a 24 g normal phase silica column with gradient elution of 0–5% EtOAc/hexanes. Concentration of solvents under nitrogen afforded product as a colorless oil (2.12 g, 13.5 mmol, 82% yield) in 96.0% ee as determined by analytical HPLC. R_f = 0.40 (10% EtOAc/hexanes); IR (neat) 3383 cm^{-1}; $[\alpha]_D^{22}$ = –53.3 (*c* 1.00, $CHCl_3$). Characterization data were consistent with reported data.[24] HPLC: Chiralcel OD-H, Daicel Chemical Industries, Ltd.; 2–40% *i*-PrOH/hexanes over 60 min; flow rate 1.0 mL/min; UV 220 nm; (*S*) t_R = 9.75 min, (*R*) t_R = 10.83 min.

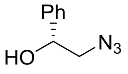

(*R*)-**13**

(*R*)-2-Azido-1-phenylethanol, (*R*)-13

Following a literature procedure[17], (*R*)-2-azido-1-phenylethanol (*R*)-**13** was prepared as described. A mixture of (*R*)-2-chloro-1-phenylethanol **S11** (472 mg, 3.01 mmol), NaN_3 (683 mg, 10.5 mmol, 3.5 equiv), and NaI (899 mg, 6.00 mmol, 2.0 equiv) in anhydrous DMF (30.0 mL) was stirred for 24 h at 85 ˚C. The reaction mixture was partitioned between Et_2O and H_2O. The organic layer was washed with brine, dried over anhydrous Na_2SO_4, filtered, and concentrated under a stream of nitrogen. The crude oil was purified by an automated MPLC system using a 24 g normal phase silica column with gradient elution of 0–10% EtOAc/hexanes. Concentration of solvents under nitrogen afforded product as a colorless oil (406 mg, 2.49 mmol, 83% yield) in ≥99.5% ee as determined by analytical HPLC. R_f = 0.25 (10% EtOAc/hexanes); IR (neat) 3392, 2097 cm^{-1}; $[\alpha]_D^{22}$ = –87.4 (*c* 1.02, $CHCl_3$); ^1H NMR (400 MHz, $CDCl_3$) δ 7.43–7.29 (m, 5H), 4.89 (m, 1H), 3.48 (m, 2H), 2.31 (br s, 1H); ^{13}C NMR (101 MHz, $CDCl_3$) δ 140.7, 128.9, 128.5, 126.1, 73.6, 58.3. HRMS (TOF, ESI) *m/z*: $[M - N_2 + H]^+$ calcd for $C_8H_{10}NO$ 136.0757, found 136.0747. HPLC: Chiralcel OJ-H, Daicel Chemical Industries, Ltd.; 2% *i*-PrOH/hexanes over 60 min; flow rate 1.0 mL/min; UV 220 nm; (*R*) t_R = 24.41 min, (*S*) t_R = 29.05 min.

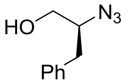

(*S*)-**14**

(*S*)-2-Azido-3-phenylpropanol, (*S*)-14[18]

(*S*)-2-Azido-3-phenylpropanol (*S*)-**14** was prepared following a previously published procedure. Characterization data were consistent with reported data.

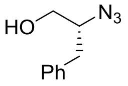

(*R*)-**14**

(*R*)-2-Azido-3-phenylpropanol, (*R*)-14[18]
(*R*)-2-Azido-3-phenylpropanol (*R*)-**14** was prepared following a previously published procedure. Characterization data were consistent with reported data.

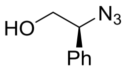

(*S*)-**15**

(*S*)-2-Azido-2-phenylethanol, (*S*)-15
Following the literature procedure[18,26], (*S*)-2-azido-2-phenylethanol (*S*)-**15** was obtained as a yellow oil (376 mg, 2.31 mmol, 58% yield) in 94.9% ee as determined by analytical HPLC. Purification was carried out by an automated MPLC system using a 24 g normal phase silica column with gradient elution of 0–10% EtOAc/hexanes. R_f = 0.22 (10% EtOAc/hexanes); IR (neat) 3356, 2094 cm^{-1}; $[\alpha]_D^{22}$ = +199.4 (*c* 0.98, $CHCl_3$); ^1H NMR (400 MHz, $CDCl_3$) δ 7.43–7.32 (m, 5H), 4.68 (dd, *J* = 7.1, 5.7 Hz, 1H), 3.80–3.72 (m, 2H); ^{13}C NMR (101 MHz, $CDCl_3$) δ 136.4, 129.1, 128.9, 127.3, 68.0, 66.7. HRMS (TOF, ESI) *m/z*: $[M - N_2 + H]^+$ calcd for $C_8H_{10}NO$ 136.0757, found 136.0749. HPLC: Chiralpak IA, Daicel Chemical Industries, Ltd.; 2–30% EtOH/hexanes over 60 min; flow rate 1.0 mL/min; UV 220 nm; (*S*) t_R = 13.38 min, (*R*) t_R = 15.38 min.

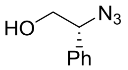

(*R*)-**15**

(*R*)-2-Azido-2-phenylethanol, (*R*)-15[19]
(*R*)-2-Azido-2-phenylethanol (*R*)-**15** was prepared following a previously published procedure. Characterization data were consistent with reported data.

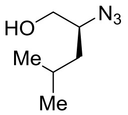

(*S*)-**16**

(*S*)-2-Azido-4-methylpentanol, (*S*)-16[18]
(*S*)-2-Azido-4-methylpentanol (*S*)-**16** was prepared following a previously published procedure. Characterization data were consistent with reported data.

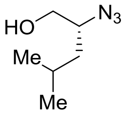

(*R*)-**16**

(*R*)-2-Azido-4-methylpentanol, (*R*)-16
Following the literature procedure[18,26], (*R*)-2-azido-4-methylpentanol (*R*)-**16** was obtained as a yellow oil (363 mg, 2.56 mmol, 63% yield). Purification was carried out by an automated MPLC system using a 24 g normal phase silica column with gradient elution of 0–15% EtOAc/hexanes. R_f = 0.42 (10% EtOAc/hexanes); IR (neat) 3356, 2101 cm^{-1}; $[\alpha]_D^{22}$ = +5.0 (*c* 1.00, $CHCl_3$); ^1H NMR (400 MHz, $CDCl_3$) δ 3.73–3.67 (m, 1H), 3.57–3.50 (m, 2H), 1.83–1.73 (m, 1H), 1.49–1.42 (m, 1H), 1.32–1.25 (m, 1H),

0.96 (dd, J = 6.6, 0.8 Hz, 6H); ^{13}C NMR (101 MHz, $CDCl_3$) δ 65.8, 62.7, 39.6, 25.1, 23.1, 22.2. HRMS (TOF, ESI) *m/z*: $[M - N_2 + H]^+$ calcd for $C_8H_{10}NO$ 116.1070, found 116.1035.

General experimental procedures for azasteroid library production

General procedure A for the preparation of A1–A9, B1–B9, C1–C7, D1–D7, E1–E2, and F1–F2: To a solution of steroidal ketone **1**–**3** (0.124–0.302 mmol, 1.0 equiv) and hydroxyalkyl azide **6**–**14** (0.242–0.593 mmol, 2.0 equiv) in anhydrous CH_2Cl_2 (0.4 M) in a nitrogen-flushed, two-dram vial at 0 ˚C was added BF_3•OEt_2 (5.0 equiv) dropwise. The vial was capped, and the reaction mixture was stirred at room temperature for 16 h. The solvent was removed under nitrogen, and the residual iminium was treated with an aqueous solution of 15% KOH (3.0 mL) and THF (0.5 mL). The biphasic mixture was stirred vigorously at room temperature for 24 h. The reaction mixture was diluted with CH_2Cl_2 (50 mL), dried over Na_2SO_4, filtered, and concentrated. Purification of analogs was carried out by an automated MPLC system on normal phase silica gel flash columns.

General procedure B for the preparation of C8–C15, D8–D15, E3–E5, and F3–F5:
*Step 1: Formation of iminium ether intermediate of **2** or **3**:* In either a flame-dried, nitrogen-flushed, two-dram vial or 5 mL-microwave vial was added BF_3•OEt_2 (62.0–124 µL, 0.500–1.00 mmol, 5.0 equiv) dropwise to a solution of 5α-dihydrotestosterone **2** (29.0–60.0 mg, 0.100–0.207 mmol, 1.0 equiv) or 5β-dihydrotestosterone **3** (43.9–58.9 mg, 0.151–0.203 mmol, 1.0 equiv) and hydroxyalkyl azides (*R*)-**7** (44.5–72.7 mg, 0.250–0.410 mmol, 2.0 equiv), (*S*)-**7** (36.8–73.5 mg, 0.299–0.415 mmol, 2.0 equiv), (*S*)-**9** (44.3–74.0 mg, 0.250–0.418 mmol, 2.0 equiv), or (*R*)-**9** (44.5–73.4 mg, 0.250–0.414 mmol, 2.0 equiv) in anhydrous CH_2Cl_2 (0.4 M) at 0 ˚C. The vial was capped, and the reaction mixture was stirred at room temperature for 16 h. The solvent was removed under nitrogen, and the residual iminium was dried under vacuum before the addition of nucleophile.

Step 2: Addition of nucleophiles:
*2.1. Synthesis of **C8**, **C13**, **D8**, **E3**, and **F3** via nucleophilic addition of sulfide:* Na_2S (65.2–79.8 mg, 0.835–1.02 mmol, 4.1–5.0 equiv) was added to a solution of iminium ether in anhydrous DMF (5.0 mL). The reaction mixture was stirred at 65 ˚C for 24 h. The reaction mixture was diluted with CH_2Cl_2 (20 mL), and was washed with a saturated solution of NH_4Cl (3 × 5 mL), brine (5 mL), dried over Na_2SO_4, filtered, and concentrated. Purification of analogs were carried out by an automated MPLC system on normal phase silica columns.

*2.2. Synthesis of **C9** and **D9** via nucleophilic addition of hydride:* To a solution of iminium residue in anhydrous MeOH (2.0 mL) at 0 ˚C was added $NaBH_4$ (30.0 mg, 0.799 mmol, 4.0 equiv) cautiously. The reaction mixture was stirred at room temperature for overnight. The reaction mixture was partitioned between saturated solution of $NaHCO_3$ (5 mL) and CH_2Cl_2 (20 mL). The organic layer was separated, dried over Na_2SO_4, filtered, and concentrated. Purification of analogs was carried out by an automated MPLC system on normal phase silica gel flash columns.

*2.3. Synthesis of **C10** and **D10** via nucleophilic addition of hydride:* 10% Pd/C (10.6–21.0 mg, 0.100-0.197 mmol, 1.0 equiv) was added to a solution of iminium ether in anhydrous EtOH (2.0–4.0 mL). The reaction was stirred under an atmosphere of hydrogen for 24 h. Purification of analogs was carried out by an automated MPLC system on normal phase silica gel flash columns.

*2.4. Synthesis of **C11**, **C14**, **D11**, **D14**, **E4**, and **F4** via nucleophilic addition of azide:* NaN_3 (51.0–52.0 mg, 0.785–0.802 mmol, 4.0 equiv) was added to a solution of iminium ether in anhydrous DMF (2.0 mL). The reaction mixture was stirred at 70 ˚C for 24 h. The reaction mixture was partitioned between EtOAc (40 mL) and H_2O (20 mL). The organic layer was washed with H_2O (2 × 5 mL), brine (5 mL), dried over Na_2SO_4, filtered, and concentrated. Purification of analogs were carried out by an automated MPLC system on normal phase silica gel columns.

*2.5. Synthesis of **C12**, **C15**, **D12**, **D15**, **E5**, and **F5** via nucleophilic addition of 4-methylbenzenethiolate:* Sodium 4-methylbenzenethiolate (0.66 M in DMF, 0.60–1.00 mL, 0.375–0.660 mmol, 3.0–4.0 equiv)

was added to a solution of iminium ether in anhydrous DMF (2.0 mL). The reaction mixture was stirred at 75 ˚C for 24 h. The reaction mixture was partitioned between EtOAc (40 mL) and H_2O (15 mL). The organic layer was washed with a saturated solution of $NaHCO_3$ (3 × 5 mL), brine (5 mL), dried over Na_2SO_4, filtered, and concentrated to afford crude residue. Purification of analogs were carried out by an automated MPLC system on normal phase silica gel columns.

General procedure C for the PCC oxidation of A11, B11, C16, and D16: To a slurry solution of **A3**, **B3**, **C2**, or **D2** (53.0–93.0 mg, 0.099–0.212 mmol) in anhydrous CH_2Cl_2 (6.0–10.0 mL) and Celite at 0 ˚C was added PCC (43.0–190.0 mg, 0.200–0.880 mmol, 2.0–4.0 equiv). The brown reaction mixture was allowed to room temperature over 30 min and stirred overnight. The reaction mixture was diluted with CH_2Cl_2, filtered over Celite and concentrated. Purification of analogs was carried out by an automated MPLC system on normal phase silica gel flash columns.

General procedure D for the optimization of G1: To a solution of *trans*-androsterone **4** (0.150 mmol, 1.0 equiv) and 3-azidopropanol **6** (0.300–0.450 mmol, 2.0–3.0 equiv) in solvent (0.38 mL, 0.4 M) in a nitrogen-flushed, two-dram vial at room temperature (unless otherwise noted) was added acid catalyst (0.0825–1.05 mmol, 0.55–7.0 equiv) dropwise. The vial was capped, and the reaction mixture was stirred at room temperature for 24 h. The solvent was removed under nitrogen, and an aqueous solution of 15% KOH (3.0 mL) was added to the iminium residue. The reaction mixture was vigorously stirred at room temperature for 24 h. The reaction mixture was diluted with CH_2Cl_2 (50 mL), dried over anhydrous Na_2SO_4, filtered, and concentrated. Purification was carried out by an automated MPLC system using a 4 g normal phase silica gel flash column with gradient elution from 0–5% $MeOH/CH_2Cl_2$.

General procedure E for the preparation of G1–G8 and H1–H6: To a solution of *trans*-androsterone **4** (0.125–0.153 mmol, 1.0 equiv) and azido alcohol **6**, **8**, **10**, **12**, or **14**–**16** (0.250–0.302 mmol, 2.0 equiv) in HFIP (0.38 mL, 0.4 M) at room temperature or in anhydrous CH_2Cl_2 (0.50 mL, 0.3 M) at 0 ˚C in a nitrogen-flushed, two-dram vial was added TfOH (14.5–26.5 µL, 0.165–0.300 mmol, 1.1–2.0 equiv) or BF_3•OEt_2 (130 µL, 1.05 mmol, 7.0 equiv) dropwise. The vial was capped, and the reaction mixture was stirred at room temperature for 24 h. The solvent was removed under nitrogen, and the residual iminium was treated with an aqueous solution of 15% KOH (3.0 mL) and THF (0.5 mL). The biphasic mixture was stirred vigorously at room temperature for 24 h. The reaction mixture was diluted with CH_2Cl_2 (50 mL), dried over Na_2SO_4, filtered, and concentrated. Purification of analogs was carried out by an automated MPLC system on normal phase silica gel flash columns.

General procedure F for the preparation of I1–I9 and J1–J6: To a solution of estrone **5** (0.149–0.200 mmol, 1.0 equiv) and hydroxyalkyl azide **6**, **8**, **10**, **12**, or **14**–**16** (0.294–0.400 mmol, 2.0 equiv) in HFIP (0.38 mL, 0.4 M) at room temperature or in anhydrous CH_2Cl_2 (0.50 mL, 0.3 M) at 0 ˚C in a nitrogen-flushed, two-dram vial was added TfOH (14.5–26.5 µL, 0.165–0.300 mmol, 1.1–2.0 equiv) or BF_3•OEt_2 (130 µL, 1.05 mmol, 7.0 equiv) dropwise. The vial was capped, and the reaction mixture was stirred at room temperature for 24 h. The solvent was removed under nitrogen, and the residual iminium was treated with an aqueous solution of 15% KOH (3.0 mL) and THF (0.5 mL). The biphasic mixture was stirred vigorously at room temperature for 24 h. The reaction mixture was diluted with a saturated solution of NH_4Cl, and extracted into CH_2Cl_2 (3 × 15 mL). The combined organic layers were washed with brine, dried over Na_2SO_4, filtered, and concentrated. Purification of analogs was carried out by an automated MPLC system on normal phase silica gel flash columns.

General procedure G for the preparation of G9–G17 and I10–I16:
Step 1: Formation of iminium ether intermediate of ***4*** *or* ***5****:* To a solution of *trans*-androsterone **4** (43.6–145.0 mg, 0.150–0.500 mmol, 1.0 equiv) or estrone **5** (40.6–81.0 mg, 0.150–0.300 mmol, 1.0 equiv) and 3-azidopropanol **6** (30.1–101 mg, 0.300–1.00 mmol, 2.0 equiv) in HFIP (0.4 M) in either a flame-dried, nitrogen-flushed two-dram vial, 20 mL–scintillation vial, or 5 mL-microwave vial was added TfOH (14.5–48.5 µL, 0.165–0.550 mmol, 1.1 equiv) dropwise. The vial was capped, and the reaction mixture was stirred at room temperature for 24 h. The solvent was removed under nitrogen, and the residual iminium was dried under vacuum before the addition of nucleophile.

Step 2: Addition of nucleophiles:

*2.1. Synthesis of **G9** and **I10** via nucleophilic addition of sulfide:* Na_2S (195–234 mg, 2.50–3.00 mmol, 10.0 equiv) was added to a solution of iminium ether in anhydrous THF (5.0 mL). The reaction mixture was stirred at 65 ˚C for 24 h. The reaction mixture was diluted with Et_2O (20 mL), and was washed with a saturated solution of NH_4Cl (3 × 5 mL), brine (5 mL), dried over Na_2SO_4, filtered, and concentrated. Purification of analogs were carried out by an automated MPLC system on normal phase silica columns.

*2.2. Synthesis of **G10**–**G11** and **I11**–**I12** via nucleophilic addition of hydride:* To a solution of iminium residue in anhydrous MeOH (2.0 mL) at 0 ˚C was added $NaBH_4$ (151 mg, 0.400 mmol, 2.0 equiv) or $NaBD_4$ (167 mg, 0.400 mmol, 2.0 equiv) cautiously. The reaction mixture was stirred at room temperature for 3 h. The reaction mixture was partitioned between saturated solution of $NaHCO_3$ (5 mL) and CH_2Cl_2 (20 mL). The organic layer was separated, dried over Na_2SO_4, filtered, and concentrated. Purification of analogs was carried out by an automated MPLC system on normal phase silica gel flash columns.

*2.3. Synthesis of **G12** and **I13** via nucleophilic addition of azide:* NaN_3 (39.0–130 mg, 0.600–2.00 mmol, 4.0 equiv) was added to a solution of iminium ether in anhydrous DMF (2.0–5.0 mL). The reaction mixture was stirred at 70 ˚C for 24 h. The reaction mixture was partitioned between Et_2O (40 mL) and H_2O (20 mL). The organic layer was washed with H_2O (2 × 5 mL), brine (5 mL), dried over Na_2SO_4, filtered, and concentrated. Purification of analogs was carried out by an automated MPLC system on normal phase silica gel flash columns.

*2.4. Synthesis of **G13**–**G17** and **I14**–**I16** via nucleophilic addition of para-substituted benzenethiolates:* Sodium thiobenzolate (0.56–0.70 M in DMF, 0.54–0.67 mL, 2.5 equiv) was added to a solution of iminium ether in anhydrous DMF (2.0 mL). The reaction mixture was stirred at 75 ˚C for 24 h. The reaction mixture was partitioned between EtOAc (40 mL) and H_2O (15 mL). The organic layer was washed with a saturated solution of $NaHCO_3$ (3 × 5 mL), brine (5 mL), dried over Na_2SO_4, filtered, and concentrated. Purification of analogs was carried out by an automated MPLC system on normal phase silica gel flash columns.

General procedure H for the preparation of G18–G20 and I17:

A mixture of steroidal azides **G12** or **I13** (0.180–0.200 mmol, 1.0 equiv), substituted-acetylene (0.207–0.400 mmol, 1.0–2.0 equiv), copper sulfate pentahydrate (0.200–0.400 mmol, 1.0–2.0 equiv), and sodium L-ascorbate (0.400–0.800 mmol, 2.0–4.0 equiv) were dissolved in *t*-BuOH/H_2O (1:1, 4.0 mL) at room temperature and stirred overnight. The reaction mixture was diluted with CH_2Cl_2 (25 mL), and the organic layer was washed with aqueous NH_4OH (3 x 5 mL), brine (5 mL), dried over Na_2SO_4, filtered, and concentrated. Purification of analogs was carried out by an automated MPLC system on normal phase silica gel flash columns.

Characterization and regiochemistry discussion for A-ring library members

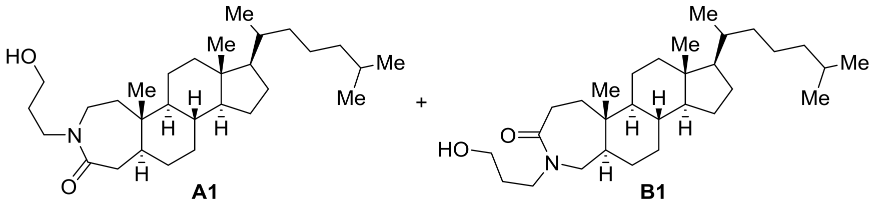

5α-Cholestane-derived A-Ring Lactams, A1 and B1

Following the general procedure A, 3-azidopropanol **6** (30.5 mg, 0.301 mmol, 2.0 equiv) was reacted with 5α-cholestan-3-one **1** (58.0 mg, 0.150 mmol) to give an inseparable mixture (38:62) of

regioisomers **A1** and **B1** as a white amorphous solid (63.4 mg, 0.138 mmol, 92% yield). Purification was carried out by an automated MPLC system using a 4 g normal phase silica column with gradient elution from 0–5% MeOH/CH_2Cl_2. R_f = 0.43 (5% MeOH/CH_2Cl_2); IR (neat) 3318, 1625 cm^{-1}; key ^1H NMR (600 MHz, $CDCl_3$) δ 3.67–3.61 (m, 2H), 2.96 (ddd, *J* = 15.7, 6.5, 1.8 Hz, 1H), 2.79 (dd, *J* = 14.3, 10.6 Hz, 1H), 2.65 (t, *J* = 13.1 Hz, 1H), 2.34 (dd, *J* = 14.5, 7.6 Hz, 1H), 2.00–1.93 (m, 2H), 1.91–1.77 (m, 3H); ^{13}C NMR (151 MHz, $CDCl_3$) δ 177.3, 177.2, 58.10, 58.06, 56.5, 56.3, 54.2, 54.0, 52.0, 48.9, 45.4, 44.8, 44.5, 44.0, 42.44, 42.40, 41.0, 40.04, 40.01, 39.98, 39.6, 38.5, 38.3, 36.3, 35.9, 35.1, 34.9, 32.12, 32.06, 31.9, 31.0, 30.32, 30.28, 28.5, 28.4, 28.1, 24.3, 24.0, 22.9, 22.7, 21.4, 21.1, 18.77, 18.75, 12.21, 12.15. **Note**: Missing carbon signals due to signal overlap of regioisomers. COSY and HSQC are included in the spectra section. HRMS (FT-ICR, ESI) *m/z*: $[M + H]^+$ calcd for $C_{30}H_{54}NO_2$ 460.4149, found 460.4149.

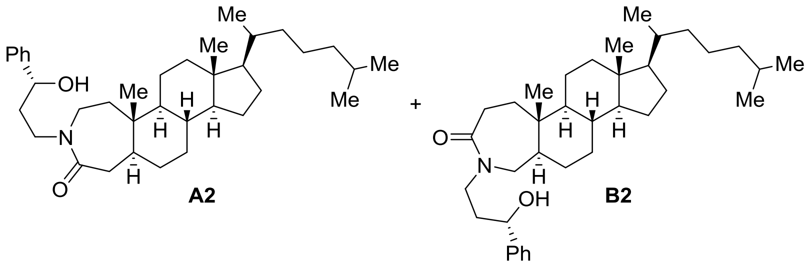

5α-Cholestane-derived A-Ring Lactams, A2 and B2

Following the general procedure A, (±)-3-azido-1-phenylpropanol **(±)-7** (44.3 mg, 0.250 mmol, 2.0 equiv) was reacted with 5α-cholestan-3-one **1** (48.3 mg, 0.125 mmol) to give a 50:50 mixture of regioisomers **A2** and **B2** as a white amorphous solid (61.4 mg, 0.115 mmol, 92% yield). Purification was carried out by an automated MPLC system using a 12 g normal phase column 0–5% MeOH/CH_2Cl_2. The mixture was intentionally not separated by column chromatography. R_f = 0.40 (5% MeOH/CH_2Cl_2); IR (neat) 3376, 1617 cm^{-1}; key ^1H NMR (400 MHz, $CDCl_3$) δ 4.67–4.62 (m, 2H), 4.15–4.03 (m, 2H), 3.73–3.63 (m, 2H), 3.15–3.07 (m, 2H), 3.01 (m, 1H), 2.71 (dd, *J* = 14.2, 10.4 Hz, 1H), 2.61–2.52 (m, 2H, contains d, 2.54, *J* = 15.6 Hz, 1H), 2.40–2.35 (m, 1H); ^{13}C NMR (101 MHz, $CDCl_3$) δ 177.21, 177.1, 144.3, 144.26, 128.47, 128.43, 127.2, 127.1, 125.7, 127.6, 70.0, 56.5, 56.4, 56.32, 56.30, 54.5, 54.3, 52.5, 49.6, 45.9, 45.7, 44.2, 42.5, 42.4, 41.2, 40.11, 40.05, 39.9, 39.65, 36.63, 38.5, 38.4, 38.1, 37.9, 36.3, 36.1, 35.9, 35.1, 34.9, 32.21, 32.17, 32.0, 31.0, 28.5, 28.3, 28.1, 24.29, 24.25, 23.9, 22.9, 22.7, 21.4, 21.1, 18.79, 18.76, 12.2, 12.1. **Note**: Missing carbon signals due to signal overlap of regioisomers. HRMS (FT-ICR, ESI) *m/z*: $[M + H]^+$ calcd for $C_{36}H_{58}NO_2$ 536.4462, found 536.4467.

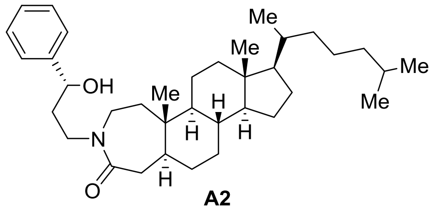

5α-Cholestane-derived A-Ring Lactam, A2

Following the general procedure A, (*R*)-3-azido-1-phenylpropanol (*R*)-**7** (44.5 mg, 0.251 mmol, 2.0 equiv) was reacted with 5α-cholestan-3-one **1** (48.4 mg, 0.125 mmol) to give **A2** as a white amorphous solid (59.5 mg, 0.111 mmol, 89% yield). Purification was carried out by an automated MPLC system using a 12 g normal phase silica column 0–40% EtOAc/hexanes. R_f = 0.54 (50% EtOAc/hexanes); mp 178–182 ˚C; IR (neat) 3373, 1613 cm^{-1}; ^1H NMR (400 MHz, $CDCl_3$) δ 7.39–7.33 (m, 4H), 7.31–7.25 (m, 1H), 4.65 (dd, *J* = 9.8, 3.2 Hz, 1H), 4.07 (ddd, *J* = 14.6, 10.6, 4.2 Hz, 1H), 3.66 (dd, *J* = 15.7, 11.8 Hz, 1H), 3.12 (dt, *J* = 14.2, 4.6 Hz, 1H), 3.01 (dd, *J* = 15.6, 6.2 Hz, 1H), 2.71 (dd, *J* = 14.1, 10.4 Hz, 1H), 2.00–1.77 (complex, 4H, contains d, *J* = 14.2 Hz, 1H), 1.69–1.65 (m, 1H), 1.59–0.93 (complex, 24H), 0.94–0.85 (complex, 12H, contains s, 0.91, 3H; s, 0.89, 3H; d, 0.87, *J* = 1.8 Hz, 3H; d, 0.85, *J* =

1.9 Hz, 3H), 0.75–0.65 (m, 4H, contains s, 3H); ^{13}C NMR (101 MHz, $CDCl_3$) δ 177.2, 144.3, 128.4 (2C), 127.2, 125.7 (2C), 70.1, 56.5, 56.3, 54.5, 45.9 45.7, 44.2, 42.4, 41.8, 40.1, 40.07, 39.6, 38.5, 37.9, 36.3, 35.9, 34.9, 32.0, 31.0, 28.3, 28.1, 24.3, 24.0, 22.9, 22.7, 21.4, 18.9, 12.2, 12.1. **Note**: APT and HSQC are included in the spectra section. HRMS (FT-ICR, ESI) *m/z*: [M + H]$^+$ calcd for $C_{36}H_{58}NO_2$ 536.4462, found 536.4463.

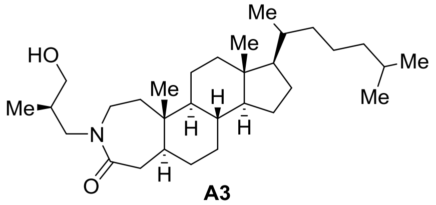

A3

5α-Cholestane-derived A-Ring Lactam, A3

Following the general procedure A, (*R*)-3-azido-2-methylpropanol (*R*)-**8** (34.4 mg, 0.300 mmol, 2.0 equiv) was reacted with 5α-cholestan-3-one **1** (58.1 mg, 0.150 mmol) to give **A3** as a white amorphous solid (50.2 mg, 0.106 mmol, 71%). Purification was carried out by an automated MPLC system using a 12 g normal phase silica with gradient elution from 0–5% MeOH/CH_2Cl_2. R_f = 0.27 (2% MeOH/CH_2Cl_2 run twice); mp 160–162 ˚C; IR (neat) 3347, 1616 cm^{-1}; ^{1}H NMR (400 MHz, $CDCl_3$) δ 3.67 (dd, *J* = 13.9, 10.2 Hz, 1H), 3.57 (dd, *J* = 15.5, 11.7 Hz, 1H), 3.43 (dd, *J* = 11.8, 3.1 Hz, 1H), 3.30 (dd, *J* = 11.9, 3.6 Hz, 1H), 2.95 (m, 1H), 2.85–2.76 (m, 2H), 1.99–1.92 (m, 2H, contains d, 1.94, *J* = 14.4 Hz, 1H), 1.85–1.61 (complex, 4H), 1.58–0.92 (complex, 25H, contains d, 0.96, *J* = 6.9 Hz, 3H), 0.90–0.84 (complex, 12H, contains t, 0.88, *J* = 3.3 Hz, 6H; d, 0.85, *J* = 2.0 Hz, 3H; d, 0.84, *J* = 1.9 Hz, 3H), 0.70 (m, 1H), 0.63 (s, 3H); ^{13}C NMR (101 MHz, $CDCl_3$) δ 177.2, 63.2, 56.5, 56.4, 54.3, 50.5, 45.5, 43.8, 42.4, 40.4, 40.08, 40.05, 39.6, 38.5, 36.2, 35.9, 34.9, 34.2, 32.1, 31.0, 28.3, 28.1, 24.2, 23.9, 22.9, 22.7, 21.4, 18.8, 15.3, 12.2, 12.1. HRMS (FT-ICR, ESI) *m/z*: [M + H]$^+$ calcd for $C_{31}H_{56}NO_2$ 474.4306, found 474.4306.

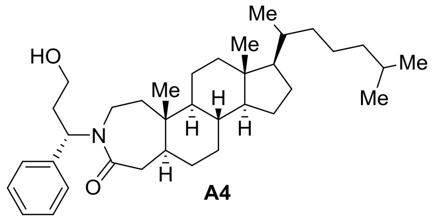

A4

5α-Cholestane-derived A-Ring Lactam, A4

Following the general procedure A, (*S*)-3-azido-3-phenylpropanol (*S*)-**9** (44.2 mg, 0.249 mmol, 2.0 equiv) was reacted with 5α-cholestan-3-one **1** (48.4 mg, 0.125 mmol) to give **A4** as a cream-colored amorphous solid (59.1 mg, 0.110 mmol, 88% yield). Purification was carried out by an automated MPLC system using a 12 g normal phase silica column with gradient elution from 0–50% EtOAc/hexanes. R_f = 0.39 (50% EtOAc/hexanes); mp 149–152 ˚C; IR (neat) 3416, 1618 cm^{-1}; ^{1}H NMR (400 MHz, $CDCl_3$) δ 7.36–7.24 (m, 5H), 5.88 (dd, *J* = 12.4, 3.3 Hz, 1H), 3.70 (m, 1H), 3.40 (m, 1H), 3.18 (dd, *J* = 15.3, 11.6 Hz, 1H), 2.82 (m, 2H), 2.13–2.02 (m, 2H, contains d, 2.06, *J* = 14.9 Hz, 1H), 1.95–1.74 (m, 3H), 1.66–1.43 (m, 5H), 1.37–0.87 (complex, 18H), 0.84 (dd, *J* = 6.5, 2.2 Hz, 9H), 0.77 (s, 3H), 0.57 (s, 3H), 0.47 (m, 1H), 0.38 (m, 1H); ^{13}C NMR (101 MHz, $CDCl_3$) δ 177.5, 139.1, 128.63 (2C), 128.55 (2C), 127.9, 58.5, 56.38, 56.32, 54.2, 52.5, 43.6, 42.3, 40.6, 40.4, 40.0, 39.6, 39.2, 38.1, 36.2, 35.8, 34.8, 32.0, 31.0. 28.3, 28.1, 24.2, 23.9, 22.9, 22.6, 21.1, 18.7, 12.0 (2C). **Note**: APT and HSQC are included in the spectra section. HRMS (FT-ICR, ESI) *m/z*: [M + H]$^+$ calcd for $C_{36}H_{58}NO_2$ 536.4462, found 536.4466.

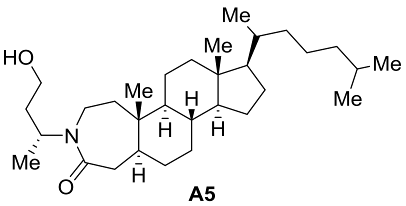

A5

5α-Cholestane-derived A-Ring Lactam, A5

Following the general procedure A, (*R*)-3-azidobutanol (*R*)-**10** (29.5 mg, 0.256 mmol, 2.0 equiv) was reacted with 5α-cholestan-3-one **1** (48.9 mg, 0.126 mmol) to give **A5** as a white amorphous solid (54.7 mg, 0.115 mmol, 92% yield). Purification was carried out by an automated MPLC system using a 12 g normal phase silica with gradient elution from 0–5% MeOH/CH_2Cl_2. R_f = 0.43 (5% MeOH/CH_2Cl_2); mp 142–146 ˚C; IR (neat) 3403, 1620 cm^{-1}; ^{1}H NMR (400 MHz, $CDCl_3$) δ 4.76 (m, 1H), 3.52 (ddd, *J* = 12.0, 5.0, 2.5 Hz, 1H), 3.30–3.20 (m, 2H), 2.98 (m, 1H), 2.82 (dd, *J* = 15.0, 10.8 Hz, 1H), 2.04 (d, *J* = 15.0 Hz, 1H), 1.97 (m, 1H), 1.87–1.77 (m, 2H), 1.73–1.64 (m, 2H), 1.59–0.85 (complex, 38H), 0.74–0.67 (m, 1H), 0.65 (s, 3H); ^{13}C NMR (101 MHz, $CDCl_3$) δ 177.7, 58.6, 56.5, 56.4, 54.4, 45.6, 43.8, 42.4, 42.0, 40.3, 40.1, 39.6, 38.5, 38.1, 36.6, 36.3, 35.9, 34.9, 32.1, 31.0, 28.4, 28.2, 24.3, 24.0, 23.0, 22.7, 21.4, 18.9, 18.8, 12.3, 12.2. HRMS (FT-ICR, ESI) *m/z*: $[M + H]^+$ calcd for $C_{31}H_{56}NO_2$ 474.4306, found 474.4309.

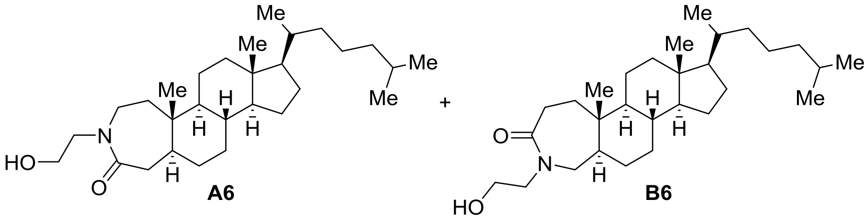

A6 B6

5α-Cholestane-derived A-Ring Lactams, A6 and B6

Following the general procedure A, 2-azidoethanol **12** (26.6 mg, 0.306 mmol, 2.0 equiv) was reacted with 5α-cholestan-3-one **1** (58.4 mg, 0.151 mmol) to give a 37:63 mixture of regioisomer **A6** and **B6** as a white amorphous solid (63.8 mg, 0.143 mmol, 95% yield). Purification was carried out by an automated MPLC system using a 4 g normal phase column with gradient elution from 0–5% MeOH/CH_2Cl_2. R_f = 0.30 (5% MeOH/CH_2Cl_2); IR (neat) 3391, 1623 cm^{-1}; key ^{1}H NMR (400 MHz, $CDCl_3$) δ 3.04 (ddd, *J* = 15.6, 6.4, 1.7 Hz, 1H), 2.79 (dd, *J* = 14.3, 10.8 Hz, 1H), 2.65 (t, *J* = 13.2 Hz, 1H), 2.58 (d, *J* = 15.5 Hz, 1H), 2.34 (ddd, *J* = 14.6, 7.7, 1.3 Hz, 1H); ^{13}C NMR (101 MHz, $CDCl_3$) δ 177.34, 177.26, 61.9, 56.5, 56.3, 54.1, 53.9, 53.1, 52.0, 51.8, 48.7, 46.6, 43.7, 42.40, 42.37, 41.0, 40.2, 40.02, 40.00, 39.6, 38.4, 38.2, 36.2, 35.9, 35.8, 35.1, 34.8, 32.3, 32.1, 31.9, 31.0, 28.4, 28.3, 28.1, 24.2, 23.9, 22.9, 22.6, 21.3, 21.1, 18.7, 12.2, 12.12, 12.09. **Note**: Missing carbon signals due to signal overlap of regioisomers. COSY and HSQC are included in the spectra section. HRMS (FT-ICR, ESI) *m/z*: $[M + Na]^+$ calcd for $C_{29}H_{51}NNaO_2$ 468.3812, found 468.3824.

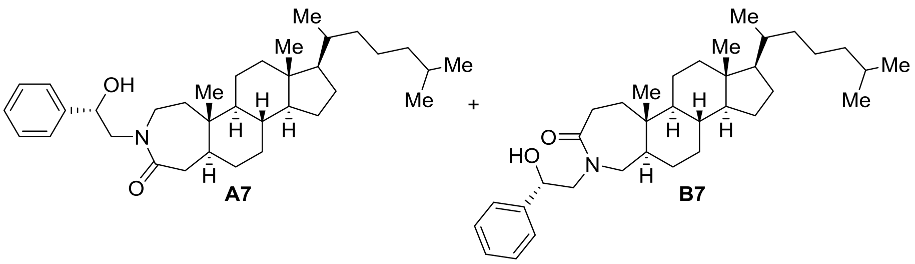

A7 B7

5α-Cholestane-derived A-Ring Lactams, A7 and B7

Following the general procedure A, (*S*)-2-azido-1-phenylethanol (*S*)-**13** (41.0 mg, 0.251 mmol, 2.0 equiv) was reacted with 5α-cholestan-3-one **1** (48.3 mg, 0.125 mmol) to give a 46:54 mixture of regioisomer **A7** and **B7** as a white amorphous solid (60.4 mg, 0.116 mmol, 93% yield). Purification

was carried out by an automated MPLC system using a 12 g normal phase column with gradient elution from 0–5% MeOH/CH_2Cl_2. R_f = 0.47 (4% MeOH/CH_2Cl_2); IR (neat) 3303, 1615 cm^{-1}; key ^{1}H NMR (400 MHz, $CDCl_3$) δ 7.40–7.32 (m, 8H), 7.30–7.26 (m, 2H), 5.10 (d, *J* = 4.8 Hz, 1H), 4.99–4.93 (m, 2H), 4.55 (d, *J* = 4.3 Hz, 1H), 3.78–3.71 (m, 2H), 3.68–3.55 (m, 3H), 3.45 (dd, *J* = 15.8, 11.8 Hz, 1H), 2.80–2.68 (m, 2H), 2.61 (t, *J* = 13.8 Hz, 1H), 2.38–2.34 (m, 1H), 2.31 (d, *J* = 14.9 Hz, 1H), 1.98–1.94 (m, 3H); ^{13}C NMR (101 MHz, $CDCl_3$) δ 178.8, 178.5, 142.7, 142.6, 128.6 (2C), 128.5 (2C), 127.62, 127.60, 126.0 (2C), 125.9 (2C), 74.5, 74.2, 59.8, 58.4, 56.6, 56.5, 56.4, 54.8, 54.1, 53.8, 47.9, 47.6, 43.6, 42.5, 42.4, 40.9, 40.10, 40.07, 40.03, 39.7, 38.4, 38.0, 36.3, 35.91, 35.90, 35.6, 35.0, 34.9, 32.2, 32.1, 31.6, 31.0, 28.34, 28.31, 28.1, 24.3, 23.96, 23.95, 23.0, 22.7, 21.3, 21.1, 18.8, 12.15, 12.13. **Note**: Missing one carbon signal due to signal overlap. HRMS (FT-ICR, ESI) *m/z*: $[M + H]^+$ calcd for $C_{35}H_{56}NO_2$ 522.4306, found 522.4306.

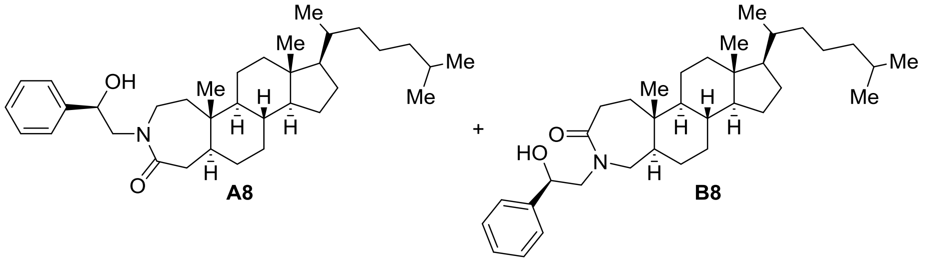

5α-Cholestane-derived A-Ring Lactams, A8 and B8

Following the general procedure A, (*R*)-2-azido-1-phenylethanol (*R*)-**13** (41.0 mg, 0.251 mmol, 2.0 equiv) was reacted with 5α-cholestan-3-one **1** (49.0 mg, 0.127 mmol) to give a 33:67 mixture of regioisomer **A8** and **B8** as a white amorphous solid (58.1 mg, 0.111 mmol, 88% yield). Purification was carried out by an automated MPLC system using a 12 g normal phase column with gradient elution from 0–5% MeOH/CH_2Cl_2. The mixture of isomers was separated by using a 12 g normal phase column with 0–50% EtOAc/hexanes to give **B8** as a white amorphous solid (32.2 mg, 0.0617 mmol, 49% yield) as the major regioisomer and **A8** as a white amorphous solid (17.0 mg, 0.0326 mmol, 26% yield) as the minor regioisomer. **B8**: R_f = 0.25 (50% EtOAc/hexanes); mp 210–213 ˚C; IR (neat) 3346, 1611 cm^{-1}; ^{1}H NMR (400 MHz, $CDCl_3$) δ 7.39–7.33 (m, 4H), 7.31–7.25 (m, 1H), 4.93 (m, 1H), 4.32 (d, *J* = 4.3 Hz, 1H), 3.64 (m, 2H), 3.54 (m, 1H), 2.62 (t, *J* = 13.4 Hz, 1H), 2.39–2.29 (m, 2H, contains d, 2.31, *J* = 15.7 Hz, 1H), 1.96 (m, 1H), 1.89–1.77 (m, 2H), 1.69–1.62 (m, 2H), 1.58–1.46 (m, 3H), 1.38–0.91 (complex, 17H), 0.90–0.83 (complex, 12H, contains d, 0.89, *J* = 6.5 Hz, 3H; d, 0.87, *J* = 1.9 Hz, 3H; d, 0.85, *J* = 1.9 Hz, 3H; s, 0.83, 3H), 0.71–0.65 (m, 1H), 0.63 (s, 3H); ^{13}C NMR (101 MHz, $CDCl_3$) δ 178.3, 142.5, 128.6 (2C), 127.3, 126.0 (2C), 74.3, 58.8, 56.5, 56.4, 54.5, 53.9, 48.6, 42.5, 40.0, 39.7, 38.2, 36.3, 35.9, 35.7, 35.1, 32.2, 31.8, 28.6, 28.3, 28.2, 24.3, 24.0, 23.0, 22.7, 21.1, 18.8, 12.2. **Note**: Missing one carbon signal due to signal overlap. HRMS (FT-ICR, ESI) *m/z*: $[M + H]^+$ calcd for $C_{35}H_{56}NO_2$ 522.4306, found 522.4310. **A8**: R_f = 0.36 (50% EtOAc/hexanes); mp 162–165 ˚C; IR (neat) 3358, 1621 cm^{-1}; ^{1}H NMR (400 MHz, $CDCl_3$) δ 7.40–7.33 (m, 4H), 7.28–7.24 (m, 1H), 4.92 (m, 1H), 4.83 (m, 1H), 3.73–3.62 (m, 2H), 3.55 (dd, *J* = 14.3, 2.6 Hz, 1H), 2.85 (m, 1H), 2.76 (m, 1H), 1.97–1.92 (m, 2H, contains d, 1.94, *J* = 14.6 Hz, 1H), 1.86–1.77 (m, 1H), 1.65 (m, 1H), 1.56–0.93 (complex, 21H), 0.89–0.82 (complex, 12H, contains d, 0.89, *J* = 6.4 Hz, 3H; d, 0.87, *J* = 2.0 Hz, 3H; d, 0.85, *J* = 2.0 Hz, 3H; 0.82, s, 3H) 0.72–0.66 (m, 1H), 0.62 (s, 3H), 0.61–0.54 (m, 1H); ^{13}C NMR (101 MHz, $CDCl_3$) δ 178.6, 142.7, 128.6 (2C), 127.6, 126.0 (2C), 74.2, 59.2, 56.6, 56.4, 54.1, 48.0, 43.4, 42.4, 40.1, 39.9, 39.7, 38.3, 36.3, 35.9, 34.8, 32.1, 31.0, 29.8, 28.4, 28.1, 24.3, 24.0, 22.9, 22.7, 21.3 18.8, 12.19, 12.12. HRMS (FT-ICR, ESI) *m/z*: $[M + H]^+$ calcd for $C_{35}H_{56}NO_2$ 522.4306, found 522.4308.

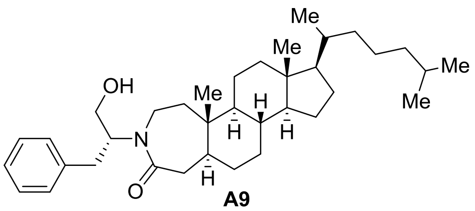

A9

5α-Cholestane-derived A-Ring Lactam, A9

Following the general procedure A, (*R*)-2-azido-3-phenylpropanol (*R*)-**14** (53.5 mg, 0.302 mmol, 2.0 equiv) was reacted with 5α-cholestan-3-one **1** (57.9 mg, 0.150 mmol) to give **A9** as a white amorphous solid (52.4 mg, 0.0978 mmol, 65% yield) as the major regioisomers; an uncharacterized, impure minor product was also obtained in this case (17.8 mg, 0.033 mmol, 22% yield). Purification was carried out by an automated MPLC system using a 12 g normal phase column with gradient elution from 0–100% EtOAc/Ether. The major isomer was subjected to a second purification using a 4 g normal phase column with gradient elution from 0–5% MeOH/CH_2Cl_2. R_f = 0.29 (25% EtOAc/Ether); mp 191–194 ˚C; IR (neat) 3415, 1620 cm^{-1}; ^1H NMR (400 MHz, $CDCl_3$) δ 7.31–7.26 (m, 2H), 7.23–7.18 (m, 3H), 4.38 (br s, 1H), 3.71 (m, 2H), 3.31 (dd, *J* = 15.7, 11.7 Hz, 1H), 2.99–2.73 (m, 3H), 2.75 (m, 1H), 1.95–1.89 (m, 2H, contains d, 1.91, *J* = 14.3 Hz, 1H), 1.80 (m, 1H), 1.65–1.46 (m, 4H), 1.42–0.93 (complex, 19H), 0.89–0.83 (complex, 11H, contains d, 0.88, *J* = 6.4 Hz, 3H; 0.86, d, *J* = 2.0 Hz, 3H; 0.85, d, *J* = 2.0 Hz, 3H), 0.79 (s, 3H), 0.61 (s, 3H), 0.50 (m, 1H); ^{13}C NMR (126 MHz, $CDCl_3$) δ 177.7, 138.5, 129.1 (2C), 128.6 (2C), 126.5, 64.0, 56.4, 56.3, 54.2, 43.6, 42.4, 41.2, 40.9, 40.0, 39.6, 38.2, 36.2, 35.9, 35.0, 34.8, 32.1, 31.0, 28.3, 28.1, 24.2, 23.9, 22.9, 22.7, 21.3, 18.7, 12.2, 12.1. **Note**: Missing two carbon signals due to signal overlap. X-ray crystal structure of this analog is provided in the CCDC (CCDC 1583534). HRMS (FT-ICR, ESI) *m/z*: $[M + H]^+$ calcd for $C_{36}H_{58}NO_2$ 536.4462, found 536.4471.

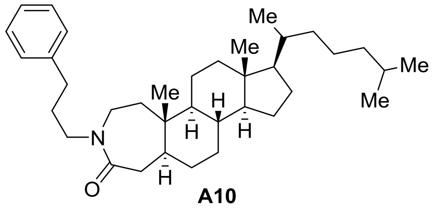

A10

5α-Cholestane-derived A-Ring Lactam, A10

A solution of **A2** (53.6 mg, 0.100 mmol), 10% Pd/C (42.6 mg, 0.400 mmol, 4.0 equiv), and acetic acid (2.0 mL) in anhydrous EtOH (15.0 mL) was placed under hydrogen atmosphere (40 psi) via a shaker-Parr hydrogenation apparatus over 24 h. The solution was filtered over Celite and concentrated. The crude residue was purified by an automated MPLC system using a 12 g normal phase silica column with gradient elution from 0–60% EtOAc/hexanes. Concentration of solvents afforded product **A10** as white amorphous solid (48.6 mg, 0.0935 mmol, 94% yield). R_f = 0.68 (50% EtOAc/hexanes); mp 146–147 ˚C; IR (neat) 1624 cm^{-1}; ^1H NMR (400 MHz, $CDCl_3$) δ 7.31–7.27 (m. 2H), 7.20–7.16 (m, 3H), 3.58 (dd, *J* = 11.7, 15.6 Hz, 1H), 3.46–3.33 (m, 2H), 2.95 (dd, *J* = 15.4, 6.2 Hz, 1H), 2.74 (dd, *J* = 14.2, 10.3 Hz, 1H), 2.62 (m, 2H), 1.98–1.91 (m, 2H, contains d, *J* = 14.3 Hz, 1H), 1.87–1.75 (m, 3H), 1.67–1.63 (m, 2H), 1.59–0.92 (complex, 22H), 0.89–0.84 (complex, 12H), 0.71–0.64 (m, 4H, contains s, 0.64, 3H); ^{13}C NMR (101 MHz, $CDCl_3$) δ 175.5, 142.0 128.52 (2C), 128.46 (2C), 126.0, 56.5. 56.4, 54.3, 47.9, 45.0, 43.8, 42.5, 41.5, 40.5, 40.1, 39.7, 38.4, 36.3, 35.9, 34.9, 33.5, 32.1, 31.1, 30.1, 28.4, 28.1, 24.3, 23.9, 22.9, 22.7, 21.4, 18.8, 12.18, 12.16. HRMS (FT-ICR, ESI) *m/z*: $[M + H]^+$ calcd for $C_{36}H_{57}NO$ 520.4513, found 520.4523.

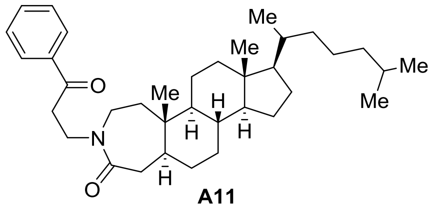

A11

5α-Cholestane-derived A-Ring Lactam, A11

Following the general procedure C, **A2** (530 mg, 0.099 mmol) was oxidized to give product **A11** as a white amorphous solid (41.2 mg, 0.077 mmol, 78% yield). Purification was carried out by an automated MPLC system using a 12 g normal phase silica column with gradient elution from 0–25% EtOAc/hexanes. R_f = 0.63 (50% EtOAc/hexanes); mp 209–214 ˚C; IR (neat) 1683, 1642 cm^{-1}; ^1H NMR (400 MHz, $CDCl_3$) δ 7.98 (m, 2H), 7.56 (m, 1H), 7.47 (m, 2H), 3.79 (dt, *J* = 13.4, 6.7 Hz, 1H), 3.72–3.61 (m, 2H), 3.26 (m, 2H), 3.19 (m, 1H), 2.74 (dd, *J* = 14.2, 10.2 Hz, 1H), 1.98–1.93 (m, 1H), 1.90 (d, *J* = 14.3 Hz, 1H), 1.85–1.76 (m, 2H), 1.66–0.91 (complex, 23H), 0.83–0.90 (complex, 12H), 0.68–0.61 (m, 4H, contains s, 0.64, 3H); ^{13}C NMR (101 MHz, $CDCl_3$) δ 199.4, 175.9, 136.9, 133.4, 128.8 (2C), 128.4 (2C), 56.5, 56.4, 54.3, 46.7, 45.4, 43.7, 42.5, 41.4, 40.5, 40.1, 39.7, 38.4, 37.8, 36.3, 35.9, 34.9, 32.1, 31.1, 28.4, 28.1, 24.3, 24.0, 23.9, 22.9, 22.7, 21.4, 18.8, 12.2. HRMS (FT-ICR, ESI) *m/z*: [M + H]$^+$ calcd for $C_{36}H_{56}NO_2$ 534.4306, found 534.4313.

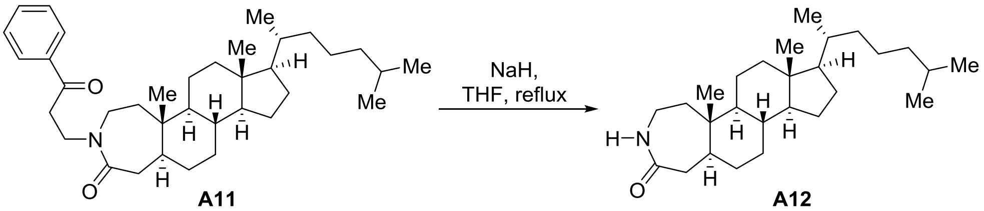

5α-Cholestane-derived A-Ring Lactam, A12

A solution of **A11** (54.3 mg, 0.102 mmol) and sodium hydride (60% dispersion in mineral oil, 20.0 mg, 0.814 mmol, 4.8 equiv) in anhydrous THF (8.0 mL) was heated at 65 ˚C for 2 h. The reaction was cooled to room temperature and quenched with a saturated solution of NH_4Cl (5 mL). The reaction mixture was diluted with CH_2Cl_2 (20 mL) and washed with saturated solution of NH_4Cl (2 x 5 mL), H_2O (5 mL) and brine (5 mL). The combined organic layer was dried over anhydrous Na_2SO_4 filtered, and concentrated. The crude residue was purified by an automated MPLC system using a 12 g normal phase silica column with gradient elution from 0–5% MeOH/CH_2Cl_2. Concentration of solvents afforded product **A12** as white amorphous solid (29.2 mg, 0.073 mmol, 72% yield). R_f = 0.23 (2% MeOH/CH_2Cl_2); mp 287–290 ˚C (decomposed); IR (neat) 3315, 3193, 1676, 1628 cm^{-1}; ^1H NMR (400 MHz, $CDCl_3$) δ 5.77 (br s, 1H), 3.39 (m, 1H), 2.98 (m, 1H), 2.75 (dd, *J* = 14.3, 11.0 Hz, 1H), 1.97 (dt, *J* = 12.6, 3.4 Hz, 1H), 1.88–1.77 (m, 3H), 1.66 (m, 1H), 1.57–0.94 (complex, 20H), 0.93–0.85 (complex, 14H), 0.74 (m, 1H), 0.65 (s, 3H); ^{13}C NMR (101 MHz, $CDCl_3$) δ 178.6, 56.5, 56.3, 54.2, 43.4, 42.4, 42.2, 40.1, 39.74, 39.65, 39.0, 38.1, 36.3, 35.9, 34.8, 32.2, 31.2, 28.4, 28.2, 24.3, 24.0, 23.0, 22.7, 21.4, 18.8, 12.16, 12.11. HRMS (FT-ICR, ESI) *m/z*: [M + H]$^+$ calcd for $C_{27}H_{48}NO$ 402.3730, found 402.3734.

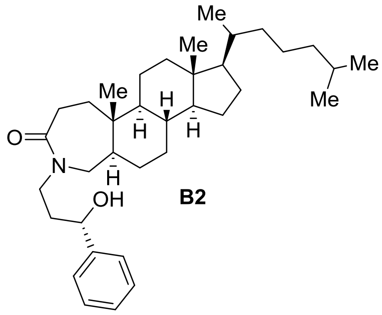

B2

5α-Cholestane-derived A-Ring Lactam, B2

Following the general procedure A, (*S*)-3-azido-1-phenylpropanol (*S*)-**7** (86.6 mg, 0.500 mmol, 2.0 equiv) was reacted with 5α-cholestan-3-one **1** (96.6 mg, 0.250 mmol) to give **B2** as a white amorphous solid (118 mg, 0.220 mmol, 88% yield). Purification was carried out by an automated MPLC system using a 12 g normal phase silica column 0–40% EtOAc/hexanes. R_f = 0.42 (50% EtOAc/hexanes); mp 194–199 ˚C; IR (neat) 3378, 1619 cm^{-1}; ^{1}H NMR (400 MHz, $CDCl_3$) δ 7.37–7.33 (m, 4H) 7.26 (m, 1H), 4.64 (dd, *J* = 9.8, 3.2 Hz, 1H), 4.10 (ddd, *J* = 14.6, 10.7, 4.3 Hz, 1H), 3.70 (dd, *J* = 15.5, 8.5 Hz, 1H), 3.11 (dt, *J* = 14.1, 4.5 Hz, 1H), 2.61–2.52 (m, 2H, contains d, 2.54, *J* = 15.5 Hz, 1H), 2.39 (m, 1H), 2.04–1.72 (complex, 6H), 1.61–1.47 (complex, 3H), 1.39–0.94 (complex, 19H), 0.91–0.85 (complex, 12H, contains s, 0.91, 3H; s, 0.89, 3H; d, 0.87, *J* = 1.9 Hz, 3H; d, 0.85, *J* = 1.9 Hz, 3H), 0.74–0.65 (m, 4H, contains s, 0.65, 3H); ^{13}C NMR (101 MHz, $CDCl_3$) δ 177.1, 144.4, 128.5 (2C), 127.1, 125.6 (2C), 70.1, 56.5, 56.3, 54.3, 52.5, 49.6, 45.9, 42.5, 40.0, 39.7, 38.4, 38.1, 36.3, 36.2, 35.9, 35.2, 32.2, 28.5, 28.3, 28.1, 24.3, 24.0, 22.9, 22.7, 21.1, 18.8, 12.2. **Note**: Missing two carbon signals due to signal overlap of methyl groups. APT and HSQC are included in the spectra section. HRMS (FT-ICR, HESI) *m/z*: [M + H]$^+$ calcd for $C_{36}H_{58}NO_2$ 536.4462, found 536.4449.

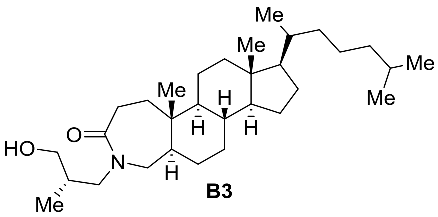

B3

5α-Cholestane-derived A-Ring Lactam, B3

Following the general procedure A, (*S*)-3-azido-2-methylpropanol (*S*)-**8** (34.7 mg, 0.300 mmol, 2.0 equiv) was reacted with 5α-cholestan-3-one **1** (58.0 mg, 0.150 mmol) to give **B3** as a white amorphous solid (59.5 mg, 0.126 mmol, 84% yield). Purification was carried out twice by an automated MPLC system first using a 12 g normal phase silica column with gradient elution from 0–40% EtOAc/Et_2O, and second using a 4 g column normal phase silica column with gradient elution from 0–5% MeOH/CH_2Cl_2. R_f = 0.35 (2% MeOH/CH_2Cl_2); mp 186–187 ˚C; IR (neat) 3417, 1623 cm^{-1}; ^{1}H NMR (400 MHz, $CDCl_3$) δ 3.68 (dd, *J* = 13.9, 10.1 Hz, 1H), 3.58 (m, 1H), 3.41 (dd, *J* = 11.9, 3.1 Hz, 1H), 3.28 (dd, *J* = 11.8, 3.7 Hz, 1H), 2.84 (dd, *J* = 13.9, 4.4 Hz, 1H), 2.64 (t, *J* = 14.0 Hz, 1H), 2.45 (d, *J* = 15.3 Hz, 1H), 2.33 (m, 1H), 1.95 (m, 1H), 1.88–1.65 (complex, 4H), 1.57–1.42 (m, 3H), 1.37–0.90 (complex, 22H, contains d, 0.95, *J* = 6.9 Hz, 3H), 0.88–0.80 (complex, 12H, contains d, 0.85, *J* = 1.9 Hz, 3H; d, 0.83, *J* = 1.9 Hz, 3H), 0.69 (m, 1H), 0.62 (s, 3H); ^{13}C NMR (101 MHz, $CDCl_3$) δ 177.2, 63.2, 56.5, 56.3, 54.0, 52.2, 50.9, 48.4, 42.5, 40.0, 39.6, 38.3, 36.3, 35.9, 35.8, 35.1, 34.3, 32.1, 31.9, 28.6, 28.3, 28.1, 24.3, 23.9, 22.9, 22.7, 21.1, 18.7, 15.2, 12.3, 12.1. HRMS (FT-ICR, ESI) *m/z*: [M + H]$^+$ calcd for $C_{31}H_{56}NO_2$ 474.4306, found 474.4305.

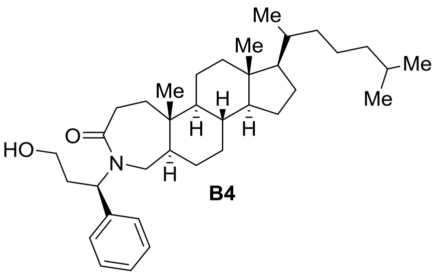

B4

5α-Cholestane-derived A-Ring Lactam, B4

Following the general procedure A, (*R*)-3-azido-3-phenylpropanol (*R*)-**9** (44.3 mg, 0.250 mmol) was reacted with 5α-cholestan-3-one **1** (48.3 mg, 0.125 mmol) to give **B4** as a white amorphous solid (58.4 mg, 0.109 mmol, 87% yield). Purification was carried out by an automated MPLC system using a 12 g normal phase silica column with gradient elution from 0–50% EtOAc/hexanes. R_f = 0.43 (50% EtOAc/hexanes); mp 187–189 ˚C; IR (neat) 3409, 1620 cm^{-1}; ^{1}H NMR (400 MHz, $CDCl_3$) δ 7.37–7.28 (m, 5H), 5.92 (dd, *J* = 12.5, 3.1 Hz, 1H), 3.72 (m, 1H), 3.42 (td, *J* = 11.7, 2.8 Hz, 1H), 3.18 (dd, *J* = 15.2, 9.0 Hz, 1H), 2.69 (t, *J* = 14.0 Hz, 1H), 2.47 (dd, *J* = 15.0, 7.4 Hz, 1H), 2.35 (d, *J* = 15.3 Hz, 1H), 2.09 (m, 1H), 1.95–1.83 (m, 3H), 1.79–1.71 (m, 1H), 1.53–0.82 (complex, 29 H), 0.77 (s, 3H), 0.58 (s, 3H), 0.52–0.43 (m, 2H), 0.34–0.23 (m, 2H); ^{13}C NMR (126 MHz, $CDCl_3$) δ 177.2, 139.1, 129.0 (2C), 128.5 (2C), 128.1, 58.5, 56.4 (2C), 54.0, 52.8, 48.8, 45.4, 42.4, 40.0, 39.6, 38.0, 36.3, 36.1, 35.9, 35.1, 32.6, 32.3, 31.6, 28.3, 28.1, 27.4, 24.2, 23.9, 22.9, 22.7, 21.0, 18.8, 12.10, 12.06. **Note**: APT and HSQC are included in the spectra section. HRMS (FT-ICR, HESI) *m/z*: $[M + H]^+$ calcd for $C_{36}H_{58}NO_2$ 536.4462, found 436.4456.

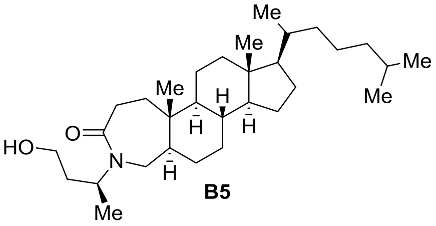

B5

5α-Cholestane-derived A-Ring Lactam, B5

Following the general procedure A, (*S*)-3-azidobutanol (*S*)-**10** (28.5 mg, 0.248 mmol, 2.0 equiv) was reacted with 5α-cholestan-3-one **1** (48.6 mg, 0.126 mmol) to give **B5** as a white amorphous (52.8 mg, 0.111 mmol, 88% yield). Purification was carried out by an automated MPLC system using a 12 g normal phase silica column with gradient elution from 0–5% MeOH/CH_2Cl_2. R_f = 0.37 (5% MeOH/CH_2Cl_2); mp 145–149 ˚C; IR (neat) 3338, 1620 cm^{-1}; ^{1}H NMR (400 MHz, $CDCl_3$) δ 4.78 (m, 1H), 3.51 (ddd, *J* = 12.1, 5.1, 2.5 Hz, 1H), 3.29–3.21 (m, 2H), 2.68 (t, *J* = 14.1 Hz, 1H), 2.50–2.40 (m, 2H, contains d, *J* = 2.48, 15.6 Hz, 1H), 1.98 (m, 1H), 1.91–1.77 (m, 2H), 1.74–1.44 (complex, 5H), 1.38–0.81 (complex, 35H), 0.70 (m, 1H), 0.64 (s, 3H); ^{13}C NMR (101 MHz, $CDCl_3$) δ 177.5, 58.6, 56.5, 56.3, 54.1, 49.9, 45.6, 44.4, 42.5. 40.0, 39.7, 38.4, 36.8, 36.3, 35.93, 35.92, 35.2, 32.5, 32.0, 28.4, 28.2, 27.6, 24.3, 24.0, 23.0, 22.7, 21.1, 18.9, 18.8, 12.3, 12.2. HRMS (FT-ICR, ESI) *m/z*: $[M + H]^+$ calcd for $C_{31}H_{56}NO_2$ 474.4306, found 474.4313.

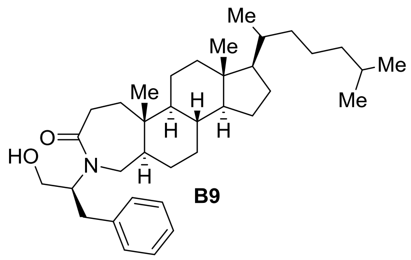

B9

5α-Cholestane-derived A-Ring Lactam, B9

Following the general procedure A, (*S*)-2-azido-3-phenylpropanol (*S*)-**14** (53.4 mg, 0.301 mmol, 2.0 equiv) was reacted with 5α-cholestan-3-one **1** (58.1 mg, 0.150 mmol) to give **B9** as a white amorphous solid (65.1 mg, 0.121 mmol, 81% yield) as the major regioisomers, in addition an uncharacterized, impure minor product was obtained (10.0 mg, 0.019 mmol, 12% yield). Purification was carried out by an automated MPLC system using a 12 g normal phase column with gradient elution from 0–5% MeOH/CH_2Cl_2. R_f = 0.23 (2% MeOH/CH_2Cl_2); mp 203–204 ˚C; IR (neat) 3415, 1618 cm^{-1}; ^{1}H NMR (400 MHz, $CDCl_3$) δ 7.31–7.26 (m, 2H), 7.24–7.20 (m, 3H), 4.64 (br s, 1H), 3.74 (dd, *J* = 11.6, 3.6 Hz, 1H), 3.64 (dd, *J* = 11.6, 7.7 Hz, 1H), 3.34 (dd, *J* = 15.6, 9.2 Hz, 1H), 2.89 (m, 2H), 2.61 (t, *J* = 13.8 Hz, 1H), 2.44 (d, *J* = 15.6 Hz, 1H), 2.33 (dd, *J* = 15.0, 7.2 Hz, 1H), 1.93 (m, 1H), 1.85–1.74 (m, 4H), 1.37–0.94 (complex, 20H), 0.91–0.81 (complex, 9H, contains s, 0.89, 3H; d, 0.87, *J* = 2.1 Hz, 3H; d, 0.85, *J* = 1.9 Hz, 3H), 0.79 (s, 3H), 0.74–0.67 (m, 1H), 0.61 (s, 3H), 0.42 (m, 1H); ^{13}C NMR (126 MHz, $CDCl_3$) δ 177.6, 138.2, 128.8 (2C), 128.6 (2C), 126.6, 64.1, 56.4, 56.3, 53.8, 48.4, 42.4, 39.9, 39.6, 37.9, 36.2, 35.9, 35.7, 35.03, 34.99, 33.0, 31.7, 28.3, 28.2, 28.1, 24.2, 23.9, 23.0, 21.0, 18.7, 12.13, 12.12. **Note**: Missing three carbon signals due to signal overlap. X-ray crystal structure of this analog is provided in the CCDC (CCDC 1583535). HRMS (FT-ICR, HESI) *m/z*: $[M + H]^+$ calcd for $C_{36}H_{58}NO_2$ 536.4462, found 536.4489.

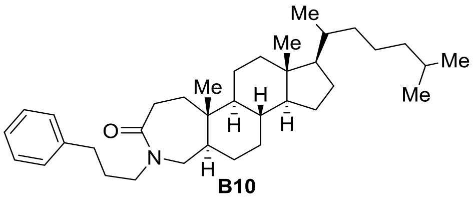

B10

5α-Cholestane-derived A-Ring Lactam, B10

A solution of **B2** (53.6 mg, 0.100 mmol), 10% Pd/C (21.3 mg, 0.200 mmol, 2.00 equiv), and acetic acid (200 µL) in anhydrous EtOH (15.0 mL) was placed under hydrogen atmosphere (40 psi) via a shaker-Parr hydrogenation apparatus over 24 h. The solution was filtered over Celite and concentrated. The crude residue was purified by purified by an automated MPLC system using a 12 g normal phase silica column with gradient elution from 0–60% EtOAc/hexanes. Concentration of solvents afforded product **B10** as white amorphous solid (36.0 mg, 0.0692 mmol, 69% yield). R_f = 0.75 (50% EtOAc/hexanes); mp 155–157 ˚C; IR (neat) 1624 cm^{-1}; ^{1}H NMR (400 MHz, $CDCl_3$) δ 7.31–7.27 (m, 2H), 7.20–7.16 (m, 3H), 3.60 (dd, *J* = 15.4, 8.5 Hz, 1H), 3.40 (m, 2H), 2.66–2.56 (m, 3H), 2.48 (d, *J* = 15.4 Hz, 1H), 2.34 (dd, *J* = 14.4, 7.5 Hz, 1H), 1.97 (m, 1H), 1.88–1.43 (complex, 8H), 1.34–0.93 (complex, 19H), 0.92–0.85 (complex, 12H), 0.71–0.64 (m, 4H, contains s, 0.64, 3H); ^{13}C NMR (101 MHz, $CDCl_3$) δ 175.4, 142.0, 128.53 (2C), 128.45 (2C), 126.0, 56.5, 56.4, 54.1, 51.6, 49.1, 48.0, 42.5, 40.1, 39.7, 38.2. 36.3, 36.0, 35.9, 35.2, 33.5, 32.6, 32.0, 30.1, 28.6, 28.4, 28.2, 24.3, 24.0, 22.9, 22.7, 21.1, 18.8, 12.2, 12.16. HRMS (FT-ICR, ESI) *m/z*: $[M + H]^+$ calcd for $C_{36}H_{57}NO$ 520.4513, found 520.4513.

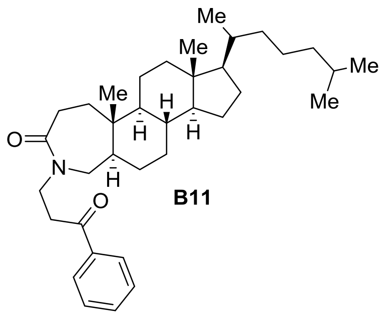

B11

5α-Cholestane-derived A-Ring Lactam, B11

Following the general procedure C, **B2** (536 mg, 0.100 mmol) was oxidized to give **B11** as a white amorphous solid (426 mg, 0.080 mmol, 80% yield). Purification was carried out by an automated MPLC system using a 12 g normal phase silica column with gradient elution from 0–25% EtOAc/hexanes. R_f = 0.25 (25% EtOAc/hexanes); mp 177–179 ˚C; IR (neat) 1678, 1641 cm^{-1}; ^1H NMR (500 MHz, $CDCl_3$) δ 7.98 (m, 2H), 7.56 (m, 1H), 7.47 (m, 2H), 3.82 (dt, *J* = 13.5, 6.7 Hz, 1H), 3.78–3.60 (m, 2H), 3.25 (m, 2H), 2.75 (d, *J* = 15.4 Hz, 1H), 2.59 (t, *J* = 13.1 Hz, 1H), 2.30 (dd, *J* = 13.9, 7.2 Hz, 1H), 1.95 (dt, *J* = 12.6, 3.4 Hz, 1H), 1.86–1.76 (m, 2H), 1.69–1.64 (m, 3H), 1.56–1.47 (m, 3H), 1.43–0.77 (complex, 29H), 0.64–0.59 (m, 4H, contains s, 0.63, 3H); ^{13}C NMR (126 MHz, $CDCl_3$) δ 199.3, 175.9, 136.8, 133.4, 128.8 (2C), 128.3 (2C), 56.5, 56.3, 54.0, 53.2, 49.1, 45.4, 42.4, 40.0, 39.6, 38.2, 37.8, 36.3, 35.9, 35.8, 35.1, 32.5, 31.9, 28.4, 28.1, 24.3, 24.0, 23.0, 22.7, 21.1, 18.8, 12.2, 12.1. **Note**: Missing one carbon signal due to signal overlap. HRMS (FT-ICR, HESI) *m/z*: $[M + H]^+$ calcd for $C_{36}H_{57}NO_2$ 534.4306, found 534.4307.

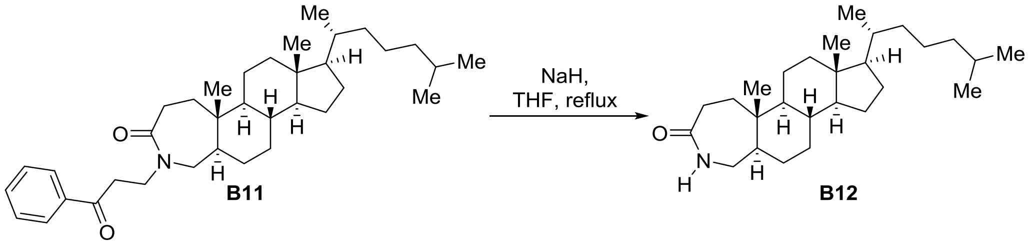

B11 B12

5α-Cholestane-derived A-Ring Lactam, B12

A solution of **B11** (88.6 mg, 0.166 mmol) and sodium hydride (60% dispersion in mineral oil, 35.0 mg, 0.879 mmol, 5.3 equiv) in anhydrous THF (14.0 mL) was heated at 65 ˚C for 2 h. The reaction was cooled to room temperature and quenched with a saturated solution of NH_4Cl (5 mL). The reaction mixture was diluted with CH_2Cl_2 (20 mL) and washed with saturated solution of NH_4Cl (2 x 5 mL), H_2O (5 mL) and brine (5 mL). The combined organic layer was dried over anhydrous Na_2SO_4 filtered, and concentrated. The crude residue was purified by an automated MPLC system using a 12 g normal phase silica column with gradient elution from 0–5% MeOH/CH_2Cl_2. Concentration of solvents afforded product **B12** as white amorphous solid (51.7 mg, 0.129 mmol, 78% yield). R_f = 0.28 (3% MeOH/CH_2Cl_2); mp 301–304 ˚C (decomposed); IR (neat) 3190, 1672, 1627 cm^{-1}; ^1H NMR (400 MHz, $CDCl_3$) δ 5.60 (br s, 1H), 3.37 (m, 1H), 2.63–2.50 (m, 2H), 2.25 (m, 1H), 1.98 (m, 1H), 1.92–1.77 (m, 2H), 1.69 (m, 1H), 1.60–1.48 (m, 3H), 1.38–0.93 (complex, 18H), 0.90–0.85 (complex, 13H), 0.73 (m, 1H), 0.65 (s, 3H); ^{13}C NMR (101 MHz, $CDCl_3$) δ 178.7, 56.6, 56.4, 53.9, 49.8, 44.7, 42.5, 40.1, 39.7, 38.8, 36.3, 35.9, 35.4, 35.2, 31.9, 31.5, 28.4, 28.2, 27.9, 24.3, 24.0, 23.0, 22.7, 21.2, 18.8, 12.20, 12.18. HRMS (FT-ICR, ESI) *m/z*: $[M + H]^+$ calcd for $C_{27}H_{48}NO$ 402.3730, found 402.3732.

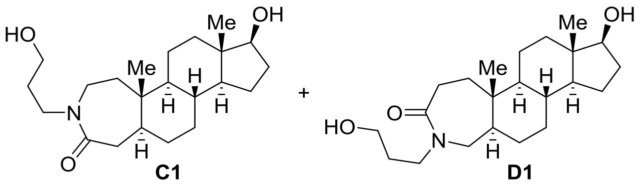

C1 D1

17β-Hydroxy-5α-androstane-derived A-Ring Lactams, C1 and D1

Following the general procedure A, 3-azidopropanol **6** (30.6 mg, 0.303 mmol, 2.0 equiv) was reacted with 5α-DHT **2** (43.6 mg, 0.150 mmol) to give a 40:60 mixture of regioisomers **C1** and **D1** as a white amorphous solid (51.6 mg, 0.142 mmol, 95% yield, UPLC/HRMS purity: ≥99.5%). Purification was carried out by an automated MPLC system using a 12 g normal phase silica column with gradient elution from 0–5% MeOH/CH_2Cl_2. R_f = 0.27 (5% MeOH/CH_2Cl_2); IR (neat) 3325, 1618, 1603 cm^{-1}; key ^{1}H NMR (400 MHz, $CDCl_3$) δ 2.91 (ddd, *J* = 15.6, 6.5, 1.8 Hz, 1H), 2.73 (dd, *J* = 14.2, 10.4 Hz, 1H), 2.59 (t, *J* = 13.0 Hz, 1H), 2.44 (d, *J* = 15.4 Hz, 1H), 2.29 (dd, *J* = 14.9, 6.9 Hz, 1H), 2.04–1.93 (m, 2H), 1.91–1.72 (m, 4H, contains d, *J* = 14.3 Hz, 1H); ^{13}C NMR (126 MHz, $CDCl_3$) δ 177.2, 177.1, 81.90, 81.86, 77.4, 77.2, 76.9, 58.11, 58.06, 54.4, 54.1, 52.0, 51.02, 51.01, 48.9, 45.3, 44.8, 44.5, 44.0, 42.9, 42.8, 41.1, 40.0, 38.6, 38.4, 36.8, 36.7, 36.0, 35.1, 34.9, 32.1, 31.6, 31.5, 30.9, 30.7, 30.6, 30.32, 30.29, 28.4, 23.4, 21.0, 20.7, 12.24, 12.19, 11.25, 11.23. **Note**: Missing carbon signals due to signal overlap of regioisomers. HRMS (FT-ICR, HESI) *m/z*: $[M + H]^+$ calcd for $C_{22}H_{38}NO_3$ 364.2846, found 364.2840.

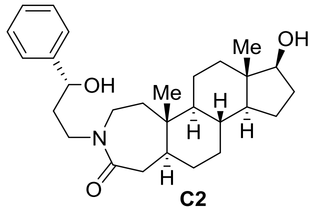

C2

17β-Hydroxy-5α-androstane-derived A-Ring Lactam, C2

Following the general procedure A, (*R*)-3-azido-1-phenylpropanol (*R*)-**7** (44.1 mg, 0.250 mmol, 2.0 equiv) was reacted with 5α-DHT **2** (36.2 mg, 0.125 mmol) to give **C2** as a cream-colored amorphous solid (46.0 mg, 0.105 mmol, 84% yield, UPLC/HRMS purity: 95.7%). Purification was carried out by an automated MPLC system using a 12 g normal phase silica column with gradient elution from 0–5% MeOH/CH_2Cl_2. R_f = 0.33 (5% MeOH/CH_2Cl_2); mp 155–157 ˚C; IR (neat) 3348, 1622 cm^{-1}; ^{1}H NMR (400 MHz, $CDCl_3$) δ 7.39–7.36 (m, 4H), 7.26 (m, 1H), 4.65 (dd, *J* = 9.9, 3.5 Hz, 1H), 4.06 (ddd, *J* = 14.6, 10.5, 4.4 Hz, 1H), 3.69–3.61 (m, 2H), 3.12 (dt, *J* = 14.5, 4.7 Hz, 1H), 3.02 (m, 1H), 2.72 (dd, *J* = 14.1, 10.3 Hz, 1H), 2.11–1.78 (complex, 6H, contains d, 1.98, *J* = 1.9 Hz, 1H), 1.70–1.65 (m, 1H), 1.62–1.54 (m, 2H), 1.51–1.15 (complex, 8H), 1.06 (m, 1H), 0.98–0.85 (complex, 5H, contains s, 0.90, 3H), 0.73–0.68 (complex, 4H, contains s, 0.73, 3H); ^{13}C NMR (126 MHz, $CDCl_3$) δ 177.1, 144.2, 128.4 (2C), 127.2, 125.7 (2C), 81.9, 70.0, 54.5, 51.0, 45.8, 45.7, 44.2, 42.8, 41.9, 40.0, 38.6, 37.8, 36.8, 34.9, 31.6, 30.9, 30.7, 23.4, 21.0, 12.1, 11.2. **Note**: APT, NOE (1D), HSQC and HMBC are included in the spectra section. HRMS (FT-ICR, HESI) *m/z*: $[M + H]^+$ calcd for $C_{28}H_{42}NO_3$ 440.3159, found 440.3162.

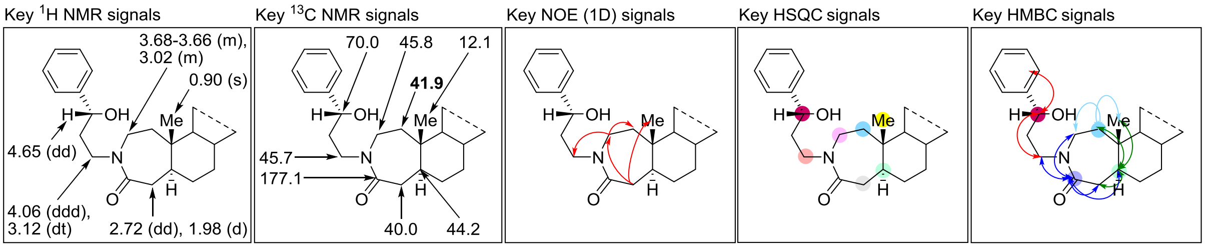

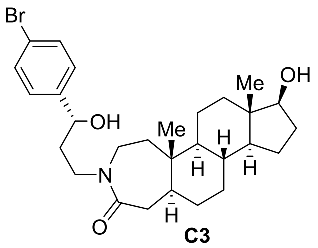

17β-Hydroxy-5α-androstane-derived A-Ring Lactam, C3

Following the general procedure A, (*R*)-3-azido-1-(4-bromophenyl)propanol (*R*)-**11** (64.1 mg, 0.250 mmol, 2.0 equiv) was reacted with 5α-DHT **2** (36.5 mg, 0.126 mmol) to give **C3** as a white amorphous solid (53.9 mg, 0.104 mol, 83% yield, UPLC/HRMS purity: ≥99.5%). Purification was carried by an automated MPLC system using a 12 g normal phase silica column with gradient elution from 0–2% MeOH/CH_2Cl_2. R_f = 0.43 (4% MeOH/CH_2Cl_2); mp 213–217 ˚C; IR (neat) 3348, 1621 cm^{-1}; ^1H NMR (400 MHz, $CDCl_3$) δ 7.46 (m, 2H), 7.25 (m, 2H), 4.59 (dd, *J* = 10.0, 3.2, 1H), 4.06 (ddd, *J* = 14.6, 10.8, 4.0 Hz, 1H), 3.72–3.62 (m, 2H), 3.10 (dt, *J* = 14.2, 4.6 Hz, 1H), 3.02 (m, 1H), 2.73 (dd, *J* = 14.1, 10.4 Hz, 1H), 2.11–2.00 (m, 1H), 1.99 (d, *J* = 14.2 Hz, 1H), 1.94–1.55 (complex, 7H), 1.57–1.03 (complex, 9H), 0.98–0.83 (m, 5H, contains s, 0.93, 3H), 0.74–0.67 (m, 4H, contains s, 0.74, 3H); ^{13}C NMR (101 MHz, $CDCl_3$) δ 177.3, 143.3, 131.5 (2C), 127.5 (2C), 120.9, 81.9, 69.4, 54.6, 51.0, 46.0, 45.7, 44.2, 43.0, 41.9, 40.0, 38.6, 37.9, 36.8, 35.0, 31.6, 30.9, 30.7, 23.4, 21.0, 12.2, 11.2. HRMS (FT-ICR, HESI) *m/z*: $[M + H]^+$ calcd for $C_{28}H_{41}NO_3$ 518.2264, found 518.2319.

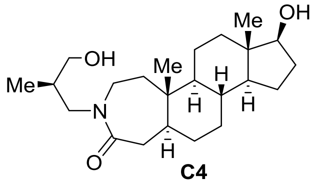

17β-Hydroxy-5α-androstane-derived A-Ring Lactam, C4

Following the general procedure A, (*R*)-3-azido-2-methylpropanol (*R*)-**8** (28.3 mg, 0.246 mmol, 2.0 equiv) was reacted with 5α-DHT **2** (36.4 mg, 0.125 mmol) to give **C4** as a white crystalline solid (40.3 mg, 0.107 mol, 85% yield, UPLC/HRMS purity: ≥99.5%). Purification was carried by an automated MPLC system using a 12 g normal phase silica column with gradient elution from 0–6% MeOH/CH_2Cl_2. R_f = 0.47 (5% MeOH/CH_2Cl_2); mp 215–218 ˚C; IR (neat) 3354, 1617 cm^{-1}; ^1H NMR (400 MHz, $CDCl_3$) δ 3.71–3.65 (dd, *J* = 13.9, 10.1 Hz, 1H), 3.64–3.56 (m, 2H), 3.44 (dd, *J* = 11.8, 3.1 Hz, 1H), 3.31 (dd, *J* = 11.8, 3.8 Hz, 1H), 2.97 (m, 1H), 2.87–2.78 (m, 2H), 2.10–2.00 (m, 1H), 1.97 (d, *J* = 14.6 Hz, 1H), 1.83–1.18 (complex, 13H), 1.05 (m, 1H), 0.97 (d, *J* = 7.0 Hz, 3H), 0.94–0.82 (complex, 5H, contains s, 0.91, 3H), 0.77–0.70 (m, 4H, contains s, 0.73, 3H); ^{13}C NMR (151 MHz, $CDCl_3$) δ 177.3, 82.0, 63.3, 54.4, 51.1, 50.7, 45.6, 43.9, 42.9, 40.4, 40.0, 38.7, 36.9, 35.0, 34.3, 31.6, 30.9, 30.7, 23.5, 21.1, 15.3, 12.3, 11.2. HRMS (FT-ICR, HESI) *m/z*: $[M + H]^+$ for $C_{23}H_{40}NO_3$ 378.3003, found 378.2996.

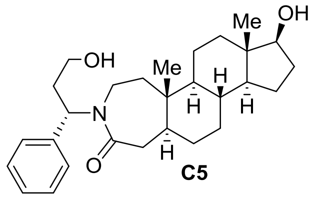

17β-Hydroxy-5α-androstane-derived A-Ring Lactam, C5

Following the general procedure A, (*S*)-3-azido-3-phenylpropanol (*S*)-**9** (44.1 mg, 0.249 mmol, 2.0 equiv) was reacted with 5α-DHT **2** (36.4 mg, 0.125 mmol) to give **C5** as a cream-colored amorphous solid (48.6 mg, 0.111 mmol, 89% yield, UPLC/HRMS purity: ≥99.5%). Purification was carried out by

an automated MPLC system using a 12 g normal phase silica column with gradient elution from 0–5% MeOH/CH_2Cl_2. R_f = 0.40 (5% MeOH/CH_2Cl_2); mp 197–202 ˚C; IR (neat) 3441, 3356, 1597 cm^{-1}; ^1H NMR (400 MHz, $CDCl_3$) δ 7.38–7.28 (m, 5H), 5.89 (dd, *J* = 12.4, 3.2 Hz, 1H), 3.72 (ddd, *J* = 12.1, 5.1, 2.5 Hz, 1H), 3.57 (t, *J* = 8.4 Hz, 1H), 3.40 (td, *J* = 11.7, 2.8 Hz, 1H), 3.20 (dd, *J* = 15.4, 11.7, 1H), 2.87–2.80 (m, 2H), 2.14–1.98 (m, 3H, contains d, 2.09, *J* = 14.8 Hz, 1H), 1.93 (m, 1H), 1.71–1.63 (m, 2H), 1.56–1.04 (complex, 10H), 0.97–0.80 (complex, 6H, contains s, 0.80, 3H), 0.67 (s, 3H), 0.49 (m, 1H), 0.40 (m, 1H); ^{13}C NMR (101 MHz, $CDCl_3$) δ 177.5, 139.1, 128.7 (2C), 128.6 (2C), 128.0, 81.9, 58.5, 54.3, 52.6, 50.9, 43.6, 42.8, 40.7, 40.4, 39.2, 38.3, 36.7, 34.9, 32.0, 31.6, 30.9, 30.7, 23.4, 20.8, 12.1, 11.1. **Note**: APT, NOE (1D), HSQC and HMBC are included in the spectra section. HRMS (FT-ICR, HESI) *m/z*: [M + H]$^+$ calcd for $C_{28}H_{42}NO_3$ 440.3159, found 440.3164.

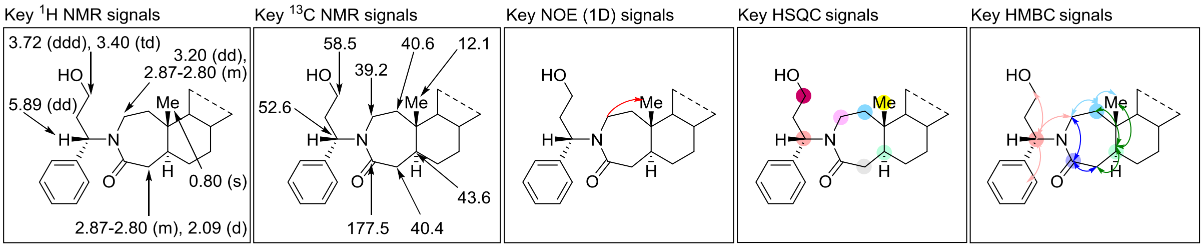

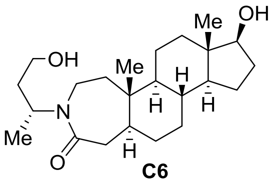

C6

17β-Hydroxy-5α-androstane-derived A-Ring Lactam, C6

Following the general procedure A, (*R*)-3-azidobutanol (*R*)-**10** (27.9 mg, 0.242 mmol, 2.0 equiv) was reacted with 5α-DHT **2** (36.0 mg, 0.124 mmol) to give **C6** as a white amorphous solid (39.7 mg, 0.105 mmol, 85% yield, UPLC/HRMS purity: 98.9%). Purification was carried out by an automated MPLC system using a 12 g normal phase silica column with gradient elution from 0–5% MeOH/CH_2Cl_2. R_f = 0.40 (5% MeOH/CH_2Cl_2); mp 179–184 ˚C; IR (neat) 3408, 3330, 1607 cm^{-1}; ^1H NMR (400 MHz, $CDCl_3$) δ 4.74 (m, 1H), 3.62 (t, *J* = 8.6 Hz, 1H), 3.52 (ddd, *J* = 12.0, 5.0, 2.5 Hz, 1H), 3.29–3.20 (m, 2H), 2.98 (m, 1H), 2.83 (dd, *J* = 15.0, 10.8 Hz, 1H), 2.10–2.01 (m, 2H, contains d, *J* = 15.0 Hz, 1H), 1.88–1.78 (m, 2H), 1.70–1.04 (complex, 17H, contains d, 1.18, *J* = 6.9 Hz, 1H), 0.96–0.83 (complex, 6H, contains s, 0.90, 3H), 0.75–0.68 (complex, 5H, contains s, 0.73, 3H); ^{13}C NMR (101 MHz, $CDCl_3$) δ 177.5, 81.9, 58.5, 54.5, 51.1, 45.5, 43.8, 42.9, 42.1, 40.4, 38.6, 38.0, 36.8, 36.6, 35.0, 31.7, 30.9, 30.7, 23.5, 21.0, 18.9, 12.3, 11.2. **Note**: APT, NOE (1D), HSQC and HMBC are included in the spectra section. HRMS (FT-ICR, HESI) *m/z*: [M + H]$^+$ calcd for $C_{23}H_{40}NO_3$ 378.3003, found 378.2984.

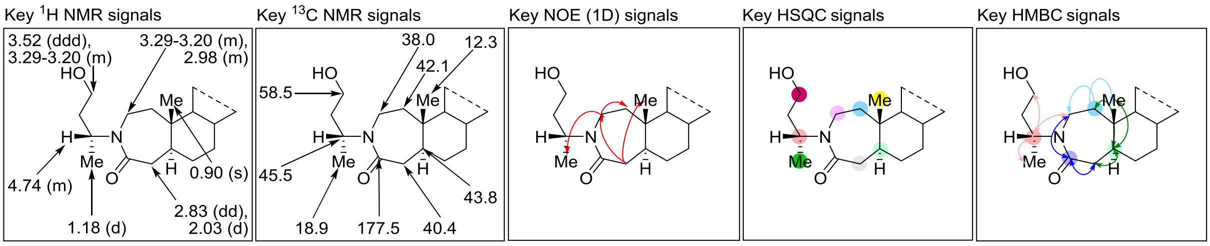

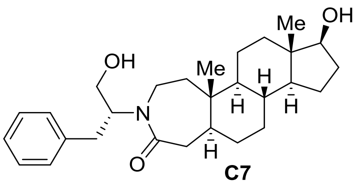

C7

17β-Hydroxy-5α-androstane-derived A-Ring Lactam, C7
Following the general procedure A, (*R*)-2-azido-3-phenylpropanol (*R*)-**14** (44.7 mg, 0.252 mmol, 2.0 equiv) was reacted with 5α-DHT **2** (35.9 mg, 0.124 mmol) to give **C7** as a white amorphous solid (35.1 mg, 0.080 mmol, 65% yield, LCMS purity: 97.3%). Purification was carried out by an automated MPLC system using a 12 g normal phase silica column with gradient elution from 0–4% MeOH/CH_2Cl_2 over 50 min. R_f = 0.32 (4% MeOH/CH_2Cl_2); IR (neat) 3375, 1619 cm^{-1}; mp 172–174 ˚C; ^1H NMR (400 MHz, $CDCl_3$) δ 7.32–7.26 (m, 2H), 7.24–7.19 (m, 3H), 3.78–3.68 (m, 2H), 3.61 (t, *J* = 8.5 Hz, 1H), 3.34 (dd, *J* = 15.9, 11.8 Hz, 1H), 3.02–2.85 (m, 3H), 2.81–2.75 (m, 1H), 2.09–1.99 (m, 4H), 1.77 (dt, *J* = 12.5, 3.4 Hz, 1H), 1.68–1.52 (m, 3H), 1.48–1.14 (complex, 7H), 1.02–0.79 (complex, 7H, contains s, 0.82, 3H), 0.70 (s, 3H), 0.53 (m, 1H); ^{13}C NMR (101 MHz, $CDCl_3$) δ 177.8, 138.5, 129.1 (2C), 128.7 (2C), 126.6, 81.9, 64.3, 54.4, 51.0, 43.7, 42.8, 41.3, 40.8, 38.4, 36.8, 35.0, 34.9, 31.6, 30.8, 30.7, 23.4, 20.9, 12.2, 11.2. **Note**: Missing two carbon signals due to signal overlap. HRMS (FT-ICR, HESI) *m/z*: $[M + H]^+$ calcd for $C_{28}H_{42}NO_3$ 440.3159, found 440.3151.

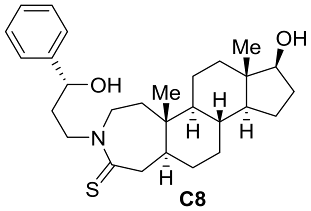

C8

17β-Hydroxy-5α-androstane-derived A-Ring Thioamide, C8
Following the general procedure B, (*R*)-3-azido-1-phenylpropanol (*R*)-**7** (72.1 mg, 0.407 mmol, 2.0 equiv) was reacted with 5α-DHT **2** (58.3 mg, 0.201 mmol) to give **C8** as an off-white solid (77.3 mg, 0.170 mmol, 85% yield, UPLC/HRMS purity: 98.9%). Purification was carried out by an automated MPLC system using a 12 g normal phase silica column with gradient elution from 0–45% EtOAc/hexanes. R_f = 0.38 (50% EtOAc/hexanes); mp 236–239 ˚C; IR (neat) 3331, 1529 cm^{-1}; ^1H NMR (400 MHz, $CDCl_3$) δ 7.39–7.33 (m, 4H), 7.29–7.27 (m, 1H), 4.73 (m, 1H), 4.68 (m, 1H), 3.91 (dd, *J* = 15.3, 11.9 Hz, 1H), 3.75 (m, 2H), 3.63 (t, *J* = 8.6 Hz, 1H), 3.36 (m, 1H), 3.10 (dd, *J* = 14.1, 9.7 Hz, 1H), 2.79 (d, *J* = 14.1 Hz, 1H), 2.21–1.78 (m, 3H), 1.88 (dd, *J* = 14.5, 6.4 Hz, 1H), 1.81 (dt, *J* = 12.4, 3.4 Hz, 1H), 1.69 (m, 1H), 1.63–1.17 (complex, 10 H), 1.05 (m, 1H), 0.99 (m, 4H, contains s, 0.91, 3H), 0.74–0.67 (m, 4H, contains s, 0.73, 3H); ^{13}C NMR (151 MHz, $CDCl_3$) δ 206.2, 143.9, 128.6 (2C), 127.6, 125.7 (2C), 81.9, 70.4, 54.6, 54.0, 51.0, 50.0, 49.8, 45.6, 42.9, 40.6, 38.4, 36.9, 36.8, 34.9, 31.6, 30.7, 23.4, 21.0, 12.6, 11.2. **Note**: Missing one carbon signal due to signal overlap. HRMS (FT-ICR, HESI) *m/z*: $[M + H]^+$ calcd for $C_{28}H_{42}NO_2S$ $[M + H]^+$ 456.2931, found 456.2921.

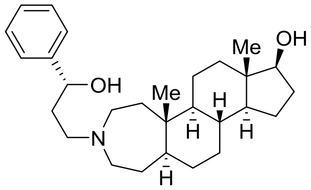

C9

17β-Hydroxy-5α-androstane-derived A-Ring Amine, C9 MC-006-179:
Method 1: Following the general procedure B, (*R*)-3-azido-1-phenylpropanol (*R*)-**7** (72.7 mg, 0.410 mmol, 2.0 equiv) was reacted with 5α-DHT **2** (58.0 mg, 0.200 mmol) to give **C9** as pale yellow sticky solid (73.6 mg, 0.173 mmol, 87% yield, UPLC/HRMS purity: ≥99.5%). Purification was carried out by

an automated MPLC system using a 12 g normal phase silica column using MeOH/CH_2Cl_2. R_f = 0.15 (4% MeOH/CH_2Cl_2). HRMS (FT-ICR, HESI) *m/z*: $[M + H]^+$ calcd for $C_{28}H_{44}NO_2$ $[M + H]^+$ 426.3367, found 426.3356.

Method 2: **C2** (110.0 mg, 0.250 mmol) was added to a stirring suspension of LAH (1M THF, 0.500 mL, 0.500 mmol, 2.0 equiv) in anhydrous THF (14 mL) at 0 ˚C. The reaction mixture was allowed to room temperature, stirred for 4 h and then refluxed for 24 h. The reaction mixture was allowed to room temperature and quenched with a saturated aqueous solution of sodium potassium tartarate (5 mL). The biphasic mixture was stirred overnight. The biphasic mixture was diluted CH_2Cl_2 (50 mL). The organic layer was washed with aqueous solution of sodium potassium tartarate (2 × 5 mL), brine (5 mL), dried over Na_2SO_4, filtered, and concentrated. Purification was carried out by an automated MPLC system using a 12 g normal phase silica column using MeOH/CH_2Cl_2 to give **C9** as white foam solid (53.9 mg, 0.127 mmol, 51% yield, UPLC/HRMS purity: ≥99.5%). mp decomposed; IR (neat) 3357, 2918, 2846, 1449 cm^{-1}; ^1H NMR (400 MHz, $CDCl_3$) δ 7.36 (m, 4H), 7.23 (m, 1H), 4.99 (dd, *J* = 7.9, 3.2 Hz, 1H), 3.61 (t, *J* = 8.6 Hz, 1H), 2.91 (m, 1H), 2.76 (m, 3H), 2.56 (m, 2H), 2.05 (m, 1H), 1.90 (m, 1H) 1.79 (m, 2H), 1.66–1.51 (complex, 4H), 1.03 (m, 1H), 0.90 (m, 2H), 0.83 (s, 3H), 0.76–0.73 (complex, 4H, contains s, 0.73, 3H); ^{13}C NMR (151 MHz, $CDCl_3$) δ 145.2, 128.3 (2C), 127.0, 125.7 (2C), 82.1, 75.5, 58.3, 54.7, 54.0, 51.3, 50.7, 46.2, 42.9, 38.5, 37.0, 35.3, 34.1, 31.7, 31.0, 30.7, 29.9, 23.6, 21.5, 13.3, 11.3. **Note**: Missing one carbon signal due to signal overlap. HRMS (FT-ICR, HESI) *m/z*: $[M + H]^+$ calcd for $C_{28}H_{44}NO_2$ $[M + H]^+$ 426.3367, found 426.3357.

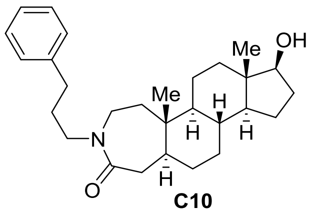

C10

17β-Hydroxy-5α-androstane-derived A-Ring Lactam C10

MC-006-180: Following the general procedure B, (*R*)-3-azido-1-phenylpropanol (*R*)-**7** (70.5 mg, 0.398 mmol, 1.9 equiv) was reacted with 5α-DHT **2** (60.0 mg, 0.207 mmol) to give **C10** as white foam solid (80.9 mg, 0.191 mmol, 92% yield, UPLC/HRMS purity: ≥99.5%). Purification was carried out by an automated MPLC system using a 12 g normal phase silica column with gradient elution from 0–3% MeOH/CH_2Cl_2. R_f = 0.38 (4% MeOH/CH_2Cl_2); mp decomposed; IR (neat) 3214, 1626 cm^{-1}; ^1H NMR (400 MHz, $CDCl_3$) δ 7.29 (m, 2H), 7.18 (m, 3H), 3.59 (m, 2H), 3.48–3.31 (m, 2H), 2.95 (dd, *J* = 15.5, 6.3 Hz, 1H), 2.74 (dd, *J* = 14.3, 10.1 Hz, 1H), 2.62 (td, *J* = 7.4, 2.5 Hz, 2H), 2.04 (m, 1H), 1.93 (d, *J* = 14.3 Hz, 1H), 1.96–1.77 (m, 4H), 1.68–1.56 (complex, 4H), 1.48–1.14 (complex, 6H), 1.03 (m, 1H), 0.95–0.83 (complex, 6H, contains s, 0.88, 3H), 0.73–0.66 (complex, 4H, contains s, 0.73, 3H); ^{13}C NMR (101 MHz, $CDCl_3$) δ 175.4, 141.9, 128.52 (2C), 128.46 (2C), 126.0, 82.0, 54.5, 51.1, 47.9, 44.9, 43.9, 42.9, 41.6, 40.5, 38.5, 36.8, 35.0, 33.4, 31.7, 30.9, 30.7, 30.1, 23.5, 21.0, 12.2, 11.2. HRMS (FT-ICR, HESI) *m/z*: $[M + H]^+$ calcd for $C_{28}H_{42}NO_2$ $[M + H]^+$ 424.3210, found 424.3203.

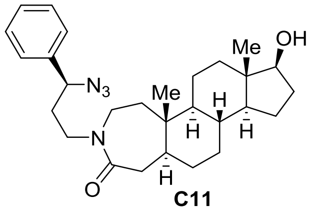

C11

17β-Hydroxy-5α-androstane-derived A-Ring Lactam, C11

Following the general procedure B, (*R*)-3-azido-1-phenylpropanol (*R*)-**7** (71.6 mg, 0.404 mmol, 2.0 equiv) was reacted with 5α-DHT **2** (57.7 mg, 0.199 mmol) to give **C11** as a yellow amorphous solid (52.0 mg, 0.112 mmol, 56% yield, UPLC/HRMS purity: 97.9%). Purification was carried out by an automated MPLC system using a 12 g normal phase silica column with gradient elution from 0–50%

EtOAc/hexanes. R_f = 0.32 (50% EtOAc/hexanes); mp 160–163 ˚C; IR (neat) 3378, 2093, 1624 cm^{-1}; ^{1}H NMR (400 MHz, $CDCl_3$) δ 7.40–7.30 (m, 5H), 4.46 (t, *J* = 7.2 Hz, 1H), 3.62 (t, *J* = 8.5 Hz, 1H), 3.56–3.45 (m, 2H), 3.32 (m ,1H), 2.92 (m, 1H), 2.67 (dd, *J* = 14.3, 10.3 Hz, 1H), 2.05 (m, 1H), 1.97 (q, *J* = 7.3 Hz, 2H), 1.91 (d, *J* = 8.96 Hz, 1H), 1.82–1.77 (m, 2H), 1.69–1.63 (m, 1H), 1.62–1.16 (complex, 10H), 1.05 (m, 1H), 0.96–0.83 (m, 5H, contains s, 3H), 0.74–0.68 (m, 4H, contains s, 3H); ^{13}C NMR (101 MHz, $CDCl_3$) δ 175.6, 139.3, 129.0 (2C), 128.6, 127.0 (2C), 82.0, 64.4, 54.4, 51.1, 45.7, 45.2, 43.8, 42.9, 41.3, 40.3, 38.5, 36.8, 35.0, 34.7, 31.7, 30.9, 30.7, 23.5, 21.0, 12.2, 11.2. HRMS (FT-ICR, HESI) *m/z*: $[M + H]^+$ calcd for $C_{28}H_{41}N_4O_2$ 465.3224, found 465.3212.

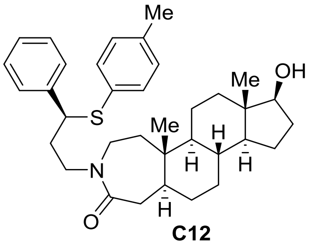

C12

17β-Hydroxy-5α-androstane-derived A-Ring Lactam, C12

Following the general procedure B, (*R*)-3-azido-1-phenylpropanol (*R*)-**7** (44.5 mg, 0.250 mmol, 2.0 equiv) was reacted with 5α-DHT **2** (36.3 mg, 0.125 mmol) to give **C12** as a yellow crystalline solid (45.2 mg, 0.0828 mmol, 66% yield, UPLC/HRMS purity: 97.0%). Purification was carried out by an automated MPLC system using a 12 g normal phase silica column with gradient elution from 0–60% EtOAc/hexanes. R_f = 0.30 (50% EtOAc/hexanes); mp 182–186 ˚C; IR (neat) 3395, 1632 cm^{-1}; ^{1}H NMR (400 MHz, $CDCl_3$) δ 7.27–7.17 (m, 5H), 7.12 (m, 2H), 6.99 (m, 2H), 4.05 (t, *J* = 7.4 Hz, 1H), 3.62 (t, *J* = 8.6 Hz, 1H), 3.44–3.28 (m, 3H), 2.83 (m, 1H), 2.58 (dd, *J* = 14.2, 10.4 Hz, 1H), 2.28 (s, 3H), 2.13 (q, *J* = 7.3 Hz, 2H), 2.04 (m, 1H), 1.85 (d, *J* = 14.3 Hz, 1H), 1.82–1.71 (m, 2H), 1.67–0.98 (complex, 11H), 0.94–0.80 (m, 5H, contains s, 0.83, 3H), 0.72 (s, 3H), 0.69–0.62 (m, 1H); ^{13}C NMR (126 MHz, $CDCl_3$) δ 175.4, 141.8, 137.6, 133.3 (2C), 130.8, 129.6 (2C), 128.5 (2C), 127.9 (2C), 127.3, 82.0, 54.3, 51.8, 51.0, 46.6, 45.0, 43.8, 42.8, 41.4, 40.4, 38.4, 36.8, 34.9, 34.3, 31.6, 30.9, 30.7, 23.4, 21.3, 21.0, 12.1, 11.2. HRMS (FT-ICR, HESI) *m/z*: $[M + H]^+$ calcd for $C_{35}H_{48}NO_2S$ $[M + H]^+$ 546.3400, found 546.3420.

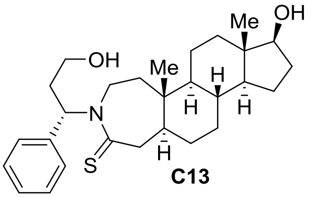

C13

17β-Hydroxy-5α-androstane-derived A-Ring Thioamide, C13

Following the general procedure B, (*S*)-3-azido-3-phenylpropanol (*S*)-**9** (74.0 mg, 0.418 mmol, 2.1 equiv) was reacted with 5α-DHT **2** (58.8 mg, 0.202 mmol) to give **C13** as an off-white amorphous solid (73.6 mg, 0.162 mmol, 80% yield, UPLC/HRMS purity: 90.3%). Purification was carried out by an automated MPLC system using a using 12 g normal phase silica column with gradient elution from 0–50% EtOAc/hexanes. R_f = 0.32 (50% EtOAc/hexanes); mp 249–251 ˚C; IR (neat) 3338, 1488, 1454 cm^{-1}; ^{1}H NMR (400 MHz, $CDCl_3$) δ 7.41–7.31 (m, 5H), 7.12 (dd, *J* = 12.0, 3.3 Hz, 1H), 3.79–3.71 (m, 1H), 3.57 (m, 1H), 3.52–3.44 (m, 3H), 3.25–3.12 (m, 2H), 2.96 (d, *J* = 14.6 Hz, 1H), 2.24–2.16 (m, 1H), 2.12–1.99 (m, 2H), 1.69–1.63 (m, 2H), 1.60–1.52 (m, 2H), 1.44–1.02 (complex, 9H), 0.96–0.81 (m, 3H), 0.81 (s, 3H), 0.66 (s, 3H), 0.41 (m, 1H), 0.21 (m, 1H); ^{13}C NMR (151 MHz, $CDCl_3$) δ 207.0, 138.0, 128.9 (2C), 128.6, 128.3 (2C), 81.9, 61.0, 58.1, 54.2, 50.8, 49.9, 44.9, 43.7, 42.8, 39.7, 38.0, 36.6, 34.8, 32.3, 31.5, 30.8, 30.7, 23.4, 20.7, 12.5, 11.1. HRMS (FT-ICR, HESI) *m/z*: $[M + H]^+$ calcd for $C_{28}H_{42}NO_2S$ $[M + H]^+$ 456.2931, found 456.2929.

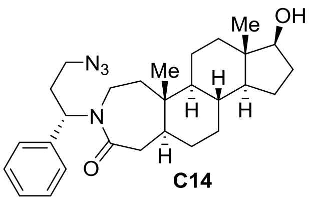

C14

17β-Hydroxy-5α-androstane-derived A-Ring Lactam, C14

Following the general procedure B, (*S*)-3-azido-3-phenylpropanol (*S*)-**9** (71.1 mg, 0.401 mmol, 2.0 equiv) was reacted with 5α-DHT **2** (57.0 mg, 0.196 mmol) to give **C14** as a pale yellow crystalline solid (54.6 mg, 0.118 mmol, 60% yield, LCMS purity: ≥99.5%). Purification was carried out by an automated MPLC system using a 12 g normal phase silica column with gradient elution from 0–50% EtOAc/hexanes. R_f = 0.32 (50% EtOAc/hexanes); mp decomposed; IR (neat) 3394, 2096, 1624 cm^{-1}; ^{1}H NMR (400 MHz, $CDCl_3$) δ 7.38–7.28 (m, 5H), 5.88 (dd, *J* = 8.9, 6.6 Hz, 1H), 3.57 (t, *J* = 8.6 Hz, 1H), 3.42–3.25 (m, 3H), 2.96 (m, 1H), 2.78 (m, 1H), 2.63 (m, 1H), 2.20–2.12 (m, 2H), 2.09–1.98 (m, 2H), 1.71–1.05 (complex, 11H), 0.96–0.80 (complex, 6H, contains s, 0.81, 3H), 0.67 (s, 3H), 0.44 (m, 1H), 0.30 (m, 1H); ^{13}C NMR (101 MHz, $CDCl_3$) δ 175.9, 139.1, 128.9 (2C), 128.1 (2C), 128.0, 82.0, 54.2, 53.4, 50.9, 49.1, 43.6, 42.8, 40.9, 40.5, 39.1, 38.2, 36.7, 34.9, 31.5, 30.9, 30.7, 29.4, 23.4, 20.8, 12.1, 11.2. HRMS (FT-ICR, HESI) *m/z*: $[M + H]^+$ calcd for $C_{28}H_{41}N_4O_2$ 465.3224, found 465.3215.

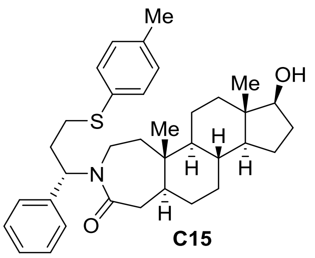

C15

17β-Hydroxy-5α-androstane-derived A-Ring Lactam, C15

Following the general procedure B, (*S*)-3-azido-3-phenylpropanol (*S*)-**9** (44.2 mg, 0.249 mmol, 2.0 equiv) was reacted with 5α-DHT **2** (36.3 mg, 0.125 mmol) to give **C15** as a white amorphous solid (44.3 mg, 0.081 mmol, 65% yield, UPLC/HRMS purity: 97.2%). Purification was carried out by an automated MPLC system using a using 12 g normal phase silica column with gradient elution from 0–40% EtOAc/hexanes. R_f = 0.53 (50% EtOAc/hexanes); mp 181–183 ˚C; IR (neat) 3313, 1613 cm^{-1}; ^{1}H NMR (400 MHz, $CDCl_3$) δ 7.34–7.24 (m, 7H), 7.10–7.08 (m, 2H), 5.87 (dd, *J* = 9.6, 5.9 Hz, 1H), 3.56 (t, *J* = 8.6 Hz, 1H), 3.23 (dd, *J* = 15.6, 11.5 Hz, 1H), 2.96–2.87 (m, 2H), 2.81–2.73 (m, 2H), 2.31 (s, 3H), 2.24–2.10 (m, 2H), 2.07–1.98 (m, 2H, contains d, 2.04, *J* = 14.3 Hz, 1H), 1.70–1.04 (complex, 13H), 0.95–0.76 (m, 5H, contains s, 0.79, 3H), 0.67 (s, 3H), 0.41 (m, 1H), 0.27 (m, 1H); ^{13}C NMR (101 MHz, $CDCl_3$) δ 175.7, 139.6, 136.5, 132.5, 130.5 (2C), 129.8 (2C), 128.6 (2C), 128.2 (2C), 127.8, 81.9, 55.0, 54.2, 50.9, 43.6, 42.8, 40.9, 40.6, 38.9, 38.2, 36.7, 34.9, 31.58, 31.55, 30.9, 30.7, 30.2, 23.4, 21.1, 20.8, 12.1, 11.1. HRMS (FT-ICR, HESI) *m/z*: $[M + H]^+$ calcd for $C_{35}H_{48}NO_2S$ $[M + H]^+$ 546.3400, found 546.3396.

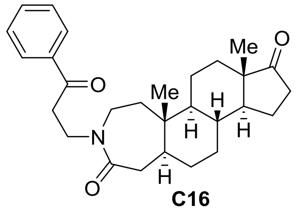

C16

17β-Hydroxy-5α-androstane-derived A-Ring Lactam, C16

Following the general procedure C, **C2** (75.0 mg, 0.170 mmol) was oxidized to give **C16** as a white amorphous solid (50 mg, 0.115 mmol, 68% yield, UPLC/HRMS purity: ≥99.5%). Purification was

carried out by an automated MPLC system using a 12 g normal phase silica column with gradient elution from 0–2% MeOH/CH_2Cl_2. Concentration of solvents afforded a slightly impure azasteroid, which was subjected to a second purification on a 4 g normal phase silica column with gradient elution 0–80% EtOAc/hexanes. R_f = 0.20 (50% EtOAc/hexanes); mp 209–212 ˚C; IR (neat) 1726, 1673, 1645, 1628 cm^{-1}; ^{1}H NMR (400 MHz, $CDCl_3$) δ 7.97 (m, 2H), 7.56 (m, 1H), 7.46 (m, 2H), 3.82–3.62 (m, 3H), 3.31–3.20 (m, 3H), 2.75 (dd, *J* = 14.3, 9.9 Hz, 1H), 2.43 (m, 1H), 2.06 (m, 1H), 1.96–1.89 (m, 2H, contains d, *J* = 14.4 Hz, 1H), 1.82–1.75 (m, 3H), 1.68–1.63 (m, 1H), 1.53–1.14 (complex, 9H), 1.04–0.91 (m, 1H), 0.89 (s, 3H), 0.84 (s, 3H), 0.71 (m, 1H); ^{13}C NMR (101 MHz, $CDCl_3$) δ 221.1, 199.3, 175.7, 136.8, 133.5, 128.8 (2C), 128.3 (2C), 54.3, 51.4, 47.6, 46.6, 45.3, 43.7, 41.4, 40.3, 38.6, 37.8, 36.0 34.5, 31.6, 30.9, 30.7, 21.8, 20.7, 13.9, 12.2. HRMS (FT-ICR, HESI) *m/z*: $[M + H]^+$ calcd for $C_{28}H_{38}NO_3$ $[M + H]^+$ 436.2846, found 436.2823.

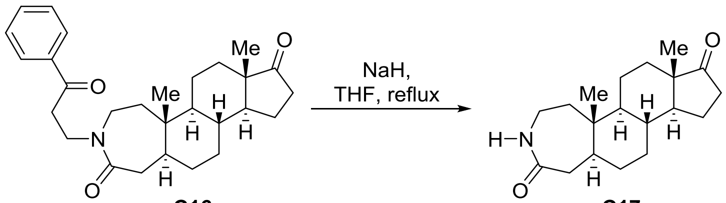

17β-Hydroxy-5α-androstane-derived A-Ring Lactam, C17

A solution of **C16** (87.0 mg, 0.200 mmol) and sodium hydride (60% dispersion in mineral oil, 38.0 mg, 1.60 mmol, 4.8 equiv) in anhydrous THF (8.0 mL) was heated at 65 ˚C for 2 h. The reaction was cooled to room temperature and quenched with a saturated solution of NH_4Cl (5 mL). The reaction mixture was diluted with CH_2Cl_2 (20 mL) and washed with saturated solution of NH_4Cl (2 x 5 mL), H_2O (5 mL) and brine (5 mL). The combined organic layer was dried over anhydrous Na_2SO_4 filtered, and concentrated. The crude residue was purified by an automated MPLC system using a 12 g normal phase silica column with gradient elution from 0–5% MeOH/CH_2Cl_2. Concentration of solvents afforded product **C17** as white amorphous solid (44.0 mg, 0.145 mmol, 73% yield, UPLC/HRMS purity: ≥99.5%). R_f = 0.25 (3% MeOH/CH_2Cl_2); mp 283–285 ˚C; IR (neat) 3303, 3219, 1736, 1668 cm^{-1}. ^{1}H NMR (400 MHz, $CDCl_3$) δ 5.77 (br s, 1H), 3.40 (ddd, *J* = 15.9, 11.8, 4.4 Hz, 1H), 3.00 (m, 1H), 2.77 (dd, *J* = 14.3, 11.0 Hz, 1H), 2.44 (dd, *J* = 19.4, 8.8 Hz, 1H), 2.08 (m, 1H), 1.97–1.78 (complex, 4H), 1.70 (m, 1H), 1.60–1.22 (complex, 10H), 1.03 (m, 1H), 0.94 (s, 3H), 0.89–0.78 (complex, 5H, contains s, 0.86, 3H); ^{13}C NMR (151 MHz, $CDCl_3$) δ 221.0, 178.3, 54.3, 51.5, 47.6, 43.4, 42.2, 39.7, 39.2, 38.0, 36.0, 34.4, 31.7, 31.0, 30.8, 21.9, 20.7, 13.9, 12.1. HRMS (FT-ICR, HESI) *m/z*: $[M + H]^+$ calcd for $C_{19}H_{20}NO_2$ 304.2271, found 304.2267.

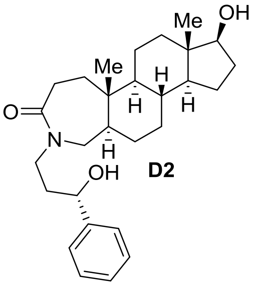

D2

17β-Hydroxy-5α-androstane-derived A-Ring Lactam, D2

Following the general procedure A, (*S*)-3-azido-1-phenylpropanol (*S*)-**7** (44.3 mg, 0.250 mmol, 2.0 equiv) was reacted with 5α-DHT **2** (36.1 mg, 0.124 mmol) to give **D2** as a cream-colored amorphous (48.1 mg, 0.109 mmol, 88% yield, UPLC/HRMS purity: ≥99.5%). Purification was carried out by an automated MPLC system using a 12 g normal phase silica column with gradient elution from 0–5% MeOH/CH_2Cl_2. R_f = 0.35 (5% MeOH/CH_2Cl_2); mp 155–157 ˚C; IR (neat) 3321, 1622 cm^{-1}; ^{1}H NMR (400 MHz, $CDCl_3$) δ 7.37–7.32 (m, 4H), 7.26 (m, 1H), 4.63 (dd, *J* = 9.8, 3.2 Hz, 1H), 4.09 (ddd, *J* = 14.6, 10.6, 4.3 Hz, 1H), 3.69 (m, 1H), 3.64 (t, *J* = 8.6 Hz, 1H), 3.11 (dt, *J* = 14.2, 4.5 Hz, 1H), 2.61–

2.52 (m, 2H, contains d, 2.54, J = 15.6 Hz, 1H), 2.11–2.02 (m, 1H), 1.95–1.72 (complex, 5H), 1.68–1.55 (complex, 2H), 1.48–1.19 (complex, 9H), 1.06 (m, 1H), 0.98–0.84 (complex, 5H, contains s, 0.92, 3H), 0.75–0.68 (complex, 4H, contains s, 0.74, 3H); ^{13}C NMR (101 MHz, $CDCl_3$) δ 177.1, 144.3, 128.5 (2C), 127.2, 125.6 (2C), 81.9, 70.0, 54.4, 52.4, 51.1, 49.6, 45.9, 42.9, 38.5, 38.0, 36.7, 36.2, 35.2, 32.1, 31.8, 30.6, 28.4, 23.5, 20.7, 12.2, 11.3. **Note**: APT, NOE (1D), HSQC and HMBC are included in the spectra section. HRMS (FT-ICR, HESI) *m/z*: $[M + H]^+$ calcd for $C_{28}H_{42}NO_3$ 440.3159, found 440.3167.

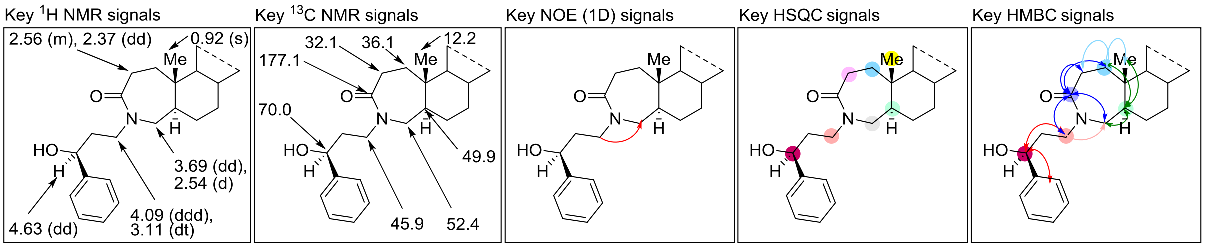

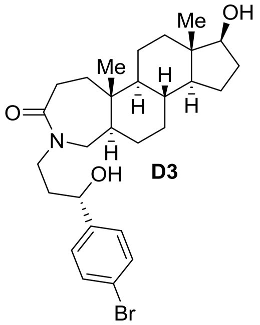

D3

17β-Hydroxy-5α-androstane-derived A-Ring Lactam, D3

Following the general procedure A, (S)-3-azido-1-(4-bromophenyl)propanol (S)-**11** (65.8 mg, 0.257 mmol, 2.0 equiv) was reacted with 5α-DHT **2** (35.9 mg. 0.124 mmol) to give **D3** as a white amorphous solid (54.9 mg, 0.106 mol, 86% yield, UPLC/HRMS purity: ≥99.5%). Purification was carried out by an automated MPLC system using a 12 g normal phase silica column with gradient elution from 0–2% MeOH/CH_2Cl_2. R_f = 0.38 (4% MeOH/CH_2Cl_2); mp 195–199 ˚C; IR (neat) 3334, 1615, 1598 cm^{-1}; ^{1}H NMR (400 MHz, $CDCl_3$) δ 7.47 (d, J = 8.4 Hz, 2H), 7.23 (d, J = 8.3 Hz, 2H), 4.58 (dd, J = 9.9, 3.1 Hz, 1H), 4.10 (dd, J = 14.6, 10.9, 3.9 Hz, 1H), 3.71 (m, 1H), 3.64 (t, J = 8.6 Hz, 1H), 3.08 (dt, J = 14.3, 4.4 Hz, 1H), 2.62–2.52 (m, 2H), 2.38 (m, 1H), 2.07 (m, 1H), 2.00–1.55 (complex, 6H), 1.49–1.20 (complex, 9H), 1.06 (m, 1H), 0.99–0.83 (m, 5H, contains s, 0.91, 3H), 0.74–0.67 (m, 4H, contains s, 0.74, 3H); ^{13}C NMR (101 MHz, $CDCl_3$) δ 177.2, 143.3, 131.5 (2C), 127.4 (2C), 120.9, 81.9, 69.4, 54.5, 52.5, 51.1, 49.8, 45.8, 42.9, 38.5, 38.0, 36.7, 36.2, 35.2, 32.1, 31.8, 30.6, 28.4, 23.5, 20.8, 12.2, 11.3. HRMS (FT-ICR, HESI) *m/z*: $[M + H]^+$ calcd for $C_{28}H_{41}NO_3$ 518.2264, found 518.2254.

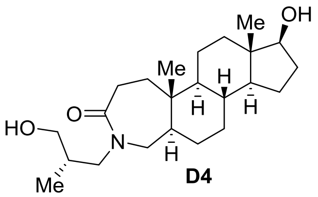

D4

17β-Hydroxy-5α-androstane-derived A-Ring Lactam, D4

Following the general procedure A, (S)-3-azido-2-methylpropanol (S)-**8** (27.4 mg, 0.238 mmol, 2.0 equiv) 5α-DHT **2** (36.2 mg, 0.125 mmol) was reacted with to give **D4** as an off-white amorphous solid (41.0 mg, 0.109 mol, 87% yield, UPLC/HRMS purity: ≥99.5%). Purification was carried out by an automated MPLC system using a 12 g normal phase silica column with gradient elution from 50–100% EtOAc/hexanes. R_f = 0.28 (4% MeOH/CH_2Cl_2); mp 196–200 ˚C; IR (neat) 3312, 1607 cm^{-1}; ^{1}H NMR (400 MHz, $CDCl_3$) δ 3.71 (dd, J = 14.0, 10.1, 1H), 3.65–3.58 (m, 2H), 3.43 (dd, J = 11.8, 3.1 Hz, 1H), 3.30 (dd, J = 11.8, 3.8 Hz, 1H), 2.86 (dd, J = 13.9, 4.4 Hz, 1H), 2.67 (t, J = 13.9 Hz, 1H), 2.50 (d, J =

15.3 Hz, 1H), 2.37 (dd, *J* = 14.8, 7.5 Hz, 1H), 2.05 (m, 1H), 1.88 (m, 1H), 1.81 (m, 1H), 1.75–1.18 (complex, 13H), 1.04 (m, 1H), 0.97 (d, *J* = 6.9 Hz, 3H), 0.85–0.86 (m, 4H, contains s, 0.91, 3H), 0.76–0.70 (m, 4H, contains s, 0.73, 3H); ^{13}C NMR (126 MHz, $CDCl_3$) δ 177.2, 81.9, 63.3, 54.2, 52.3, 51.1, 50.7, 48.4, 42.9, 38.5, 36.8, 35.9, 35.2, 34.3, 32.1, 31.5, 30.6, 28.5, 23.5, 20.7, 15.2, 12.4, 11.3. HRMS (FT-ICR, HESI) *m/z*: $[M + H]^+$ calcd for $C_{23}H_{40}NO_3$ 378.3003, found 378.2996.

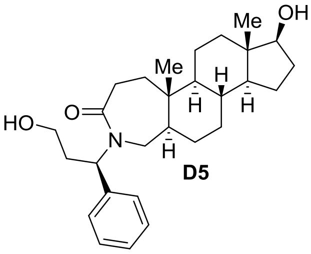

D5

17β-Hydroxy-5α-androstane-derived A-Ring Lactam, D5

Following the general procedure A, (*R*)-3-azido-3-phenylpropanol (*R*)-**9** (44.4 mg, 0.251 mmol, 2.0 equiv) was reacted with 5α-DHT **2** (36.3 mg, 0.125 mmol) to give **D5** as a cream-colored amorphous solid (49.5 mg, 0.113 mmol, 90% yield, UPLC/HRMS purity: ≥99.5%). Purification was carried by an automated MPLC system using a 12 g normal phase silica column with gradient elution from 0–4% MeOH/CH_2Cl_2. R_f = 0.40 (5% MeOH/CH_2Cl_2); mp 248–254 ˚C; IR (neat) 3344, 1613 cm^{-1}; ^{1}H NMR (400 MHz, $CDCl_3$) δ 7.37–7.28 (m, 5H), 5.92 (ddd, *J* = 12.0, 5.1, 2.5 Hz, 1H), 3.57 (t, *J* = 8.5 Hz, 1H), 3.42 (td, *J* = 11.7, 2.9 Hz, 1H), 3.19 (dd, *J* = 15.3, 9.0 Hz, 1H), 2.69 (m, 1H), 2.49 (m, 1H), 2.37 (d, *J* = 15.4 Hz, 1H), 2.12–1.84 (complex, 4H), 1.76 (m, 1H), 1.59–1.08 (complex, 8H), 1.03–0.74 (m, 7H, contains s, 0.78, 3H), 0.66 (s, 3H) 0.52–0.44 (m, 2H), 0.37–0.22 (m, 2H); ^{13}C NMR (101 MHz, $CDCl_3$) δ 177.2, 139.0, 129.0 (2C), 128.5 (2C), 128.1, 81.9, 58.5, 54.1, 52.8, 50.9, 48.8, 45.4, 42.8, 38.1, 36.7, 36.1, 35.1, 32.5, 32.2, 31.2, 30.6, 27.2, 23.4, 20.6, 12.1, 11.2. **Note**: APT, NOE (1D), HSQC and HMBC are included in the spectra section. HRMS (FT-ICR, HESI) *m/z*: $[M + H]^+$ calcd for $C_{28}H_{42}NO_3$ 440.3159, found 440.3162.

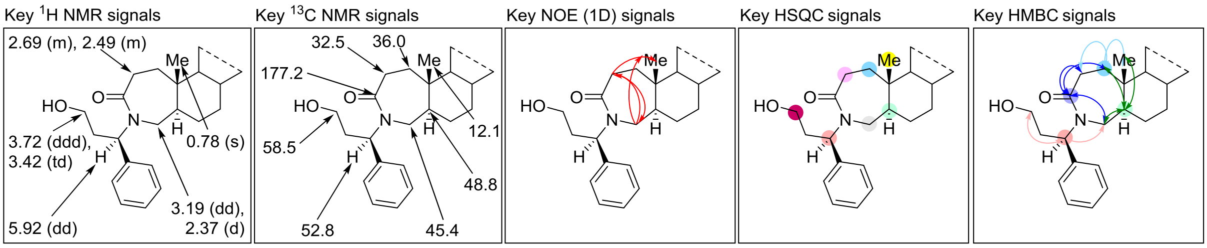

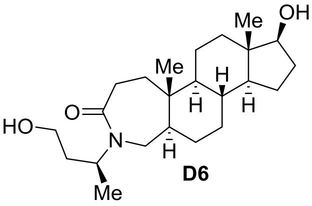

D6

17β-Hydroxy-5α-androstane-derived A-Ring Lactam, D6

Following the general procedure A, (*S*)-3-azidobutanol (*S*)-**10** (29.5 mg, 0.256 mmol, 2.0 equiv) was reacted with 5α-DHT **2** (36.5 mg, 0.126 mmol) to give **D6** as a white amorphous solid (40.3 mg, 0.107 mmol, 85% yield, UPLC/HRMS purity: 96.3%). Purification was carried out by an automated MPLC system using a 12 g normal phase silica column with gradient elution from 0–5% MeOH/CH_2Cl_2. R_f = 0.20 (80% EtOAc/hexanes); mp 217–219 ˚C; IR (neat) 3357, 1620 cm^{-1}; ^{1}H NMR (400 MHz, $CDCl_3$) δ 4.78 (m, 1H), 3.63 (t, *J* = 8.7 Hz, 1H), 3.51 (m, 1H), 3.29–3.22 (m, 2H), 2.69 (t, *J* = 14.2 Hz, 1H), 2.51–2.42 (m, 2 H, contains d, 2.49, *J* = 15.3 Hz, 1H), 2.49 (m, 1H), 1.89 (dd, *J* = 14.1, 7.5 Hz, 1H), 1.81 (dt, *J* = 12.3, 3.4 Hz, 1H), 1.75–1.53 (m, 4H), 1.48–1.12 (complex, 13H, contains d, 1.17, *J* = 8.1 Hz, 3H), 1.04 (m, 1H), 0.97–0.84 (m, 5H, contains s, 0.91, 3H), 0.75–0.68 (m, 4H, contains s, 0.73,

3H); ^{13}C NMR (101 MHz, $CDCl_3$) δ 177.4, 81.9, 58.6, 54.2, 51.1, 50.0, 45.6, 44.4, 42.9, 38.5, 36.7, 36.0, 35.5, 32.5, 31.6, 30.6, 27.5, 23.5, 20.7, 18.9, 12.3, 11.3. **Note**: Missing one carbon signal due to signal overlap. APT, NOE (1D), HSQC and HMBC are included in the spectra section. HRMS (FT-ICR, HESI) *m/z*: $[M + H]^+$ calcd for $C_{23}H_{40}NO_3$ 378.3003, found 378.2989.

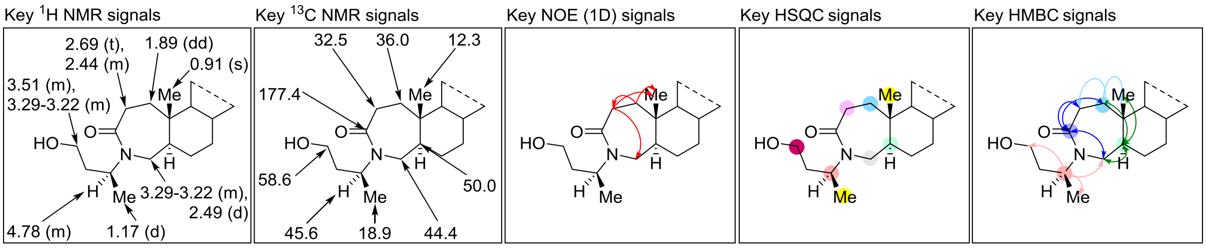

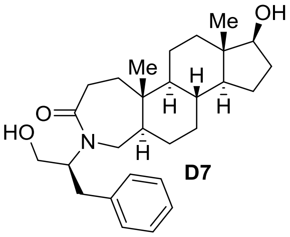

D7

17β-Hydroxy-5α-androstane-derived A-Ring Lactam, D7

Following the general procedure A, (*S*)-2-azido-3-phenylpropanol (*S*)-**14** (45.8 mg, 0.458 mmol, 2.0 equiv) was reacted with 5α-DHT **2** (36.5 mg, 0.126 mmol) to give **D7** as a white amorphous solid (42.0 mg, 0.096 mmol, 76% yield, LCMS purity: 96.0%) containing a slight impurity of minor regioisomer. Purification was carried out by an automated MPLC system using a 12 g normal phase silica column with gradient elution from 0–4% MeOH/CH_2Cl_2. R_f = 0.28 (4% MeOH/CH_2Cl_2); mp 157–159 ˚C; IR (neat) 3390, 1621 cm^{-1}; ^{1}H NMR (400 MHz, $CDCl_3$) δ 7.32–7.27 (m, 2H), 7.24–7.19 (m, 2H), 4.68 (br s, 1H), 3.74 (dd, *J* = 11.6, 3.6 Hz, 1H), 3.67–3.58 (m, 2H), 3.39 (dd, *J* = 15.6, 9.3 Hz, 1H), 2.992.82 (m, 2H), 2.66 (t, *J* = 13.9 Hz, 1H), 2.49–2.39 (m, 2H), 2.26 (m, 2H), 2.09–1.99 (m, 1H), 1.82–1.74 (m, 2H), 1.64–1.50 (m, 3H), 1.46–1.36 (m, 1H), 1.32–0.95 (complex, 7H), 0.89–0.76 (m, 4H, contains s, 0.81, 3H), 0.76–0.65 (m, 4H, contains s, 0.69, 3H), 0.42 (m, 1H); ^{13}C NMR (151 MHz, $CDCl_3$) δ 178.0, 138.0, 128.8, 128.7, 126.7, 81.9, 64.1, 54.0, 51.0, 48.4, 42.9, 38.1, 36.7, 35.7, 35.0, 34.9, 32.6, 31.3, 30.6, 28.1, 23.4, 20.6, 12.2, 11.2. **Note**: Missing carbon two carbon signals due to signal overlap. HRMS (FT-ICR, HESI) *m/z*: $[M + H]^+$ calcd for $C_{28}H_{42}NO_3$ 440.3159, found 440.3154.

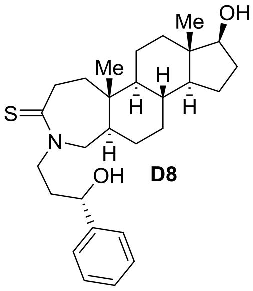

D8

17β-Hydroxy-5α-androstane-derived A-Ring Thioamide, D8

Following the general procedure B, (*S*)-3-azido-1-phenylpropanol (*S*)-**7** (71.2 mg, 0.402 mmol, 2.0 equiv) was reacted with 5α-DHT **2** (58.6 mg, 0.202 mmol) to give **D8** as a white amorphous solid (41.2 mg, 0.090 mmol, 45% yield, UPLC/HRMS purity: 97.6%). Purification was carried out by an automated MPLC system using a using 12 g normal phase silica column with gradient elution from 0–40% EtOAc/hexanes. R_f = 0.42 (50% EtOAc/hexanes); mp 229–233 ˚C; IR (neat) 3428, 3302, 1520 cm-1; ^{1}H NMR (400 MHz, $CDCl_3$) δ 7.36 (m, 4H), 7.28 (m, 1H), 4.80 (ddd, *J* = 13.4, 10.1, 4.9 Hz, 1H), 4.73 (m, 1H), 3.96 (m, 2H), 3.70–3.62 (m, 2H), 3.21 (dd, *J* = 14.2, 7.1 Hz, 1H), 2.97 (t, *J* = 13.3 Hz,

1H), 2.88 (d, J = 15.1 Hz, 1H), 2.11–2.02 (m, 4H), 1.84–1.72 (m, 2H), 1.66–1.55 (m, 2H), 1.50–1.19 (complex, 7H), 1.05 (m, 1H), 0.97–0.84 (m, 5H, contains s, 0.92, 3H), 0.76–0.69 (m, 4H, contains s, 0.74, 3H); ^{13}C NMR (151 MHz, $CDCl_3$) δ 206.2, 143.9, 128.6 (2C), 127.4, 125.5 (2C), 81.8, 70.1, 56.1, 54.4, 54.1, 51.0, 48.3, 42.9, 42.2, 38.2, 37.4, 37.3, 36.6, 35.1, 31.6, 30.6, 28.2, 23.4, 20.6, 12.6, 11.3. HRMS (FT-ICR, HESI) *m/z*: $[M + H]^+$ calcd for $C_{28}H_{42}NO_2S$ 456.2931, found 456.2921.

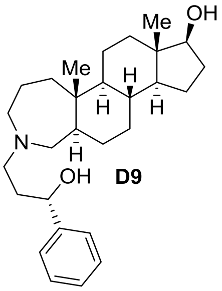

D9

17β-Hydroxy-5α-androstane-derived A-Ring Amine, D9

Method 1: Following the general procedure B, (*S*)-3-azido-1-phenylpropanol (*S*)-**7** (71.1 mg, 0.401 mmol, 2.0 equiv) was reacted with 5α-DHT **2** (58.9 mg, 0.203 mmol) to give **D9** as pale yellow sticky solid (43.1 mg, 0.101 mmol, 50% yield, UPLC/HRMS purity: ≥99.5%). Purification was carried out by an automated MPLC system using a 12 g normal phase silica column using $MeOH/CH_2Cl_2$. R_f = 0.15 (4% $MeOH/CH_2Cl_2$). HRMS (FT-ICR, HESI) *m/z*: $[M + H]^+$ calcd for $C_{28}H_{44}NO_2$ $[M + H]^+$ 426.3367, found 426.3357.

Method 2: **D2** (97.0 mg, 0.221 mmol) was added to a stirring suspension of LAH (1M THF, 0.43 mL, 0.430 mmol, 2.0 equiv) in anhydrous THF (10 mL) at 0 ˚C. The reaction mixture was allowed to room temperature, stirred for 4 h and then refluxed for 24 h. The reaction mixture was allowed to room temperature and quenched with a saturated aqueous solution of sodium potassium tartarate (5 mL). The biphasic mixture was stirred overnight. The biphasic mixture was diluted CH_2Cl_2 (50 mL). The organic layer was washed with aqueous solution of sodium potassium tartarate (2 × 5 mL), brine (5 mL), dried over Na_2SO_4, filtered, and concentrated. Purification was carried out by an automated MPLC system using a 12 g normal phase silica column using $MeOH/CH_2Cl_2$ to give **D9** as white foam solid (54.7 mg, 0.129 mmol, 58% yield, UPLC/HRMS purity: ≥99.5%). mp decomposed; IR (neat) 3337, 2920, 2848, 1448 cm^{-1}; ^{1}H NMR (400 MHz, $CDCl_3$) δ 7.36 (m, 4H), 7.23 (m, 1H), 4.95 (dd, J = 6.9, 4.7 Hz, 1H), 3.63 (t, J = 8.5 Hz, 1H), 2.86–2.61 (complex, 5H), 2.35 (d, J = 13.6 Hz, 1H), 2.05 (m, 1H), 1.83–1.18 (complex, 17H), 1.04 (m, 1H), 0.98–0.82 (complex, 5H, contains s, 0.82, 3H), 0.76–0.67 (complex, 4H, contains s, 0.73, 3H); ^{13}C NMR (101 MHz, $CDCl_3$) δ 145.4, 128.3 (2C), 126.9, 125.7 (2C), 82.1, 75.7, 58.3, 57.5, 55.3, 53.5, 51.3, 45.7, 43.0, 40.0, 38.3, 37.1, 35.4, 34.6, 31.7, 30.8, 28.9, 23.5, 22.0, 21.4, 14.0, 11.3. HRMS (FT-ICR, HESI) *m/z*: $[M + H]^+$ calcd for $C_{28}H_{44}NO_2$ $[M + H]^+$ 426.3367, found 426.3356.

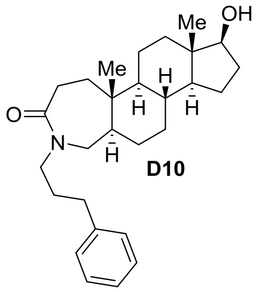

D10

17β-Hydroxy-5α-androstane-derived A-Ring Lactam, D10

Following the general procedure B, (*S*)-3-azido-1-phenylpropanol (*S*)-**7** (36.8 mg, 0.208 mmol, 2.1 equiv) was reacted with 5α-DHT **2** (29.0 mg, 0.100 mmol) to give **D10** as white foam solid (39.6 mg, 0.093 mmol, 93% yield, UPLC/HRMS purity: ≥99.5%). Purification was carried out by an automated

MPLC system using a 12 g normal phase silica column with gradient elution from 0–3% MeOH/CH_2Cl_2. R_f = 0.38 (4% MeOH/CH_2Cl_2); mp decomposed; IR (neat) 3212, 1621 cm^{-1}; ^1H NMR (400 MHz, $CDCl_3$) δ 7.34 (m, 2H), 7.25 (m, 3H), 3.67 (m, 2H), 3.47 (m, 2H), 2.68 (m, 3H), 2.55 (d, *J* = 15.5 Hz, 1H), 2.39 (m, 1H), 2.11 (m, 1H), 1.97–1.83 (complex, 4H), 1.79–1.59 (complex, 4H), 1.10 (m, 1H), 1.02–0.90 (complex, 5H, contains s, 0.95, 3H), 0.83–0.72 (complex, contains s, 0.79, 3H); ^{13}C NMR (101 MHz, $CDCl_3$) δ 175.3, 141.9, 128.5 (2C), 128.4 (2C), 126.0, 81.9, 54.2, 51.5, 51.1, 49.1, 48.0, 42.8, 38.3, 36.8, 36.0, 35.2, 33.4, 32.5, 31.5, 30.6, 30.3, 28.4, 23.4, 20.7, 12.2, 11.2. HRMS (FT-ICR, HESI) *m/z*: $[M + H]^+$ calcd for $C_{28}H_{42}NO_2$ $[M + H]^+$ 424.3210, found 424.3202.

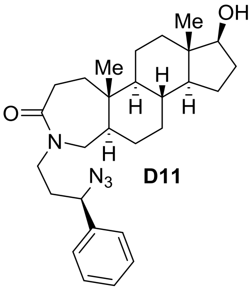

D11

17β-Hydroxy-5α-androstane-derived A-Ring Lactam, D11

Following the general procedure B, (*S*)-3-azido-1-phenylpropanol (*S*)-**7** (71.0 mg, 0.401 mmol, 2.0 equiv) was reacted with 5α-DHT **2** (58.3 mg, 0.201 mmol) to give **D11** as a yellow amorphous solid (67.9 mg, 0.146 mmol, 73% yield, UPLC/HRMS purity: ≥99.5%). Purification was carried out by an automated MPLC system using a using 12 g normal phase silica column with gradient elution from 0–80% EtOAc/hexanes. R_f = 0.34 (50% EtOAc/hexanes); mp 148–150 ˚C; IR (neat) 3353, 2094, 1624 cm^{-1}; ^1H NMR (400 MHz, $CDCl_3$) δ 7.39–7.28 (m, 5H), 4.44 (t, *J* = 7.1 Hz, 1H), 3.61 (t, *J* = 8.5 Hz, 1H), 3.57–3.50 (m, 2H), 3.26 (dt, *J* = 13.7, 7.0 Hz, 1H), 2.51 (m, 1H), 2.44 (d, *J* = 15.4 Hz, 1H), 2.29 (m, 1H), 2.03 (m, 1H), 1.95 (q, *J* = 7.3 Hz, 2H), 1.87–1.67 (complex, 4H), 1.64–1.52 (m, 2H), 1.47–1.17 (complex, 7H), 1.03 (m, 1H), 0.96–0.87 (m, 2H), 0.85 (s, 3H), 0.72–0.66 (complex, 4H, contains s, 3H); ^{13}C NMR (101 MHz, $CDCl_3$) δ 175.4, 139.2, 129.0 (2C), 128.5, 127.0 (2C), 81.8, 64.3, 54.1, 51.8, 51.0, 49.0, 45.9, 42.8, 38.2, 36.7, 35.9, 35.1, 34.6, 32.3, 31.4, 30.5, 28.4, 23.4, 20.7, 12.2, 11.2. HRMS (FT-ICR, HESI) *m/z*: $[M + H]^+$ calcd for $C_{28}H_{41}N_4O_2$ 465.3224, found 465.3223.

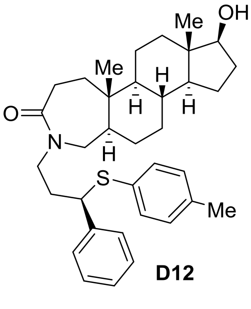

D12

17β-Hydroxy-5α-androstane-derived A-Ring Lactam, D12

Following the general procedure B, (*S*)-3-azido-1-phenylpropanol (*S*)-**7** (53.4 mg, 0.301 mmol, 2.0 equiv) was reacted with 5α-DHT **2** (45.7 mg, 0.157 mmol) to give **D12** as a yellow amorphous solid (57.3 mg, 0.105 mmol, 67% yield, UPLC/HRMS purity: 94.2%). Purification was carried out by an automated MPLC system using a using 12 g normal phase silica column with gradient elution from 0–40% EtOAc/hexanes. R_f = 0.22 (50% EtOAc/hexanes); mp 149–153 ˚C; IR (neat) 3390, 1632 cm^{-1}; ^1H NMR (400 MHz, $CDCl_3$) δ 7.26–7.16 (m, 5H), 7.11 (m, 2H), 7.00 (m, 2H), 4.04 (t, *J* = 7.2 Hz, 1H), 4.62 (t, *J* = 8.6 Hz, 1H), 3.46–3.52 (m, 3H), 2.46–2.35 (m, 2H), 2.28–2.22 (m, 4H, contains s, 2.28, 3H), 2.15-2.00 (m, 3H, contains q, 2.12, *J* = 7.3 Hz, 2H), 1.84–1.77 (m, 2H), 1.70–1.53 (complex, 5H), 1.47–1.16 (complex, 10H), 1.06–0.98 (m, 1H), 0.95–0.82 (m, 4H, contains s, 3H, 0.83), 0.72 (s, 3H), 0.66 (m, 1H); ^{13}C NMR (101 MHz, $CDCl_3$) δ 175.4, 141.8, 137.7, 133.4 (2C), 130.6, 129.6 (2C), 128.5 (2C), 127.9 (2C), 127.3, 81.9, 54.1, 51.7, 51.1, 49.0, 46.9, 42.9, 38.3, 36.8, 35.9, 35.2, 34.3, 32.3, 31.4,

30.6, 28.4, 23.5, 21.3, 20.7, 12.2, 11.2. **Note**: Missing one carbon signal due to signal overlap. HRMS (FT-ICR, HESI) *m/z*: $[M + H]^+$ calcd for $C_{35}H_{48}NO_2S$ 546.3400, found 546.3394.

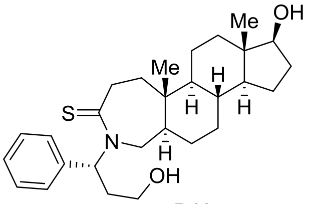

D13

17β-Hydroxy-5α-androstane-derived A-Ring Thioamide, D13

Following the general procedure B, (*R*)-3-azido-3-phenylpropanol (*R*)-**9** (73.4 mg, 0.414 mmol, 2.0 equiv) was reacted with 5α-DHT **2** (58.9 mg, 0.203 mmol) to give **D13** as a pale yellow amorphous solid (54.5 mg, 0.120 mmol, 59% yield, UPLC/HRMS purity: ≥99.5%). Purification was carried out using 12 g silica column with gradient elution from 0–50% EtOAc/hexanes. R_f = 0.34 (50% EtOAc/hexanes); IR (neat) 3410, 3310, 1495, 1451 cm^{-1}; mp 243–245 ˚C; ^1H NMR (400 MHz, $CDCl_3$) δ 7.43–7.31 (m, 5H), 7.14 (dd, J = 12.0, 3.2 Hz, 1H), 3.74 (m, 1H), 3.60–3.45 (m, 3H), 3.36 (dd, J = 14.8, 7.8 Hz, 1H), 3.01 (m, 1H), 2.72 (dd, J = 14.9 Hz, 1H), 2.18 (m, 1H), 2.11–1.96 (m, 2H), 1.88 (m, 1H), 1.75 (dt, J = 12.3, 3.4 Hz, 1H), 1.56–1.07 (complex, 9H), 1.00–0.73 (complex, 6H, contains s, 0.78, 3H), 0.63 (s, 3H), 0.40 (m, 3H), 0.18 (m, 1H); ^{13}C NMR (151 MHz, $CDCl_3$) δ 206.4, 138.1, 128.8 (2C), 128.7, 128.6 (2C), 81.9, 61.1, 58.1, 54.2, 50.8, 49.6, 47.9, 42.8, 42.3, 37.8, 37.2, 36.6, 35.0, 32.5, 31.1, 30.5, 27.1, 23.3, 20.5, 12.4, 11.2. HRMS (FT-ICR, HESI) *m/z*: $[M + H]^+$ calcd for $C_{28}H_{42}NO_2S$ 456.2931, found 456.2920.

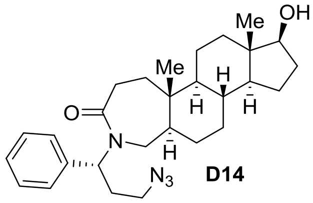

D14

17β-Hydroxy-5α-androstane-derived A-Ring Lactam, D14

Following the general procedure B, (*R*)-3-azido-3-phenylpropanol (*R*)-**9** (71.6 mg, 0.404 mmol, 2.0 equiv) was reacted with 5α-DHT **2** (58.0 mg, 0.200 mmol) to give **D14** as an orange amorphous solid (60.5 mg, 0.130 mmol, 65% yield, UPLC/HRMS purity: 94.4%). Purification was carried out by an automated MPLC system using a 12 g silica column with gradient elution from 0–60% EtOAc/hexanes. R_f = 0.28 (50% EtOAc/hexanes); mp 228–235 ˚C; IR (neat) 3275, 2091, 1611 cm^{-1}; ^1H NMR (400 MHz, $CDCl_3$) δ 7.34–7.28 (m, 5H), 5.93 (t, *J* = 7.7 Hz, 1H), 3.56 (t, *J* = 8.5 Hz, 1H), 3.42–3.25 (m, 3H), 2.64 (m, 1H), 2.49–2.42 (m, 2H, contains d, 1.57, *J* = 15.2 Hz, 1H), 2.14 (q, *J* = 7.5 Hz, 2H), 2.00 (m, 1H), 1.83 (m, 1H), 1.75 (m, 1H), 1.57–1.32 (m, 4H), 1.21–1.08 (m, 4H), 1.02–0.91 (m, 2H), 0.81–0.73 (m, 4H), 0.66 (s, 3H), 0.56 (m, 1H), 0.39 (m, 1H), 0.34–0.20 (m, 2H); ^{13}C NMR (101 MHz, $CDCl_3$) δ 175.6, 139.2, 128.7 (2C), 128.3 (2C), 128.1, 81.9, 54.1, 53.2, 50.9, 49.1, 48.7, 45.2, 42.8, 38.1, 36.7, 36.1, 35.1, 32.6, 31.2, 30.6, 29.5, 27.6, 23.4, 20.6, 12.1, 11.2. HRMS (FT-ICR, HESI) *m/z*: $[M + H]^+$ calcd for $C_{28}H_{41}N_4O_2$ 465.3224, found 465.3217.

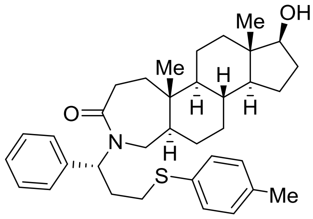

D15

17β-Hydroxy-5α-androstane-derived A-Ring Lactam, D15

Following the general procedure B, (*R*)-3-azido-3-phenylpropanol (*R*)-**9** (44.5 mg, 0.250 mmol, 2.0 equiv) was reacted with 5α-DHT **2** (36.3 mg, 0.125 mmol) to give **D15** as a cream-colored amorphous solid (57.0 mg, 0.104 mmol, 83% yield, UPLC/HRMS purity: 97.5%). Purification was carried out using 12 g silica column with gradient elution from 0–50% EtOAc/hexanes. R_f = 0.27 (50% EtOAc/hexanes); IR (neat) 3400, 1625 cm^{-1}; mp decomposed; ^1H NMR (400 MHz, $CDCl_3$) δ 7.30–7.21 (m, 7H), 7.07–7.05 (m, 2H), 5.89 (dd, *J* = 9.3, 6.1 Hz, 1H), 3.53 (t, *J* = 8.5 Hz, 1H), 3.19 (dd, *J* = 15.5, 8.9 Hz, 1H), 2.93–2.86 (m, 1H), 2.78–2.71 (m, 1H), 2.59 (t, *J* = 13.6 Hz, 1H), 2.42–2.37 (m, 1H), 2.28 (s, 3H), 2.19–2.05 (m, 2H), 1.98–1.93 (m, 1H), 1.79 (m, 1H), 1.71 (m, 1H), 1.53–1.05 (complex, 8H), 0.97–0.85 (m, 2H), 0.78–0.69 (m, 4H, contains s, 0.74, 3H), 0.62 (s, 3H), 0.52 (m, 1H), 0.34 (m, 1H), 0.23 (m, 2H); ^{13}C NMR (101 MHz, $CDCl_3$) δ 175.4, 139.6, 136.5, 132.5, 130.5, 129.9, 128.6, 128.4, 127.9, 81.9, 54.9, 54.1, 50.9, 48.7, 45.1, 42.8, 38.0, 36.7, 36.2, 35.1, 32.7, 31.5, 31.2, 30.6, 30.2, 27.6, 23.4, 21.1, 20.6, 12.1, 11.1. HRMS (FT-ICR, HESI) *m/z*: $[M + H]^+$ calcd for $C_{35}H_{48}NO_2S$ 546.3400, found 546.3396.

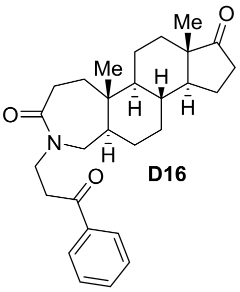

D16

17β-Hydroxy-5α-androstane-derived A-Ring Lactam, D16

Following the general procedure C, **D2** (93.0 mg, 0.212 mmol) was oxidized to give **D16** as a white amorphous solid (68.6 mg, 0.157 mmol, 74% yield, UPLC/HRMS purity: 96.1%). Purification was carried out by an automated MPLC system using a 12 g normal phase silica column with gradient elution from 0–2% MeOH/CH_2Cl_2. Concentration of solvents afforded a slightly impure azasteroid, which was subjected to a second purification on a 4 g normal phase silica column with gradient elution 0–80% EtOAc/hexanes. R_f = 0.20 (50% EtOAc/hexanes); mp 217–221 ˚C; IR (neat) 1723, 1676, 1644 cm^{-1}; ^1H NMR (400 MHz, $CDCl_3$) δ 7.98 (m, 2H), 7.57 (m, 1H), 7.47 (m, 2H), 3.82 (dt, *J* = 13.3, 6.6 Hz, 1H), 3.73–3.65 (m, 2H), 3.33–3.20 (m, 2H), 2.81 (d, *J* = 15.5 Hz, 1H), 2.60 (m, 1H), 2.43 (dd, *J* = 19.9, 8.3 Hz, 1H), 2.33 (dd, *J* = 14.4, 7.7 Hz, 1H), 2.10–2.01 (m, 1H), 1.94–1.75 (complex, 4H), 1.72–1.67 (m, 1H), 1.54–1.43 (complex, 3H), 1.38–1.16 (complex, 6H), 0.97–0.89 (m, 4H, contains s, 0.89, 3H), 0.84 (s, 3H), 0.69 (1H); ^{13}C NMR (101 MHz, $CDCl_3$) δ 221.0, 199.2, 175.7, 136.9, 133.5, 128.8 (2C), 128.3 (2C), 54.1, 53.2, 51.4, 49.1, 47.6, 45.4, 38.4, 37.9, 36.0, 35.8, 34.7, 32.4, 31.6, 30.7, 28.0, 21.9, 20.4, 13.9, 12.3. HRMS (FT-ICR, HESI) *m/z*: $[M + H]^+$ calcd for $C_{28}H_{38}NO_3$ 436.2846, found 436.2851.

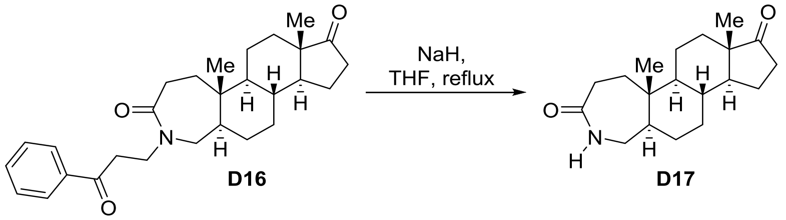

17β-Hydroxy-5α-androstane-derived A-Ring Lactam, D17

A solution of **D16** (87.0 mg, 0.200 mmol) and sodium hydride (60% dispersion in mineral oil, 38.0 mg, 1.60 mmol, 4.8 equiv) in anhydrous THF (8.0 mL) was heated at 65 ˚C for 2 h. The reaction was cooled to room temperature and quenched with a saturated solution of NH_4Cl (5 mL). The reaction mixture was diluted with CH_2Cl_2 (20 mL) and washed with saturated solution of NH_4Cl (2 x 5 mL), H_2O (5 mL)

and brine (5 mL). The combined organic layer was dried over anhydrous Na_2SO_4 filtered, and concentrated. The crude residue was purified by an automated MPLC system using a 12 g normal phase silica column with gradient elution from 0–4% MeOH/CH_2Cl_2. Concentration of solvents afforded product **D17** as white amorphous solid (46.7 mg, 0.153 mmol, 76% yield, UPLC/HRMS purity: ≥99.5%). R_f = 0.25 (4% MeOH/CH_2Cl_2); mp decomposed; IR (neat) 3202, 3091, 1732, 1647 cm^{-1}. ^{1}H NMR (400 MHz, $CDCl_3$) δ 6.02 (br s, 1H), 3.39 (m, 1H), 2.60 (m, 2H), 2.44 (dd, J = 19.2, 8.9 Hz, 1H), 2.27 (m, 1H), 2.07 (m, 1H), 1.96–1.21 (complex, 16H), 1.00–0.79 (complex, 5H, contains s, 0.94, 3H; s, 0.86, 3H); ^{13}C NMR (151 MHz, $CDCl_3$) δ 220.9, 178.5, 54.0, 51.5, 49.8, 47.6, 44.6, 39.0, 36.0, 35.4, 34.8, 31.7, 31.4, 30.7, 27.6, 21.9, 20.4, 13.9, 12.2. HRMS (FT-ICR, HESI) *m/z*: $[M + H]^+$ calcd for $C_{19}H_{30}NO_2$ 304.2271, found 304.2270.

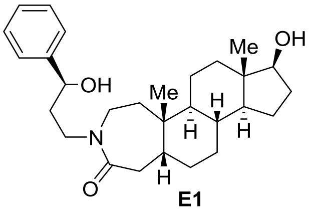

E1

17β-Hydroxy-5β-androstane-derived A-Ring Lactam, E1

Following the general procedure A, (*S*)-3-azido-1-phenylpropanol (*S*)-**7** (105 mg, 0.593 mmol, 2.0 equiv) was reacted with 5β-DHT **3** (87.6 mg, 0.302 mmol) to give **E1** a white amorphous solid (117 mg, 0.267 mmol, 89% yield, UPLC/HRMS purity: 97.6%). Purification was carried out by an automated MPLC system using a 12 g normal phase silica column with gradient elution from 0–2% MeOH/CH_2Cl_2. R_f = 0.35 (4% MeOH/CH_2Cl_2); mp 79–101 ˚C; IR (neat) 3374, 1616 cm^{-1}; ^{1}H NMR (400 MHz, $CDCl_3$) δ 7.38–7.30 (m, 4H), 7.25–7.22 (m, 1H), 4.63 (dd, J = 9.8, 3.2 Hz, 1H), 4.04 (ddd, J = 14.5, 10.5, 4.2 Hz, 1H), 3.66 (t, J = 8.5 Hz, 1H), 3.53 (dd, J = 15.4, 10.5 Hz, 1H), 3.15 (dt, J = 14.1, 4.7 Hz, 1H), 3.08–2.97 (m, 2H), 2.13–2.03 (m, 2H, contains d, J = 14.4 Hz, 1H), 1.99-1.75 (complex, 5H), 1.60 (m, 1H), 1.48–1.21 (complex, 10H), 1.15–1.01 (m, 5H, contains s, 1.01, 3H), 0.95–0.83 (m, 1H), 0.75 (s, 3H); ^{13}C NMR (151 MHz, $CDCl_3$) δ 176.8, 144.3, 128.5 (2C), 127.2, 125.7 (2C), 82.0, 70.2, 51.1, 45.9, 44.5, 43.2, 42.2, 41.0, 40.0, 39.3, 37.6, 37.1, 37.0, 36.0, 30.8, 29.9, 25.7, 24.2, 23.5, 21.0, 11.3. **Note**: HSQC and HMBC are included in the spectra section. HRMS (FT-ICR, HESI) *m/z*: $[M + H]^+$ calcd for $C_{28}H_{42}NO_3$ 440.3159, found 440.3148.

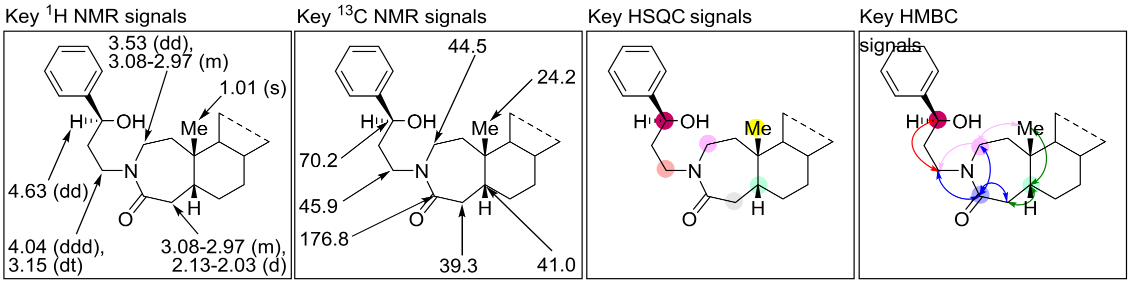

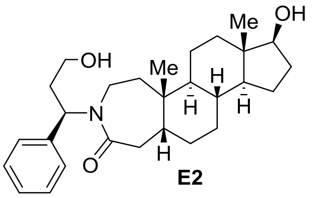

E2

17β-Hydroxy-5β-androstane-derived A-Ring Lactam, E2

Following the general procedure A, (*R*)-3-azido-3-phenylpropanol (*R*)-**9** (43.8 mg, 0.247 mmol, 2.0 equiv) was reacted with 5β-DHT **3** (36.1 mg, 0.124 mmol) to give **E2** as a white amorphous solid (49.1 mg, 0.112 mmol, 90% yield, UPLC/HRMS purity: 97.7%). Purification was carried out by an automated MPLC system using a 12 g normal phase silica column with gradient elution from 0–5%

MeOH/CH_2Cl_2. R_f = 0.38 (5% MeOH/CH_2Cl_2); IR (neat) 3369, 1615 cm^{-1}; mp decomposed; ^1H NMR (400 MHz, $CDCl_3$) δ 7.37–7.26 (m, 5H), 5.97 (dd, J = 12.4, 3.3 Hz, 1H), 3.72 (ddd, J = 12.0, 5.1, 2.6 Hz, 1H), 3.63 (t, J = 8.5 Hz, 1H), 3.44 (td, J = 11.6, 2.9 Hz, 1H), 3.17 (dd, J = 15.5, 11.9 Hz, 1H), 3.03 (dd, J = 15.2, 8.0 Hz, 1H), 2.45 (br s, 1H), 2.27 (d, J = 15.4 Hz, 1H), 2.18–2.01 (m, 2H), 1.97–1.75 (m, 4H), 1.61–1.53 (m, 1H), 1.49–0.97 (complex, 12H), 0.94–0.77 (m, 5H, contains s, 0.82, 3H), 0.69 (s, 3H); ^{13}C NMR (151 MHz, $CDCl_3$) δ 176.7, 138.9, 128.7, 128.5, 127.9, 81.9, 58.6, 52.8, 51.0, 43.1, 42.7, 40.2, 39.6, 38.9, 38.2, 36.9, 36.7, 35.9, 31.9, 30.7, 29.7, 25.6, 23.4, 20.9, 11.3. **Note**: Missing one carbon signal due to signal overlap. NOE (1D), HSQC and HMBC are included in the spectra section. HRMS (FT-ICR, HESI) m/z: $[M + H]^+$ for $C_{28}H_{42}NO_3$ 440.3159, found 440.3180.

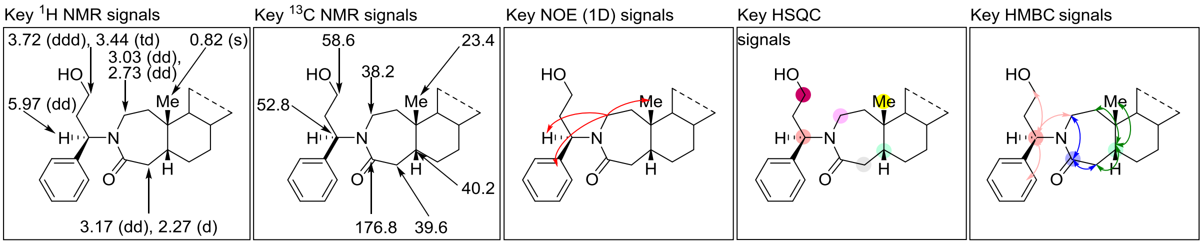

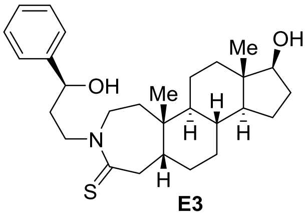
E3

17β-Hydroxy-5β-androstane-derived A-Ring Thioamide, E3

Following the general procedure B, (S)-3-azido-1-phenylpropanol (S)-**7** (73.5 mg, 0.415 mmol, 2.1 equiv) was reacted with 5β-DHT **3** (58.9 mg, 0.203 mmol) to give **E3** as a pale yellow foam solid (57.7 mg, 0.127 mmol, 62% yield, UPLC/HRMS purity: 98.0%). Purification was carried out using 12 g silica column with gradient elution from 0–50% EtOAc/hexanes. R_f = 0.33 (50% EtOAc/hexanes); IR (neat) 3425, 3312, 1495, 1451 cm^{-1}; ^1H NMR (400 MHz, $CDCl_3$) δ 7.38–7.32 (m, 4H), 7.29–7.25 (m, 1H), 4.75–4.64 (m, 2H), 3.85 (m, 2H), 3.74 (m, 1H), 3.65 (m, 1H), 3.46 (dd, J = 13.8, 11.2 Hz, 1H), 3.26 (dd, J= 15.2, 7.0 Hz, 1H), 2.92 (d, J = 13.8 Hz, 1H), 2.16–1.89 (complex, 4H), 1.85 (m, 1H), 1.77 (m, 1H), 1.64–1.02 (complex, 11H), 1.00 (s, 3H), 0.86 (m, 2H), 0.75 (s, 3H); ^{13}C NMR (151 MHz, $CDCl_3$) δ 206.2, 143.9, 128.6 (2C), 127.5, 125.7 (2C), 82.0, 70.3, 53.9, 51.1, 49.2, 48.6, 43.2, 42.8, 41.6 (br), 38.4, 37.1, 37.0, 36.9, 35.9, 30.8, 30.2, 25.7, 24.8 (br), 23.5, 21.0, 11.3. **Note**: Two broad carbon signals were consistently observed amongst cis-AB analogs. HRMS (FT-ICR, HESI) m/z: $[M + H]^+$ calcd for $C_{28}H_{42}NO_2S$ 456.2931, found 456.2920.

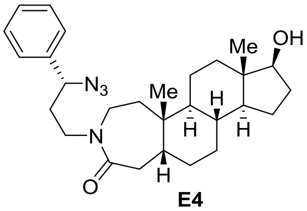
E4

17β-Hydroxy-5β-androstane-derived A-Ring Lactam, E4

Following the general procedure B, (S)-3-azido-1-phenylpropanol (S)-**7** (69.9 mg, 0.394 mmol, 2.0 equiv) was reacted with 5β-DHT **3** (58.2 mg, 0.200 mmol) to give **E4** as a cream-colored amorphous solid (76.1 mg, 0.164 mmol, 82% yield, UPLC/HRMS purity: 98.8%). Purification was carried out by an automated MPLC system using a 12 g normal phase silica column with gradient elution from 0–50% EtOAc/hexanes. R_f = 0.25 (50% EtOAc/hexanes); mp 137–142 ˚C; IR (neat) 3393, 2094, 1628 cm^{-1}; ^1H NMR (400 MHz, $CDCl_3$) δ 7.41–7.30 (m, 5H), 4.45 (t, J = 7.2 Hz, 1H), 3.65 (t, J = 8.6 Hz, 1H), 3.62–

3.55 (m, 1H), 3.37 (dd, J = 15.2, 10.2 Hz, 1H), 3.26 (m, 1H), 3.02–2.92 (m, 2H), 2.12–2.03 (m, 2H, contains d, 2.06, J = 14.7 Hz, 1H), 1.97 (q, J = 7.3 Hz, 2H), 1.92–1.82 (m, 2H), 1.73 (m, 1H), 1.63–1.58 (m, 1H), 1.47–1.20 (complex, 10H), 1.13–0.96 (m, 5H, contains s, 0.98, 3H), 0.92–0.82 (m, 1H), 0.74 (s, 3H); ^{13}C NMR (101 MHz, $CDCl_3$) δ 175.0, 139.4, 129.0 (2C), 128.5, 127.0 (2C), 82.0, 64.4, 51.0, 45.9, 44.0, 43.2 (br), 42.6, 40.6, 39.7, 39.6, 37.0, 36.9, 36.0, 34.5, 30.8, 29.9, 25.7, 23.8 (br), 23.5, 21.0, 11.3. **Note**: Two broad carbon signals were consistently observed amongst *cis*-AB analogs. HRMS (FT-ICR, HESI) *m/z*: $[M + H]^+$ calcd for $C_{28}H_{41}N_4O_2$ 465.3224, found 465.3254.

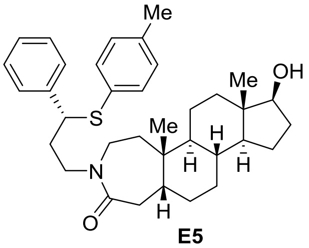

E5

17β-Hydroxy-5β-androstane-derived A-Ring Lactam, E5

Following the general procedure B, (*S*)-3-azido-1-phenylpropanol (*S*)-**7** (53.0 mg, 0.299 mmol, 2.0 equiv) was reacted with 5β-DHT **3** (43.9 mg, 0.151 mmol) to give **E5** as a white crystalline solid (46.1 mg, 0.084 mmol, 56% yield, UPLC/HRMS purity: 98.6%). Purification was carried out using 12 g silica column with gradient elution from 0–40% EtOAc/hexanes. R_f = 0.33 (50% EtOAc/hexanes); IR (neat) 3380, 1621 cm^{-1}; mp 142–147 ˚C; ^{1}H NMR (400 MHz, $CDCl_3$) δ 7.26–7.17 (m, 5H), 7.13 (m, 2H), 7.00 (m, 2H), 4.04 (t, J = 7.2 Hz, 1H), 3.65 (t, J = 8.5 Hz, 1H), 3.46–3.29 (m, 2H), 3.23 (dd, J = 10.4, 15.2 Hz, 1H), 2.90–2.81 (m, 2H), 2.28 (s, 3H), 2.18–2.04 (m, 3H), 1.99 (d, J = 14.6 Hz, 1H), 1.89–1.75 (m, 3H), 1.68–1.54 (m, 2H), 1.47–0.94 (complex, 11H), 0.94 (s, 3H), 0.91–0.78 (m, 1H), 0.73 (s, 3H); ^{13}C NMR (101 MHz, $CDCl_3$) δ 175.0, 141.8, 137.8, 133.6 (2C), 130.7, 129.6 (2C), 128.5 (2C), 127.9 (2C), 127.3, 82.0, 52.0, 51.1, 47.0, 43.8, 43.2, 42.3 (br), 40.5, 39.6, 39.5, 37.0, 36.9, 35.9, 34.0, 30.8, 29.8, 25.7, 23.8 (br), 23.5, 21.3, 21.0, 11.3. **Note**: Two broad carbon signals were consistently observed amongst *cis*-AB analogs. HRMS (FT-ICR, HESI) *m/z*: $[M + H]^+$ calcd for $C_{35}H_{48}NO_2S$ 546.3400, found 546.3394.

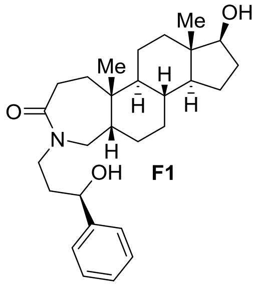

F1

17β-Hydroxy-5β-androstane-derived A-Ring Lactam, F1

Following the general procedure A, (*R*)-3-azido-1-phenylpropanol (*R*)-**7** (44.3 mg, 0.250 mmol, 2.0 equiv) was reacted with 5β-DHT **3** (36.6 mg, 0.126 mmol) to give **F1** as a white amorphous solid (45.9 mg, 0.104 mmol, 83% yield, UPLC/HRMS purity: ≥99.5%). Purification was carried out by an automated MPLC system using a 12 g normal phase silica column with gradient elution from 0–3.5% MeOH/CH_2Cl_2. R_f = 0.44 (5% MeOH/CH_2Cl_2); mp 79–101 ˚C; IR (neat) 3367, 1626 cm^{-1}; ^{1}H NMR (400 MHz, $CDCl_3$) δ 7.36–7.32 (m, 4H), 7.27–7.23 (m, 1H), 4.63 (dd, J = 9.6, 3.4 Hz, 1H), 4.10–4.01 (m, 2H), 3.66 (t, J = 8.5 Hz, 1H), 3.14 (dt, J = 14.1, 4.7 Hz, 1H), 2.74 (d, J = 15.4 Hz, 1H), 2.56 (dd, J = 15.0, 11.8 Hz, 1H), 2.29 (m, 1H), 2.07 (m, 1H), 1.95–1.79 (complex, 5H), 1.62–1.21 (complex, 11H), 1.18–1.09 (m, 1H), 1.06–0.99 (m, 4H, contains s, 1.02, 3H), 0.91–0.75 (m, 5H, contains s, 0.75, 3H); ^{13}C NMR (151 MHz, $CDCl_3$) δ177.2, 144.2, 128.5 (2C), 127.3, 125.7 (2C), 81.9, 70.3, 52.4, 51.0, 45.9, 45.6, 43.2, 37.9, 37.1, 37.0, 36.0, 34.4, 30.7, 28.8, 26.6, 24.3, 23.5, 20.7, 11.3. **Note**: Missing two carbon

signals due to signal overlap. COSY, NOE (1D), HSQC and HMBC are included in the spectra section. HRMS (FT-ICR, HESI) *m/z*: $[M + H]^+$ calcd for $C_{28}H_{42}NO_3$ 440.3159, found 440.3136.

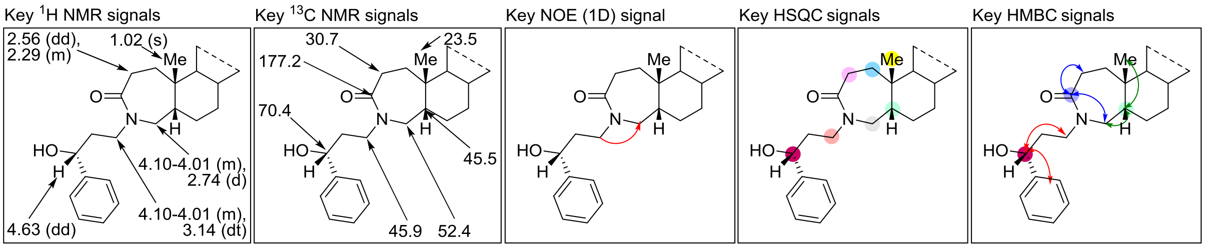

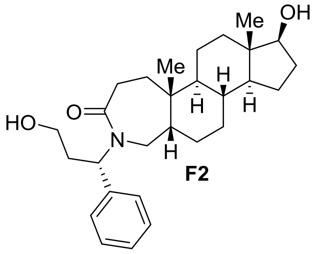

F2

17β-Hydroxy-5β-androstane-derived A-Ring Lactam, F2

Following the general procedure A, (*S*)-3-azido-3-phenylpropanol (*S*)-**9** (44.0 mg, 0.248 mmol, 2.0 equiv) was reacted with 5β-DHT **3** (36.6 mg, 0.126 mmol) to give **F2** as a white amorphous solid (48.1 mg, 0.109 mmol, 87% yield, UPLC/HRMS purity: 98.8%). Purification was carried out by an automated MPLC system using a 12 g normal phase silica column with gradient elution from 0–5% MeOH/CH_2Cl_2. R_f = 0.38 (5% MeOH/CH_2Cl_2); mp 223–227 ˚C; IR (neat) 3355, 1633 cm^{-1}; ^1H NMR (400 MHz, $CDCl_3$) δ 7.36–7.27 (m, 5H), 5.87 (dd, *J* = 12.2, 3.3 Hz, 1H), 3.73 (ddd, *J* = 12.1, 5.0, 2.6 Hz, 1H), 3.64–3.56 (m, 2H), 3.42 (td, *J* = 11.6, 2.9 Hz, 1H), 2.67 (dd, *J* = 15.3, 12.3 Hz, 1H), 2.57 (d, *J* = 15.2 Hz, 1H), 2.36 (dd, *J* = 15.4, 8.2 Hz, 1H), 2.11–1.79 (complex, 5H), 1.54–0.94 (complex, 13H), 0.79 (s, 3H), 0.69–0.53 (m, 5H, contains s, 0.69, 3H); ^{13}C NMR (151 MHz, $CDCl_3$) δ 177.5, 138.8, 128.6 (2C), 128.4 (2C), 127.9, 81.7, 58.2, 52.7, 50.8, 44.9, 44.4, 43.0, 41.1, 36.7, 35.6, 34.1, 31.8, 30.7, 30.4, 27.4, 26.2, 24.2, 23.2, 20.3, 11.0. **Note**: Missing one carbon signal due to signal overlap. NOE (1D), HSQC and HMBC have been included in the spectra section. HRMS (FT-ICR, HESI) *m/z*: $[M + H]^+$ calcd for $C_{28}H_{42}NO_3$ 440.3159, found 440.3178.

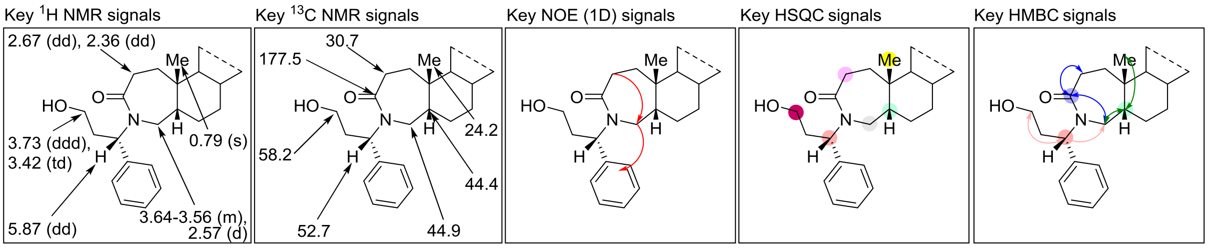

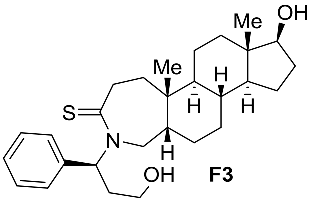

F3

17β-Hydroxy-5β-androstane-derived A-Ring Thioamide, F3

Following the general procedure B, (*S*)-3-azido-3-phenylpropanol (*S*)-**9** (72.0 mg, 0.406 mmol, 2.0 equiv) was reacted with 5β-DHT **3** (58.2 mg, 0.200 mmol) to give **F3** as a pale-yellow amorphous solid (71.6 mg, 0.156 mmol, 78% yield, UPLC/HRMS purity: ≥99.5%). Purification was carried out using 12 g silica column with gradient elution from 0–40% EtOAc/hexanes. R_f = 0.28 (50% EtOAc/hexanes);

mp 226 ˚C (decomposed); IR (neat) 3366, 1501, 1446 cm^{-1}; ^{1}H NMR (400 MHz, $CDCl_3$) δ 7.41–7.31 (m, 5H), 7.08 (dd, *J* = 10.7, 4.4 Hz, 1H), 3.85 (dd, *J* = 14.8, 10.2 Hz, 1H), 3.74 (m, 1H), 3.61 (m, 2H), 3.50 (m, 1H), 3.21 (dd, *J* = 15.1, 8.3 Hz, 1H), 2.98 (dd, *J* = 15.0, 11.8 Hz, 1H), 2.92 (d, *J* = 14.8 Hz, 1H), 2.21–1.99 (m, 3H), 1.93 (dd, *J* = 15.1, 8.4 Hz, 1H), 1.03 (m, 1H), 1.55–0.83 (complex, 11H), 0.74 (s, 3H), 0.72–0.57 (m, 2H), 0.69 (s, 3H), 0.39 (m, 1H); ^{13}C NMR (151 MHz, $CDCl_3$) δ 207.0, 138.2, 128.8 (2C), 128.7, 128.4 (2C), 81.9, 61.2, 58.3, 51.0, 49.5, 44.0, 43.2, 41.5, 40.9, 36.9, 36.6, 35.8, 34.9, 32.4, 30.6, 27.5, 26.3, 24.4, 23.4, 20.6, 11.2. **Note**: Two broad carbon signals were consistently observed amongst *cis*-AB analogs. HRMS (FT-ICR, HESI) *m/z*: [M + H]$^+$ calcd for $C_{28}H_{42}NO_2S$ [M + H]$^+$ 456.2931, found 456.2924.

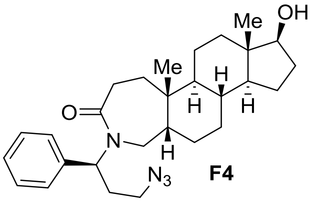

F4

17β-Hydroxy-5β-androstane-derived A-Ring Lactam, F4
Following the general procedure B, (*S*)-3-azido-3-phenylpropanol (*S*)-**9** (69.7 mg, 0.393 mmol, 2.0 equiv) was reacted with 5β-DHT **3** (58.2 mg, 0.200 mmol) to give **F4** as an orange amorphous solid (77.9 mg, 0.168 mmol, 84% yield, UPLC/HRMS purity: ≥99.5%). Purification was carried out by an automated MPLC system using a 12 g normal phase silica column with gradient elution from 0–40% EtOAc/hexanes. R_f = 0.22 (50% EtOAc/hexanes); mp 174–178 ˚C; IR (neat) 3396, 2095, 1628 cm^{-1}; ^{1}H NMR (400 MHz, $CDCl_3$) δ 7.35–7.27 (m, 5H), 5.88 (m, 1H), 3.70 (dd, *J* = 15.3, 10.1 Hz, 1H), 3.63 (t, *J* = 8.5 Hz, 1H), 3.41 (m, 1H), 3.29 (m, 1H), 2.68–2.58 (m, 2H), 2.32 (dd, *J* = 14.9, 8.5 Hz, 1H), 2.21–2.11 (m, 2H), 2.04 (m, 1H), 1.88–1.79 (m, 2H), 1.56–0.93 (complex, 13H), 0.85–0.79 (m, 4H, contains s, 0.77, 3H), 0.69 (s, 3H), 0.53 (m, 1H); ^{13}C NMR (101 MHz, $CDCl_3$) δ 175.8, 139.1, 128.7 (2C), 128.2 (2C), 128.1, 81.9, 53.6, 51.0, 49.1, 45.1, 44.7, 43.2, 41.4 (br), 36.94, 36.97, 35.9, 34.2, 31.1, 30.7, 29.5, 27.9, 26.4, 24.1 (br), 23.4, 20.6, 11.2. **Note**: Two broad carbon signals were consistently observed amongst *cis*-AB analogs. HRMS (FT-ICR, HESI) *m/z*: [M + H]$^+$ calcd for $C_{28}H_{41}N_4O_2$ 465.3224, found 465.3213.

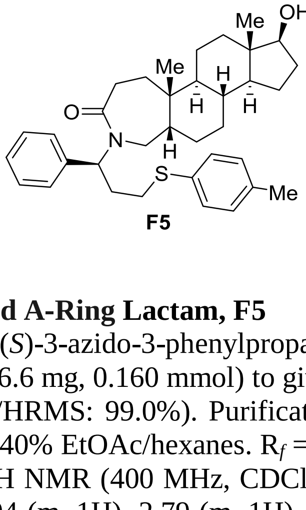

F5

17β-Hydroxy-5β-androstane-derived A-Ring Lactam, F5
Following the general procedure B, (*S*)-3-azido-3-phenylpropanol (*S*)-**9** (51.0 mg, 0.288 mmol, 2.0 equiv) was reacted with 5β-DHT **3** (46.6 mg, 0.160 mmol) to give **F5** as a white crystalline solid (66.8 mg, 0.122 mmol, 76% yield, UPLC/HRMS: 99.0%). Purification was carried out using 12 g silica column with gradient elution from 0–40% EtOAc/hexanes. R_f = 0.20 (40% EtOAc/hexanes); mp 212–215 ˚C; IR (neat) 3406, 1628 cm^{-1}; ^{1}H NMR (400 MHz, $CDCl_3$) δ 7.32–7.23 (m, 7H), 7.09 (m, 2H), 5.89 (m, 1H), 3.67–3.61 (m, 2H), 2.94 (m, 1H), 2.79 (m, 1H), 2.64–2.57 (m, 2H), 2.42–2.31 (m, 4H contains s, 2.31, 3H), 2.24–2.12 (m, 2H), 2.05 (m, 1H), 1.87–1.79 (m, 3H), 1.57–0.95 (complex, 11H), 0.88–0.74 (m, 4H, contains s, 0.76, 3H), 0.70–0.60 (4H, contains s, 0.70, 3H), 0.49 (m, 1H); ^{13}C NMR (101 MHz, $CDCl_3$) δ 175.8, 139.4, 136.4, 132.4, 130.4 (2C), 129.9 (2C), 128.6 (2C), 128.2 (2C), 128.0, 82.0, 55.3, 51.1, 45.0, 44.6, 43.2, 41.5 (br), 37.0, 36.9, 35.9, 34.1, 31.4, 30.9, 30.7, 30.1, 27.9, 26.4, 24.0 (br), 23.5, 21.2, 20.6, 11.2. **Note**: Two broad carbon signals were consistently observed amongst

cis-AB analogs. HRMS (FT-ICR, HESI) *m/z*: $[M + H]^+$ calcd for $C_{35}H_{48}NO_2S$ 546.3400, found 546.3414.

Characterization and regiochemistry discussion for D-ring library members

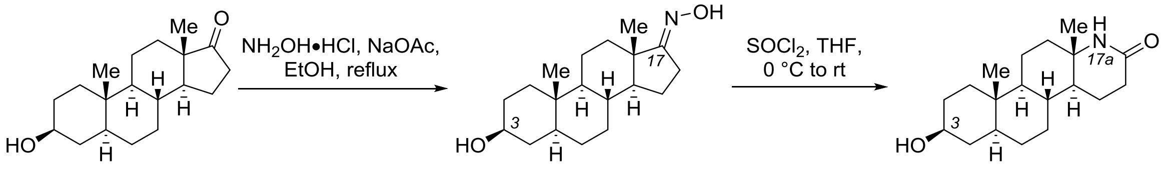

4 S12 Beck1

Synthesis of Beck1
Intermediate **S12** was prepared following a previously published procedure[27,28]. Characterization data were consistent with reported data.[11] UPLC/HRMS purity: ≥99.5%.

3β-Hydroxy-5α-androstane-derived D-Ring Lactam, Beck1[12]
Following the literature procedure[29], to a solution of **S12** (106 mg, 0.349 mmol) in anhydrous THF (7.0 mL, 0.05 M) at 0 ˚C was added $SOCl_2$ (0.25 mL, 10.0 equiv). The reaction mixture was stirred for 2 h at 0 ˚C, and stirred at room temperature overnight. The reaction mixture was terminated with a saturated aqueous solution of $NaHCO_3$ (20 mL) and H_2O (15 mL). The aqueous layer was extracted with CH_2Cl_2, and the combined organic layers were washed with a solution of saturated $NaHCO_3$, H_2O, brine, dried over Na_2SO_4, filtered, and concentrated. Purification was carried out by an automated MPLC system using a 12 g normal phase silica column with gradient elution of 0–5% MeOH/CH_2Cl_2. Concentration of appropriate fractions afforded product as a cream-colored amorphous solid (53.0 mg, 0.174 mmol, 50% yield, UPLC/HRMS purity: ≥99.5%). R_f = 0.19 (3% MeOH/CH_2Cl_2); mp 286–294 ˚C (decomposed); IR (neat) 3155, 3038, 1654 cm^{-1}; ^1H NMR (400 MHz, $CDCl_3$) δ 6.18 (s, 1H), 3.59 (m, 1H), 2.50–2.29 (m, 2H), 2.24 (br s, 1H), 1.95–1.76 (m, 3H), 1.73–1.63 (m, 3H), 1.59 (m, 1H), 1.50–1.16 (complex, 9H), 1.17–1.07 (complex, 4H, contains s, 1.14, 3H), 1.02–0.80 (complex, 3H), 0.79 (s, 3H); ^{13}C NMR (101 MHz, $CDCl_3$) δ 172.2, 71.2, 54.79, 53.82, 47.8, 44.4, 40.2, 38.0, 36.9, 35.9, 35.7, 31.5, 30.9, 30.6, 28.5, 22.3, 21.3, 19.9, 12.4. HRMS (FT-ICR, HESI) *m/z*: $[M + H]^+$ calcd for $C_{19}H_{32}NO_2$ 306.2428, found 306.2424.

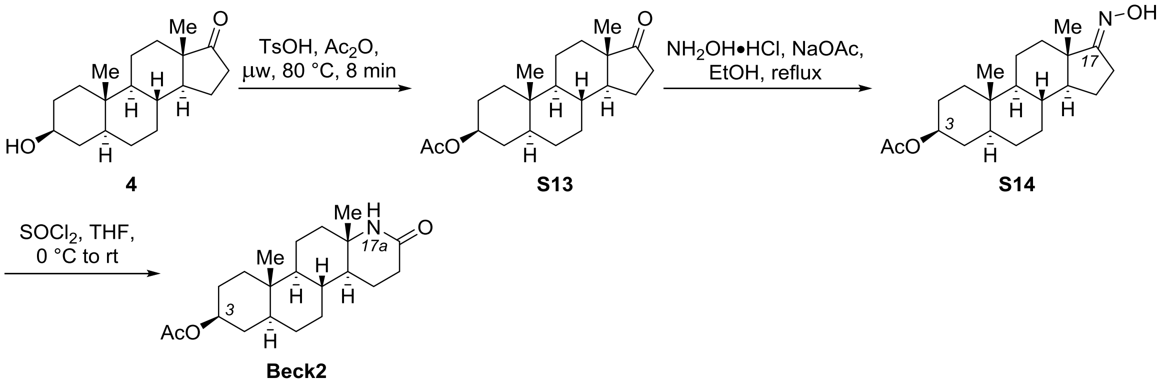

4 S13 S14

Beck2

Synthesis of Beck2
Intermediate **S13** was prepared and characterization data were consistent with reported data.[13]

3β-Acetoxy-5α-hydroxyandrostan-17-one Oxime, S14
Intermediate **S14** was prepared following a previously published procedure[27,28] as described. To a solution of **S13** (598 mg, 1.80 mmol), NH_2OH•HCl (500 mg, 7.20 mmol, 4.0 equiv), NaOAc (591 mg, 7.20 mmol, 4.0 equiv) in anhydrous EtOH (9.0 mL, 0.2 M) was refluxed for 6 h. EtOH was removed under a stream of nitrogen, and the residual crude was dissolved in H_2O (40 mL). The aqueous layer was extracted with Et_2O (3 x 15 mL), filtered, and concentrated. Purification was carried by an

automated MPLC system using a 24 g normal phase silica column with gradient elution of 0–30% EtOAc/hexanes. Concentration of fractions afforded product as a white amorphous solid (548 mg, 1.58 mmol, 88% yield, UPLC/HRMS purity: ≥99.5%). R_f = 0.44 (30% EtOAc/hexanes); mp 186–188 ˚C; IR (neat) 3456, 1714 cm^{-1}; ^{1}H NMR (400 MHz, $CDCl_3$) δ 4.68 (m, 1H), 2.62–2.45 (m, 2H), 2.03–1.99 (complex, 4H, contains s, 2.02, 3H), 1.86–1.80 (m, 3H), 1.76–1.71 (m, 2H), 1.67–1.59 (m, 2H), 1.55–1.15 (complex, 9H), 1.09–0.91 (complex, 5H, contains s, 0.93, 3H), 0.84 (s, 3H), 0.76–0.70 (m, 1H); ^{13}C NMR (101 MHz, $CDCl_3$) δ 173.9, 170.8, 73.7, 54.3, 54.0, 44.7, 36.8, 35.8, 34.9, 34.1, 33.9, 31.5, 28.4, 27.6, 26.1, 23.1, 21.6, 20.8, 17.1, 12.3. **Note**: Missing one carbon signal due to signal overlap. HRMS (FT-ICR, HESI) *m/z*: $[M + H]^+$ calcd for $C_{21}H_{34}NO_3$ 348.2533, found 348.2528.

3β-Hydroxy-5α-androstane-derived D-Ring Lactam, Beck2

Following a literature procedure[29], to a solution of **S14** (139 mg, 0.400 mmol) in anhydrous THF (5.0 mL) at 0 ˚C was added $SOCl_2$ (0.29 mL, 10.0 equiv). The reaction mixture was stirred for 2 h at 0 ˚C, and stirred at room temperature overnight. The reaction mixture was terminated with saturated aqueous solution of $NaHCO_3$ (20 mL) and H_2O (15 mL). The aqueous layer was extracted with CH_2Cl_2, and the combined organic layers were washed with a solution of saturated $NaHCO_3$, H_2O, brine, dried over Na_2SO_4, filtered, and concentrated. Purification was carried out by an automated MPLC system using a 12 g normal phase silica column with gradient elution of 0–2% MeOH/CH_2Cl_2. Concentration of fractions afforded product as a cream-colored amorphous solid (60.9 mg, 0.175 mmol, 44% yield, UPLC/HRMS purity: 98.0%). R_f = 0.25 (2% MeOH/CH_2Cl_2); mp decomposed; IR (neat) 3186, 3055, 1731, 1673 cm^{-1}; ^{1}H NMR (400 MHz, $CDCl_3$) δ 5.91 (br s, 1), 4.69 (m, 1H), 2.50–2.30 (m, 2H), 2.02 (s, 3H), 1.96–1.61 (complex, 7H), 1.55–1.17 (complex, 10H), 1.15 (s, 3H), 1.04 (m, 1H), 0.96–0.79 (complex, 5H, contains s, 0.81, 3H); ^{13}C NMR (101 MHz, $CDCl_3$) δ 172.0, 170.8, 73.5, 54.70 53.7, 47.7, 44.3, 40.2, 36.6, 35.9, 35.7, 33.9, 30.8, 30.7, 28.4, 27.5, 22.4, 21.6, 21.3, 19.9, 12.3. HRMS (FT-ICR, HESI) *m/z*: $[M + H]^+$ calcd for $C_{21}H_{34}NO_3$ 348.2533, found 348.2532.

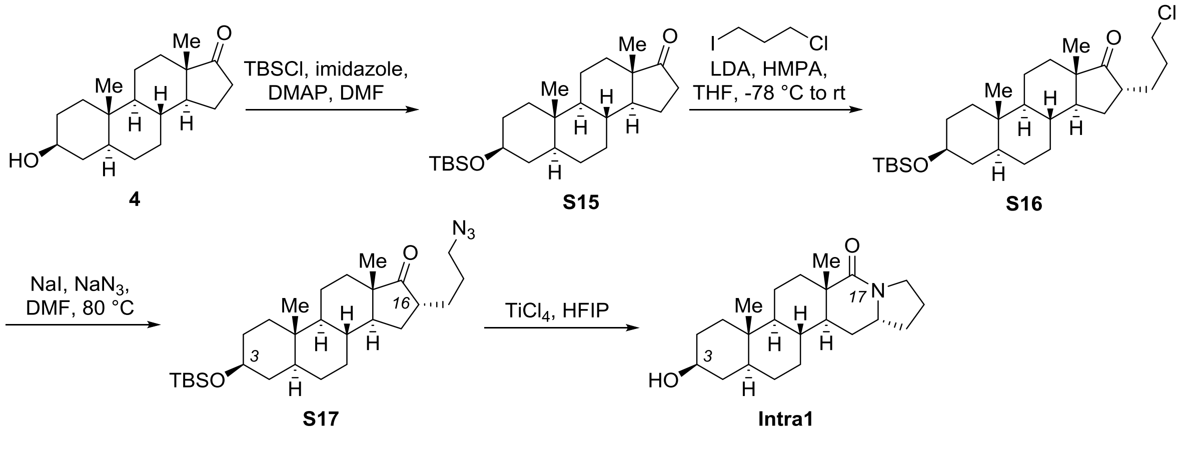

Intramolecular Schmidt reaction of *trans*-androsterone 4 and 5

Intermediate 3β-(*tert*-butyldimethylsilyl)oxy)-epiandrosterone **S15** was prepared following a previously published procedure.[14,30] Characterization data were consistent with reported data (mp 162–164 ˚C).[11,30]

3β-((*tert*-Butyldimethylsilyl)oxy)-16α-(3′-chloropropyl)-5α-androstan-17-one, S16

Following a literature procedure[31], **S16** was prepared as described: To a cooled 1.0 M LDA solution (260 µL, 1.82 mmol, 1.2 equiv) in anhydrous THF (15.0 mL, 0.1 M) at –78 ˚C was added **S15** (609 mg, 1.51 mmol) cautiously. The reaction mixture was allowed to –10 ˚C and stirred for 1 h. To the pale yellow solution, HMPA (1.10 mL, 6.32 mmol, 4.2 equiv) was added followed by 1-chloro-3-iodopropane (240 µL, 2.24 mmol, 1.5 equiv). The pale yellow solution was stirred at –10 ˚C for 1 h, warmed to room temperature, and stirred overnight. The reaction mixture was quenched with a solution of saturated NH_4Cl (40 mL) and H_2O (20 mL). The aqueous layer was extracted with Et_2O (3 × 30 mL). The combined organic layer was washed with brine (15 mL), dried over Na_2SO_4, filtered, and

concentrated. Purification was carried out by an automated MPLC system using a 24 g normal phase silica column with gradient elution of 0–40% CH_2Cl_2/hexanes. Fractions containing mixtures of **S15** and **S16** were subjected to a second automated purification using the same gradient. Concentration of fractions afforded product as a white amorphous solid (578 mg, 1.20 mmol, 80% yield). R_f = 0.80 (100% CH_2Cl_2); R_f = 0.58 (30% CH_2Cl_2/hexanes); IR (neat) 1736 cm^{-1}; ^1H NMR (400 MHz, $CDCl_3$) δ 3.58–3.48 (m, 3H), 2.42 (m, 1H), 1.93–1.16 (complex, 18H), 1.08 (m, 1H), 0.99–0.77 (complex, 19H, contains s, 0.89, 3H; s, 0.88, 9H; s, 0.82, 3H), 0.66 (m, 1H), 0.05 (s, 6H); ^{13}C NMR (101 MHz, $CDCl_3$) δ 222.1, 72.1, 54.7, 49.5, 48.7, 45.2, 45.0, 44.2, 38.8, 37.3, 35.9, 35.2, 32.0, 31.9, 31.3, 31.0, 28.8, 28.6, 28.0, 26.1 (3C), 20.5, 18.4, 14.7, 12.5, -4.4 (2C). **Note**: HMBC and NOE (1D) have been included in the spectra section. HRMS (FT-ICR, APCI) *m/z*: $[M + H]^+$ calcd for $C_{22}H_{50}ClO_2Si$ 481.3263, found 481.3259.

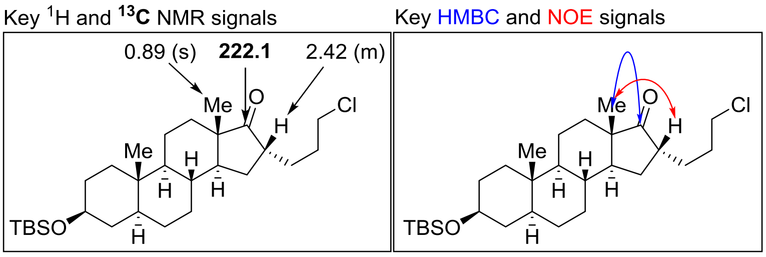

16α-(3′-Azidopropyl)-3β-((*tert*-butyldimethylsilyl)oxy)-5α-androstan-17-one, S17

Following a literature procedure[31], **S17** was prepared as described. A mixture of **S16** (482 mg, 1.00 mmol), NaI (300 mg, 2.00 mmol, 2.0 equiv), NaN_3 (195 mg, 3.00 mmol, 3.0 equiv) in anhydrous DMF (10.0 mL, 1.0 M) was stirred at 80 ˚C overnight. The reaction mixture was quenched with H_2O (50 mL). The aqueous layer was extracted with Et_2O (3 × 30 mL). The combined organic layer was washed with H_2O (3 × 15 mL), brine (15 mL), dried over Na_2SO_4, filtered, and concentrated. Purification was carried out by an automated MPLC system using a 24 g normal phase silica column with gradient elution of 0–6% EtOAc/hexanes. Concentration of fractions afforded product as a white amorphous solid (433 mg, 0.887 mmol, 89% yield) containing a minor unidentified impurity. The intermediate was used in the next step without further purification. R_f = 0.36 (4% EtOAc/hexanes); mp 82–86 ˚C; IR (neat) 2092, 1733 cm^{-1}; ^1H NMR (400 MHz, $CDCl_3$) δ 3.54 (m, 1H), 3.33–3.20 (m, 2H), 2.41 (m, 1H), 1.82–1.16 (complex, 18H), 1.08 (m, 1H), 0.99–0.87 (complex, 16H, contains s, 0.89, 3H; s, 0.88, 9H), 0.82 (s, 3H), 0.66 (m, 1H), 0.05 (s, 6H); ^{13}C NMR (101 MHz, $CDCl_3$) δ 222.0, 72.1, 54.7, 51.5, 49.5, 48.6, 45.2, 44.4, 38.7, 37.3, 35.8, 35.2, 32.0, 31.9, 31.0, 28.6, 28.5, 27.9, 27.6, 26.1 (3C), 20.5, 18.4, 14.7, 12.5, -4.397, -4.405. HRMS (FT-ICR, APCI) *m/z*: $[M – N_2 + H]^+$ calcd for $C_{22}H_{50}N_3O_2Si$ 460.3605, found 460.3600.

3β-Hydroxy-5α-androstane-derived D-Ring Lactam, Intra1

Following a literature procedure[31], **Intra1** was prepared as described. To a solution of **S17** (48.5 mg, 0.099 mmol) in HFIP (0.5 mL) at 0 ˚C under nitrogen atmosphere was added $TiCl_4$ (1.0 M in CH_2Cl_2, 149 µL, 0.149 mmol, 1.5 equiv). The vial was capped, and the reaction mixture was stirred at room temperature for 24 h. The reaction mixture was concentrated under a stream of nitrogen. The residue was diluted with CH_2Cl_2, washed with a solution of saturated NH_4Cl (2 × 5 mL), solution of saturated $NaHCO_3$ (5 mL), brine (5 mL), dried over Na_2SO_4, filtered, and concentrated. Purification was carried out by an automated MPLC system using a 12 g normal phase silica column with gradient elution of 0–4% MeOH/CH_2Cl_2. Concentration of fractions afforded product as a white amorphous solid (28.7 mg, 0.083 mmol, 84% yield, UPLC/HRMS purity: 96.5%). R_f = 0.25 (2% MeOH/CH_2Cl_2); mp 199–209 ˚C; IR (neat) 3393, 1621 cm^{-1}; ^1H NMR (400 MHz, $CDCl_3$) δ 3.63–3.51 (m, 3H), 3.37 (m, 1H), 2.12–2.01 (m, 2H), 1.93–1.54 (complex, 9H), 1.51–1.16 (complex, 9H), 1.12–0.93 (complex, 5H, contains s, 1.03, 3H), 0.90–0.79 (complex, 4H, contains s, 0.79, 3H), 0.65 (m, 1H); ^{13}C NMR (101 MHz, $CDCl_3$) δ 176.3, 71.4, 53.9, 53.2, 44.7, 44.3, 43.3, 40.3, 38.1, 37.2, 36.9, 35.6, 35.5, 33.9, 31.6, 31.2, 28.7, 28.5, 22.1, 20.2, 15.3, 12.4. HRMS (FT-ICR, HESI) *m/z*: $[M + H]^+$ calcd for $C_{22}H_{36}NO_2$ 346.2741, found 346.2737.

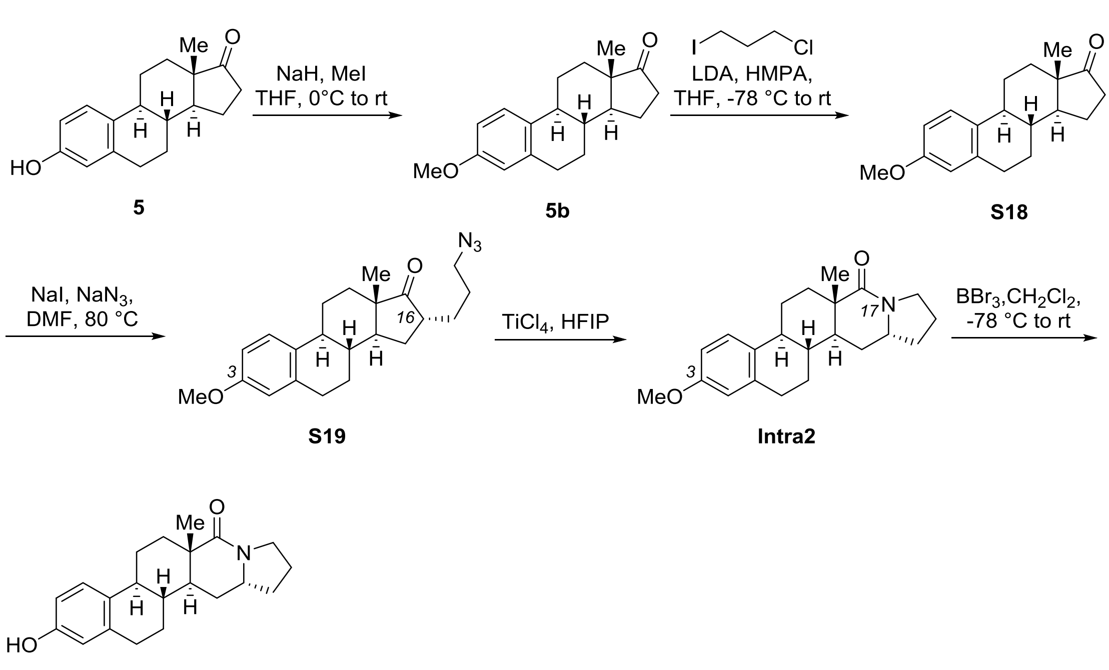

Intra3

Intramolecular Schmidt reaction of estrone 5

Intermediate **5b** was prepared following a previously published procedure.[15] Characterization data were consistent with reported data (mp 168–173 ˚C).[15,32]

16α-(3′-Chloropropyl)-3-methoxy-estrone, S18

Following a literature procedure, **S18** was prepared as described. To a cooled 1.0 M LDA solution (260 µL, 1.82 mmol, 1.2 equiv) in anhydrous THF (20.0 mL) at –78 ˚C was added mestrone **5a** (425 mg, 1.50 mmol) cautiously. The reaction mixture was allowed to –10 ˚C and stirred for 1 h. To the pale yellow solution, HMPA (1.10 mL, 6.32 mmol, 4.2 equiv) was added followed by 1-chloro-3-iodopropane (240 µL, 2.24 mmol, 1.5 equiv). The pale yellow solution was stirred at –10 ˚C for 1 h, warmed to room temperature, and stirred overnight. The reaction mixture was quenched with a solution of saturated NH_4Cl (40 mL) and H_2O (20 mL). The aqueous layer was extracted with Et_2O (3 × 30 mL). The combined organic layer was washed with brine (15 mL), dried over Na_2SO_4, filtered, and concentrated. Purification was carried out by an automated MPLC system using a 24 g normal phase silica column with gradient elution of 0–10% Et_2O/hexanes. Fractions containing mixtures of **5a** and **S18** were subjected to a second automated purification using the same gradient. Concentration of appropriate fractions afforded desired product as a yellow oil/solid (426 mg, 1.17 mmol, 78% yield) containing a minor unidentified impurity. The intermediate was used in the next step without further purification. R_f = 0.40 (10% EtOAc/hexanes); IR (neat) 1730 cm^{-1}; ^1H NMR (400 MHz, $CDCl_3$) δ 7.20 (m, 1H), 6.73 (dd, *J* = 8.6, 2.8 Hz, 1H), 6.65 (d, *J* = 2.8 Hz, 1H), 3.78 (s, 3H), 3.61–3.46 (m, 2H), 2.92–2.89 (m, 2H), 2.49 (m, 1H), 2.39 (m, 1H), 2.25 (m, 1H), 2.01–1.82 (complex, 5H), 1.79–1.71 (m, 1H), 1.66–1.38 (complex, 7H), 0.95 (s, 3H). **Note**: ^1H NMR signals confirmed by HSQC correlation. HSQC has been included in the spectra section. ^{13}C NMR (101 MHz, $CDCl_3$) δ 221.6, 157.7, 137.9, 132.1, 126.5, 114.0, 111.7, 55.4, 48.8, 48.4, 44.9, 44.3, 44.1, 38.5, 31.8, 31.4, 30.0, 28.8, 27.8, 26.6, 26.0, 14.8. HRMS (FT-ICR, APCI) *m/z*: $[M + H]^+$ $C_{22}H_{30}ClO_2$ calcd for $[M + H]^+$ 361.1929, found 361.1929.

16α-(3′-Azidopropyl)-3-methoxy-estrone, S19

Following a literature procedure[31], **S19** was prepared as described. A mixture of **S18** (419 mg, 1.16 mmol), NaI (348 mg, 2.32 mmol, 2.0 equiv), NaN_3 (226 mg, 3.48 mmol, 3.0 equiv) in anhydrous DMF (12.0 mL, 1.0 M) was stirred at 80 ˚C overnight. The reaction mixture was quenched with H_2O (50 mL). The aqueous layer was extracted with Et_2O (3 × 30 mL). The combined organic layer was washed with H_2O (3 × 15 mL), brine (15 mL), dried over Na_2SO_4, filtered, and concentrated. Purification was carried out by an automated MPLC system using a 24 g normal phase silica column with gradient elution of 0–

10% EtOAc/hexanes. Concentration of fractions afforded desired product as a white oil (391 mg, 1.06 mmol, 92% yield) containing a minor unidentified impurity. The intermediate was used in the next step without further purification. R_f = 0.29 (10% EtOAc/hexanes); IR (neat) 2091, 1733 cm^{-1}; ^{1}H NMR (400 MHz, $CDCl_3$) δ 7.20 (d, *J* = 8.6 Hz, 1H), 6.72 (dd, *J* = 8.6, 2.8 Hz, 1H), 6.65 (d, *J* = 2.7 Hz, 1H), 3.78 (s, 3H), 3.36–3.24 (m, 2H), 2.92–2.89 (m, 2H), 2.49 (m, 1H), 2.39 (m, 1H), 2.25 (m, 1H), 2.02–1.32 (complex, 13H), 0.95 (s, 3H). **Note**: ^{1}H NMR signals confirmed by HSQC correlation. NOE (1D) and HSQC has been included in the spectra section. ^{13}C NMR (101 MHz, $CDCl_3$) δ 221.6, 157.7, 137.9, 132.1, 126.5, 114.0, 111.7, 55.4, 51.5, 48.8, 48.4, 44.5, 44.1, 38.5, 31.8, 29.8, 28.5, 27.72, 27.68, 26.6, 26.0, 14.7. HRMS (FT-ICR, HESI) *m/z*: $[M – N_2 + H]^+$ calcd for $C_{22}H_{30}N_3O_2$ 340.2271, found 340.2267.

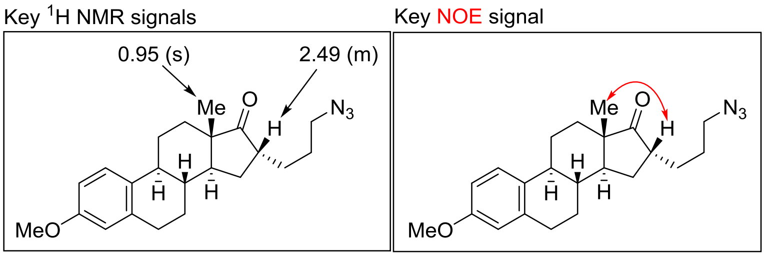

3-Methoxy-1,3,5-estratriene-derived D-Ring Lactam, Intra2

Following a literature procedure[31], **Intra2** was prepared as described. To a solution of **S19** (95.7 mg, 0.260 mmol) in HFIP (2.0 mL) at 0 ˚C under nitrogen atmosphere was added $TiCl_4$ (1.0 M in CH_2Cl_2, 130 µL, 0.130 mmol, 0.5 equiv). The vial was capped, and the reaction mixture was stirred at room temperature for 24 h. The reaction mixture was concentrated under nitrogen. The residue was diluted with CH_2Cl_2, washed with a solution of saturated NH_4Cl (2 × 8 mL), solution of saturated $NaHCO_3$ (8 mL), brine (8 mL), dried over Na_2SO_4, filtered, and concentrated. Purification was carried out by an automated MPLC system using a 12 g normal phase silica column with gradient elution of 0–70% EtOAc/hexanes. Concentration of fractions afforded product as a white amorphous solid (61.3 mg, 0.181 mmol, 69% yield, UPLC/HRMS purity: 97.7%). R_f = 0.25 (50% EtOAc/hexanes); mp 144–149 ˚C; IR (neat) 1612 cm^{-1}; ^{1}H NMR (400 MHz, $CDCl_3$) δ 7.24 (d, *J* = 8.7 Hz, 1H), 6.73 (dd, *J* = 8.6, 2.8 Hz, 1H), 6.62 (d, *J* = 2.7 Hz, 1H), 3.78 (s, 3H), 3.61 (m, 2H), 3.41 (m, 1H), 2.85 (m, 2H), 2.37 (m, 1H), 2.27–2.11 (m, 2H), 2.05–1.89 (m, 3H), 1.81–1.68 (m, 3H), 1.58–1.24 (complex, 6H), 1.08 (s, 3H); ^{13}C NMR (101 MHz, $CDCl_3$) δ 176.3, 157.7, 137.7, 132.4, 126.6, 113.7, 111.8, 55.4, 54.0, 44.9, 42.7, 42.0, 40.7, 40.4, 35.6, 33.9, 30.2, 28.4, 26.6, 26.0, 22.2, 15.2. HRMS (FT-ICR, HESI) *m/z*: $[M + H]^+$ calcd for $C_{22}H_{30}NO_2$ 340.2271, found 340.2269.

3-Hydroxy-1,3,5-estratriene-derived D-Ring Lactam, Intra3

To a solution of **Intra2** (51.5 mg, 0.152 mmol) in anhydrous CH_2Cl_2 (5.0 mL) at −78 ˚C was added BBr_3 (1.0 M in CH_2Cl_2, 1.20 mL, 1.20 mmol, 8.0 equiv). The reaction mixture was stirred at −78 ˚C for 1 h, warmed to room temperature over 4 h, and continued stirring at room temperature for 1 h (pinkish-orange suspension). The reaction mixture was quenched with two drops of water and MeOH (2 mL). The solvent was removed under nitrogen, the residue was redissolved in MeOH, and loaded on silica gel for purification. Purification was carried out by an automated MPLC system using a 12 g normal phase silica column with gradient elution of 0–3% $MeOH/CH_2Cl_2$. Concentration of fractions afforded **Intra3** as a white amorphous solid (38.1 mg, 0.117 mmol, 77% yield, UPLC/HRMS purity: ≥99.5%). R_f = 0.20 (2% $MeOH/CH_2Cl_2$); mp 283–300 ˚C (decomposed); IR (neat) 3303, 1621, 1606 cm^{-1}; ^{1}H NMR (600 MHz, DMSO-d_6) δ 8.99 (s, 1H), 7.07 (d, *J* = 8.5 Hz, 1H), 6.51 (d, *J* = 9.4 Hz, 1H), 6.43 (m, 1H), 3.59 (m, 1H), 3.40 (m, 1H), 3.20 (m, 1H), 2.70 (m, 2H), 2.27 (m, 1H), 2.09 (m, 2H), 1.93 (m, 3H), 1.81 (m, 1H), 1.67 (m, 2H), 1.56–1.37 (m, 2H), 1.29–1.18 (m, 4H), 0.98 (s, 3H); ^{13}C NMR (151 MHz, DMSO-d_6) δ 174.6, 155.0, 137.0, 130.1, 126.1, 114.6, 112.8, 53.1, 44.1, 42.1, 41.3, 40.0934, 40.0933, 34.8, 33.9, 29.4, 27.5, 25.9, 25.6, 21.5, 14.9. HRMS (FT-ICR, HESI) *m/z*: $[M + H]^+$ calcd for $C_{21}H_{28}NO_2$ 326.2115, found 326.2111.

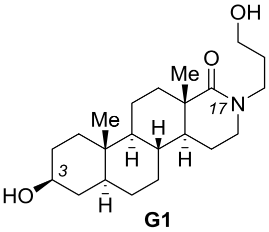

G1

3β-Hydroxy-5α-androstane-derived D-Ring Lactam, G1

Following the general procedure E, 3-azidopropanol **6** (30.3 mg, 0.300 mmol, 2.0 equiv) was reacted with *trans*-androsterone **4** (43.6 mg, 0.150 mmol) to give **G1** as a white amorphous solid (51.0 mg, 0.141 mmol, 94% yield, UPLC/HRMS purity: ≥99.5%). Purification was carried out by an automated MPLC system using a 12 g normal phase silica column with gradient elution from 0–5% MeOH/CH_2Cl_2. R_f = 0.35 (5% MeOH/CH_2Cl_2); mp 177–181 ˚C; IR (neat) 3237, 1625 cm^{-1}; ^1H NMR (400 MHz, $CDCl_3$) δ 3.69–3.55 (m, 2H), 3.49–3.39 (m, 2H), 3.32–3.17 (m, 3H), 2.60 (br s, 2H), 2.19 (m, 1H), 1.94 (m, 1H), 1.89–1.22 (complex, 17H), 1.13–1.06 (m, 4H, contains s, 1.12, 3H), 1.00–0.93 (m, 1H), 0.91–0.62 (m, 1H), 0.80 (s, 3H), 0.68 (m, 1H); ^{13}C NMR (101 MHz, $CDCl_3$) δ 178.3, 71.3, 57.9, 53.4, 47.1, 45.9, 44.4, 43.0, 41.3, 38.1, 36.8, 35.7, 34.8, 34.6, 31.6, 31.0, 29.3, 28.6, 21.0, 20.3, 18.6, 12.4. **Note**: APT, HSQC and HMBC are included in the spectra section. X-ray crystal structure of this analog is provided in the CCDC (CCDC 1583536). HRMS (FT-ICR, HESI) *m/z*: [M + H]$^+$ calcd for $C_{22}H_{38}NO_3$ 364.2846, found 364.2840.

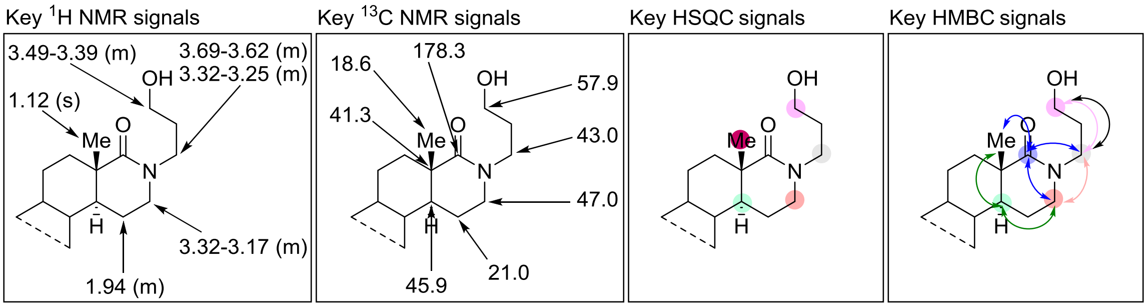

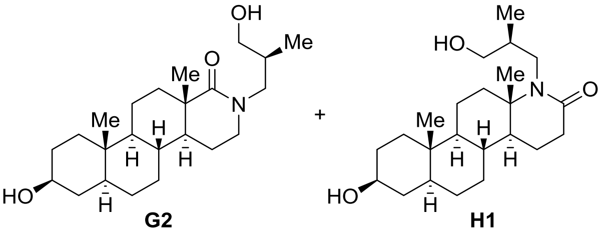

G2 + **H1**

3β-Hydroxy-5α-androstane-derived D-Ring Lactams, G2 and H1

Following the general procedure E, (*S*)-3-azido-2-methylpropanol (*S*)-**8** (34.6 mg, 0.300 mmol, 2.0 equiv) was reacted with *trans*-androsterone **4** (43.7 mg, 0.151 mmol) to give **G2** as a white amorphous solid to afford a separable mixture of two regioisomeric lactams **G2** (30.3 mg, 0.0803 mmol, 53% yield, UPLC/HRMS purity: 98.7%) and **H1** (19.9 mg, 0.053 mmol, 35%, UPLC/HRMS purity: ≥99.5%). A 56:44 regioisomeric ratio was observed by ^1H NMR of crude reaction mixture. Purification was carried out by an automated MPLC system using a 12 g normal phase silica column with gradient elution from 0–20% EtOAc/Ether. Concentration of fractions afforded slightly impure **G2** and **H1**. A second purification of separated regioisomers using a 4 g column with gradient elution from 0–5% MeOH/DCM afforded pure regioisomers. **G2:** R_f = 0.26 (50% EtOAc/Ether); IR (neat) 3440, 3283, 1632 cm^{-1}; mp 174–177 ˚C; ^1H NMR (400 MHz, $CDCl_3$) δ 3.65–3.53 (m, 2H), 3.35 (dd, *J* = 11.9, 3.3 Hz, 1H), 3.27 (m, 3H), 2.90 (dd, *J* = 13.9, 4.3 Hz), 2.16 (m, 1H), 1.93–1.51 (complex, 7H), 1.43–1.19 (complex. 9H), 1.10–1.03 (m, 4H, contains s, 1.10, 3H), 0.98–0.80 (m, 5H, contains d, *J* = 7.0 Hz, 3H), 0.78 (s, 3H), 0.66 (m, 1H); ^{13}C NMR (126 MHz, $CDCl_3$) δ 178.3, 71.2, 63.1, 53.3, 49.7, 48.6, 46.2,

44.4, 41.4, 38.0, 36.8, 35.7, 34.85, 34.79, 33.7, 31.5, 30.9, 28.6, 21.0, 20.3, 18.5, 15.3, 12.3. HRMS (FT-ICR, HESI) *m/z*: $[M + H]^+$ calcd for $C_{23}H_{40}NO_3$ 378.3003, found 378.2991. **H1:** R_f = 0.11 (50% EtOAc/Ether); IR (neat) 3443, 3309, 1603 cm^{-1}; mp 196–198 ˚C; ^1H NMR (400 MHz, $CDCl_3$) δ 3.83 (dd, *J* = 14.8, 11.4 Hz, 1H), 3.58 (tt, *J* = 11.0, 4.8 Hz, 1H), 3.48–3.44 (m, 1H), 3.33 (dd, *J* = 11.9, 2.9 Hz, 1H) 3.03 (dd, *J* = 14.7, 4.6 Hz, 1H), 2.58–2.42 (m, 2H), 1.95 (m, 1H), 1.89 (m, 1H), 1.83–1.70 (complex, 4H), 1.58 (m, 1H), 1.52 (td, *J* = 12.8, 3.9 Hz, 1H), 1.41–1.18 (complex, 8H), 1.17 (s, 3H), 1.14–1.04 (m, 1H), 1.00–0.94 (m, 4H, contains d, *J* = 6.9 Hz, 3H), 0.91–0.80 (m, 1H), 0.76–0.71 (m, 4H, contains s, 0.76, 3H); ^{13}C NMR (126 MHz, $CDCl_3$) δ 172.3, 71.1, 62.7, 59.1, 52.7, 48.4, 44.2, 42.7, 37.9, 37.8, 37.5, 36.8, 36.1, 35.5, 31.4, 30.8, 30.2, 28.5, 21.8, 21.6, 20.1, 15.6, 12.2. HRMS (FT-ICR, HESI) *m/z*: $[M + H]^+$ calcd for $C_{23}H_{40}NO_3$ 378.3003, found 378.2986.

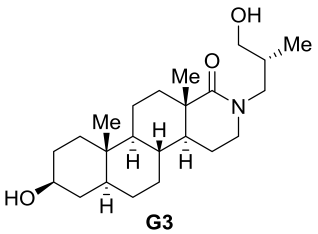

G3

3β-Hydroxy-5α-androstane-derived D-Ring Lactam, G3

Following the general procedure E, (*R*)-3-azido-2-methylpropanol (*R*)-**8** (34.8 mg, 0.302 mmol, 2.0 equiv) was reacted with *trans*-androsterone **4** (43.9 mg, 0.151 mmol) to give **G3** as a white amorphous solid (53.2 mg, 0.141 mmol, 93% yield, UPLC/HRMS purity: ≥99.5%). Purification was carried out by an automated MPLC system using a 12 g normal phase silica column with gradient elution from 0–5% MeOH/CH_2Cl_2. R_f = 0.33 (5% MeOH/CH_2Cl_2); mp 168–171 ˚C; IR (neat) 3380, 1614 cm^{-1}; ^1H NMR (400 MHz, $CDCl_3$) δ 3.89 (dd, *J* = 13.8, 11.0 Hz, 1H), 3.58 (tt, *J* = 10.7, 4.8 Hz, 1H), 3.41–3.27 (m, 3H), 3.18 (m, 1H), 2.77 (br s, 2H), 2.53 (dd, *J* = 13.9, 4.3 Hz, 1H), 2.18 (m, 1H), 1.93 (m, 1H), 1.88–1.18 (complex, 15H), 1.11–1.05 (m, 4H, contains s, 1.11, 3H), 1.00–0.96 (m, 4H, contains d, *J* = 7.0 Hz, 3H), 0.94–0.80 (m, 1H), 0.79 (s, 3H), 0.67 (m, 1H); ^{13}C NMR (101 MHz, $CDCl_3$) δ 178.2, 71.1, 63.1, 53.3, 49.1, 47.7, 45.5, 44.4, 41.3, 38.0, 36.8, 35.6, 34.7, 34.4, 33.5, 31.4, 30.9, 28.8, 21.0, 30.2, 18.5, 15.4, 12.3. HRMS (FT-ICR, HESI) *m/z*: $[M + H]^+$ calcd for $C_{23}H_{41}NO_3$ 378.3008 found 378.2995.

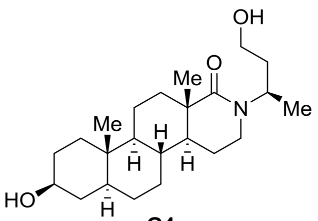

G4

3β-Hydroxy-5α-androstane-derived D-Ring Lactam, G4

Following the general procedure E, (*R*)-3-azidobutanol (*R*)-**10** (34.4 mg, 0.299 mmol, 2.0 equiv) was reacted with *trans*-androsterone **4** (44.4 mg, 0.153 mmol) to give **G4** as a white amorphous solid (52.9 mg, 0.140 mmol, 92% yield, UPLC/HRMS purity: 97.4%). Purification was carried out by an automated MPLC system using a 12 g normal phase silica column with gradient elution from 0–4% MeOH/CH_2Cl_2. R_f = 0.31 (5% MeOH/CH_2Cl_2); mp 214–217 ˚C; IR (neat) 3380, 3291, 1596 cm^{-1}; ^1H NMR (400 MHz, $CDCl_3$) δ 4.87 (m, 1H), 3.59 (tt, *J* = 10.7, 4.8, 1H), 3.51 (ddd, *J* = 12.1, 5.0, 2.4 Hz, 1H), 3.27 (td, *J* = 11.8, 2.6, 1H), 3.19–3.03 (m, 2H), 2.59 (br s, 2H), 2.19 (m, 1H), 1.96 (m, 1H), 1.89–1.22 (complex, 16H), 1.18 (dd, *J* = 7.1 Hz, 3H), 1.13–1.05 (m, 1H), 0.97 (m, 1H), 0.91–0.82 (m, 1H), 0.80 (s, 3H), 0.68 (m, 1H); ^{13}C NMR (101 MHz, $CDCl_3$) δ 178.4, 71.3, 58.6, 53.5, 45.6, 44.5, 44.3, 41.9, 40.2, 38.1, 36.8, 36.2, 35.7, 34.7, 34.6, 31.6, 31.0, 28.6, 20.6, 20.3, 18.8, 18.4, 12.4. **Note**: APT, NOE (1D), HSQC and HMBC are included in the spectra section. HRMS (FT-ICR, HESI) *m/z*: $[M + H]^+$ calcd for $C_{23}H_{40}NO_3$ 378.3003 found 378.2995.

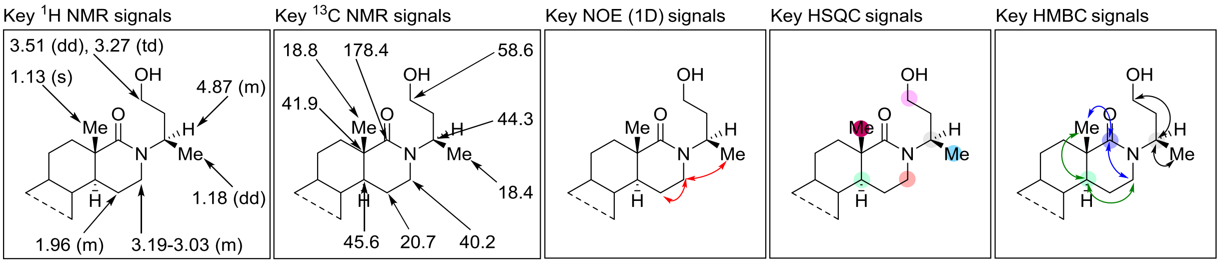

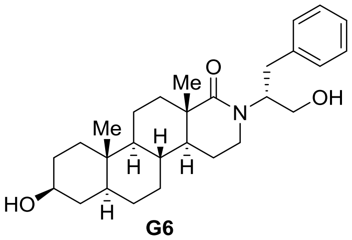
G6

3β-Hydroxy-5α-androstane-derived-derived D-Ring Lactam, G6

Following the general procedure E, (*R*)-2-azido-3-phenylpropanol (*R*)-**14** (53.2 mg, 0.300 mmol, 2.0 equiv) was reacted with *trans*-androsterone **4** (43.7 mg, 0.150 mmol) to give **G6** as a white amorphous solid (54.0 mg, 0.123 mmol, 82% yield, UPLC/HRMS purity: 97.2%) containing a minor uncharacterized regioisomer (regioisomer ratio 89:11). Purification was carried out by an automated MPLC system using a 12 g normal phase silica column with gradient elution from 0–5% MeOH/CH_2Cl_2 over 40 min. R_f = 0.30 (80% EtOAc/hexanes); mp 109–118 ˚C; IR (neat) 3426, 3280, 1603 cm^{-1}; key ^{1}H NMR (400 MHz, $CDCl_3$) δ 7.31–7.24 (m, 2H), 7.22–7.18 (m, 3H), 4.06 (m, 1H), 3.95 (m, 1H), 3.83–3.71 (m, 2H), 3.61–3.53 (m, 2H), 3.13–3.05 (m, 2H), 2.96–2.89 (m, 2H), 2.12 (m, 1H); ^{13}C NMR (101 MHz, $CDCl_3$) δ 178.8, 138.7, 129.2 (2C), 128.5 (2C), 126.5, 71.3, 64.1, 53.3, 46.9, 45.1, 44.4, 41.7, 38.1, 36.8, 35.7, 34.79, 34.78, 33.9, 31.6, 30.9, 29.8, 28.6, 21.2, 20.3, 18.1, 12.3. **Note**: ^{1}H and ^{13}C NMR spectra of mixture are included in the spectra section. Characterization above only denote peaks of the major regioisomer. HRMS (FT-ICR, HESI) *m/z*: $[M + H]^+$ calcd for $C_{28}H_{42}NO_3$ 440.3159, 440.3139.

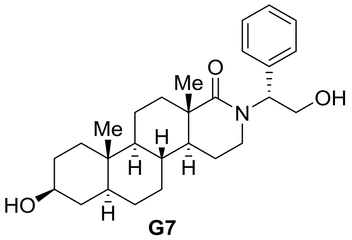
G7

3β-Hydroxy-5α-androstane-derived D-Ring Lactam, G7

Following the general procedure E, (*R*)-2-azido-2-phenylethanol (*R*)-**15** (40.8 mg, 0.250 mmol, 2.0 equiv) was reacted with *trans*-androsterone **4** (36.2 mg, 0.125 mmol) to give **G7** as a yellow amorphous solid (27.0 mg, 0.0634 mmol, 51% yield, UPLC/HRMS purity: 98.9%). Purification was carried out by an automated MPLC system using a 12 g normal phase silica column with gradient elution from 50–100% EtOAc/hexanes. R_f = 0.36 (4% MeOH/CH_2Cl_2); IR (neat) 3364, 1601 cm^{-1}; mp 212–214 ˚C; ^{1}H NMR (400 MHz, $CDCl_3$) δ 7.35–7.19 (m, 5H), 5.66 (t, *J* = 7.1 Hz, 1H), 4.10 (d, *J* = 7.1 Hz, 2H), 3.57 (m, 1H), 3.21 (ddd, *J* = 12.4, 7.3, 2.0 Hz, 1H), 2.82 (ddd, *J* = 12.4, 10.6, 7.1 Hz, 1H), 2.21 (m, 1H), 1.87–1.18 (complex, 17H), 1.15 (s, 3H), 1.10–0.91 (m, 2H), 0.85–0.74 (m, 4H, contains s, 0.78, 3H), 0.67 (m, 1H); ^{13}C NMR (126 MHz, $CDCl_3$) δ 179.0, 137.0, 128.7 (2C), 128.0 (2C), 127.8, 71.2, 62.1, 59.0, 53.2, 45.1, 44.4, 42.9, 41.9, 38.0, 36.8, 35.6, 34.91, 34.86, 31.4, 30.8, 28.5, 21.0, 20.3, 18.3, 12.3. HRMS (FT-ICR, HESI) *m/z*: $[M + H]^+$ calcd for $C_{27}H_{40}NO_3$ 426.3003, found 426.2985.

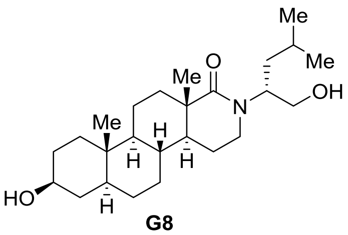

G8

3β-Hydroxy-5α-androstane-derived D-Ring Lactam, G8

Following the general procedure E, (*R*)-2-azido-4-methylpentanol (*R*)-**16** (43.2 mg, 0.302 mmol, 2.0 equiv) was reacted with trans-androsterone **4** (43.9 mg, 0.151 mmol) to give **G8** as a white amorphous solid (51.0 mg, 0.126 mmol, 83% yield, UPLC/HRMS purity: 92.4%). Purification was carried out by an automated MPLC system using a 12 g normal phase silica column with gradient elution from 0–5% MeOH/CH_2Cl_2 over 50 min. R_f = 0.27 (5% MeOH/CH_2Cl_2); IR (neat) 3364, 1611 cm^{-1}; mp 97–115 ˚C; key ^{1}H NMR (400 MHz, $CDCl_3$) δ 4.30 (m, 1H), 3.67 (dd, *J* = 11.5, 3.8 Hz, 1H), 3.64–3.55 (m, 2H), 2.16 (m, 1H), 1.95 (m, 1H); ^{13}C NMR (101 MHz, $CDCl_3$) δ 179.0, 71.3, 64.1, 55.8, 53.4, 45.4, 44.5, 43.3, 41.8, 38.1, 36.9, 35.7, 34.97, 34.95, 31.6, 31.0, 28.6, 25.1, 23.3, 22.5, 21.2, 20.4, 18.2, 12.4. **Note**: Missing one carbon signal due to signal overlap; ^{1}H and ^{13}C NMR spectra of mixture are included in the spectra section. Characterization above only denote peaks of the major regioisomer. HRMS (FT-ICR, HESI) *m/z*: $[M + H]^+$ calcd for $C_{25}H_{45}NO_3$ 406.3316, found 406.3298.

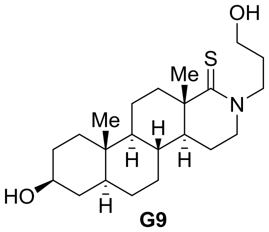

G9

3β-Hydroxy-5α-androstane-derived D-Ring Thioamide, G9

Following the general procedure G, **G9** was prepared as a white amorphous solid (49.1 mg, 0.129 mmol, 52% yield, UPLC/HRMS purity: 98.6%). Purification was carried out by an automated MPLC system using a 12 g normal phase silica column with gradient elution from 0–1.5% MeOH/CH_2Cl_2. R_f = 0.33 (5% MeOH/CH_2Cl_2); mp 212–216 ˚C; IR (neat) 3241, 1519 cm^{-1}; ^{1}H NMR (400 MHz, $CDCl_3$) δ 4.46 (dt, *J* = 13.3, 6.6 Hz, 1H), 3.88 (dt, *J* = 13.4, 5.9 Hz, 1H), 3.62–3.47 (m, 3H), 3.44–3.31 (m, 2H), 2.74 (m, 1H), 2.05 (m, 1H), 1.90–1.53 (complex, 7H), 1.46–1.22 (complex, 9H), 1.18 (s, 3H), 1.09 (m, 1H), 0.97 (m, 1H), 0.85 (m, 1H), 0.79 (s, 3H), 0.65 (m, 1H); ^{13}C NMR (101 MHz, $CDCl_3$) δ 211.7, 71.3, 58.1, 53.0, 51.7, 50.6, 46.4, 44.6, 44.4, 39.7, 38.0, 36.8, 36.0, 35.6, 31.6, 31.0, 29.6, 28.6, 21.3, 21.2, 12.3. **Note**: Missing one carbon signal due to signal overlap. HRMS (FT-ICR, HESI) *m/z*: $[M + H]^+$ calcd for $C_{22}H_{38}NO_2S$ 380.2618, found 380.2612.

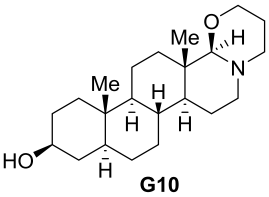

G10

3β-Hydroxy-5α-androstane-derived D-Ring Oxazinane, G10

Following the general procedure G, **G10** was prepared as a white amorphous solid (59.4 mg, 0.171 mmol, 85% yield, LCMS Purity: ≥99.5%). Purification was carried out by an automated MPLC system using a 12 g normal phase silica column with gradient elution from 0–10% MeOH (0.5% NH_4OH)/CH_2Cl_2. R_f = 0.25 (5% MeOH (0.5% NH_4OH)/CH_2Cl_2); mp 178–184 ˚C; IR (neat) 3350, 2929, 2848 cm^{-1}; ^{1}H NMR (400 MHz, $CDCl_3$) δ 4.00 (m, 1H), 3.58 (m, 1H), 3.38 (m, 1H), 2.96–2.81 (m, 3H),

2.11–1.94 (m, 3H), 1.83–1.71 (m, 4H), 1.61–1.18 (complex, 11H), 1.10–1.01 (m, 1H), 0.99–0.88 (m, 5H, contains s, 0.95, 3H), 0.83–0.72 (m, 5H, contains s, 0.78, 3H), 0.65 (m, 1H); ^{13}C NMR (101 MHz, $CDCl_3$) δ 102.3, 71.4, 67.9, 54.8, 54.6, 53.9, 49.4, 44.6, 38.3, 37.9, 37.0, 35.9, 35.7, 34.5, 31.6, 31.0, 28.7, 25.9, 23.7, 20.2, 13.6, 12.4. HRMS (FT-ICR, HESI) *m/z*: $[M + H]^+$ calcd for $C_{22}H_{38}NO_2$ 348.2897, found 348.2890. **Note**: X-ray crystal structure of this analog is provided in the CCDC (CCDC 1583518).

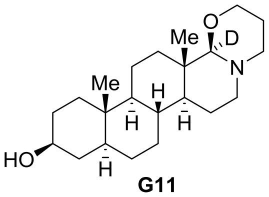

G11

3β-Hydroxy-5α-androstane-derived D-Ring Oxazinane, G11

Following the general procedure G, **G11** was prepared as a white amorphous solid (45.0 mg, 0.128 mmol, 64% yield, LCMS Purity: ≥99.5%). Purification was carried out by an automated MPLC system using a 12 g normal phase silica column with gradient elution from 0–10% MeOH (0.5% NH_4OH)/CH_2Cl_2. R_f = 0.25 (5% MeOH (0.5% NH_4OH)/CH_2Cl_2); mp 178–184 °C; IR (neat) 3519, 3243, 2930, 2847 cm^{-1}; ^{1}H NMR (400 MHz, $CDCl_3$) δ 4.01 (m, 1H), 3.58 (m, 1H), 3.38 (m, 1H), 2.96–2.82 (m, 2H), 2.13–1.95 (m, 3H), 1.83–1.71 (m, 3H), 1.58–1.18 (complex, 12H), 1.10–1.03 (m, 1H), 0.99–0.91 (m, 5H, contains s, 0.96, 3H), 0.84–0.72 (m, 5H, contains s, 0.78, 3H), 0.65 (m, 1H); ^{13}C NMR (101 MHz, $CDCl_3$) δ 71.4, 67.5, 54.7, 54.5, 53.8, 49.3, 44.5, 38.2, 38.0, 36.9, 35.83, 35.80, 35.7, 34.4, 31.6, 31.0, 28.7, 25.6, 23.4, 20.1, 13.4, 12.4. HRMS (FT-ICR, HESI) *m/z*: $[M + H]^+$ calcd for $C_{22}H_{37}DNO_2$ 349.2960, found 349.2952.

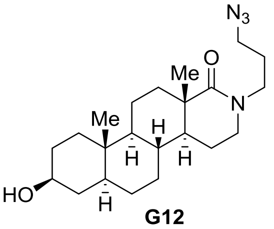

G12

3β-Hydroxy-5α-androstane-derived D-Ring Lactam, G12

Following the general procedure G, **G12** was prepared as an off-white amorphous solid (166 mg, 0.429 mmol, 86% yield, UPLC/HRMS purity: ≥99.5%). Purification was carried out by an automated MPLC system using a 12 g normal phase silica column with gradient elution from 0–75% EtOAc/hexanes. R_f = 0.25 (50% EtOAc/hexanes); mp 135–139 °C; IR (neat) 3469, 3330, 2092, 1607 cm^{-1}; ^{1}H NMR (400 MHz, $CDCl_3$) δ 3.59 (m, 1H), 3.49 (m, 1H), 3.35–3.18 (complex, 5H), 2.18 (m, 1H), 1.95–1.72 (complex, 6H), 1.66–1.49 (m, 3H), 1.45–1.19 (complex, 9H), 1.13–1.05 (m, 4H, contains s, 1.09, 3H), 0.97 (m, 1H), 0.91–0.82 (m, 1H), 0.80 (s, 3H); ^{13}C NMR (101 MHz, $CDCl_3$) δ 176.8, 71.3, 53.4, 49.5, 47.6, 46.0, 44.9, 44.5, 41.2, 38.1, 36.9, 35.7, 34.9, 34.6, 31.9, 31.0, 28.6, 36.7, 21.2, 20.3. 18.4, 12.4. HRMS (FT-ICR, HESI) *m/z*: $[M + H]^+$ calcd for $C_{22}H_{37}N_4O_2$ 389.2911, found 389.2904.

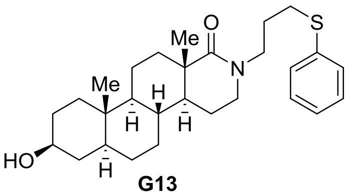

G13

3β-Hydroxy-5α-androstane-derived D-Ring Lactam, G13

Following the general procedure G, **G13** was prepared as a white amorphous solid (48.7 mg, 0.107 mmol, 71% yield, UPLC/HRMS purity: 98.6%). Purification was carried out by an automated MPLC

system using a 12 g normal phase silica column with gradient elution from 0–50% EtOAc/hexanes. R_f = 0.48 (50% EtOAc/hexanes); mp 133–136 ˚C; IR (neat) 3367, 1617 cm^{-1}; ^{1}H NMR (400 MHz, $CDCl_3$) δ 7.36–7.25 (m, 4H), 7.17 (m, 1H), 3.63–3.48 (m, 2H), 3.30–3.15 (m, 3H), 2.29–2.86 (m, 2H), 2.18 (m, 1H), 1.92–1.21 (complex, 18H), 1.12–1.05 (m, 4H, contains s, 1.08, 3H), 1.00–0.91 (m, 1H), 0.89–0.80 (m, 1H), 0.79 (s, 3H) 0.66 (m, 1H); ^{13}C NMR (101 MHz, $CDCl_3$) δ 176.7, 136.6, 129.3 (2C), 129.0 (2C), 126.1, 71.3, 53.4, 47.3, 46.2, 46.0, 44.5, 41.2, 38.1, 36.9, 35.7, 34.9, 34.6, 31.6, 31.3, 31.0, 28.6, 27.0, 21.2, 20.3, 18.4, 12.4. HRMS (FT-ICR, HESI) *m/z*: $[M + H]^+$ calcd for $C_{28}H_{42}NO_2S$ 456.2931, found 456.2923.

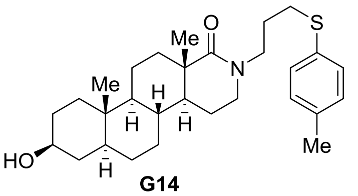

G14

3β-Hydroxy-5α-androstane-derived D-Ring Lactam, G14

Following the general procedure G, **G14** was prepared as a white amorphous solid (53.1 mg, 0.113 mmol, 76% yield, UPLC/HRMS purity: 99.0%). Purification was carried out by an automated MPLC system using a 12 g normal phase silica column with gradient elution from 0–60% EtOAc/hexanes. R_f = 0.24 (50% EtOAc/hexanes); mp 171–175 ˚C; IR (neat) 3413, 1614 cm^{-1}; ^{1}H NMR (400 MHz, $CDCl_3$) δ 7.24 (m, 3H), 7.09 (m, 2H), 3.59 (m, 1H), 3.51 (m, 1H), 3.29–3.14 (m, 3H), 2.85 (m, 2H), 2.31 (s, 3H), 2.17 (m, 1H), 1.92–1.21 (complex, 18H), 1.12–1.05 (m, 4H, contains s, 1.07, 3H), 1.02–0.93 (m, 1H), 0.89–0.82 (m, 1H), 0.79 (s, 3H), 0.66 (m, 1H); ^{13}C NMR (101 MHz, $CDCl_3$) δ 176.7, 136.3, 132.7, 130.2 (2C), 129.8 (2C), 71.3, 53.4, 47.3, 46.2, 45.9, 44.5, 41.2, 38.1, 36.9, 35.7, 34.9, 34.6, 32.0, 31.6, 31.0, 28.6, 27.0, 21.2, 21.1, 20.3, 18.5, 12.4. HRMS (FT-ICR, HESI) *m/z*: $[M + H]^+$ calcd for $C_{29}H_{44}NO_2S$ 470.3087, found 470.3078.

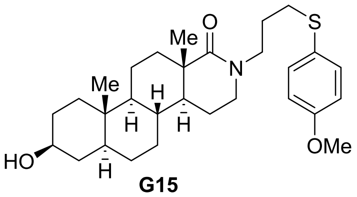

G15

3β-Hydroxy-5α-androstane-derived D-Ring Lactam, G15

Following the general procedure G, **G15** was prepared as a white amorphous solid (47.4 mg, 0.0976 mmol, 65% yield, UPLC/HRMS purity: 98.5%). Purification was carried out by an automated MPLC system using a 12 g normal phase silica column with gradient elution from 0–55% EtOAc/hexanes. R_f = 0.27 (50% EtOAc/hexanes); mp 152–155 ˚C; IR (neat) 3402, 1612 cm^{-1}; ^{1}H NMR (400 MHz, $CDCl_3$) δ 7.33 (m, 2H), 6.84 (m, 2H), 3.79 (s, 3H), 3.59 (m, 1H), 3.49 (m, 1H), 3.28–3.13 (m, 3H), 2.78 (m, 2H), 2.17 (m, 1H), 1.91–1.21 (complex, 18H), 1.12–1.04 (m, 4H, contains s, 1.07, 3H), 1.00–0.93 (m, 1H), 0.90–0.80 (m, 1H), 0.79 (s, 3H), 0.66 (m, 1H); ^{13}C NMR (101 MHz, $CDCl_3$) δ 176.6, 159.1, 133.3 (2C), 126.6, 114.7 (2C), 71.3, 55.5, 53.2, 47.3, 46.1, 45.9, 44.5, 41.2, 38.1, 36.8, 35.7, 34.8, 34.6, 33.5, 31.6, 31.0, 28.6, 27.0, 21.2, 20.3, 18.4, 12.3. HRMS (FT-ICR, HESI) *m/z*: $[M + H]^+$ calcd 486.3036, found 486.3028.

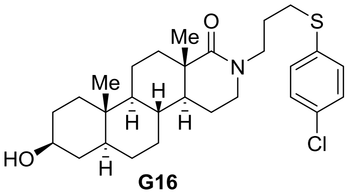

G16

3β-Hydroxy-5α-androstane-derived D-Ring Lactam, G16

Following the general procedure G, **G16** was prepared as a white amorphous solid (51.0 mg, 0.104 mmol, 69% yield, UPLC/HRMS purity: 96.6%). Purification was carried out by an automated MPLC system using a 12 g normal phase silica column with gradient elution from 0–65% EtOAc/hexanes. R_f = 0.28 (50% EtOAc/hexanes); mp 188–196 ˚C; IR (neat) 3393, 1613 cm^{-1}; ^1H NMR (400 MHz, $CDCl_3$) δ 7.24 (m, 4H), 3.63–3.48 (m, 2H), 3.29–3.15 (m, 3H), 2.89 (m, 2H), 2.17 (m, 1H), 1.92–1.21 (complex, 18H), 1.12–1.05 (m, 4H, contains s, 1.07, 3H), 0.96 (m, 1H), 0.89–0.80 (m, 1H), 0.79 (s, 3H), 0.66 (m, 1H); ^{13}C NMR (101 MHz, $CDCl_3$) δ 176.8, 135.1, 132.1, 130.7 (2C), 129.2 (2C), 71.3, 53.4, 47.3, 46.2, 45.9, 44.5, 41.2, 38.1, 36.9, 35.7, 34.8, 34.6, 31.5850, 31.5849, 31.0, 28.6, 26.9, 21.2, 20.3, 18.5, 12.4. HRMS (FT-ICR, HESI) *m/z*: $[M + H]^+$ calcd for $C_{28}H_{41}ClNO_2S$ 490.2541, found 490.2532.

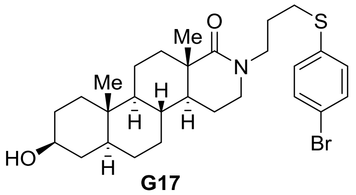

G17

3β-Hydroxy-5α-androstane-derived D-Ring Lactam, G17

Following the general procedure G, **G17** was prepared as a white amorphous solid (48.9 mg, 0.0913 mmol, 61% yield, UPLC/HRMS purity: 98.9%). Purification was carried out by an automated MPLC system using a 12 g normal phase silica column with gradient elution from 0–60% EtOAc/hexanes. R_f = 0.29 (50% EtOAc/hexanes); mp 196–199 ˚C; IR (neat) 3408, 1610 cm^{-1}; ^1H NMR (400 MHz, $CDCl_3$) δ 7.39 (m, 2H), 7.18 (m, 2H), 3.59 (m, 1H), 3.56–3.49 (m, 1H), 3.30–3.15 (m, 3H), 2.87 (m, 2H), 2.18 (m, 1H), 1.91–1.21 (complex, 18H), 1.13–1.05 (m, 4H, contains s, 1.08, 3H), 0.97 (m, 1H), 0.90–0.80 (m, 1H), 0.79 (s, 3H). 0.67 (m, 1H); ^{13}C NMR (101 MHz, $CDCl_3$) δ 176.8, 135.9, 132.1 (2C), 130.8 (2C), 119.9, 71.3, 53.4, 47.3, 46.2, 45.9, 44.5, 41.2, 38.1, 36.9, 35.7, 34.8, 34.6, 31.6, 31.4, 31.0, 28.6, 26.8, 21.2, 20.3, 18.5, 12.4. HRMS (FT-ICR, HESI) *m/z*: $[M + H]^+$ calcd for $C_{28}H_{41}BrNO_2S$ 534.2036, found 534.2028.

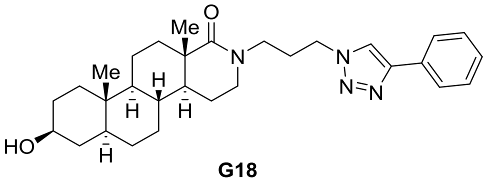

G18

3β-Hydroxy-5α-androstane-derived D-Ring Lactam, G18

Following the general procedure H, **G18** was prepared as a white amorphous solid (81.7 mg, 0.167 mmol, 83% yield, UPLC/HRMS purity: 98.9%). Purification was carried out by an automated MPLC system using a 12 g normal phase silica column with gradient elution from 0–5% MeOH/CH_2Cl_2. R_f = 0.25 (3% MeOH/CH_2Cl_2); mp 197–202 ˚C; IR (neat) 3400, 1619 cm^{-1}; ^1H NMR (400 MHz, $CDCl_3$) δ 7.93 (s, 1H), 7.84 (m, 2H), 7.41 (m, 2H), 7.32 (m, 1H), 4.40 (m, 2H), 3.58 (m, 1H), 3.42 (m, 2H), 3.30–3.15 (m, 2H), 2.36–2.13 (m, 3H), 1.87 (m, 1H), 1.69 (m, 2H), 1.72–1.10 (complex, 12H), 1.06–0.98 (m, 4H, contains s, 1.06, 3H), 2.93 (s, 3H) 0.50 (m, 1H); ^{13}C NMR (101 MHz, $CDCl_3$) δ 172.3, 147.7, 130.8, 128.9 (2C), 128.2, 125.9 (2C), 120.4, 71.3, 53.1, 48.5, 47.3, 45.6, 44.8, 44.3, 41.2, 38.1, 36.7, 35.6, 34.8, 34.5, 31.5, 30.9, 28.6, 27.7, 21.0, 20.2, 18.4, 12.3. HRMS (FT-ICR, HESI) *m/z*: $[M + H]^+$ calcd for $C_{30}H_{43}N_4O_2$ 491.3381, found 491.3372.

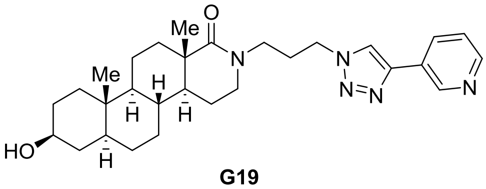

G19

3β-Hydroxy-5α-androstane-derived D-Ring Lactam, G19

Following the general procedure H, **G19** was prepared as a white amorphous solid (74.9 mg, 0.152 mmol, 84% yield, UPLC/HRMS purity: 97.9%). Purification was carried out by an automated MPLC system using a 12 g normal phase silica column with gradient elution from 0–5% MeOH/CH_2Cl_2. R_f = 0.16 (5% MeOH/CH_2Cl_2); mp 190–194 ˚C; IR (neat) 3353, 1640, 1609 cm^{-1}; ^{1}H NMR (400 MHz, $CDCl_3$) δ 9.04 (s, 1H), 8.57 (m, 1H), 8.19 (m, 1H), 8.08 (s, 1H), 7.36 (ddd, *J* = 7.9, 4.8, 0.8 Hz,) 4.42 (m, 2H), 3.59 (m, 1H), 3.50–3.17 (m, 4H), 2.34–2.12 (m, 3H), 1.91 (m, 1H), 1.83 (m, 1H), 1.71 (m, 2H), 1.62–1.15 (complex, 11H), 1.08–0.99 (m, 4H, contains s, 1.08, 3H), 0.95–0.79 (m, 2H), 0.77 (s, 3H), 0.54 (m, 1H); ^{13}C NMR (101 MHz, $CDCl_3$) δ 177.4, 149.3, 147.3, 144.6, 133.1, 127.0, 123.8, 120.9, 71.2, 53.2, 48.5, 47.3. 45.7, 44.6, 44.4, 41.3, 38.1, 36.8, 35.6, 34.8, 34.6, 31.5, 30.9, 28.6, 27.8, 21.1, 20.2, 18.5, 12.3. HRMS (FT-ICR, HESI) *m/z*: $[M + H]^+$ calcd for $C_{29}H_{42}N_5O_2$ 492.3333, found 492.3322.

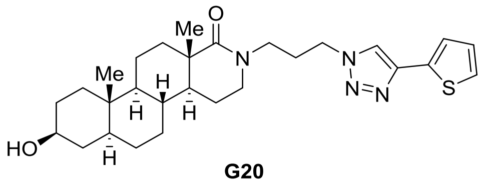

G20

3β-Hydroxy-5α-androstane-derived D-Ring Lactam, G20

Following the general procedure H, **G20** was prepared as a white foam/crystalline solid (81.5 mg, 0.164 mmol, 91% yield, UPLC/HRMS purity: 95.9%). Purification was carried out by an automated MPLC system using a 12 g normal phase silica column with gradient elution from 0–5% MeOH/CH_2Cl_2. R_f = 0.35 (5% MeOH/CH_2Cl_2); mp 140–144 ˚C; IR (neat) 3345, 1622 cm^{-1}; ^{1}H NMR (500 MHz, $CDCl_3$) δ 7.83 (s, 1H), 7.68 (m, 1H), 7.46 (m, 1H), 7.37 (m, 1H), 4.38 (m, 2H), 3.59 (tt, *J* = 10.7, 4.8 Hz, 1H), 3.41 (m, 2H), 3.28–3.15 (m, 2H), 2.28 (m, 1H), 2.16 (m, 2H), 1.88 (m, 1H), 1.82–1.67 (m, 3H), 1.60–1.18 (complex, 11H), 1.12 (m, 1H), 1.06–1.00 (m, 4H, contains s, 1.06, 3H), 0.91 (m, 1H), 0.85–0.78 (m, 1H), 0.76 (s, 3H), 0.50 (m, 1H); ^{13}C NMR (126 MHz, $CDCl_3$) δ 177.3 143.9, 132.0, 126.4, 126.0, 121.1, 120.2, 71.3, 53.0, 48.5, 47.3, 45.5, 44.8, 44.3, 41.1, 38.1, 36.7, 35.6, 34.7, 34.5, 31.5, 30.9, 28.6, 27.6, 21.0, 20.2, 18.4, 12.3. HRMS (FT-ICR, HESI) *m/z*: $[M + H]^+$ calcd for $C_{28}H_{41}N_4O_2S$ 497.2945, found 497.2936.

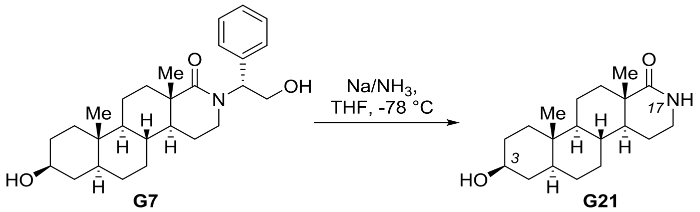

3β-Hydroxy-5α-androstane-derived D-Ring Lactam, G21

To a solution of **G7** (57.2 mg, 0.134 mmol) in anhydrous THF (4.0 mL) was condensed liquid NH_3 (~15 mL) at –78 ˚C. To the cooled solution was added pieces of sodium metal until the solution turned blue. The blue solution was stirred at –78 ˚C for 2 h, and was quenched by adding solid NH_4Cl slowly (until the disappearance of blue). The resulting mixture was diluted with H_2O (20 mL) and EtOAc (30 mL). The organic layer was washed with brine, dried over anhydrous Na_2SO_4, filtered, and concentrated. Purification was carried out by an automated MPLC system using a 12 g normal phase silica column with gradient elution from 0–5% MeOH/CH_2Cl_2 to afford **G21** as a white amorphous solid (37.1 mg, 0.121 mmol, 90% yield, UPLC/HRMS purity: ≥99.5%). R_f = 0.26 (4% MeOH/CH_2Cl_2); IR (neat) 3378, 1646 cm^{-1}; mp 253–256 ˚C; ^{1}H NMR (400 MHz, $CDCl_3$) δ 5.52 (s, 1H), 3.59 (tt, *J* = 10.7, 4.8 Hz, 1H), 3.34 (m, 1H), 3.24 (m, 1H), 2.14 (m, 1H), 1.91–1.72 (m, 3H), 1.67–1.21 (complex, 12H), 1.14 (s, 3H), 1.12–1.05 (m, 1H), 1.00–0.83 (m, 2H), 0.80 (s, 3H), 0.69 (m, 1H); ^{13}C NMR (101 MHz, $CDCl_3$) δ 179.2, 71.3, 53.5, 46.0, 44.4, 41.6, 41.1, 38.1, 36.8, 35.8, 34.6, 34.0, 31.6, 31.2, 28.6, 20.5, 20.1, 18.4, 12.4. HRMS (FT-ICR, HESI) *m/z*: $[M + H]^+$ calcd for $C_{19}H_{32}NO_2$ 306.2428, found 306.2423.

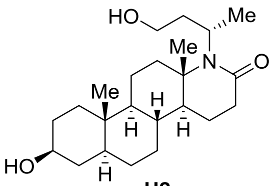

H2

3β-Hydroxy-5α-androstane-derived D-Ring Lactam, H2

Following the general procedure E, (*S*)-3-azidobutanol (*S*)-**10** (34.6 mg, 0.300 mmol 2.0 equiv) was reacted with *trans*-androsterone **4** (43.6 mg, 0.150 mmol) to give **H2** as a white amorphous solid (38.8 mg, 0.103 mmol, 69% yield, UPLC/HRMS purity: 97.2%). Purification was carried out by an automated MPLC system using a 12 g normal phase silica column with gradient elution from 0–5% MeOH/CH_2Cl_2. R_f = 0.26 (5% MeOH/CH_2Cl_2); IR (neat) 3352, 1612, 1594 cm^{-1}; mp 242–245 ˚C; ^{1}H NMR (400 MHz, $CDCl_3$) δ 3.73–3.67 (m, 2H), 3.64–3.56 (m, 2H), 2.52 (dd, *J* = 18.3, 6.5 Hz, 1H), 2.41–2.32 (m, 1H), 2.28–2.16 (m, 4H), 2.06–1.98 (m, 1H), 1.93–1.68 (complex, 4H), 1.59 (m, 1H), 1.46–1.20 (complex, 12H, contains d, *J* = 6.6 Hz, 3H), 1.18 (s, 3H), 1.14–1.06 (m, 1H), 1.02–0.83 (m, 2H), 0.78–0.71 (m, 4H, contains s, 0.78, 3H); ^{13}C NMR (101 MHz, $CDCl_3$) δ 171.6, 71.2, 61.9, 60.3, 53.1, 49.6, 48.0, 44.3, 38.5, 37.9, 37.6, 36.8, 36.2, 35.6, 33.3, 31.4, 31.0, 28.6, 21.9, 19.1, 18.9, 18.4, 12.3. **Note**: APT, NOE (1D), HSQC and HMBC are included in the spectra section. HRMS (FT-ICR, HESI) *m/z*: [M + H]$^+$ calcd for $C_{23}H_{40}NO_3$ 378.3003 found 378.2995.

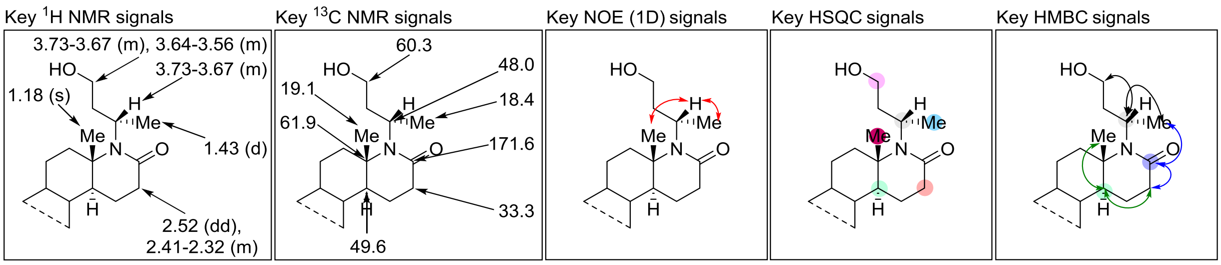

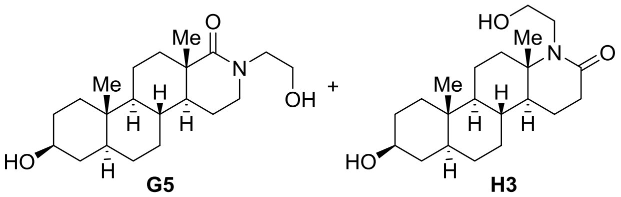

G5 **H3**

Following the general procedure E, azidoethanol **12** (26.1 mg, 0.300 mmol, 2.0 equiv) was reacted with *trans*-androsterone **4** (43.6 mg, 0.150 mmol) to give an inseparable mixture (30:70) of regioisomers **G5** and **H3** as a white amorphous solid (50.4 mg, 0.144 mmol, 96% yield, UPLC/HRMS purity: ≥99.5%). Purification was carried out by an automated MPLC system using a 12 g normal phase silica column with gradient elution from 0–5% MeOH/CH_2Cl_2. R_f = 0.26 (5% MeOH/CH_2Cl_2); IR (neat) 3328, 1600 cm^{-1}; key ^{1}H NMR (400 MHz, $CDCl_3$) δ 4.46 (br s, 1H), 3.86 (m, 1H), 3.77 (m, 2H), 3.65–3.56 (complex, 4H), 3.37 (m, 1H), 3.26 (m, 1H), 2.52 (m, 1H), 2.44 (m, 1H); ^{13}C NMR (151 MHz, $CDCl_3$) δ 179.5, 173.7, 71.3, 71.2, 65.0, 62.2, 60.5, 53.4, 53.0, 51.4, 48.8, 48.7, 45.7, 44.9, 44.4, 44.3, 41.3, 38.2, 38.1, 38.0, 36.9, 36.3, 35.7, 35.6, 34.8, 34.5, 31.7, 31.6, 31.5, 31.0, 30.9, 28.6, 28.5, 21.6, 21.2, 20.3, 19.3, 19.0, 18.5, 12.4, 12.3. **Note**: Missing one carbon signal due to signal overlap of regioisomers. HRMS (FT-ICR, HESI) *m/z*: [M + H]$^+$ calcd for $C_{21}H_{36}NO_3$ 350.2690, found 350.2683.

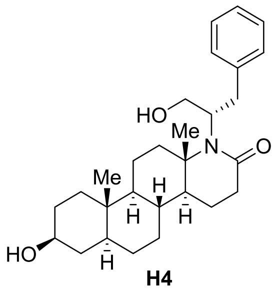

H4

3β-Hydroxy-5α-androstane-derived D-Ring Lactam, H4

Following the general procedure E, (*S*)-2-azido-3-phenylpropanol (*S*)-**14** (53.2 mg, 0.300 mmol, 2.0 equiv) was reacted with *trans*-androsterone **4** (43.6 mg, 0.150 mmol) to give **H4** as a white amorphous solid (58.9 mg, 0.134 mmol, 89% yield, UPLC/HRMS purity: 96.9%). Purification was carried out by an automated MPLC system using a 12 g normal phase silica column with gradient elution from 50–100% EtOAc/hexanes. R_f = 0.26 (80% EtOAc/hexanes); mp 236–240 ˚C; IR (neat) 3332, 1596 cm^{-1}; ^1H NMR (400 MHz, $CDCl_3$) δ 7.31–7.18 (m, 5H), 3.76–3.68 (m, 2H), 3.63–3.49 (m, 3H), 2.99 (m, 1H), 2.56 (m, 1H), 2.42 (ddd, *J* = 18.6, 10.2, 8.0 Hz, 1H), 1.98 (m, 1H), 1.92–1.79 (m, 3H), 1.71–1.65 (m, 2H), 1.61–1.56 (m, 1H), 1.44–1.03 (complex, 13H), 1.00–0.80 (m, 4H, contains s, 0.88, 3H), 0.76–0.68 (m, 4H, contains s, 0.74, 3H); ^{13}C NMR (101 MHz, $CDCl_3$) δ 173.3, 140.0, 129.7 (2C), 128.6 (2C), 126.6, 71.2, 65.6, 62.0, 59.7, 53.0, 49.8, 44.3, 37.9, 37.8, 36.8, 36.2, 35.6, 34.6, 33.1, 31.4, 31.1, 28.5, 21.7, 18.8, 18.3, 12.2. HRMS (FT-ICR, HESI) *m/z*: $[M + H]^+$ calcd for $C_{28}H_{42}NO_3$ 440.3159, found 440.3137.

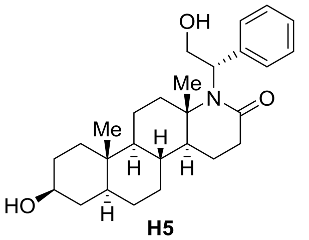

H5

3β-Hydroxy-5α-androstane-derived D-Ring Lactam, H5

Following the general procedure E, (*S*)-2-azido-2-phenylethanol (*S*)-**15** (40.8 mg, 0.250 mmol, 2.0 equiv) was reacted with *trans*-androsterone **4** (36.3 mg, 0.125 mmol) to give **H5** as a white amorphous solid (40.8 mg, 0.0959 mmol, 77% yield, UPLC/HRMS purity: 97.8%). Purification was carried out by an automated MPLC system using a 12 g normal phase silica column with gradient elution from 50–100% EtOAc/hexanes. R_f = 0.38 (5% MeOH/CH_2Cl_2); mp 212–217 ˚C; IR (neat) 3352, 3235, 1608 cm^{-1}; ^1H NMR (400 MHz, $CDCl_3$) δ 7.33–7.28 (m, 4H), 7.24–7.19 (m, 1H), 4.75 (m, 1H), 4.41 (dd, *J* = 11.9, 5.3 Hz, 1H), 4.03 (dd, *J* = 11.9, 2.4 Hz, 1H), 3.60 (m, 1H), 2.62 (ddd, *J* = 18.8, 7.3, 1.6 Hz, 1H), 2.48 (ddd, *J* = 18.8, 10.5, 8.4 Hz, 1H), 2.16 (m, 1H), 1.99–1.91 (m, 2H), 1.83–1.68 (m, 4H), 1.62–1.19 (complex, 11H), 1.15 (s, 3H), 1.12–0.88 (m, 3H), 0.82–0.76 (m, 4H, contains s, 0.77, 3H); ^{13}C NMR (101 MHz, $CDCl_3$) δ 173.0, 138.3, 128.4 (2C), 127.2 (2C), 126.7, 71.1, 66.1, 62.2, 59.7, 53.0, 49.9, 44.3, 38.4, 37.9, 36.9, 36.4, 35.6, 32.9, 31.4, 31.1, 28.5, 22.0, 19.3, 19.1, 12.3. HRMS (FT-ICR, HESI) *m/z*: $[M + H]^+$ calcd for $C_{27}H_{39}NO_3$ 426.3003, found 426.2983.

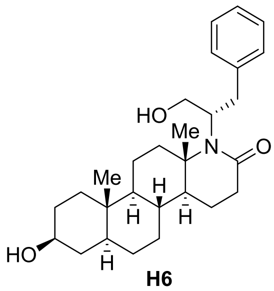

H6

3β-Hydroxy-5α-androstane-derived D-Ring Lactam, H6

Following the general procedure E, (*S*)-2-azido-4-methylpentanol (*S*)-**16** (43.2 mg, 0.302 mmol, 2.0 equiv) was reacted with *trans*-androsterone **4** (43.9 mg, 0.151 mmol) to give **H6** as a white amorphous solid (52.6 mg, 0.130 mmol, 86% yield, UPLC/HRMS purity: ≥99.5%). Purification was carried out by an automated MPLC system using a 12 g normal phase silica column with gradient elution from 0–80% EtOAc/hexanes. R_f = 0.33 (80% EtOAc/hexanes); mp 164–168 ˚C; IR (neat) 3317, 1604 cm^{-1}; ^{1}H NMR (400 MHz, $CDCl_3$) δ 5.68 (d, *J* = 9.3 Hz, 1H), 3.82 (m, 1H), 3.67–3.56 (m, 2H), 3.51 (m, 1H), 2.64 (ddd, *J* = 13.6, 11.1, 4.0 Hz, 1H), 2.50 (ddd, *J* = 18.6, 7.0, 1.5 Hz, 1H), 2.37 (ddd, *J* = 18.8, 11.0, 8.1 Hz, 1H), 2.10 (dt, *J* = 12.3, 3.6 Hz, 1H), 1.93–1.06 (complex, 20H, contains s, 1.18, 3H), 1.02–0.84 (complex, 10H, contains dd, 0.94, *J* = 6.6, 1.3 Hz, 6H), 0.79–0.71 (complex, 4H, contains s, 0.78, 3H); ^{13}C NMR (101 MHz, $CDCl_3$) δ 172.8, 71.1, 64.9, 61.9, 55.1, 53.0, 49.7, 44.3, 37.9, 37.5, 36.8, 36.0, 35.6, 33.0, 31.4, 31.0, 25.5, 24.1, 21.9, 21.8, 18.9, 18.8, 12.3. **Note**: Missing two carbons signal due to signal overlap. HRMS (FT-ICR, HESI) *m/z*: $[M + H]^+$ calcd for $C_{25}H_{44}NO_3$ 406.3316, found 406.3296.

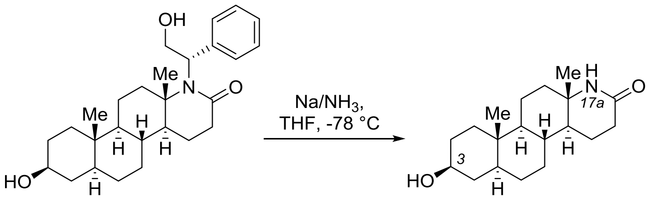

H6 **Beck1**

3β-Hydroxy-5α-androstane-derived D-Ring Lactam, Beck1

To a solution of **H5** (85.0 mg, 0.200 mmol) in anhydrous THF (4.0 mL) was condensed liquid NH_3 (~15 mL) at –78 ˚C. To the cooled solution was added pieces of sodium metal until the solution turned blue. The blue solution was stirred at –78˚C for 2 h, and was quenched by adding solid NH_4Cl slowly (until the disappearance of blue). The resulting mixture was diluted with H_2O (20 mL) and EtOAc (30 mL). The organic layer was washed with brine, dried over anhydrous Na_2SO_4, filtered, and concentrated. Purification was carried out by an automated MPLC system using a 12 g normal phase silica column with gradient elution from 0–5% MeOH/CH_2Cl_2 to afford **Beck1** as a white amorphous solid (56.8 mg, 0.186 mmol, 93% yield, UPLC/HRMS purity: ≥99.5%). Characterization data were consistent to **Beck1**. HRMS (FT-ICR, HESI) *m/z*: $[M + H]^+$ calcd for $C_{19}H_{32}NO_2$ 306.2428, found 306.2423.

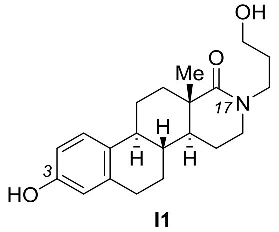

I1

3-Hydroxy-1,3,5-estratriene-derived D-Ring Lactam, I1

Following the general procedure F, 3-azidopropanol **6** (30.5 mg, 0.302 mmol, 2.0 equiv) was reacted with estrone **5a** (40.6 mg, 0.150 mmol) to give **I1** as a white amorphous solid (46.7 mg, 0.136 mmol, 91% yield, UPLC/HRMS purity: ≥99.5%). Purification was carried out by an automated MPLC system using a 4 g normal phase silica column with gradient elution from 0–5% MeOH/CH_2Cl_2. R_f = 0.35 (5% MeOH/CH_2Cl_2); mp 228–235 ˚C; IR (neat) 3124, 1599 cm^{-1}; ^{1}H NMR (400 MHz, DMSO-*d*$_6$) δ 9.00 (s, 1H), 7.06 (d, *J* = 8.4 Hz, 1H), 6.51 (dd, *J* = 8.4, 2.6, 1H), 6.44 (d, *J* = 2.6 Hz, 1H), 3.39–3.22 (complex, 8H), 3.20–3.13 (m, 1H), 2.72 (m, 2H), 2.28 (m, 1H), 2.11 (m, 2H), 1.99 (m, 1H), 1.63–1.15 (complex, 6H), 1.02 (s, 3H); ^{13}C NMR (126 MHz, DMSO-*d*$_6$) δ 175.2, 155.0, 137.0, 130.2, 125.9, 114.6, 112.8, 58.3, 46.3, 43.9, 43.4, 42.3, 40.4, 38.1, 34.4, 29.8, 29.3, 25.7, 25.5, 20.4, 17.9. HRMS (FT-ICR, HESI) *m/z*: $[M + H]^+$ calcd for $C_{21}H_{30}NO_3$ 344.2220, found 344.2214.

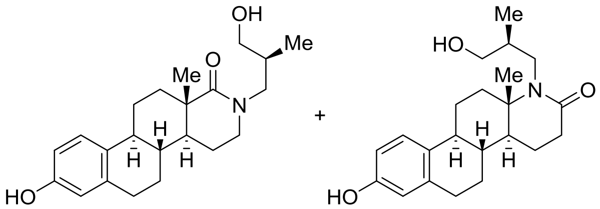

I2 **J1**

3-Hydroxy-1,3,5-estratriene-derived D-Ring Lactams, I2 and J1

Following the general procedure F, (*S*)-3-azido-2-methylpropanol (*S*)-**8** (46.0 mg, 0.400 mmol, 2.0 equiv) was reacted with estrone **5a** (54.0 mg, 0.200 mmol) to afford a separable mixture of two regioisomeric lactams **I2** (37.1 mg, 0.104 mmol, 52% yield, UPLC/HRMS purity: ≥99.5%) and **J1** (24.2 mg, 0.068 mmol, 34% yield, UPLC/HRMS purity: ≥99.5%). A 60:40 regioisomeric ratio was observed by analytical HPLC of crude reaction mixture: Chiralpak IA, Daicel Chemical Industries, Ltd.; 0–30% EtOH/hexanes over 60 min; flow rate 1.0 mL/min; UV 220 nm; **I2** = 40.48 min, **J1** = 48.09 min. Purification was carried out by an automated MPLC system using a 12 g normal phase silica column with gradient elution from 0–80% EtOAc/hexanes. **I2:** R_f = 0.48 (80% EtOAc/hexanes); mp 189–200 ˚C; IR (neat) 3197, 1615, 1602 cm^{-1}; ^{1}H NMR (400 MHz, DMSO-*d*$_6$) δ 9.00 (s, 1H), 7.06 (d, *J* = 8.4 Hz, 1H), 6.51 (dd, *J* = 8.4, 2.6 Hz, 1H), 6.44 (d, J = 2.5 Hz, 1H) 4.42 (t, *J* = 5.5 Hz, 1H), 3.37–3.19 (complex, 6H), 3.00 (dd, *J* = 13.2, 7.3 Hz, 1H), 2.72 (m, 2H), 2.27 (m, 1H), 2.17–2.08 (m, 2H), 1.98 (m, 1H), 1.88 (m, 1H), 1.65–1.15 (complex, 6H), 1.03 (s, 3H), 0.79 (dd, *J* = 6.8 Hz, 3H); ^{13}C NMR (126 MHz, DMSO-*d*$_6$) δ 175.6, 155.0, 137.0, 130.2, 126.0, 114.7, 112.8, 63.7, 48.9, 46.9, 43.9, 42.3, 40.6, 38.2, 34.5, 33.6, 29.4, 25.8, 25.6, 20.5, 18.1, 14.9. HRMS (FT-ICR, HESI) *m/z* calcd for $C_{22}H_{32}NO_3$ $[M + H]^+$ 358.2377, found 358.2372. **J1:** R_f = 0.29 (80% EtOAc/hexanes); mp 255–268 ˚C; IR (neat) 3135, 1569 cm^{-1}; ^{1}H NMR (400 MHz, DMSO-*d*$_6$) δ 9.02 (s, 1H), 7.06 (d, *J* = 8.4 Hz, 1H), 6.52 (dd, *J* = 8.4, 2.6 Hz, 1H), 6.45 (d, *J* = 2.6 Hz, 1H), 4.43 (t, *J* = 5.5 Hz, 1H), 3.38 (dd, *J* = 14.0, 8.0 Hz, 1H), 3.28–3.16 (m, 3H), 2.72 (m, 2H), 2.40–2.33 (m, 3H), 2.26 (m, 1H), 2.16 (m, 1H), 2.04–1.92 (m, 2H), 1.84 (h, *J* = 6.9, 6.2 Hz, 1H), 1.65 (m, 1H), 1.55–1.36 (m, 2H), 1.29–1.15 (m, 3H), 1.12 (s, 3H), 0.79 (d, *J* = 6.8 Hz, 3H); ^{13}C NMR (126 MHz, DMSO-*d*$_6$) δ 170.4, 155.1, 137.0, 129.9, 126.0, 114.6, 112.8, 64.3, 59.5, 46.7, 42.9, 41.8, 40.1, 37.5, 37.0, 31.0, 29.4, 26.6, 25.8, 19.5, 19.2, 15.3. HRMS (FT-ICR, HESI) *m/z*: $[M + H]^+$ calcd for $C_{22}H_{33}NO_3$ 358.2377, found 358.2370.

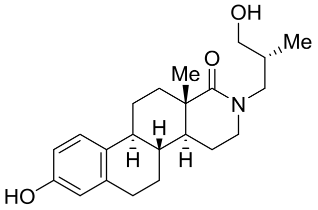

I3

3-Hydroxy-1,3,5-estratriene-derived D-Ring Lactam, I3

Following the general procedure F, (*R*)-3-azido-2-methylpropanol (*R*)-**8** (34.5 mg, 0.300 mmol, 2.0 equiv) was reacted with estrone **5a** (40.6 mg, 0.150 mmol) to give **I3** as a white amorphous solid (45.8

mg, 0.128 mmol, 85% yield, UPLC/HRMS purity: 96.4%). Purification was carried out by an automated MPLC system using a 12 g normal phase silica column with gradient elution from 0–50% EtOAc/hexanes. R_f = 0.15 (50% EtOAc/hexanes); mp 219–222 ˚C; IR (neat) 3514, 1602 cm^{-1}; ^1H NMR (400 MHz, DMSO-d_6) δ 9.00 (s, 1H), 7.06 (d, J = 8.4 Hz, 1H), 6.51 (dd, J = 8.4, 2.6 Hz, 1H), 6.44 (d, 2.6 Hz, 1H), 4.37 (m, 1H), 3.36–3.16 (complex, 5H), 2.95 (dd, J = 13.3, 7.2 Hz, 1H), 2.72 (m, 2H), 2.28 (m, 1H), 2.17–2.09 (m, 2H), 1.99 (m, 2H), 1.88 (h, J = 6.8 Hz, 1H), 1.61–1.15 (complex, 6H), 3.02 (s, 3H), 0.81 (d, J = 6.8 Hz, 3H); ^{13}C NMR (151 MHz, DMSO-d_6) δ 175.7, 155.0, 137.0, 130.2, 126.0, 114.7, 112.8, 63.8, 49.0, 46.9, 43.8, 42.3, 40.6, 38.1, 34.5, 33.5, 29.4, 25.8, 25.6, 20.4, 18.1, 14.9. HRMS (FT-ICR, HESI) m/z: $[M + H]^+$ calcd for $C_{22}H_{32}NO_3$ 358.2377, found 358.2371.

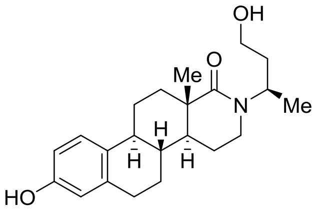

I4

3-Hydroxy-1,3,5-estratriene-derived D-Ring Lactam, I4

Following the general procedure F, (R)-3-azidobutanol (R)-**10** (33.8 mg, 0.294 mmol, 2.0 equiv) was reacted with estrone **5a** (40.4 mg, 0.149 mmol) to give **I4** as a white amorphous solid (46.2 mg, 0.129 mmol, 86% yield, UPLC/HRMS purity: 97.9%). Purification was carried out by an automated MPLC system using a 12 g normal phase silica column with gradient elution from 0–4% MeOH/CH_2Cl_2. R_f = 0.38 (5% MeOH/CH_2Cl_2); mp 246–242 ˚C; IR (neat) 3160, 1585 cm^{-1}; ^1H NMR (600 MHz, DMSO-d_6) δ 8.95 (s, 1H), 7.05 (d, J = 8.5 Hz, 1H), 6.52 (dd, J = 8.4, 2.6 Hz, 1H), 6.44 (d, J = 2.6 Hz, 1H), 4.57 (m, 1H), 4.27 (t, J = 5.4 Hz, 1H), 3.30 (m, 1H), 3.21–3.10 (m, 2H), 2.73 (m, 1H), 2.27 (m, 1H), 2.16–1.98 (complex, 4H), 1.64 (m, 1H), 1.55–1.41 (complex, 4H), 1.34–1.15 (complex, 3H), 1.06 (d, J = 6.9 Hz, 3H), 1.02 (s, 3H); ^{13}C NMR (151 MHz, DMSO-d_6) δ 175.2, 154.9, 136.9, 130.2, 125.8, 114.6, 112.7, 58.1, 44.8, 43.6, 42.2, 40.8, 38.1, 36.1, 34.5, 29.2, 25.7, 25.5, 20.3, 18.0, 17.3. **Note**: Missing one carbon signal due to signal overlap; APT has been included in the spectra section. HRMS (FT-ICR, HESI) m/z: $[M + H]^+$ calcd for $C_{22}H_{32}NO_3$ 358.2377, found 358.2371.

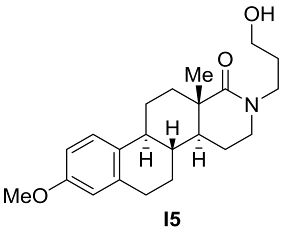

I5

3-Methoxy-1,3,5-estratriene-derived D-Ring Lactam, I5

Following the general procedure F, 3-azidopropanol **6** (30.6 mg, 0.303 mmol 2.0 equiv) was reacted with estrone 3-methyl ether **5b** (42.7 mg, 0.150 mmol) to give **I5** as an off-white amorphous solid (50.0 mg, 0.140 mmol, 93% yield, UPLC/HRMS purity: 97.8%). Purification was carried out by an automated MPLC system using a 12 g normal phase silica column with gradient elution from 0–5% MeOH/CH_2Cl_2. R_f = 0.35 (5% MeOH/CH_2Cl_2); mp 142–145 ˚C; IR (neat) 3354, 1601 cm^{-1}; ^1H NMR (400 MHz, $CDCl_3$) δ 7.22 (d, J = 8.6 Hz, 1H), 6.73 (dd, J = 8.6, 2.8 Hz, 1H), 6.63 (d, J = 2.6 Hz, 1H), 4.20 (br s, 1H), 3.77 (s, 3H), 3.68 (ddd, J = 14.0, 8.2, 4.6 Hz, 1H), 3.52–3.41 (m, 2H), 3.89–3.25 (m, 3H), 2.87 (m, 2H), 2.41–2.32 (m, 2H), 2.30–2.21 (m, 1H), 2.06 (m, 2H), 1.79–1.21 (complex, 8H), 1.16 (s, 3H); ^{13}C NMR (101 MHz, $CDCl_3$) δ 178.2, 157.8, 137.6, 132.3, 126.4, 113.7, 111.8, 58.0, 55.3, 47.0, 44.7, 43.0, 42.9, 41.4, 38.5, 34.6, 30.0, 29.4, 26.4, 25.9, 21.0, 18.5. HRMS (FT-ICR, HESI) m/z: [M + H]$^+$ calcd for $C_{22}H_{32}NO_3$ 358.2377, found 358.2371.

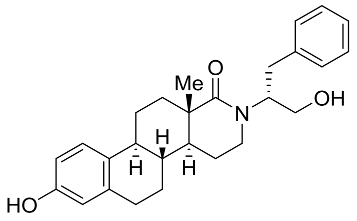

I7

3-Hydroxy-1,3,5-estratriene-derived D-Ring Lactam, I7

Following the general procedure F, (*R*)-2-azido-3-phenylpropanol (*R*)-**14** (53.2 mg, 0.300 mmol, 2.0 equiv) was reacted with estrone **5a** (41.2 mg, 0.152 mmol) to give **I7** as a white crystalline solid (45.6 mg, 0.109 mmol, 72% yield, UPLC/HRMS purity: 98.5%) containing a minor uncharacterized regioisomer. Purification was carried out by an automated MPLC system using a 12 g normal phase silica column with gradient elution from 0–5% MeOH/CH_2Cl_2. R_f = 0.40 (5% MeOH/CH_2Cl_2); IR (neat) 3298, 1609 cm^{-1}; mp 115–134 °C; ^{1}H NMR (400 MHz, DMSO-*d*$_6$) δ 9.00 (s, 1H), 7.25 (m, 2H), 7.18 (m, 3H), 7.03 (d, *J* = 8.5 Hz, 1H), 6.50 (dd, *J* = 8.4, 2.6 Hz, 1H), 6.42 (d, *J* = 2.7 Hz, 1H), 4.73 (m, 1H), 3.57 (m, 1H), 3.48 (m, 1H), 3.14 (m, 2H), 2.82 (m, 2H), 2.69 (m, 3H), 2.21 (m, 1H), 2.00–1.86 (m, 3H), 1.40–1.03 (complex, 7H), 0.94 (s, 3H); ^{13}C NMR (151 MHz, DMSO-*d*$_6$) δ 175.6, 155.0, 138.9, 137.0, 130.2, 128.9 (2C), 128.0 (2C), 125.93, 125.90, 114.6, 112.8, 61.0, 43.1, 42.4, 40.7, 38.2, 34.7, 33.7, 29.3, 25.8, 25.5, 20.4, 17.5. **Note**: Missing two carbon signals due to signal overlap; ^{1}H and ^{13}C NMR spectra of mixture are included in the spectra section. Characterization above only denote peaks of the major regioisomer. HRMS (FT-ICR, HESI) *m/z*: [M + H]$^+$ calcd for $C_{27}H_{34}NO_3$ 420.2533, found 420.2527.

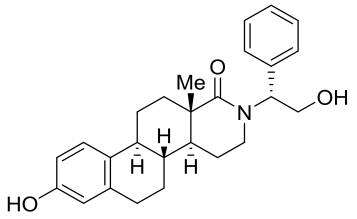

I8

3-Hydroxy-1,3,5-estratriene-derived D-Ring Lactam, I8

Following the general procedure F, (*R*)-2-azido-2-phenylethanol (*R*)-**15** (41.7 mg, 0.256 mmol, 2.0 equiv) was reacted with estrone **5a** (34.5 mg, 0.128 mmol) to give **I8** as a yellow solid/oil (29.1 mg, 0.072 mmol, 56% yield, UPLC/HRMS purity: ≥99.5%) containing a minor uncharacterized regioisomer. Purification was carried out by an automated MPLC system using a 12 g normal phase silica column with gradient elution from 0–2% MeOH/CH_2Cl_2. R_f = 0.17 (2% MeOH/CH_2Cl_2, run twice); IR (neat) 3414, 3344, 1591 cm^{-1}; ^{1}H NMR (400 MHz, DMSO-*d*$_6$) δ 8.99 (s, 1H), 7.34 (m, 2H), 7.26 (m, 3H), 7.05 (d, *J* = 8.5 Hz, 1H), 6.51 (dd, *J* = 8.4, 2.6 Hz, 1H), 6.43 (d, *J* = 2.6 Hz, 1H), 5.63 (t, *J* = 7.3 Hz, 1H), 4.87 (t, *J* = 5.5 Hz, 1H), 3.92 (m, 1H), 3.83 (m, 1H), 2.84 (m, 1H), 2.70 (m, 2H), 2.28 (m, 1H), 2.13 (m, 1H), 1.93 (m, 1H), 1.58–1.11 (complex, 8H), 1.08 (s, 3H); ^{13}C NMR (101 MHz, DMSO-*d*$_6$) δ 175.8, 155.0, 138.5, 137.0, 130.2, 128.3 (2C), 127.7 (2C), 126.9, 125.9, 114.6, 112.8, 59.7, 56.4, 43.3, 42.1, 41.1, 40.9, 38.4, 34.8, 29.3, 25.7, 25.6, 20.4, 17.8. **Note**: ^{1}H and ^{13}C NMR spectra of mixture are included in the spectra section. Characterization above only denote peaks of the major regioisomer. HRMS (FT-ICR, HESI) *m/z*: [M + H]$^+$ calcd for $C_{26}H_{32}NO_3$ 406.2377, found 406.2376.

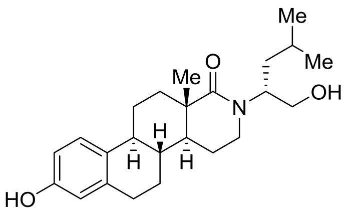

I9

3-Hydroxy-1,3,5-estratriene-derived D-Ring Lactam, I9

Following the general procedure F, (*R*)-2-azido-4-methylpentanol (*R*)-**16** (43.4 mg, 0.303 mmol, 2.0 equiv) was reacted with estrone **5a** (41.6 mg, 0.153 mmol) to give **I9** as a white crystalline solid (40.2 mg, 0.104 mmol, 68% yield, UPLC/HRMS purity: 98.8%). Purification was carried out by an automated MPLC system using a 12 g normal phase silica column with gradient elution from 0–5% MeOH/CH_2Cl_2. R_f = 0.17 (5% MeOH/CH_2Cl_2); IR (neat) 3247, 1601 cm^{-1}; mp 117–142 ˚C; ^{1}H NMR (400 MHz, $CDCl_3$) δ 9.00 (s, 1H), 7.05 (d, *J* = 8.5 Hz, 1H), 6.51 (dd, *J* = 8.5, 2.5 Hz, 1H), 6.44 (d, *J* = 2.6 Hz, 1H), 4.58 (t, *J* = 5.6 Hz, 1H), 4.48 (m, 1H), 3.42–3.24 (m, 3H), 3.09 (m, 1H), 2.72 (m, 2H), 2.29–2.25 (m, 1H), 2.17–2.08 (m, 2H), 2.00–1.97 (m, 2H), 1.56–1.45 (complex, 9H), 1.02 (s, 3H), 0.87 (dd, *J* = 8.5, 6.3 Hz, 6H); ^{13}C NMR (151 MHz, $CDCl_3$) δ 175.6, 155.0, 137.0, 130.3, 125.9, 114.6, 112.8, 61.8, 43.6, 42.3, 40.9, 38.4, 36.2, 34.9, 29.4, 25.7, 25.6, 24.4, 23.5, 21.9, 20.5, 17.8. **Note**: Missing two carbon signals due to signal overlap. HRMS (FT-ICR, HESI) *m/z*: $[M + H]^+$ calcd for $C_{24}H_{36}NO_3$ 386.2690, found 386.2684.

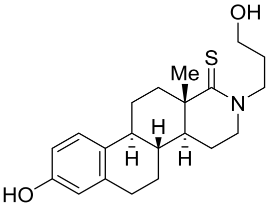

I10

3-Hydroxy-1,3,5-estratriene-derived D-Ring Thioamide, I10

Following the general procedure G, **I10** was prepared as a white amorphous solid (55.1 mg, 0.153 mmol, 51% yield, UPLC/HRMS purity: 92.0%). Purification was carried out by an automated MPLC system using a 12 g normal phase silica column with gradient elution from 0–1.5% MeOH/CH_2Cl_2. R_f = 0.40 (5% MeOH/CH_2Cl_2); mp 216–219 ˚C; IR (neat) 3227, 1531, 1501 cm^{-1}; ^{1}H NMR (400 MHz, DMSO-d_6) δ 8.99 (s, 1H), 7.06 (d, *J* = 8.5 Hz, 1H), 6.52 (d, *J* = 8.5 Hz, 1H), 6.44 (s, 1H), 4.50 (m, 1H), 4.02 (m, 1H), 3.78 (m, 1H), 3.63 (m, 1H), 3.51 (m, 1H), 3.43 (m, 2H), 2.70 (m, 3H), 2.31 (m, 1H), 2.07 (m, 3H), 1.81 (m, 2H), 1.64–1.15 (complex, 6H), 1.08 (s, 3H); ^{13}C NMR (126 MHz, DMSO-d_6) δ 207.8, 155.0, 137.0, 130.2, 126.0, 114.7, 112.8, 58.5, 52.2, 50.6, 45.2, 42.3, 41.9, 39.3, 29.4, 28.2, 26.4, 25.8, 20.6, 20.4. **Note**: Missing one carbon signal due to signal overlap. HRMS (FT-ICR, HESI) *m/z*: $[M + H]^+$ calcd for $C_{21}H_{30}NO_2S$ 360.1992, found 360.1990.

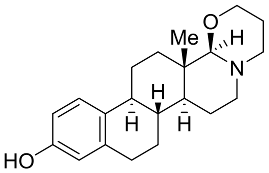

I11

3-Hydroxy-1,3,5-estratriene-derived D-Ring Oxazinane, I11

Following the general procedure G, **I11** was prepared as a white amorphous solid (54.2 mg, 0.166 mmol, 83% yield, LCMS purity: ≥99.5%). Purification was carried out by an automated MPLC system using a 12 g normal phase silica column with gradient elution from 0–8% MeOH (0.5% NH_4OH)/CH_2Cl_2. R_f = 0.20 (5% MeOH (0.5% NH_4OH)/CH_2Cl_2); mp 206–212 ˚C; IR (neat) 2936, 2851,

1609, 1509 cm^{-1}; ^{1}H NMR (400 MHz, DMSO-d_6) δ 8.97 (s, 1H), 7.03 (d, *J* = 8.6 Hz, 1H), 6.50 (dd, *J* = 8.4, 2.6 Hz, 1H), 6.42 (d, *J* = 2.6 Hz, 1H), 3.93 (m, 1H), 3.29 (m, 1H), 2.83–2.67 (m, 4H), 2.21 (m, 1H), 2.12 (m, 1H), 2.05–1.90 (m, 3H), 1.84–1.68 (m, 2H), 1.56 (m, 1H), 1.40 (m, 1H), 1.30–1.10 (complex, 6H), 1.03–0.96 (m, 1H), 0.89 (s, 3H); ^{13}C NMR (126 MHz, DMSO-d_6) δ 154.9, 137.0, 130.4, 125.9, 114.7, 112.8, 101.1, 66.5, 53.9, 53.6, 47.3, 42.8, 37.9, 37.7, 35.5, 29.4, 25.7, 25.6, 25.4, 23.0, 13.6. HRMS (FT-ICR, HESI) *m/z*: [M + H]$^+$ calcd for $C_{21}H_{30}NO_2$ 328.2271, found 328.2267.

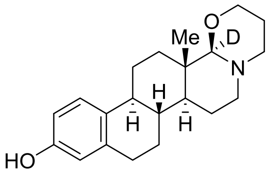

I12

3-Hydroxy-1,3,5-estratriene-derived D-Ring Oxazinane, I12

Following the general procedure G, **I12** was prepared as a white amorphous solid (53.9 mg, 0.164 mmol, 82% yield, LCMS purity: ≥99.5%). Purification was carried out by an automated MPLC system using a 12 g normal phase silica column with gradient elution from 0–10% MeOH (0.5% NH_4OH)/CH_2Cl_2. R_f = 0.20 (5% MeOH (0.5% NH_4OH)/CH_2Cl_2); mp 212–215 ˚C; IR (neat) 2936, 2855, 1610, 1505 cm^{-1}; ^{1}H NMR (400 MHz, DMSO-d_6) δ 8.97 (s, 1H), 7.03 (d, *J* = 8.3 Hz, 1H), 6.50 (dd, *J* = 8.4, 2.6 Hz, 1H), 6.42 (d, *J* = 2.6 Hz, 1H), 3.93 (m, 1H), 3.30 (m, 1H), 2.83 (m, 1H), 2.75–2.68 (m, 3H), 2.21 (m, 1H), 2.12 (m, 1H), 2.05–1.89 (m, 3H), 1.84–1.68 (m, 2H), 1.55 (m, 1H), 1.40 (m, 1H), 1.30–1.05 (complex, 5H), 1.02–0.96 (m, 1H), 0.89 (s, 3H); ^{13}C NMR (126 MHz, DMSO-d_6) δ 154.9, 137.0, 130.4, 125.9, 114.7, 112.8, 101.1, 66.5, 53.9, 53.6, 47.3, 42.8, 37.9, 37.6, 35.5, 29.4, 25.7, 25.6, 25.4, 23.0, 13.6. HRMS (FT-ICR, HESI) *m/z*: [M + H]$^+$ calcd for $C_{21}H_{29}DNO_2$ 329.2334, found 329.2329.

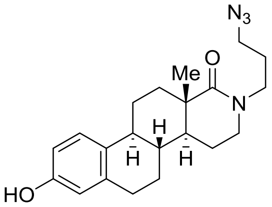

I13

3-Hydroxy-1,3,5-estratriene-derived D-Ring Lactam, I13

Following the general procedure G, **I13** was prepared as an off-white amorphous solid (45.2 mg, 0.123 mmol, 82% yield, UPLC/HRMS purity: 94.5%). Purification was carried out by an automated MPLC system using a 4 g normal phase silica column with gradient elution from 0–30% EtOAc/hexanes. R_f = 0.50 (50% EtOAc/hexanes); mp 204–210 ˚C; IR (neat) 3323, 2098, 1619, 1607 cm^{-1}; ^{1}H NMR (400 MHz, DMSO-d_6) δ 9.00 (s, 1H), 7.06 (d, *J* = 8.4 Hz, 1H), 6.51 (dd, *J* = 8.4, 2.7 Hz, 1H), 6.43 (d, *J* = 2.6 Hz, 1H), 3.40–3.14 (complex, 6H), 2.72 (m, 2H), 2.28 (m, 1H), 2.11 (m, 2H), 2.00 (m, 2H), 1.72 (p, *J* = 6.9 Hz, 2H), 1.61–1.11 (complex, 6H), 1.02 (s, 3H); ^{13}C NMR (101 MHz, DMSO-d_6) δ 175.1, 155.0, 137.0, 130.2, 125.8, 114.6, 112.8, 48.6, 46.3, 43.8, 43.7, 42.3, 40.5, 38.1, 34.4, 29.3, 25.9, 25.7, 25.5, 20.4, 17.9. HRMS (FT-ICR, HESI) *m/z*: [M + H]$^+$ calcd for $C_{21}H_{29}N_4O_2$ 369.2285, found 369.2283.

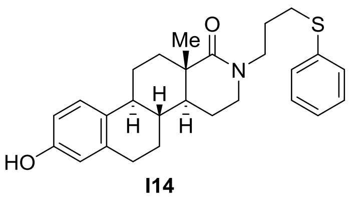

I14

3-Hydroxy-1,3,5-estratriene-derived D-Ring Lactam, I14

Following the general procedure G, **I14** was prepared as an off-white amorphous solid (40.6 mg, 0.0932 mmol, 62% yield, UPLC/HRMS purity: 97.8%). Purification was carried out by an automated MPLC system using a 12 g normal phase silica column with gradient elution from 0–60% EtOAc/hexanes. R_f = 0.63 (50% EtOAc/hexanes); mp 209–215 ˚C; IR (neat) 3053, 1560, 1585 cm^{-1}; ^{1}H NMR (400 MHz, DMSO-d_6) δ 9.01 (s, 1H), 7.32 (m, 4H), 7.19 (m, 1H), 7.05 (d, *J* = 8.5 Hz, 1H), 6.51 (dd, *J* = 8.4, 2.6 Hz, 1H), 6.44 (d, *J* = 2.6 Hz, 1H), 3.41 (m, 1H), 3.32–3.19 (m, 4H), 2.91 (m, 2H), 2.72 (m, 2H), 2.26 (m, 1H), 2.10 (m, 2H), 1.98 (m, 1H), 1.76 (p, *J* = 7.2 Hz, 2H), 1.57–1.14 (complex, 6H), 1.01 (s, 3H); ^{13}C NMR (126 MHz DMSO-d_6) δ 175.2, 155.0, 137.0, 136.3, 130.2, 129.1 (2C), 128.0 (2C), 126.0, 125.6, 114.7, 112.8, 46.5, 45.4, 43.8, 42.3, 40.5, 38.1, 34.4, 29.7, 29.4, 26.3, 25.8, 25.6, 20.4, 18.0. HRMS (FT-ICR, HESI) *m/z*: calcd for $C_{27}H_{34}NO_2S$ [M + H]$^+$ 436.2305, found 436.2301.

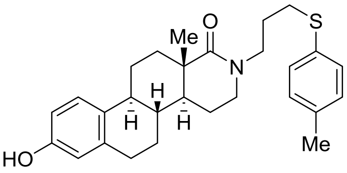

I15

3-Hydroxy-1,3,5-estratriene-derived D-Ring Lactam, I15

Following the general procedure G, **I15** was prepared as a white amorphous solid (40.2 mg, 0.0894 mmol, 60% yield, UPLC/HRMS purity: 99.0%). Purification was carried out by an automated MPLC system using a 12 g normal phase silica column with gradient elution from 0–50% EtOAc/hexanes. R_f = 0.50 (50% EtOAc/hexanes); mp 208–212 ˚C; IR (neat) 3282, 1602 cm^{-1}; ^{1}H NMR (400 MHz, DMSO-d_6) δ 9.00 (s, 1H), 7.22 (m, 2H), 7.13 (m, 2H), 7.05 (d, *J* = 8.4 Hz, 1H), 6.51 (dd, *J* = 8.4, 2.6 Hz, 1H), 6.44 (d, *J* = 2.6 Hz, 1H), 3.39 (m, 1H), 3.29–3.16 (m, 3H), 2.86 (m, 2H), 2.72 (m, 2H), 2.32–2.25 (m, 4H, contains s, 2.26, 3H), 2.10 (m, 2H), 1.97 (m, 2H), 1.72 (p, *J* = 7.2 Hz, 2H), 1.60–1.14 (complex, 6H), 1.00 (s, 3H); ^{13}C NMR (126 MHz, DMSO-d_6) δ 175.1, 155.0, 137.0, 135.3, 132.4, 130.2, 129.7 (2C), 128.9 (2C), 126.0, 114.7, 112.8, 46.4, 45.4, 43.8, 42.3, 40.5, 38.1, 34.4, 30.4, 29.4, 26.4, 25.8, 25.6, 20.5, 20.4, 18.0. HRMS (FT-ICR, HESI) *m/z*: [M + H]$^+$ calcd for $C_{28}H_{36}NO_2S$ 450.2461, found 450.2458.

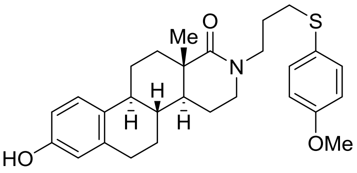

I16

3-Hydroxy-1,3,5-estratriene-derived D-Ring Lactam, I16

Following the general procedure G, **I16** was prepared as an off-white crystalline solid (44.0 mg, 0.0945 mmol, 63% yield, UPLC/HRMS purity: ≥99.5%). Purification was carried out by an automated MPLC system using a 12 g normal phase silica column with gradient elution from 0–80% EtOAc/hexanes. R_f = 0.38 (50% EtOAc/hexanes); mp 206–209 ˚C; IR (neat) 3301, 1618, 1606 cm^{-1}; ^{1}H NMR (400 MHz, DMSO-d_6) δ 9.00 (s, 1H), 7.32 (m, 2H), 7.05 (d, *J* = 8.4 Hz, 1H), 6.91 (m, 2H), 6.51 (dd, *J* = 8.4, 2.6 Hz, 1H), 6.44 (d, *J* = 2.6 Hz, 1H), 3.74 (s, 3H), 3.37 (m, 1H), 3.29–3.16 (m, 3H), 2.78 (m, 2H), 2.72 (m, 2H), 2.27 (m, 1H), 2.11 (m, 2H), 1.98 (m, 2H), 1.69 (p, *J* = 7.2 Hz, 2H), 1.59–1.16 (complex, 6H), 1.00 (s, 3H); ^{13}C NMR (126 MHz, DMSO-d_6) δ 175.1, 158.3, 155.0, 137.0, 132.2 (2C), 130.2, 125.97 (2C), 125.91, 114.8, 114.7, 112.8, 55.2, 46.4, 45.3, 43.8, 42.3, 40.5, 38.1, 34.4, 32.0, 29.4, 26.4, 25.8, 25.6, 20.4, 18.0. HRMS (FT-ICR, HESI) *m/z*: [M + H]$^+$ calcd for $C_{28}H_{36}NO_3S$ 466.2410, found 466.2408.

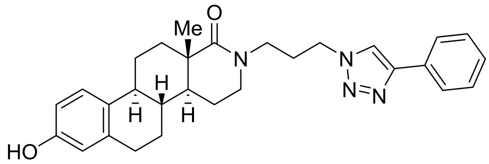

I17

3-Hydroxy-1,3,5-estratriene-derived D-Ring Lactam, I17

Following the general procedure H, **I17** was prepared as a white crystalline/amorphous solid (58.8 mg, 0.125 mmol, 63% yield, UPLC/HRMS purity: ≥99.5%). Purification was carried out by an automated MPLC system using a 12 g normal phase silica column with gradient elution from 30–85% EtOAc/hexanes. R_f = 0.30 (80% EtOAc/hexanes); mp 217–223 ˚C; IR (neat) 3363, 1593 cm^{-1}; ^{1}H NMR (400 MHz, DMSO-d_6) δ 9.00 (s, 1H), 8.60 (s, 1H), 7.84 (m, 2H), 7.44 (m, 2H), 7.32 (m, 1H), 7.04 (d, *J* = 8.5 Hz, 1H), 6.51 (dd, *J* = 8.4, 2.6 Hz, 1H), 6.44 (d, *J* = 2.6 Hz, 1H), 4.37 (t, *J* = 7.0 Hz, 1H), 3.39–3.35 (m, 2H), 3.29–3.21 (m, 2H), 2.71 (m, 2H), 2.25 (m, 1H), 2.16–2.06 (m, 5H), 1.98 (m, 2H), 1.59–1.17 (complex, 6H), 1.02 (s, 3H); ^{13}C NMR (126 MHz, DMSO-d_6) δ 175.4, 155.0, 146.3, 137.0, 130.4, 130.2, 128.9 (2C), 127.8, 126.0, 125.1 (2C), 121.4, 114.7, 112.8, 47.7, 46.3, 43.8, 43.7, 42.2, 40.5, 38.1, 34.3, 29.4, 27.2, 25.8, 25.6, 20.4, 18.0. HRMS (FT-ICR, HESI) *m/z*: $[M + H]^+$ calcd for $C_{29}H_{35}N_4O_2$ 471.2755, found 471.2752.

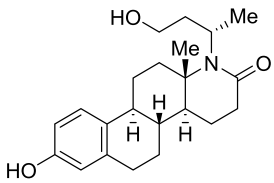

J2

3-Hydroxy-1,3,5-estratriene-derived D-Ring Lactam, J2

Following the general procedure F, (*S*)-3-azidobutanol (*S*)-**10** (34.6 mg, 0.300 mmol, 2.0 equiv) was reacted with estrone **5a** (40.5 mg, 0.150 mmol) to give **J2** as an off-white amorphous solid (36.7 mg, 0.103 mmol, 68% yield, UPLC/HRMS purity: 96.1%). Purification was carried out by an automated MPLC system using a 12 g normal phase silica column with gradient elution from 0–6% MeOH/CH_2Cl_2. R_f = 0.13 (4% MeOH/CH_2Cl_2); mp 248–255 ˚C; IR (neat) 3328, 3114, 1620 cm^{-1}; ^{1}H NMR (600 MHz, DMSO-d_6) δ 8.96 (s, 1H), 7.05 (d, *J* = 8.6 Hz, 1H), 6.52 (dd, *J* = 8.5, 2.6 Hz, 1H), 6.45 (d, *J* = 2.6 Hz, 1H), 4.33 (t, *J* = 5.0 Hz, 1H), 3.62 (m, 1H), 3.45–3.34 (m, 2H), 2.73 (m, 2H), 2.39–2.19 (complex, 5H), 2.12–2.02 (m, 2H), 1.86 (m, 2H), 1.55 (m, 1H), 1.44–1.33 (m, 2H), 1.30 (d, *J* = 6.5 Hz, 3H), 1.27–1.20 (m, 3H), 1.15 (s, 3H); ^{13}C NMR (151 MHz, DMSO-d_6) δ 168.8, 155.0, 136.9, 129.9, 125.8, 114.5, 112.8, 60.6, 58.5, 47.7, 46.6, 42.0, 39.3, 38.4, 36.9, 33.0, 29.2, 26.9, 25.9, 18.61, 18.56, 18.1. **Note**: APT has been included in the spectra section. HRMS (FT-ICR, HESI) *m/z*: $[M + H]^+$ calcd for $C_{22}H_{32}NO_3$ 358.2377, found 358.2370.

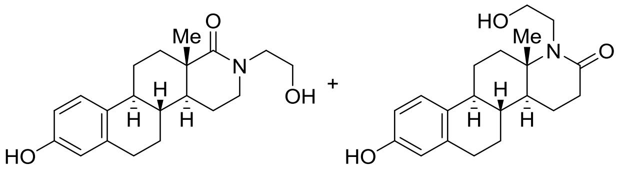

I6 J3

3-Hydroxy-1,3,5-estratriene-derived D-Ring Lactams, I6 and J3

Following the general procedure F, 2-azidoethanol **12** (26.1 mg, 0.300 mmol, 2.0 equiv) was reacted with estrone **5a** (40.6 mg, 0.150 mmol) to give a mixture of **I6**:**J3** as an off-white amorphous solid (46.3 mg, 0.141 mmol, 94% yield). Purification was carried out by an automated MPLC system using a 12 g normal phase silica column with gradient elution from 0–5% MeOH/CH_2Cl_2. A 30:70 regioisomeric ratio was observed by analytical HPLC of crude reaction mixture: Chiralpak IB, Daicel Chemical Industries, Ltd.; 0–30% EtOH/hexanes over 60 min; flow rate 1.0 mL/min; UV 220 nm; **I6** = 28.03 min, **J3** = 34.55 min. The mixture was subjected to a second purification using Chiralpak IB prep HPLC with gradient elution from 0–5% EtOH/hexanes over 60 min to give **I6** (36.1 mg, 0.110 mmol, 27% yield, UPLC/HRMS purity: 98.1%) as a white amorphous solid and **J3** (68.9 mg, 0.209 mmol, 52% yield, UPLC/HRMS purity: 99.0%) as a white amorphous solid. **I6** + **J3** mixture: R_f = 0.38 (5% MeOH/CH_2Cl_2); IR (neat) 3334, 1596, 1567 cm^{-1}. **Note**: ^{1}H and ^{13}C NMR spectra of mixture are included in the spectra section. **I6**: mp decomposed; IR (neat) 3479, 3309, 1595 cm^{-1}; ^{1}H NMR (400 MHz, DMSO-d_6) δ 9.03 (s, 1H), 7.06 (d, J = 8.5 Hz, 1H), 6.51 (dd, J = 8.4, 2.7 Hz, 1H), 6.44 (d, J = 2.6 Hz, 1H), 4.64 (m, 1H), 3.49–3.16 (complex, 5H), 2.72 (m, 2H), 2.28 (m, 1H), 2.17–2.07 (m, 2H), 1.97 (m, 2H), 1.60–1.14 (complex, 7H), 1.02 (s, 3H); ^{13}C NMR (126 MHz, DMSO-d_6) δ 175.2, 155.1, 137.0, 130.2, 126.0, 114.7, 112.8, 58.6, 49.2, 47.7, 43.8, 42.4, 40.5, 38.1, 34.5, 29.4, 25.8, 25.6, 20.5, 18.1. HRMS (FT-ICR, HESI) m/z: $[M + H]^+$ calcd for $C_{20}H_{28}NO_3$ 330.2064, found 330.2059. **J3**: mp 259–262 ˚C; IR (neat) 3332, 1566 cm^{-1}; ^{1}H NMR (400 MHz, DMSO-d_6) δ 9.02 (s, 1H), 7.06 (d, J = 8.5 Hz, 1H), 6.52 (dd, J = 8.4, 2.6 Hz, 1H), 6.44 (d, J = 2.6 Hz, 1H), 4.67 (t, J = 5.4 Hz, 1H), 3.58–3.45 (m, 2H), 3.33 (m, 1H), 3.13 (m, 1H), 2.72 (m, 2H), 2.41–2.15 (complex, 5H), 2.03 (m, 1H), 1.92 (m, 1H), 1.59 (m, 1H), 1.49–1.35 (m, 2H), 1.30–1.19 (m, 3H), 1.11 (s, 3H); ^{13}C NMR (126 MHz, DMSO-d_6) δ 169.7, 155.1, 137.0, 129.9, 126.1, 114.6, 112.9, 60.0, 59.2, 46.9, 43.4, 42.0, 39.6, 37.4, 31.3, 29.4, 26.7, 25.8, 18.9, 18.6. HRMS (FT-ICR, HESI) m/z: $[M + H]^+$ calcd for $C_{20}H_{28}NO_3$ 330.2064, found 330.2059.

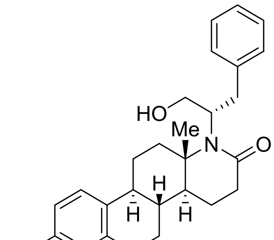

J4

3-Hydroxy-1,3,5-estratriene-derived D-Ring Lactam, J4

Following the general procedure F, (S)-2-azido-3-phenylpropanol (S)-**14** (53.2 mg, 0.300 mmol, 2.0 equiv) was reacted with estrone **5a** (40.6 mg, 0.150 mmol) to give **J4** as a white amorphous solid (57.3 mg, 0.137 mmol, 91% yield, UPLC/HRMS purity: 96.8%). Purification was carried out by an automated MPLC system using a 12 g normal phase silica column with gradient elution from 0–80% EtOAc/hexanes. R_f = 0.21 (50% EtOAc/hexanes); mp 246–253 ˚C; IR (neat) 3247, 1602 cm^{-1}; ^{1}H NMR (400 MHz, DMSO-d_6) δ 9.01 (s, 1H), 7.27 (m, 2H), 7.17 (3H), 7.01 (d, J = 8.5 Hz, 1H), 6.50 (dd, J = 8.4, 2.6 Hz, 1H), 6.42 (d, J = 2.6 Hz, 1H), 4.98 (m, 1H), 3.72 (m, 2H), 3.50 (m, 1H), 3.27 (m, 1H), 3.09 (dd, J = 13.2, 5.9 Hz, 1H), 2.67 (m, 2H), 2.43–2.24 (m, 3H), 2.16 (m, 1H), 2.07 (m, 1H), 1.97 (m, 1H), 1.83 (m, 1H), 1.61 (m, 1H), 1.39 (m, 1H), 1.26–1.04 (m, 4H), 0.47 (s, 3H); ^{13}C NMR (126 MHz, DMSO-d_6) δ 170.6, 155.1, 140.5, 137.0, 129.8, 129.5 (2C), 128.2 (2C), 126.1, 126.0, 114.6, 112.8, 63.4, 60.4, 59.7, 47.6, 42.0, 39.6, 37.1, 34.4, 32.7, 29.3, 26.7, 25.9, 18.5, 17.6. HRMS (FT-ICR, HESI) m/z: $[M + H]^+$ calcd for $C_{27}H_{34}NO_3$ 420.2533, found 420.2525.

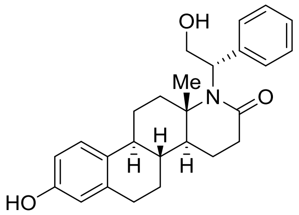

J5

3-Hydroxy-1,3,5-estratriene-derived D-Ring Lactam, J5

Following the general procedure F, (*S*)-2-azido-2-phenylethanol (*S*)-**15** (64.6 mg, 0.396 mmol, 2.0 equiv was reacted with estrone **5a** (54.5 mg, 0.201 mmol) to give **J5** as a white amorphous solid (41.5 mg, 0.102 mmol, 51% yield, UPLC/HRMS purity: ≥99.5%). Purification was carried out by an automated MPLC system using a 12 g normal phase silica column with gradient elution from 0–2% MeOH/CH_2Cl_2. R_f = 0.23 (2% MeOH/CH_2Cl_2); mp 254–259 ˚C; IR (neat) 3233, 1734 cm^{-1}; ^{1}H NMR (400 MHz, $CDCl_3$) δ 9.02 (s, 1H), 7.36 (d, *J* = 7.7 Hz, 2H), 7.25 (m, 2H), 7.15 (m, 1H), 7.07 (d, *J* = 8.5 Hz, 1H), 6.53 (dd, *J* = 8.4, 2.7 Hz, 1H), 6.45 (d, *J* = 2.6 Hz, 1H), 5.02 (t, *J* = 5.4 Hz, 1H), 4.16 (m, 1H), 4.03 (m, 1H), 2.74 (m, 1H), 2.43–2.27 (m, 5H), 2.07–2.05 (m, 1H), 1.99–1.92 (m, 1H), 1.78 (m, 1H), 1.60–1.44 (m, 2H), 1.33–1.21 (m, 4H), 1.14 (s, 3H); ^{13}C NMR (101 MHz, $CDCl_3$) δ 170.1, 155.1, 141.0, 137.0, 129.9, 127.5, 127.2, 126.0, 125.7, 114.6, 112.8, 65.4, 64.9, 61.1, 59.2, 47.8, 42.0, 37.5, 32.5, 29.3, 27.0, 26.0, 19.2, 18.6, 15.1. **Note**: Missing one carbon signal due to signal overlap. APT has been included in the spectra section. HRMS (FT-ICR, HESI) *m/z*: $[M + H]^+$ calcd for $C_{26}H_{32}NO_3$ 406.2377, found 406.2370.

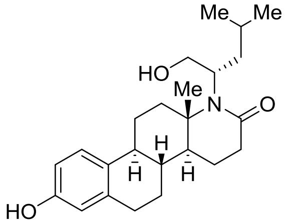

J6

3-Hydroxy-1,3,5-estratriene-derived D-Ring Lactam, J6

Following the general procedure F, (*S*)-2-azido-4-methylpentanol (*S*)-**16** (43.0 mg, 0.300 mmol, 2.0 equiv) was reacted with estrone **5a** (0.0411 mg, 0.152 mmol to give **J6** as a white amorphous solid (50.6 mg, 0.131 mmol, 86% yield, UPLC/HRMS purity: ≥99.5%). Purification was carried out by an automated MPLC system using a 12 g normal phase silica column with gradient elution from 0–80% EtOAc/hexanes. R_f = 0.25 (50% EtOAc/hexanes); mp 233–236 ˚C; IR (neat) 3152, 1598, 1571 cm^{-1}; ^{1}H NMR (400 MHz, DMSO-d_6) δ 9.02 (s, 1H), 7.06 (d, *J* = 8.4 Hz, 1H), 6.52 (dd, *J* = 8.4, 2.6 Hz, 1H), 6.44 (d, *J* = 2.6 Hz, 1H), 4.96 (dd, *J* = 6.7, 4.4 Hz, 1H), 3.75 (m, 1H), 3.56 (m, 1H), 3.42 (m, 1H), 2.73 (m, 2H), 2.40–2.15 (complex, 6H), 2.03 (m, 1H), 1.88 (m, 1H), 1.64 (m, 2H), 1.48–1.15 (complex 9H, contains s, 1.15, 3H), 0.90 (dd, *J* = 18.9, 6.5 Hz, 6H); ^{13}C NMR (126 MHz, DMSO-d_6) δ 170.2, 155.1, 136.9, 129.9, 125.9, 114.6, 112.8, 63.7, 60.7, 54.9, 47.5, 41.9, 39.5, 37.3, 32.5, 29.3, 26.9, 25.9, 25.4, 23.7, 22.3, 18.9, 18.5. **Note**: Missing one carbon signals due to signal overlap. APT has been included in the spectra section. HRMS (FT-ICR, HESI) *m/z*: $[M + H]^+$ calcd for $C_{24}H_{36}NO_3$ 386.2690, found 386.2683.

Supplementary Figures

Supplementary Figure 16. ^{1}H NMR spectrum in $CDCl_3$, 400 MHz, of **S1**.

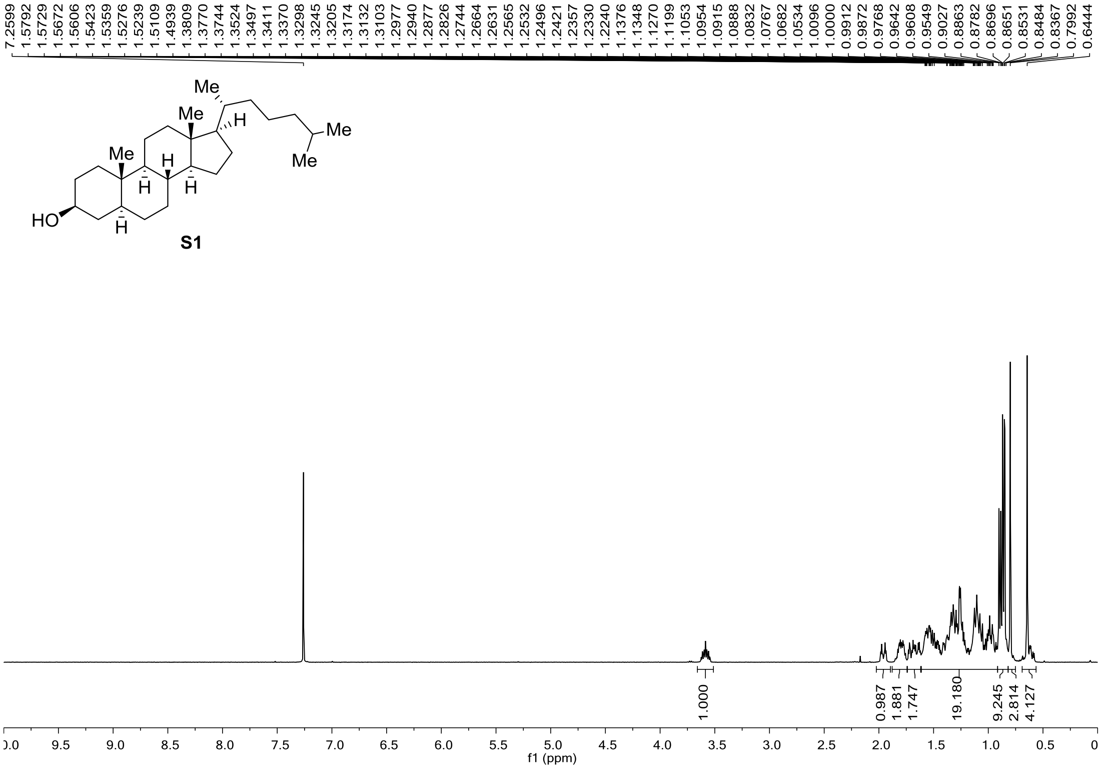

Supplementary Figure 17. ^{13}C NMR spectrum in $CDCl_3$, 101 MHz, of **S1**.

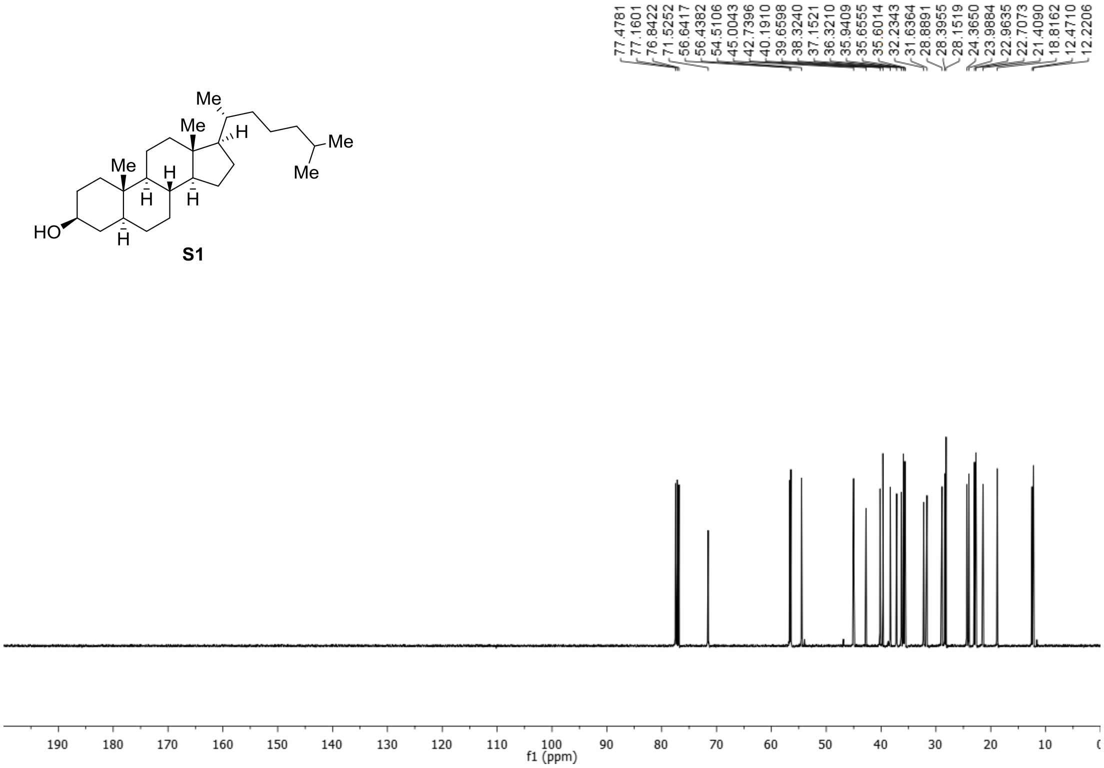

Supplementary Figure 18. ^{1}H NMR spectrum in $CDCl_3$, 400 MHz, of **1**.

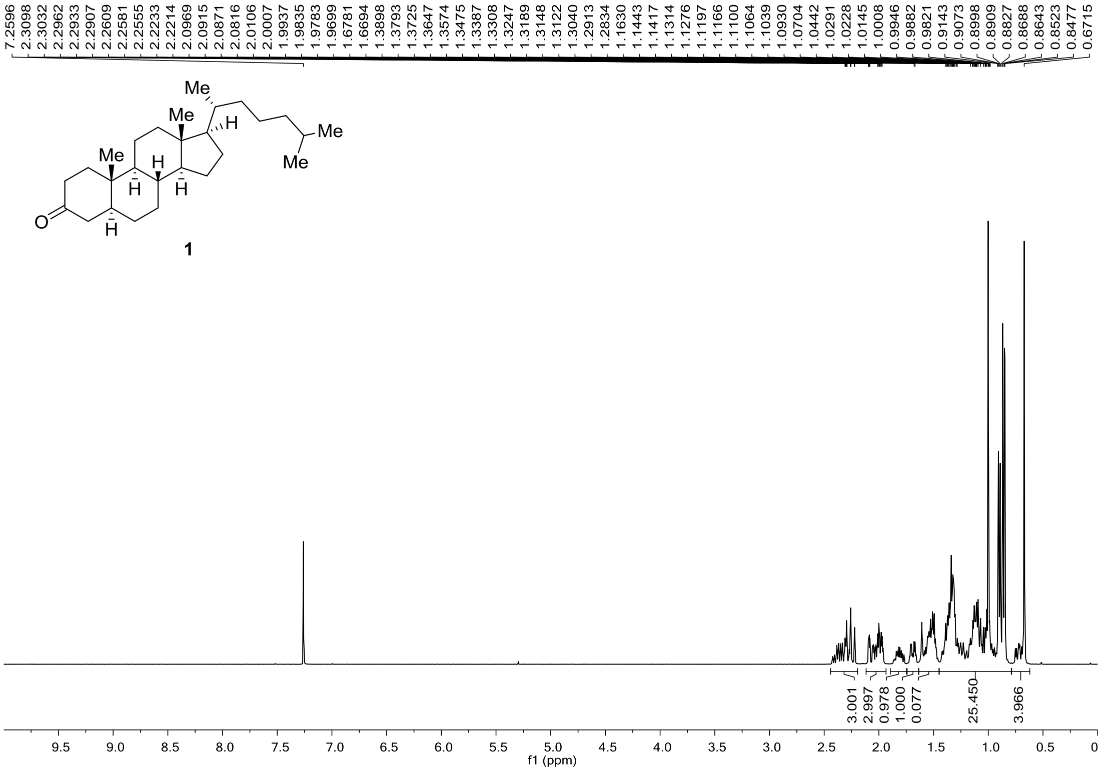

Supplementary Figure 19. ^{13}C NMR (APT) spectrum in $CDCl_3$, 101 MHz, of **1**.

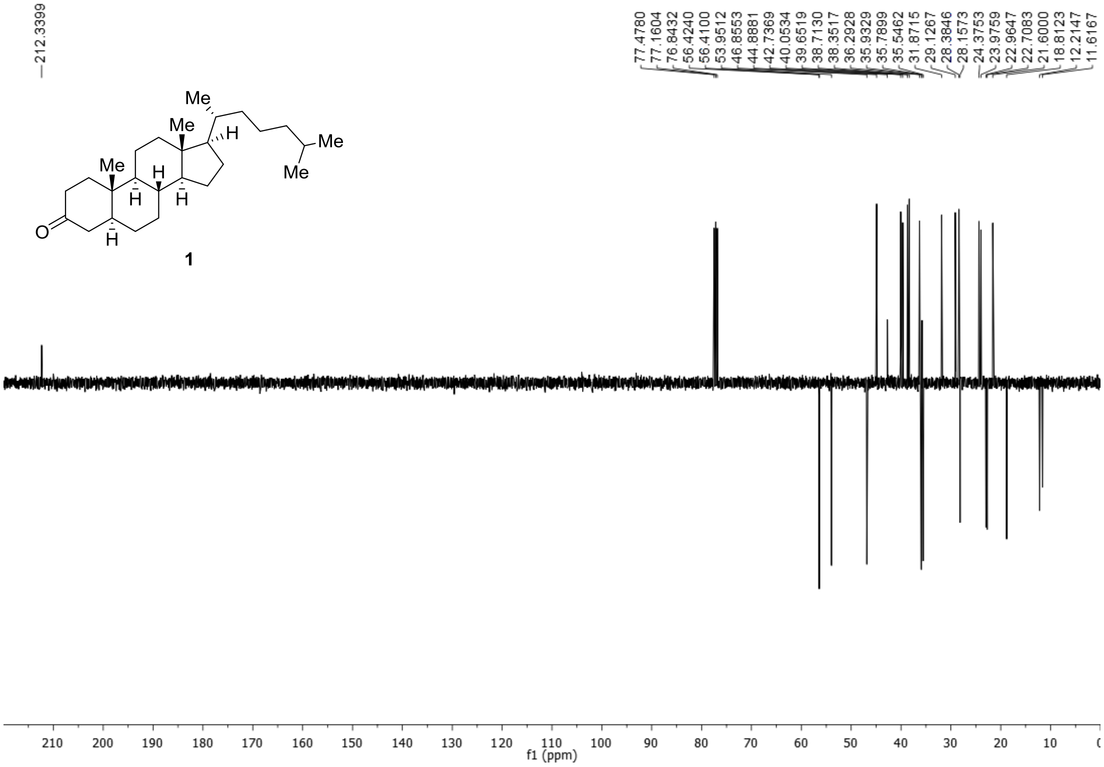

Supplementary Figure 20. ^{1}H NMR spectrum in $CDCl_3$, 400 MHz, of **2**.

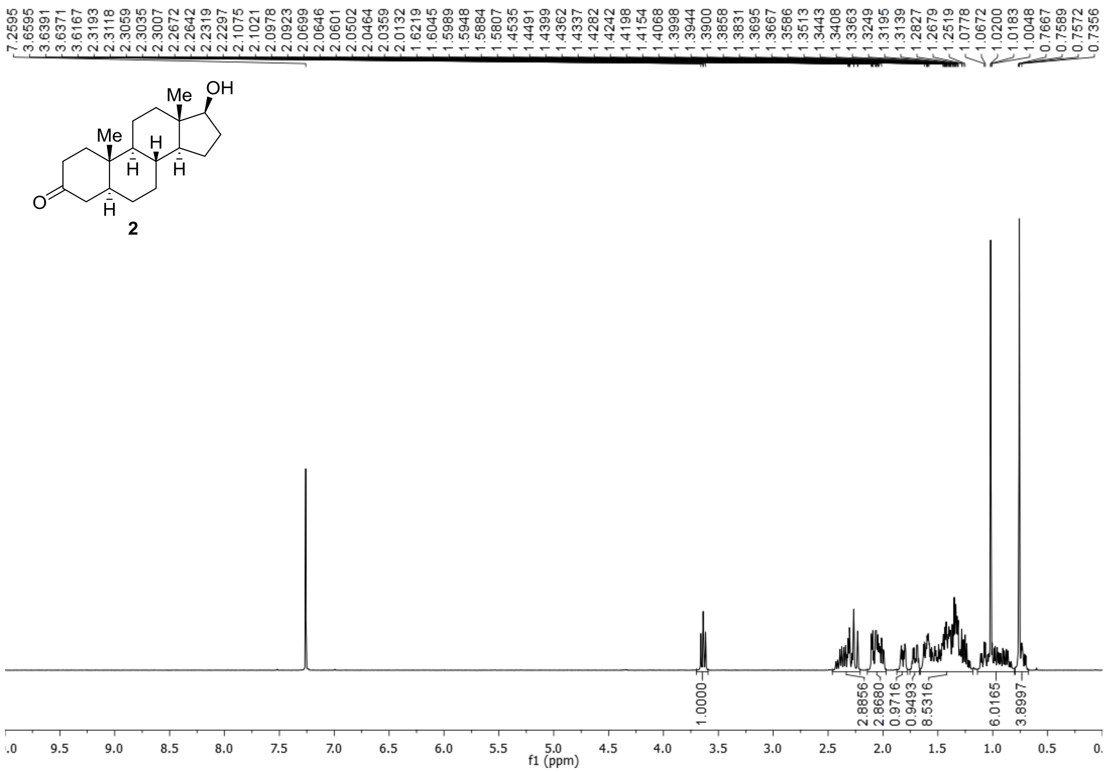

Supplementary Figure 21. ^{13}C NMR spectrum in $CDCl_3$, 101 MHz, of **2**.

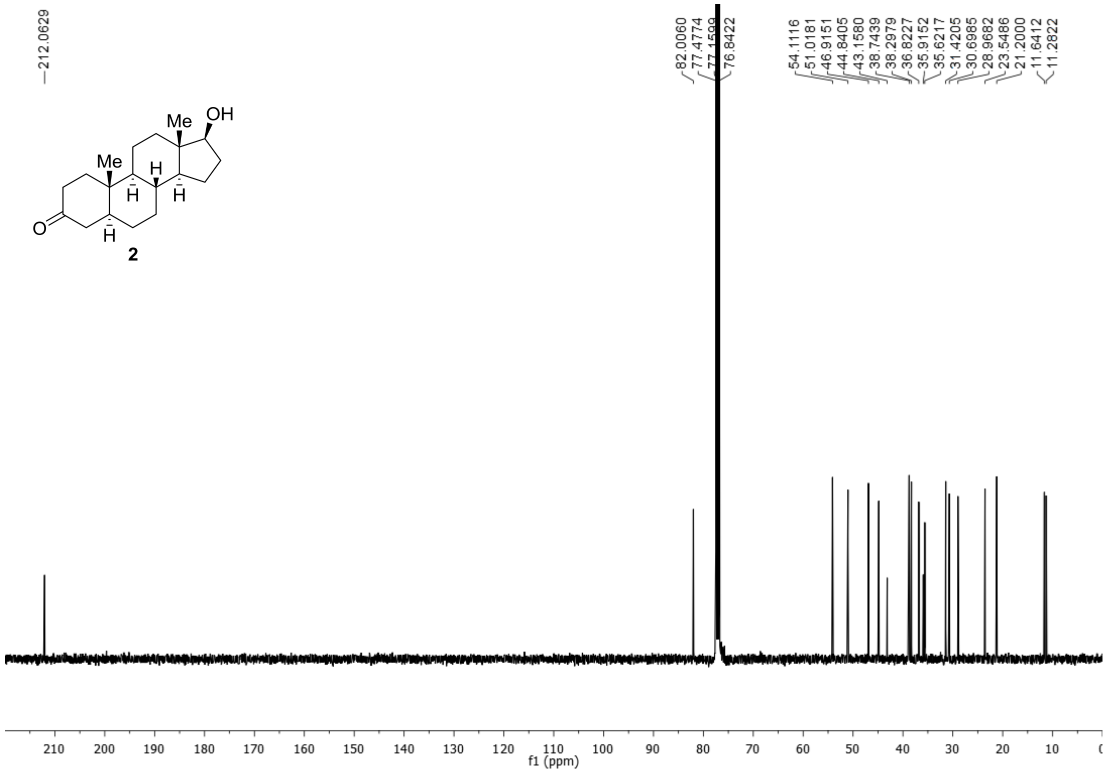

Supplementary Figure 22. ^{1}H NMR spectrum in $CDCl_3$, 400 MHz, of **3**.

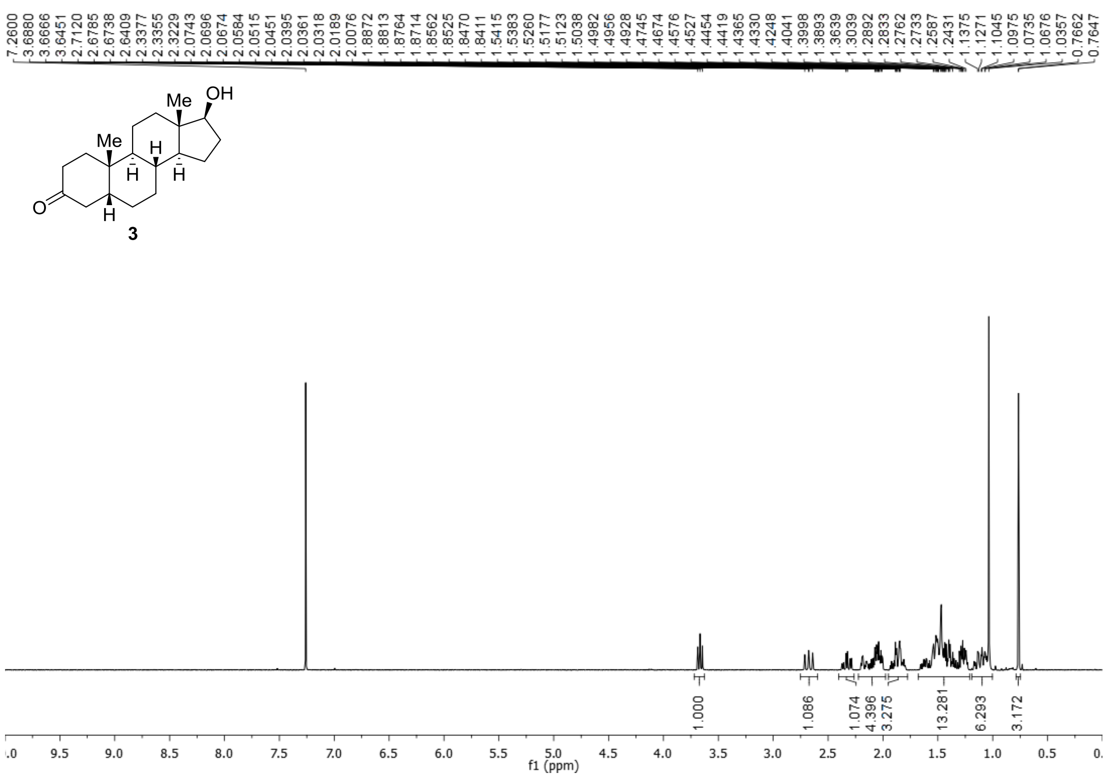

Supplementary Figure 23. ^{13}C NMR spectrum in $CDCl_3$, 101 MHz, of **3**.

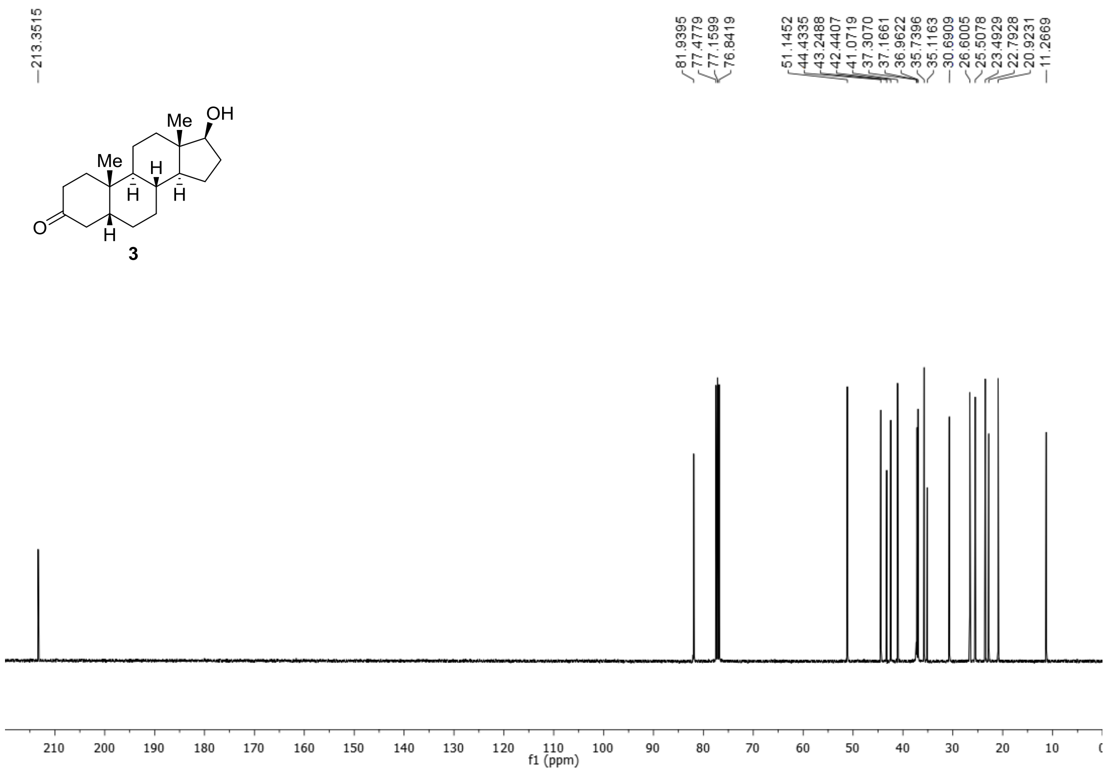

Supplementary Figure 24. ^{1}H NMR spectrum in $CDCl_3$, 400 MHz, of **6**.

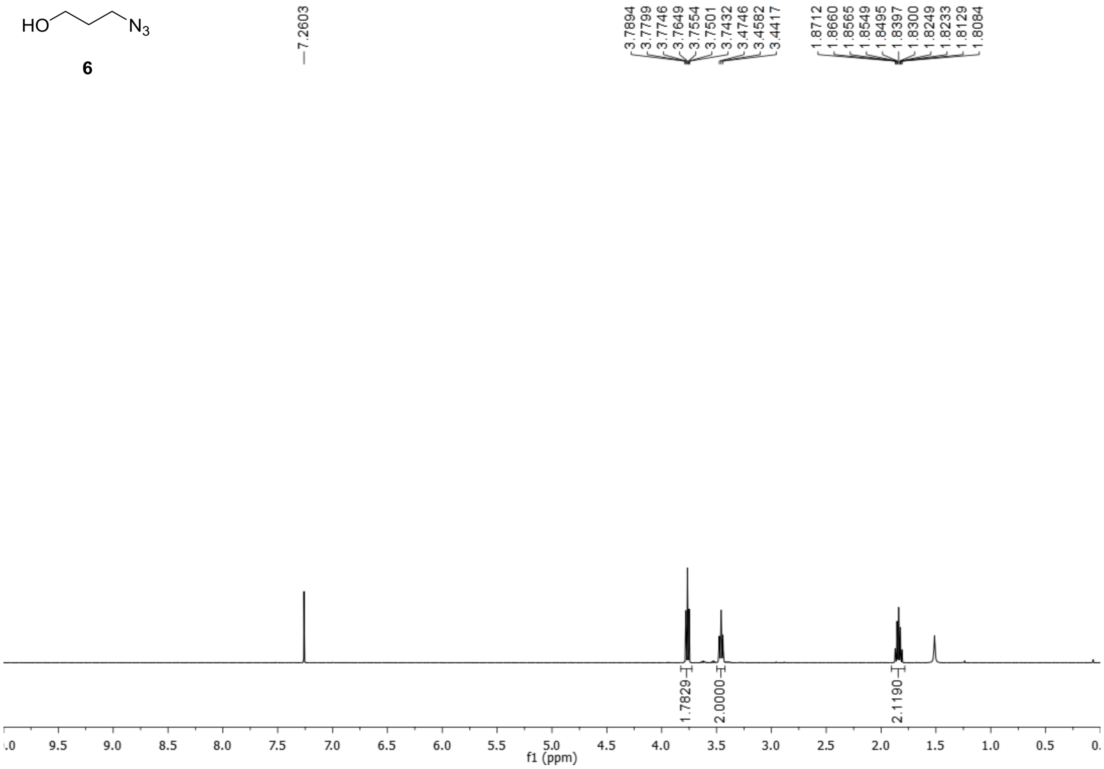

Supplementary Figure 25. ^{13}C NMR spectrum in $CDCl_3$, 101 MHz, of **6**.

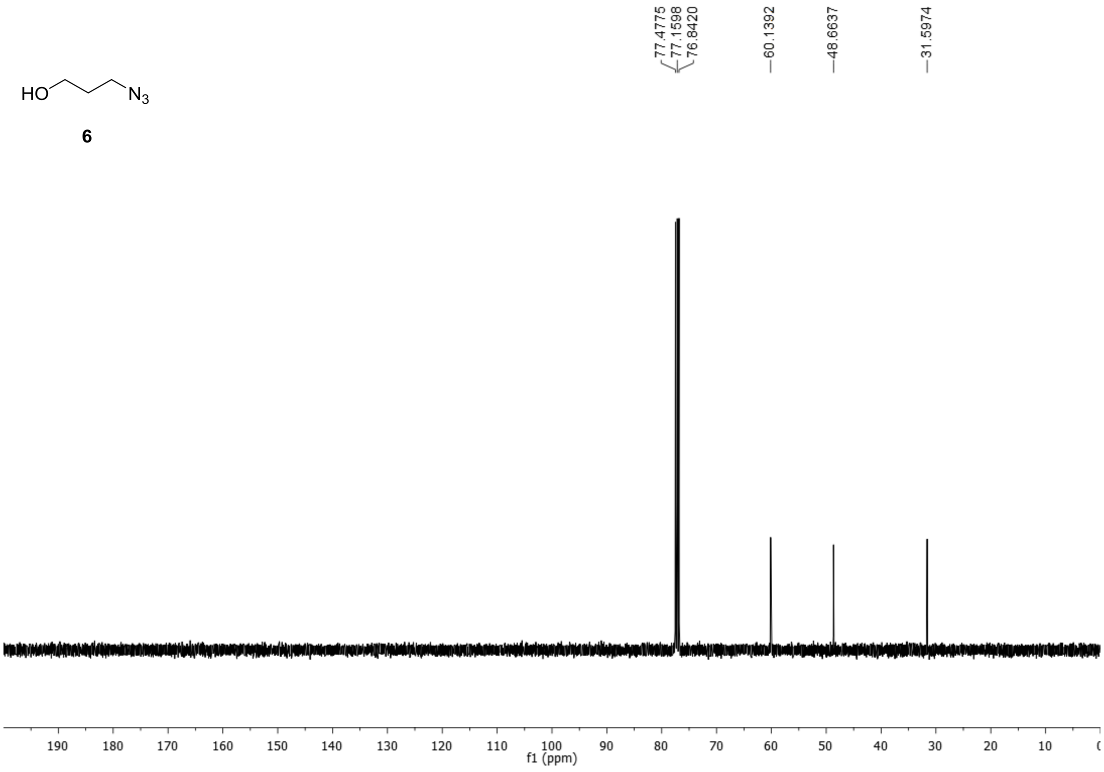

Supplementary Figure 26. ^{1}H NMR spectrum in $CDCl_3$, 400 MHz, of **S2**.

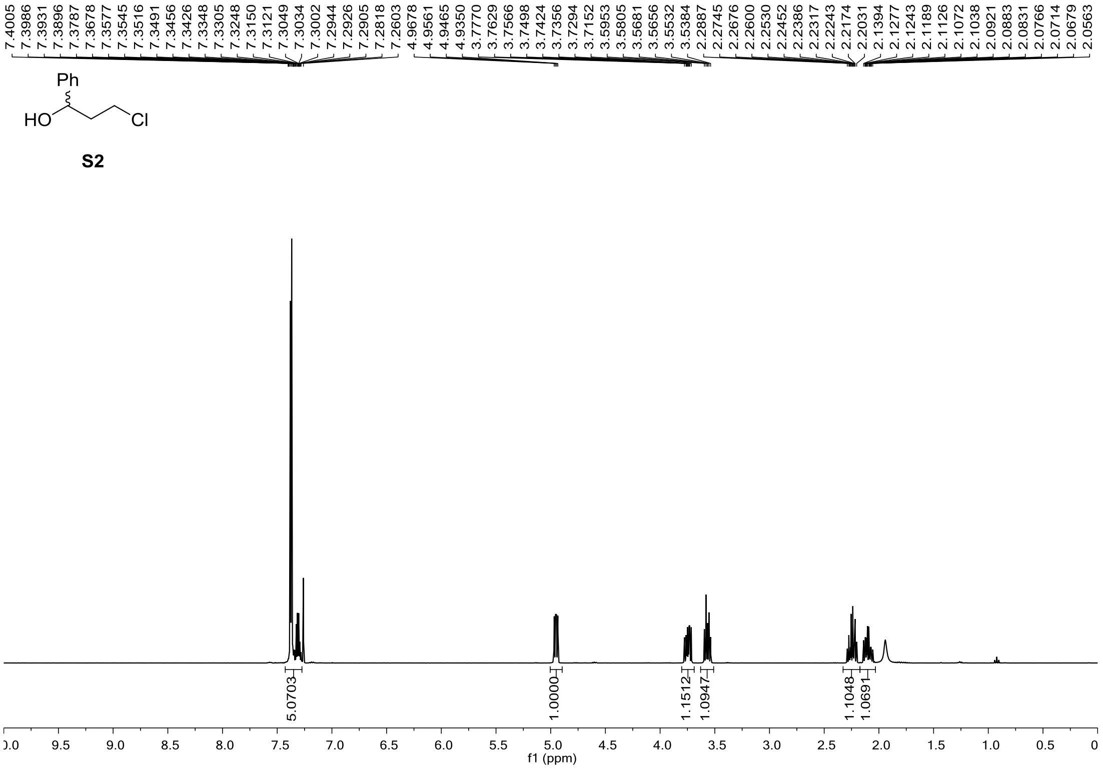

Supplementary Figure 27. ^{13}C NMR spectrum in $CDCl_3$, 101 MHz, of **S2**.

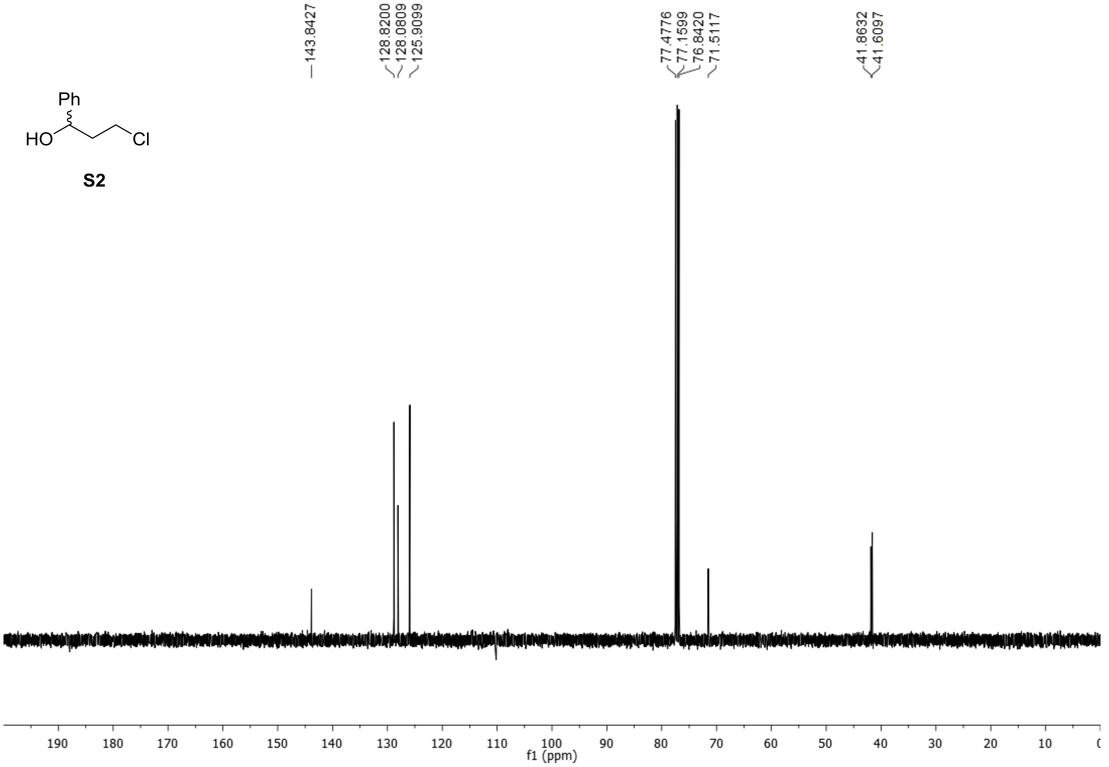

Supplementary Figure 28. ^{1}H NMR spectrum in $CDCl_3$, 400 MHz, of (±)-**7**.

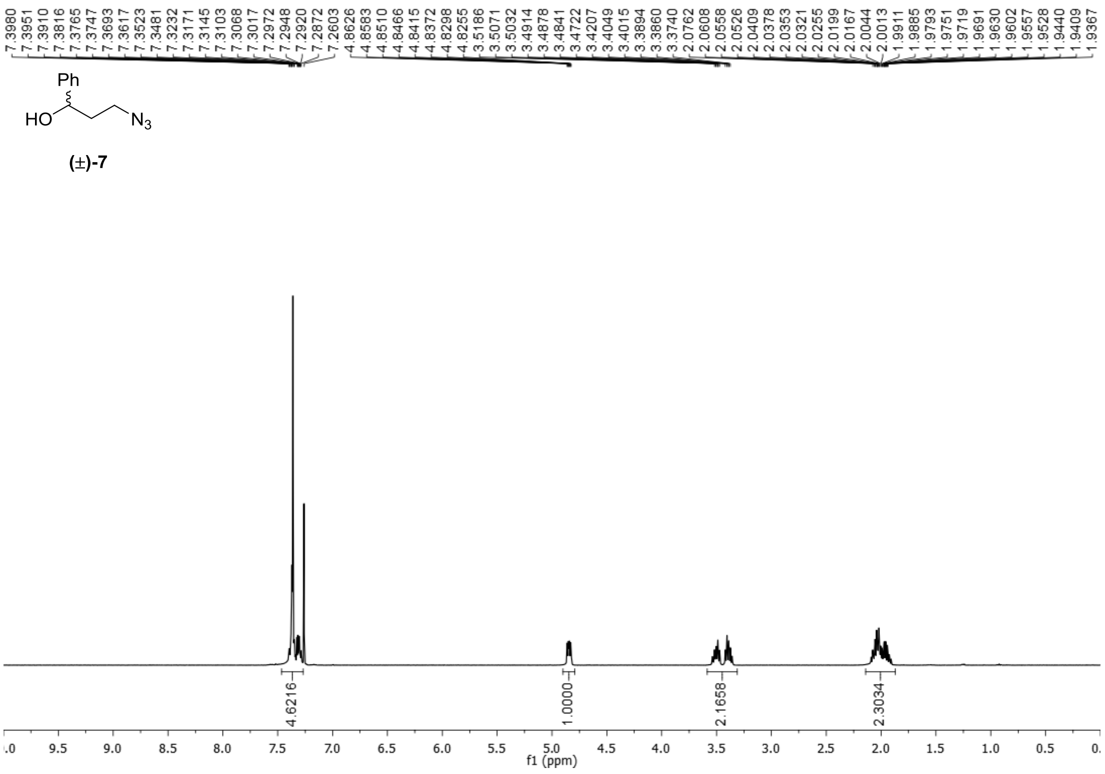

Supplementary Figure 29. ^{13}C NMR spectrum in $CDCl_3$, 101 MHz, of (±)-**7**.

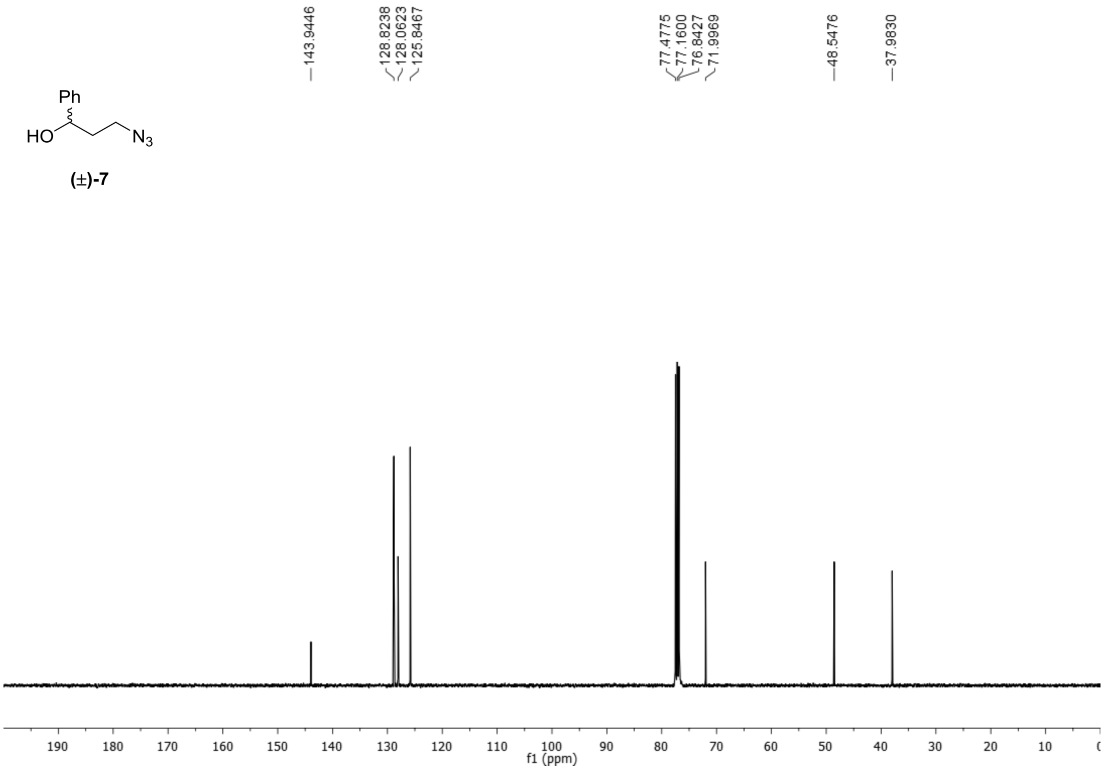

Supplementary Figure 30. ^{1}H NMR spectrum in $CDCl_3$, 400 MHz, of (*R*)-**7**.

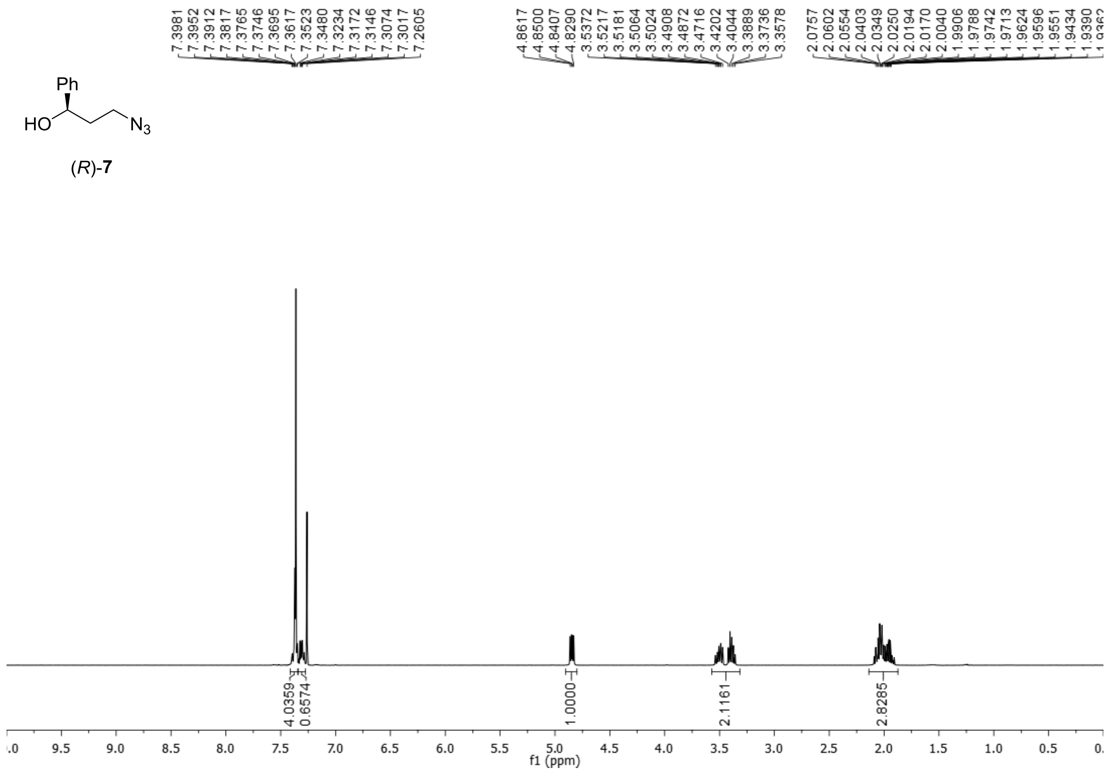

Supplementary Figure 31. ^{13}C NMR spectrum in $CDCl_3$, 101 MHz, of (*R*)-**7**.

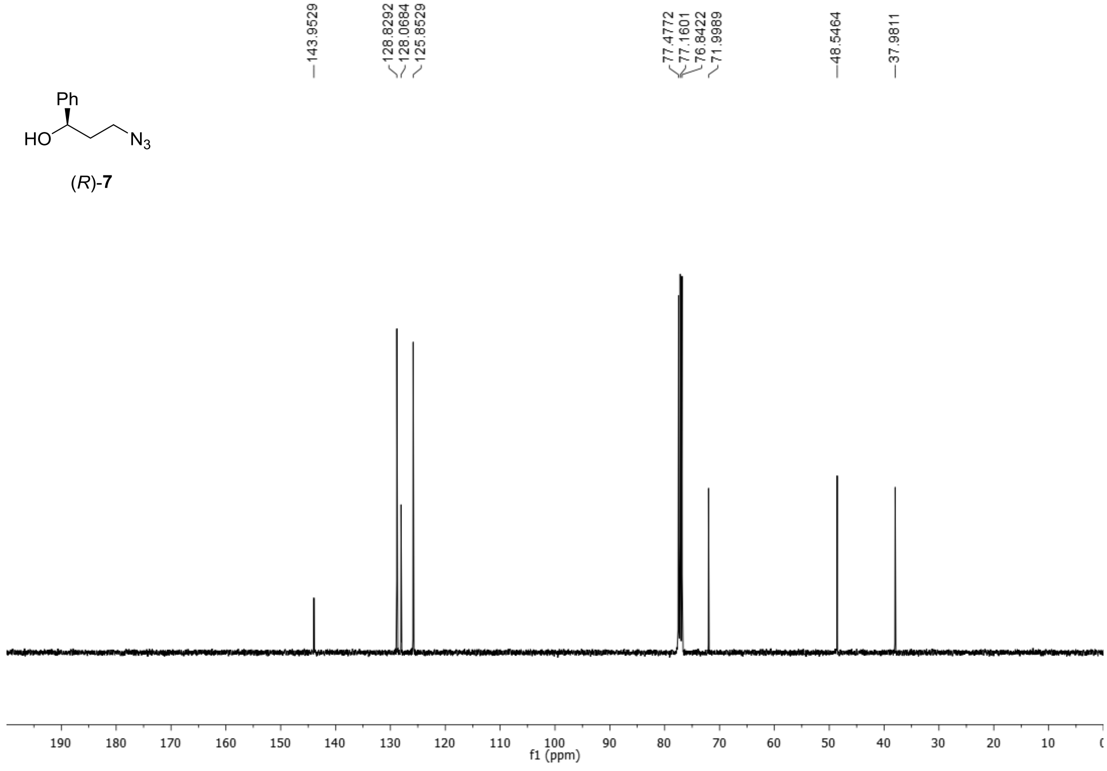

Supplementary Figure 32. ^{1}H NMR spectrum in $CDCl_3$, 400 MHz, of (*S*)-**7**.

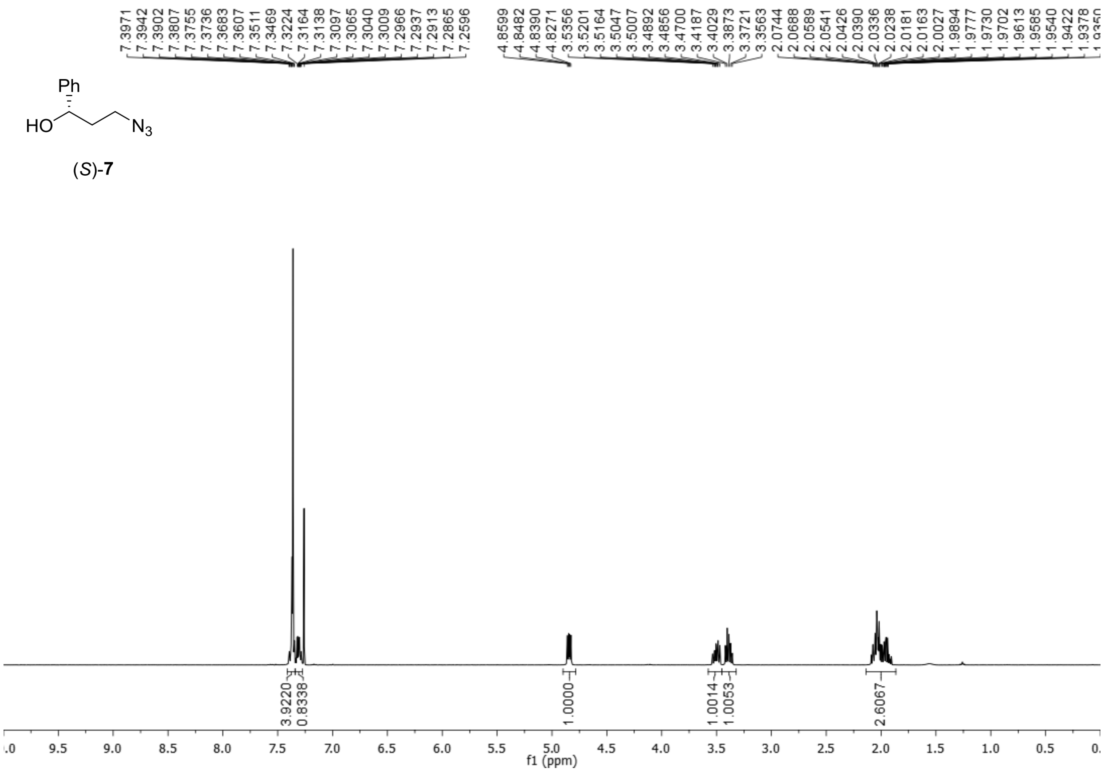

Supplementary Figure 33. ^{13}C NMR spectrum in $CDCl_3$, 101 MHz, of (*S*)-**7**.

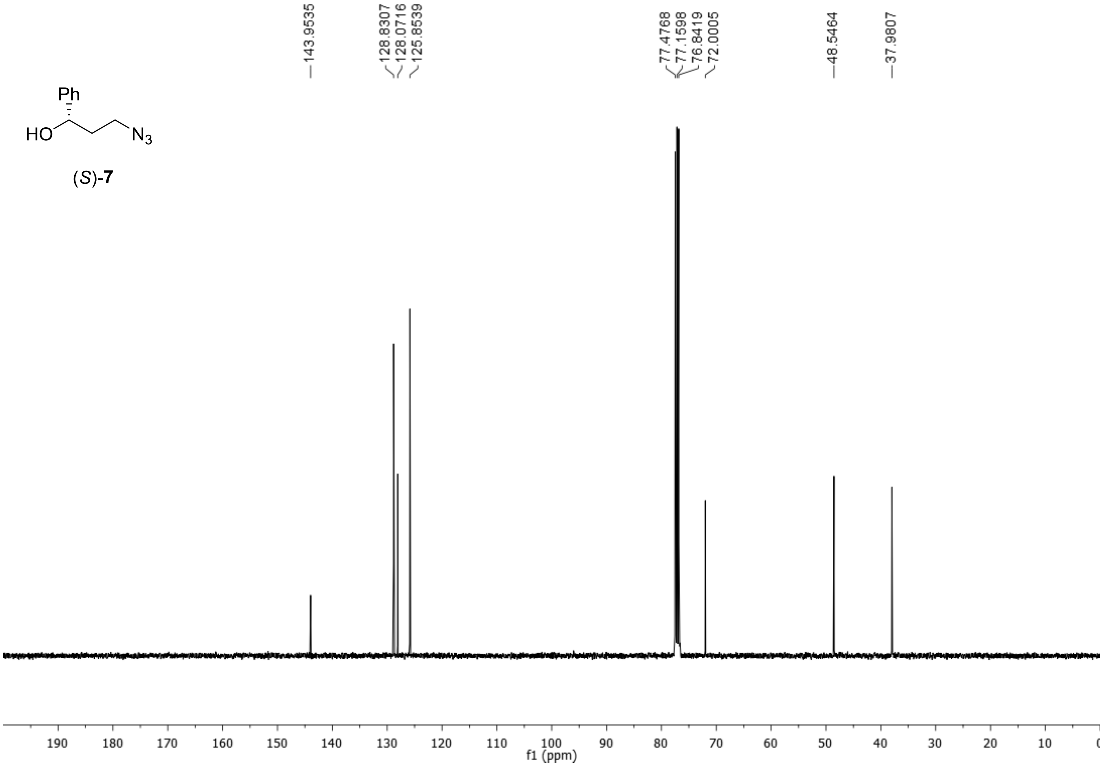

Supplementary Figure 34. ^{1}H NMR spectrum in $CDCl_3$, 400 MHz, of (*R*)-**8**.

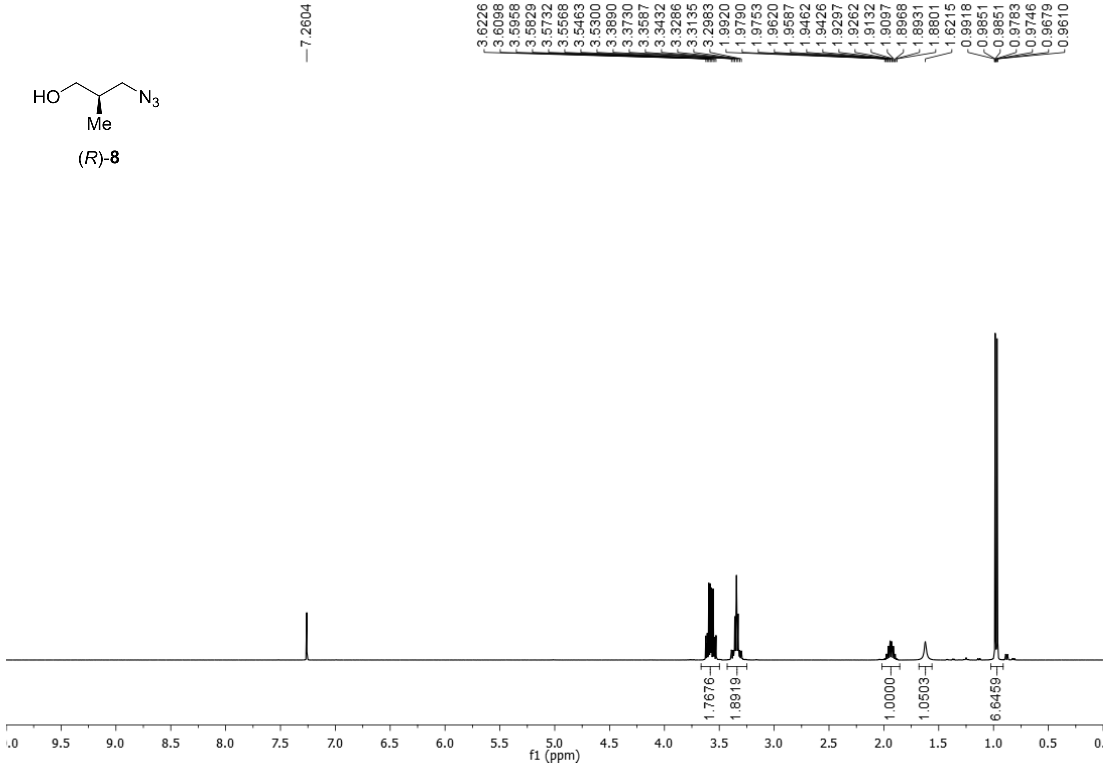

Supplementary Figure 35. ^{13}C NMR spectrum in $CDCl_3$, 101 MHz, of (*R*)-**8**.

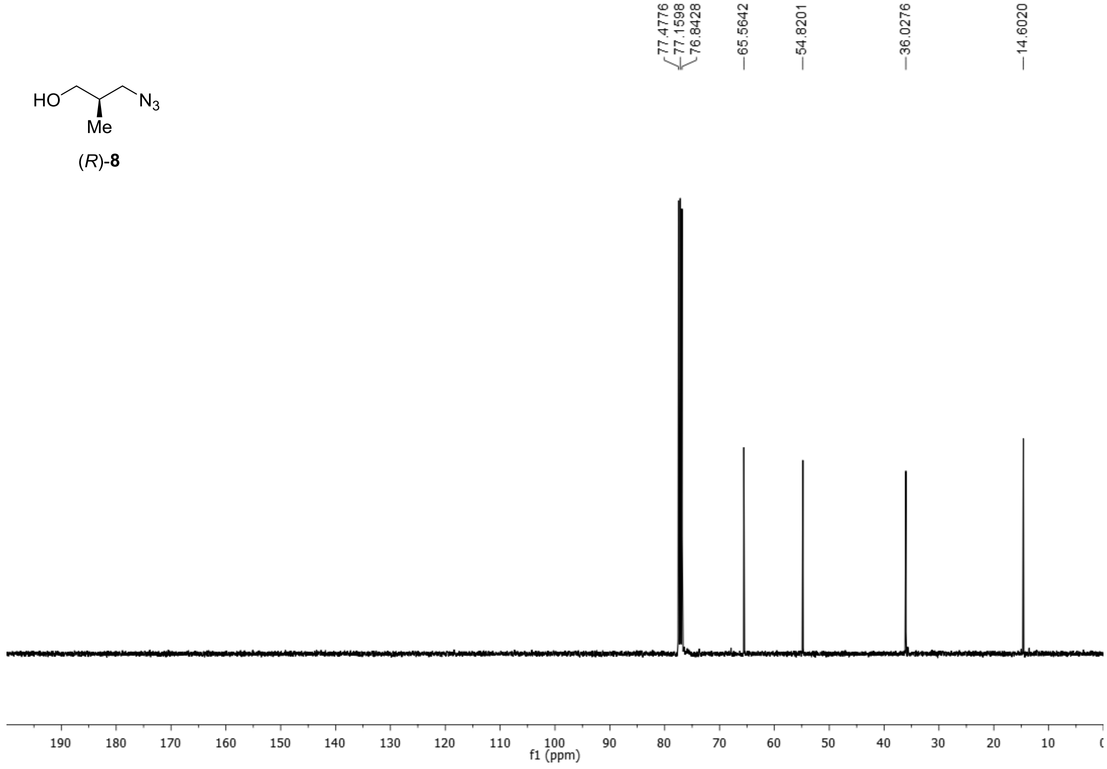

Supplementary Figure 36. ^{1}H NMR spectrum in $CDCl_3$, 400 MHz, of (*S*)-**8**.

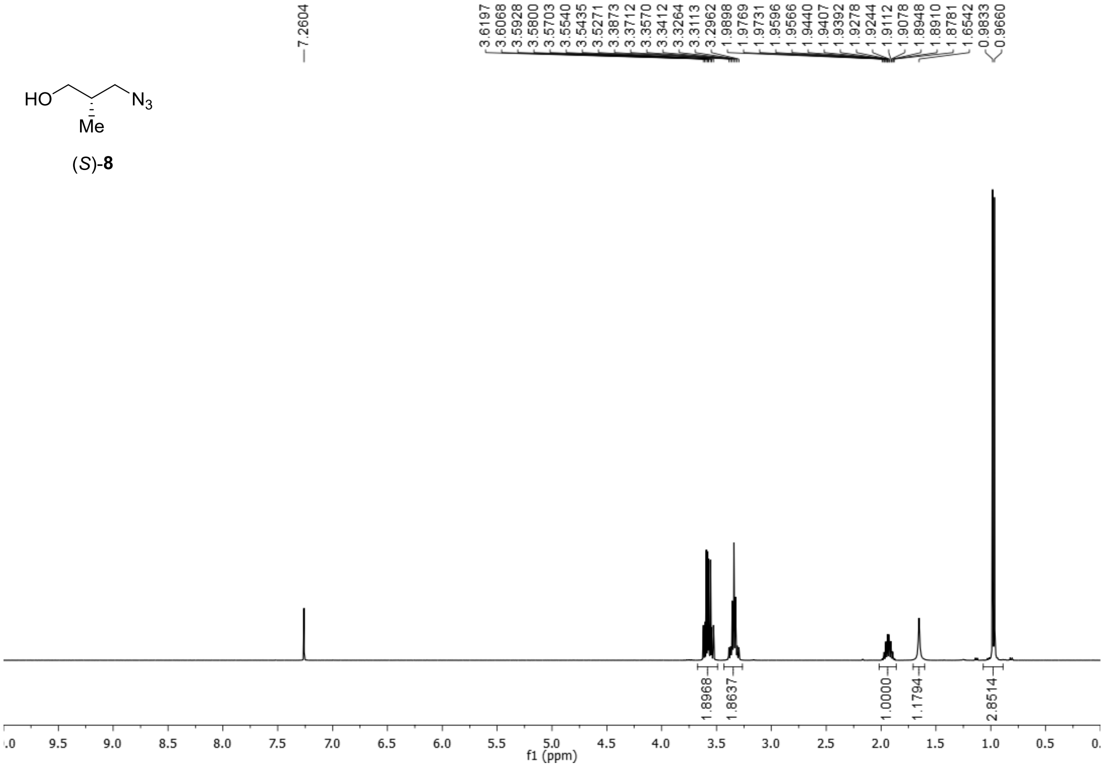

Supplementary Figure 37. ^{13}C NMR spectrum in $CDCl_3$, 101 MHz, of (*S*)-**8**.

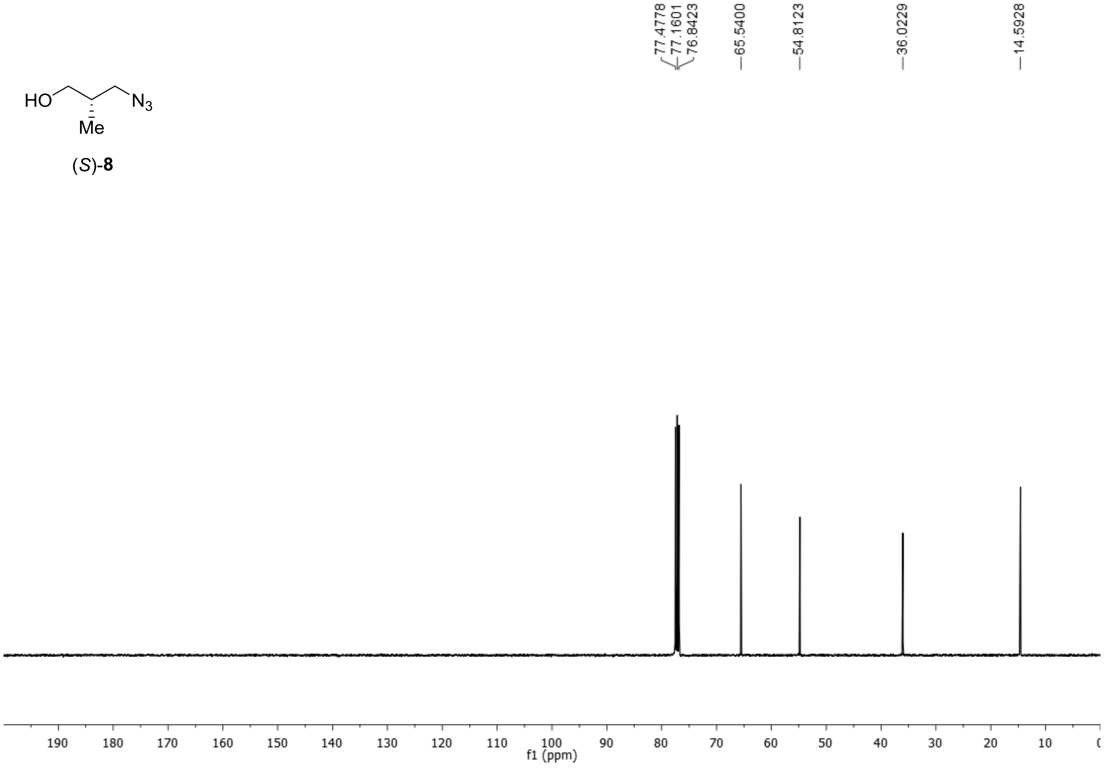

Supplementary Figure 38. ^{1}H NMR spectrum in $CDCl_3$, 400 MHz, of (*R*)-**9**.

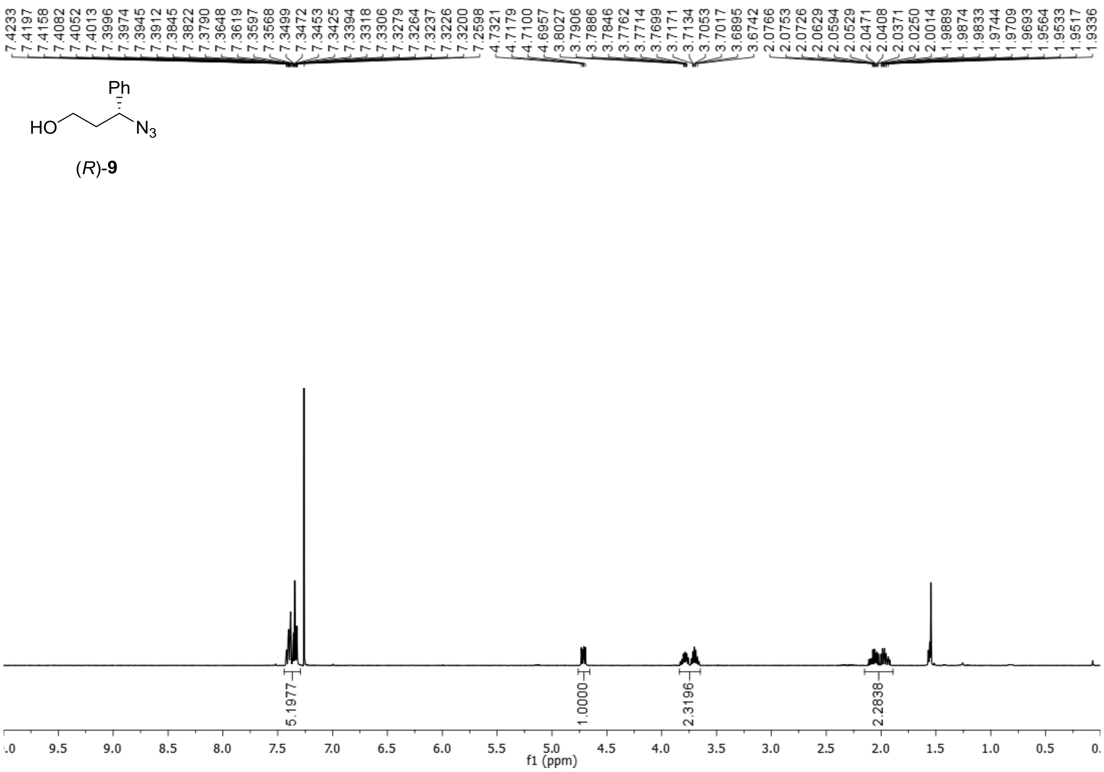

Supplementary Figure 39. ^{13}C NMR spectrum in $CDCl_3$, 101 MHz, of (*R*)-**9**.

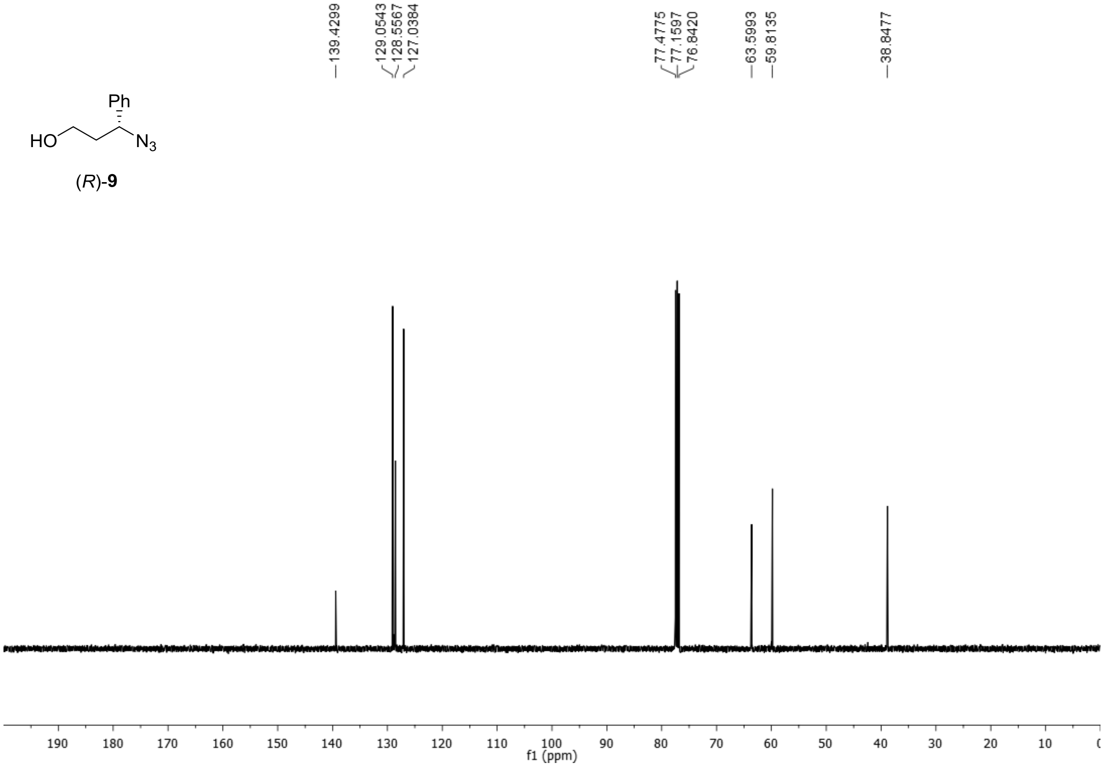

Supplementary Figure 40. ^{1}H NMR spectrum in $CDCl_3$, 400 MHz, of **S3**.

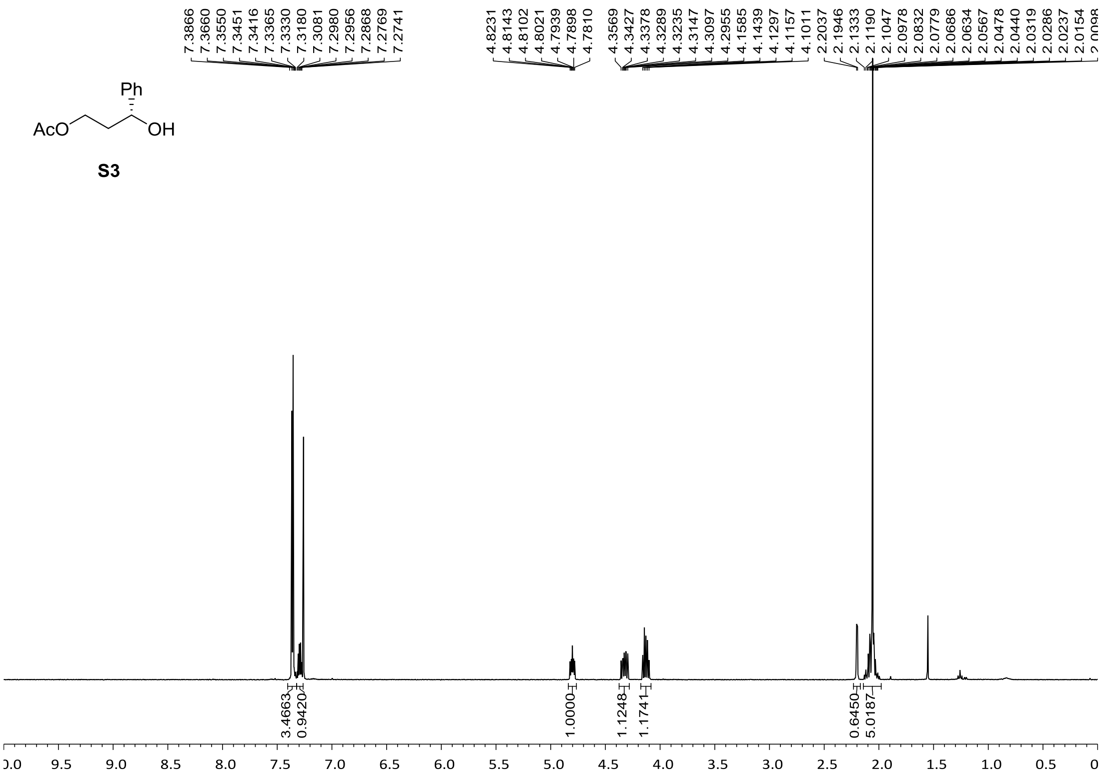

Supplementary Figure 41. ^{13}C NMR spectrum in $CDCl_3$, 101 MHz, of **S3**.

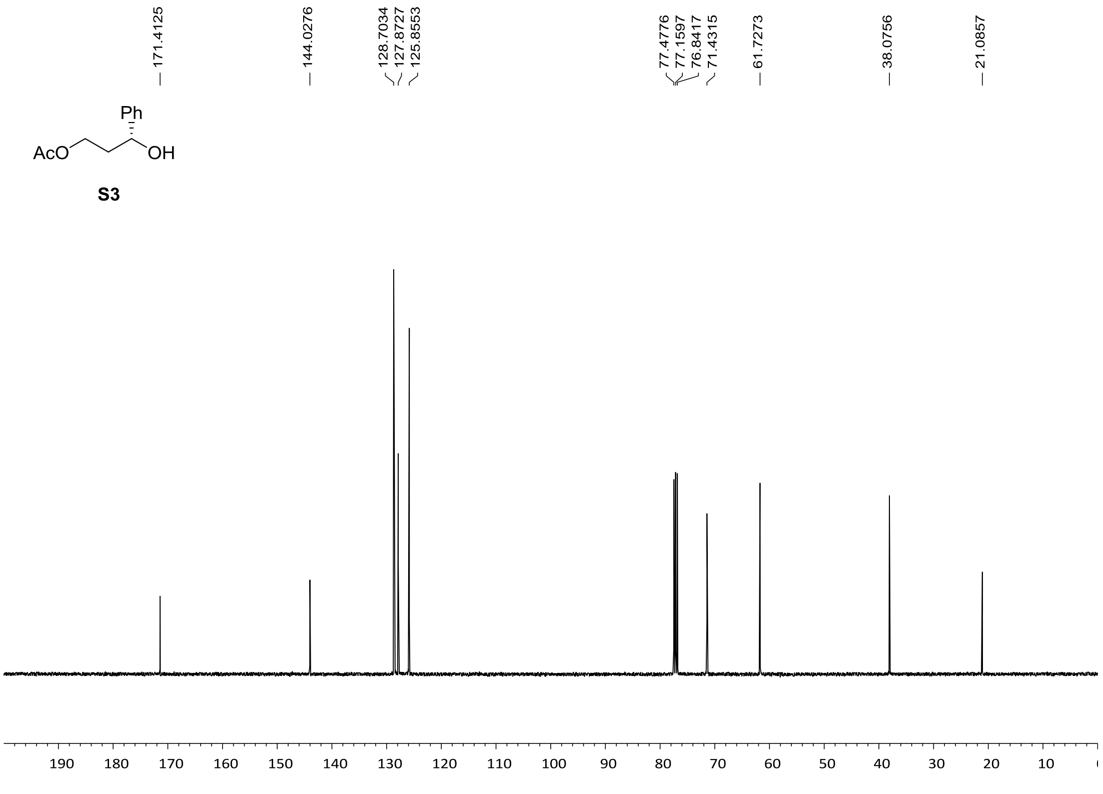

Supplementary Figure 42. ^{1}H NMR spectrum in $CDCl_3$, 400 MHz, of (*S*)-**9**.

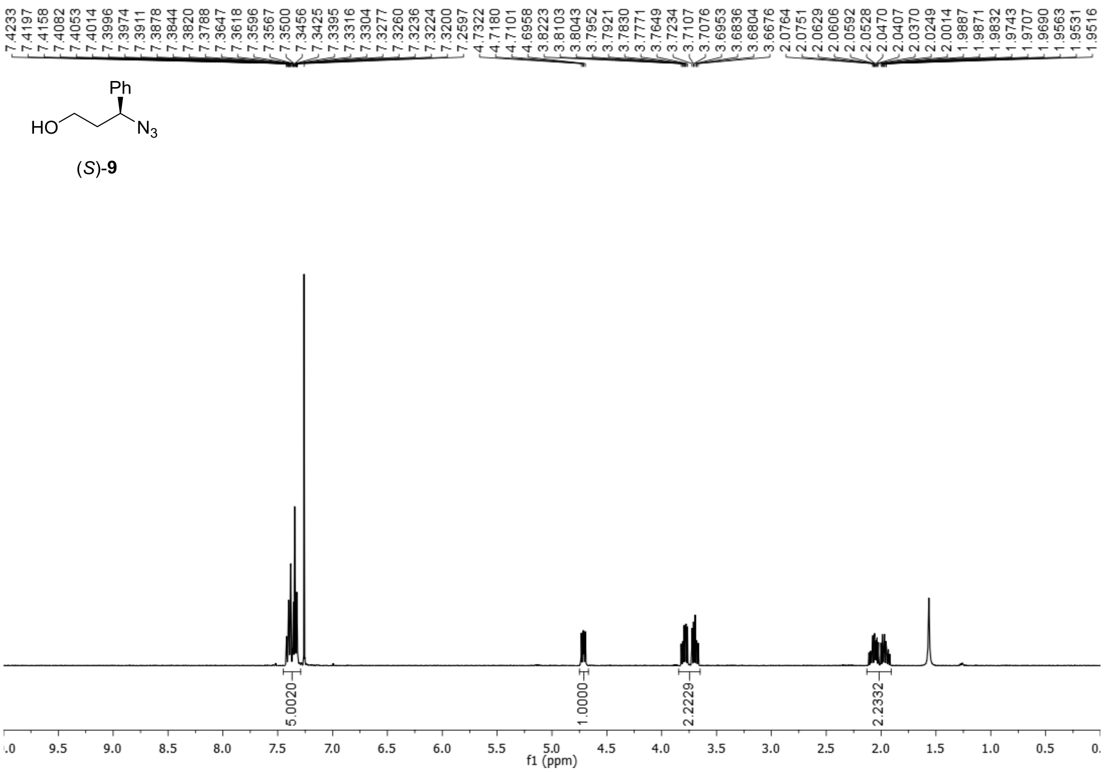

Supplementary Figure 43. ^{13}C NMR spectrum in $CDCl_3$, 101 MHz, of (*S*)-**9**.

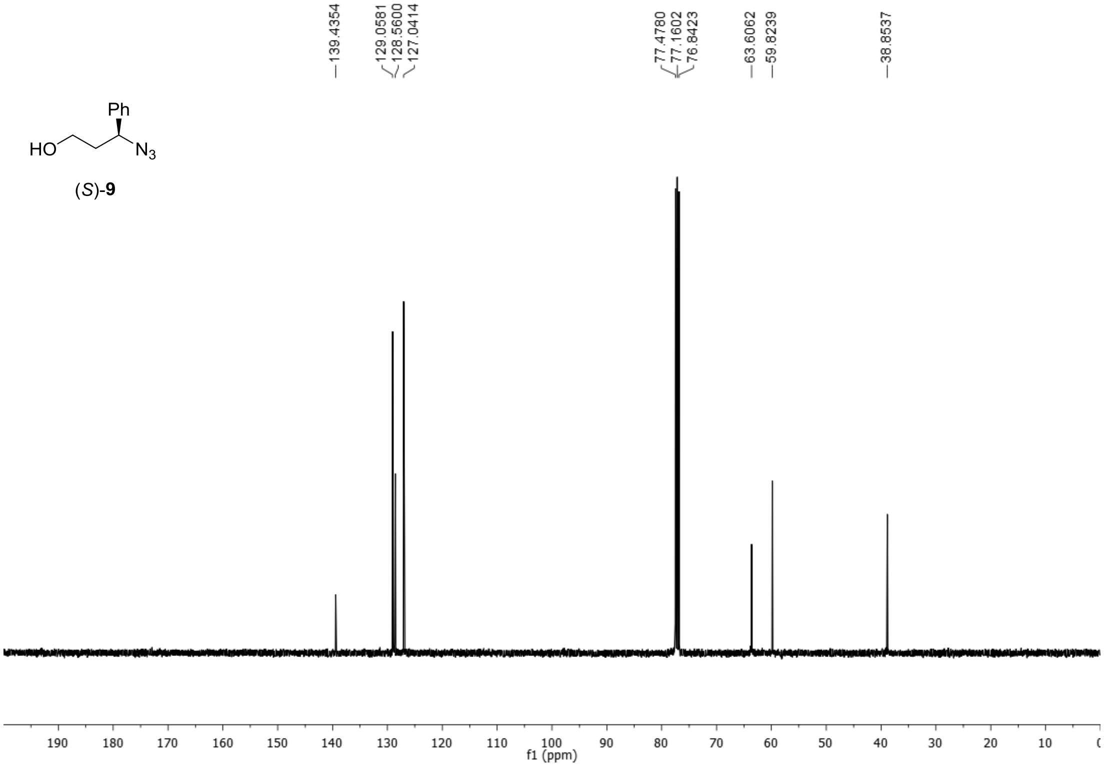

Supplementary Figure 44. ^{1}H NMR spectrum in $CDCl_3$, 400 MHz, of **S4**.

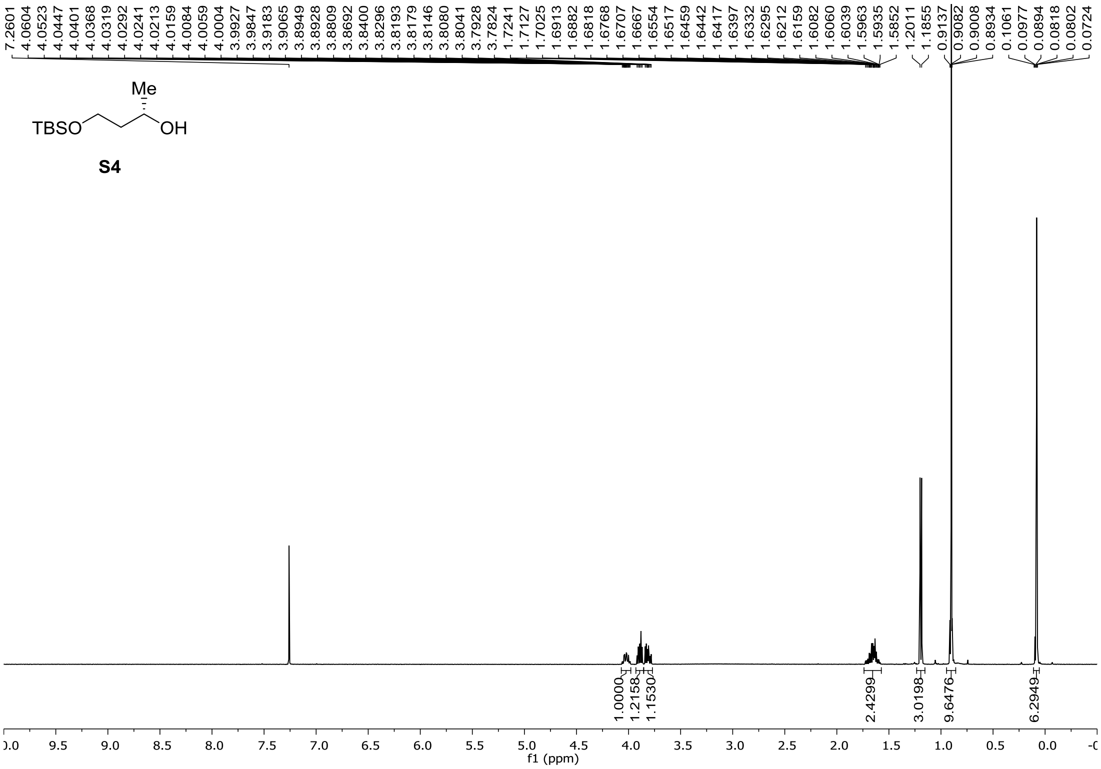

Supplementary Figure 45. ^{13}C NMR spectrum in $CDCl_3$, 101 MHz, of **S4**.

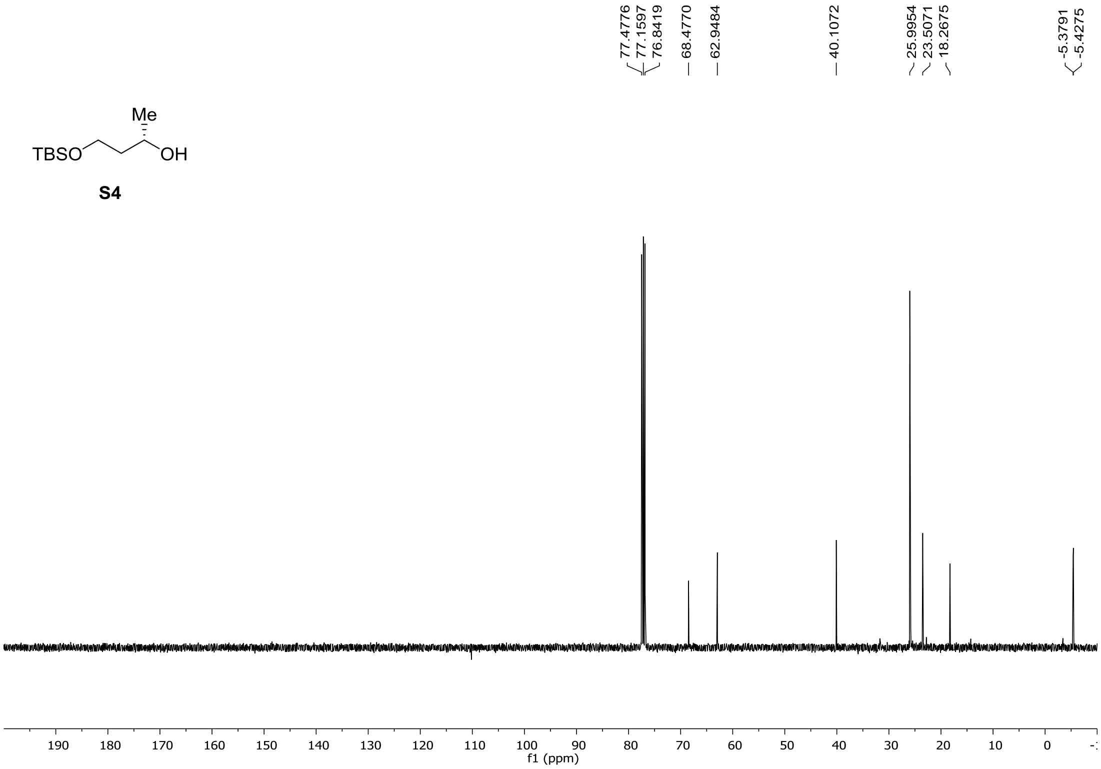

Supplementary Figure 46. ^{1}H NMR spectrum in $CDCl_3$, 400 MHz, of **S5**.

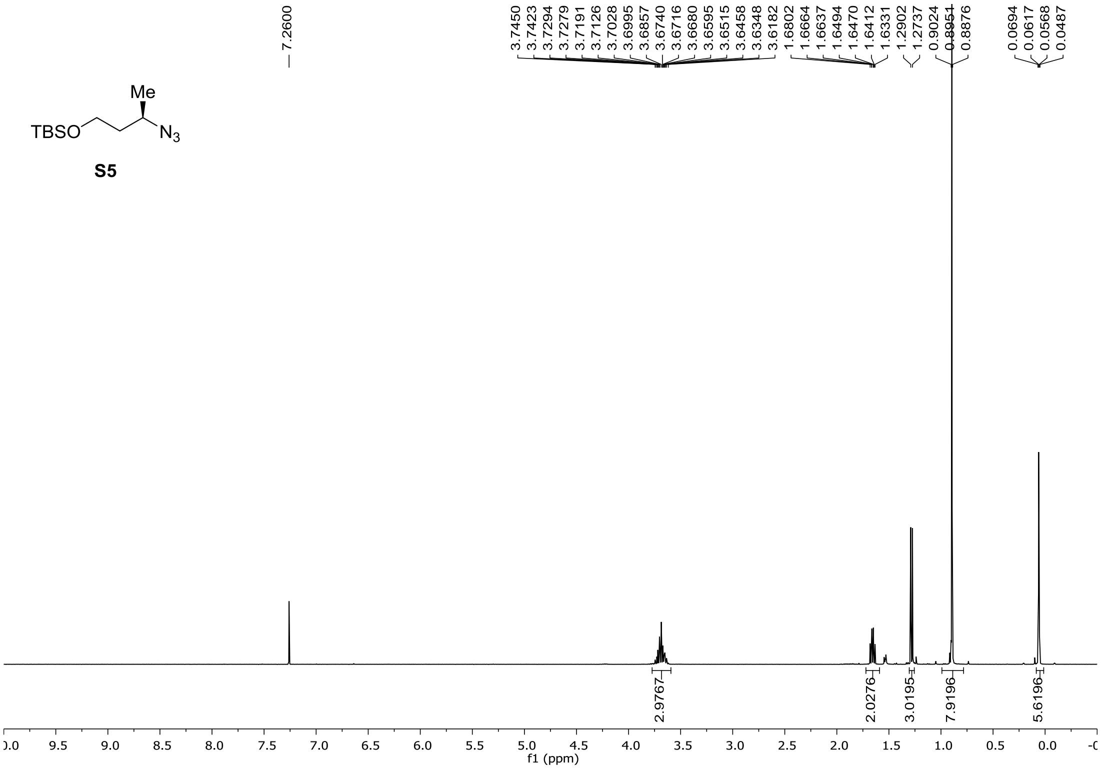

Supplementary Figure 47. ^{13}C NMR spectrum in $CDCl_3$, 101 MHz, of **S5**.

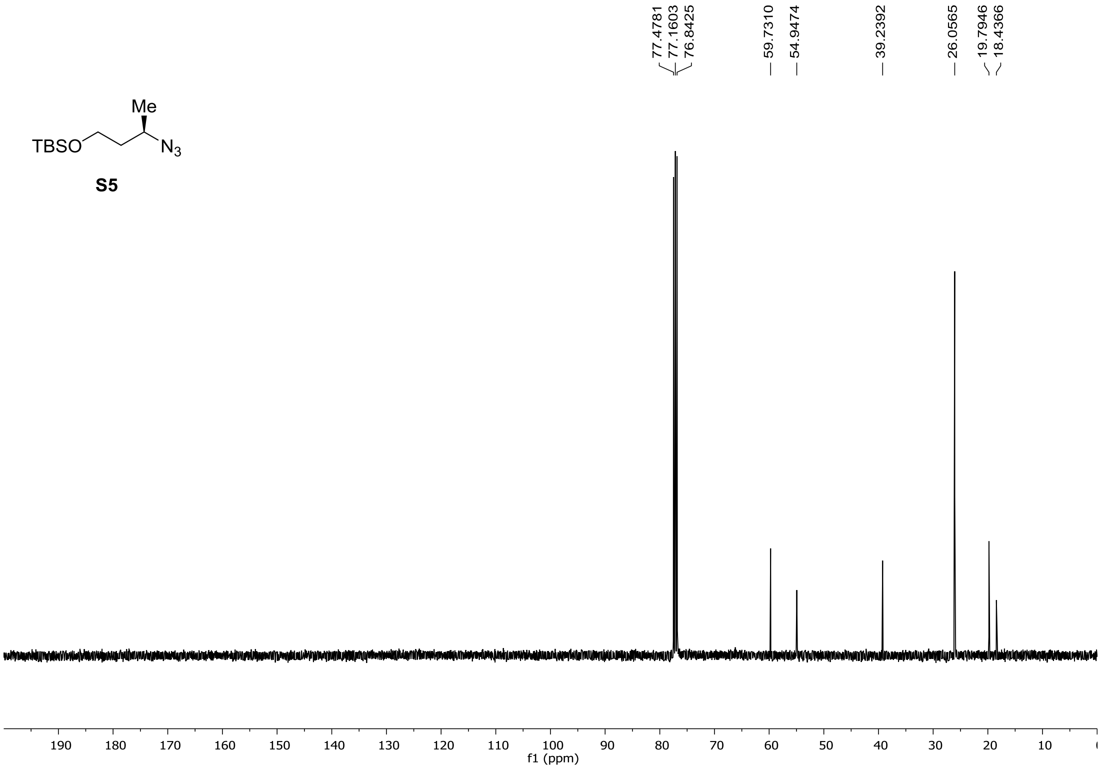

Supplementary Figure 48. ^{1}H NMR spectrum in $CDCl_3$, 400 MHz, of (*R*)-**10**.

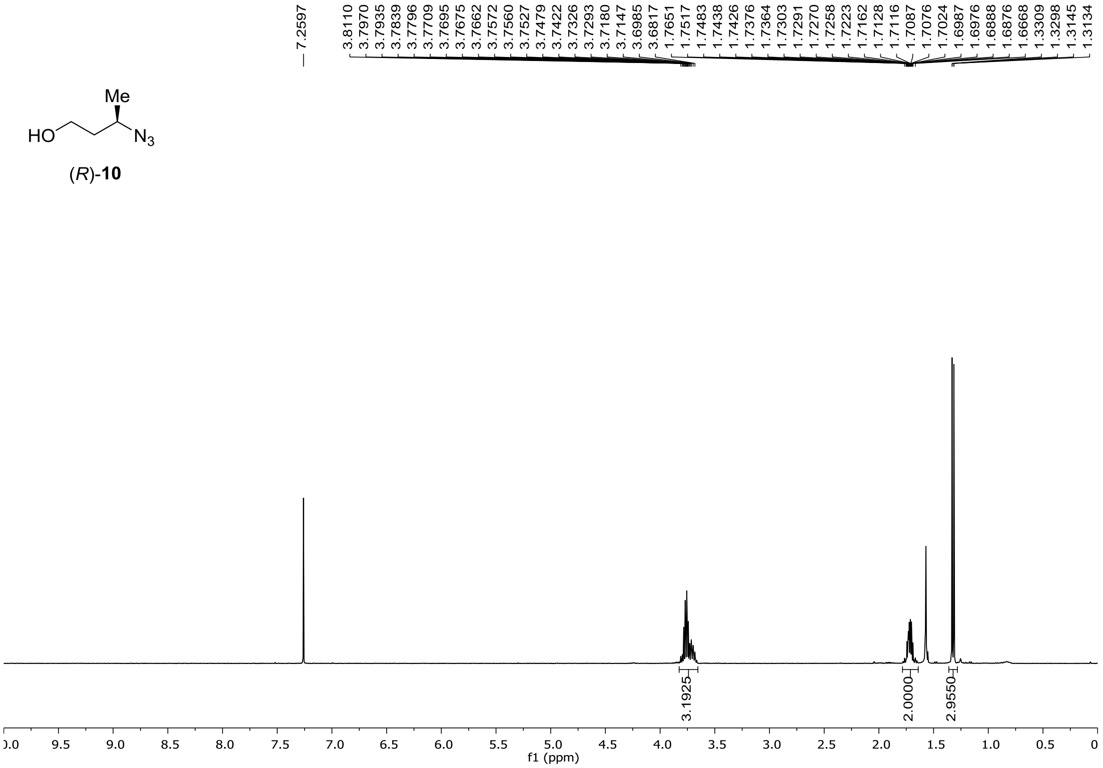

Supplementary Figure 49. ^{13}C NMR spectrum in $CDCl_3$, 101 MHz, of (*R*)-**10**.

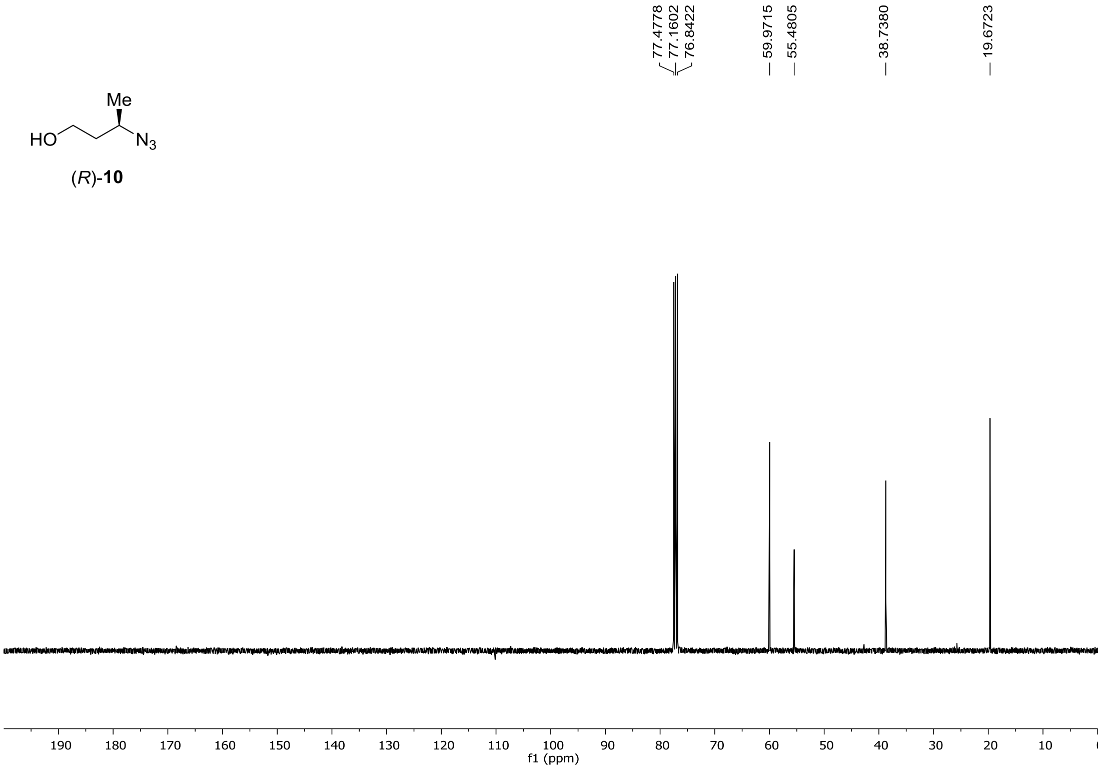

Supplementary Figure 50. ^{1}H NMR spectrum in $CDCl_3$, 400 MHz, of **S6**.

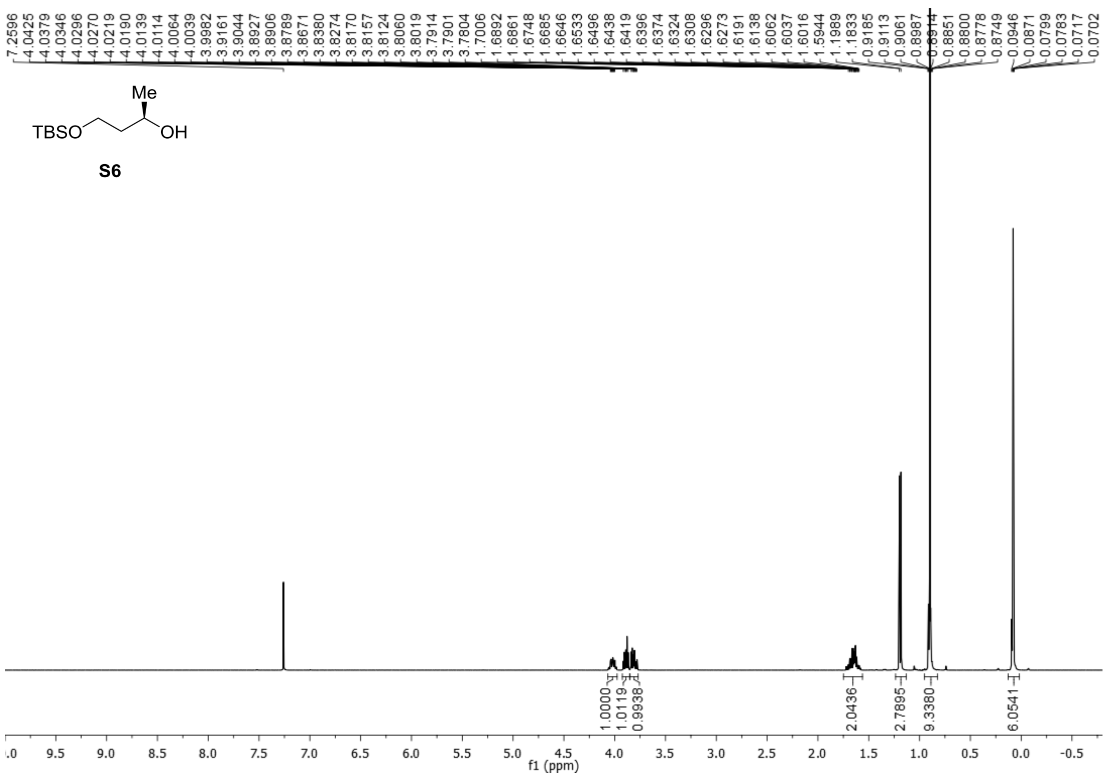

Supplementary Figure 51. ^{13}C NMR spectrum in $CDCl_3$, 101 MHz, of **S6**.

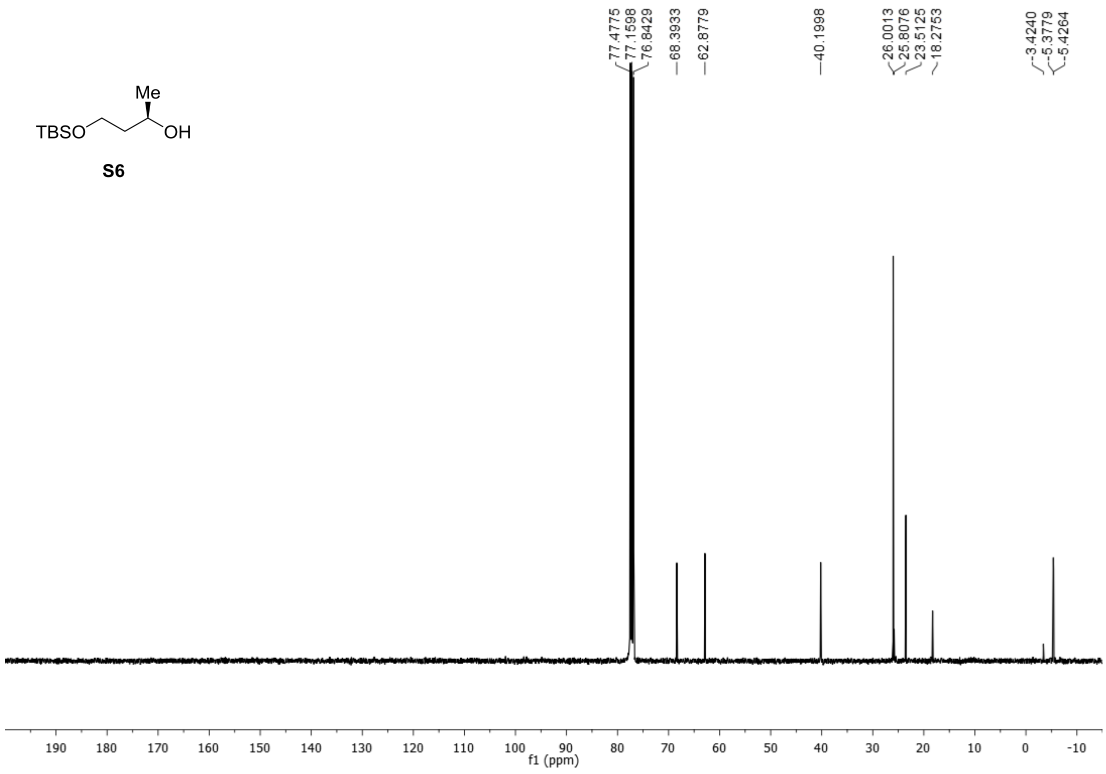

Supplementary Figure 52. ^{1}H NMR spectrum in $CDCl_3$, 400 MHz, of **S7**.

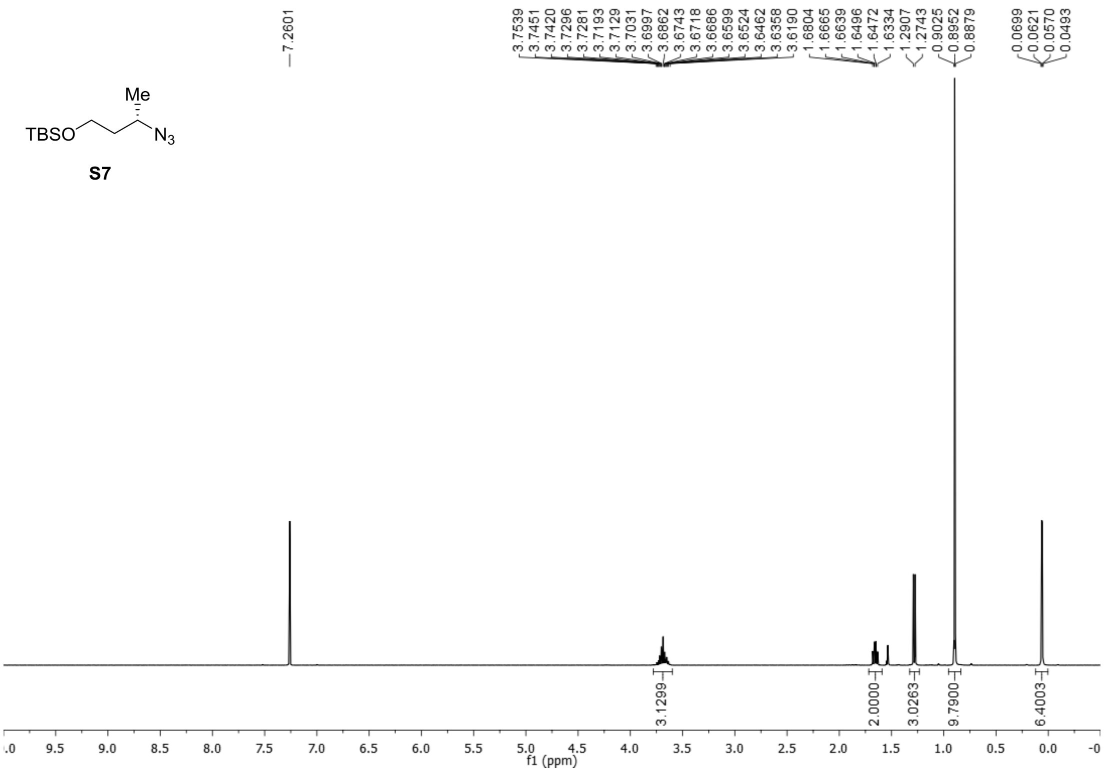

Supplementary Figure 53. ^{13}C NMR spectrum in $CDCl_3$, 101 MHz, of **S7**.

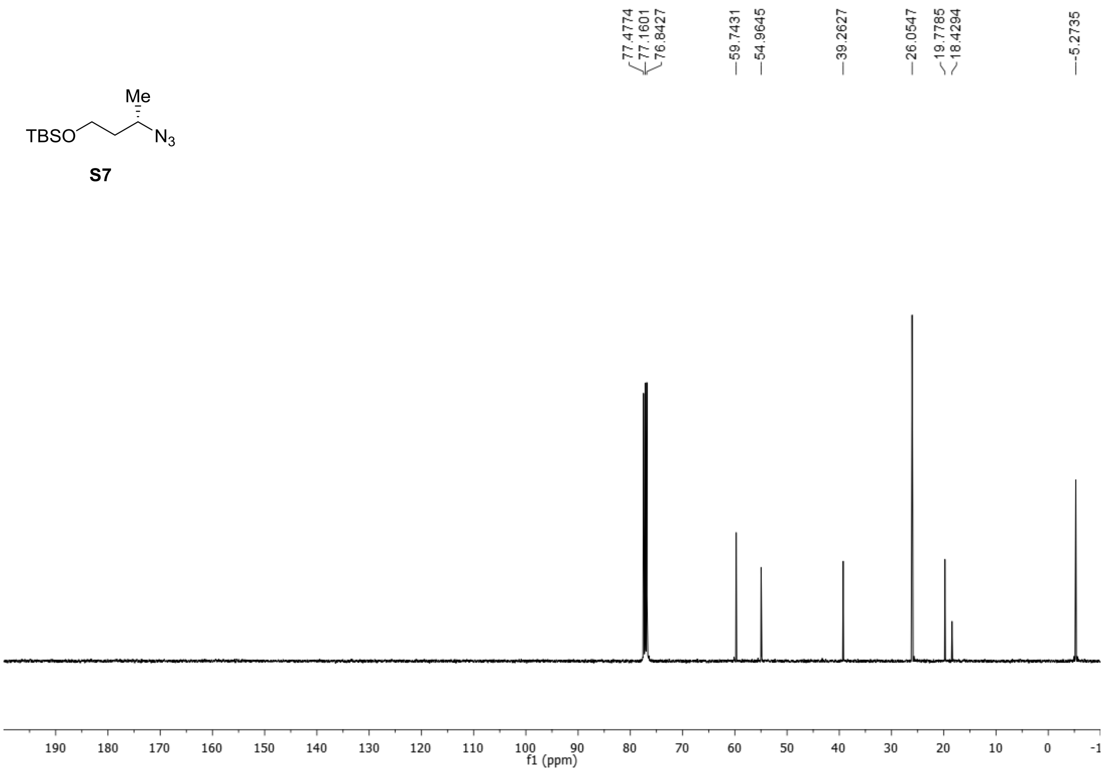

Supplementary Figure 54. ^{1}H NMR spectrum in $CDCl_3$, 400 MHz, of (*S*)-**10**.

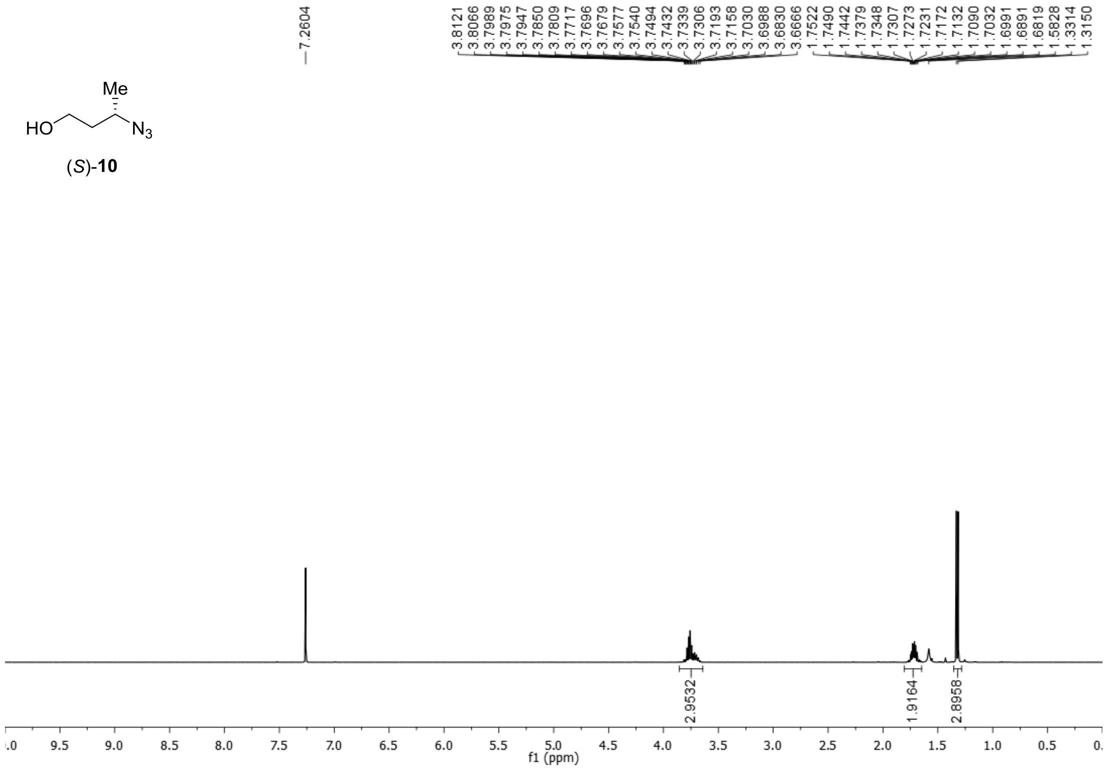

Supplementary Figure 55. ^{13}C NMR spectrum in $CDCl_3$, 101 MHz, of (*S*)-**10**.

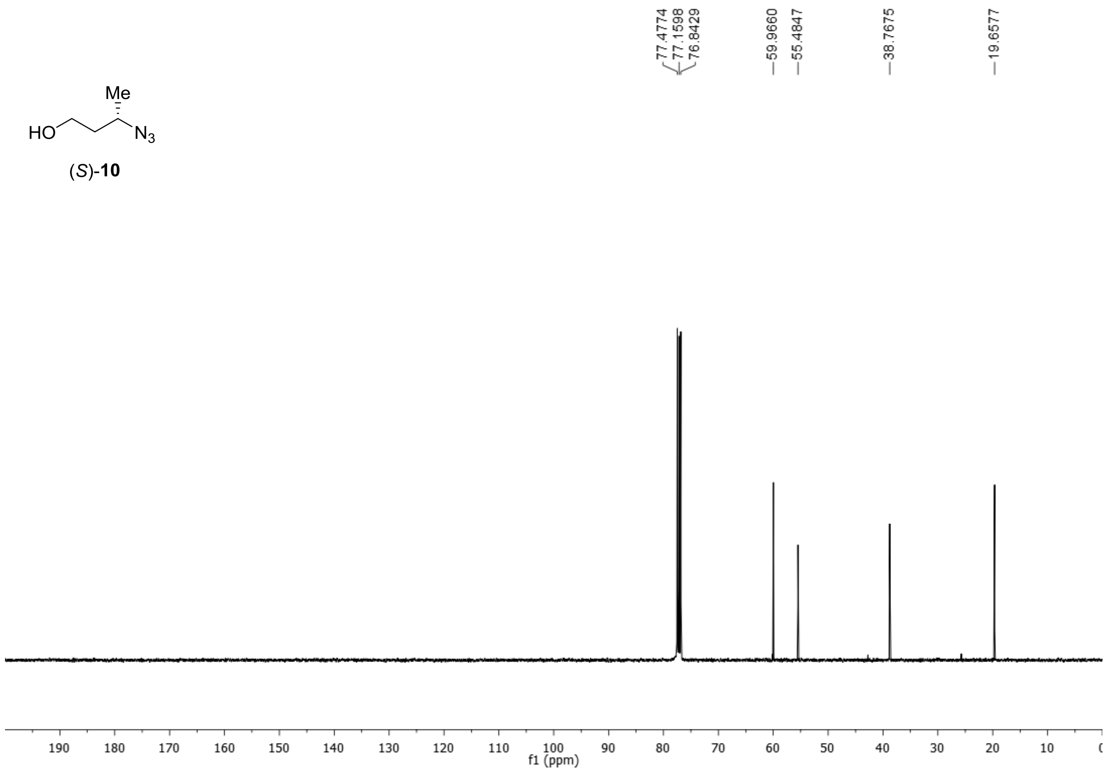

Supplementary Figure 56. ^{1}H NMR spectrum in $CDCl_3$, 400 MHz, of **S8**.

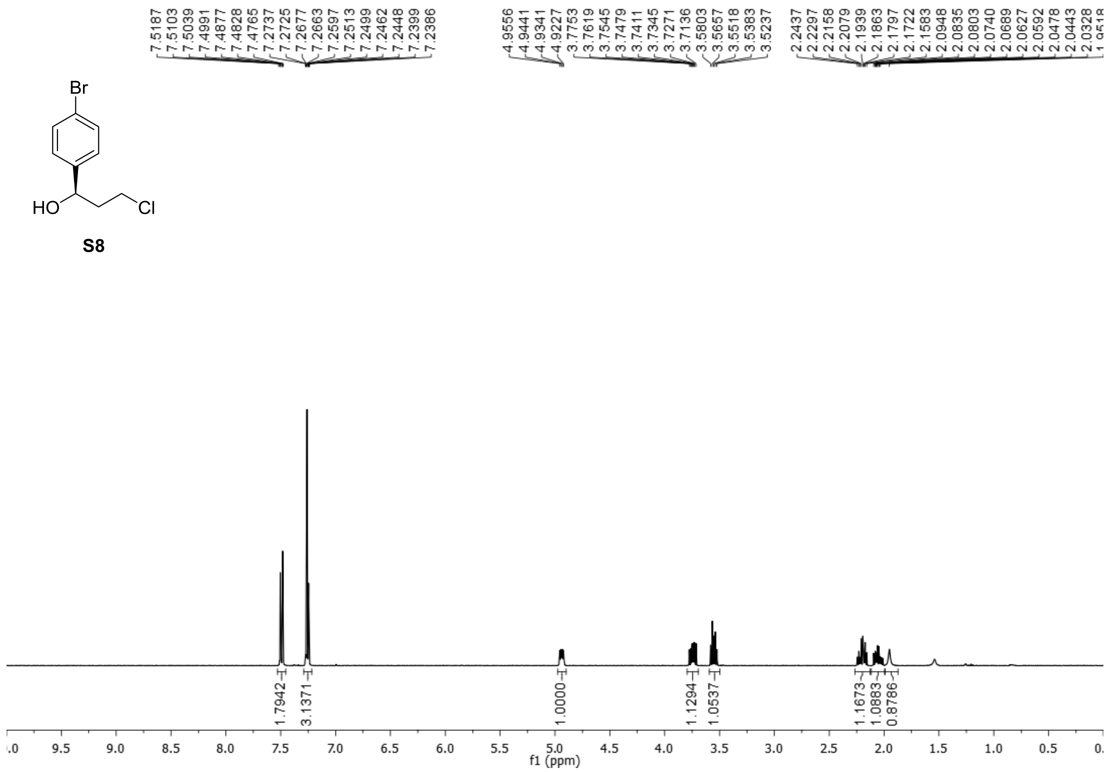

Supplementary Figure 57. ^{13}C NMR spectrum in $CDCl_3$, 101 MHz, of **S8**.

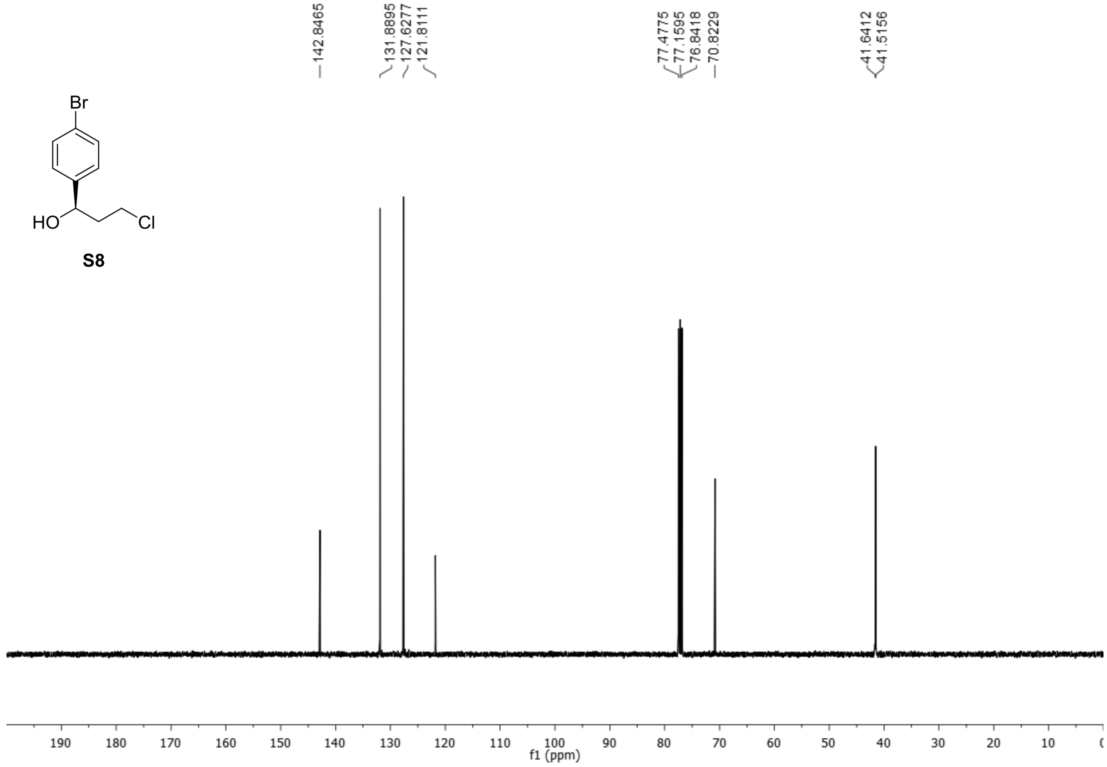

Supplementary Figure 58. ^{1}H NMR spectrum in $CDCl_3$, 400 MHz, of (*R*)-**11**.

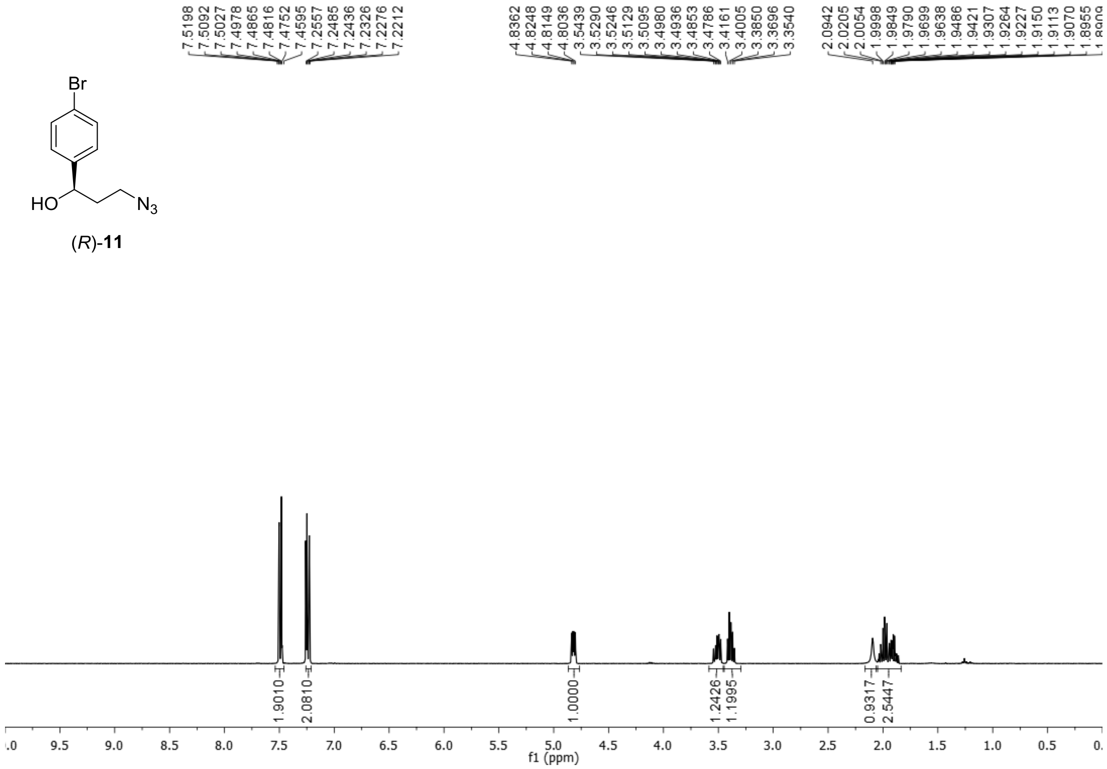

Supplementary Figure 59. ^{13}C NMR spectrum in $CDCl_3$, 101 MHz, of (*R*)-**11**.

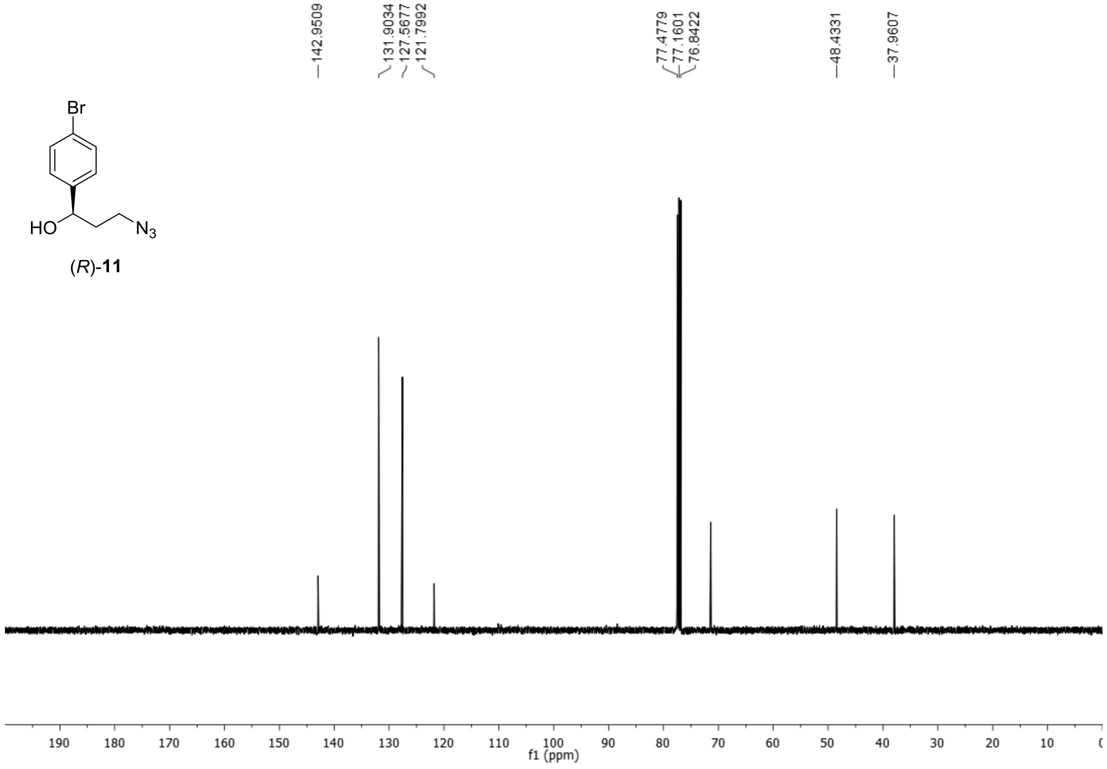

Supplementary Figure 60. ^{1}H NMR spectrum in $CDCl_3$, 400 MHz, of **S9**.

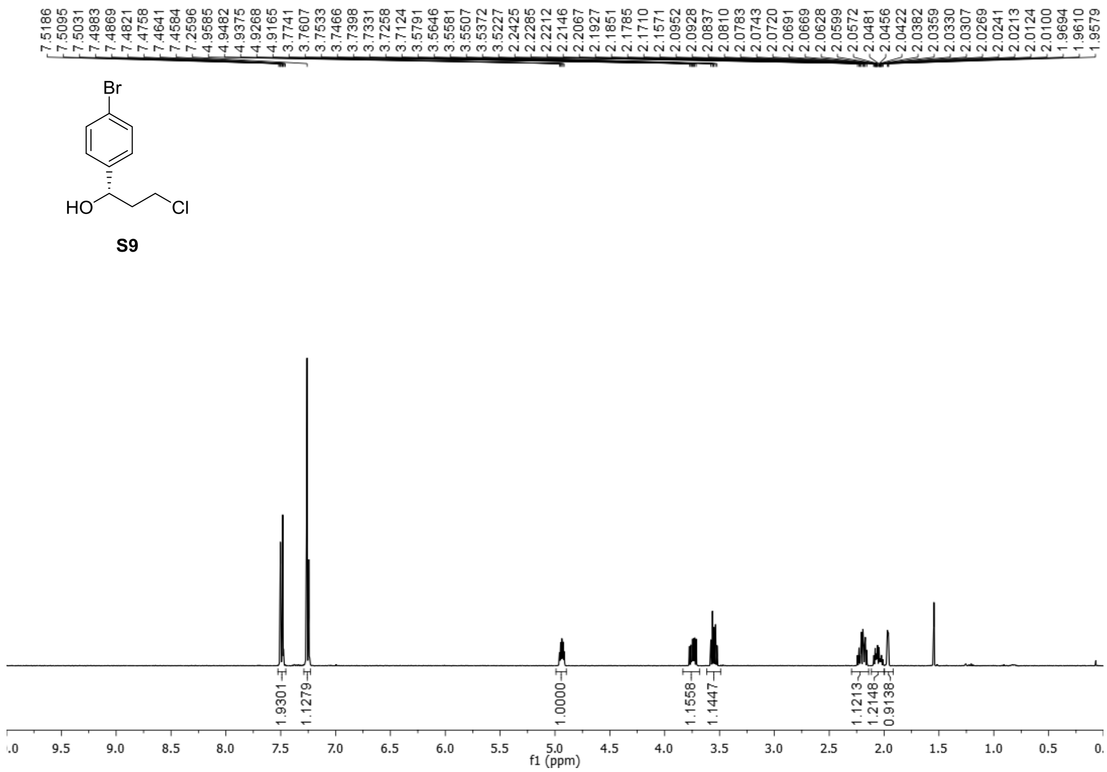

Supplementary Figure 61. ^{13}C NMR spectrum in $CDCl_3$, 101 MHz, of **S9**.

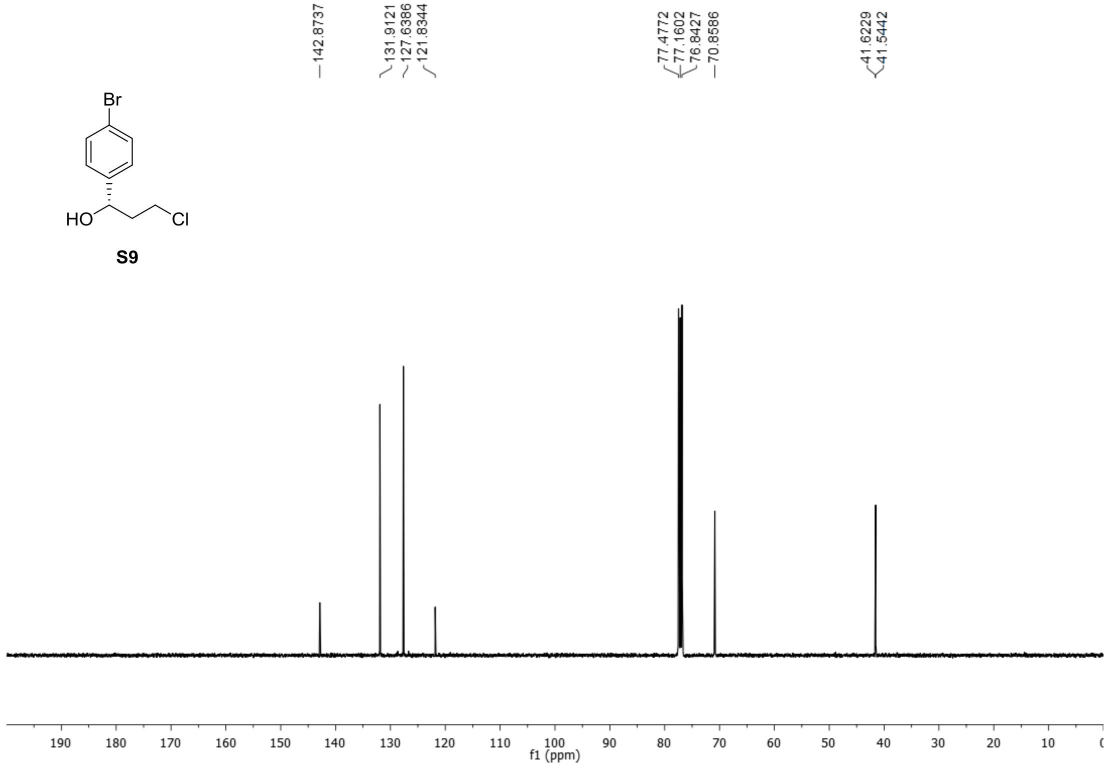

Supplementary Figure 62. ^{1}H NMR spectrum in $CDCl_3$, 400 MHz, of (*S*)-**11**.

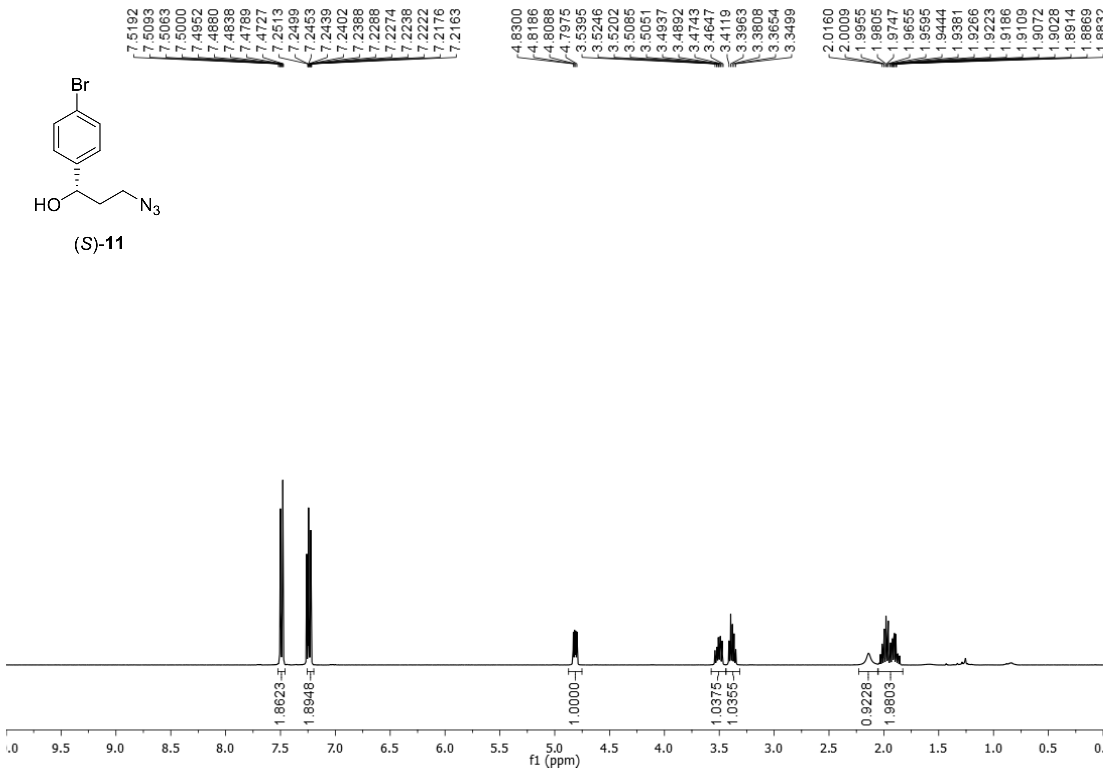

Supplementary Figure 63. ^{13}C NMR spectrum in $CDCl_3$, 101 MHz, of (*S*)-**11**.

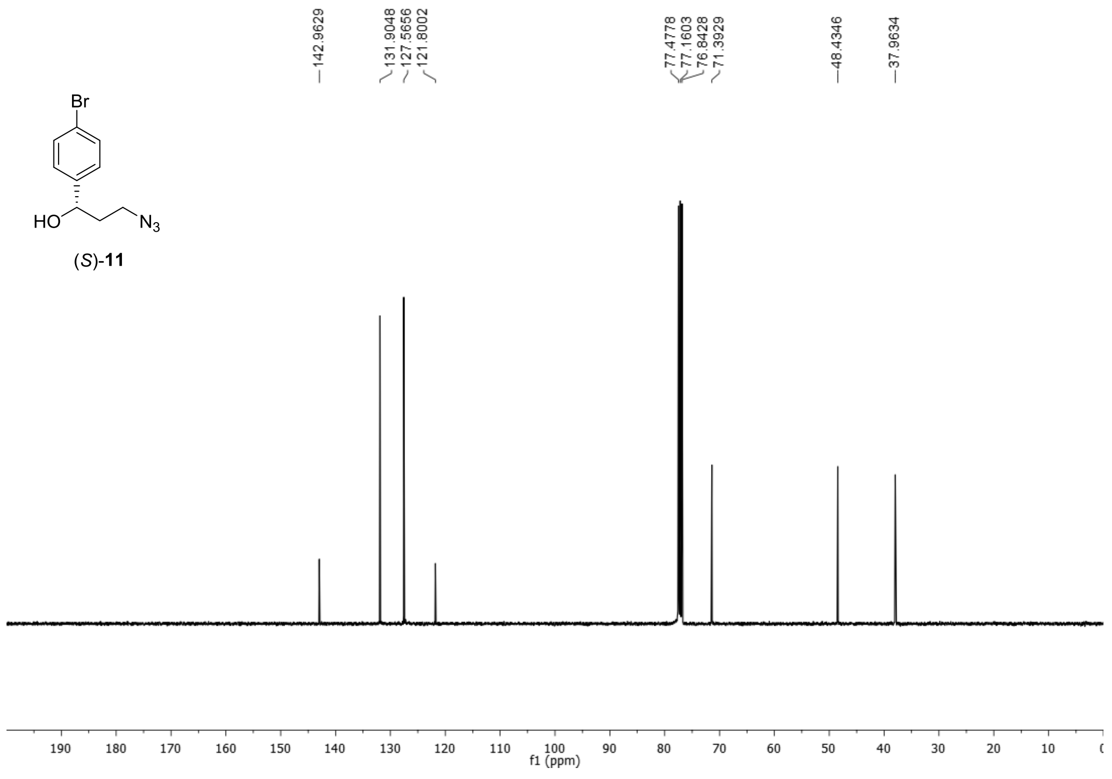

Supplementary Figure 64. ^{1}H NMR spectrum in $CDCl_3$, 400 MHz, of **12**.

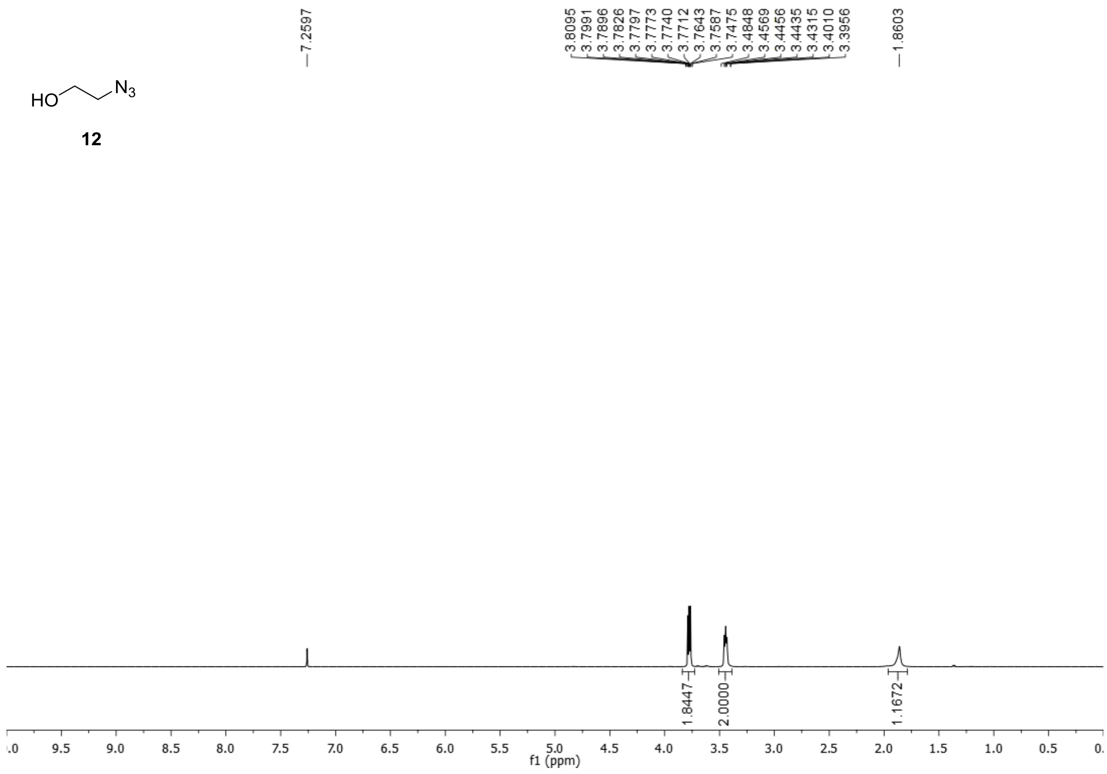

Supplementary Figure 65. ^{13}C NMR spectrum in $CDCl_3$, 101 MHz, of **12**.

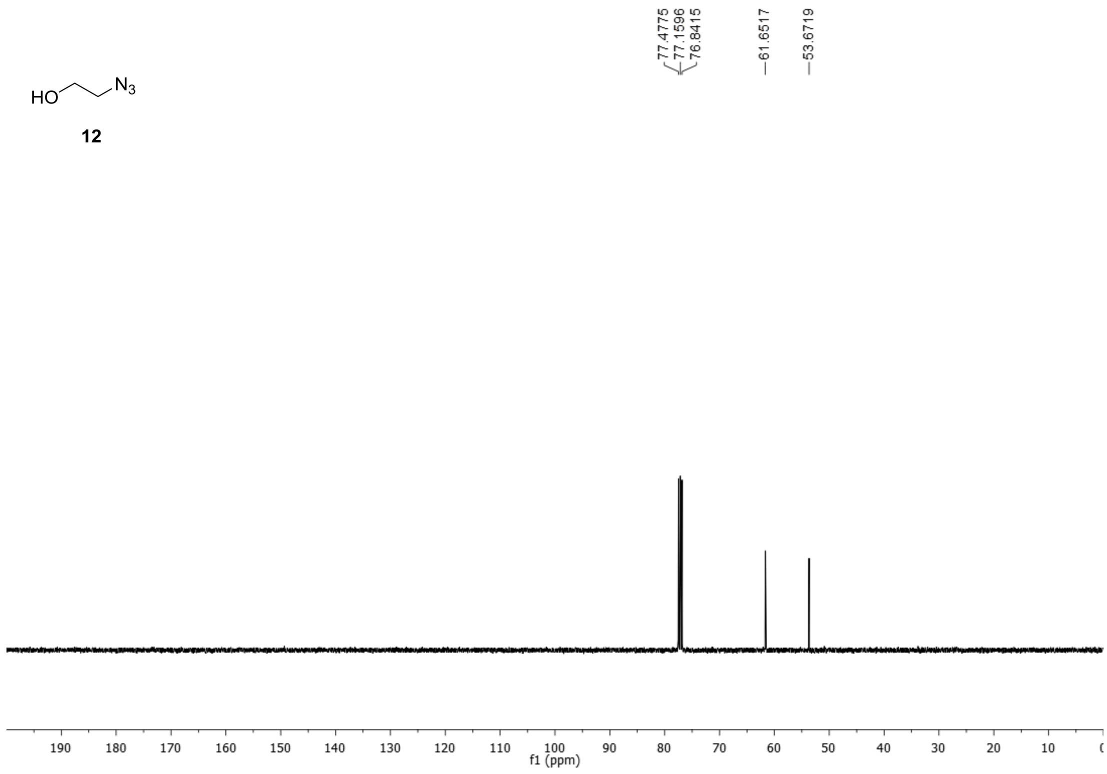

Supplementary Figure 66. ^{1}H NMR spectrum in $CDCl_3$, 400 MHz, of **S10**.

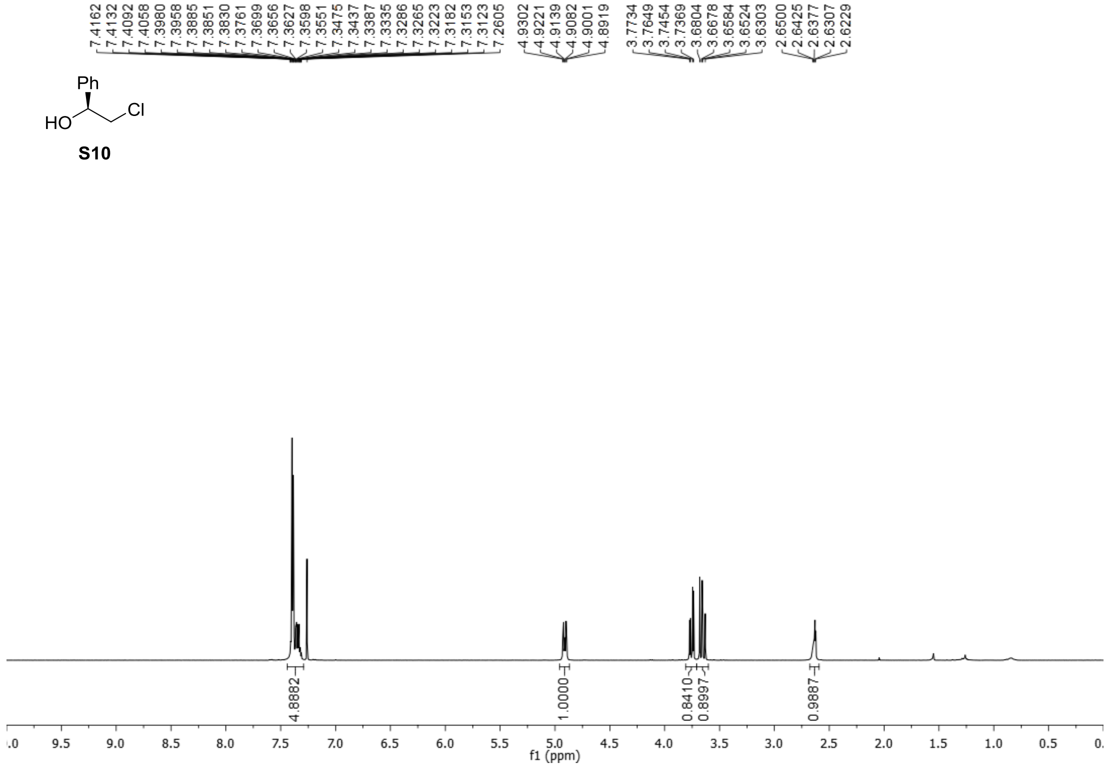

Supplementary Figure 67. ^{13}C NMR spectrum in $CDCl_3$, 101 MHz, of **S10**.

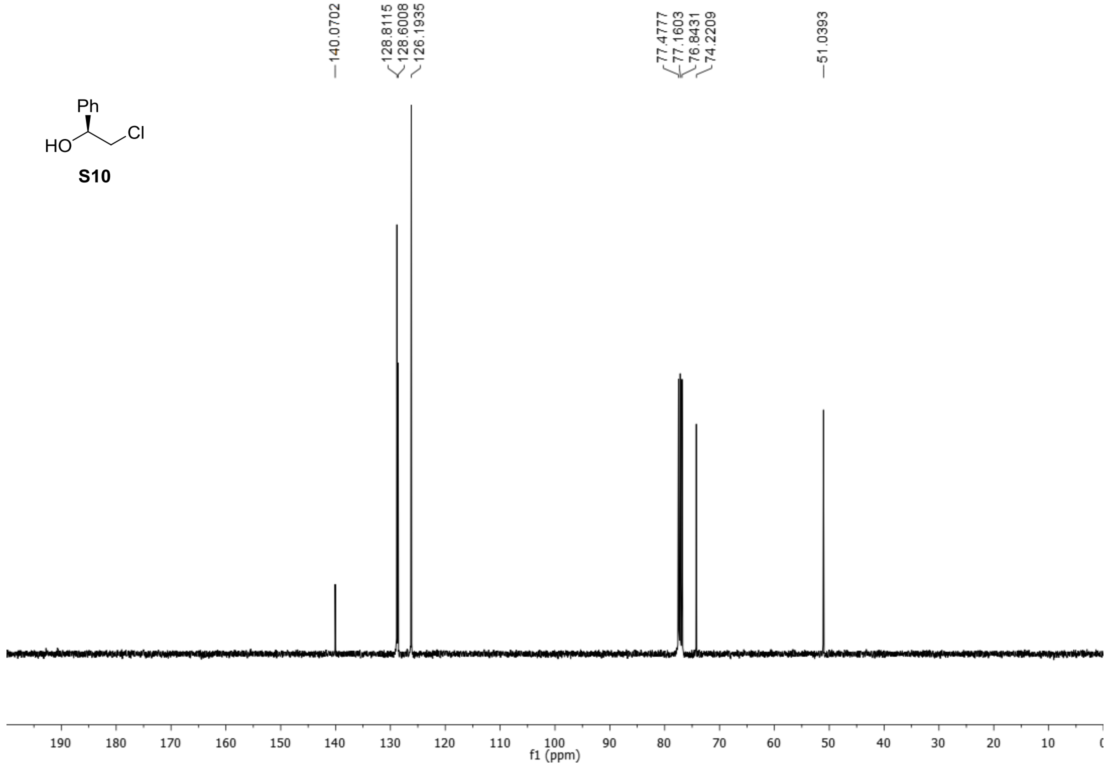

Supplementary Figure 68. ^{1}H NMR spectrum in $CDCl_3$, 400 MHz, of (*S*)-**13**.

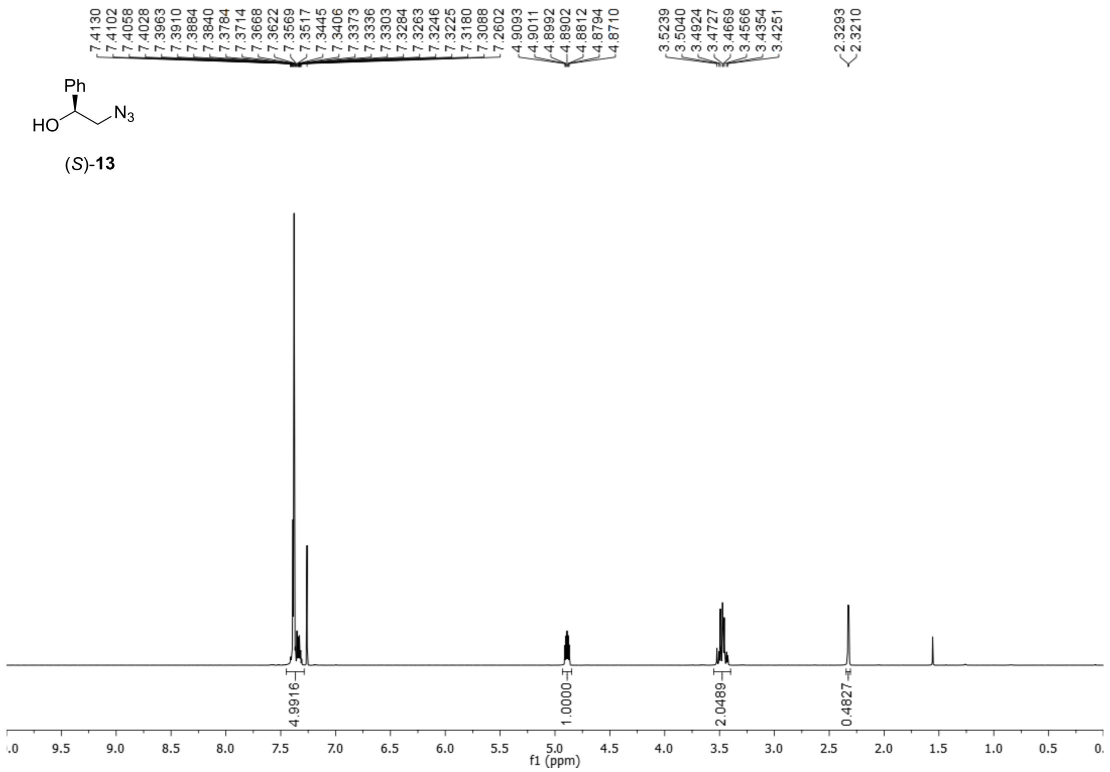

Supplementary Figure 69. ^{13}C NMR spectrum in $CDCl_3$, 101 MHz, of (*S*)-**13**.

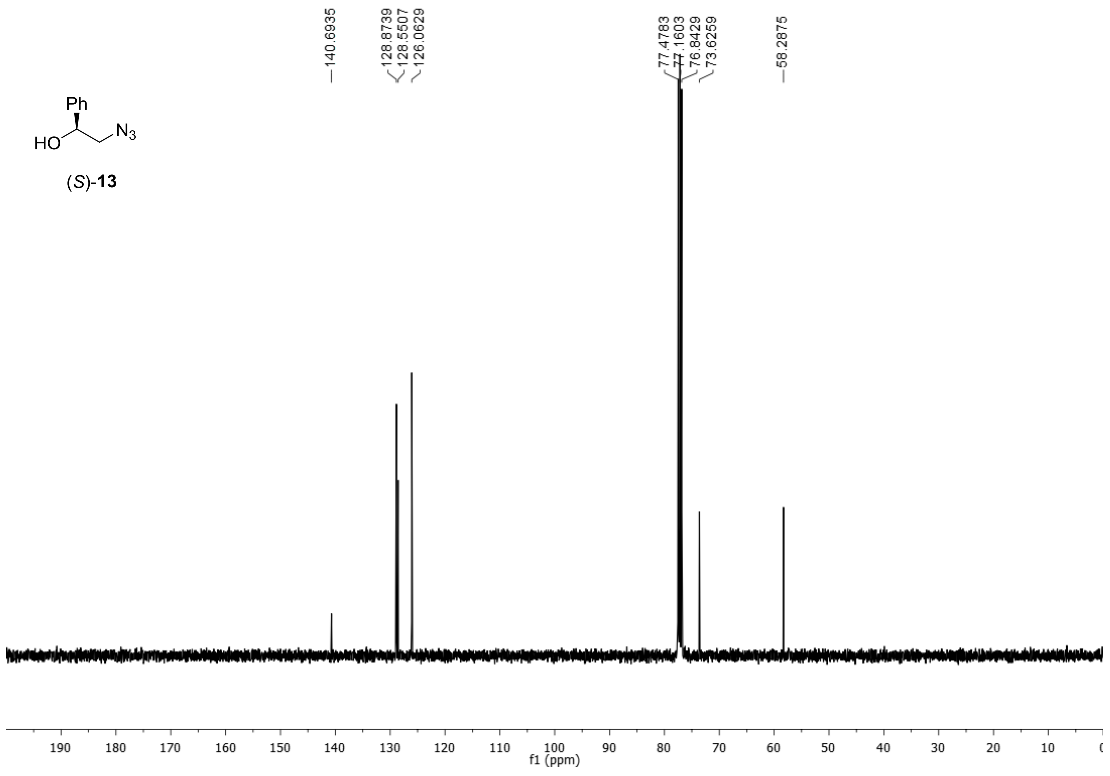

Supplementary Figure 70. ^{1}H NMR spectrum in $CDCl_3$, 400 MHz, of **S11**.

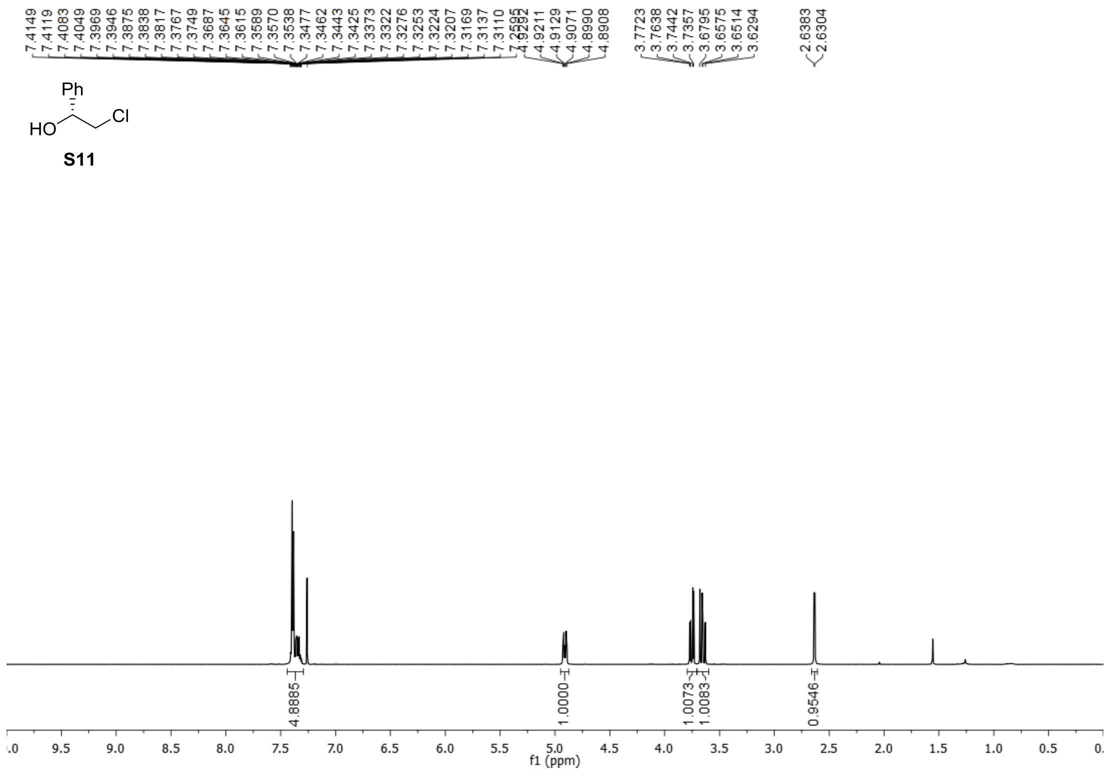

Supplementary Figure 71. ^{13}C NMR spectrum in $CDCl_3$, 101 MHz, of **S11**.

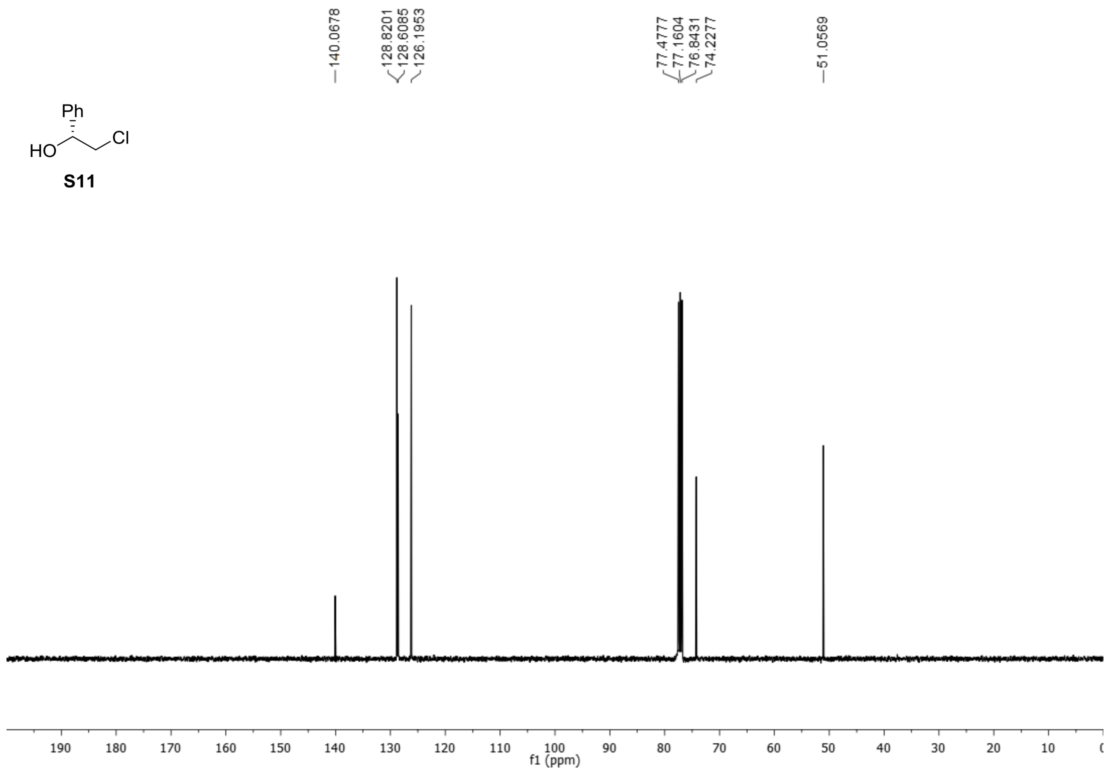

Supplementary Figure 72. ^{1}H NMR spectrum in $CDCl_3$, 400 MHz, of (*R*)-**13**.

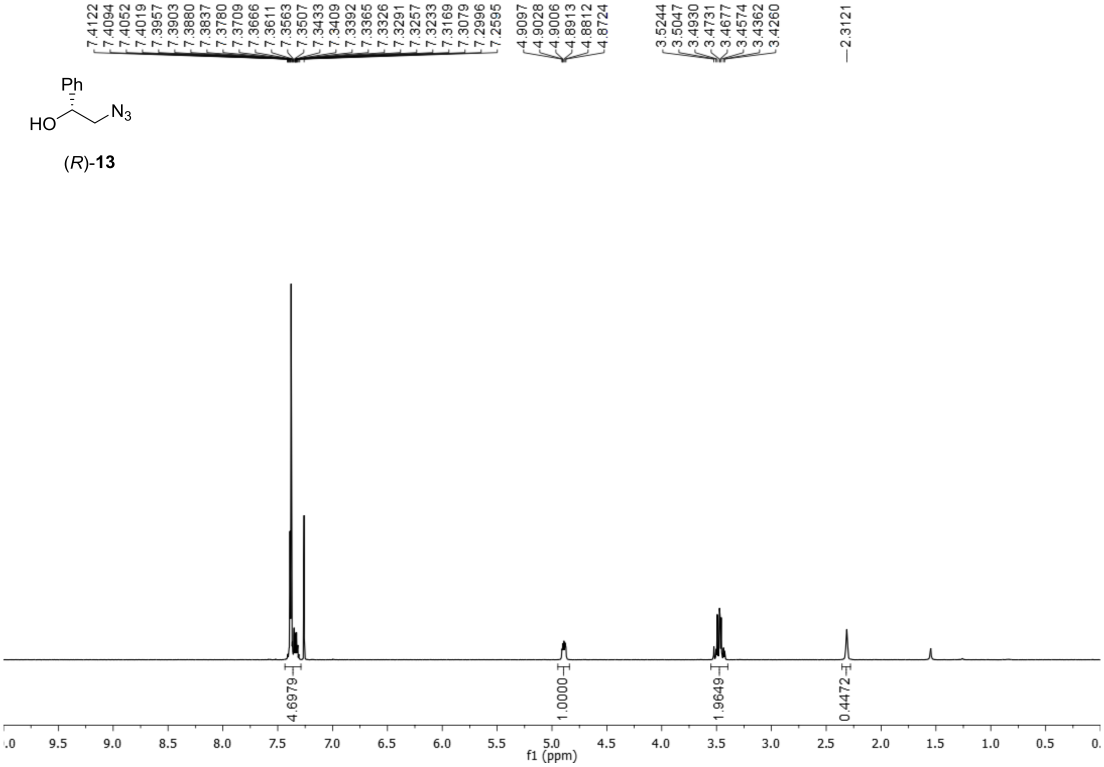

Supplementary Figure 73. ^{13}C NMR spectrum in $CDCl_3$, 101 MHz, of (*R*)-**13**.

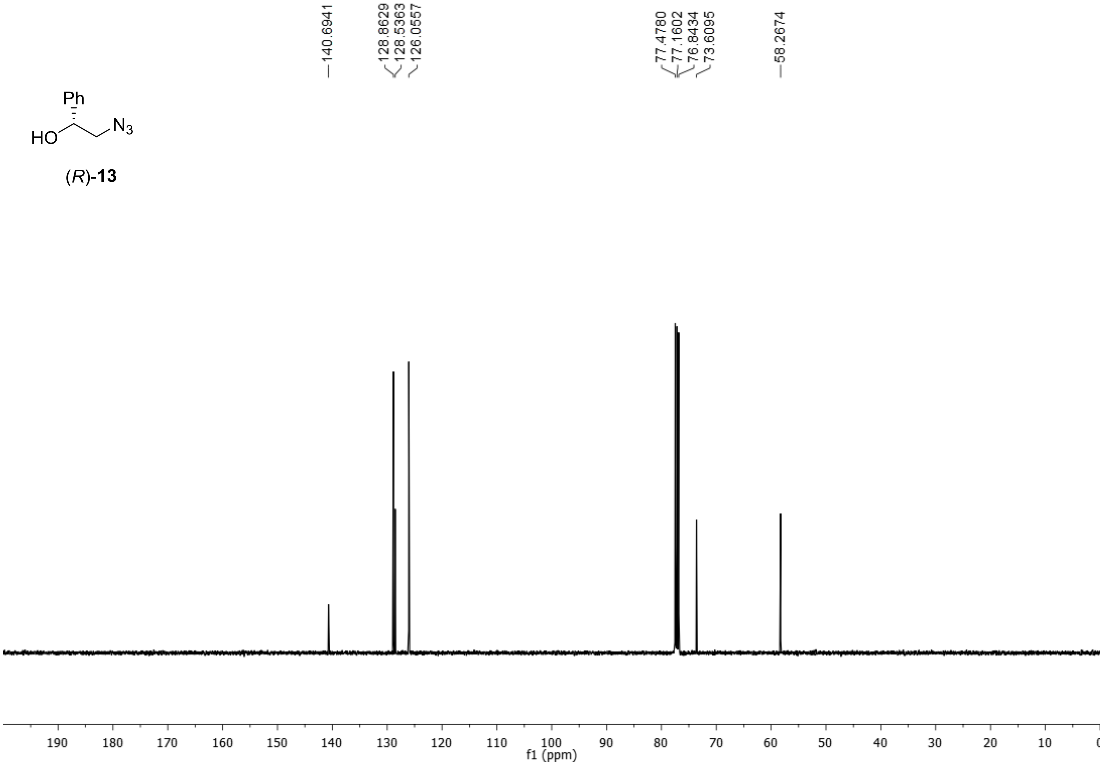

Supplementary Figure 74. ^{1}H NMR spectrum in $CDCl_3$, 400 MHz, of (*S*)-**14**.

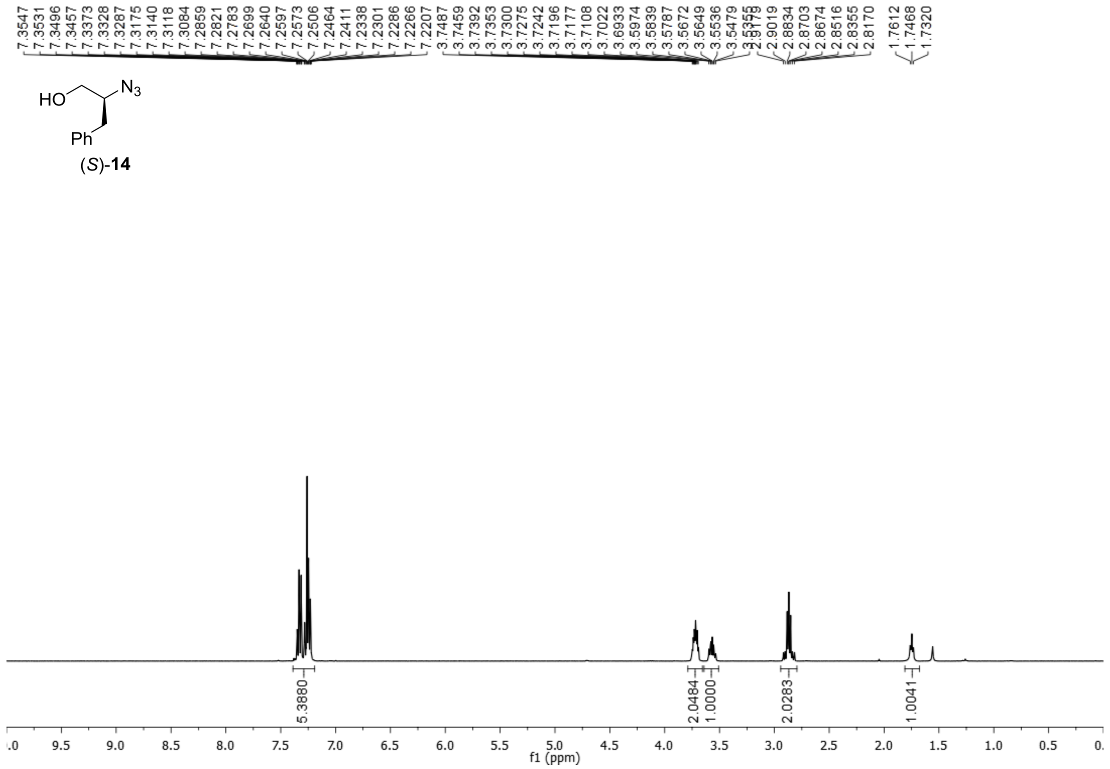

Supplementary Figure 75. ^{13}C NMR spectrum in $CDCl_3$, 101 MHz, of (*S*)-**14**.

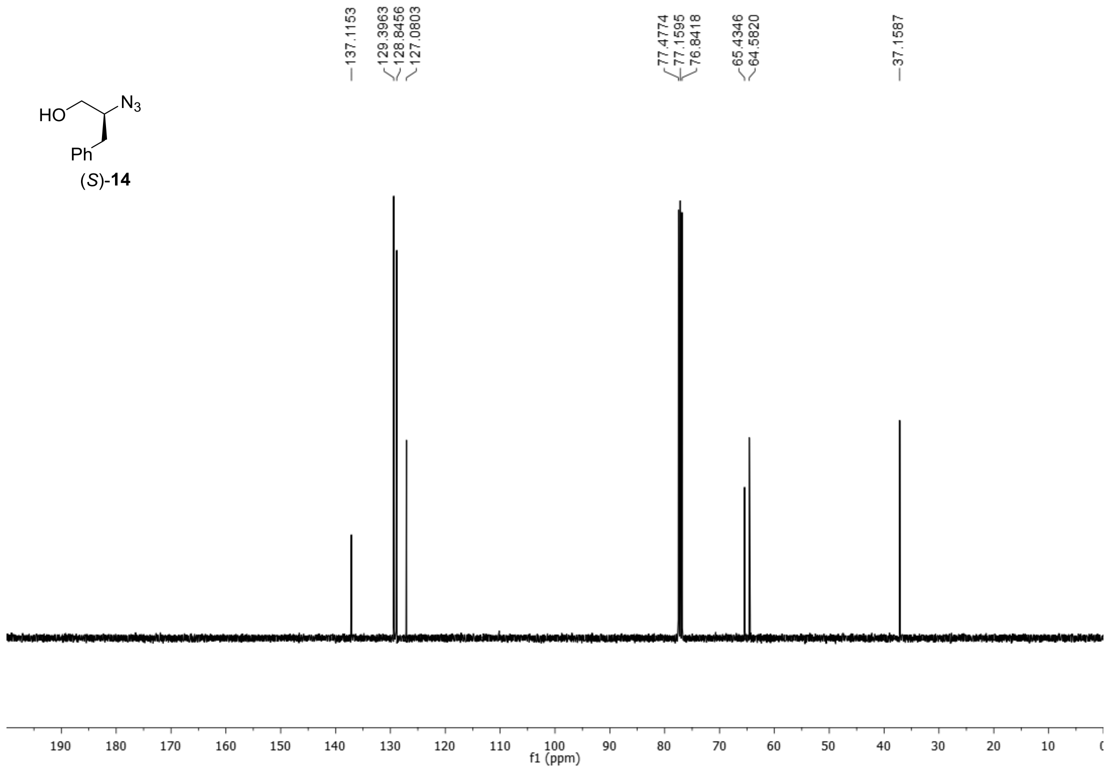

Supplementary Figure 76. ^{1}H NMR spectrum in $CDCl_3$, 400 MHz, of (*R*)-**14**.

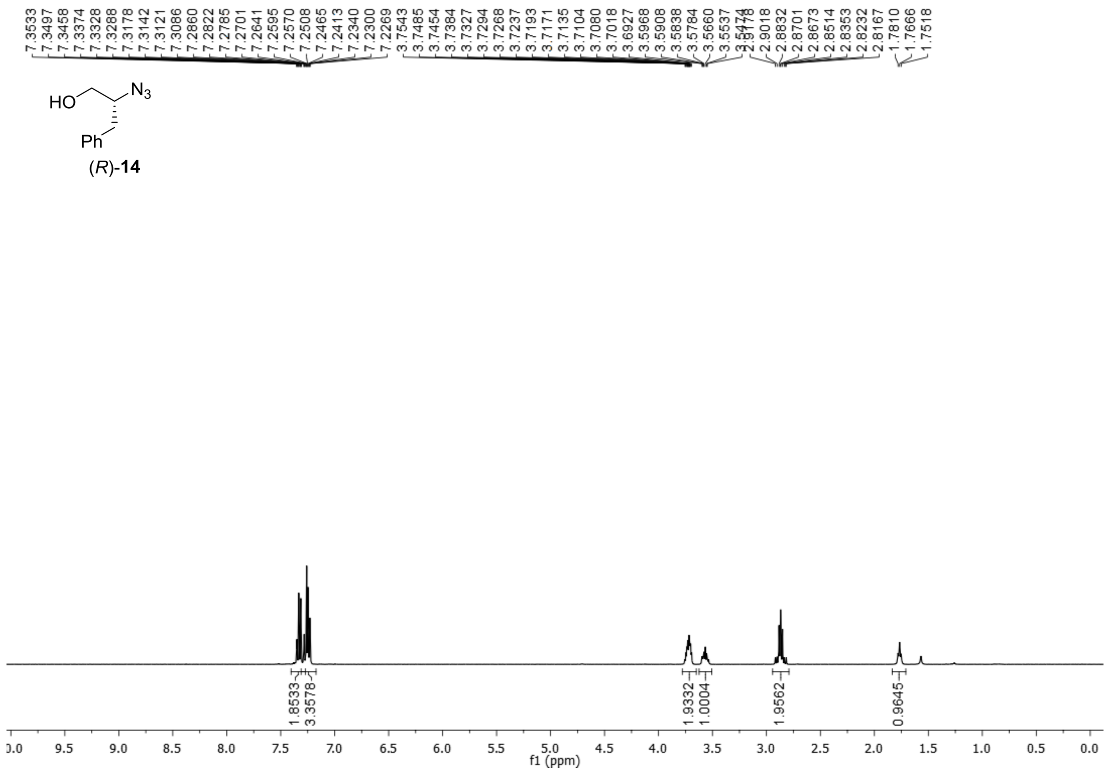

Supplementary Figure 77. ^{13}C NMR spectrum in $CDCl_3$, 101 MHz, of (*R*)-**14**.

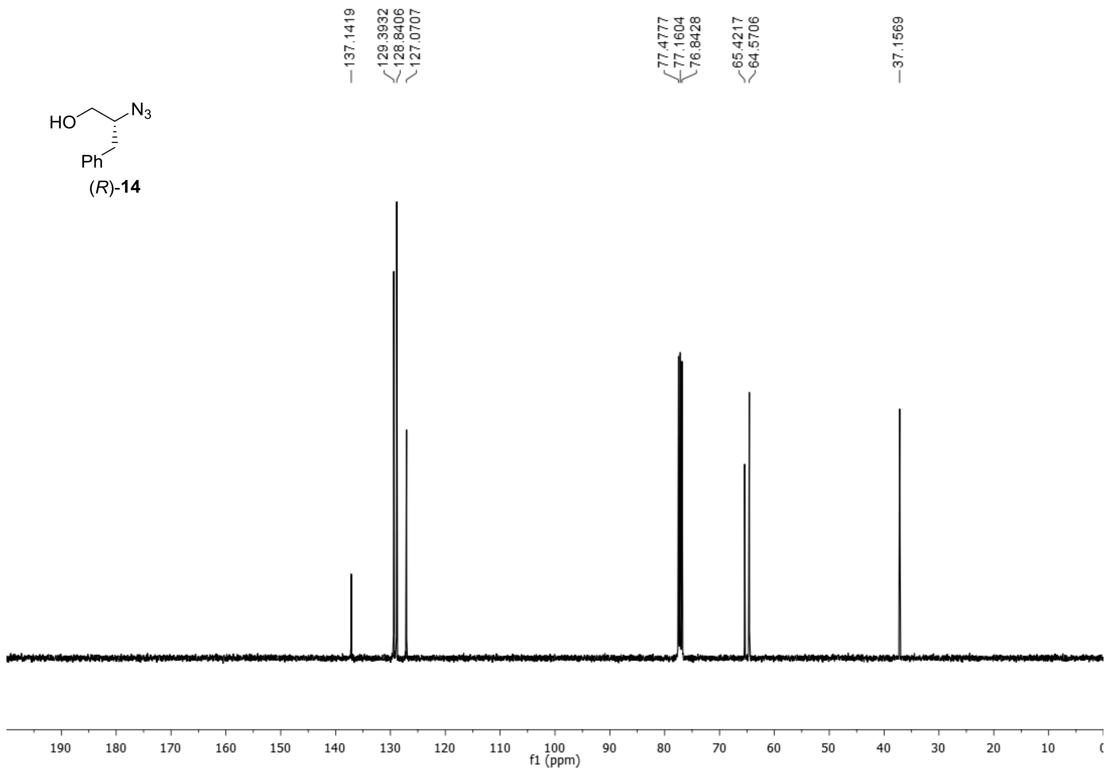

Supplementary Figure 78. ^{1}H NMR spectrum in $CDCl_3$, 400 MHz, of (*S*)-**15**.

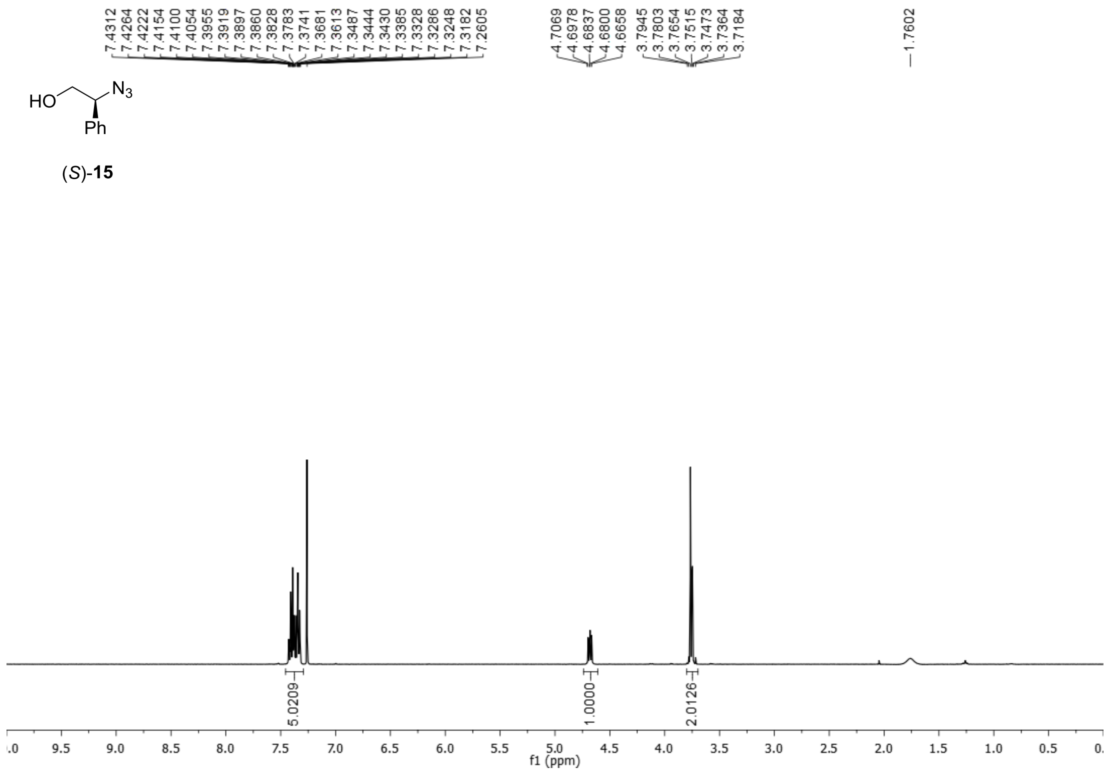

Supplementary Figure 79. ^{13}C NMR spectrum in $CDCl_3$, 101 MHz, of (*S*)-**15**.

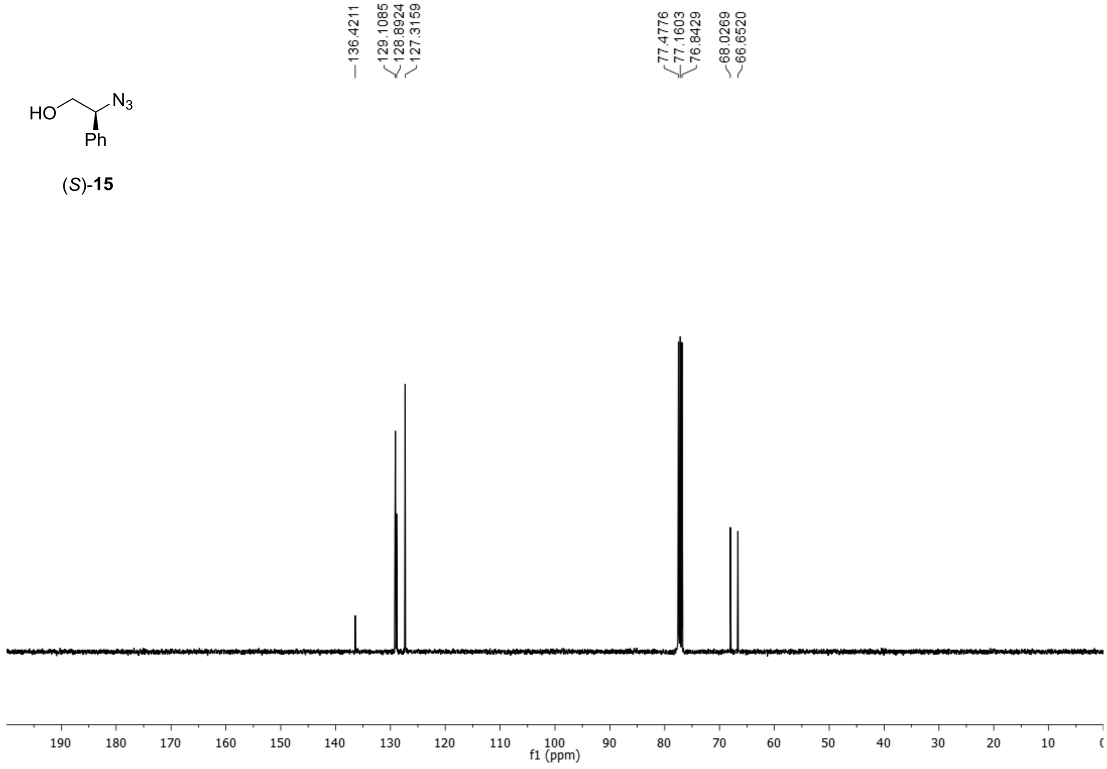

Supplementary Figure 80. ^{1}H NMR spectrum in $CDCl_3$, 400 MHz, of (*R*)-**15**.

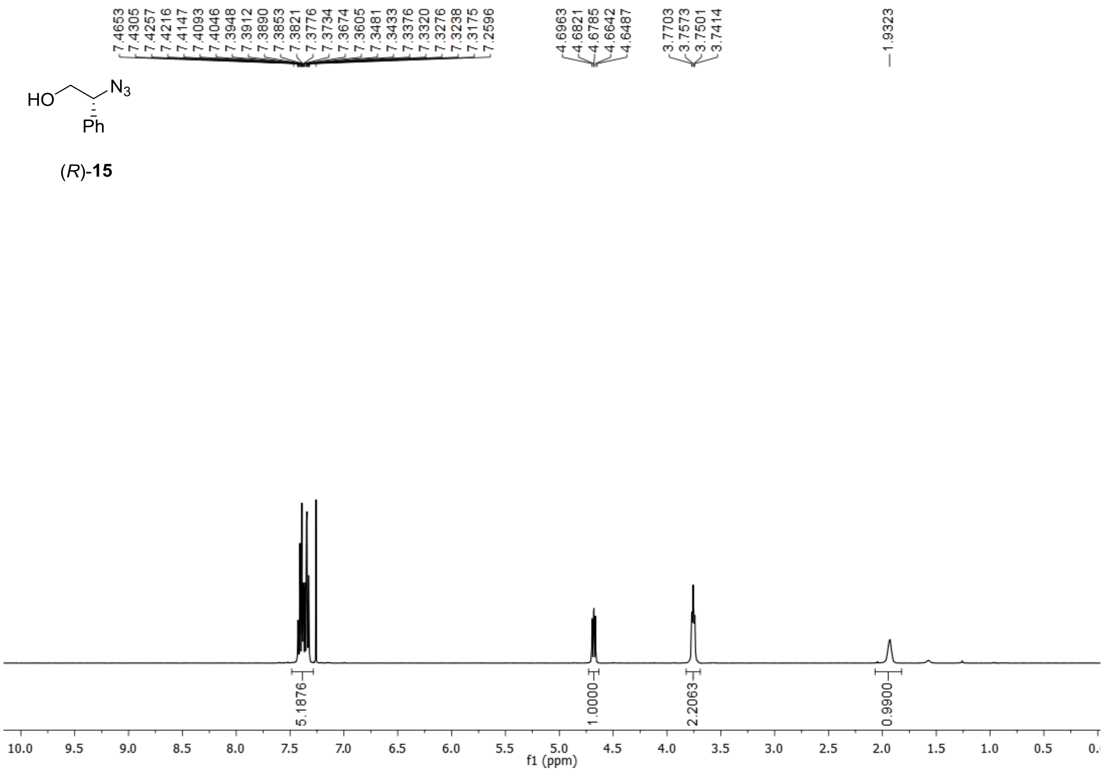

Supplementary Figure 81. ^{13}C NMR spectrum in $CDCl_3$, 101 MHz, of (*R*)-**15**.

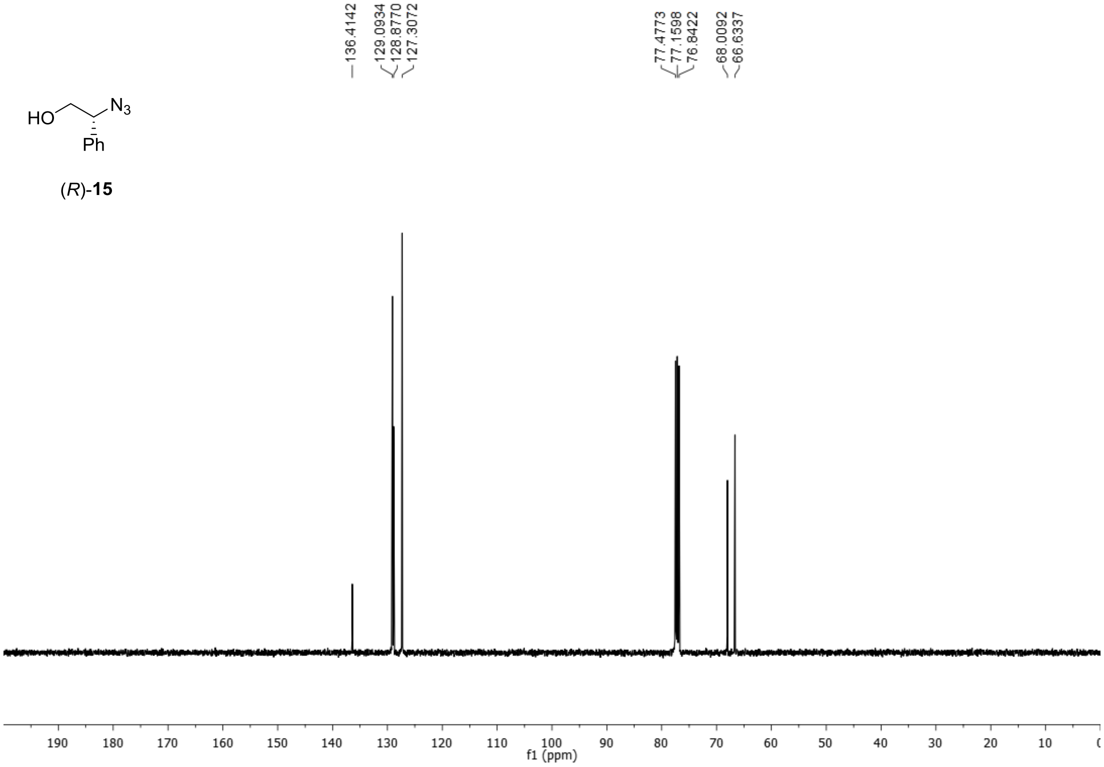

Supplementary Figure 82. ^{1}H NMR spectrum in $CDCl_3$, 400 MHz, of (*S*)-**16**.

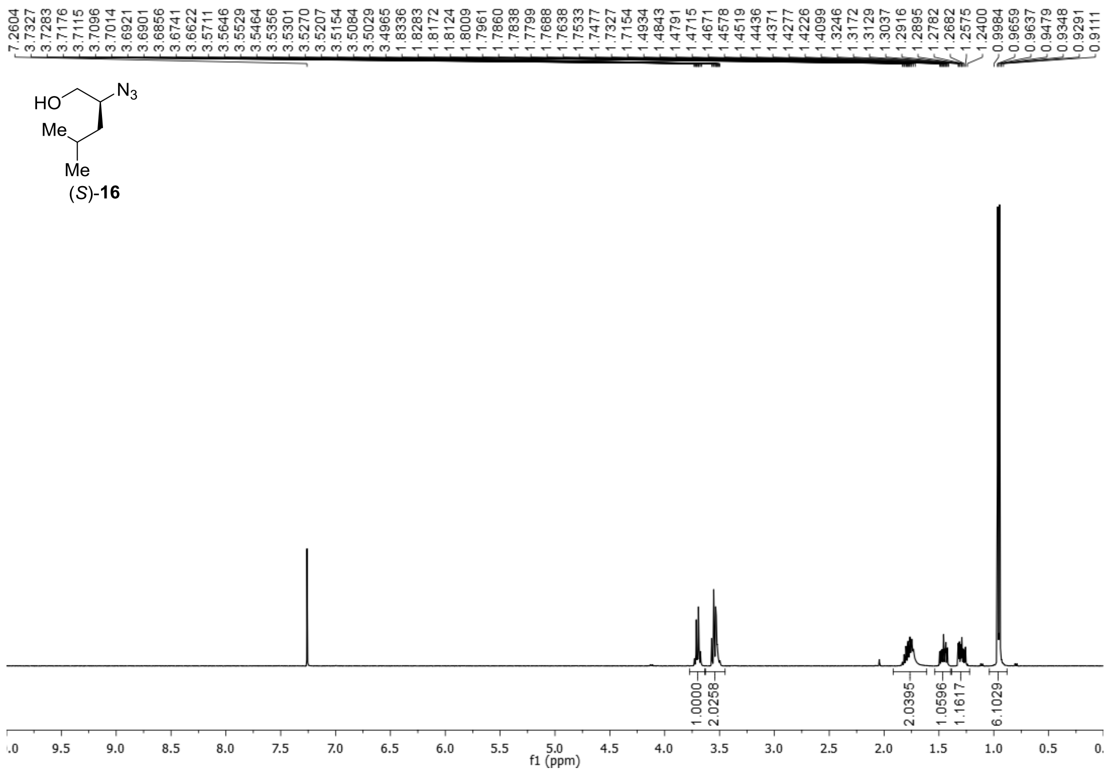

Supplementary Figure 83. ^{13}C NMR spectrum in $CDCl_3$, 101 MHz, of (*S*)-**16**.

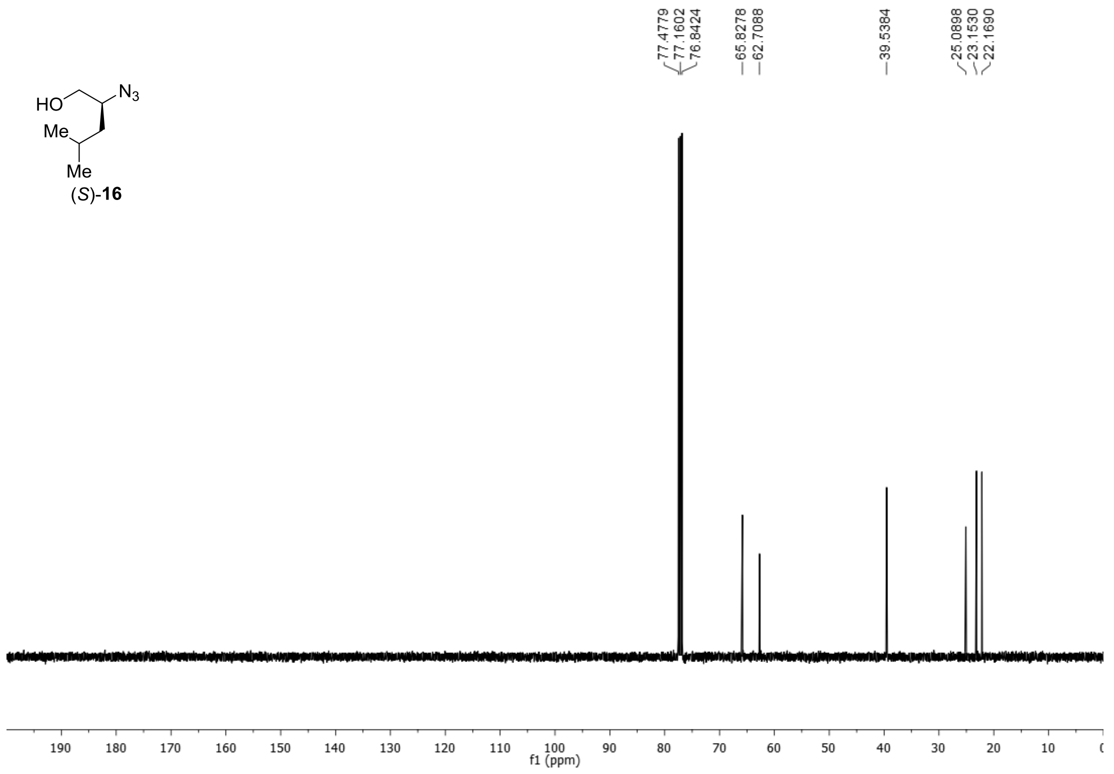

Supplementary Figure 84. ^{1}H NMR spectrum in $CDCl_3$, 400 MHz, of (*R*)-**16**.

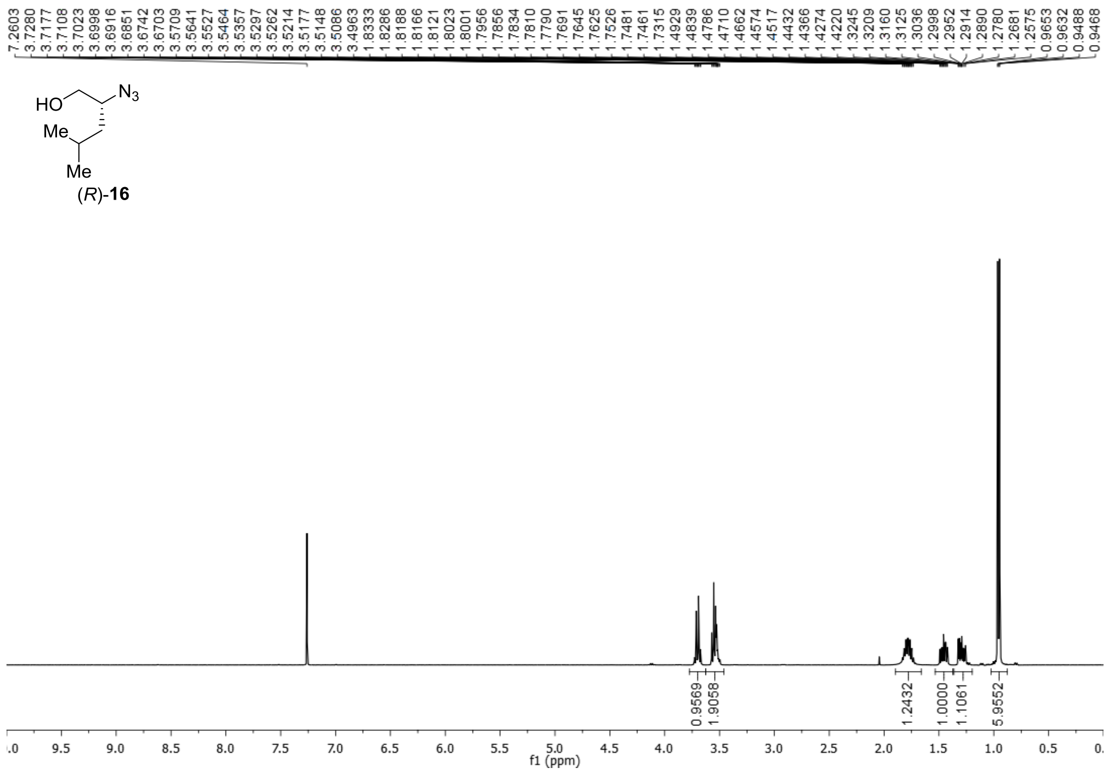

Supplementary Figure 85. ^{13}C NMR spectrum in $CDCl_3$, 101 MHz, of (*R*)-**16**.

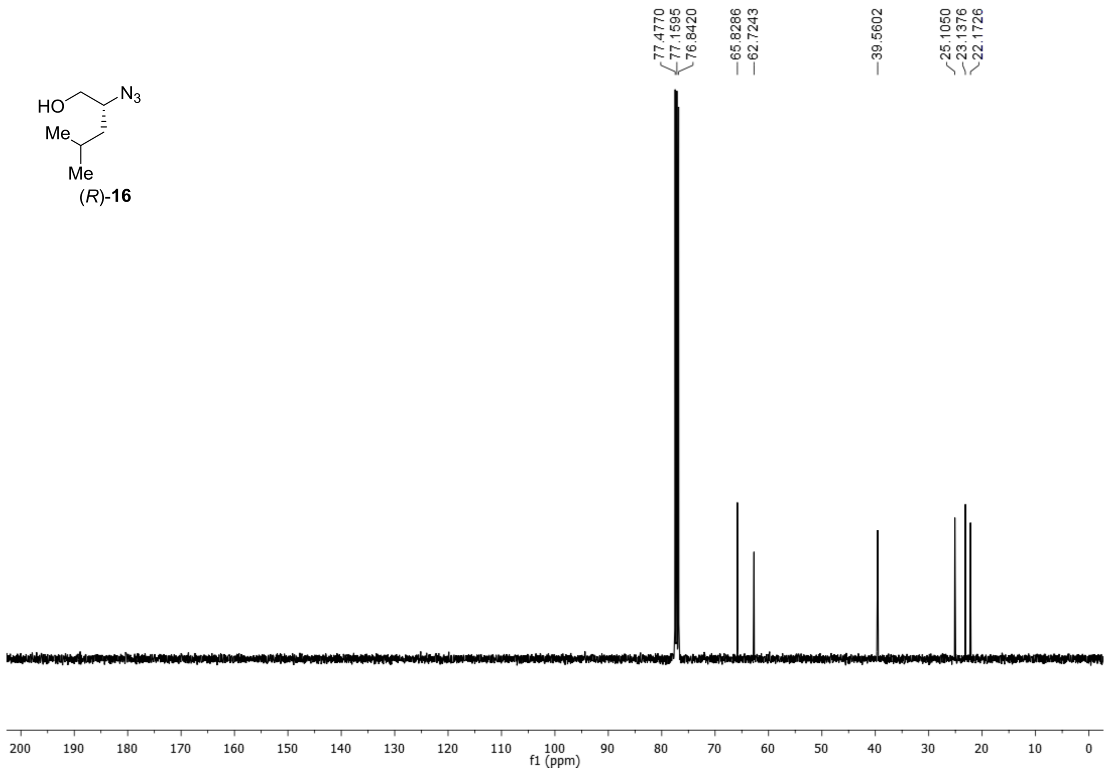

Supplementary Figure 86. ^{1}H NMR spectrum in $CDCl_3$, 600 MHz, of **A1** and **B1**.

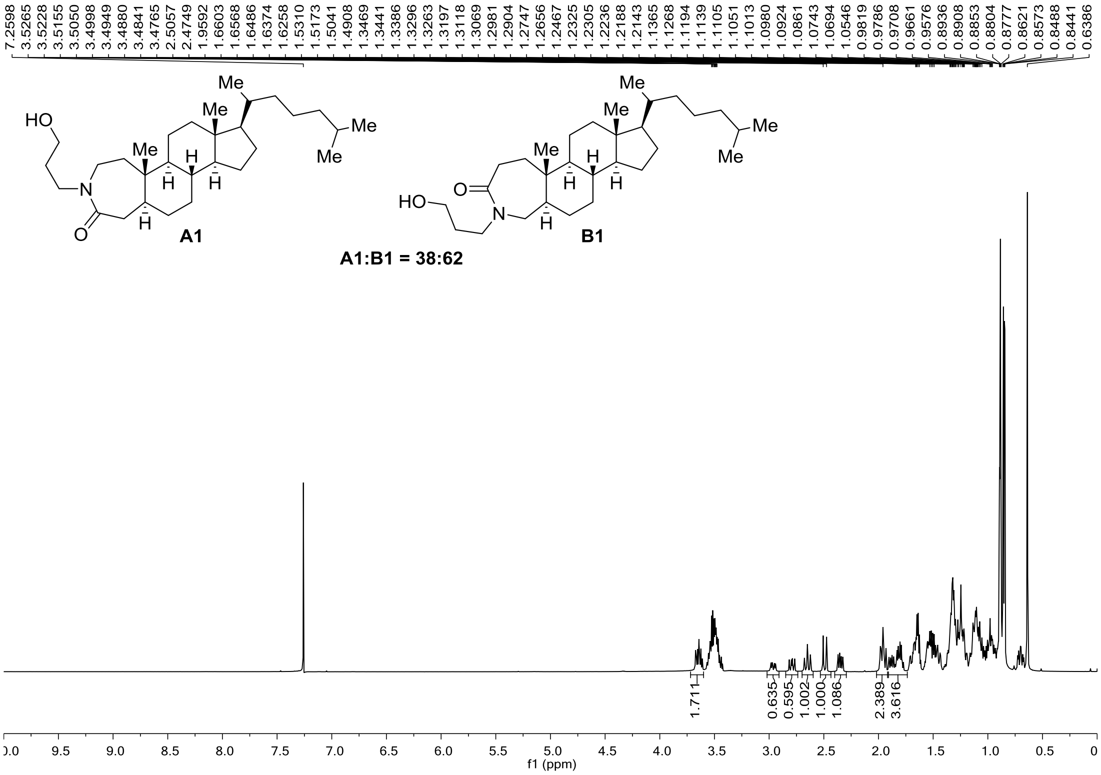

Supplementary Figure 87. ^{13}C NMR spectrum in $CDCl_3$, 151 MHz, of **A1** and **B1**.

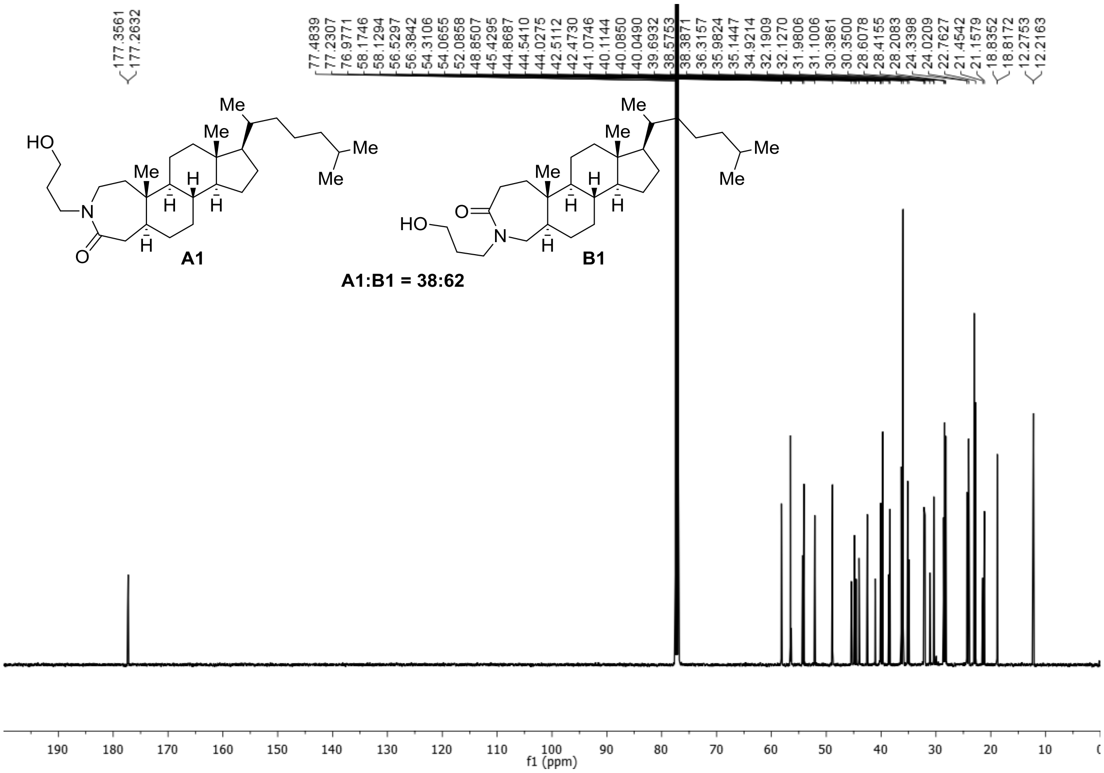

Supplementary Figure 88. COSY spectrum of **A1** and **B1**.

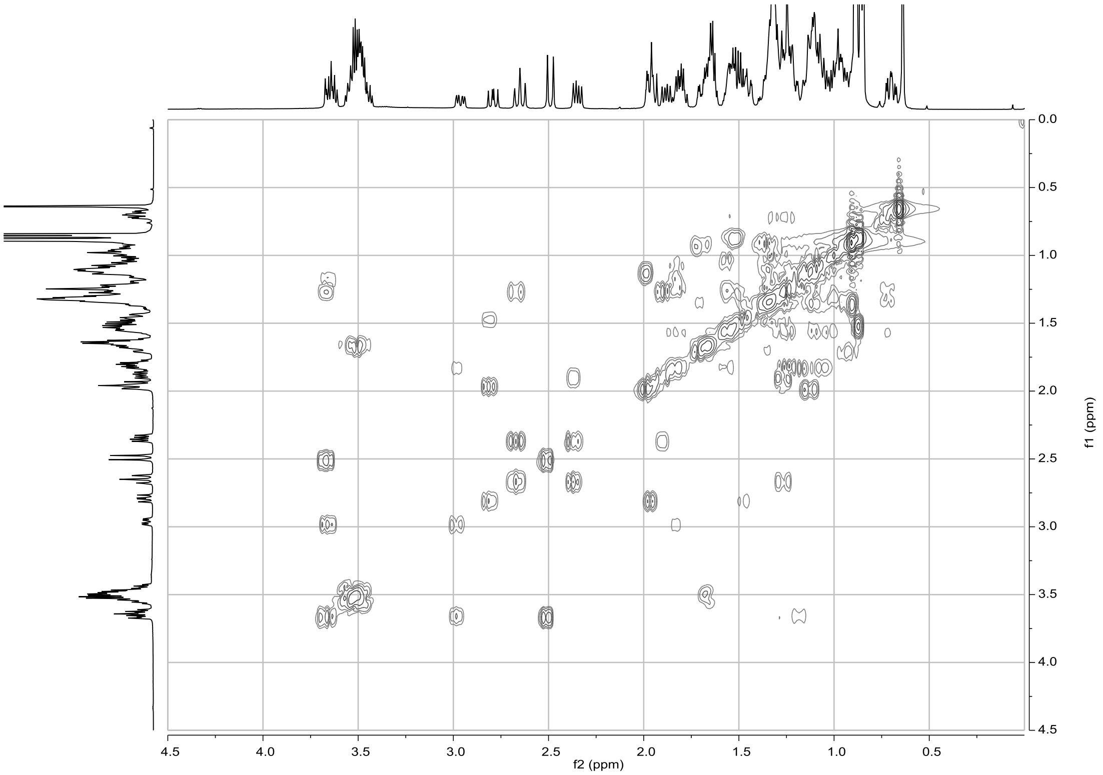

Supplementary Figure 89. HSQC spectrum of **A1** and **B1**.

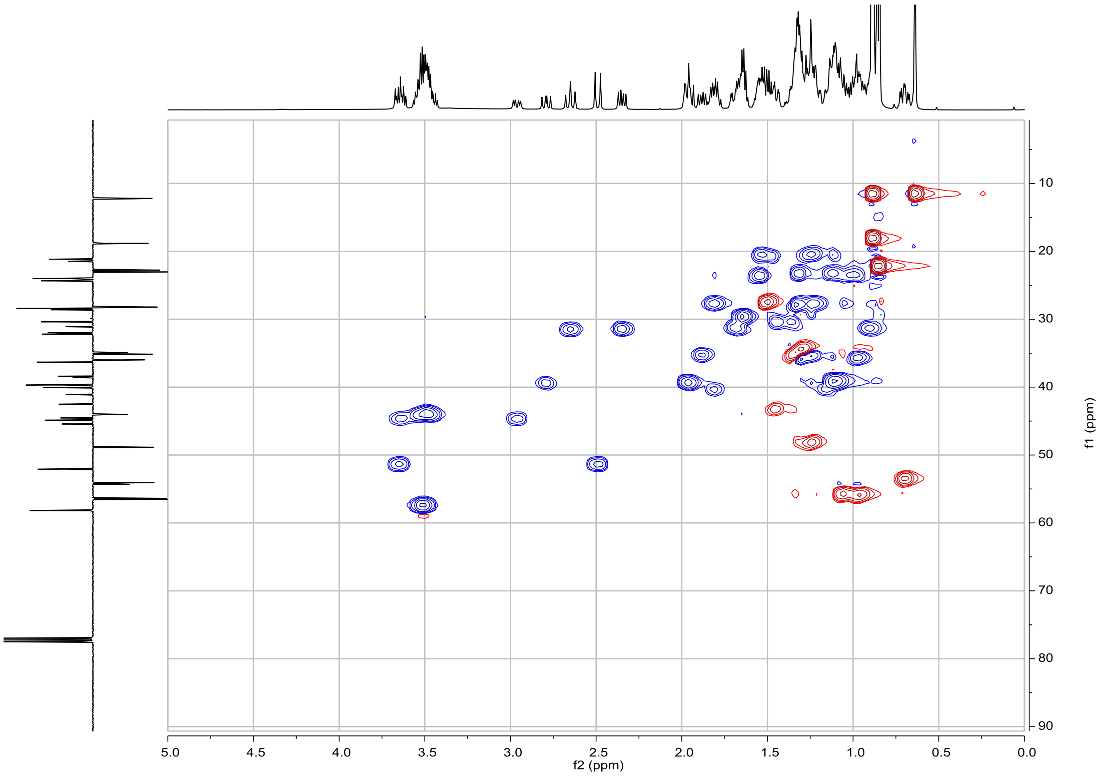

Supplementary Figure 90. ^{1}H NMR spectrum in $CDCl_3$, 400 MHz, of **A2** and **B2**.

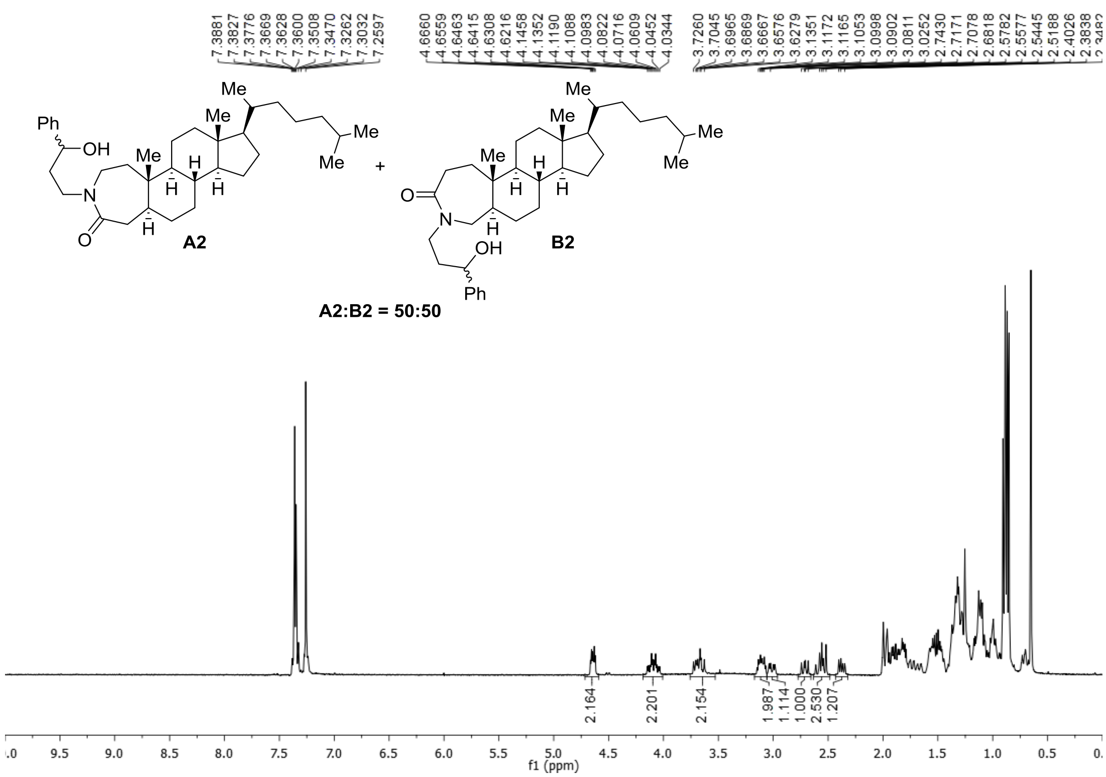

Supplementary Figure 91. ^{13}C NMR spectrum in $CDCl_3$, 101 MHz, of **A2** and **B2**.

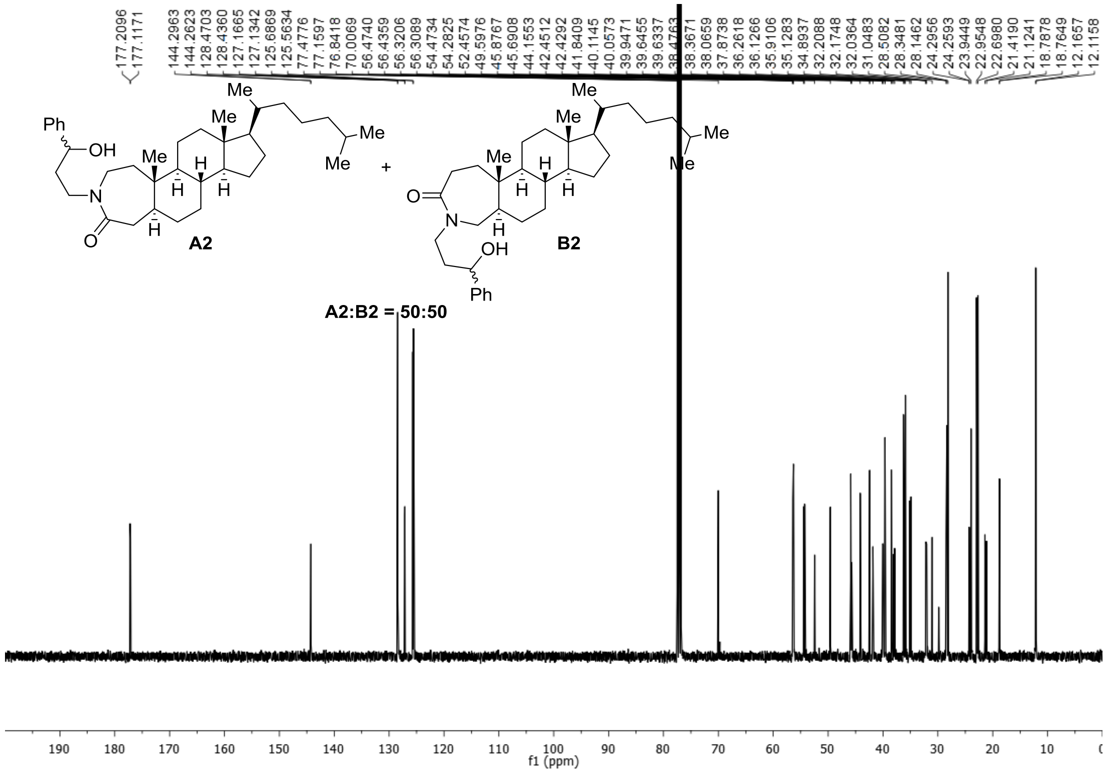

Supplementary Figure 92. ^{1}H NMR spectrum in $CDCl_3$, 400 MHz, of **A2**.

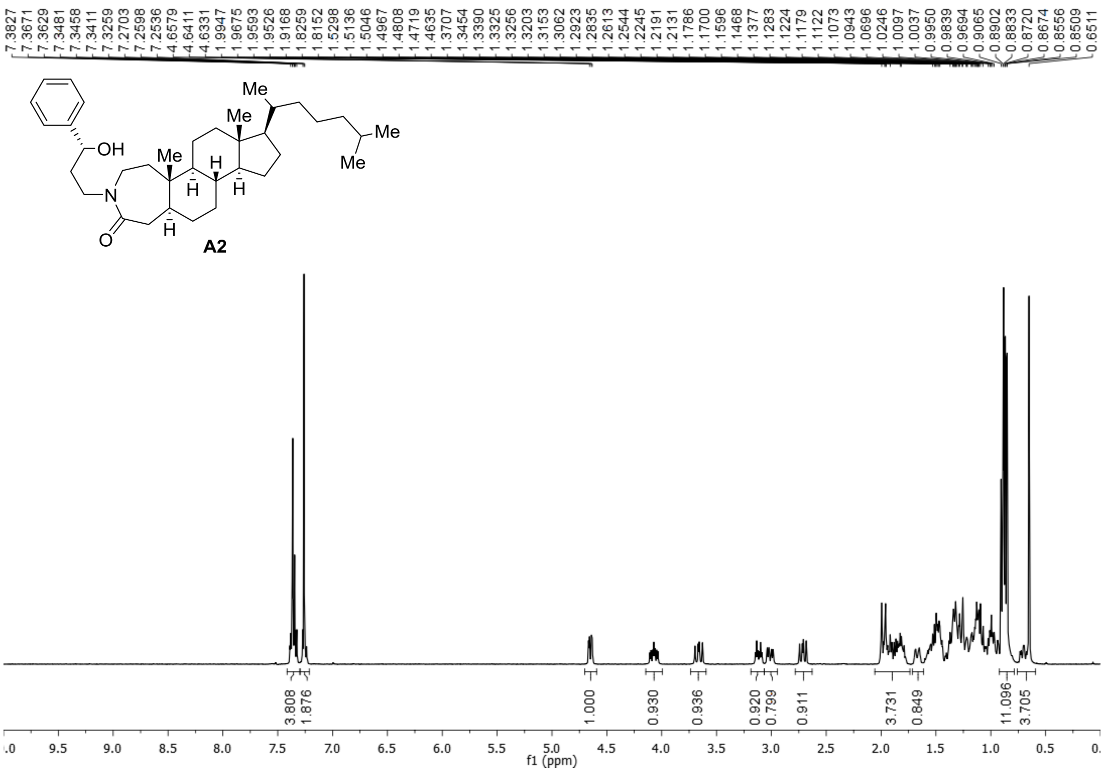

Supplementary Figure 93. ^{13}C NMR spectrum in $CDCl_3$, 101 MHz, of **A2**.

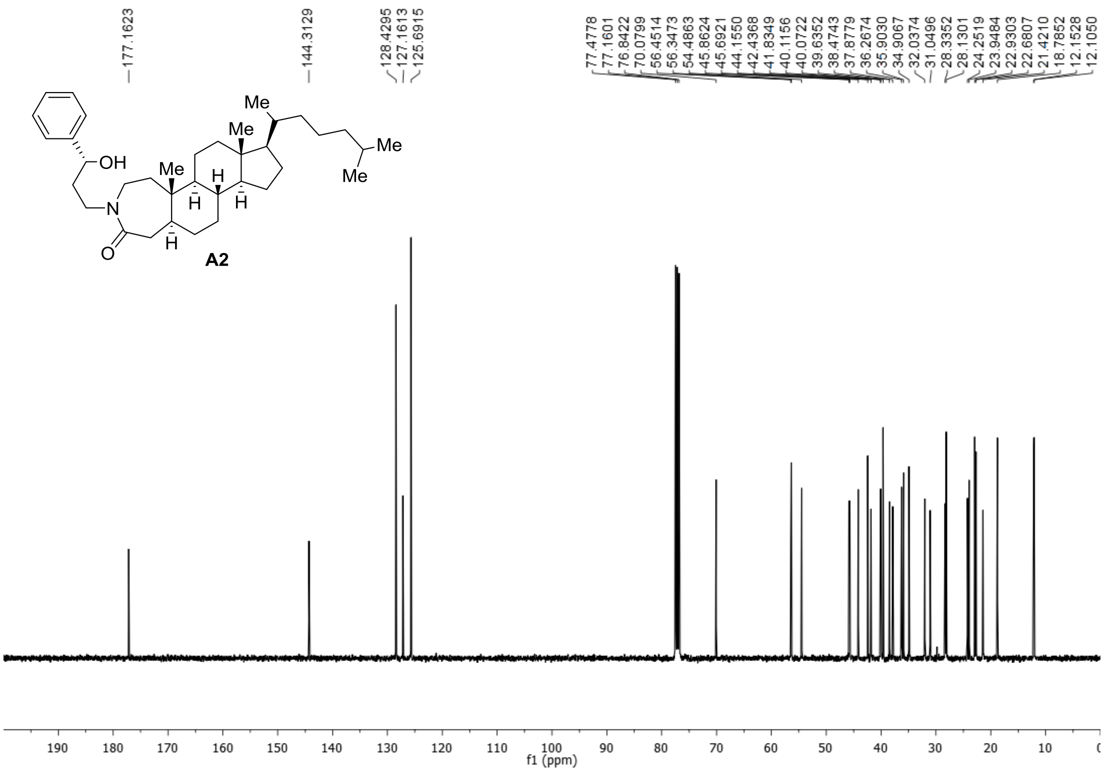

Supplementary Figure 94. ^{13}C NMR (APT) spectrum in $CDCl_3$, 101 MHz, of **A2**.

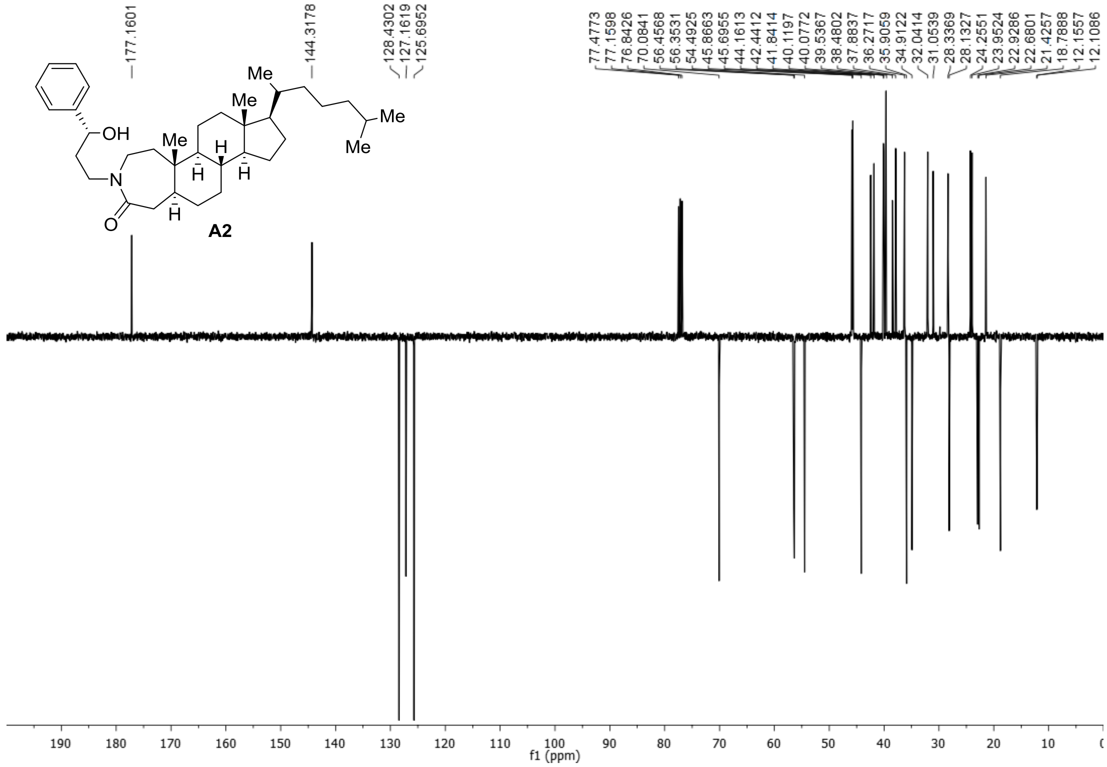

Supplementary Figure 95. HSQC spectrum of **A2**.

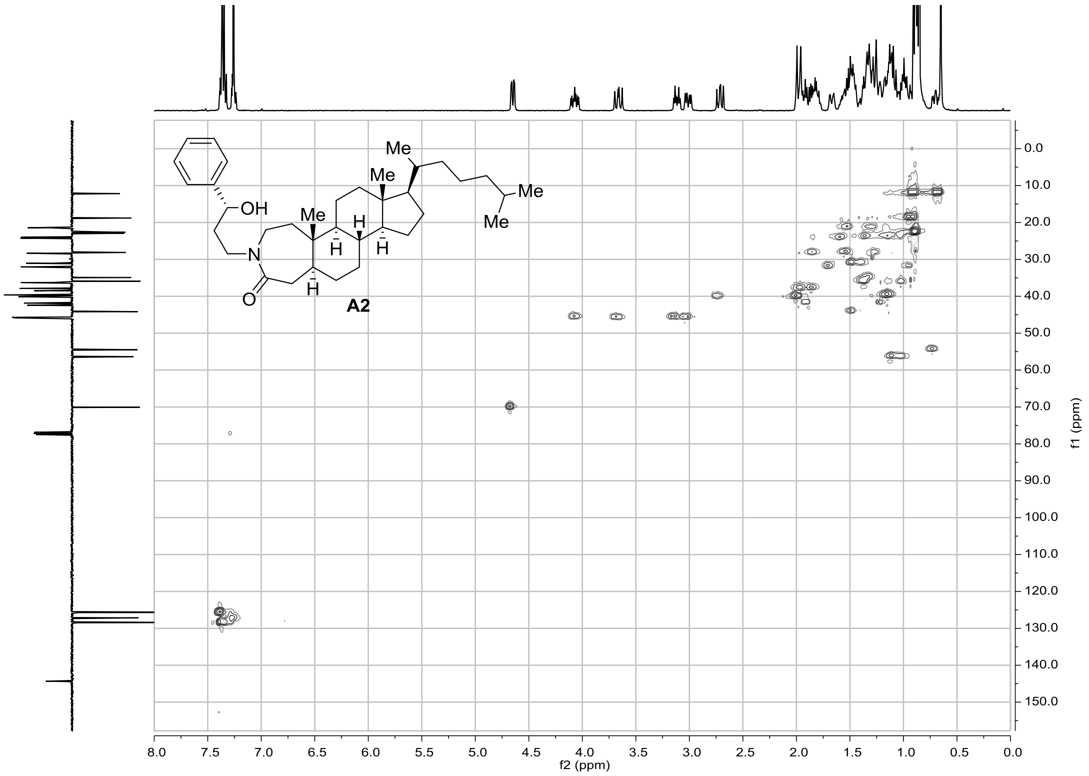

Supplementary Figure 96. ^{1}H NMR spectrum in $CDCl_3$, 400 MHz, of **A3**.

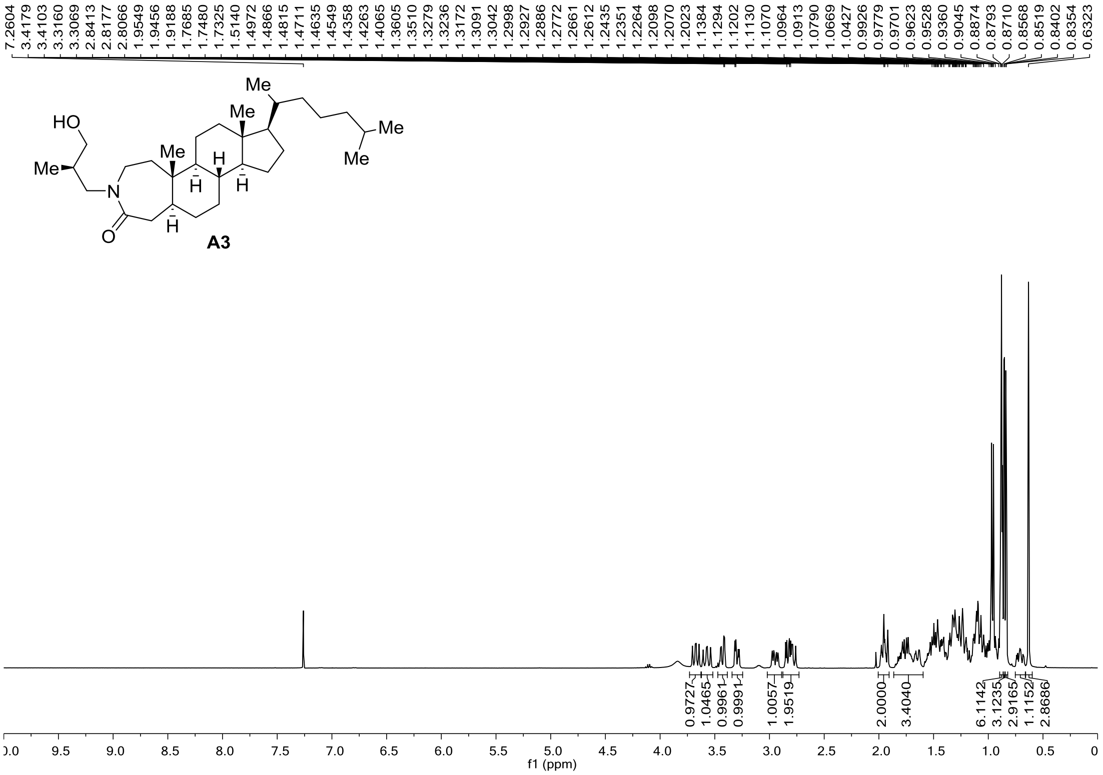

Supplementary Figure 97. ^{13}C NMR spectrum in $CDCl_3$, 101 MHz, of **A3**.

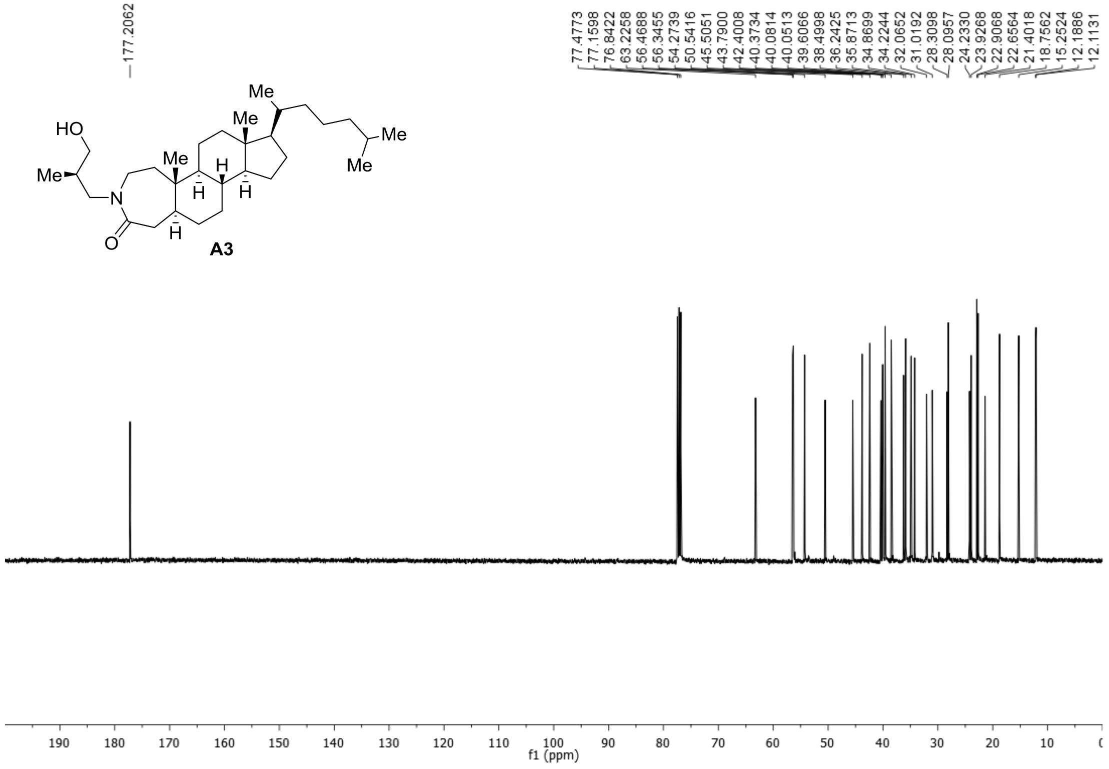

Supplementary Figure 98. ^{1}H NMR spectrum in $CDCl_3$, 400 MHz, of **A4**.

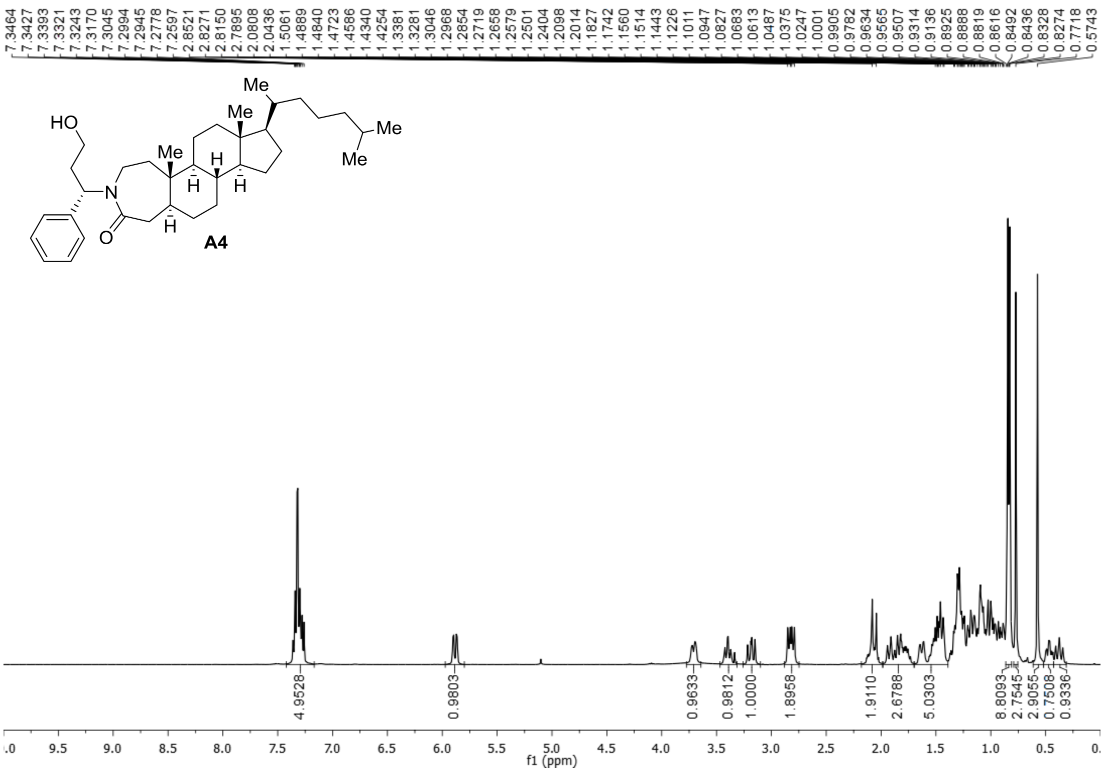

Supplementary Figure 99. ^{13}C NMR spectrum in $CDCl_3$, 101 MHz, of **A4**.

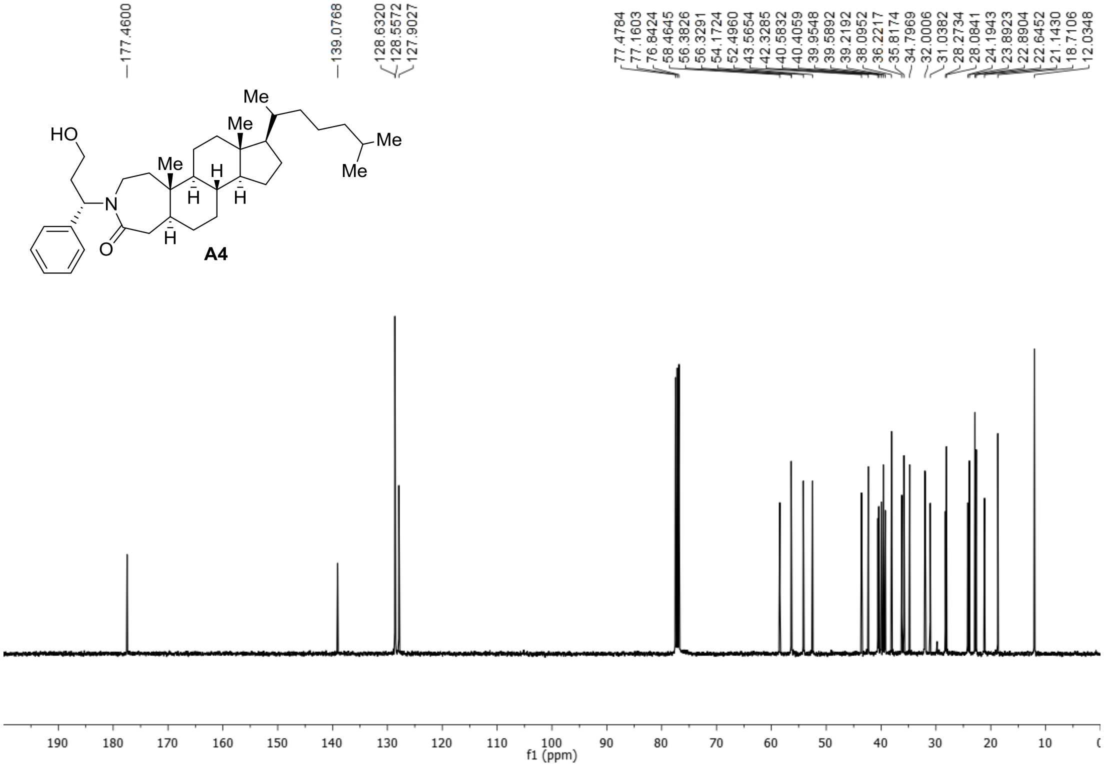

Supplementary Figure 100. ^{13}C NMR (APT) spectrum in $CDCl_3$, 101 MHz, of **A4**.

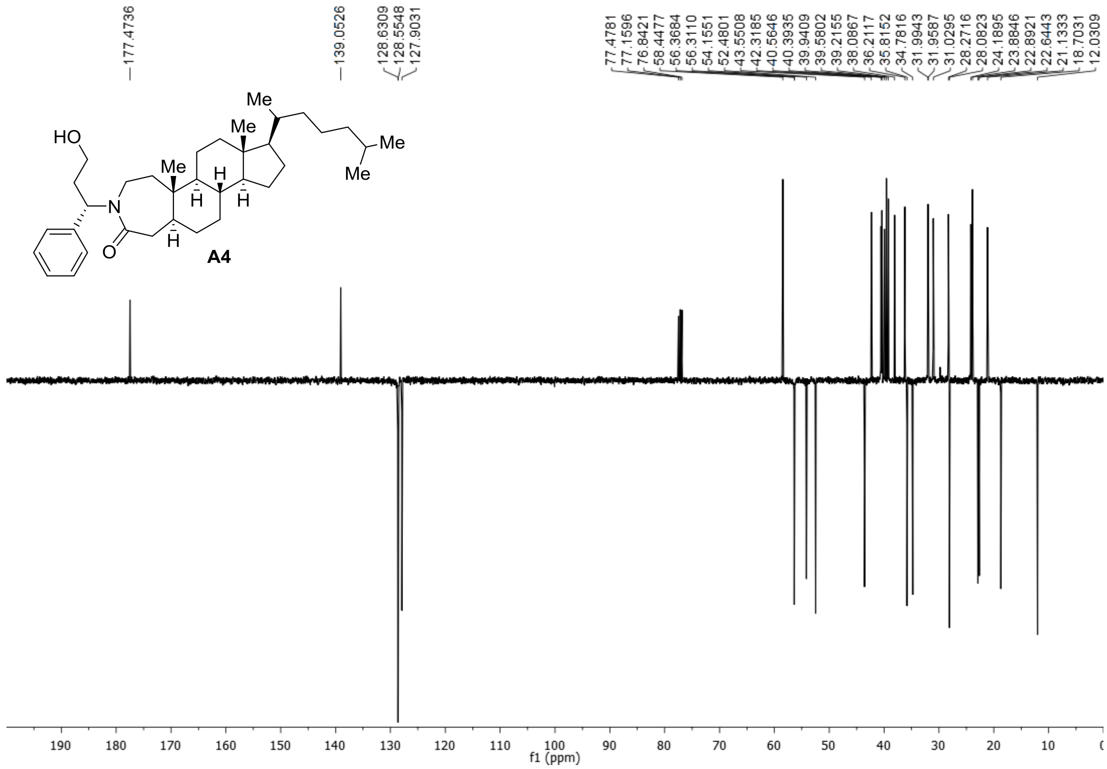

Supplementary Figure 101. HSQC spectrum of **A4**.

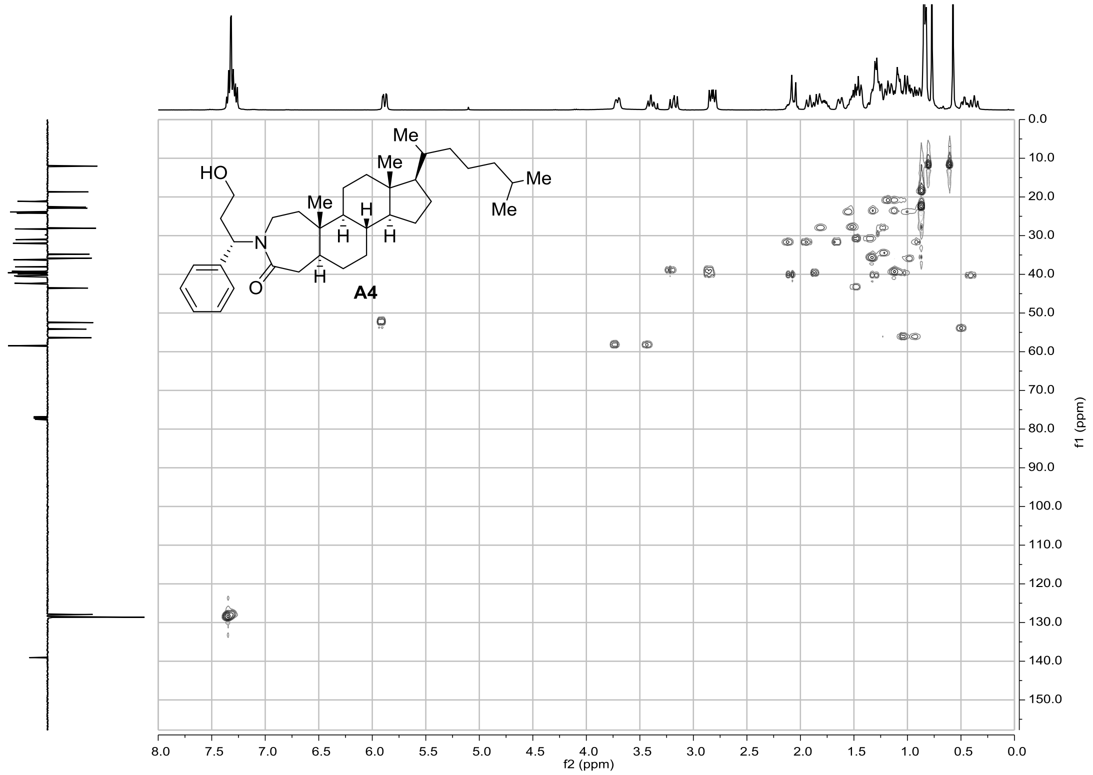

Supplementary Figure 102. ^{1}H NMR spectrum in $CDCl_3$, 400 MHz, of **A5**.

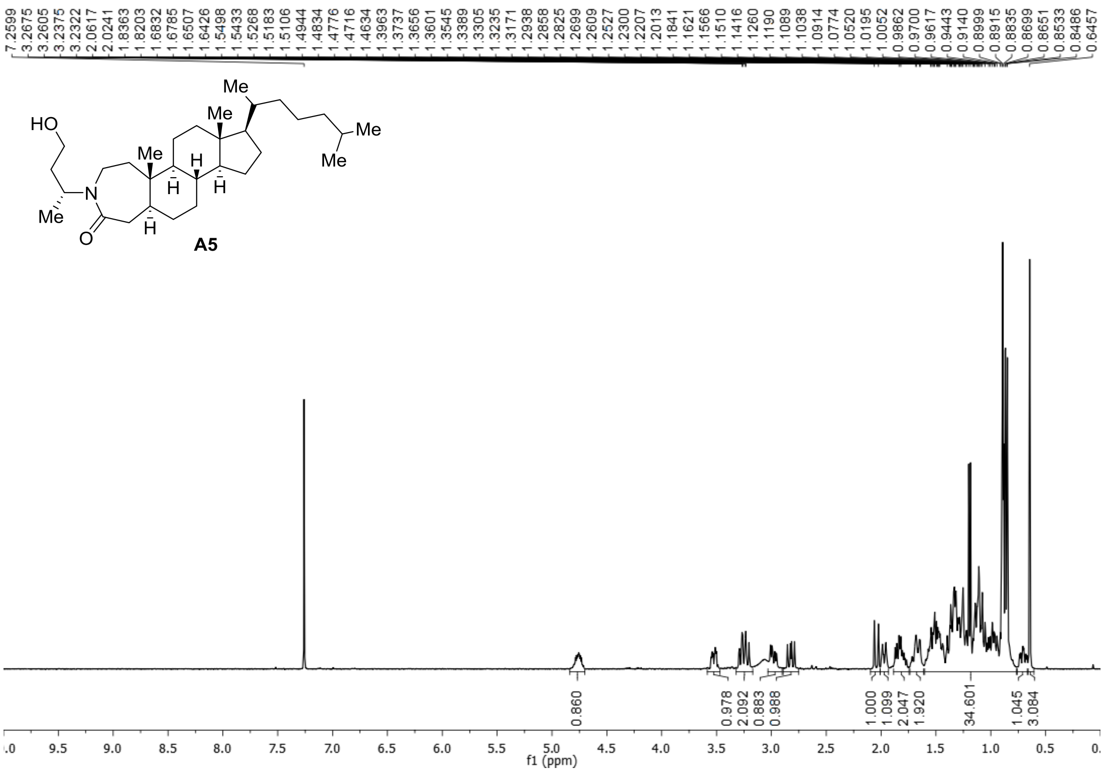

Supplementary Figure 103. ^{13}C NMR spectrum in $CDCl_3$, 101 MHz, of **A5**.

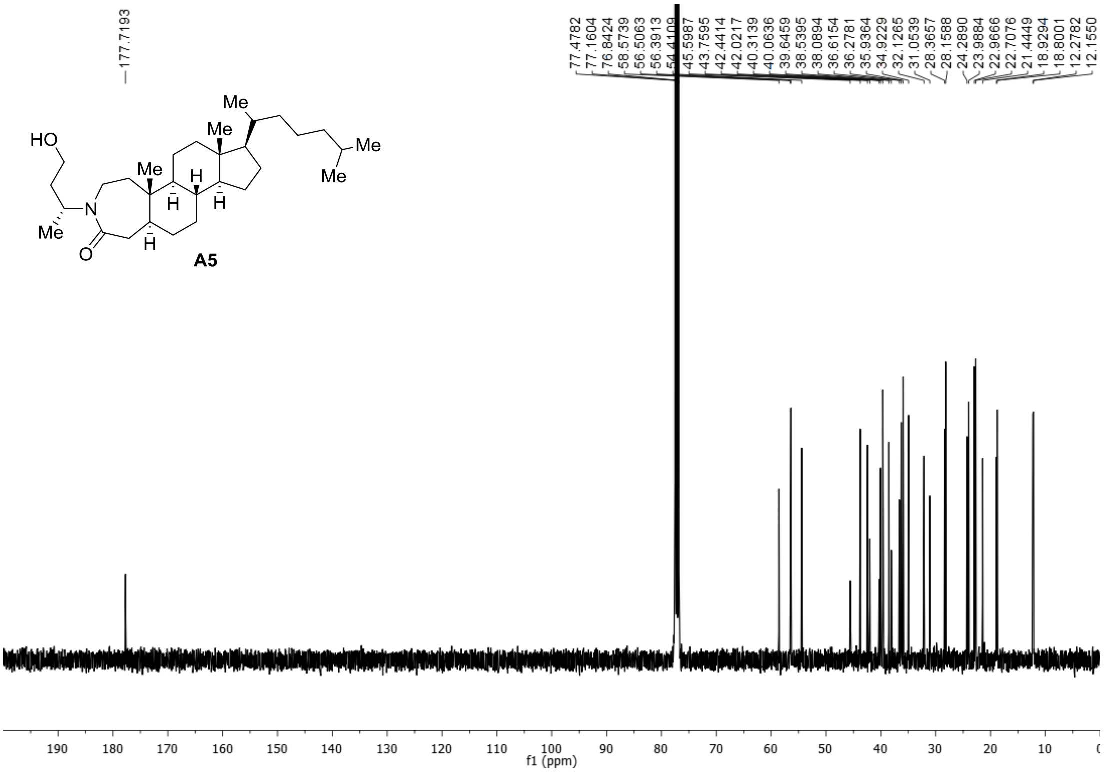

Supplementary Figure 104. ^{1}H NMR spectrum in $CDCl_3$, 400 MHz, of **A6** and **B6**.

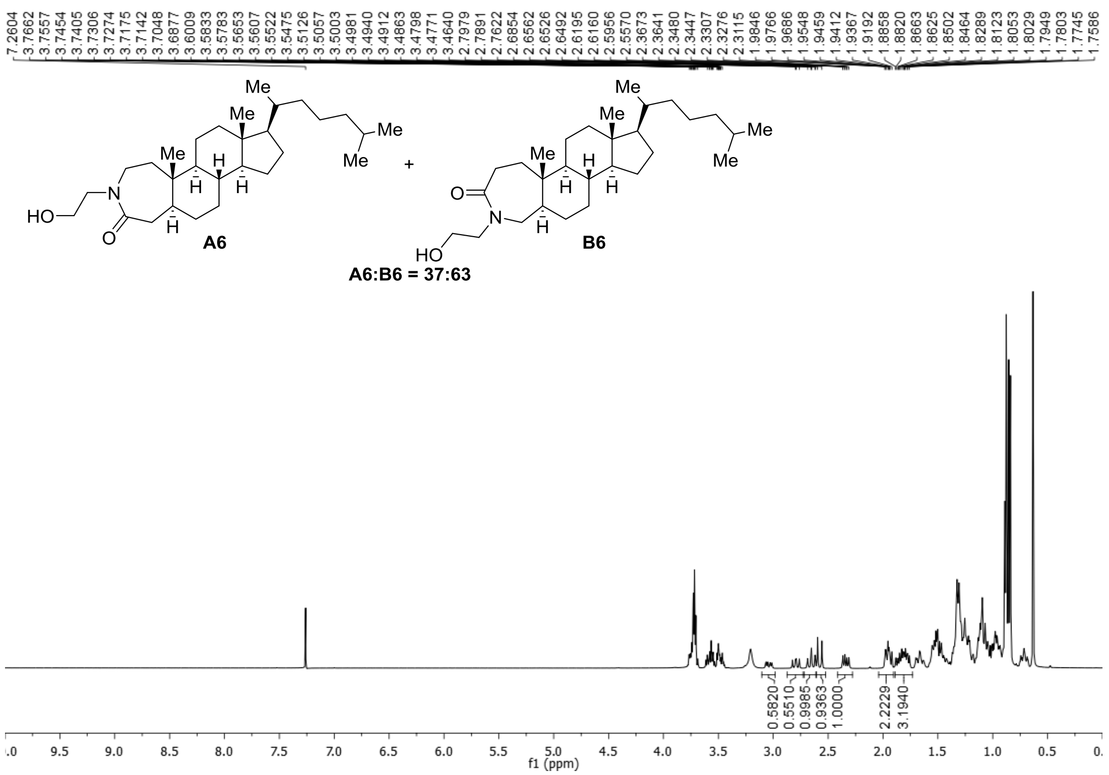

Supplementary Figure 105. ^{13}C NMR spectrum in $CDCl_3$, 101 MHz, of **A6** and **B6**.

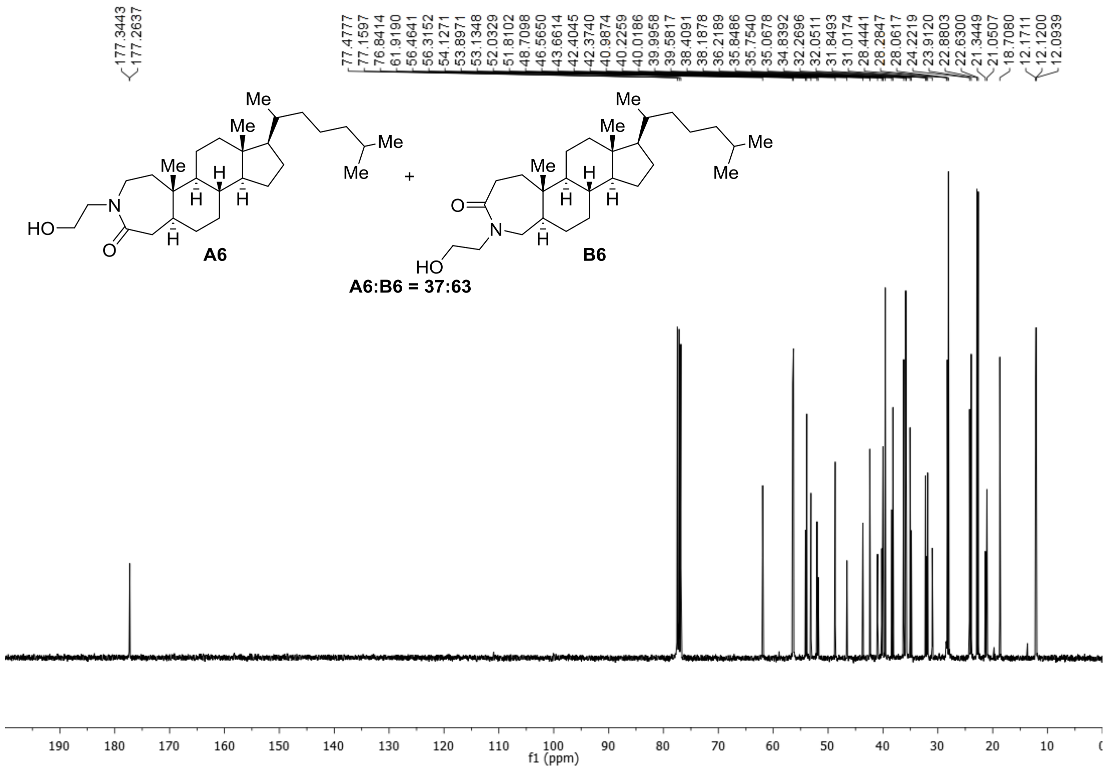

Supplementary Figure 106. COSY of **A6** and **B6**.

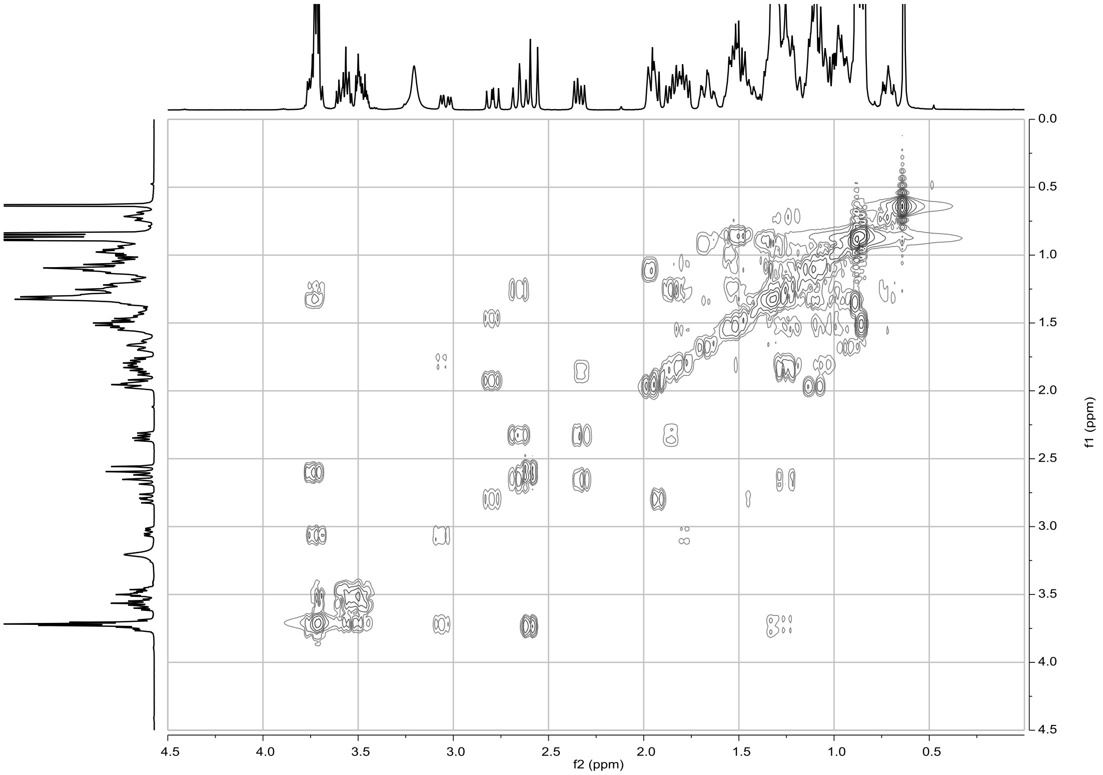

Supplementary Figure 107. HSQC of **A6** and **B6**.

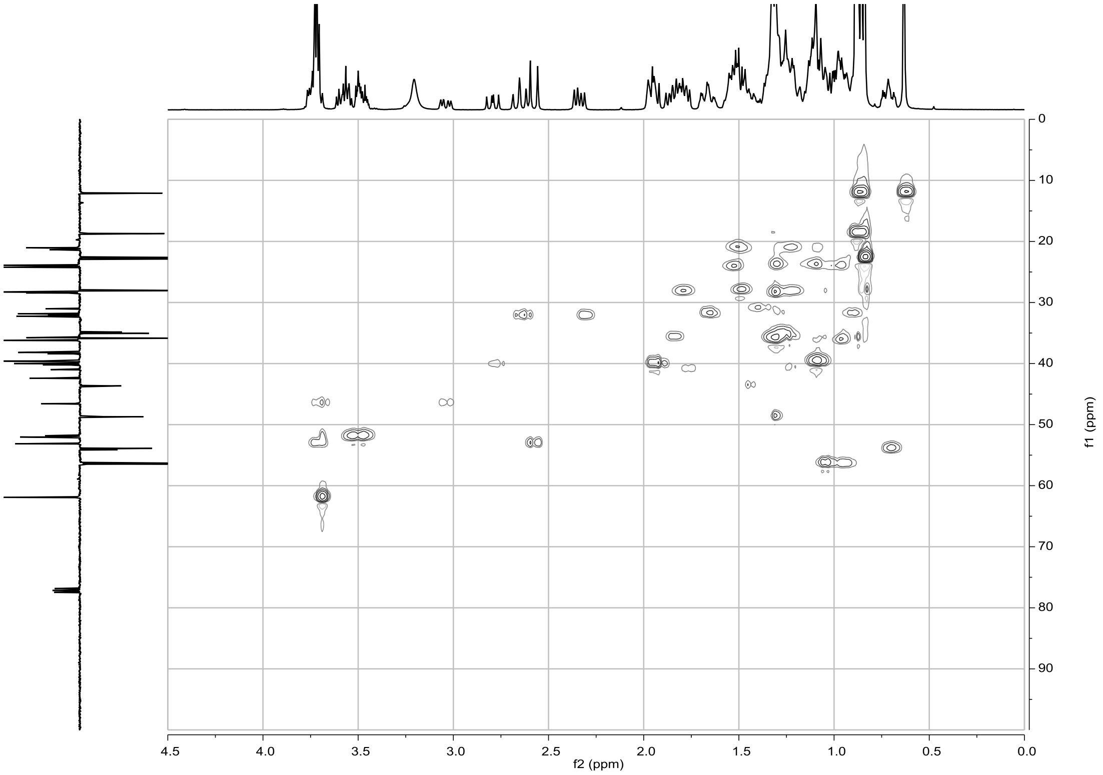

Supplementary Figure 108. ^{1}H NMR spectrum in $CDCl_3$, 400 MHz, of **A7** and **B7**.

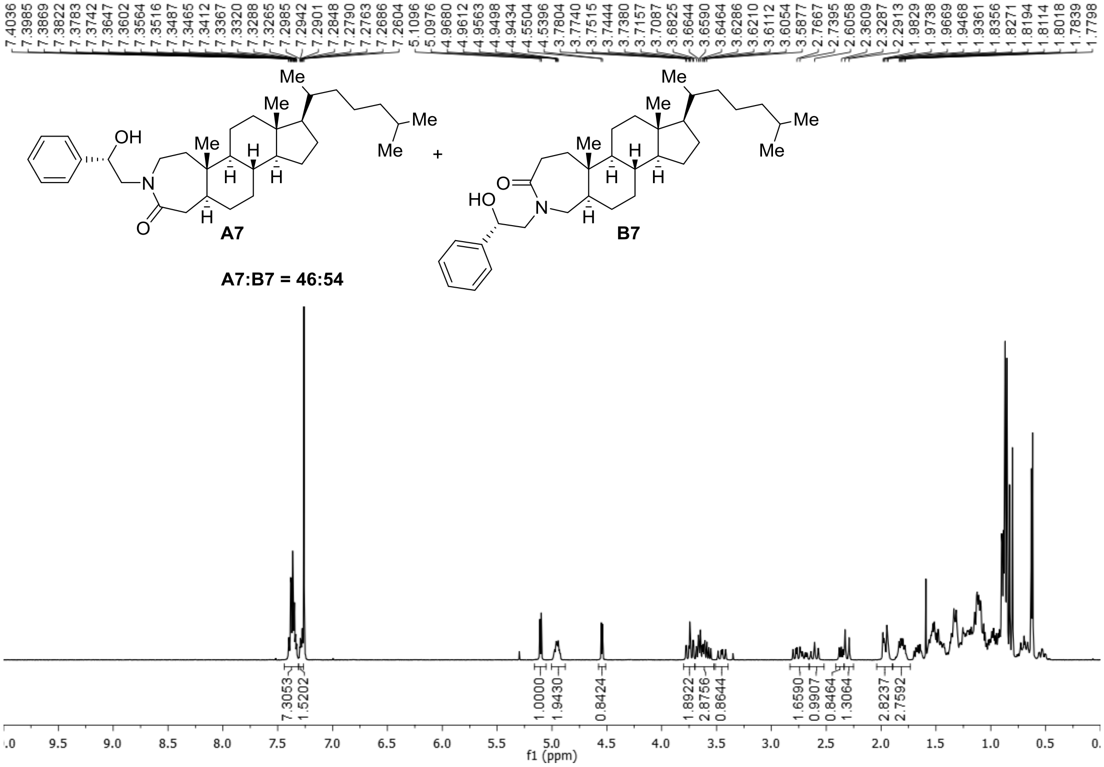

Supplementary Figure 109. ^{13}C NMR spectrum in $CDCl_3$, 101 MHz, of **A6** and **B6**.

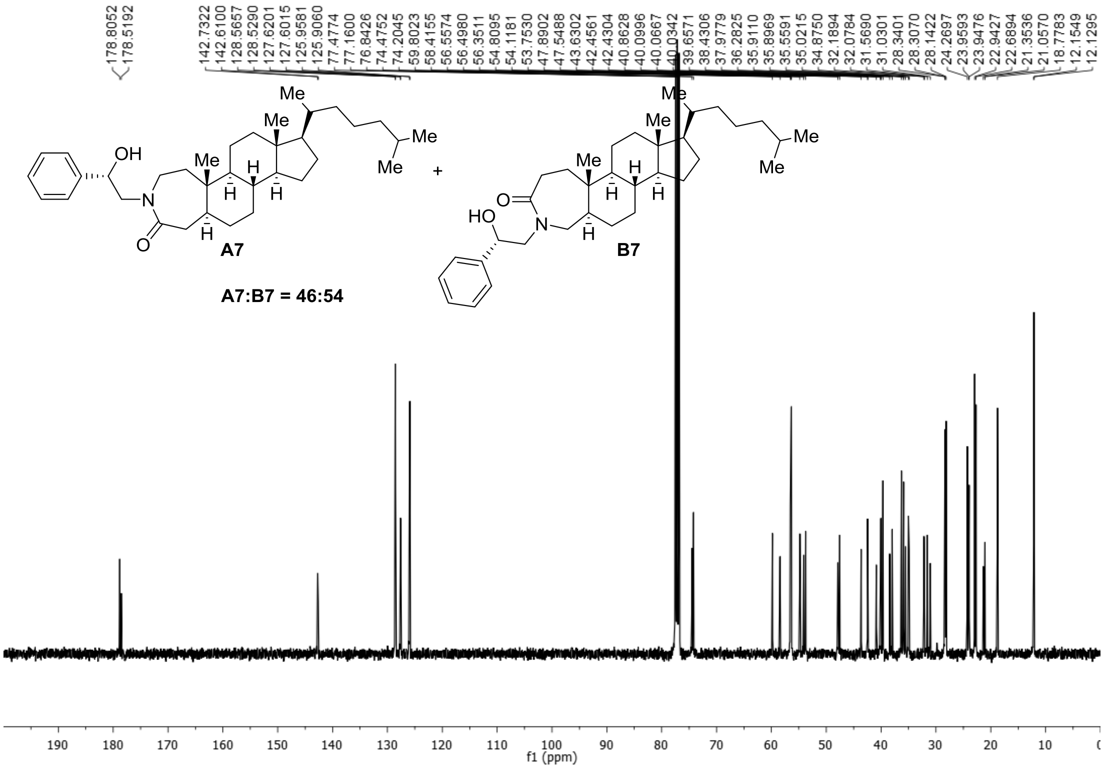

Supplementary Figure 110. ^{1}H NMR spectrum in $CDCl_3$, 400 MHz, of **A8**.

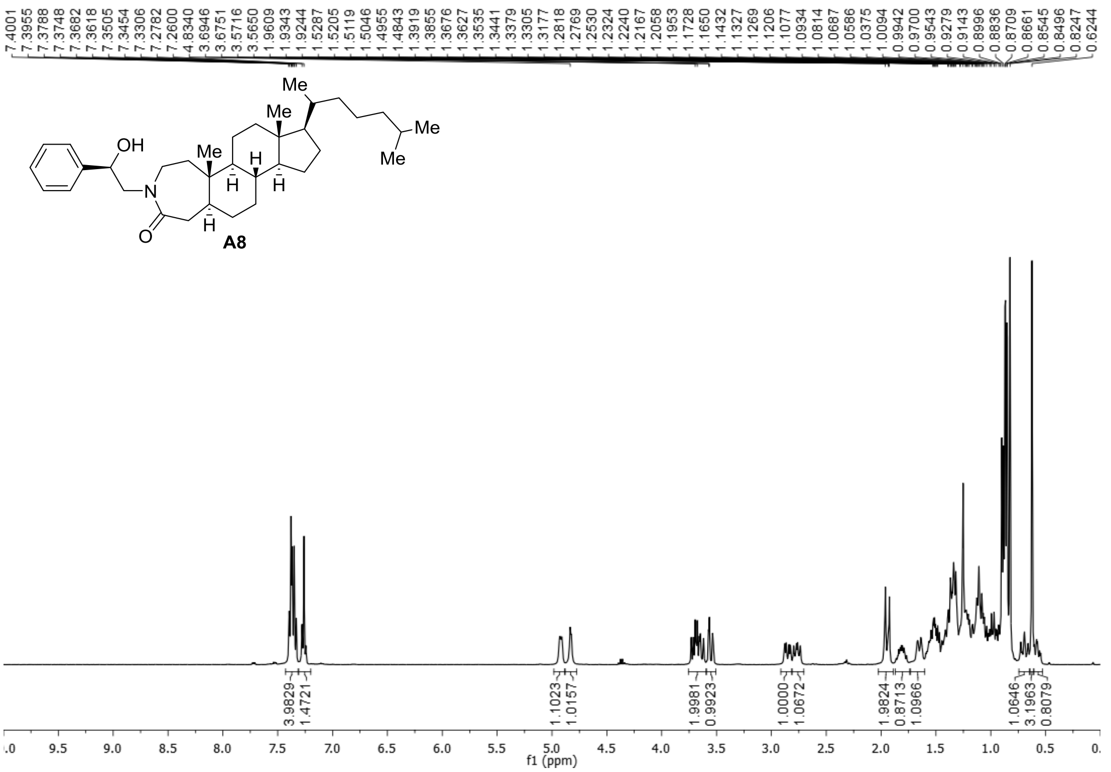

Supplementary Figure 111. ^{13}C NMR spectrum in $CDCl_3$, 101 MHz, of **A8**.

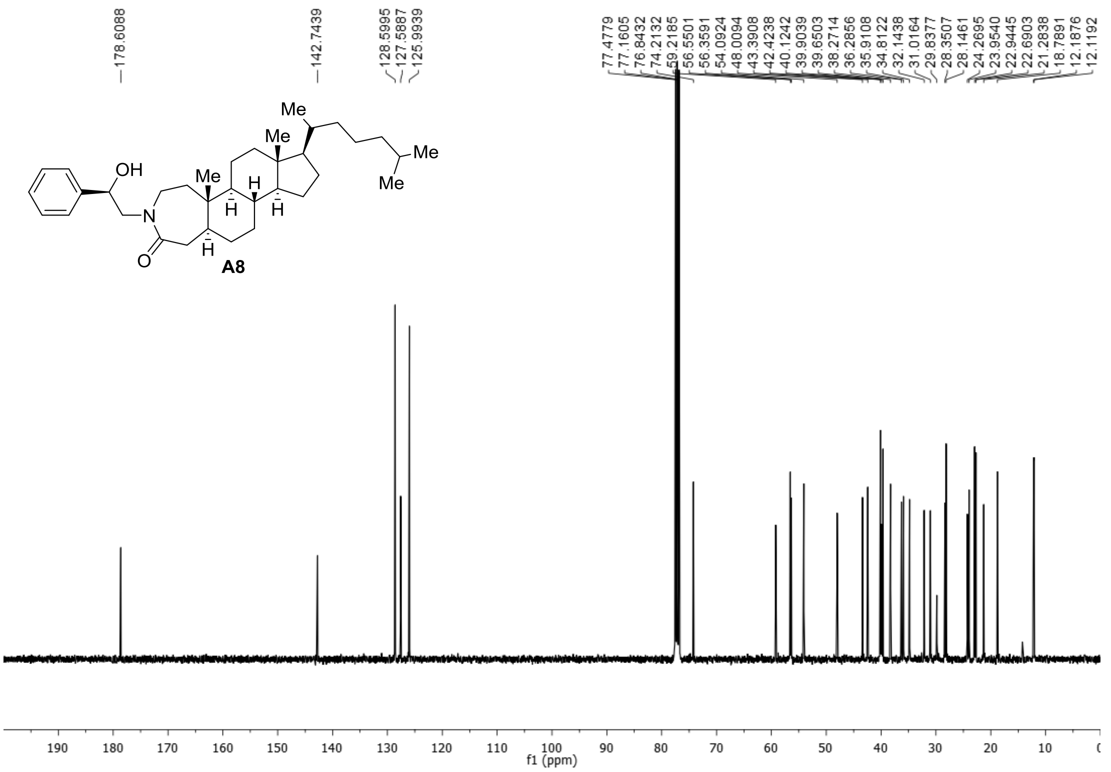

Supplementary Figure 112. ^{1}H NMR spectrum in $CDCl_3$, 400 MHz, of **B8**.

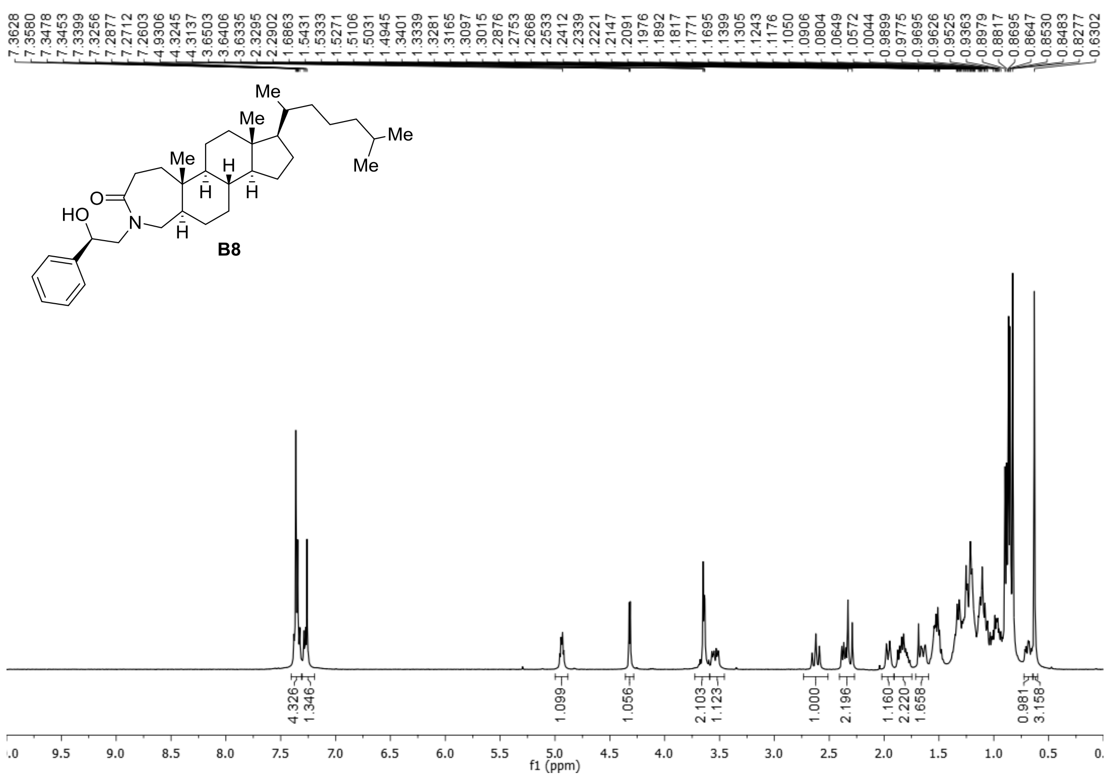

Supplementary Figure 113. ^{13}C NMR spectrum in $CDCl_3$, 101 MHz, of **B8**.

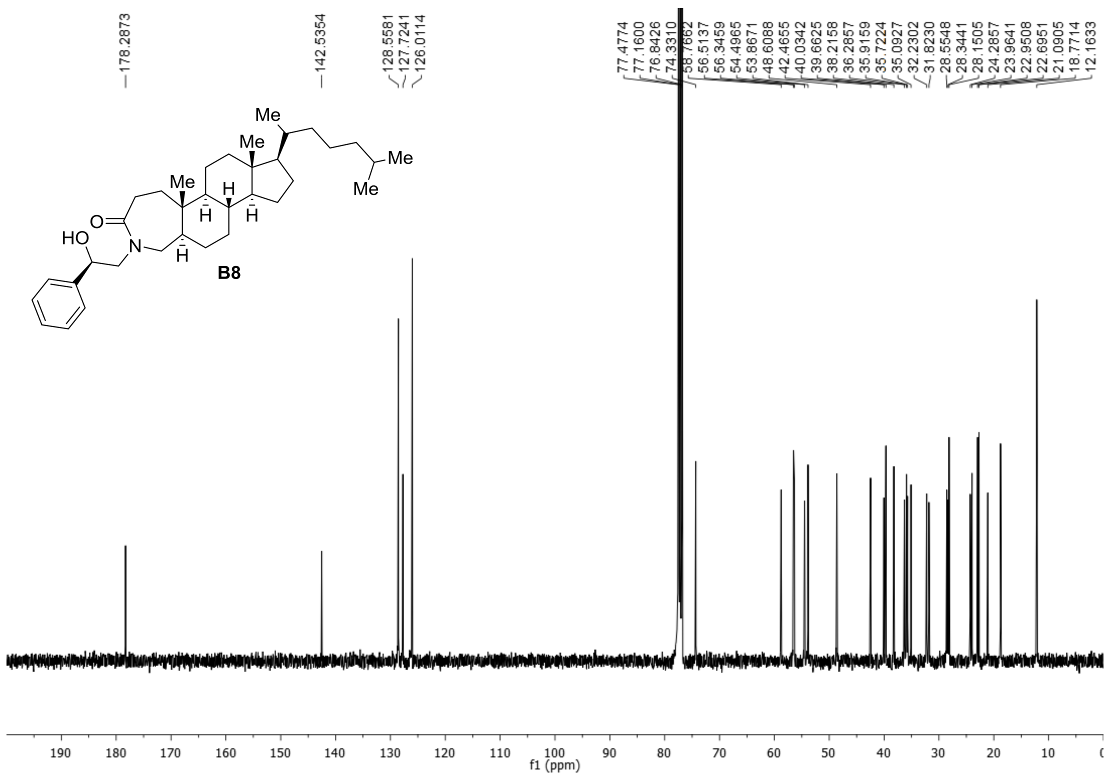

Supplementary Figure 114. ^{1}H NMR spectrum in $CDCl_3$, 400 MHz, of **A9**.

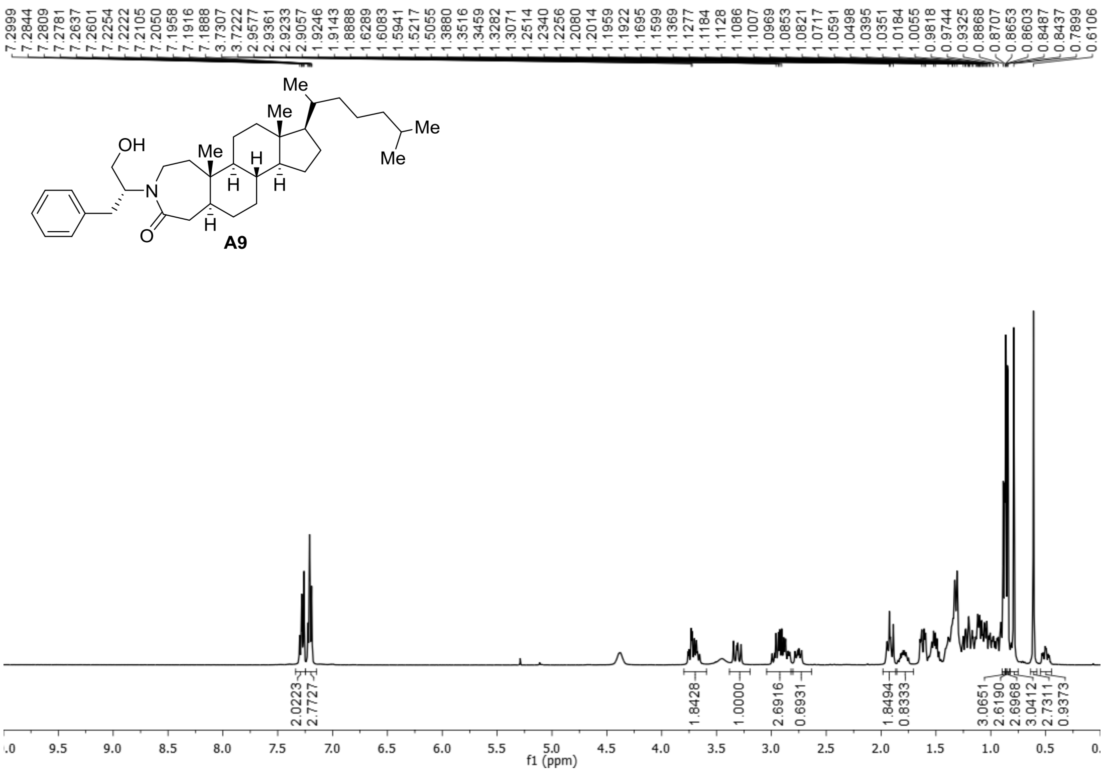

Supplementary Figure 115. ^{13}C NMR spectrum in $CDCl_3$, 126 MHz, of **A9**.

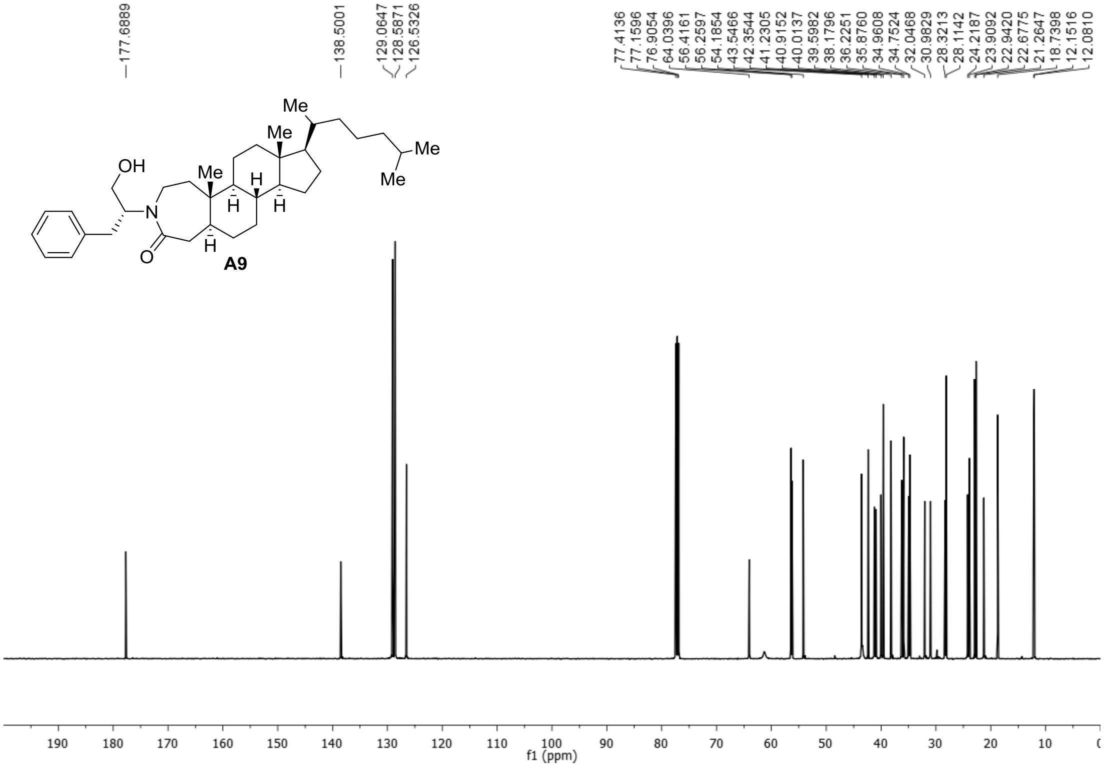

Supplementary Figure 116. ^{1}H NMR spectrum in $CDCl_3$, 400 MHz, of **A10**.

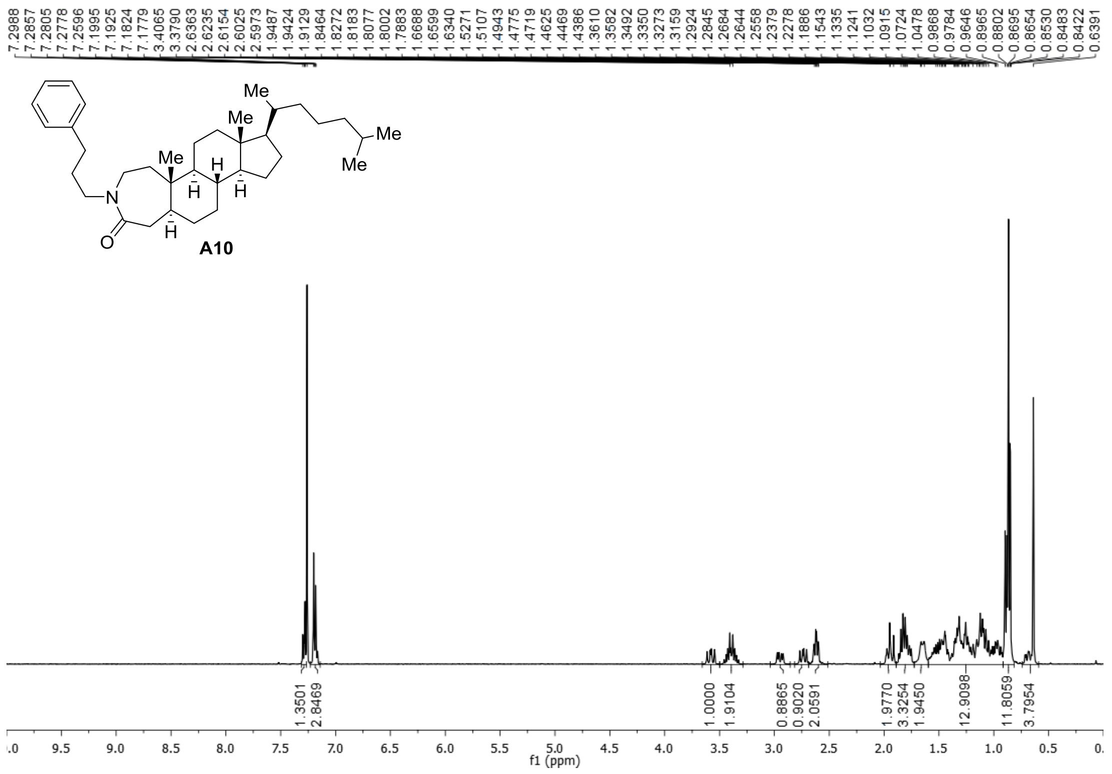

Supplementary Figure 117. ^{13}C NMR spectrum in $CDCl_3$, 101 MHz, of **A10**.

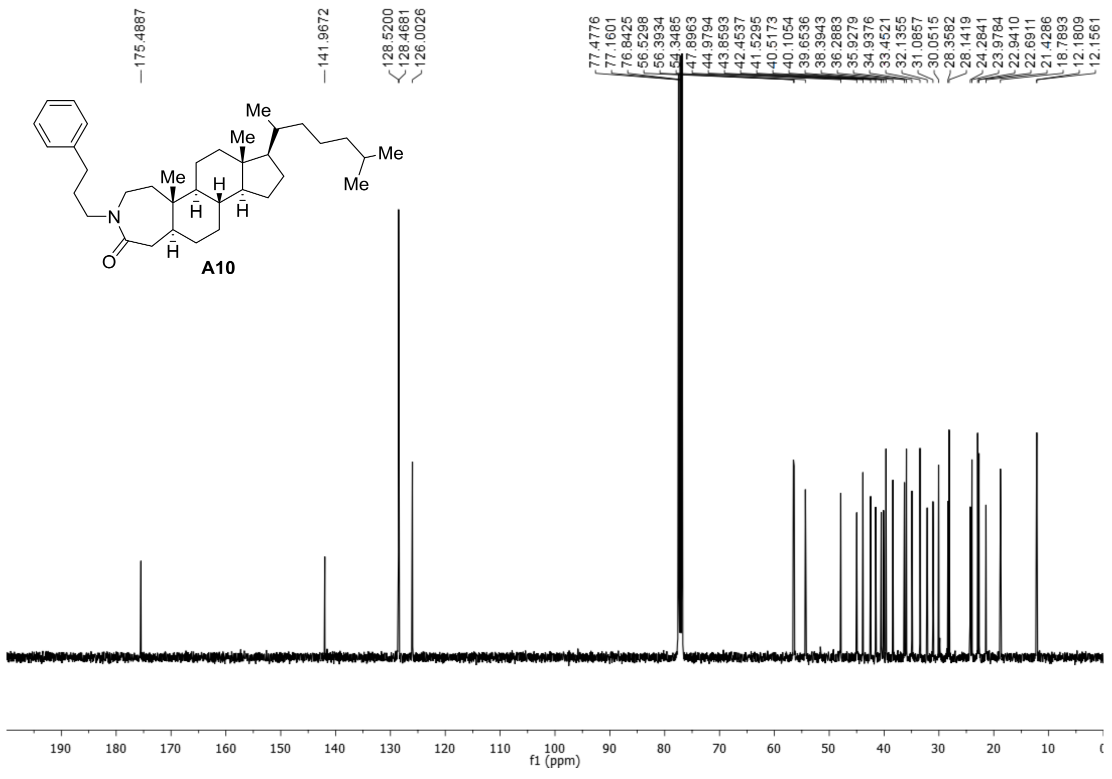

Supplementary Figure 118. ^{1}H NMR spectrum in $CDCl_3$, 400 MHz, of **A11**.

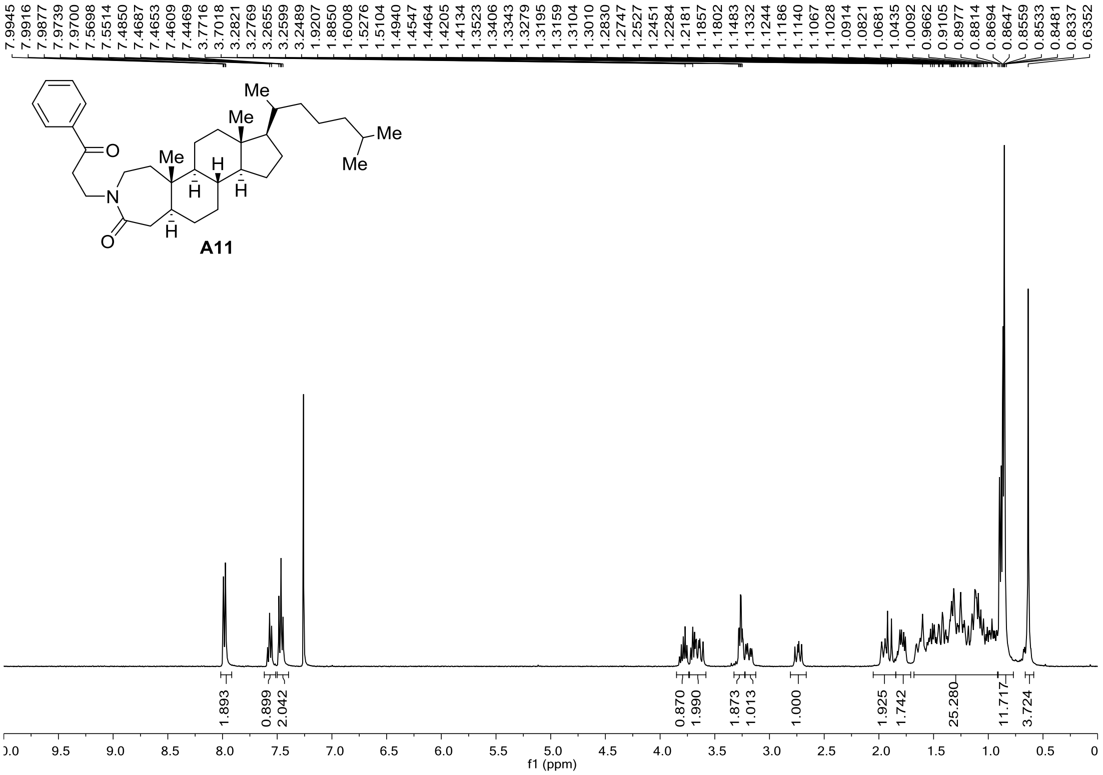

Supplementary Figure 119. ^{13}C NMR spectrum in $CDCl_3$, 101 MHz, of **A11**.

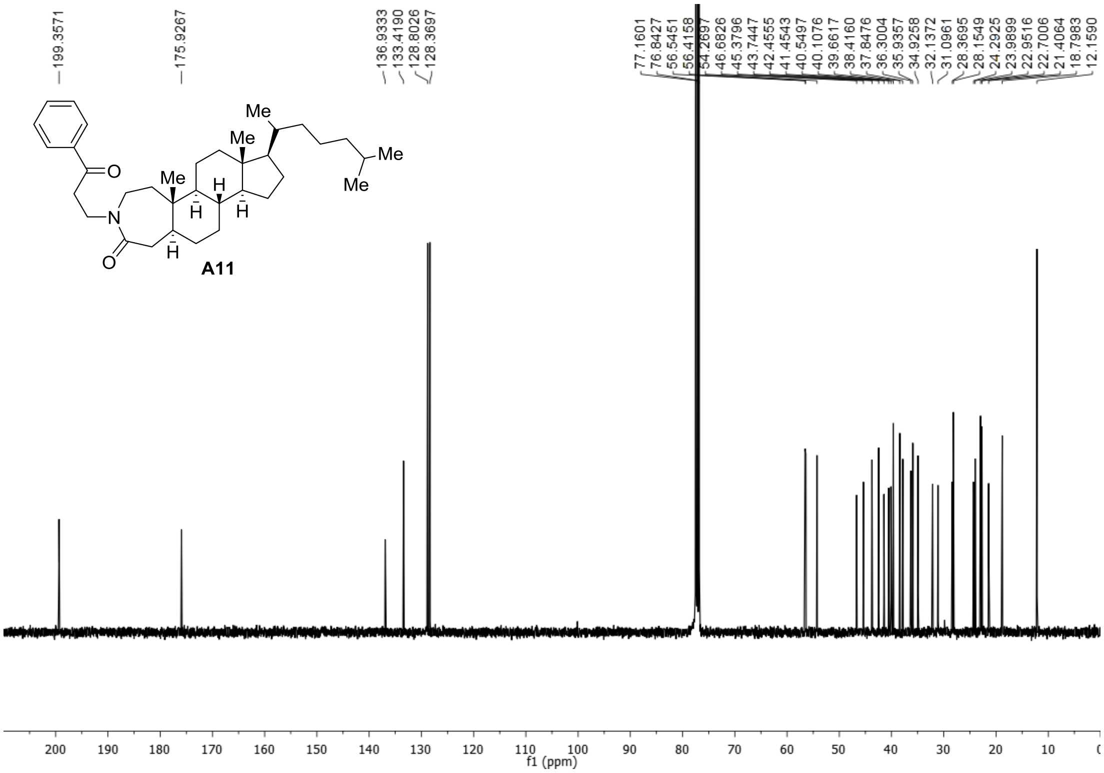

Supplementary Figure 120. ^{1}H NMR spectrum in $CDCl_3$, 400 MHz, of **A12**.

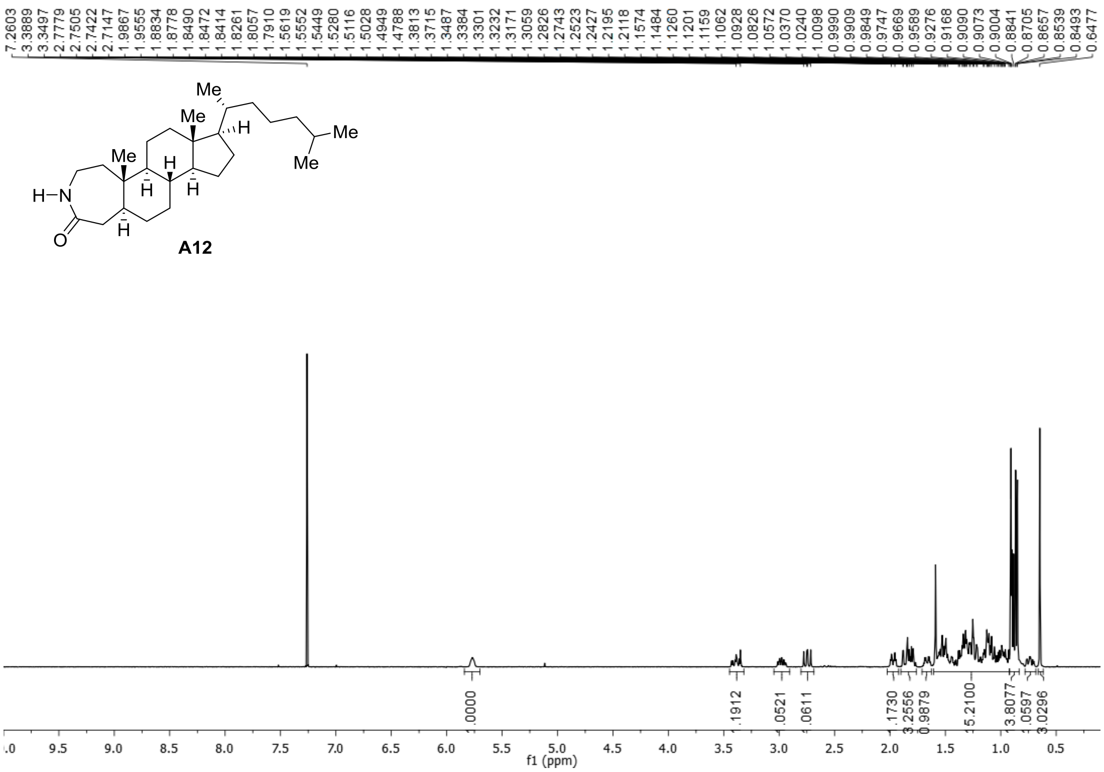

Supplementary Figure 121. ^{13}C NMR (APT) spectrum in $CDCl_3$, 101 MHz, of **A12**.

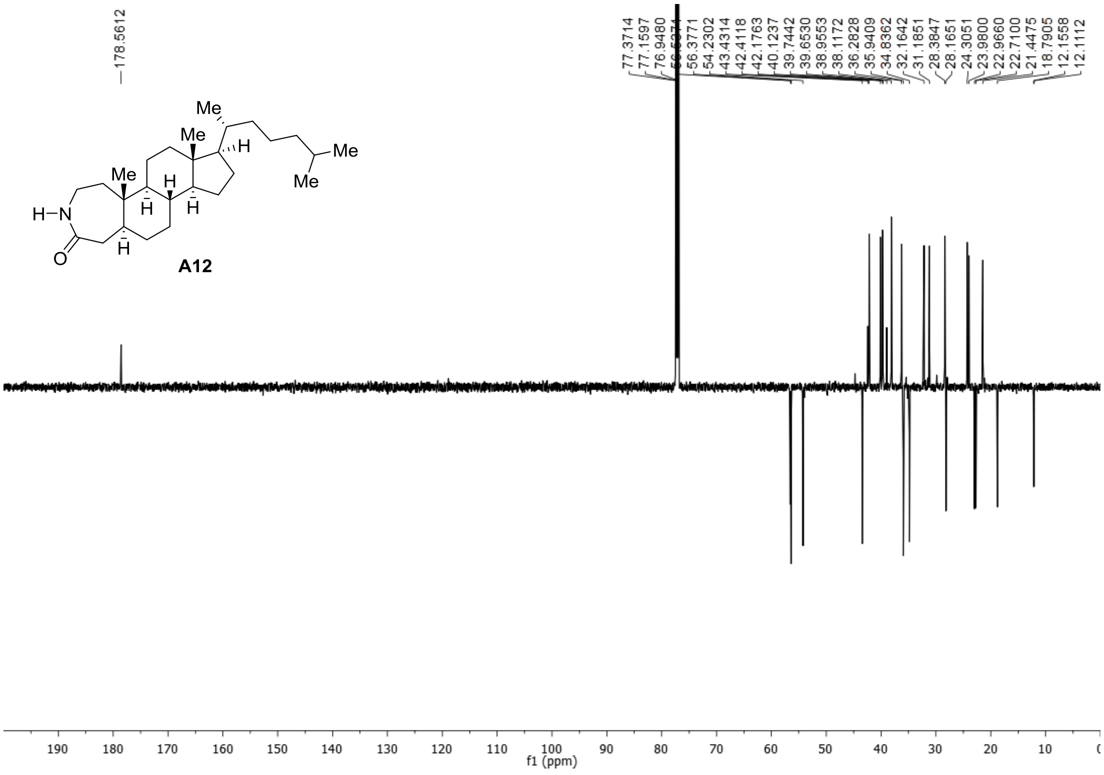

Supplementary Figure 122. ^{1}H NMR spectrum in $CDCl_3$, 400 MHz, of **B2**.

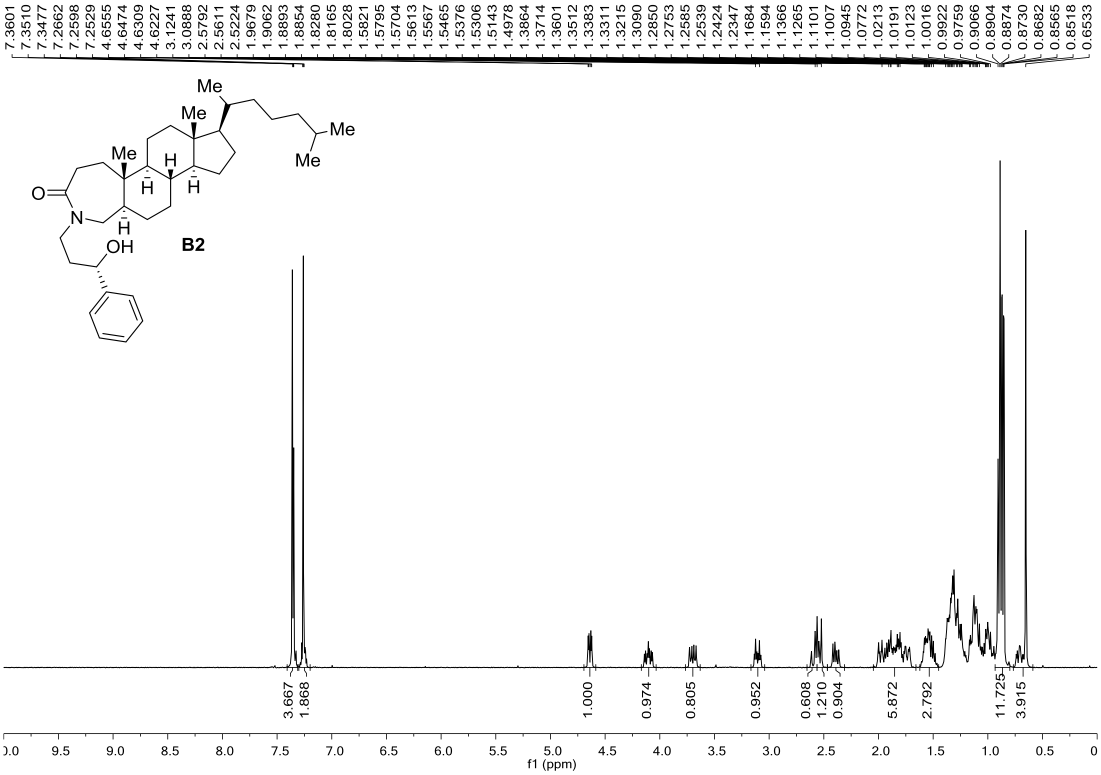

Supplementary Figure 123. ^{13}C NMR spectrum in $CDCl_3$, 101 MHz, of **B2**.

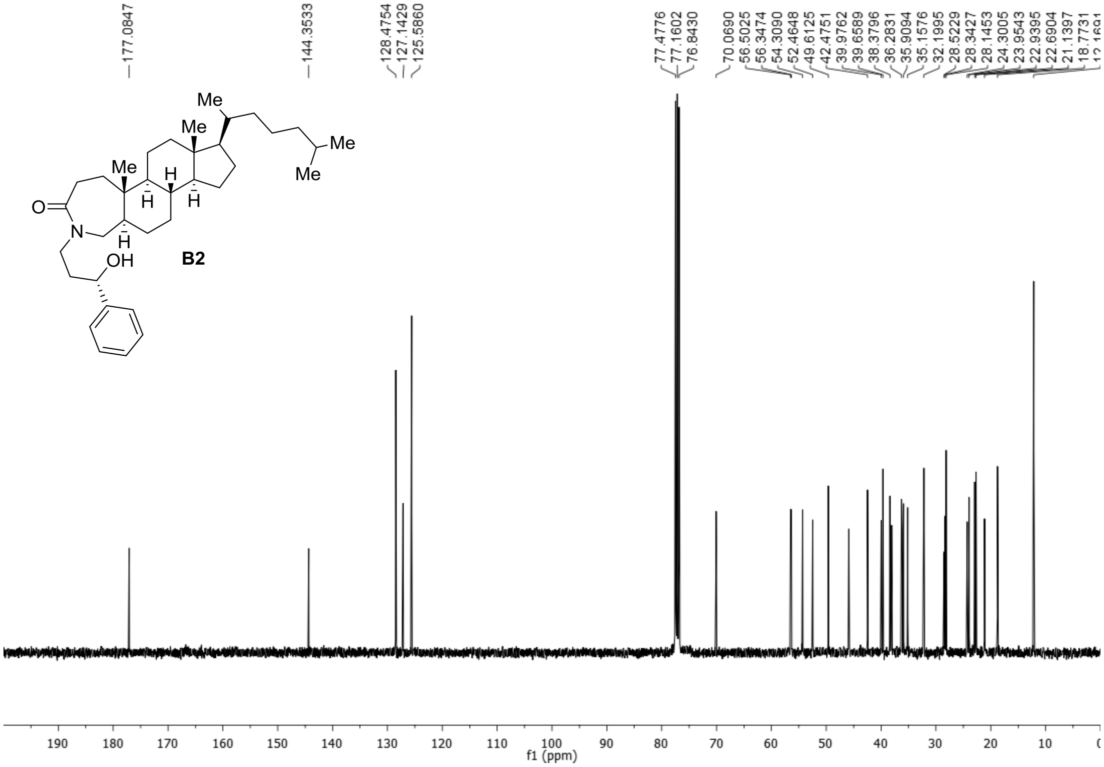

Supplementary Figure 124. ^{13}C NMR (APT) spectrum in $CDCl_3$, 101 MHz, of **B2**.

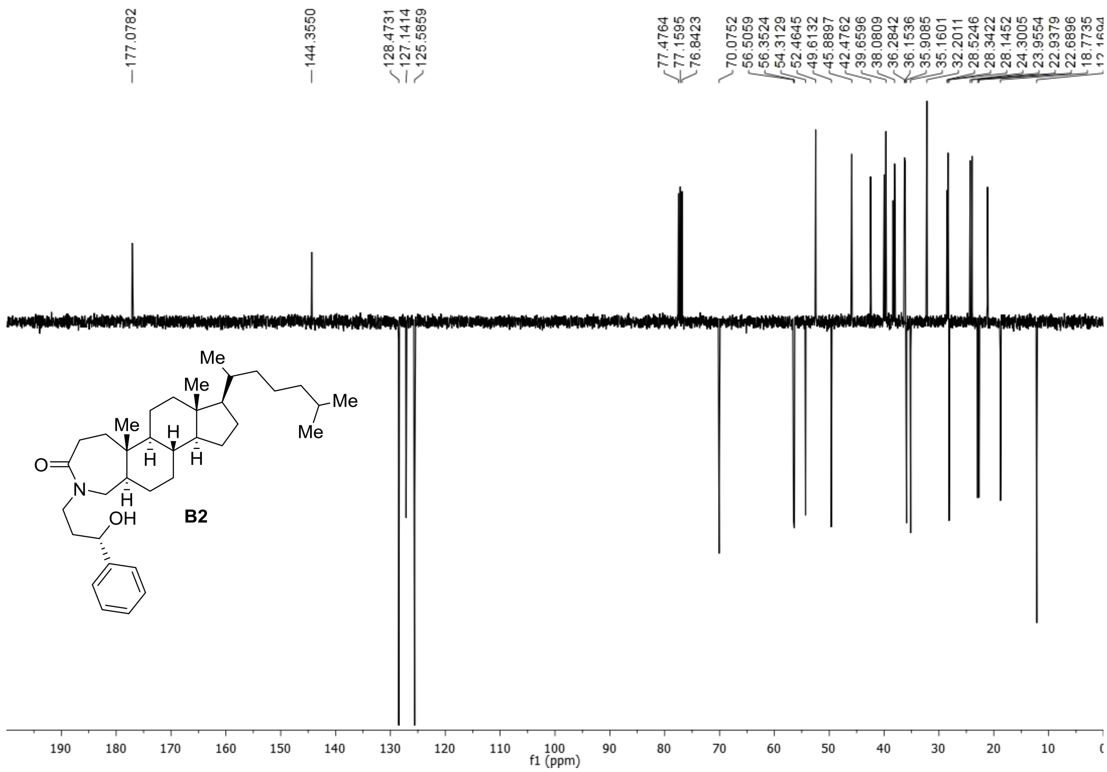

Supplementary Figure 125. HSQC spectrum of **B2**.

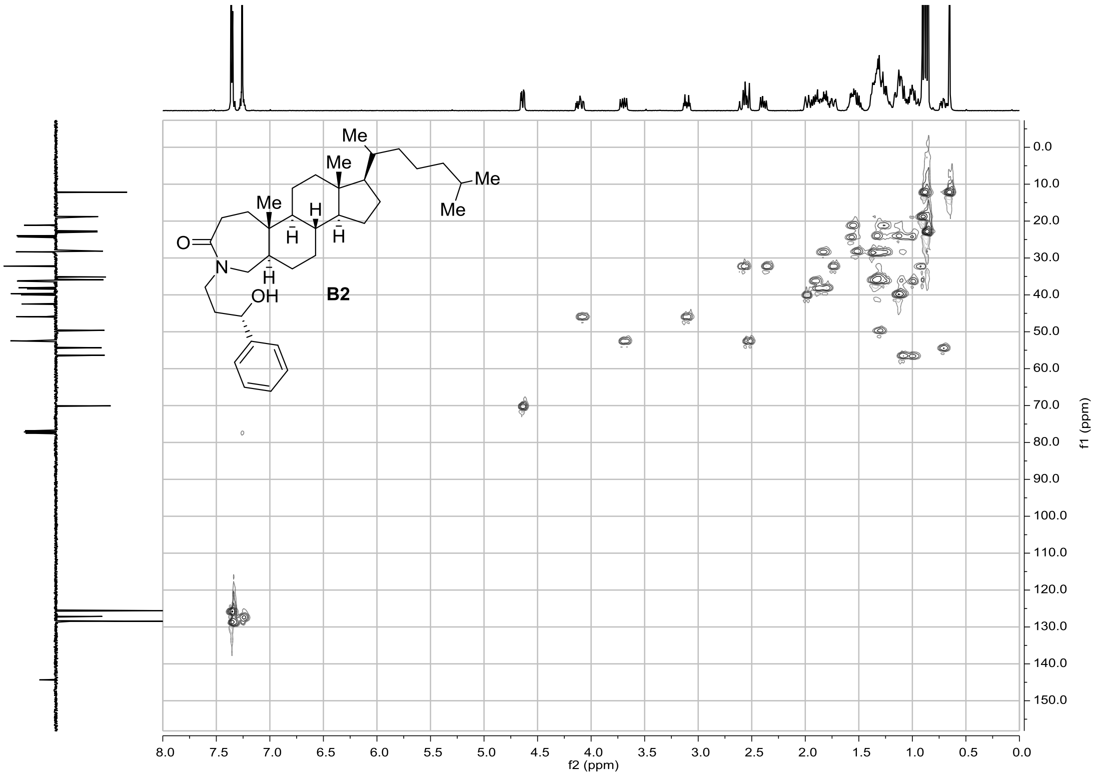

Supplementary Figure 126. ^{1}H NMR spectrum in $CDCl_3$, 400 MHz, of **B3**.

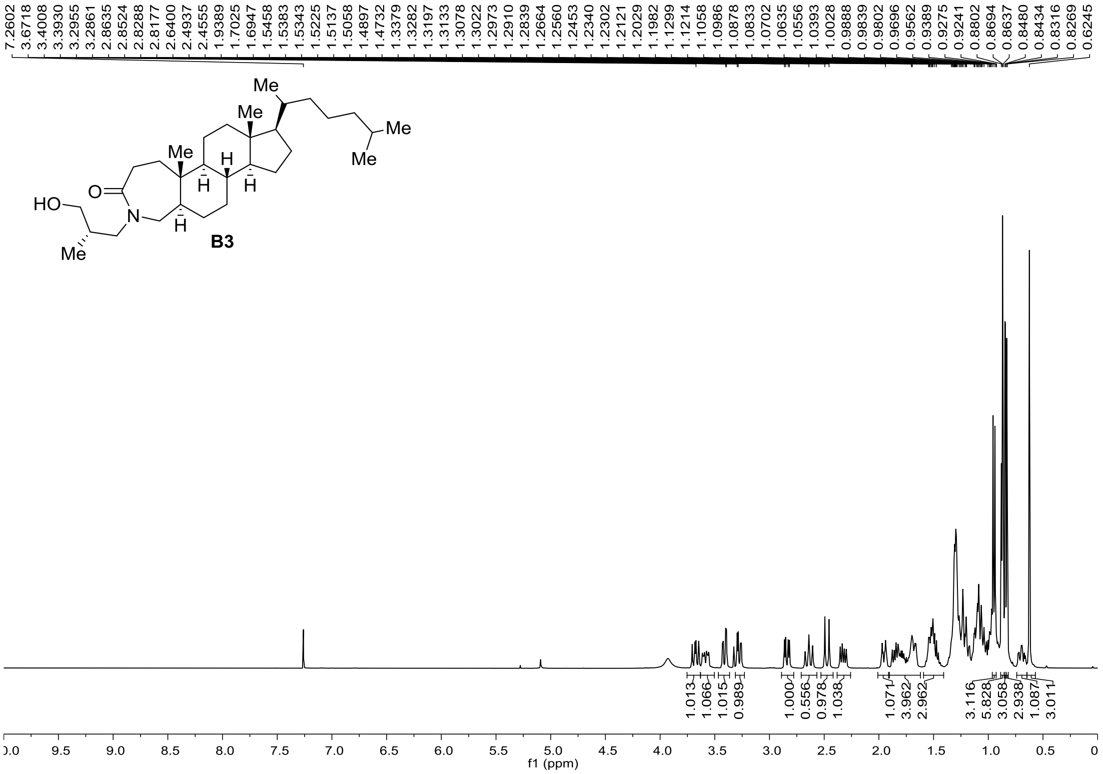

Supplementary Figure 127. ^{13}C NMR spectrum in $CDCl_3$, 101 MHz, of **B3**.

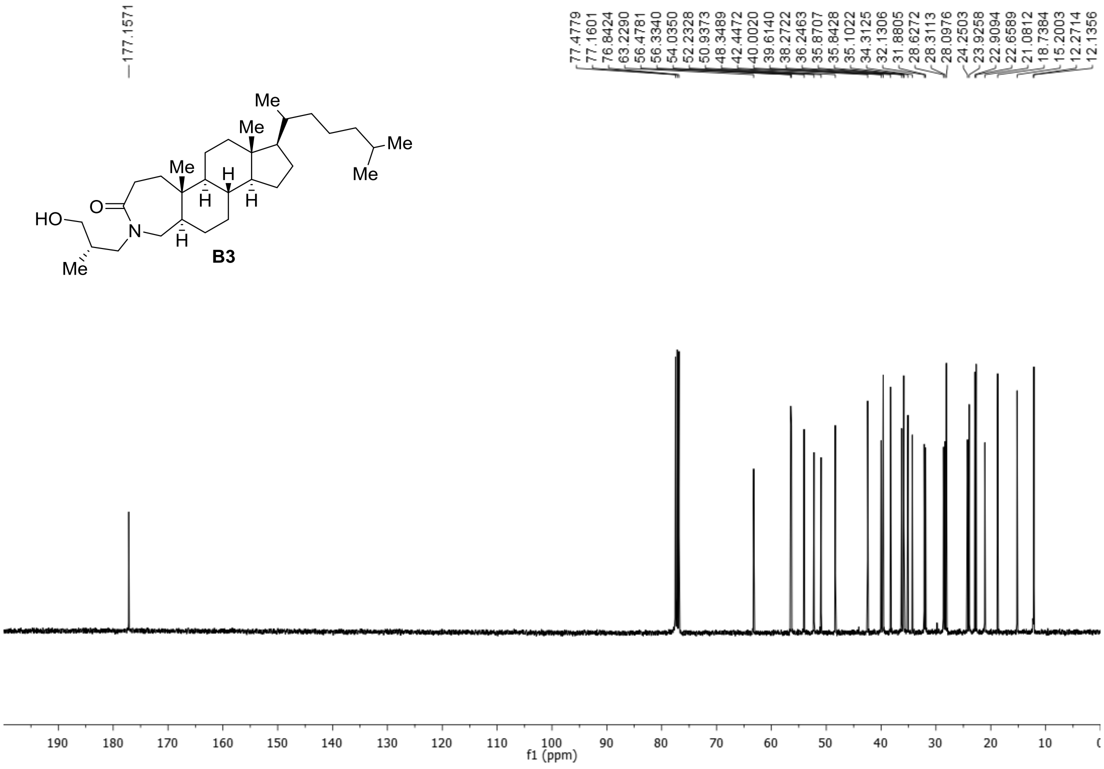

Supplementary Figure 128. ^{1}H NMR spectrum in $CDCl_3$, 400 MHz, of **B4**.

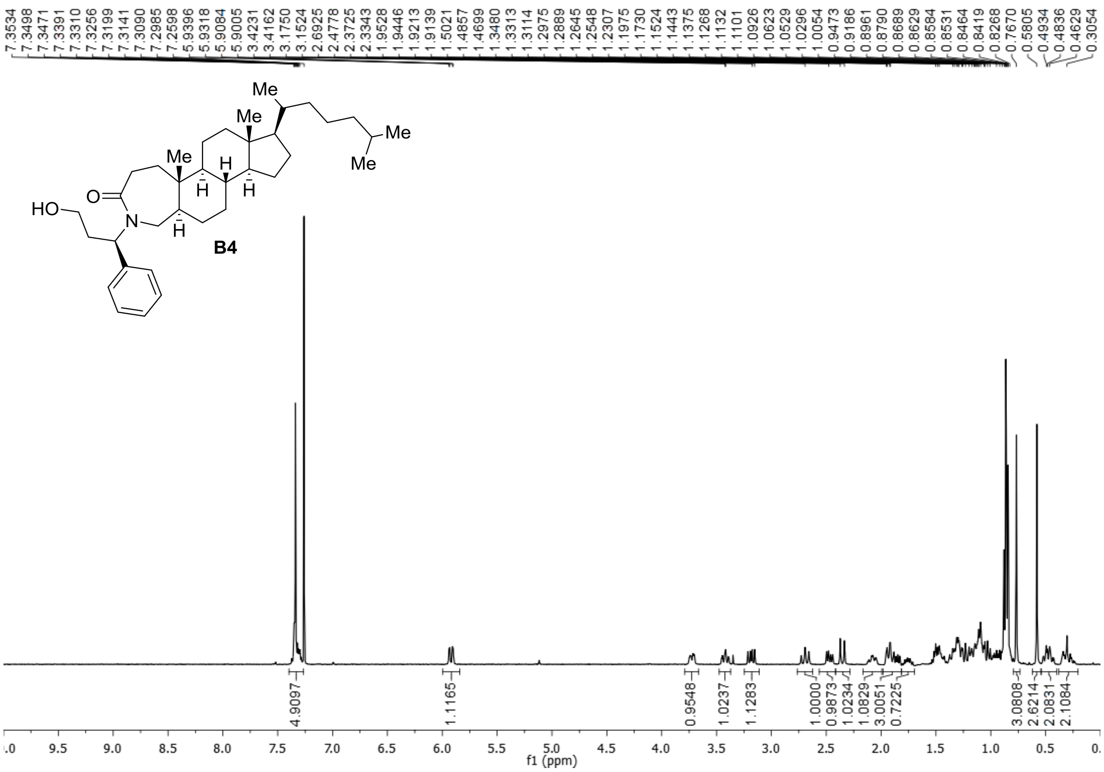

Supplementary Figure 129. ^{13}C NMR spectrum in $CDCl_3$, 126 MHz, of **B4**.

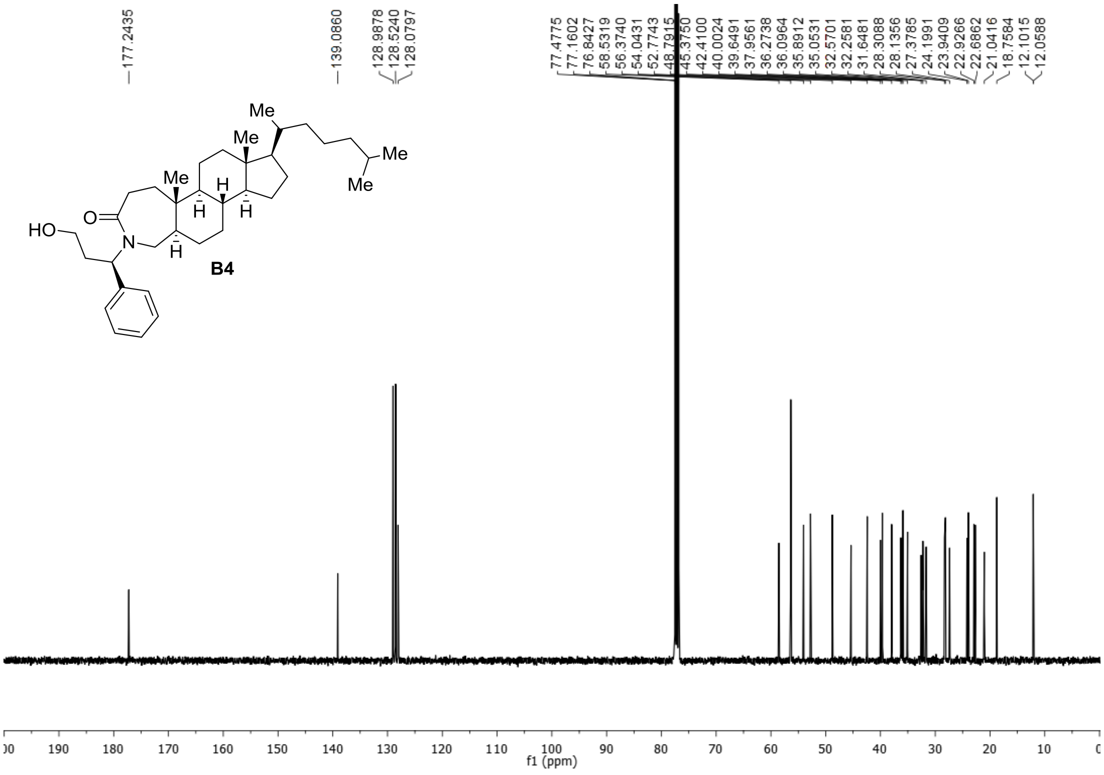

Supplementary Figure 130. ^{13}C NMR (APT) spectrum in $CDCl_3$, 126 MHz, of **B4**.

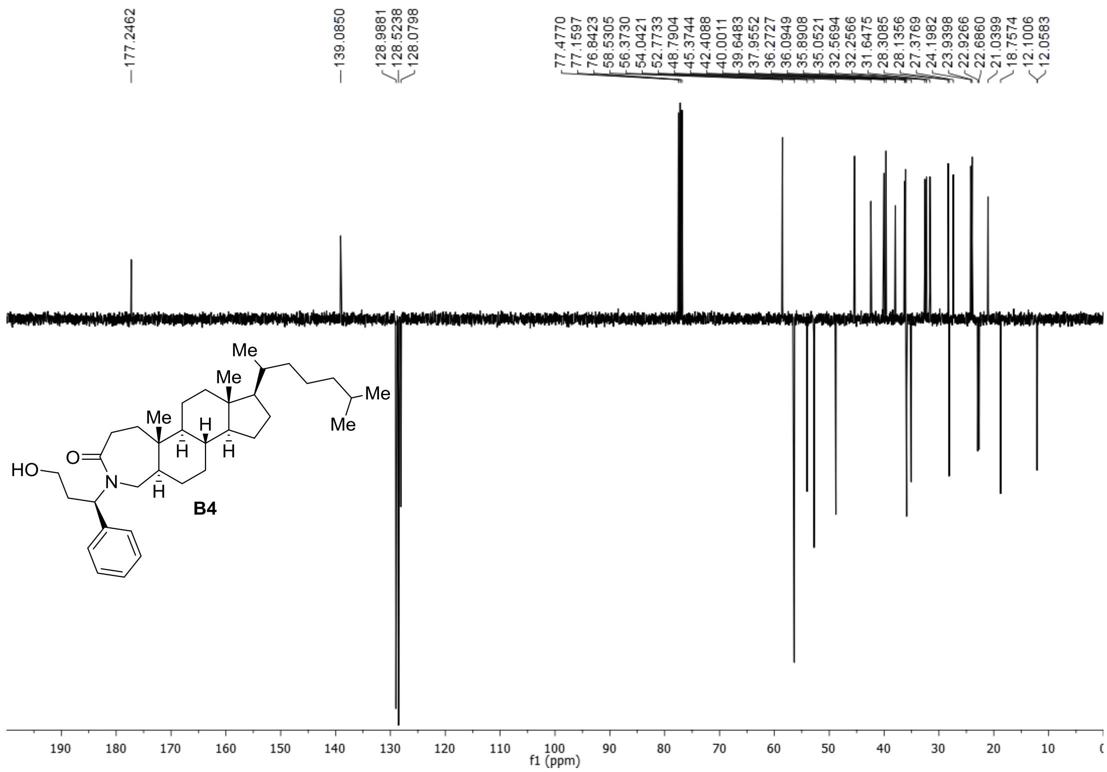

Supplementary Figure 131. HSQC spectrum of **B4**.

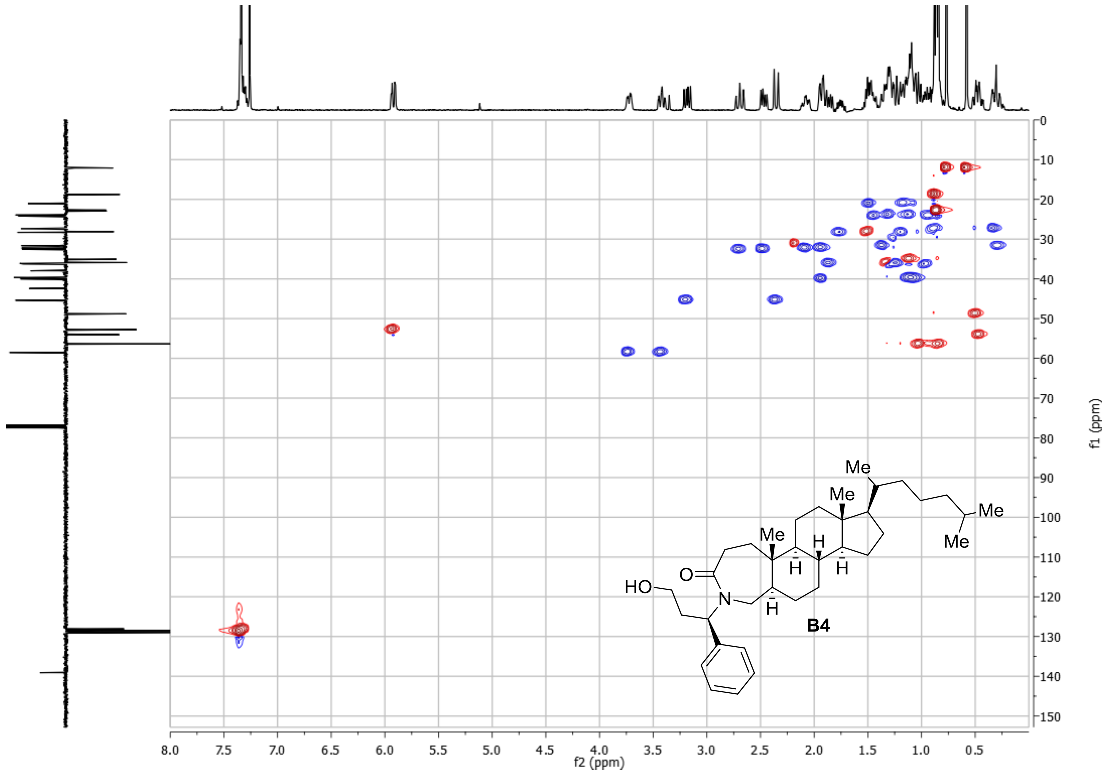

Supplementary Figure 132. ^{1}H NMR spectrum in $CDCl_3$, 400 MHz, of **B5**.

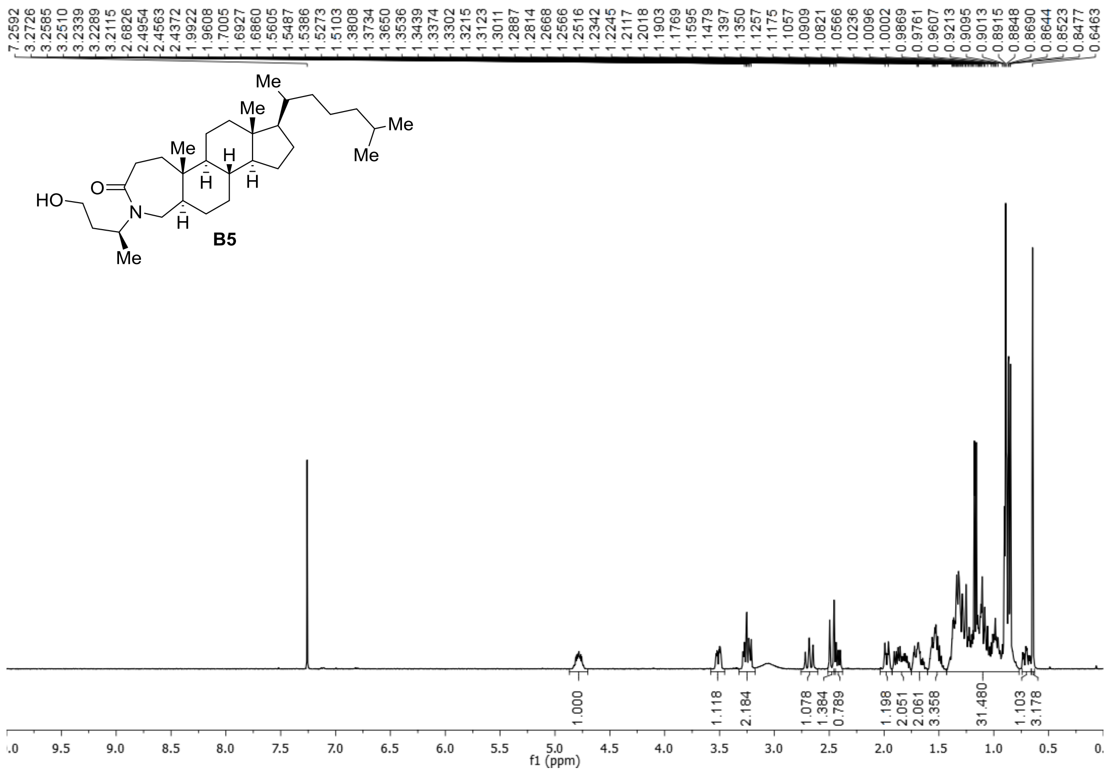

Supplementary Figure 133. ^{13}C NMR spectrum in $CDCl_3$, 101 MHz, of **B5**.

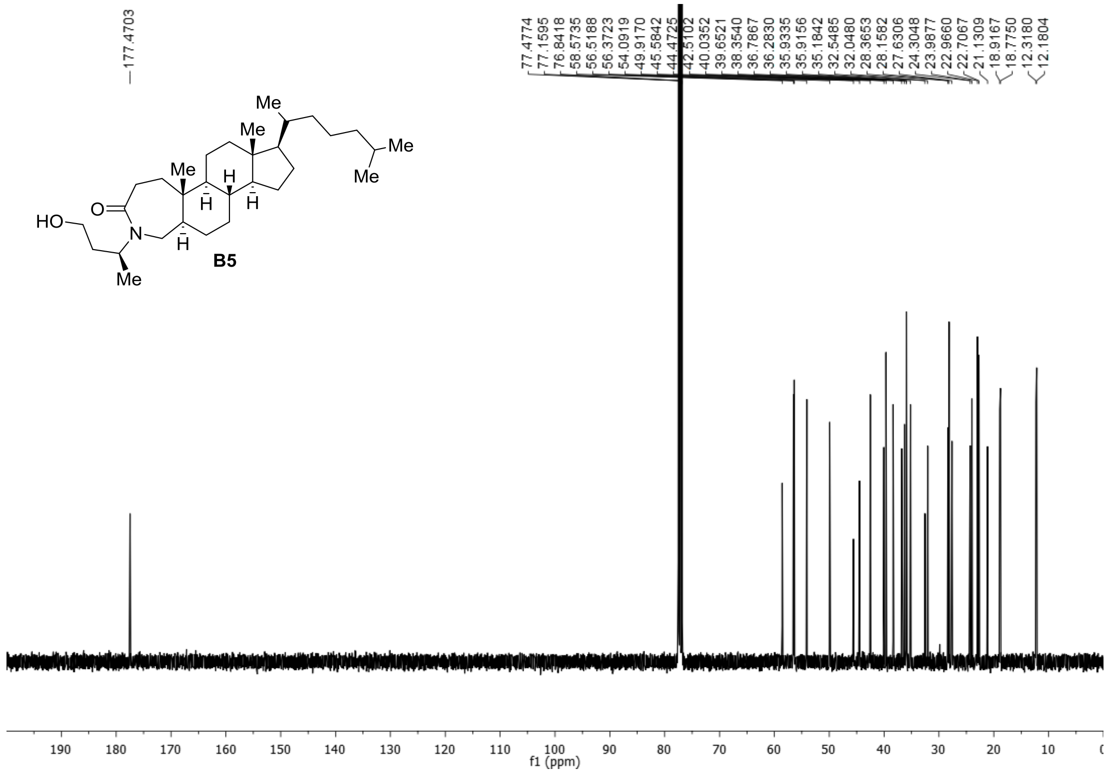

Supplementary Figure 134. ^{1}H NMR spectrum in $CDCl_3$, 400 MHz, of **B9**.

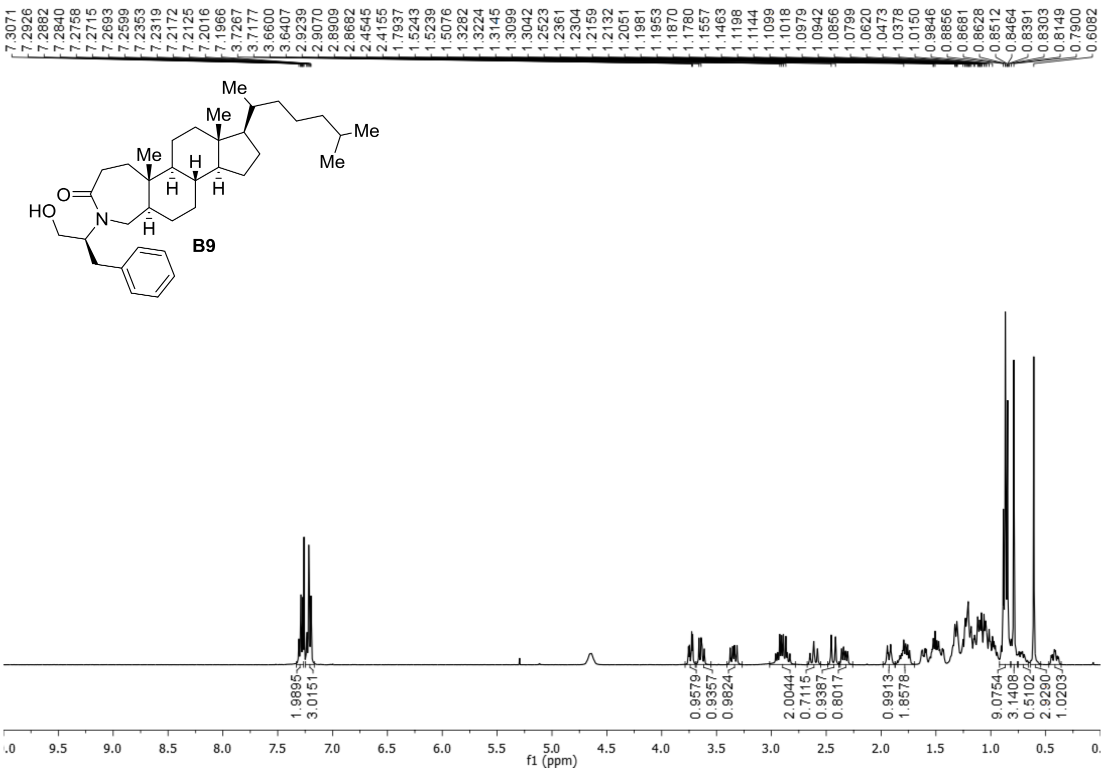

Supplementary Figure 135. ^{13}C NMR spectrum in $CDCl_3$, 126 MHz, of **B9**.

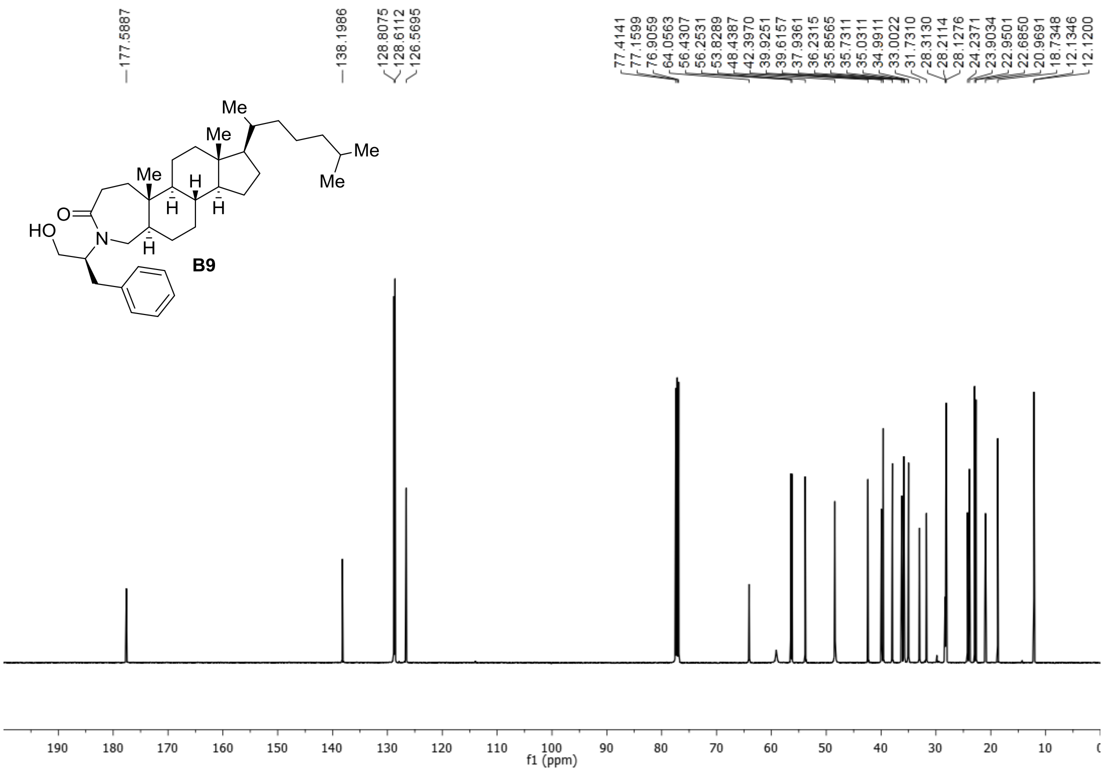

Supplementary Figure 136. ^{1}H NMR spectrum in $CDCl_3$, 400 MHz, of **B10**.

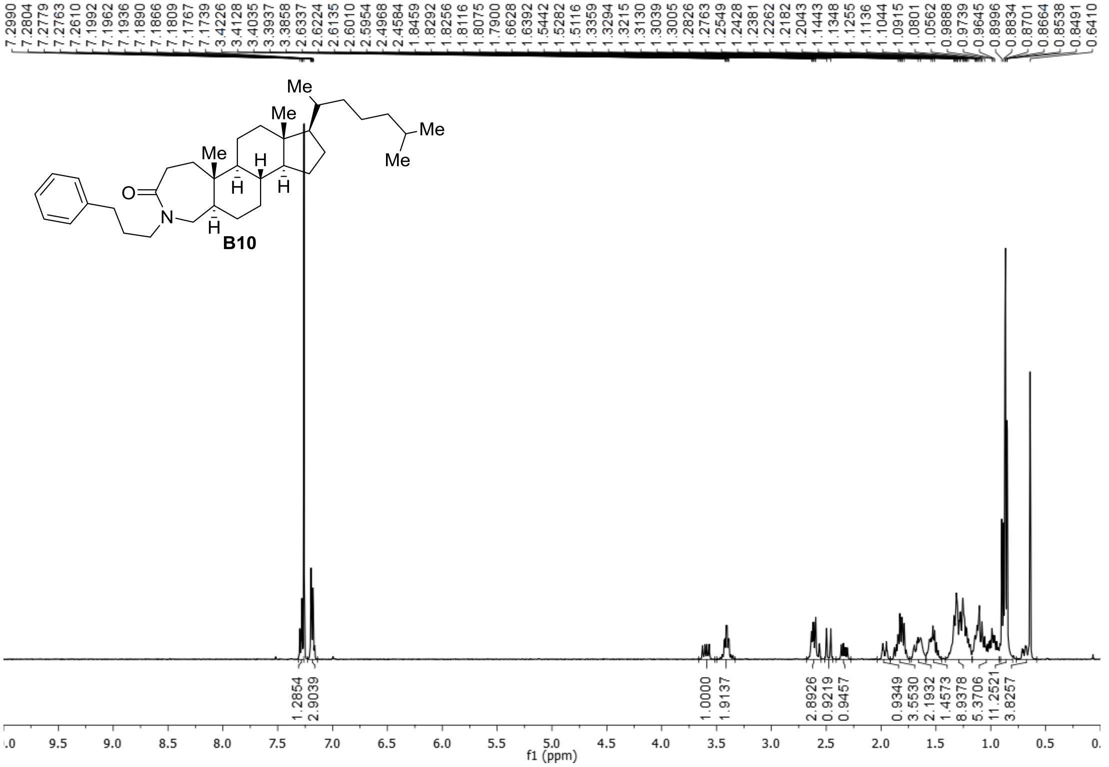

Supplementary Figure 137. ^{13}C NMR spectrum in $CDCl_3$, 101 MHz, of **B10**.

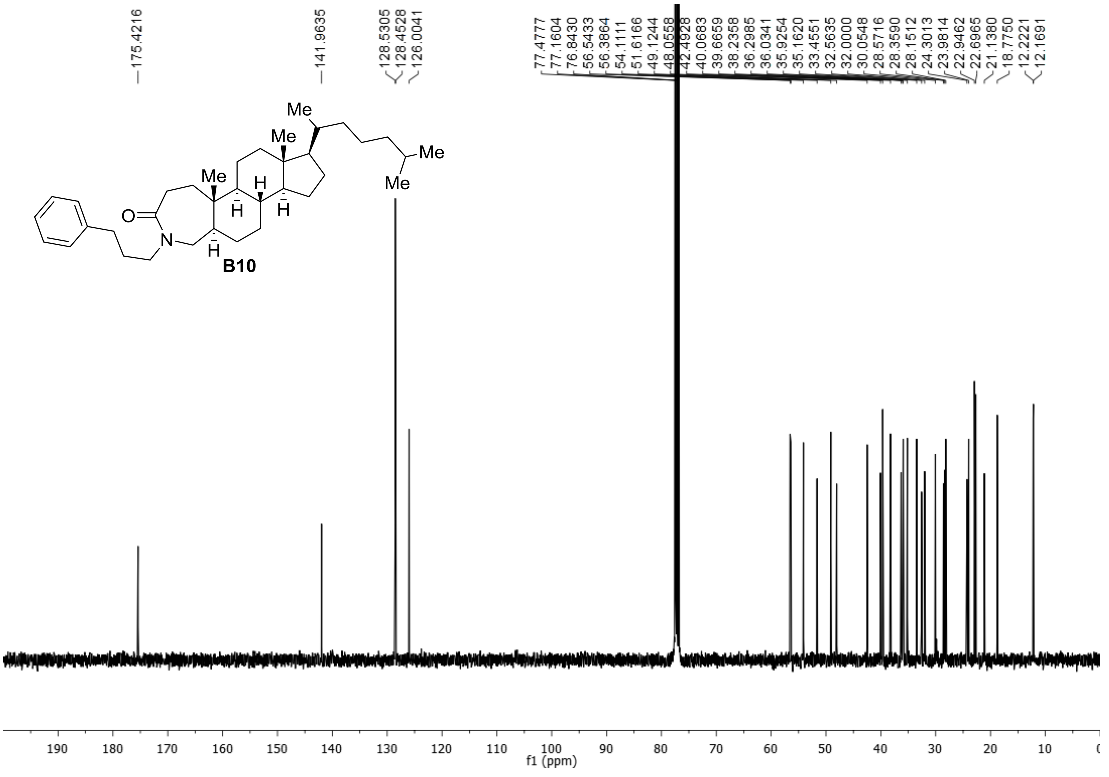

Supplementary Figure 138. ^{1}H NMR spectrum in $CDCl_3$, 500 MHz, of **B11**.

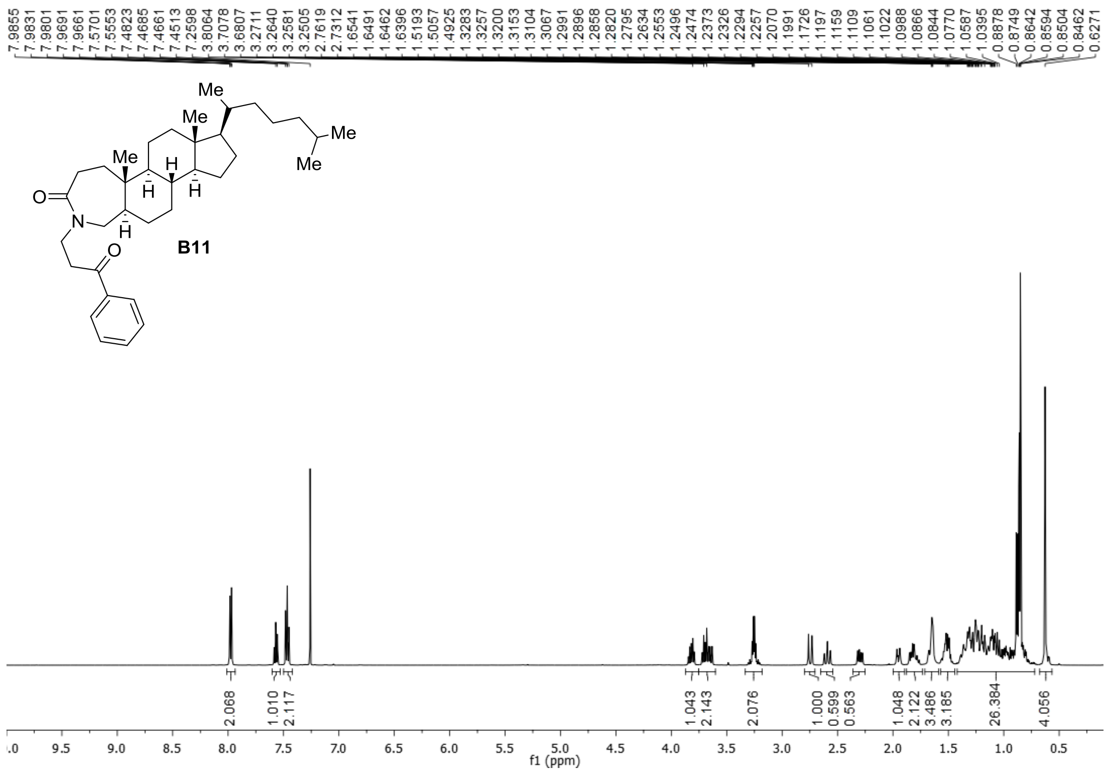

Supplementary Figure 139. ^{13}C NMR spectrum in $CDCl_3$, 126 MHz, of **B11**.

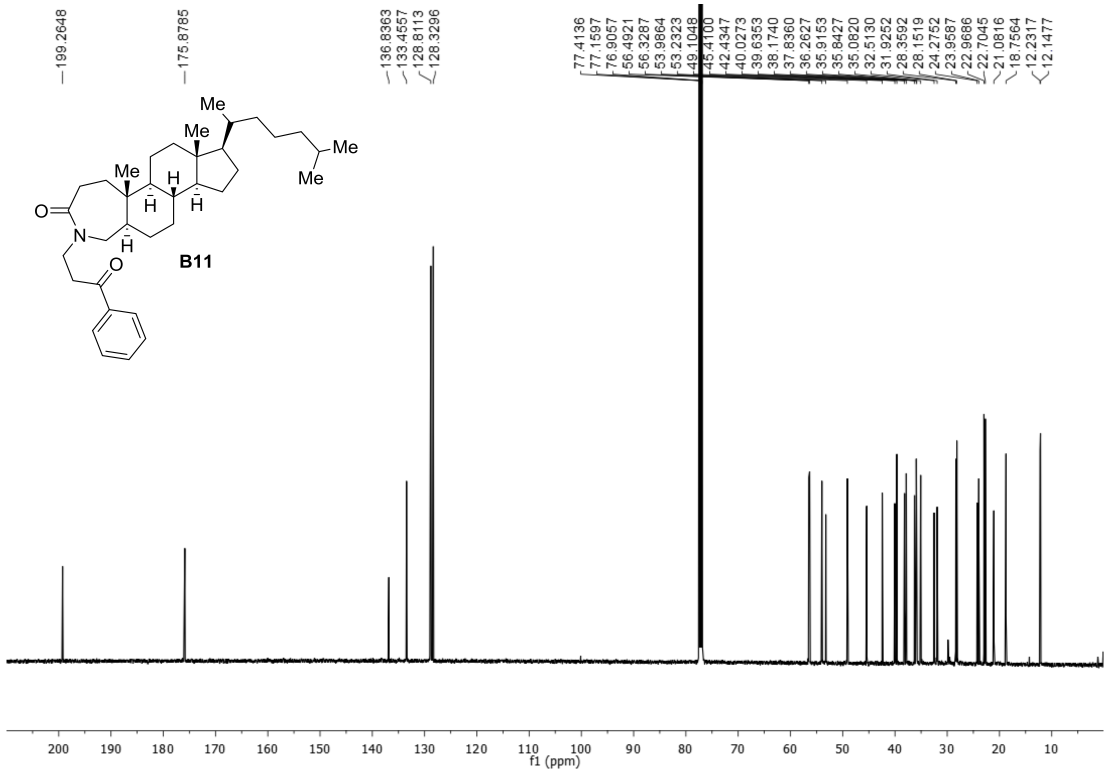

Supplementary Figure 140. ^{1}H NMR spectrum in $CDCl_3$, 400 MHz, of **B12**.

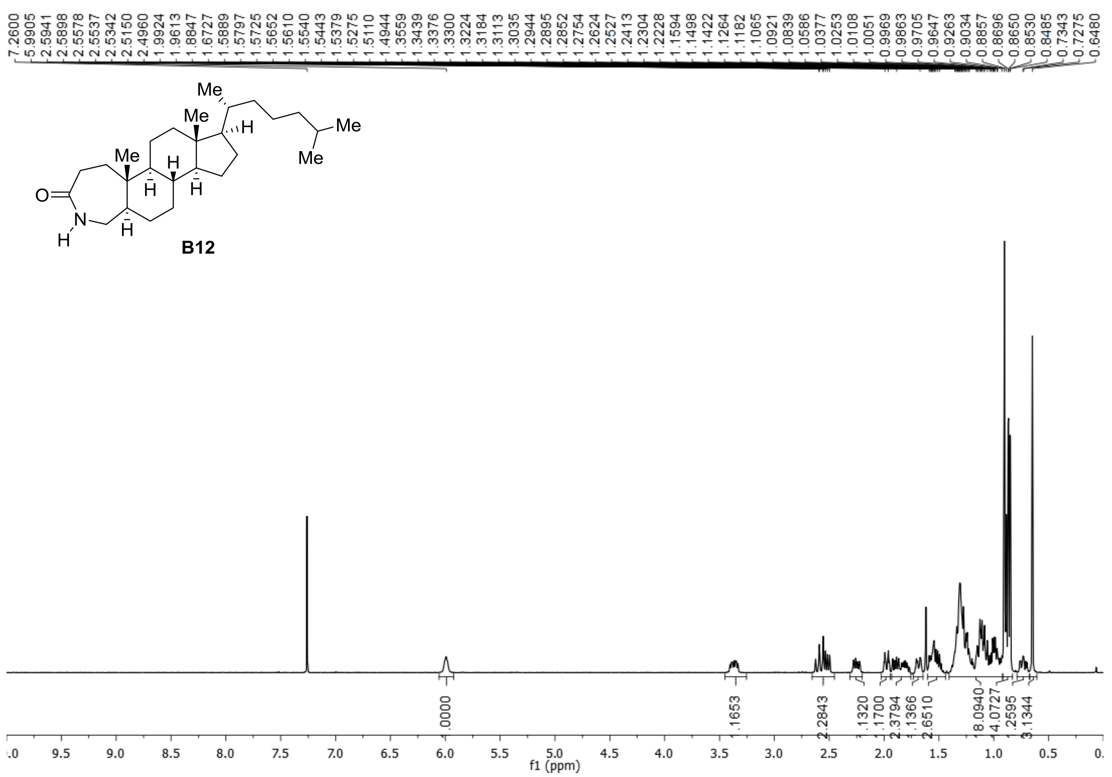

Supplementary Figure 141. ^{13}C NMR (APT) spectrum in $CDCl_3$, 101 MHz, of **B12**.

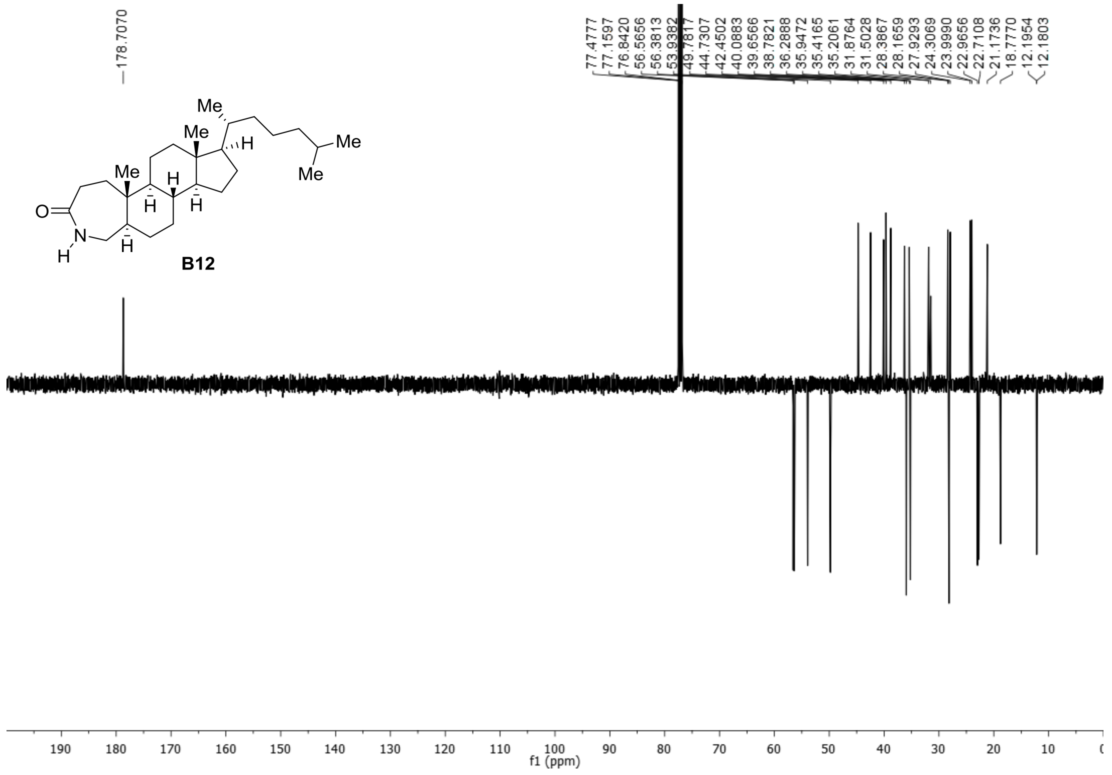

Supplementary Figure 142. ^{1}H NMR spectrum in $CDCl_3$, 400 MHz, of **C1** and **D1**.

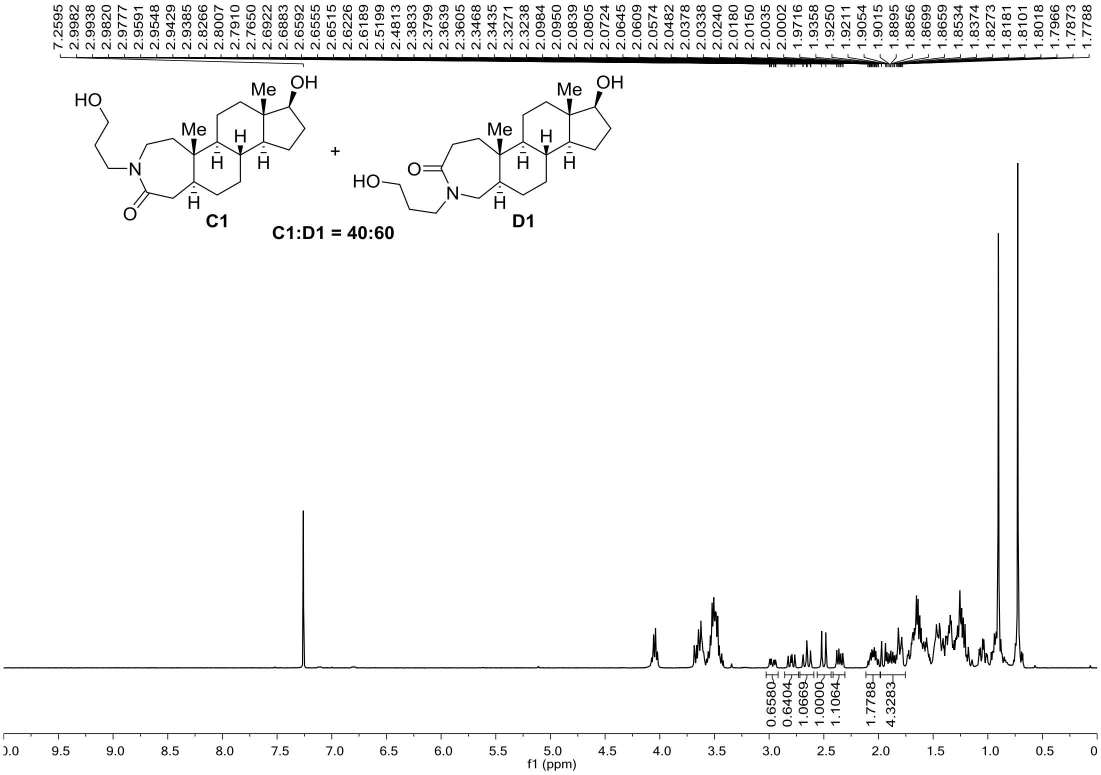

Supplementary Figure 143. ^{13}C NMR spectrum in $CDCl_3$, 126 MHz, of **C1** and **D1**.

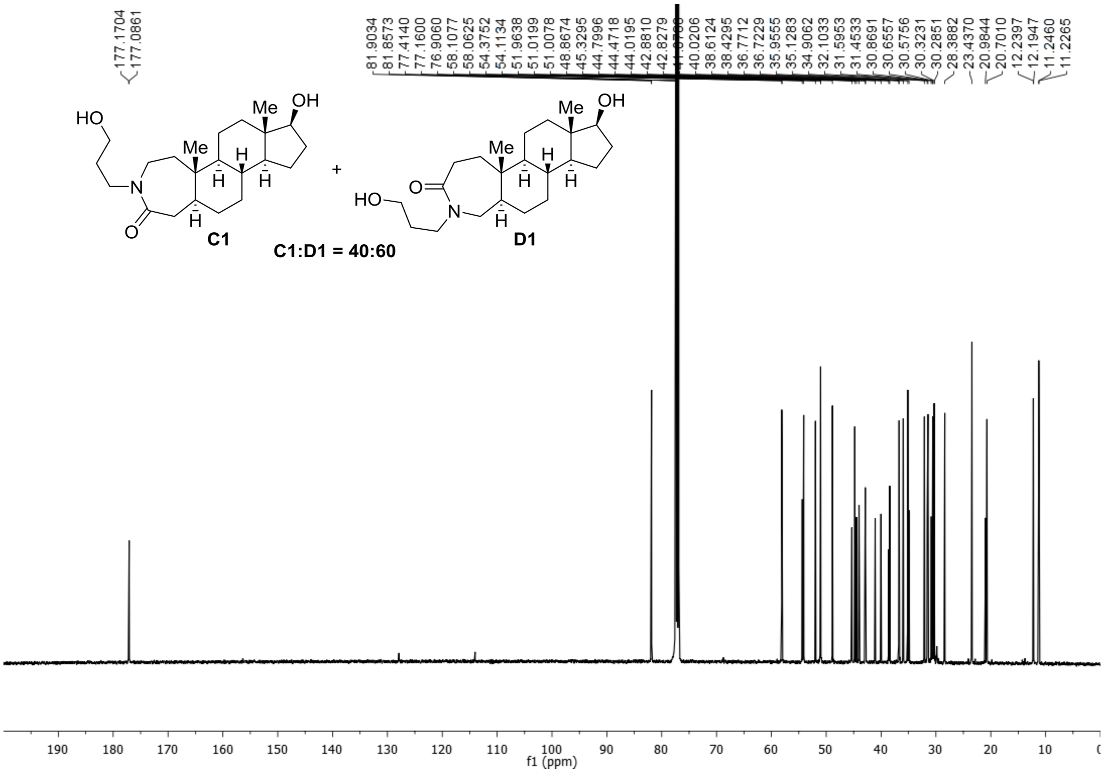

Supplementary Figure 144. ^{1}H NMR spectrum in $CDCl_3$, 400 MHz, of **C2**.

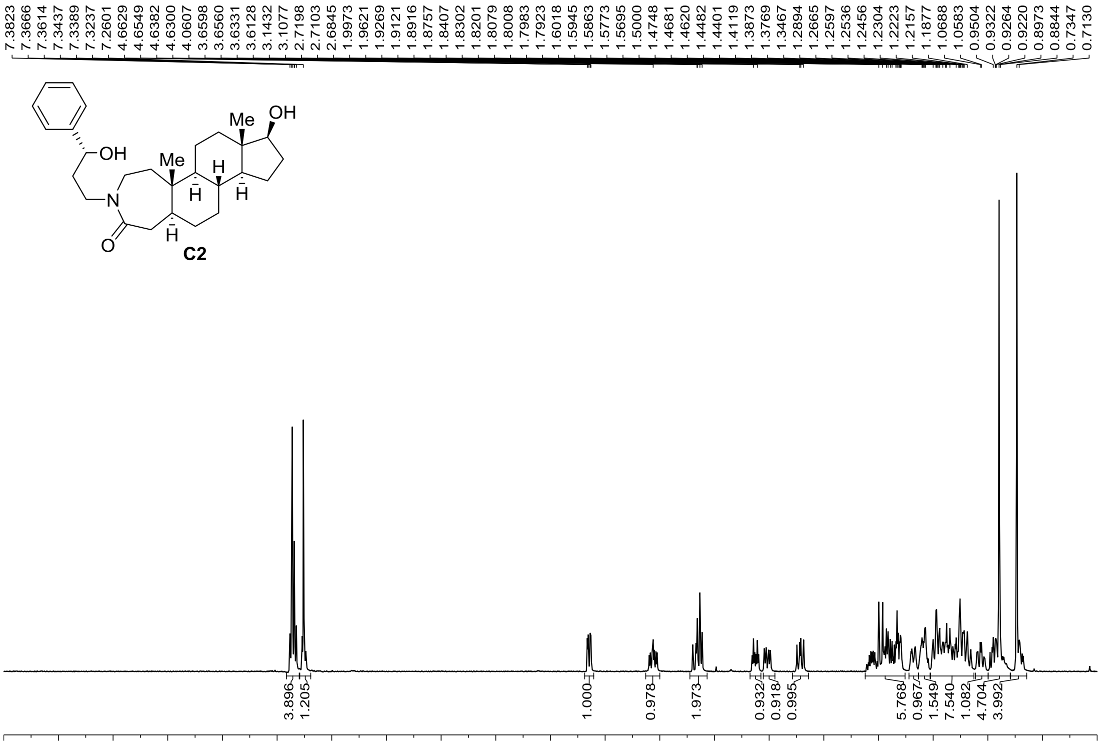

Supplementary Figure 145. ^{13}C NMR spectrum in $CDCl_3$, 126 MHz, of **C2**.

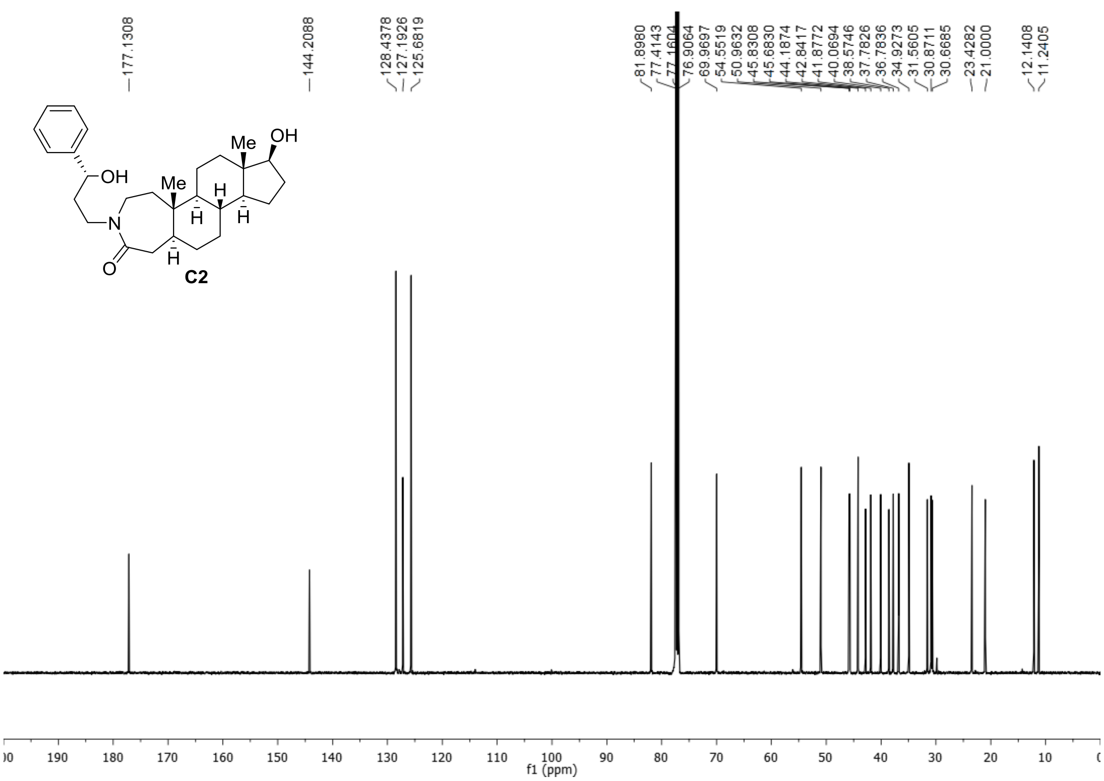

Supplementary Figure 146. ^{13}C NMR (APT) spectrum in $CDCl_3$, 126 MHz, of **C2**.

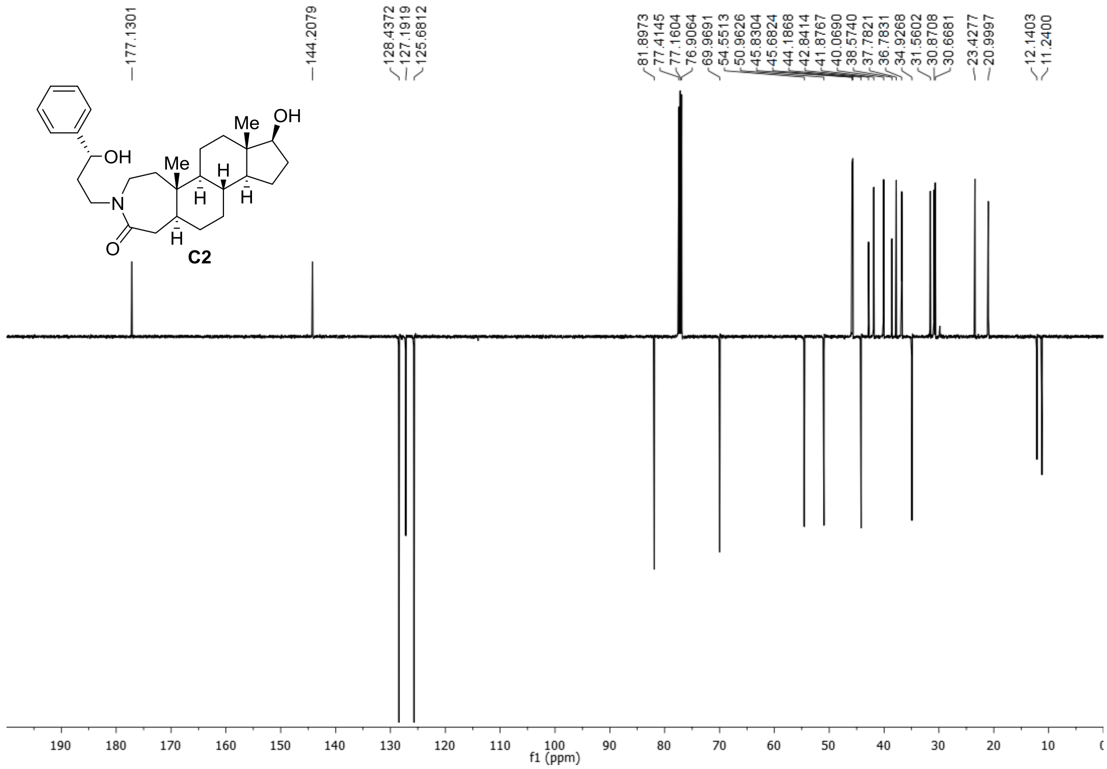

Supplementary Figure 147. 1D NOE spectrum in $CDCl_3$, 400 MHz, of **C2**.

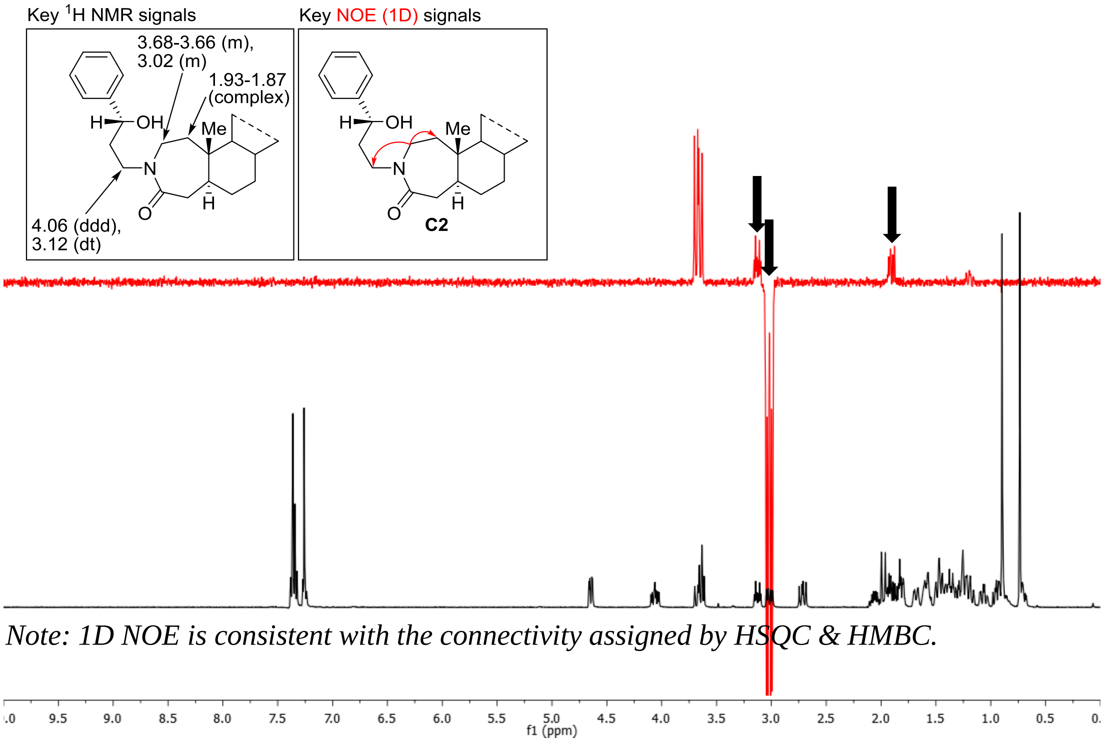

Note: 1D NOE is consistent with the connectivity assigned by HSQC & HMBC.

Supplementary Figure 148. 1D NOE spectrum in $CDCl_3$, 400 MHz, of **C2**.

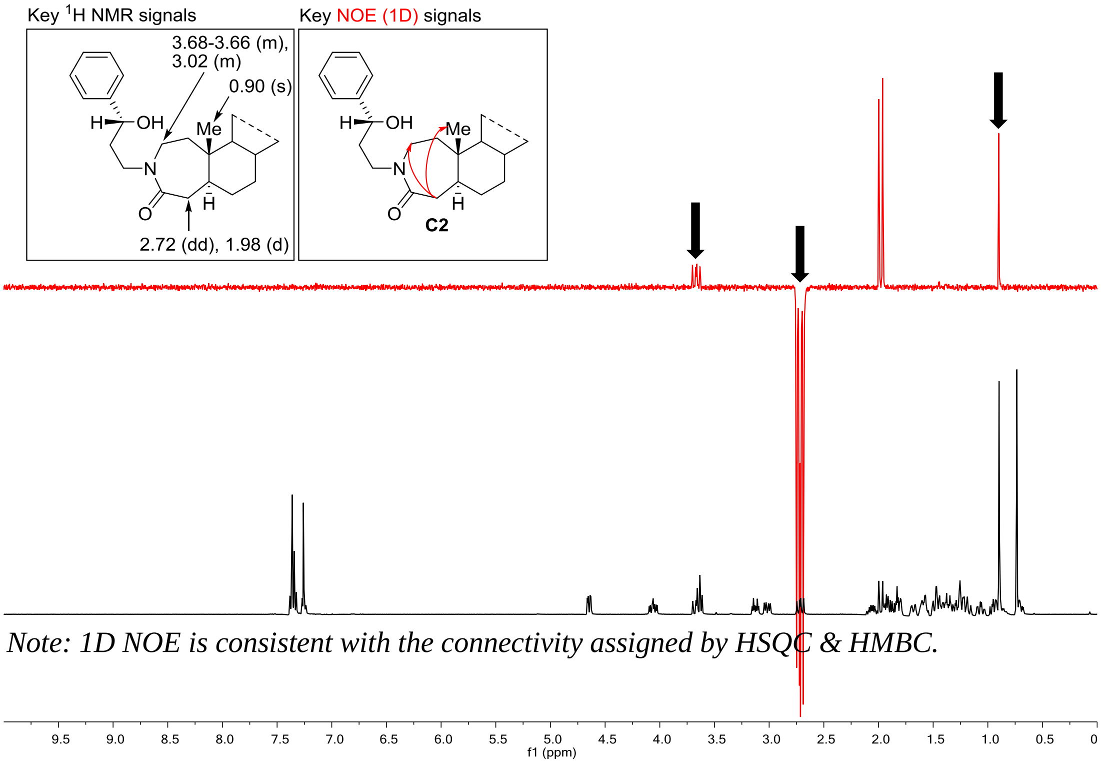

Note: 1D NOE is consistent with the connectivity assigned by HSQC & HMBC.

Supplementary Figure 149. HSQC spectrum of **C2**.

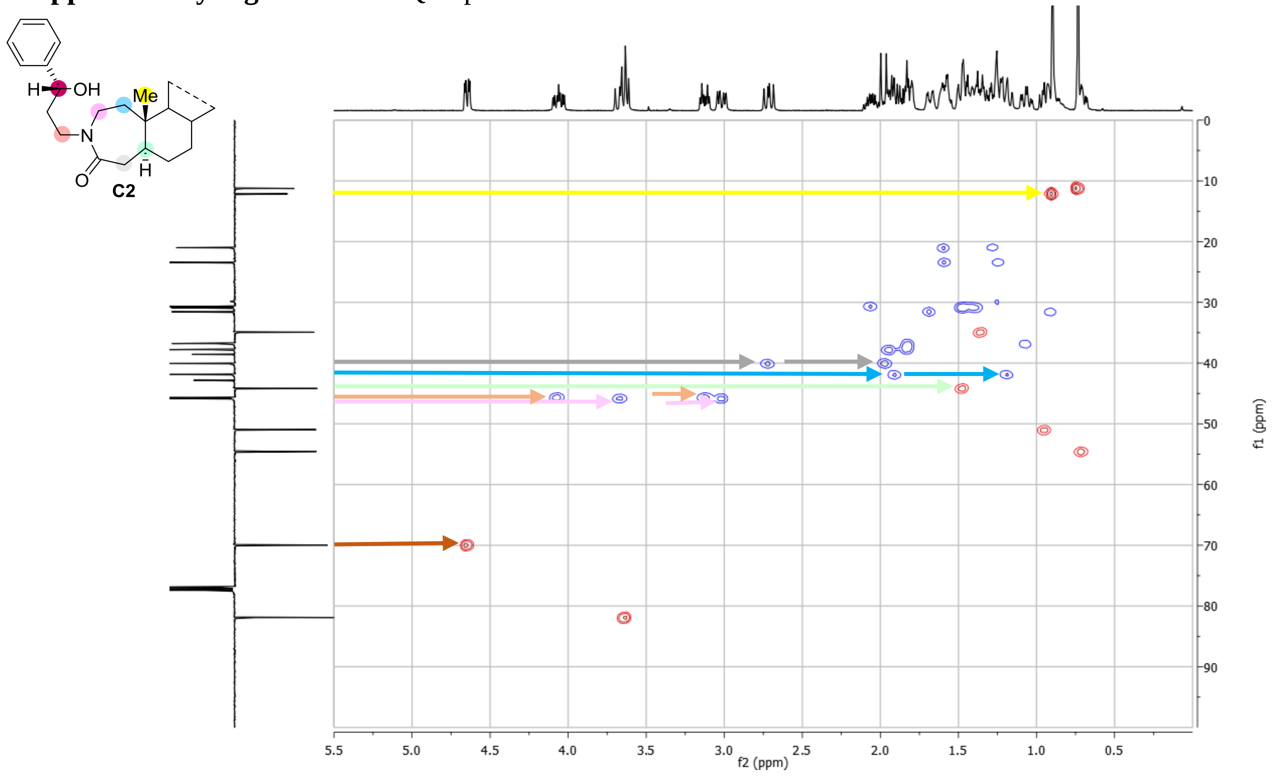

Supplementary Figure 150. HMBC spectrum of **C2**.

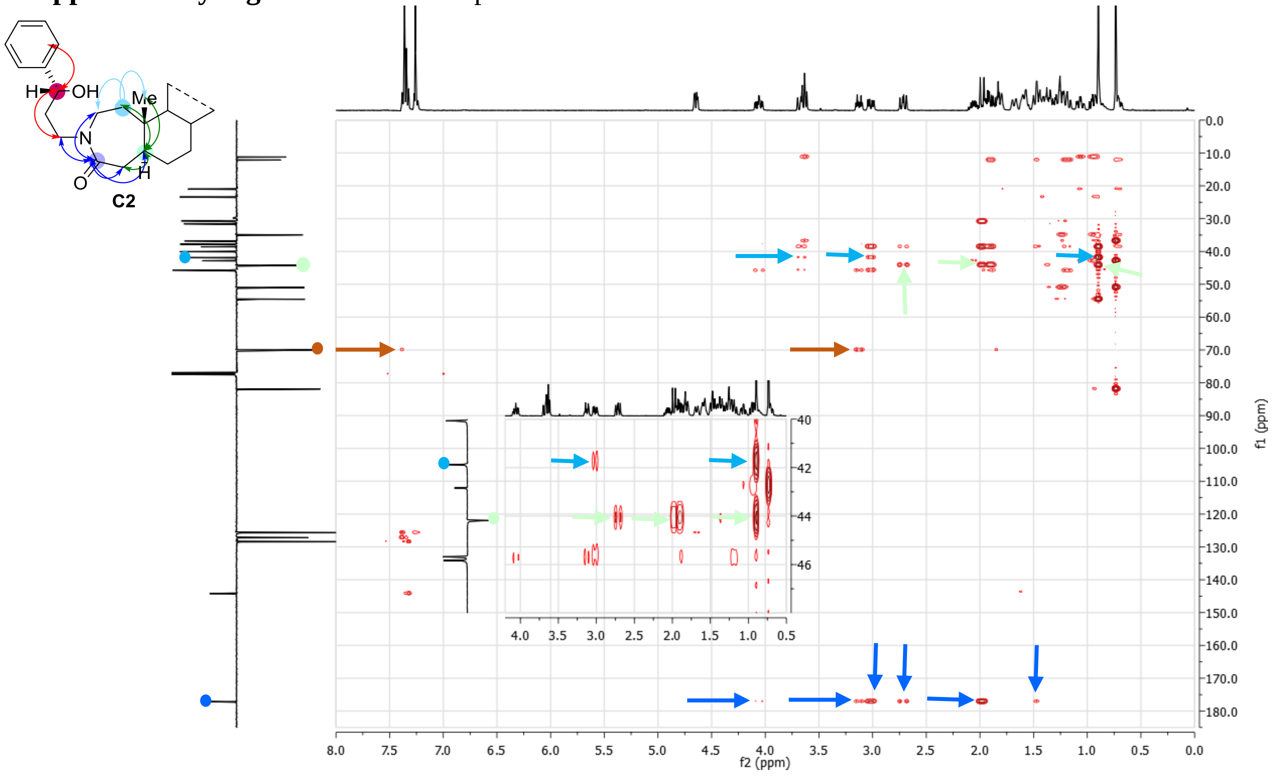

Supplementary Figure 151. ^{1}H NMR spectrum in $CDCl_3$, 400 MHz, of **C3**.

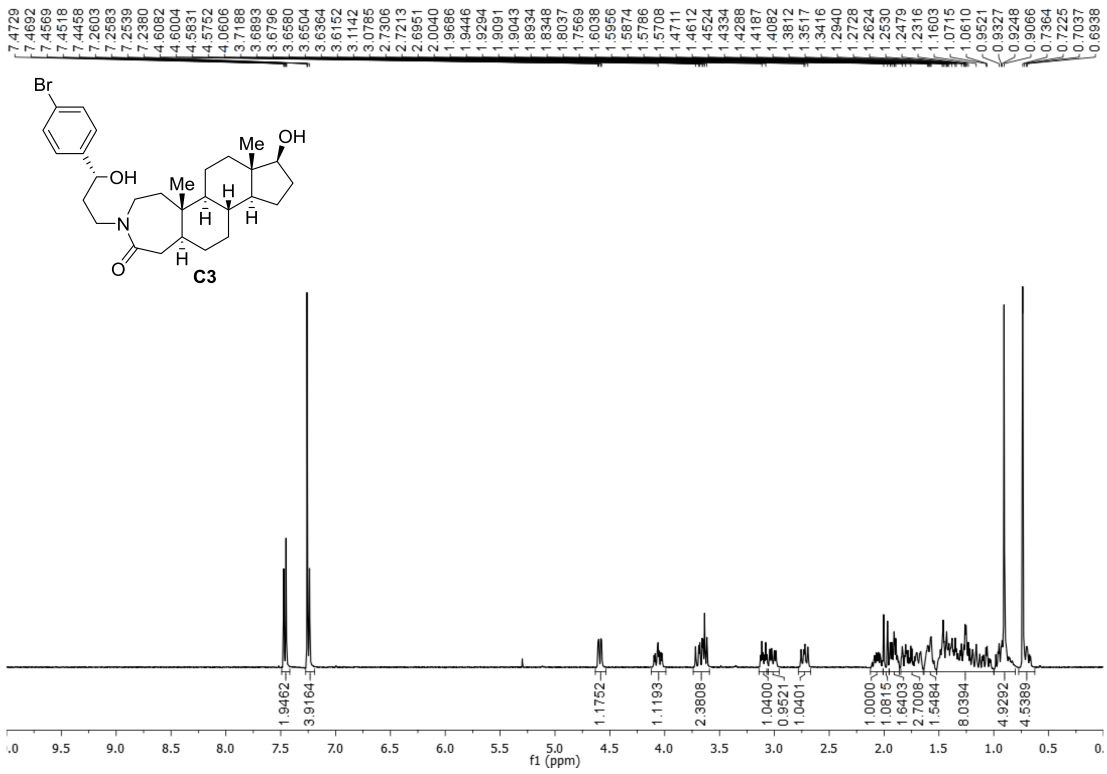

Supplementary Figure 152. ^{13}C NMR spectrum in $CDCl_3$, 126 MHz, of **C3**.

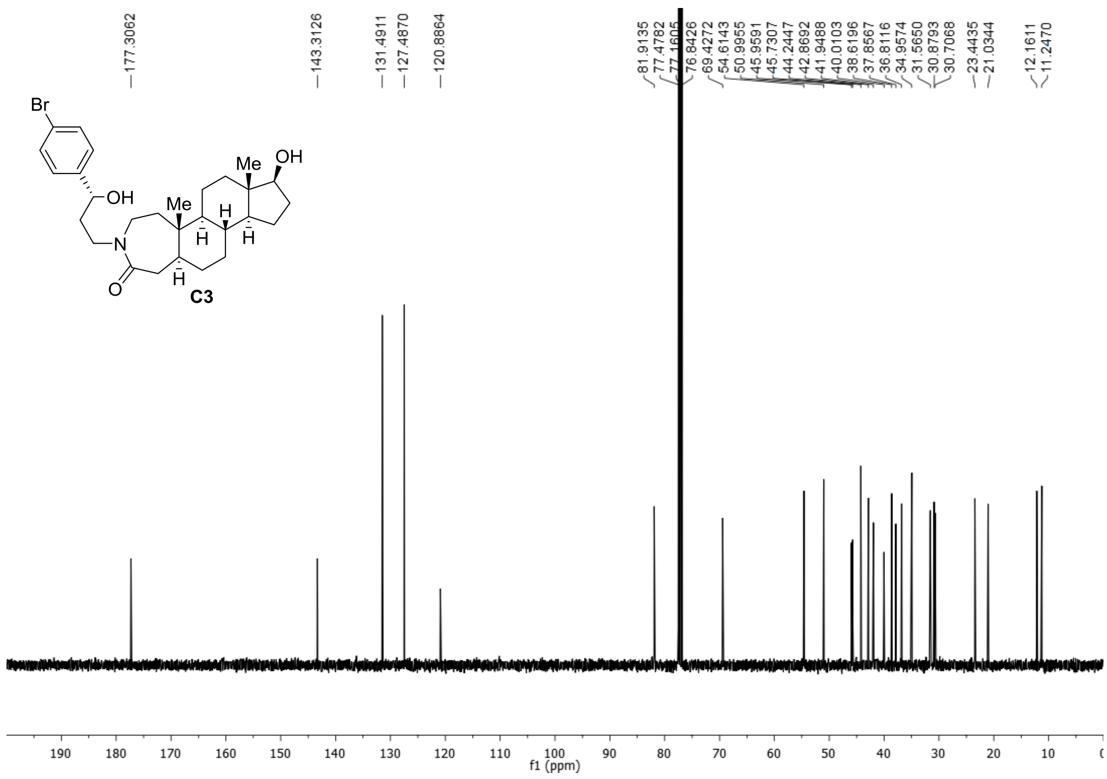

Supplementary Figure 153. ^{1}H NMR spectrum in $CDCl_3$, 400 MHz, of **C4**.

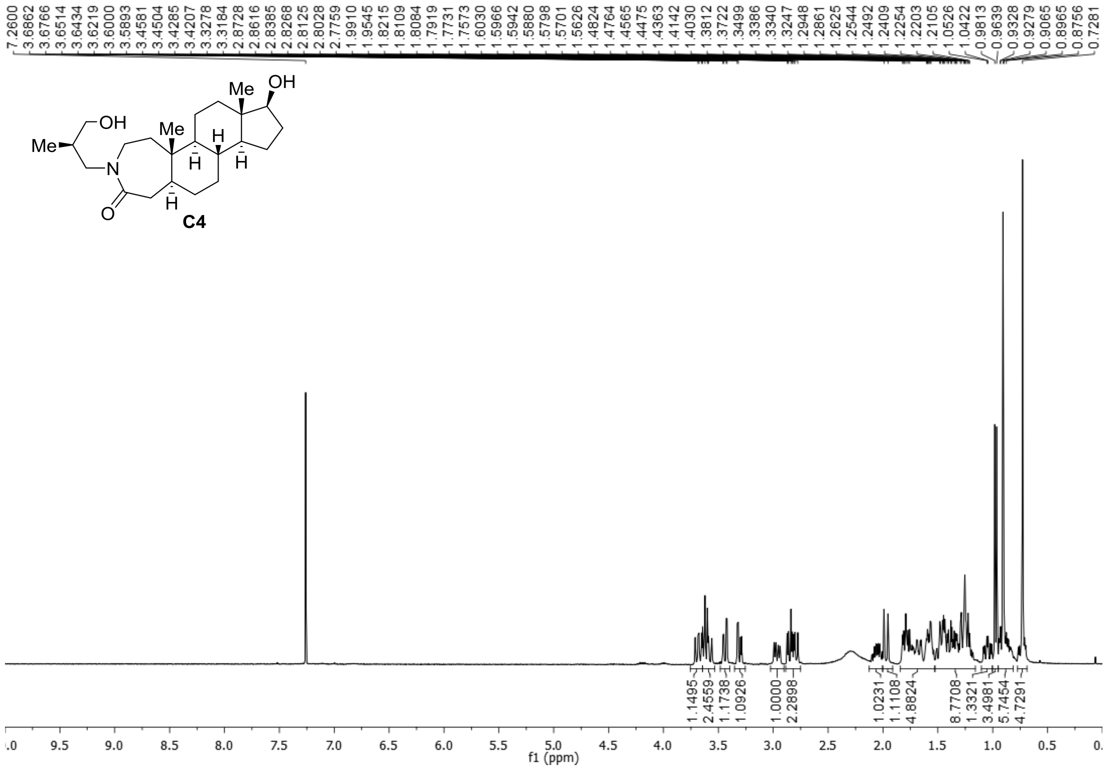

Supplementary Figure 154. ^{13}C NMR spectrum in $CDCl_3$, 151 MHz, of **C4**.

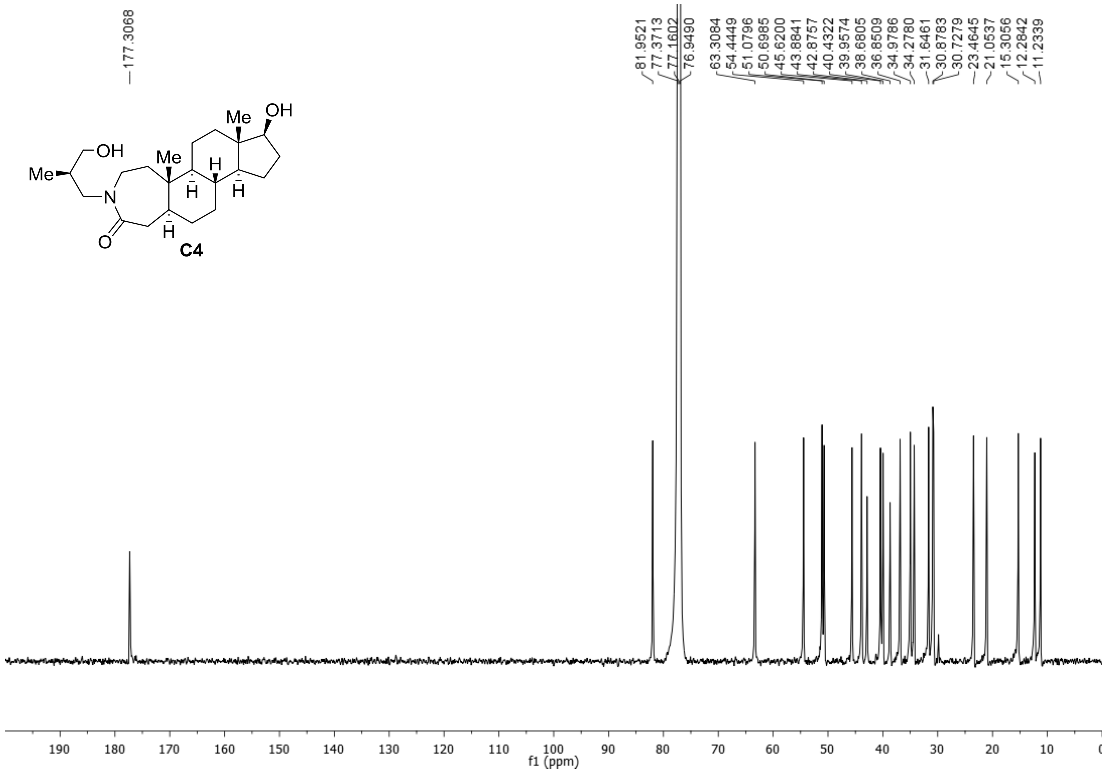

Supplementary Figure 155. ^{1}H NMR spectrum in $CDCl_3$, 400 MHz, of **C5**.

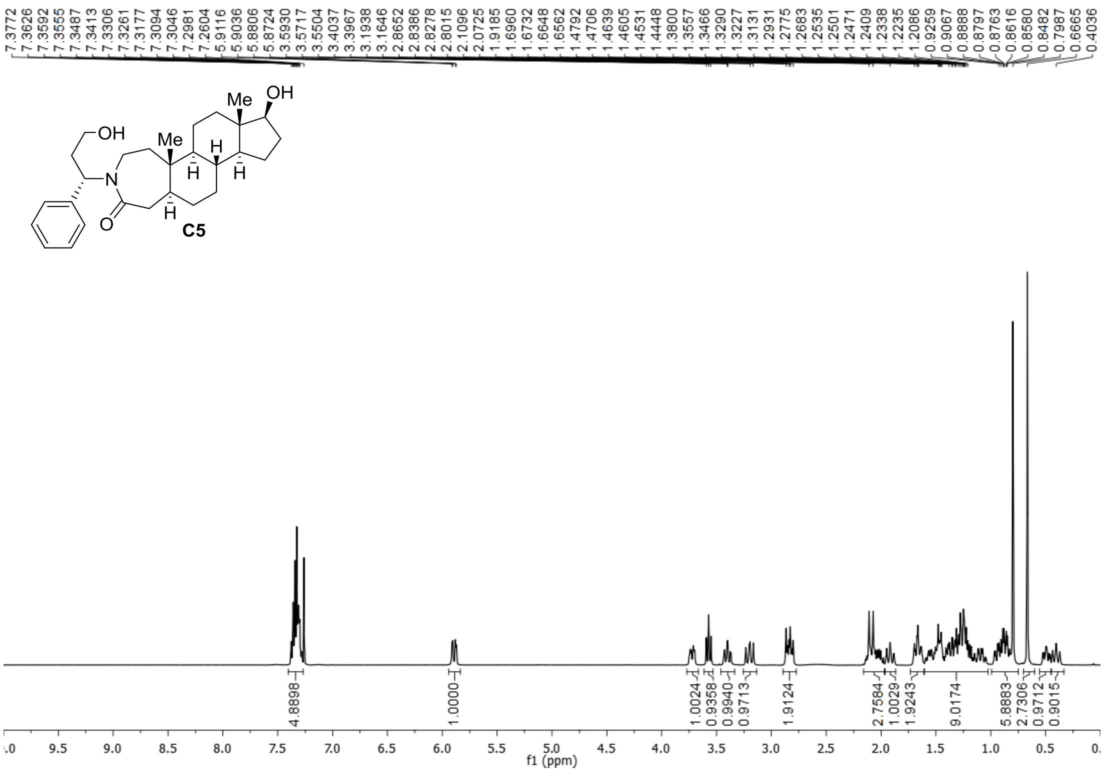

Supplementary Figure 156. ^{13}C NMR spectrum in $CDCl_3$, 101 MHz, of **C5**.

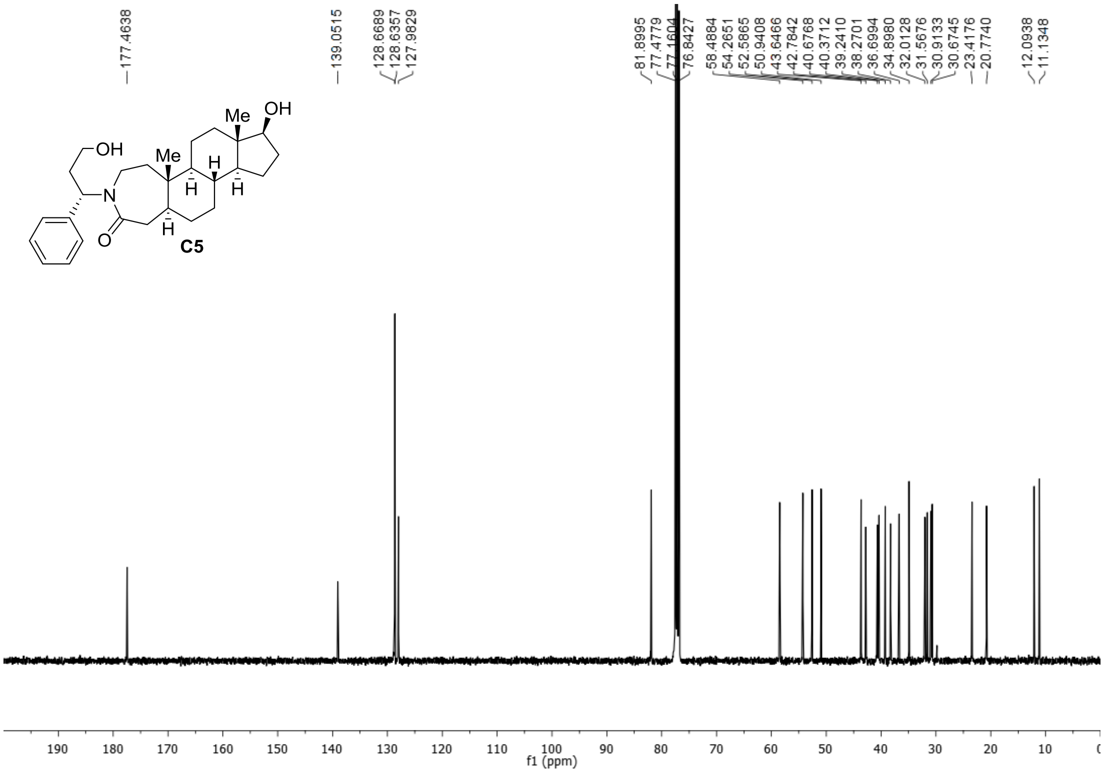

Supplementary Figure 157. ^{13}C NMR (APT) spectrum in $CDCl_3$, 101 MHz, of **C5**.

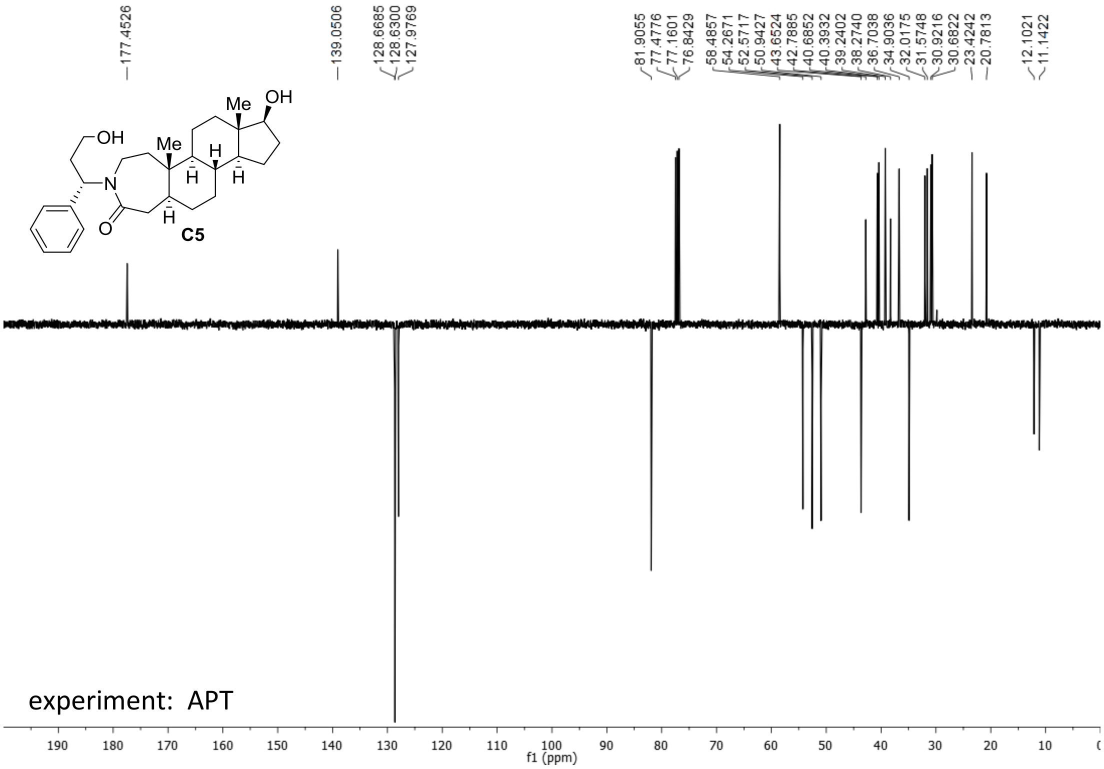

Supplementary Figure 158. 1D NOE spectrum in $CDCl_3$, 400 MHz, of **C5**.

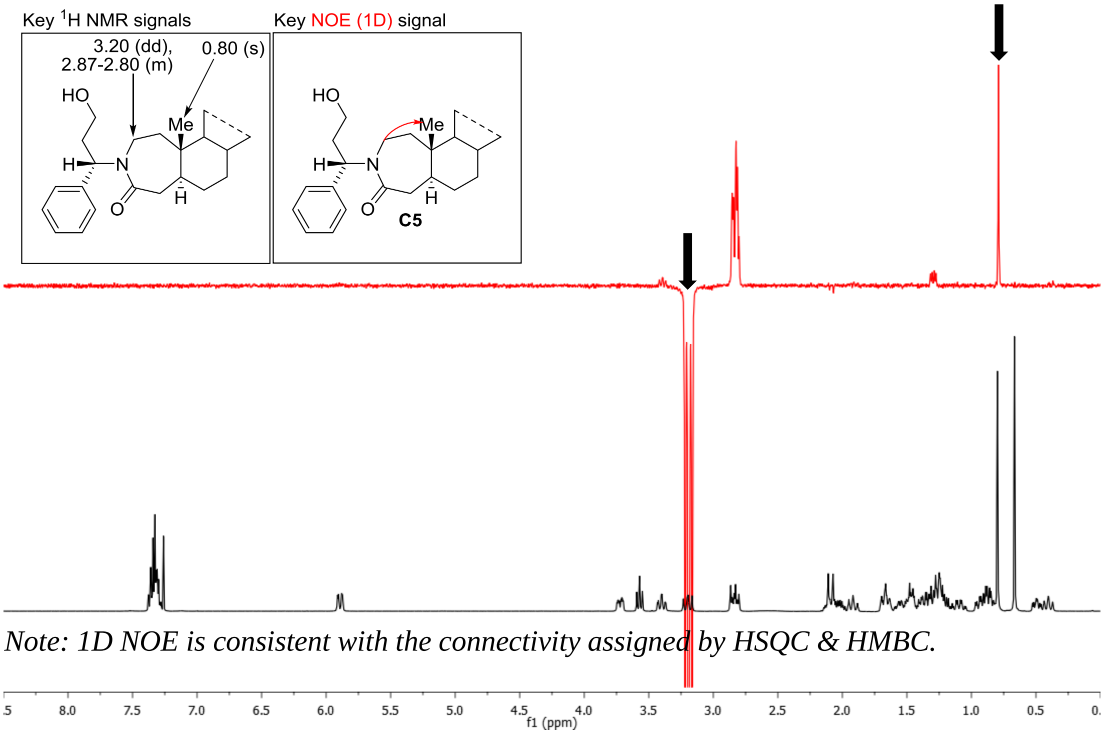

Note: 1D NOE is consistent with the connectivity assigned by HSQC & HMBC.

Supplementary Figure 159. HSQC spectrum of **C5**.

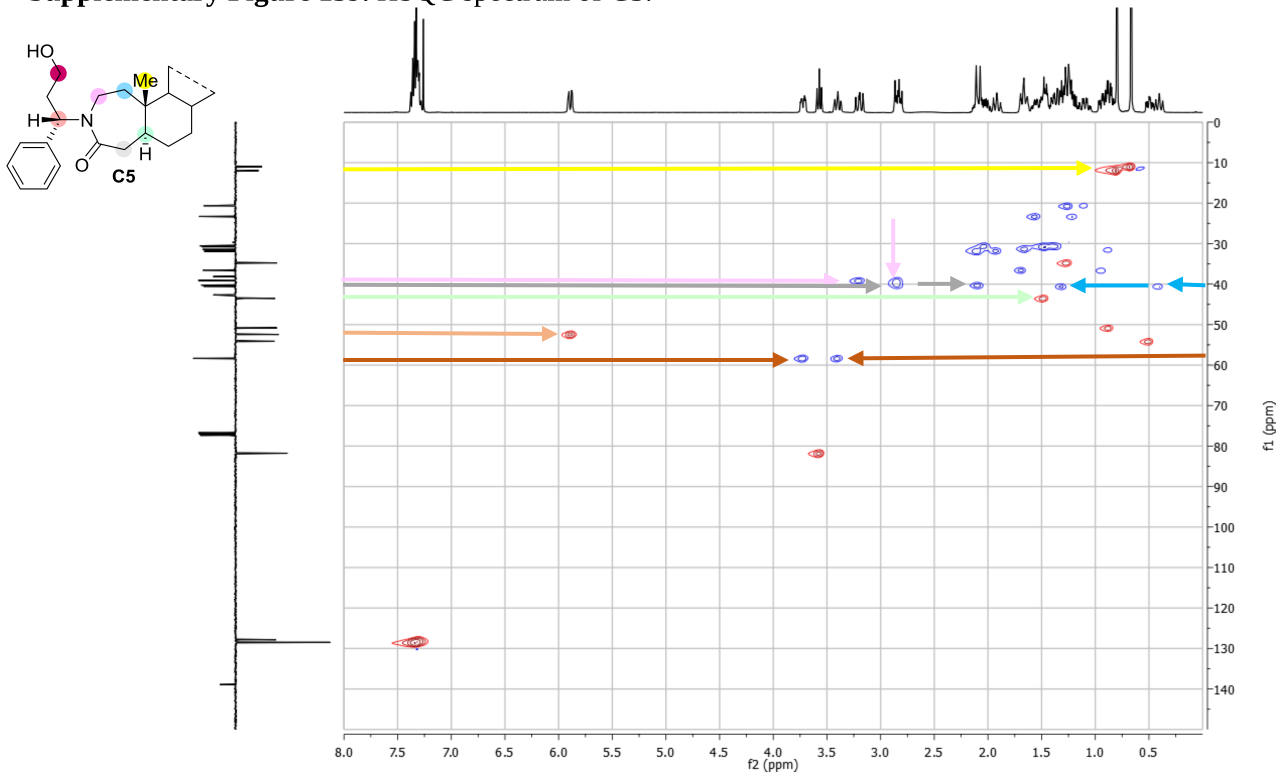

Supplementary Figure 160. HMBC spectrum of **C5**.

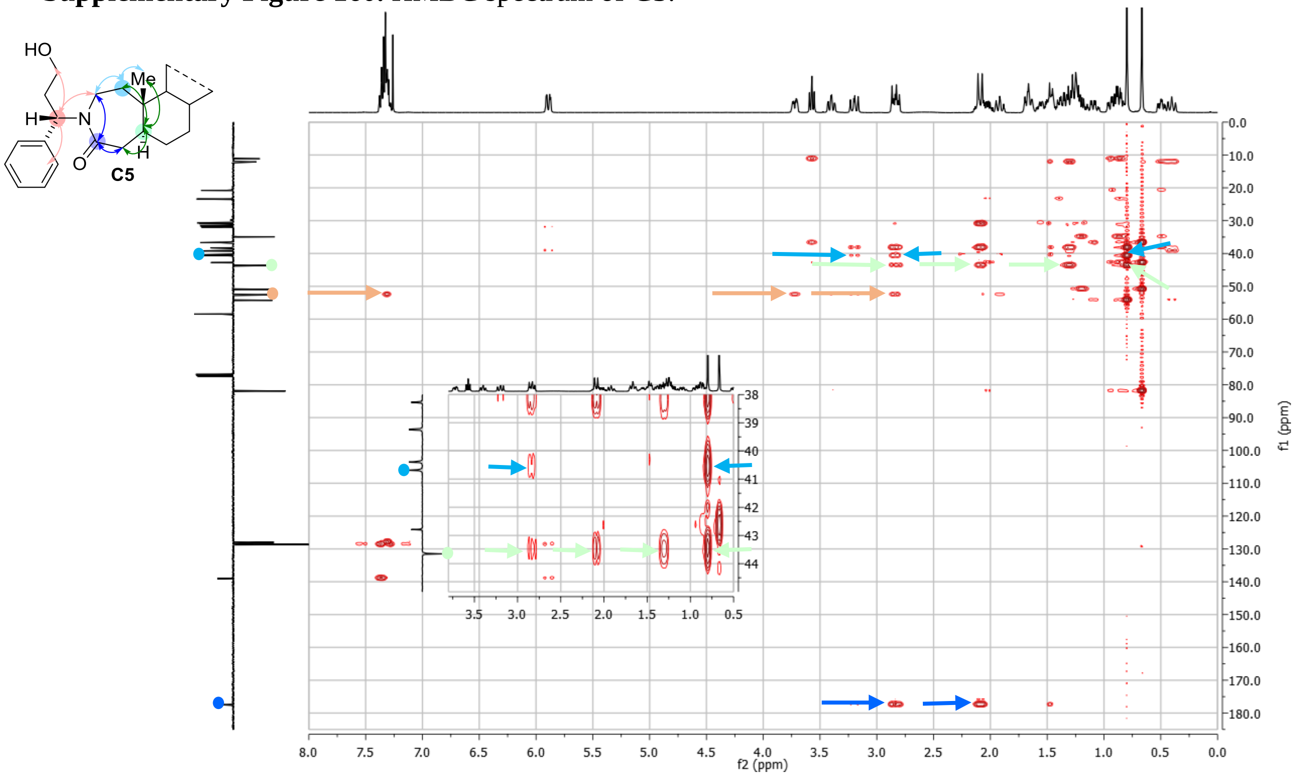

Supplementary Figure 161. ^{1}H NMR spectrum in $CDCl_3$, 400 MHz, of **C6**.

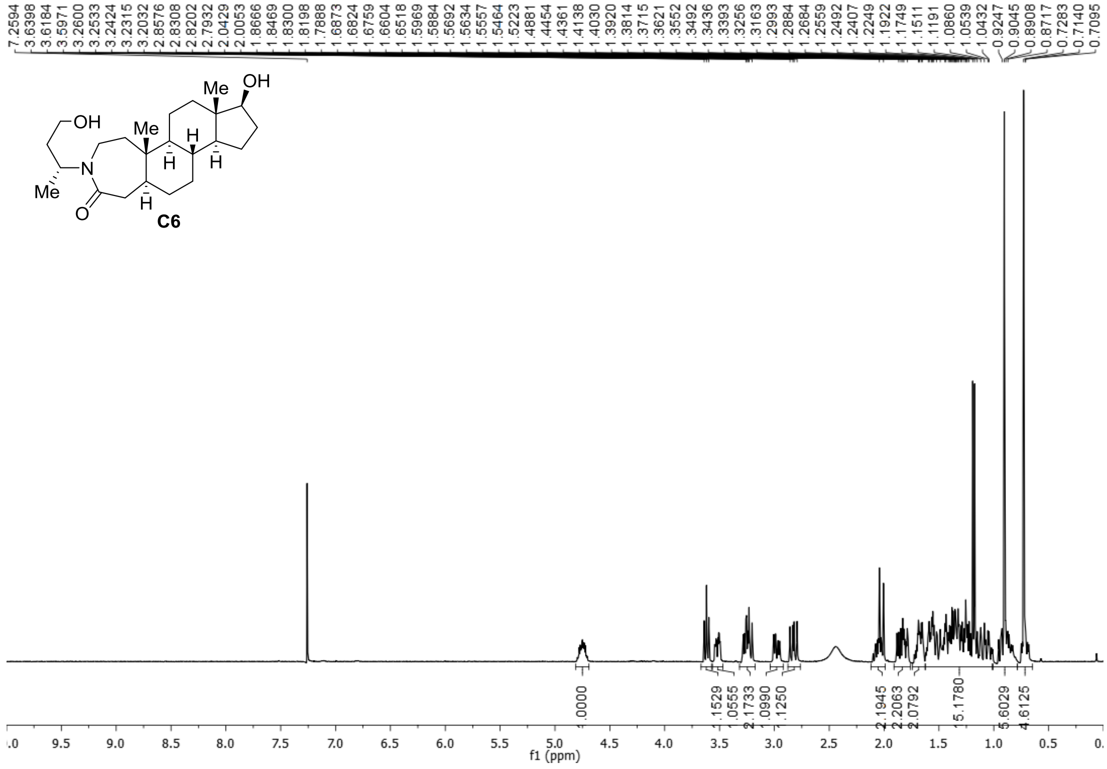

Supplementary Figure 162. ^{13}C NMR spectrum in $CDCl_3$, 101 MHz, of **C6**.

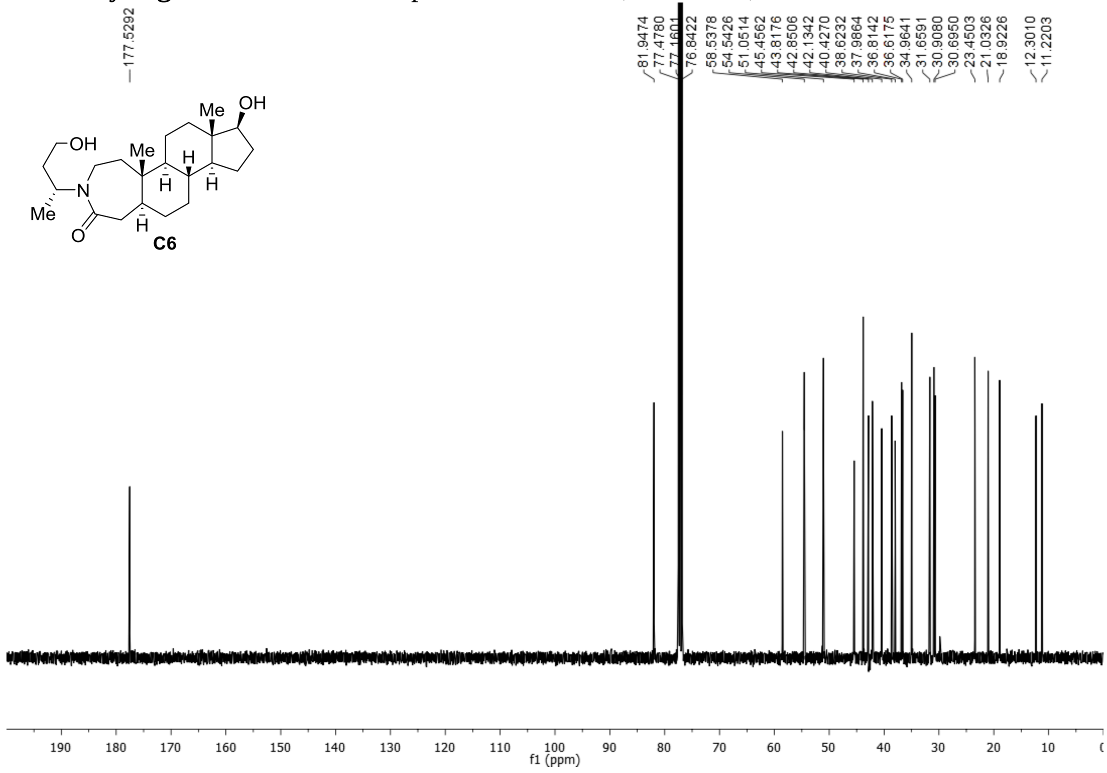

Supplementary Figure 163. ^{13}C NMR (APT) spectrum in $CDCl_3$, 101 MHz, of **C6**.

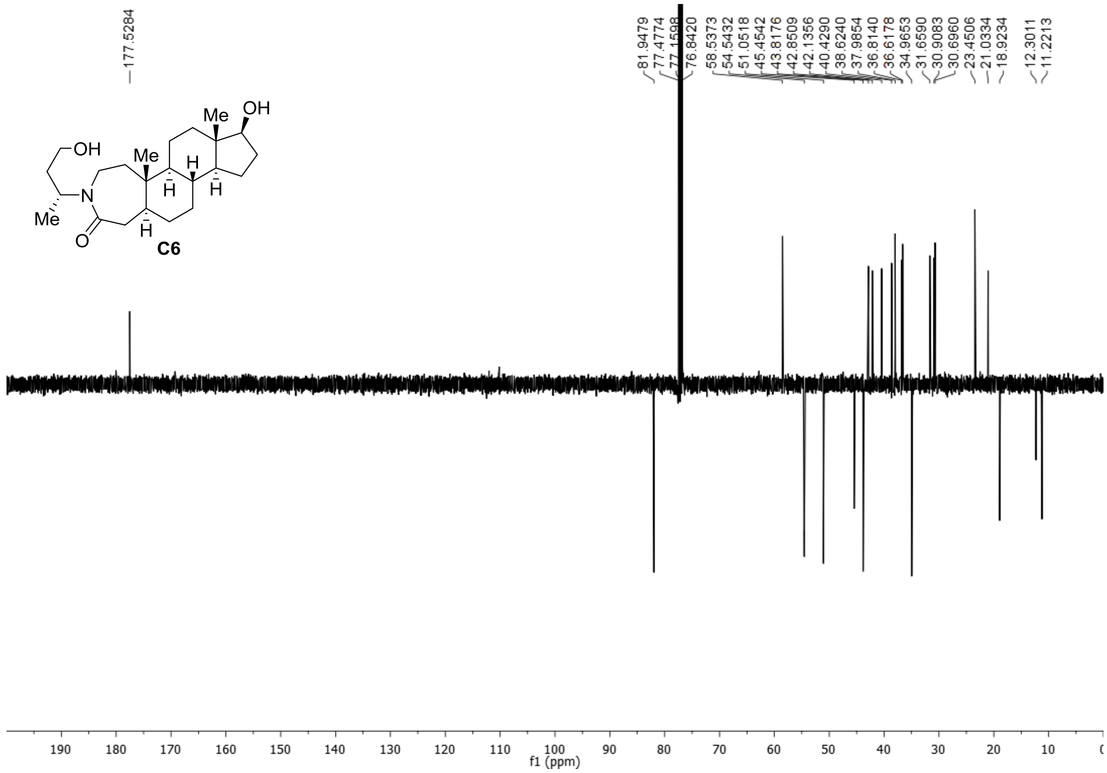

Supplementary Figure 164. 1D NOE spectrum in $CDCl_3$, 400 MHz, of **C6**.

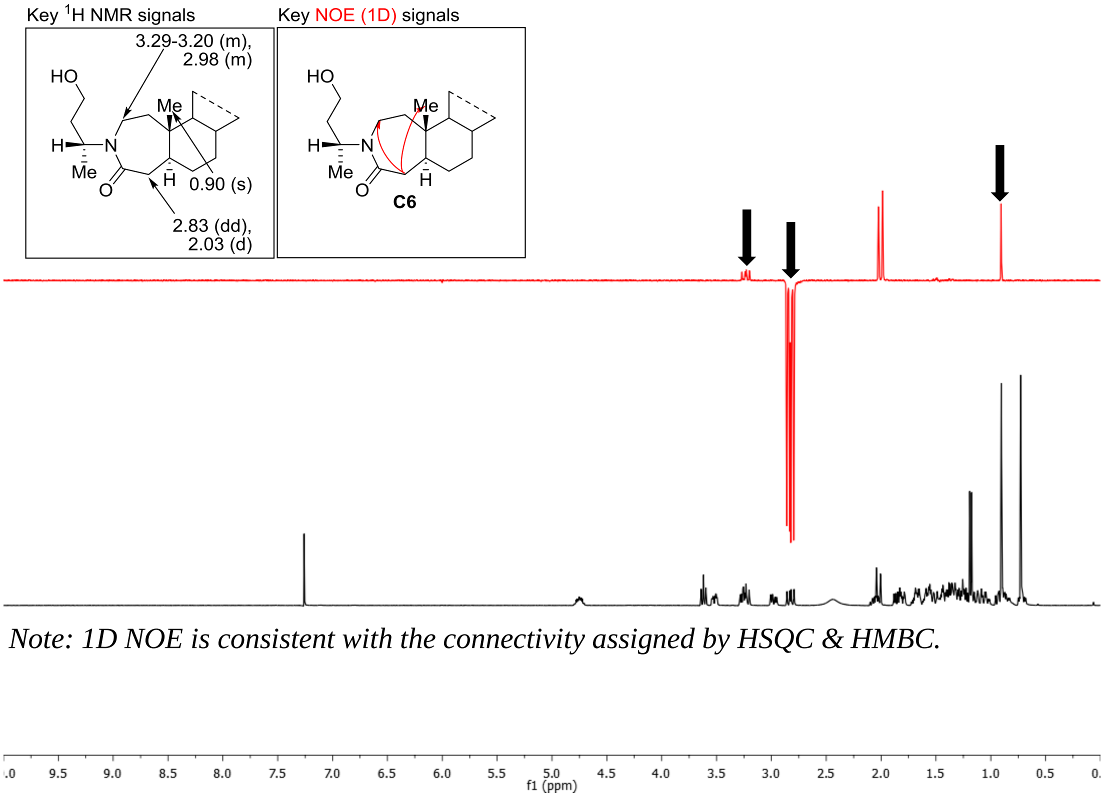

Supplementary Figure 165. 1D NOE spectrum in $CDCl_3$, 400 MHz, of **C6**.

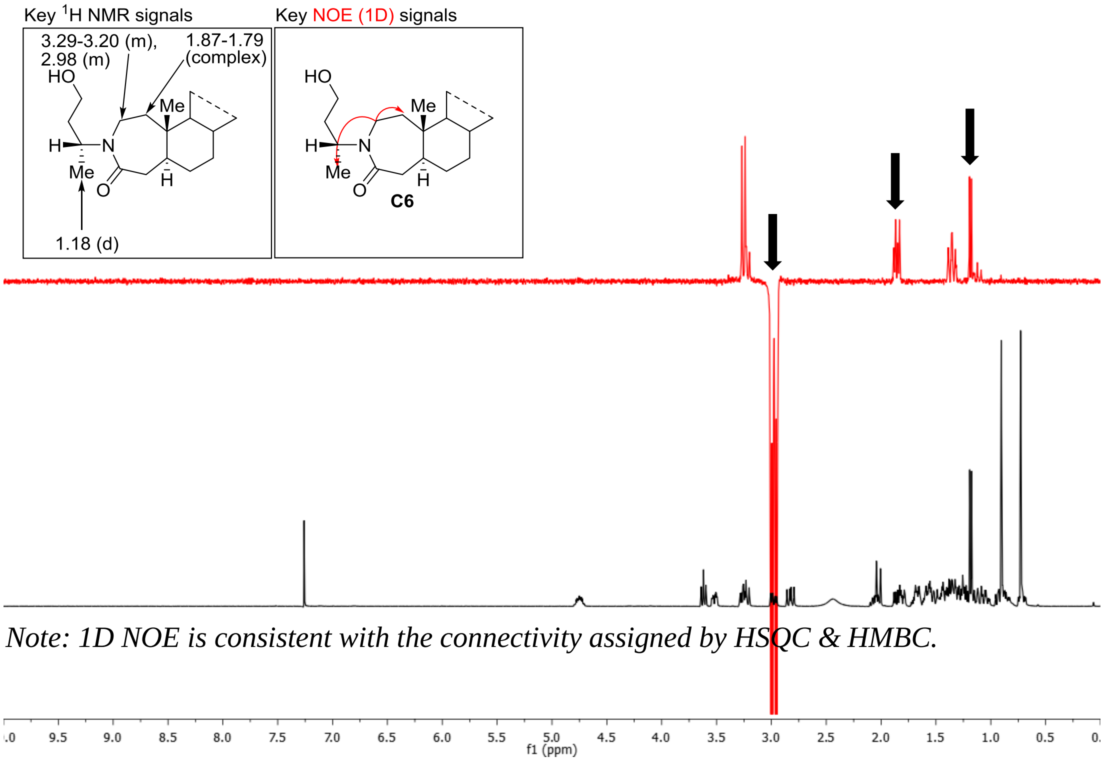

Supplementary Figure 166. HSQC spectrum of **C6**.

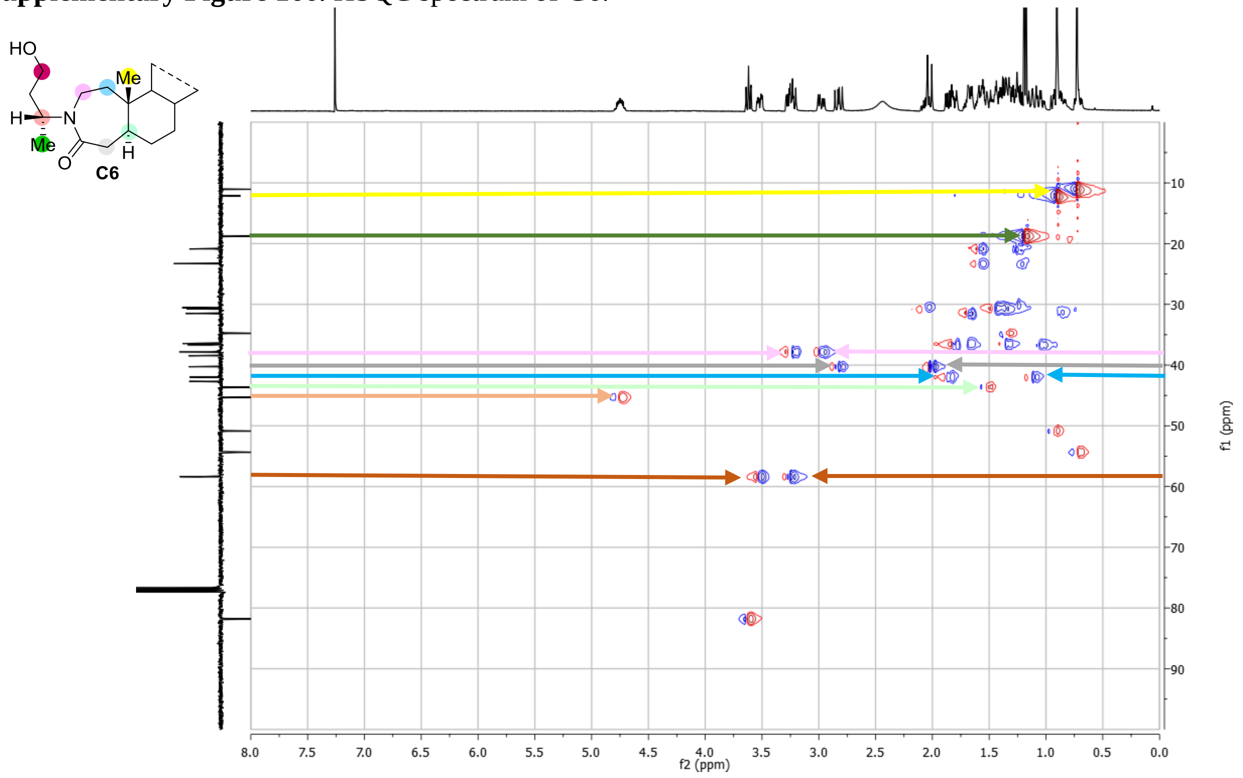

Supplementary Figure 167. HMBC spectrum of **C6**.

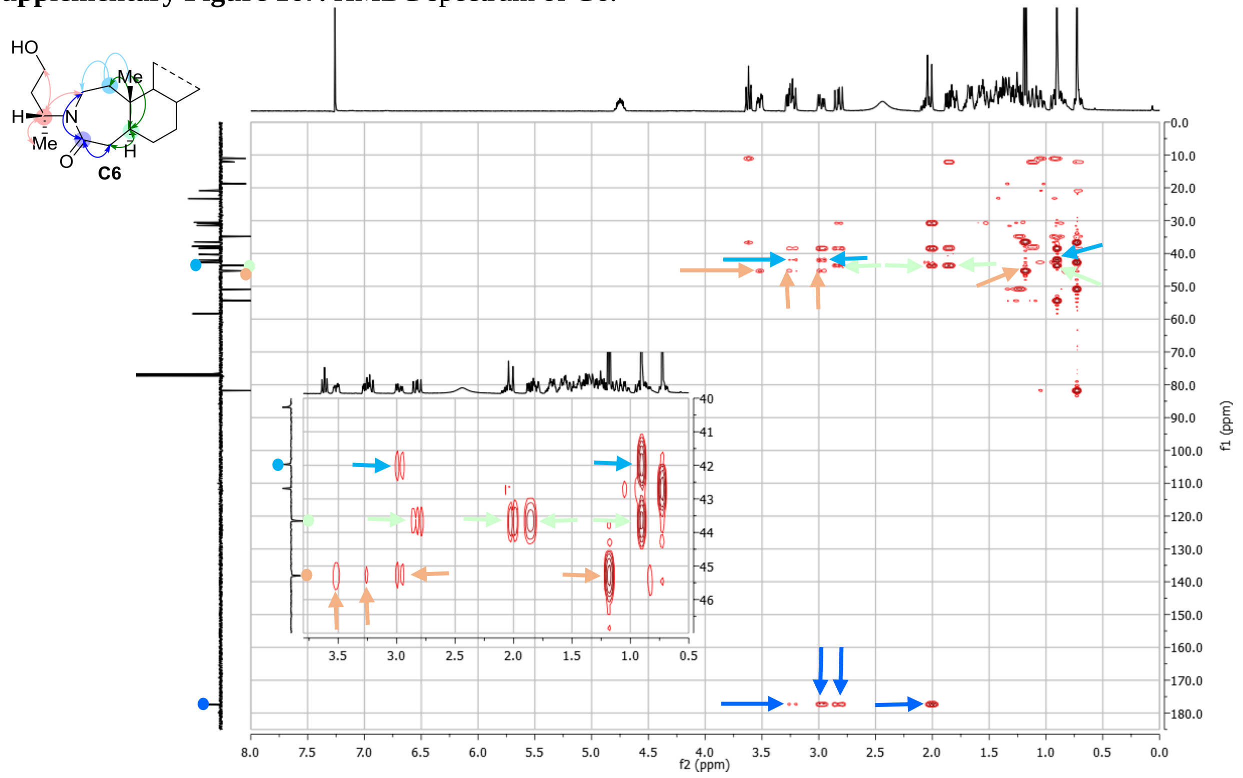

Supplementary Figure 168. ^{1}H NMR spectrum in $CDCl_3$, 400 MHz, of **C7**.

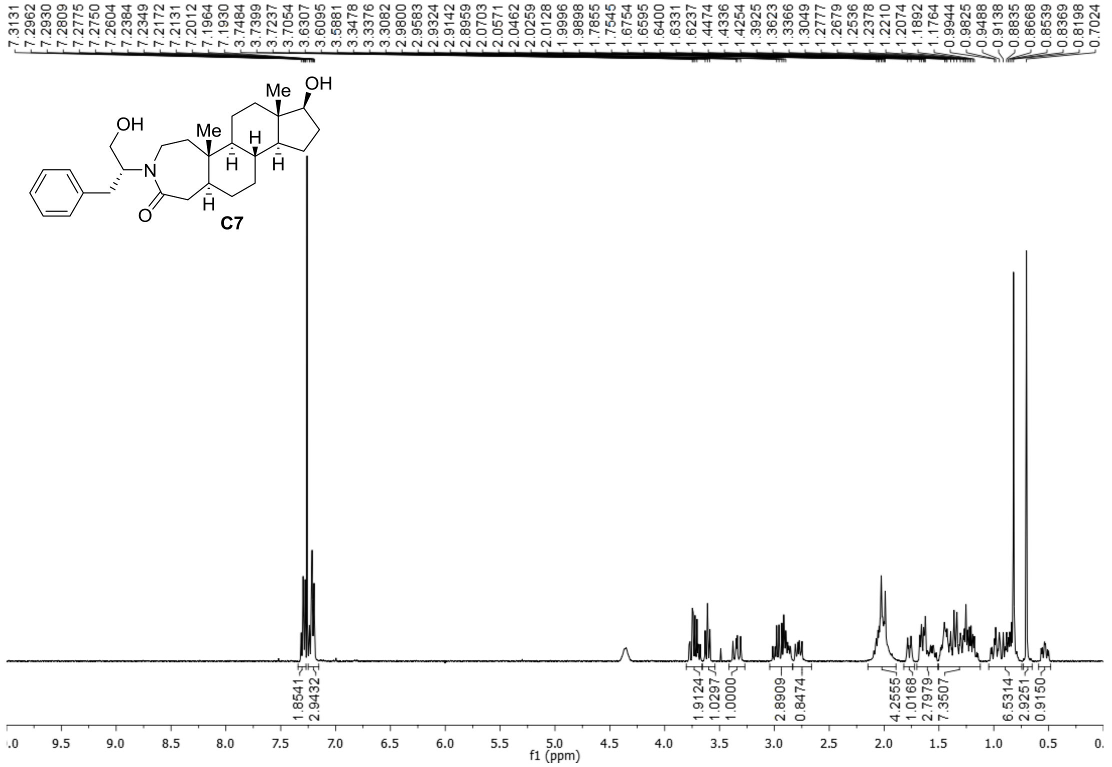

Supplementary Figure 169. ^{13}C NMR spectrum in $CDCl_3$, 101 MHz, of **C7**.

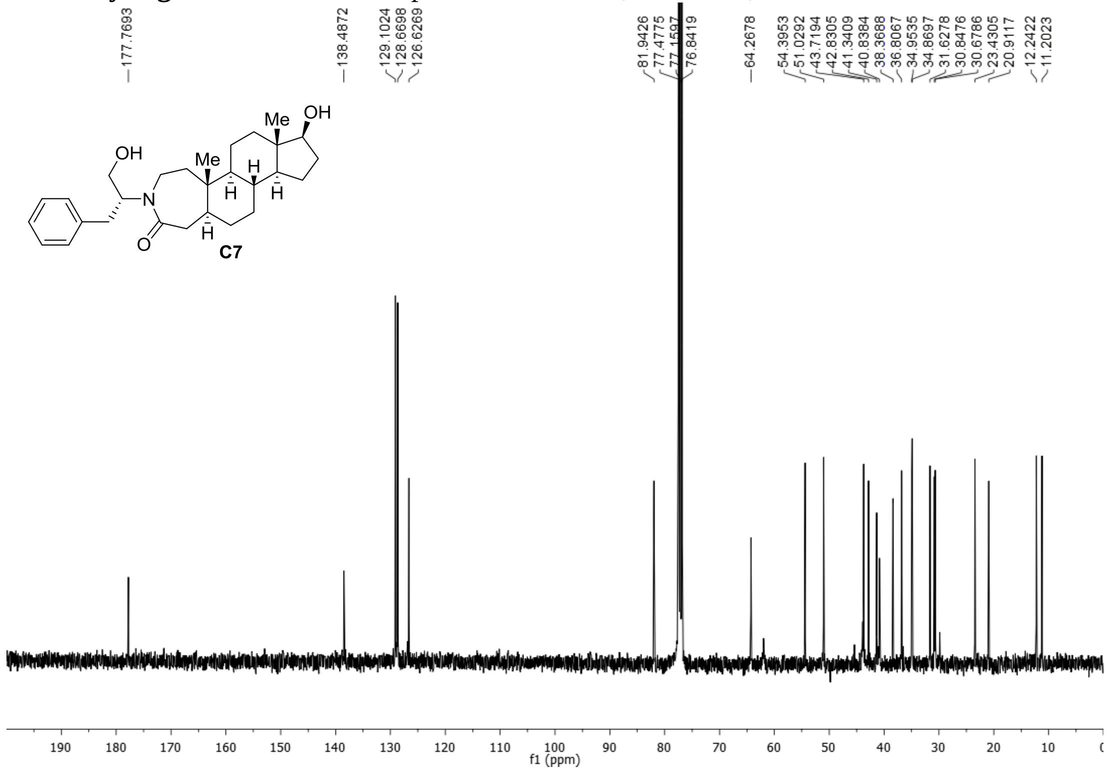

Supplementary Figure 170. ^{1}H NMR spectrum in $CDCl_3$, 400 MHz, of **C8**.

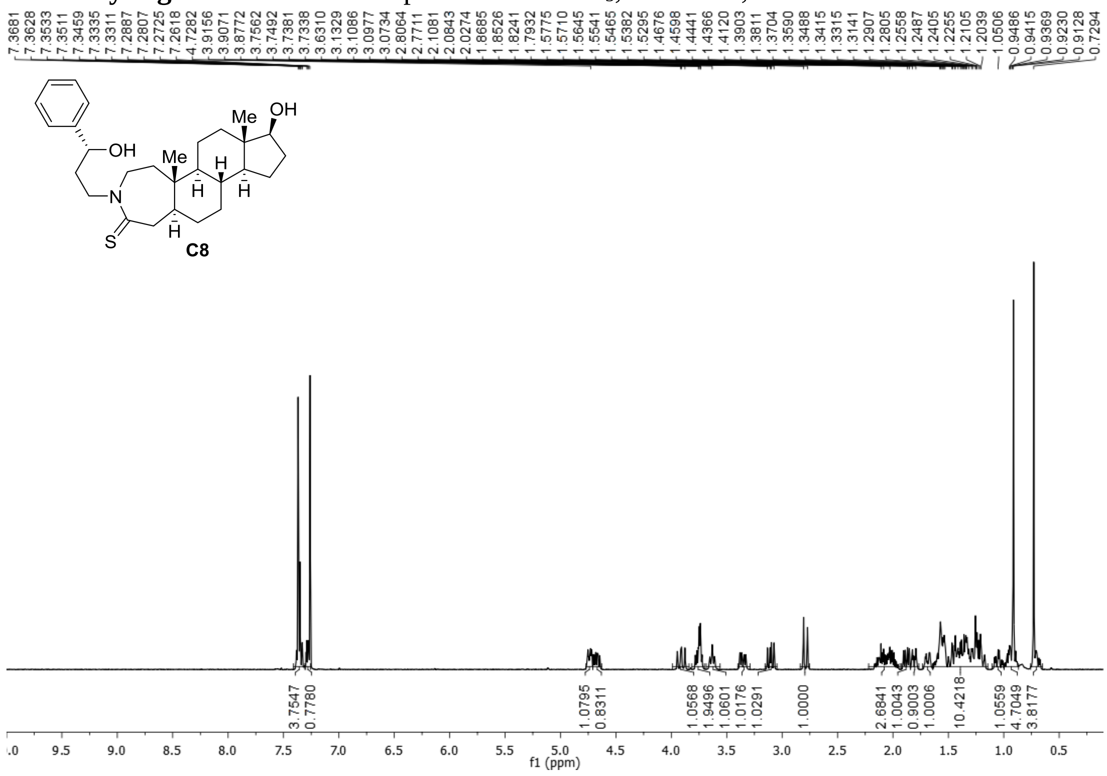

Supplementary Figure 171. ^{13}C NMR spectrum in $CDCl_3$, 151 MHz, of **C8**.

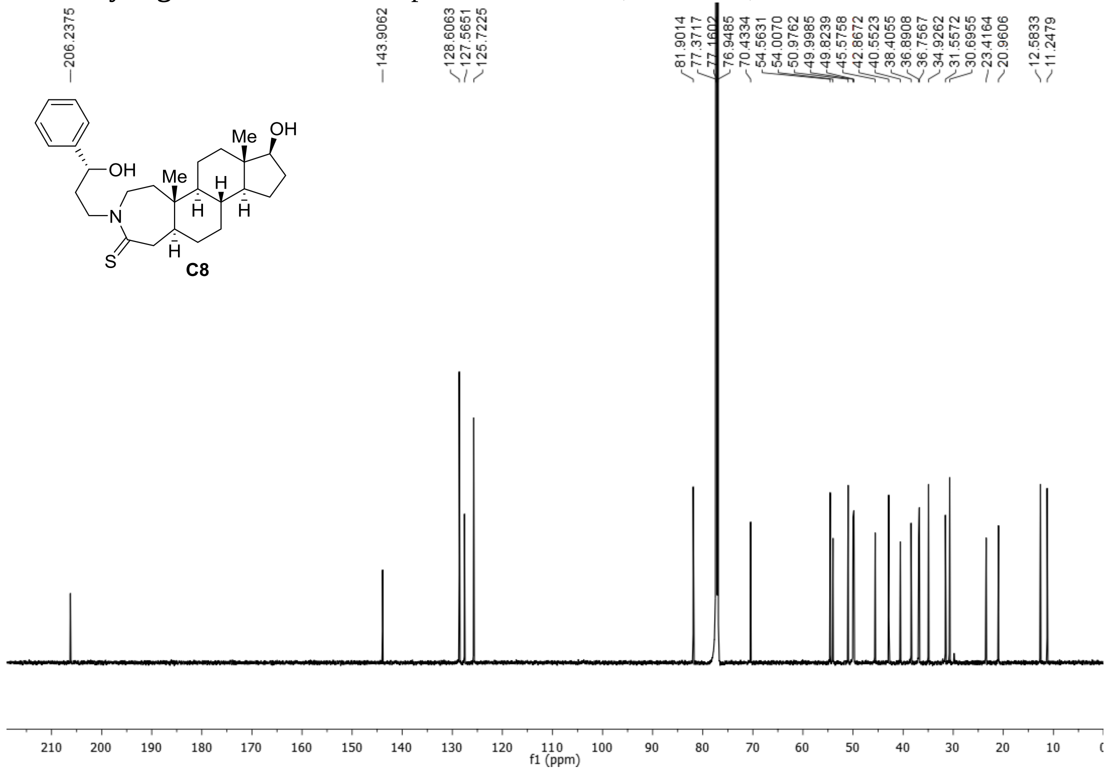

Supplementary Figure 172. ^{1}H NMR spectrum in $CDCl_3$, 400 MHz, of **C9**.

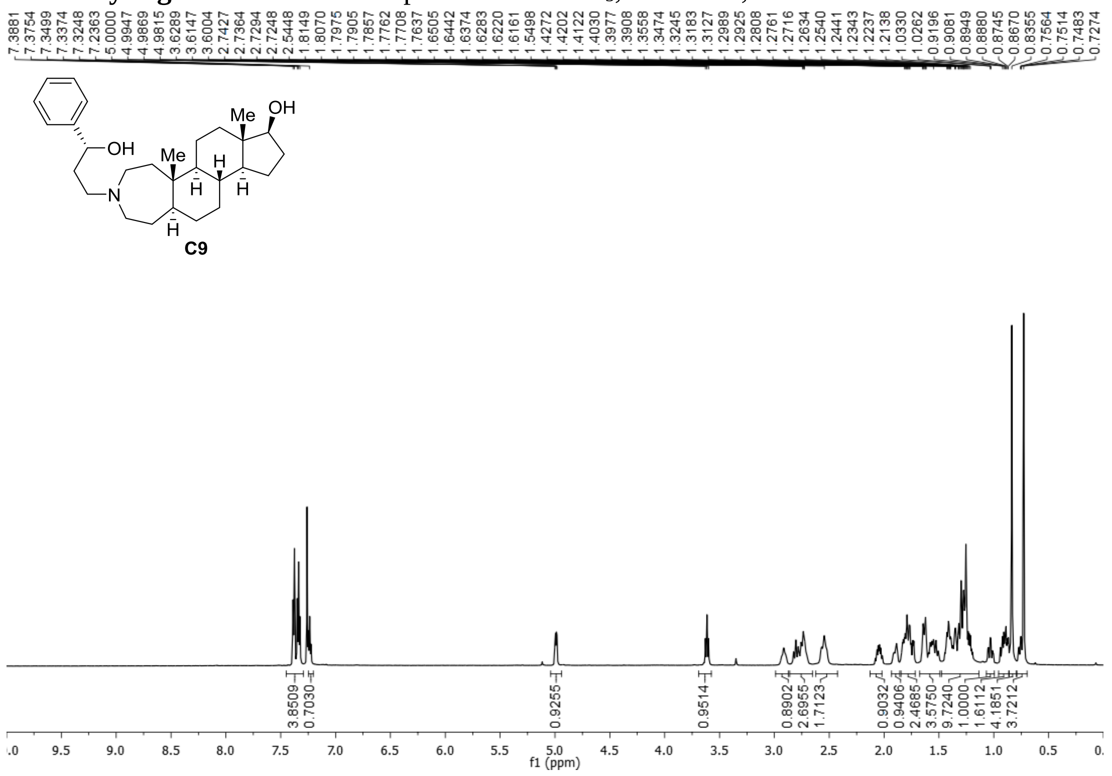

Supplementary Figure 173. ^{13}C NMR (APT) spectrum in $CDCl_3$, 151 MHz, of **C9**.

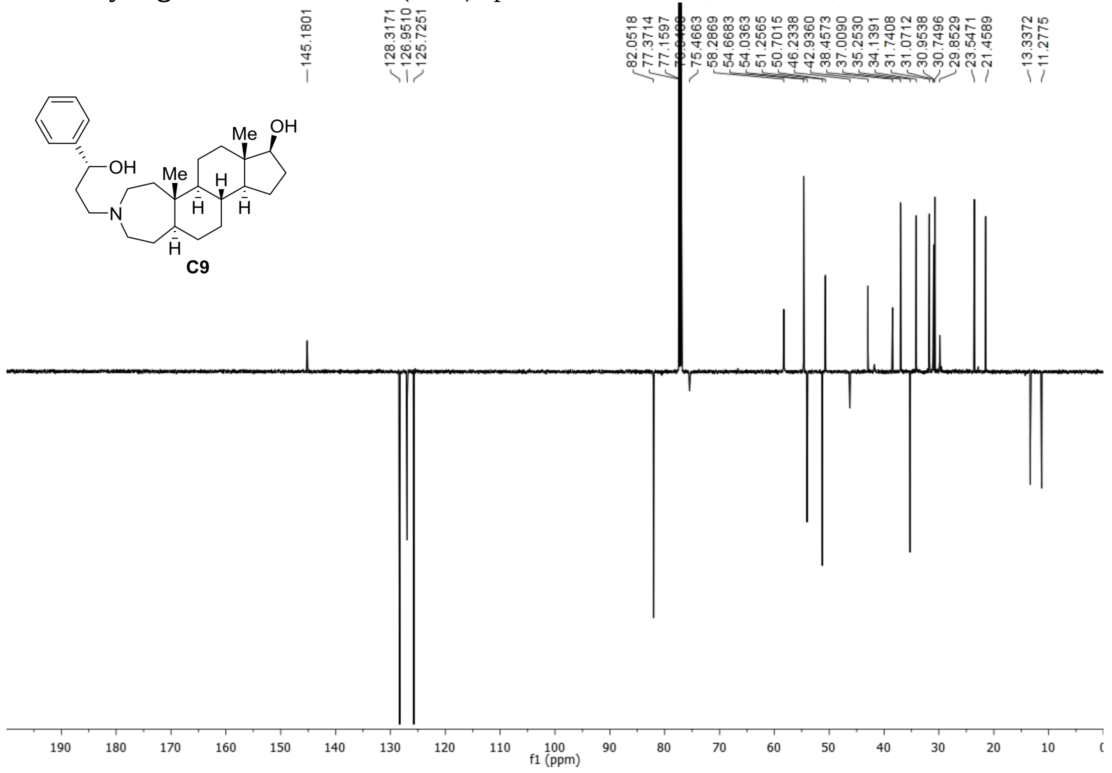

Supplementary Figure 174. ^{1}H NMR spectrum in $CDCl_3$, 400 MHz, of **C10**.

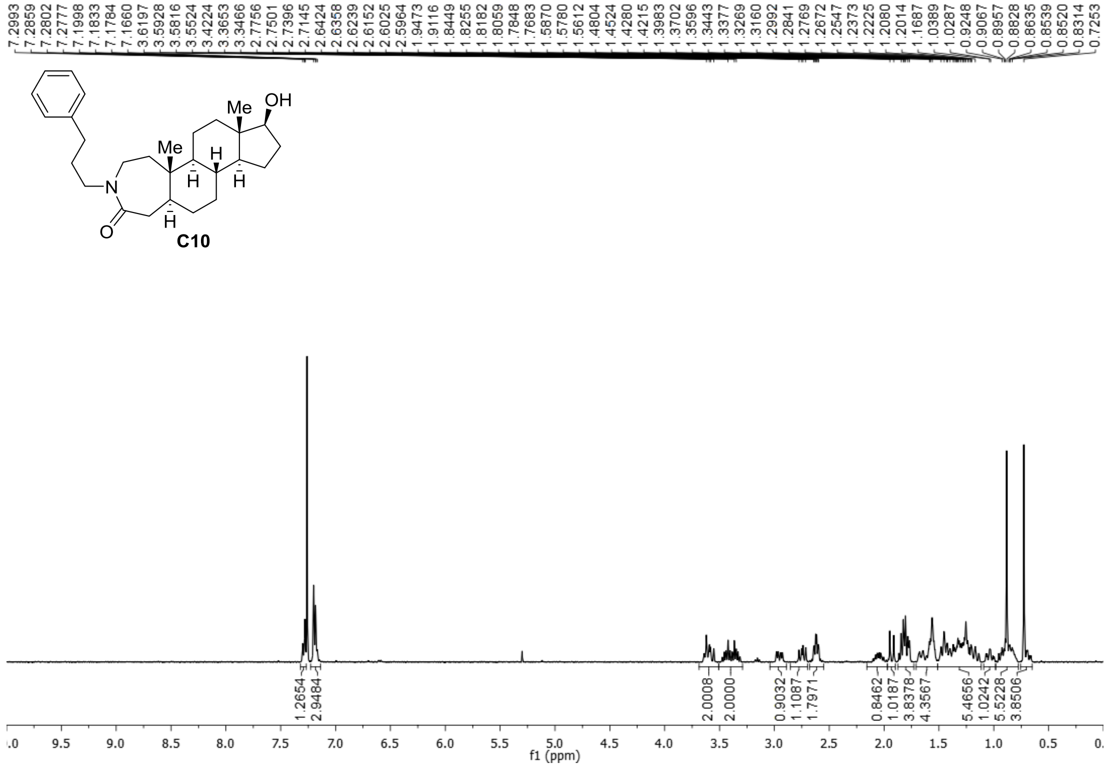

Supplementary Figure 175. ^{13}C NMR (APT) spectrum in $CDCl_3$, 101 MHz, of **C10**.

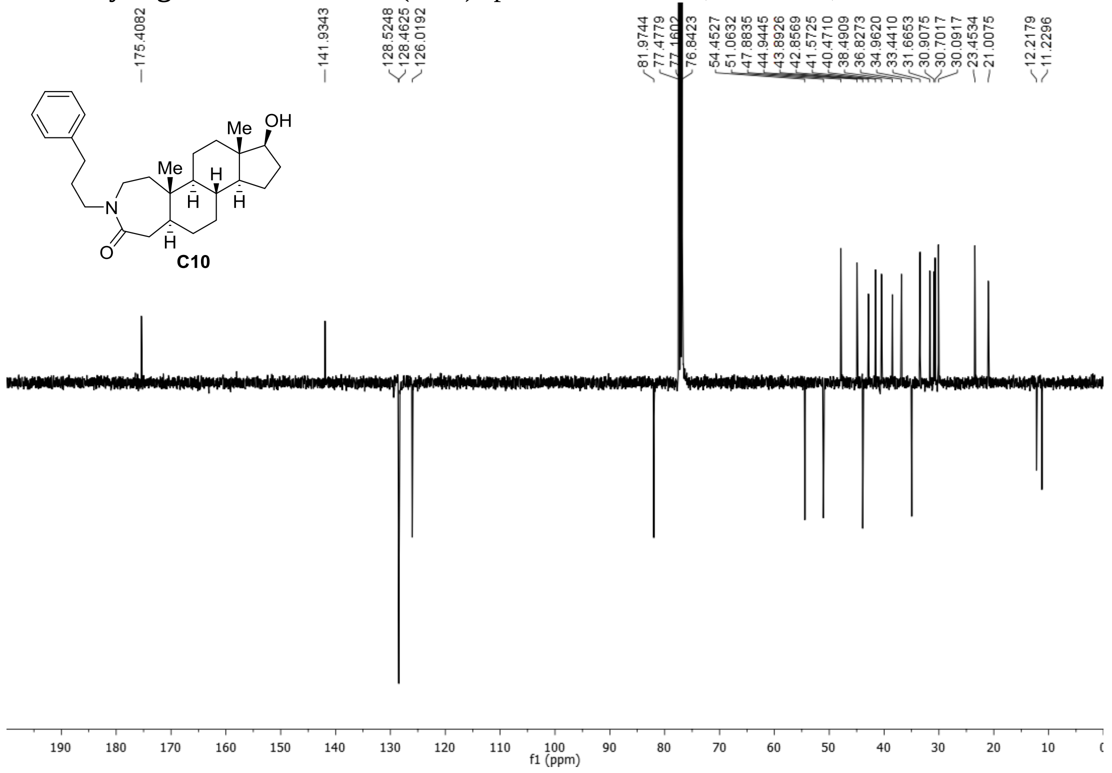

Supplementary Figure 176. ^{1}H NMR spectrum in $CDCl_3$, 400 MHz, of **C11**.

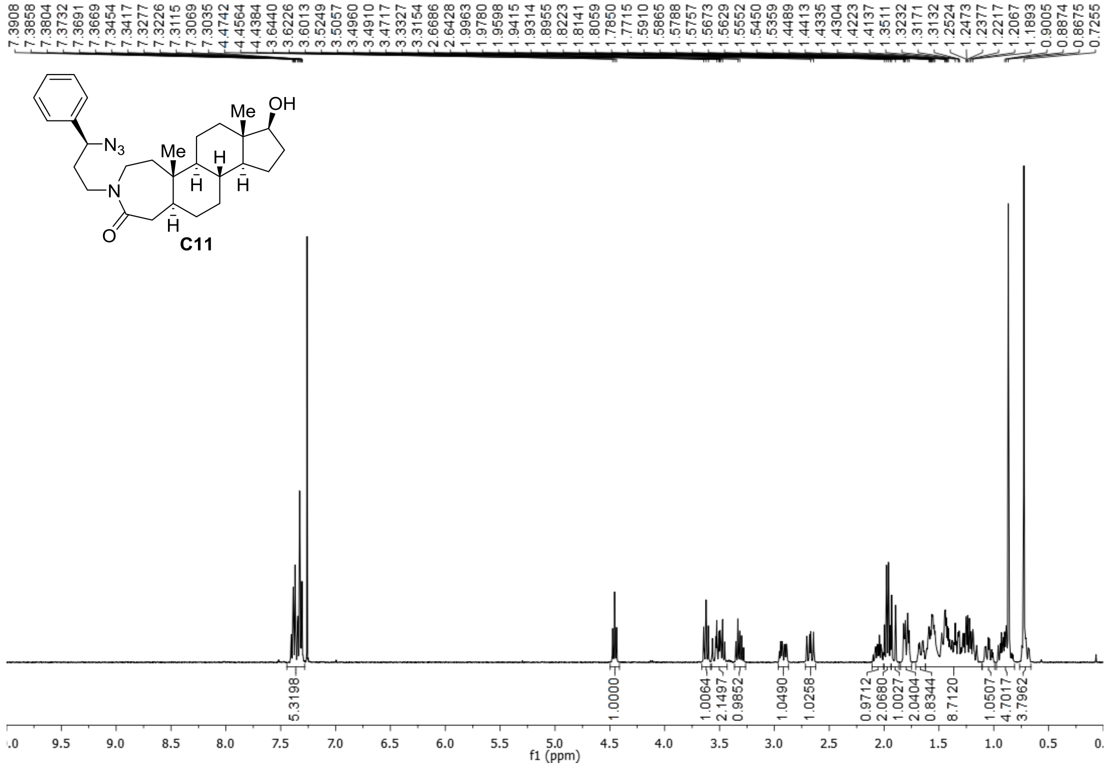

Supplementary Figure 177. ^{13}C NMR spectrum in $CDCl_3$, 101 MHz, of **C11**.

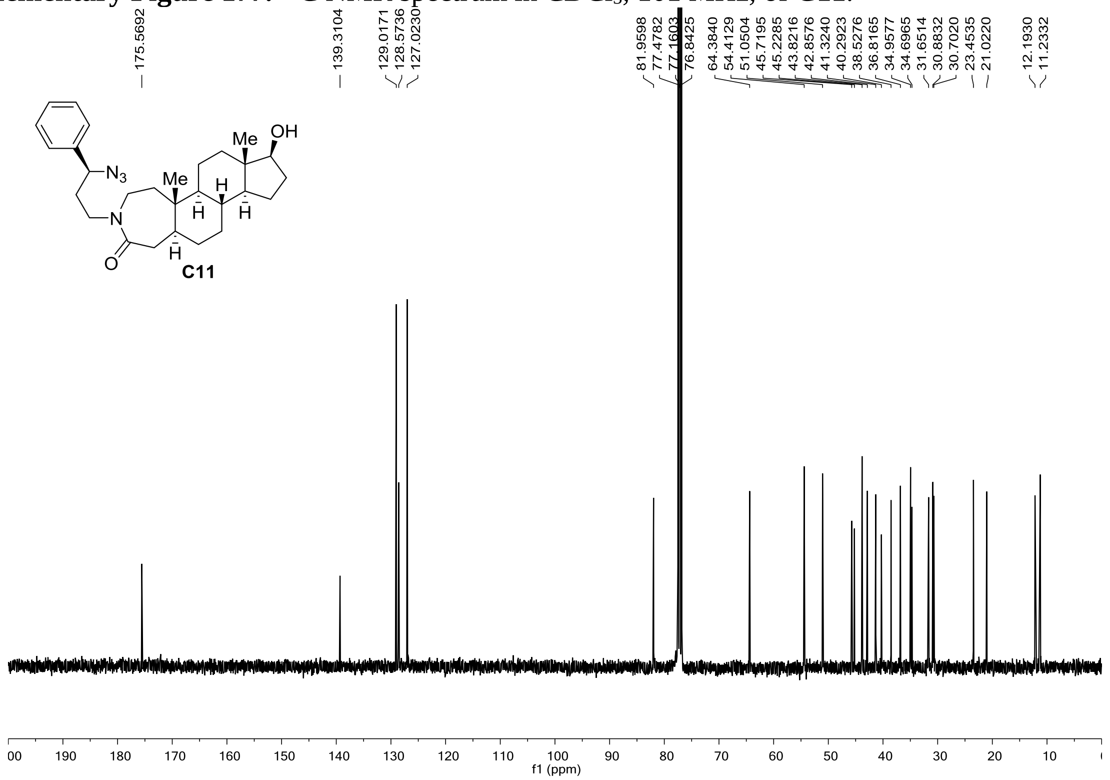

Supplementary Figure 178. ^{1}H NMR spectrum in $CDCl_3$, 400 MHz, of **C12**.

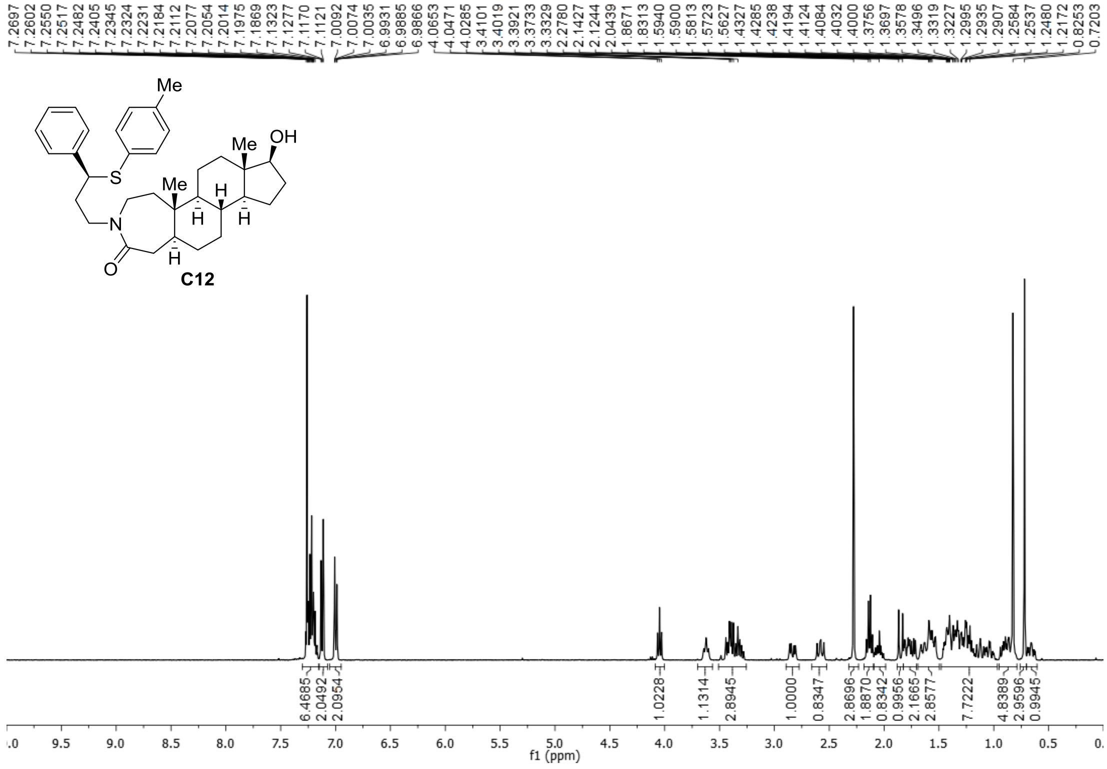

Supplementary Figure 179. ^{13}C NMR spectrum in $CDCl_3$, 126 MHz, of **C12**.

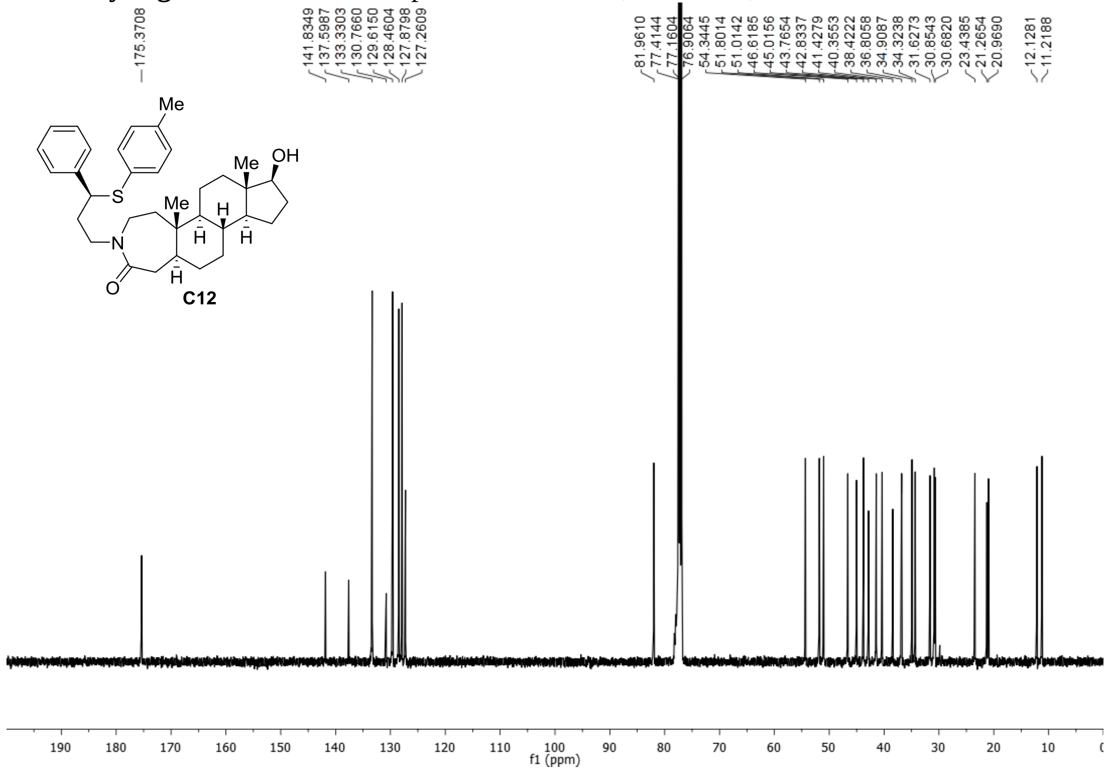

Supplementary Figure 180. ^{1}H NMR spectrum in $CDCl_3$, 400 MHz, of **C13**.

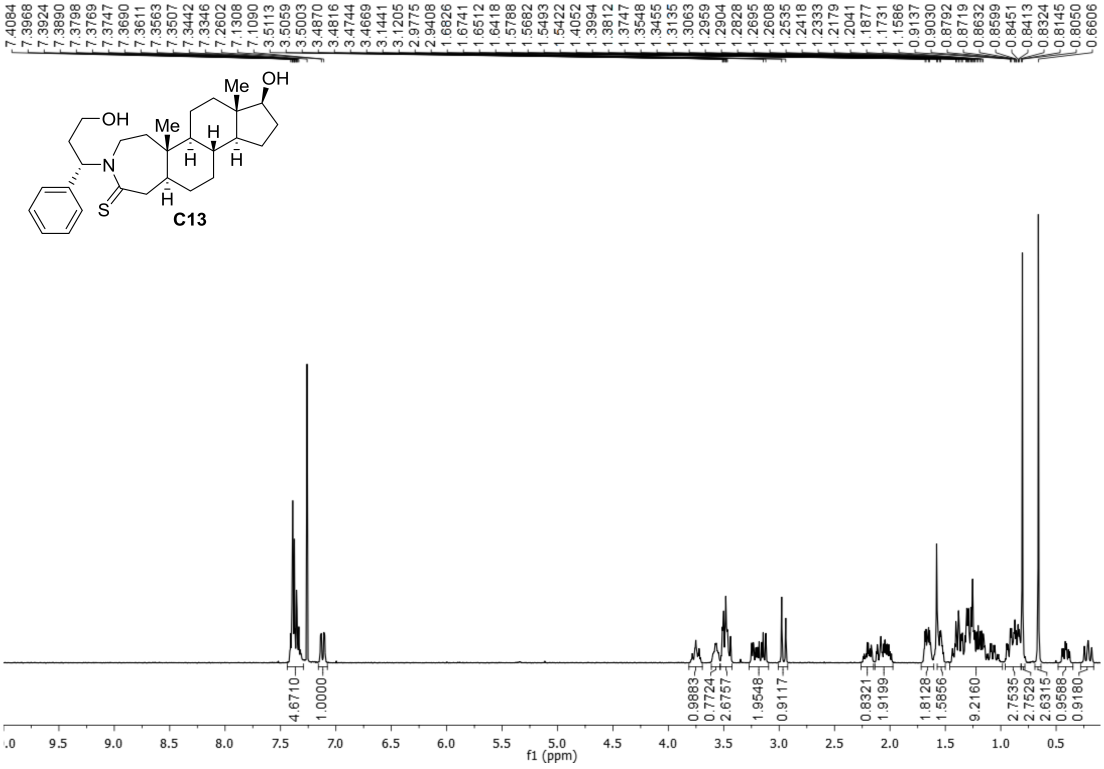

Supplementary Figure 181. ^{13}C NMR spectrum in $CDCl_3$, 151 MHz, of **C13**.

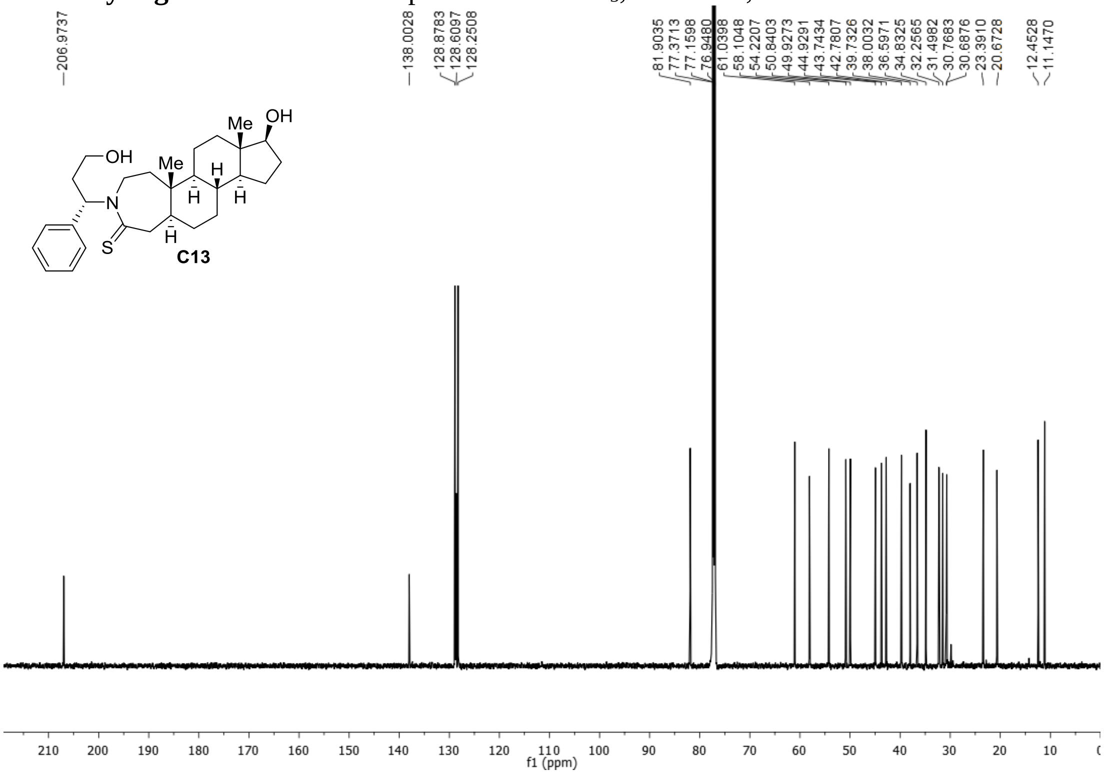

Supplementary Figure 182. ^{1}H NMR spectrum in $CDCl_3$, 400 MHz, of **C14**.

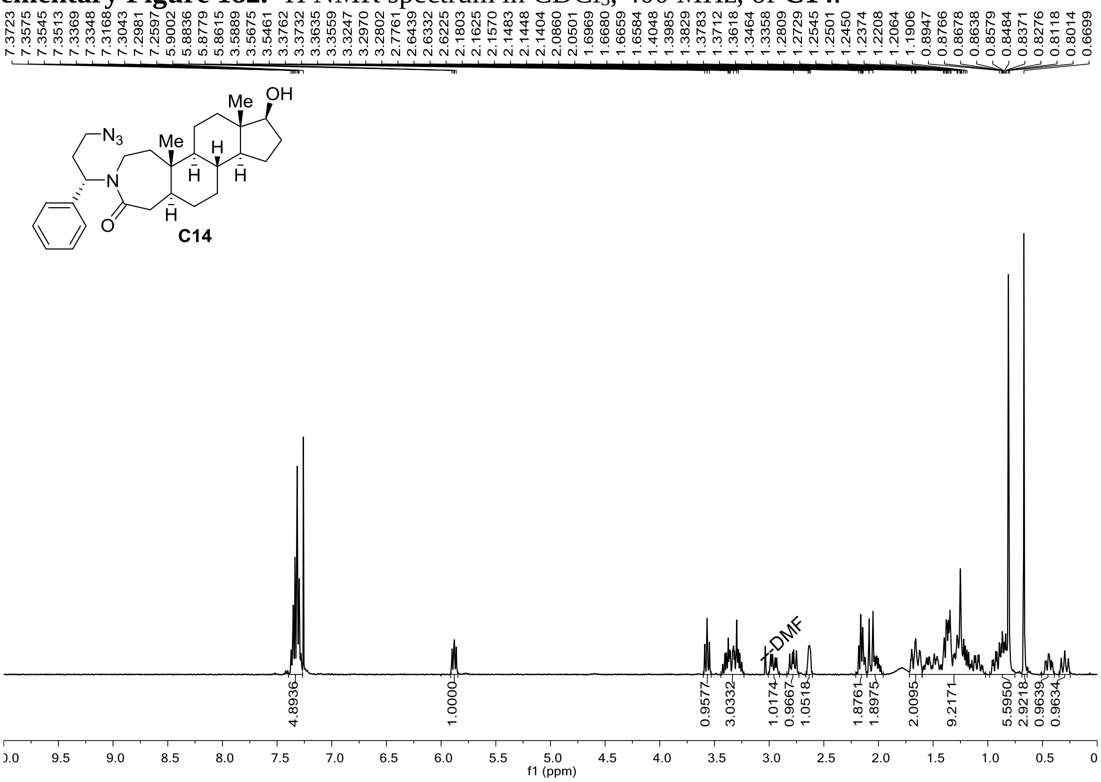

Supplementary Figure 183. ^{13}C NMR spectrum in $CDCl_3$, 101 MHz, of **C14**.

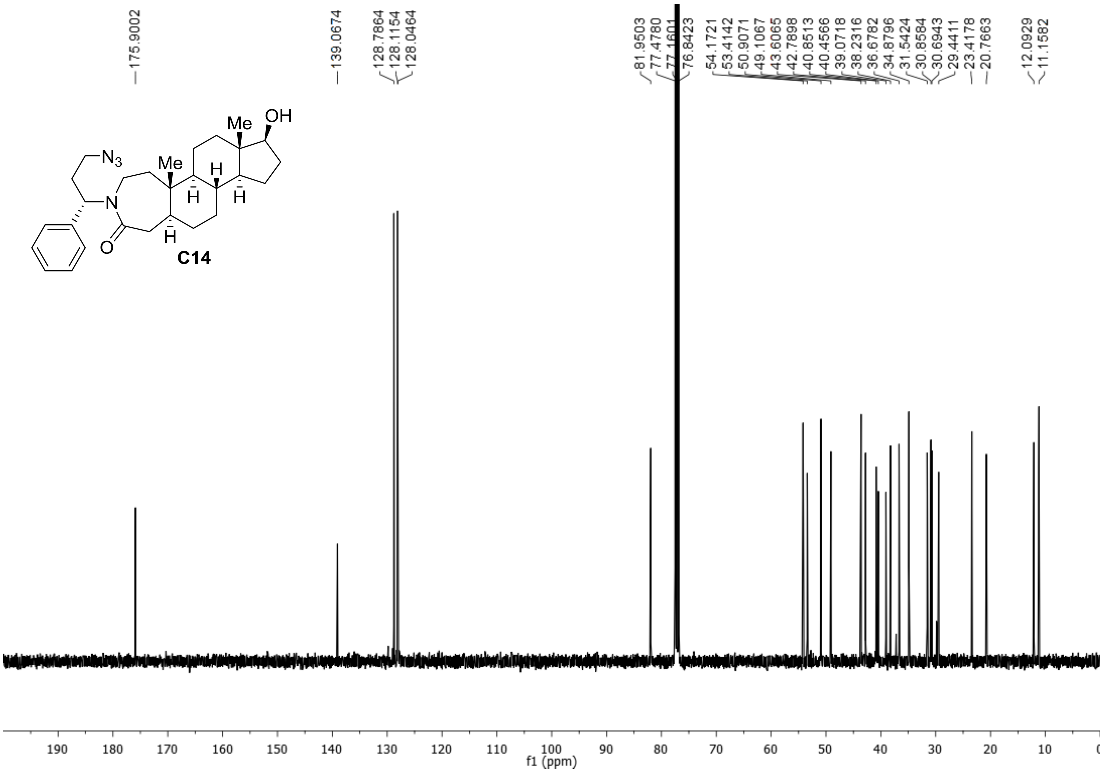

Supplementary Figure 184. ^{1}H NMR spectrum in $CDCl_3$, 400 MHz, of **C15**.

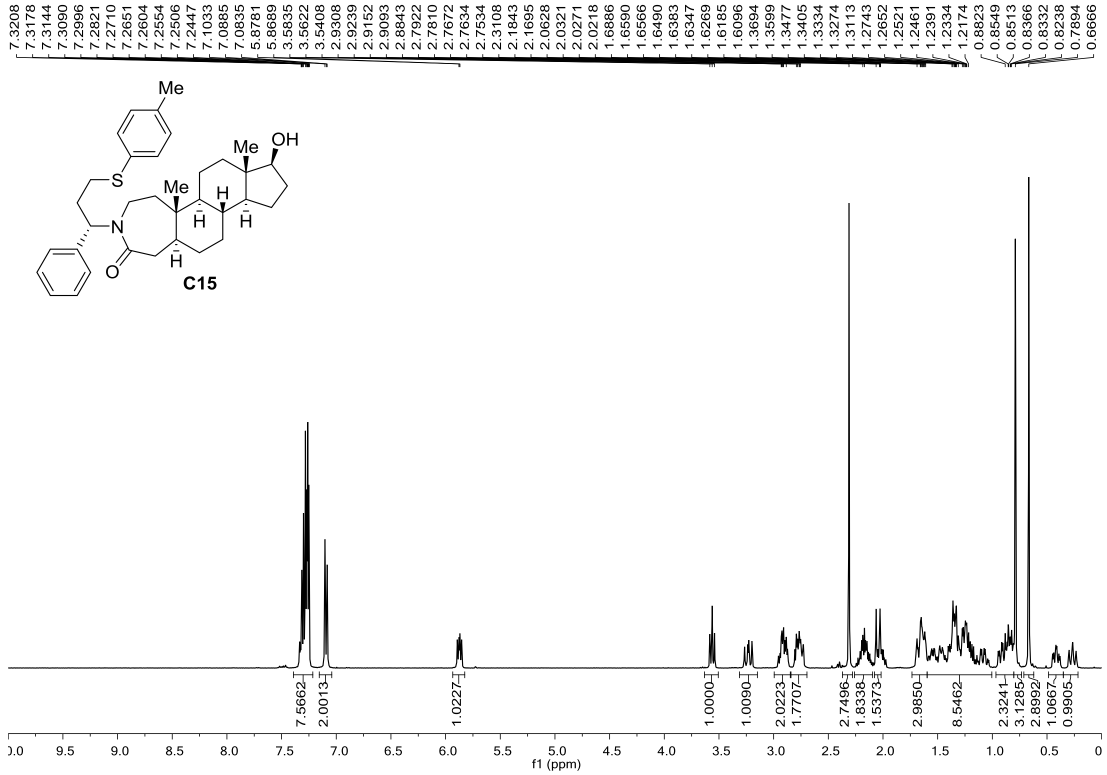

Supplementary Figure 185. ^{13}C NMR spectrum in $CDCl_3$, 101 MHz, of **C15**.

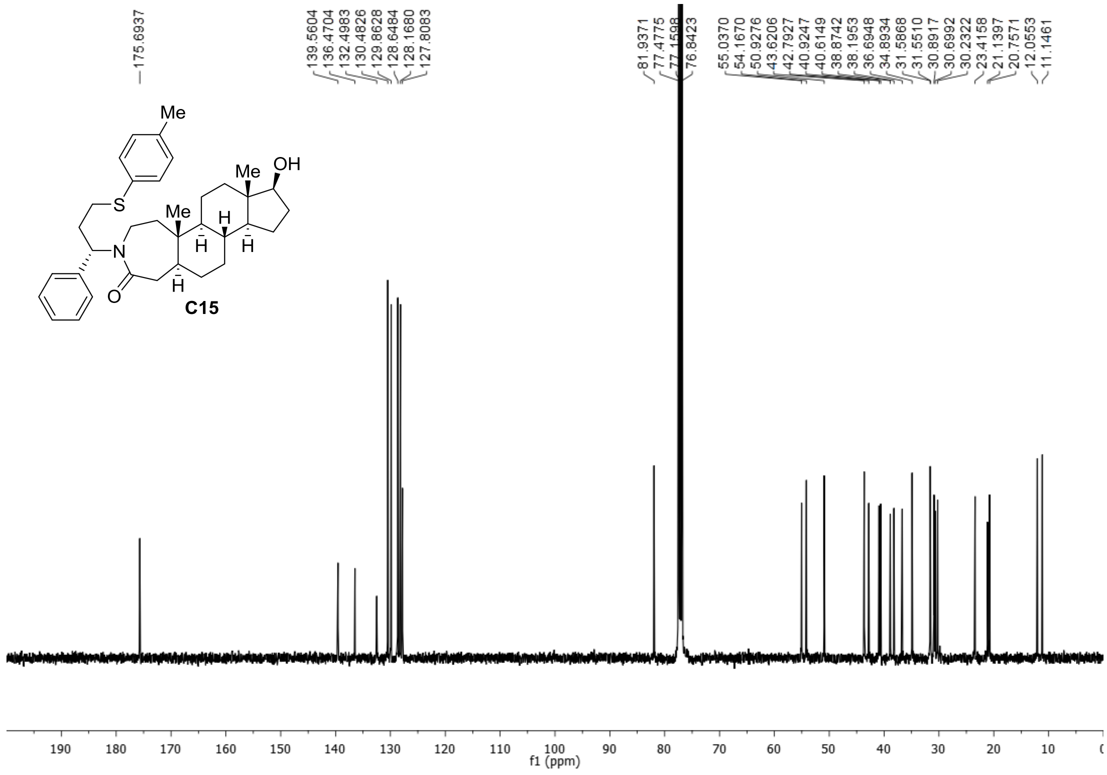

Supplementary Figure 186. ^{1}H NMR spectrum in $CDCl_3$, 400 MHz, of **C16**.

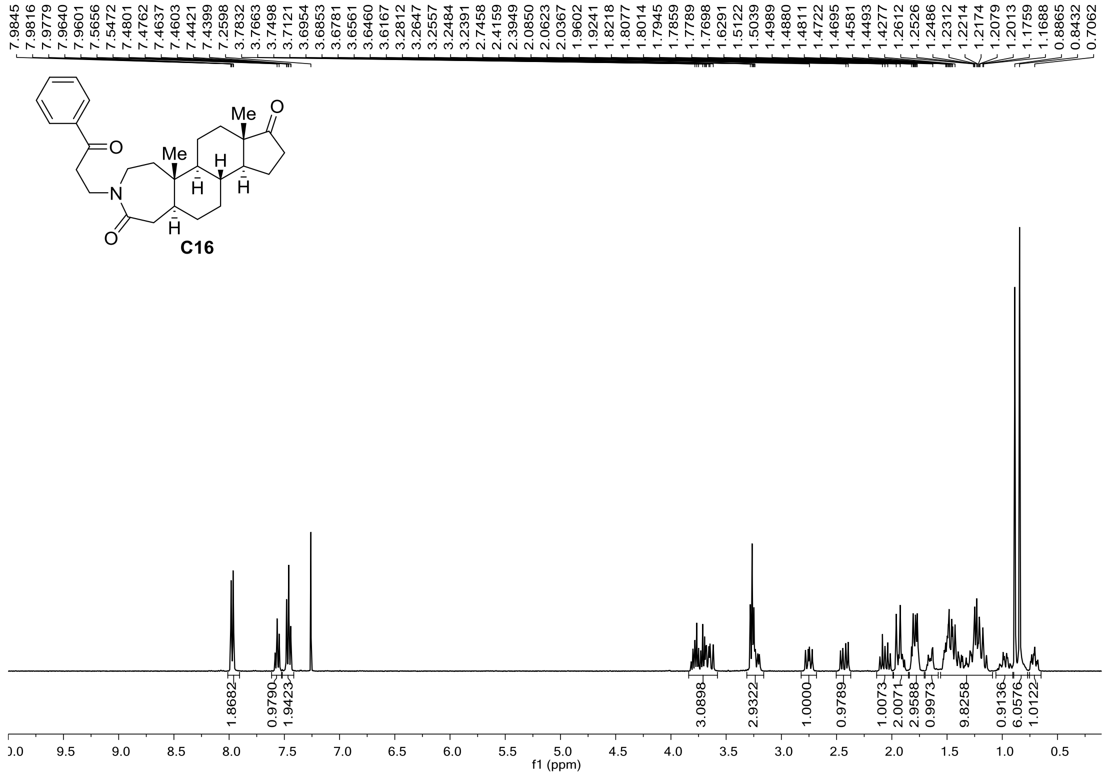

Supplementary Figure 187. ^{13}C NMR spectrum in $CDCl_3$, 101 MHz, of **C16**.

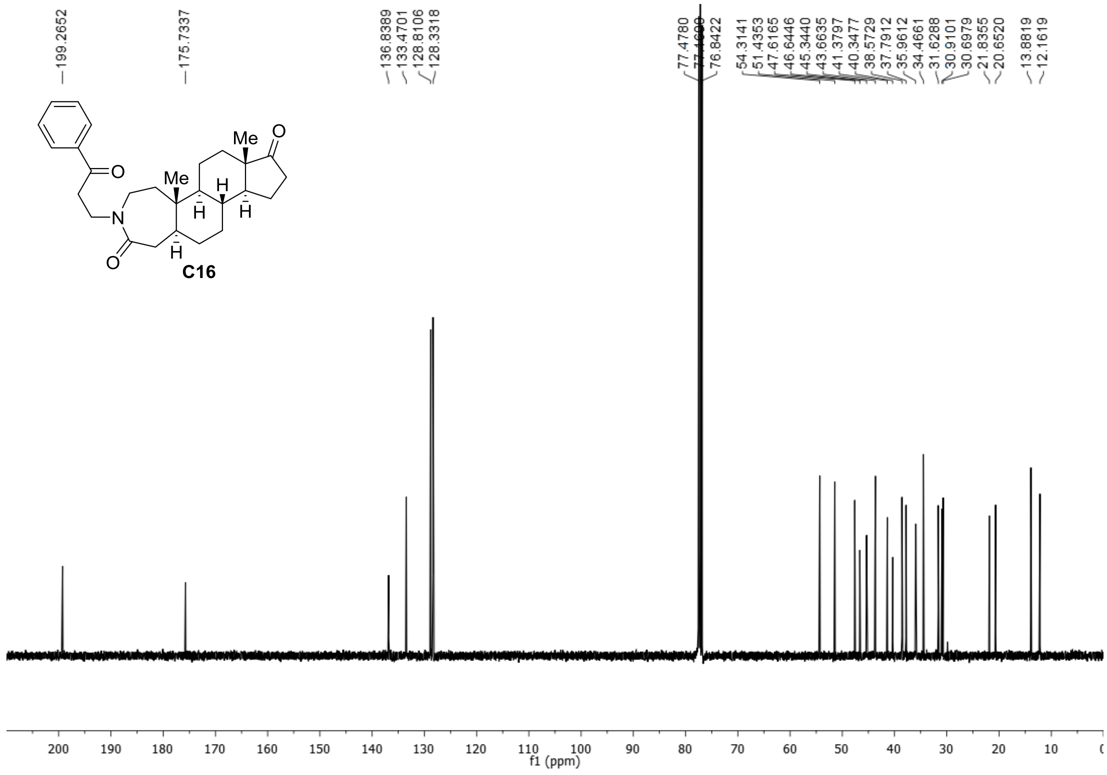

Supplementary Figure 188. ^{1}H NMR spectrum in $CDCl_3$, 400 MHz, of **C17**.

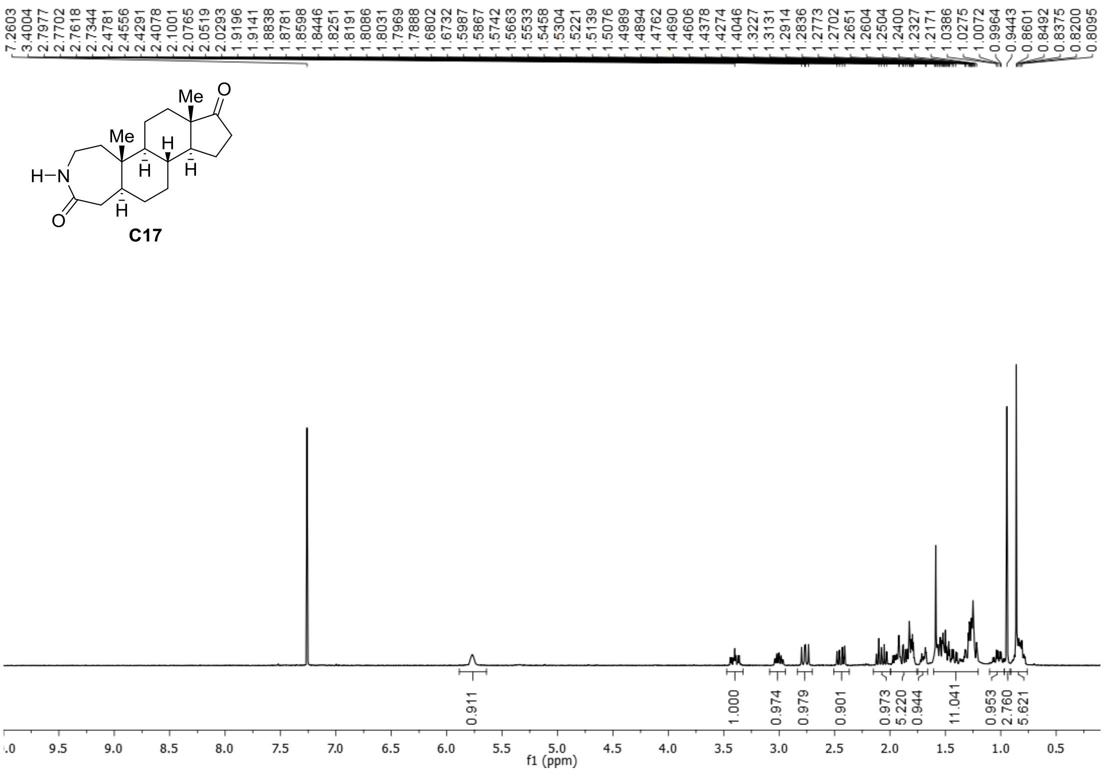

Supplementary Figure 189. ^{13}C NMR spectrum in $CDCl_3$, 151 MHz, of **C17**.

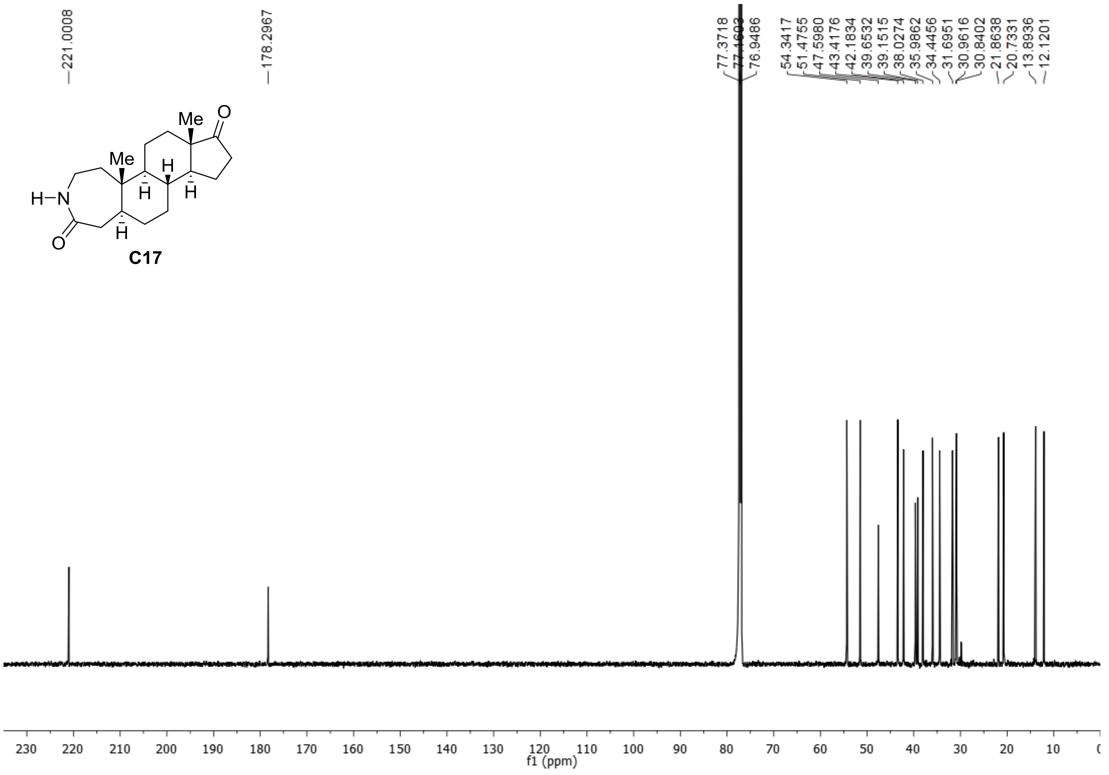

Supplementary Figure 190. ^{1}H NMR spectrum in $CDCl_3$, 400 MHz, of **D2**.

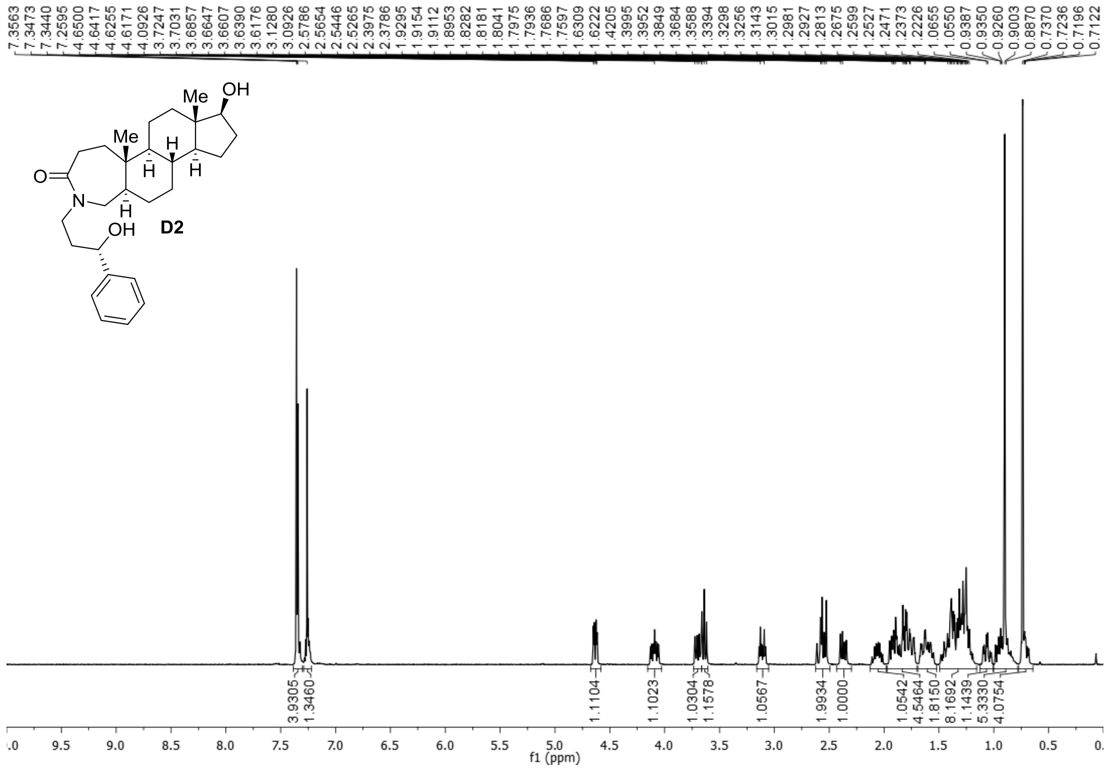

Supplementary Figure 191. ^{13}C NMR spectrum in $CDCl_3$, 101 MHz, of **D2**.

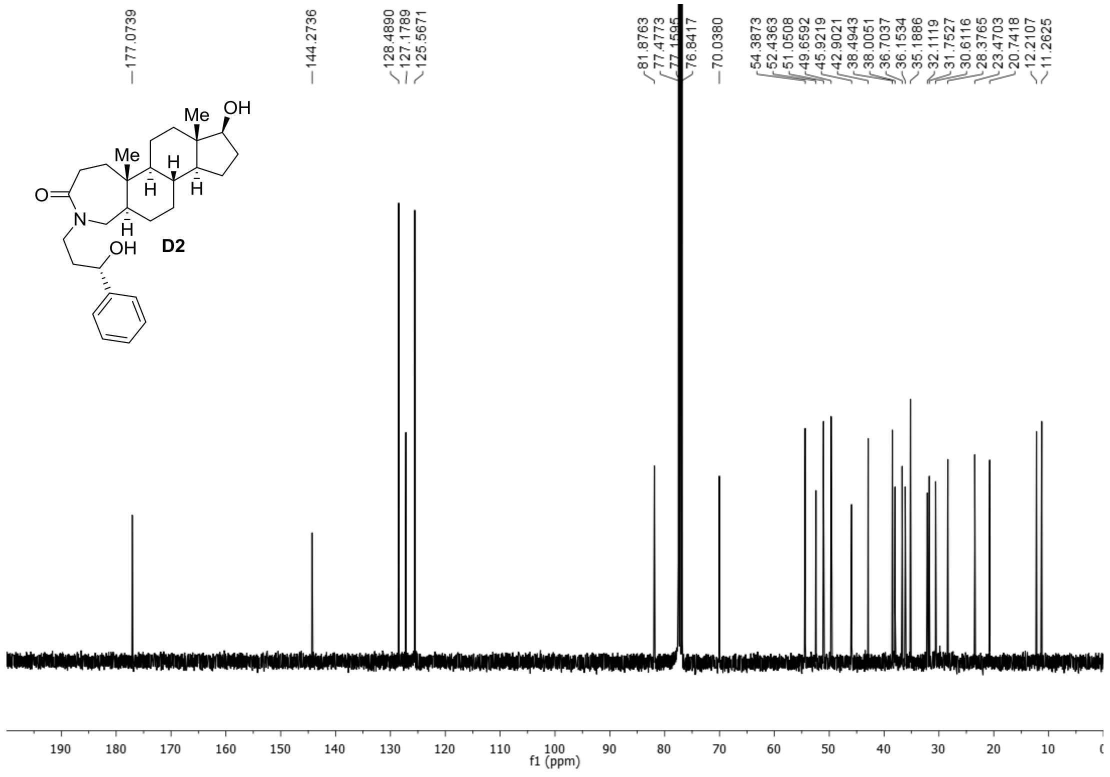

Supplementary Figure 192. ^{13}C NMR (APT) spectrum in $CDCl_3$, 101 MHz, of **D2**.

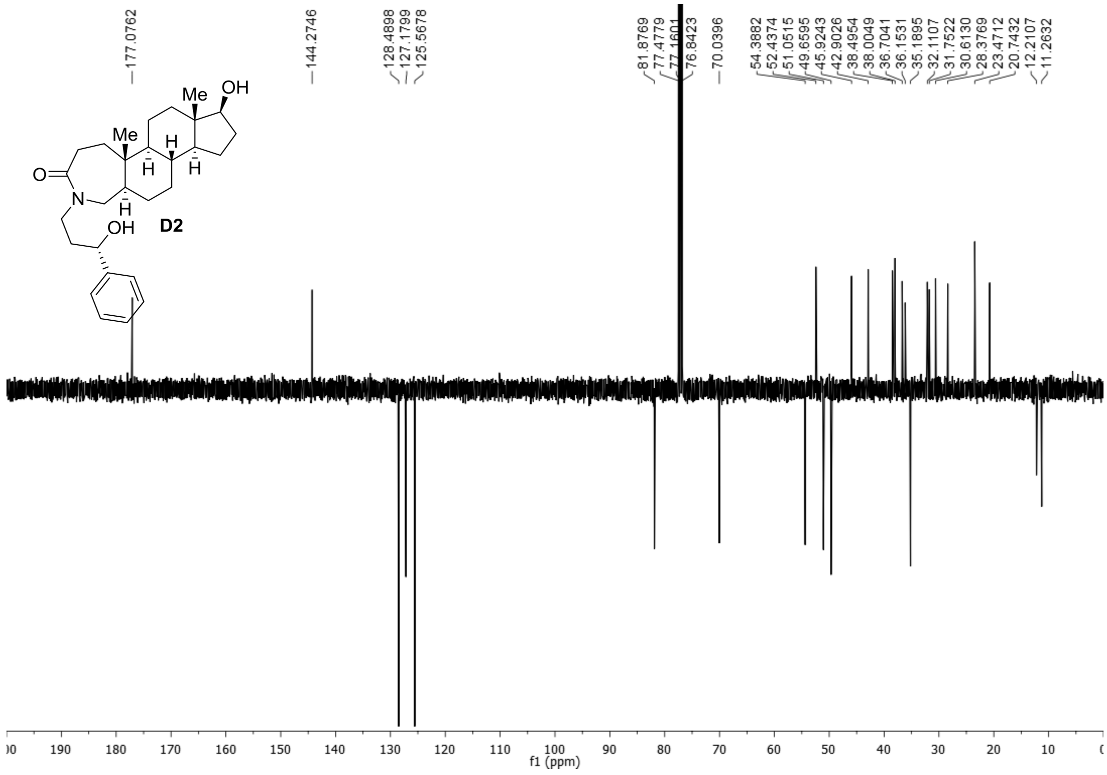

Supplementary Figure 193. 1D NOE spectrum in $CDCl_3$, 400 MHz, of **D2**.

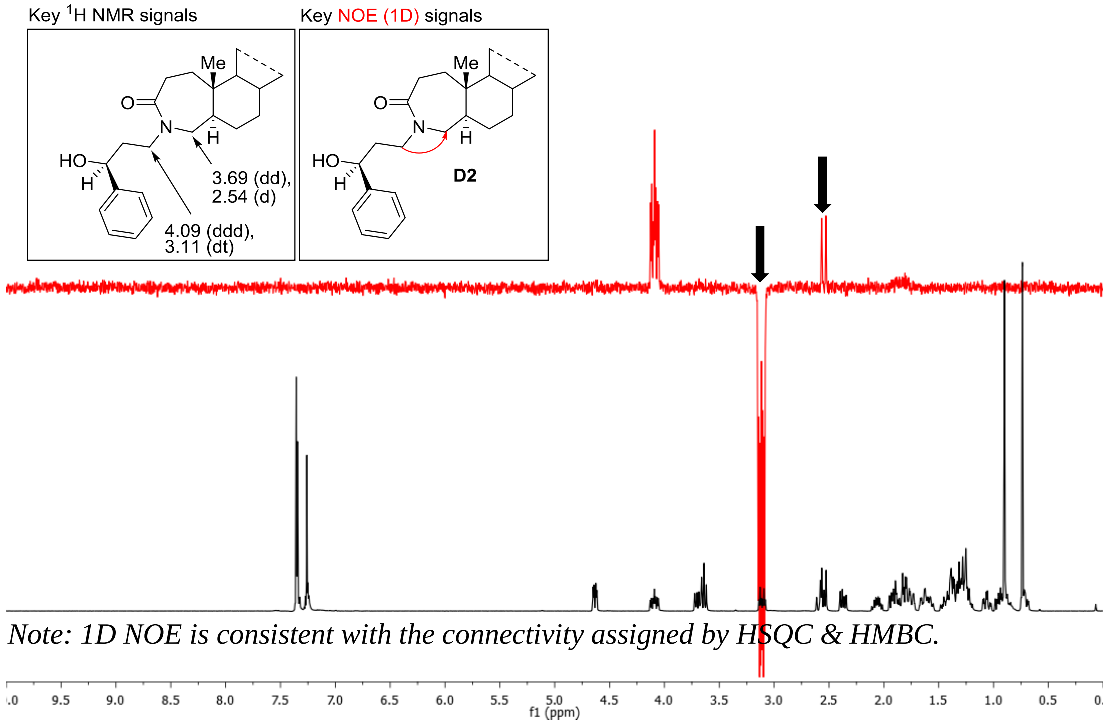

Note: 1D NOE is consistent with the connectivity assigned by HSQC & HMBC.

Supplementary Figure 194. HSQC spectrum of **D2**.

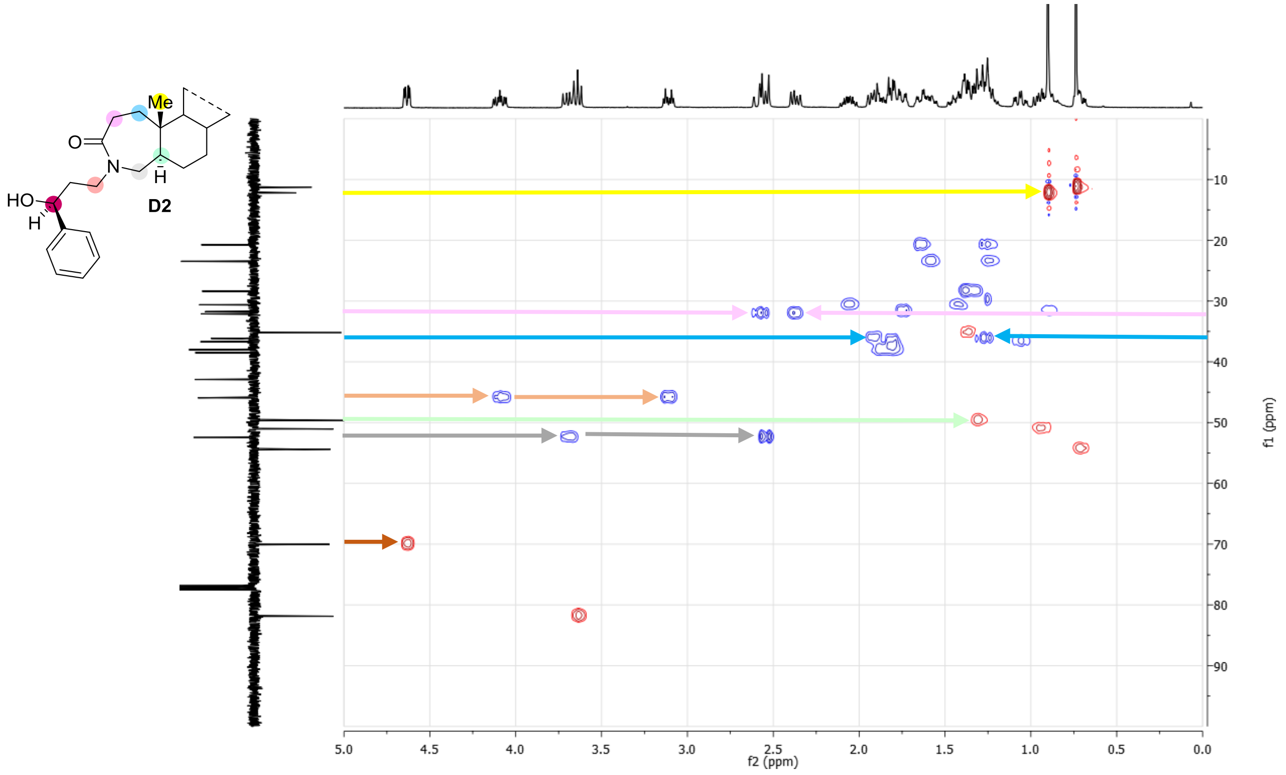

Supplementary Figure 195. HMBC spectrum of **D2**.

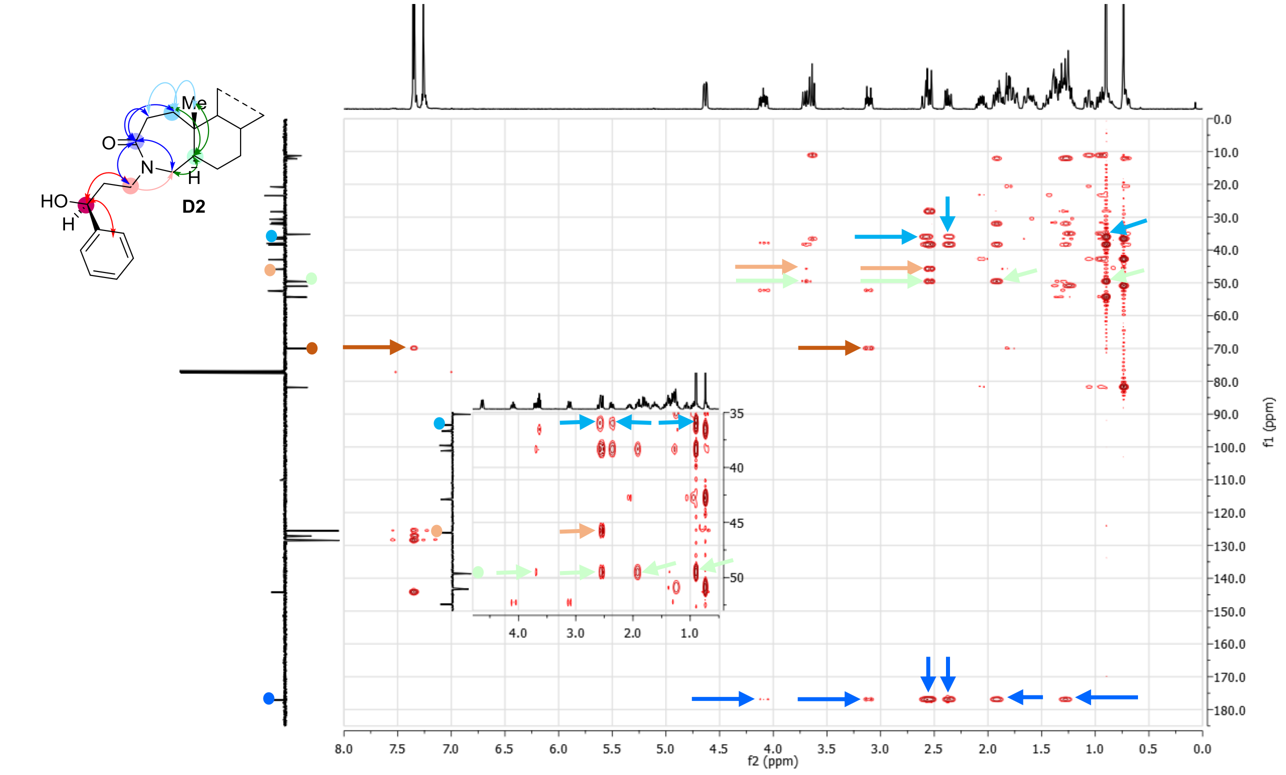

Supplementary Figure 196. ^{1}H NMR spectrum in $CDCl_3$, 400 MHz, of **D3**.

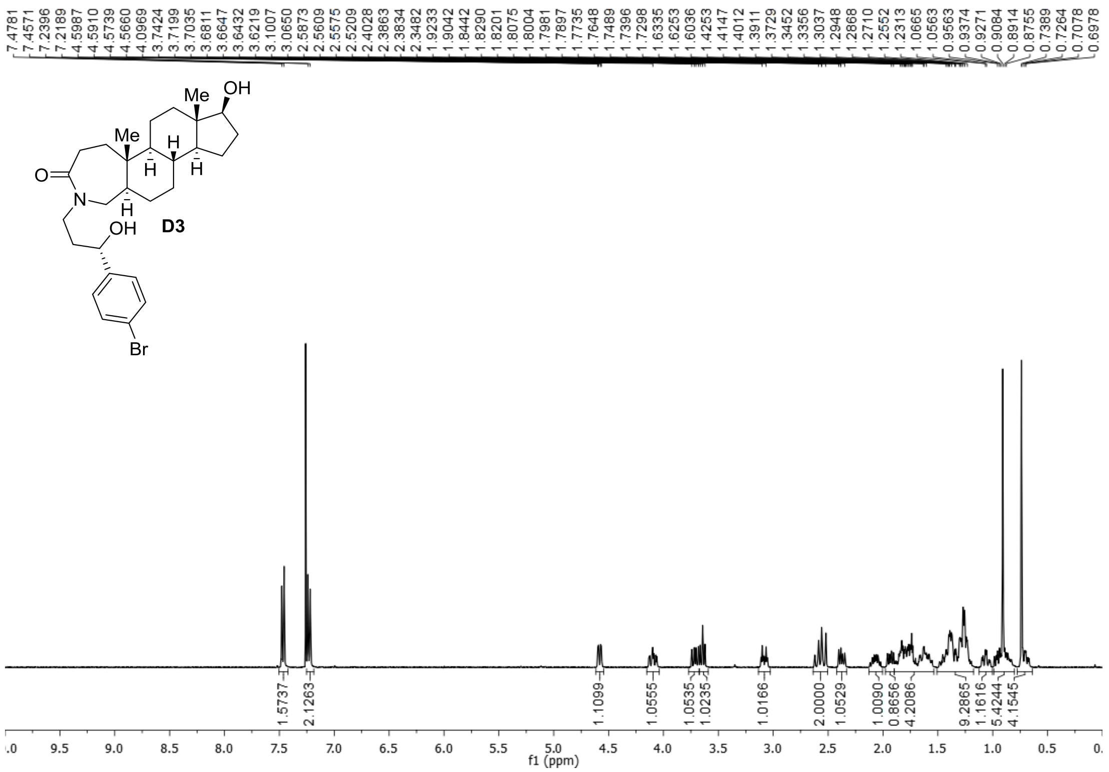

Supplementary Figure 197. ^{13}C NMR spectrum in $CDCl_3$, 101 MHz, of **D3.**

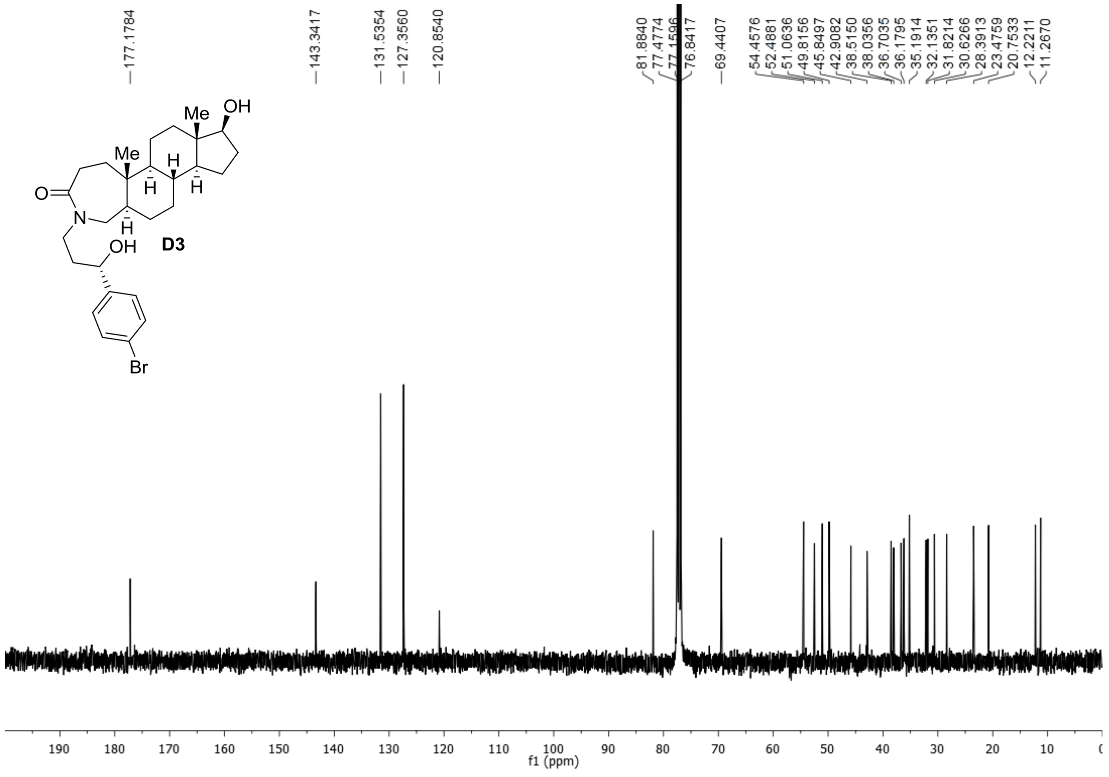

Supplementary Figure 198. ^{1}H NMR spectrum in $CDCl_3$, 400 MHz, of **D4**.

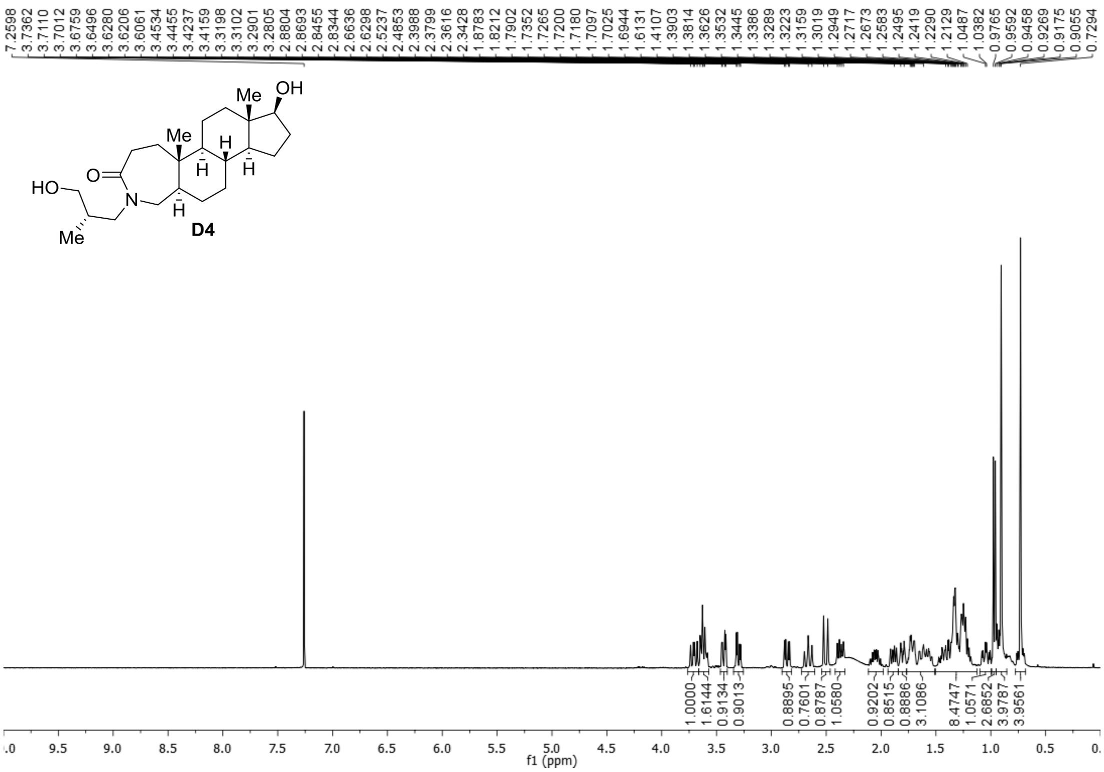

Supplementary Figure 199. ^{13}C NMR spectrum in $CDCl_3$, 126 MHz, of **D4.**

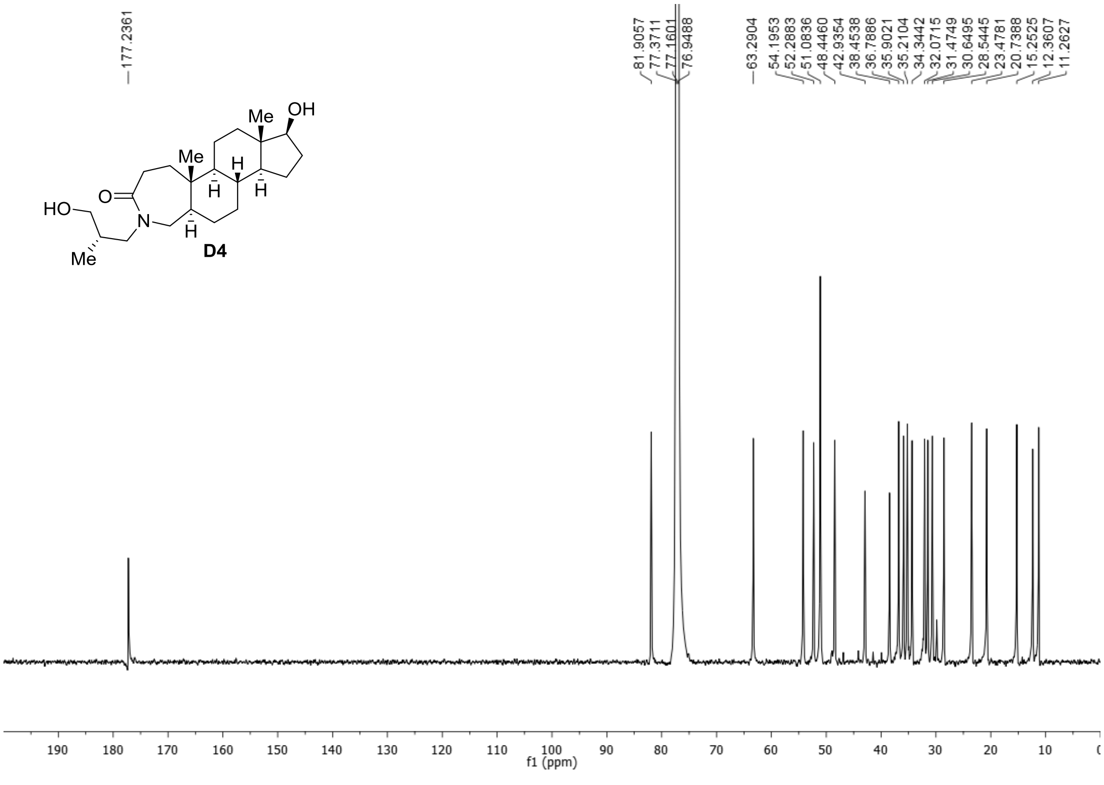

Supplementary Figure 200. ^{1}H NMR spectrum in $CDCl_3$, 400 MHz, of **D5**.

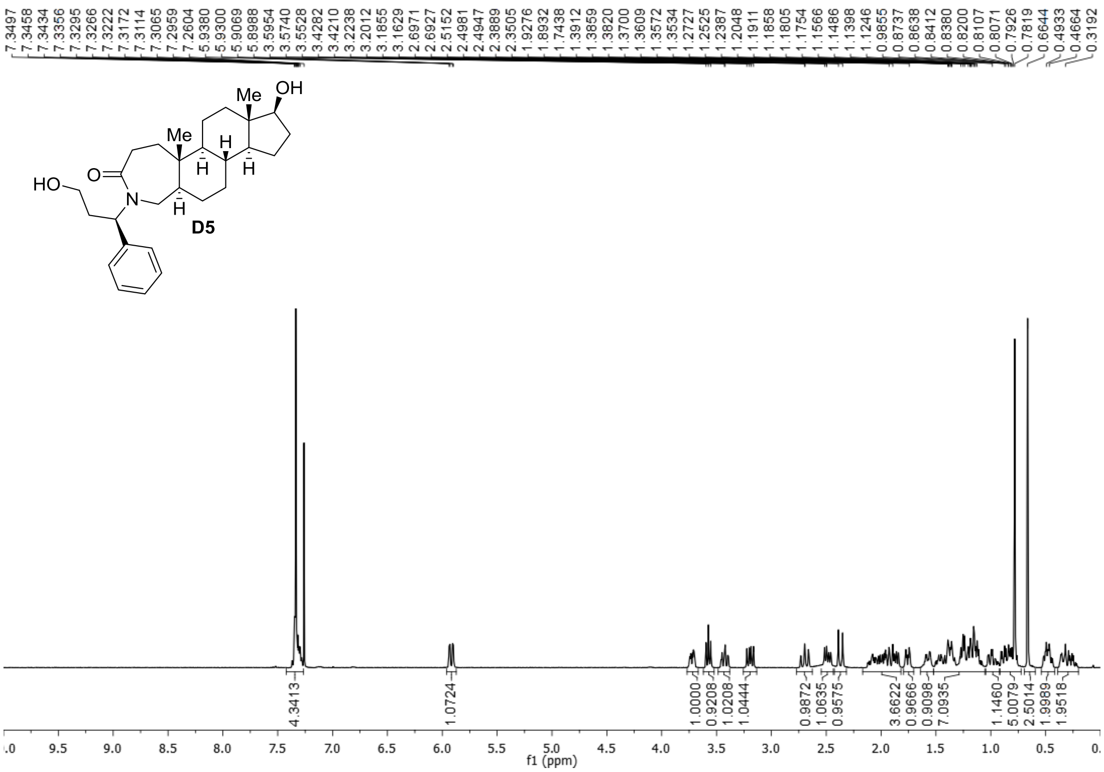

Supplementary Figure 201. ^{13}C NMR spectrum in $CDCl_3$, 101 MHz, of **D5**.

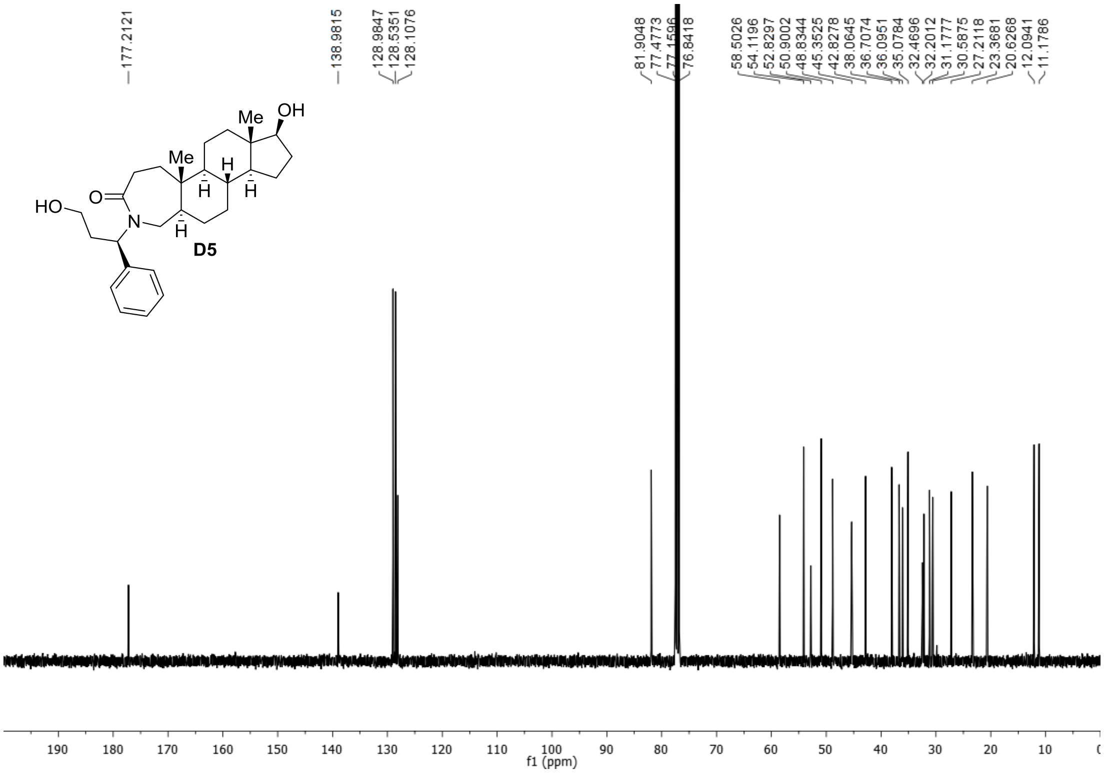

Supplementary Figure 201. ^{13}C NMR (APT) spectrum in $CDCl_3$, 101 MHz, of **D5**.

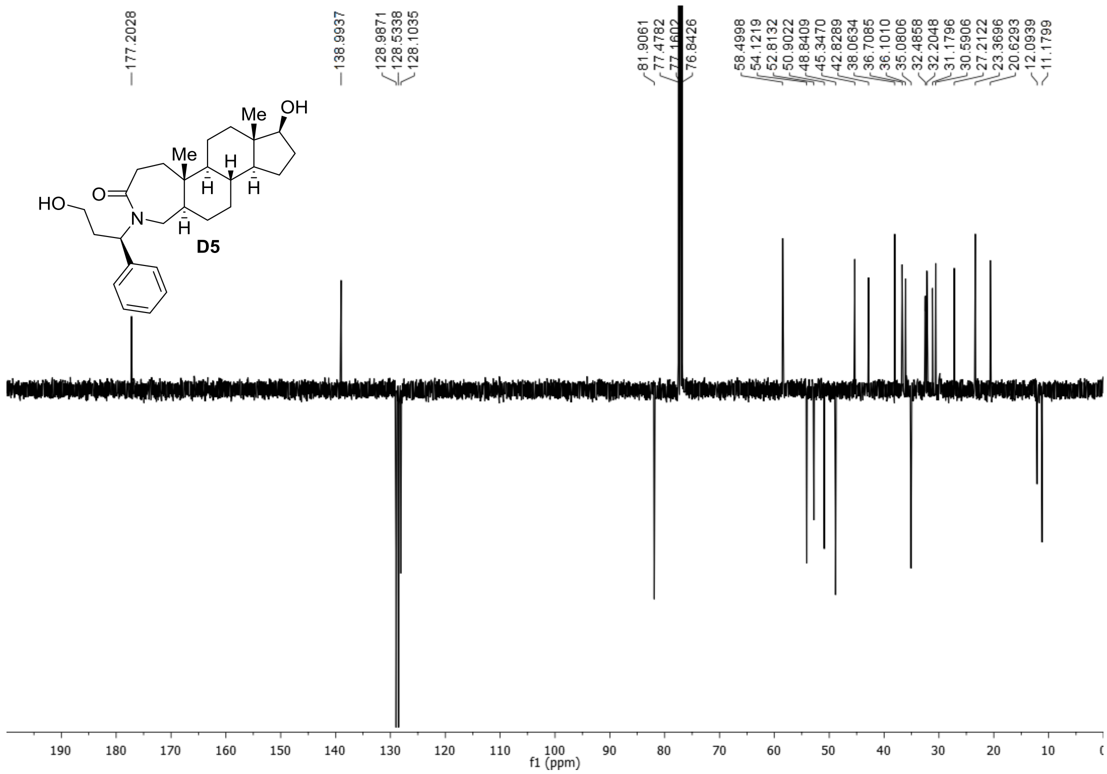

Supplementary Figure 202. 1D NOE spectrum in $CDCl_3$, 400 MHz, of **D5**.

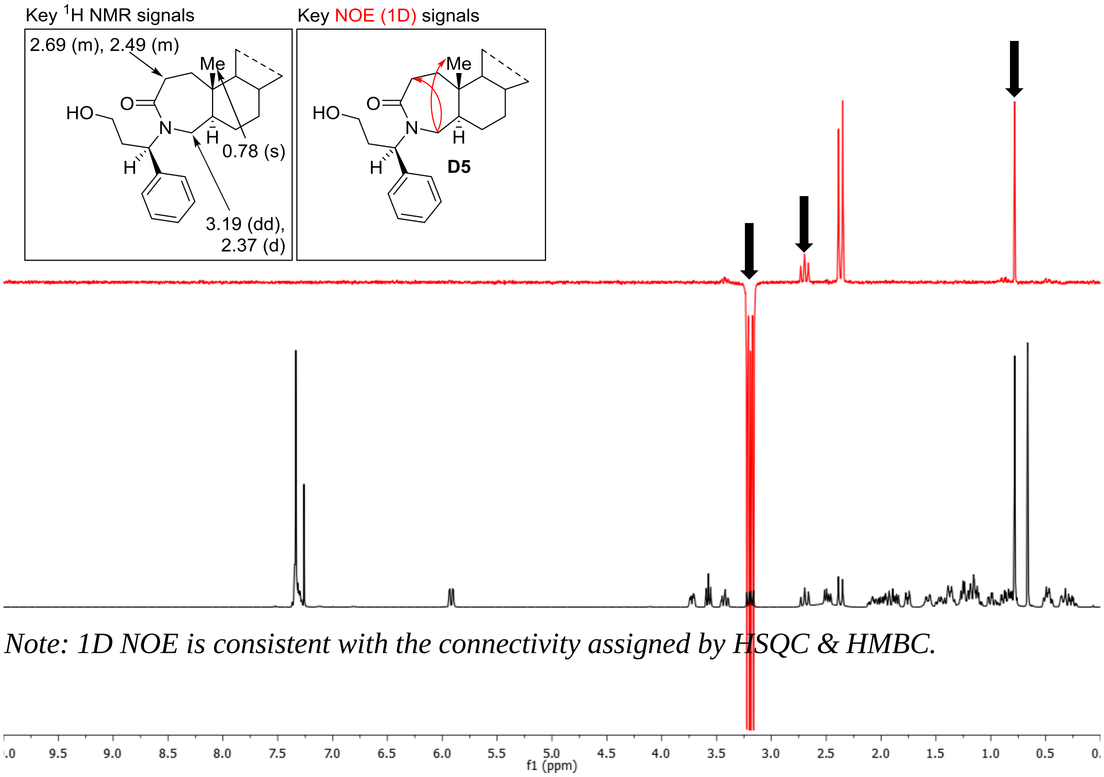

Note: 1D NOE is consistent with the connectivity assigned by HSQC & HMBC.

Supplementary Figure 203. 1D NOE spectrum in $CDCl_3$, 400 MHz, of **D5**.

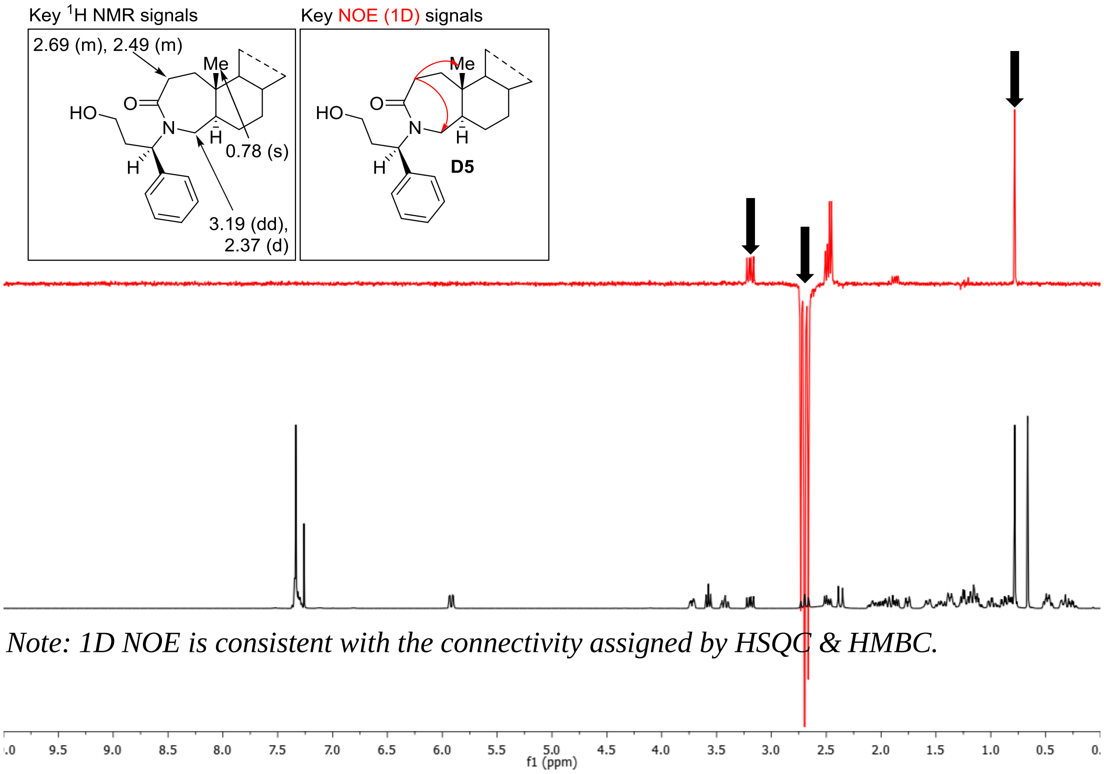

Note: 1D NOE is consistent with the connectivity assigned by HSQC & HMBC.

Supplementary Figure 204. HSQC spectrum of **D5**.

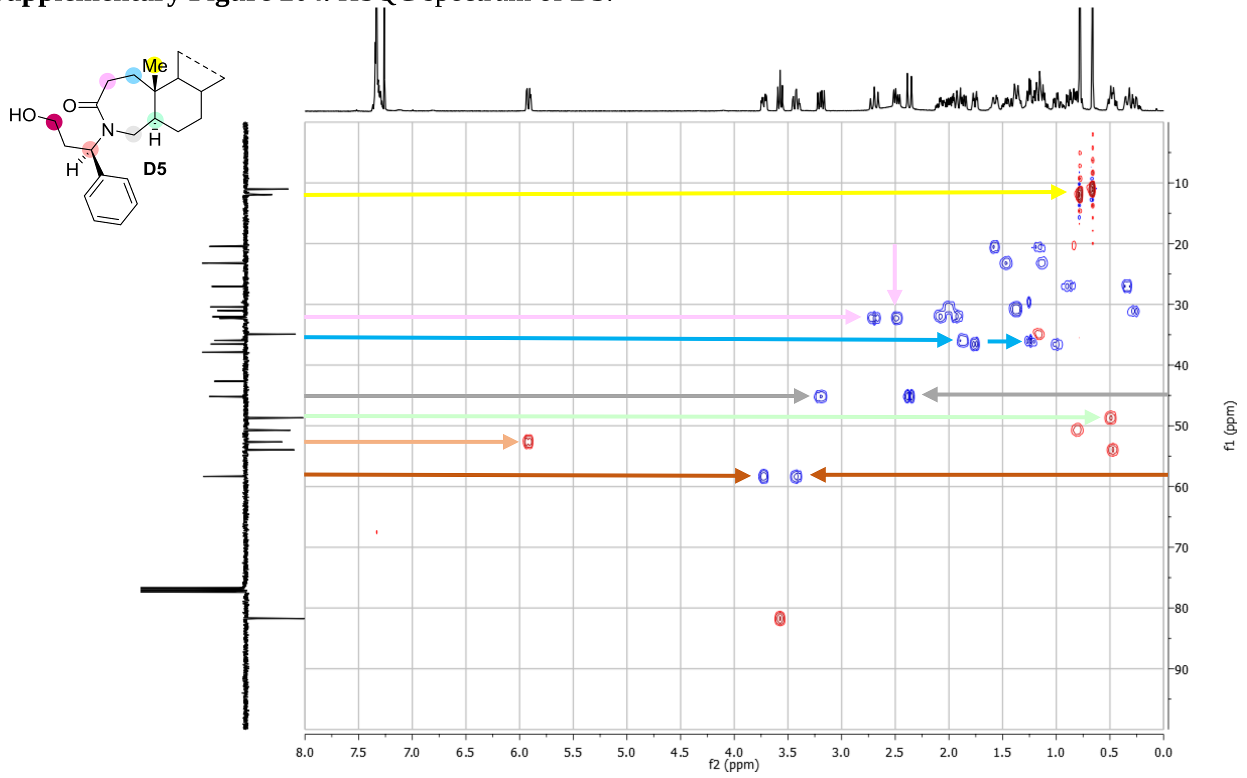

Supplementary Figure 205. HMBC spectrum of **D5**.

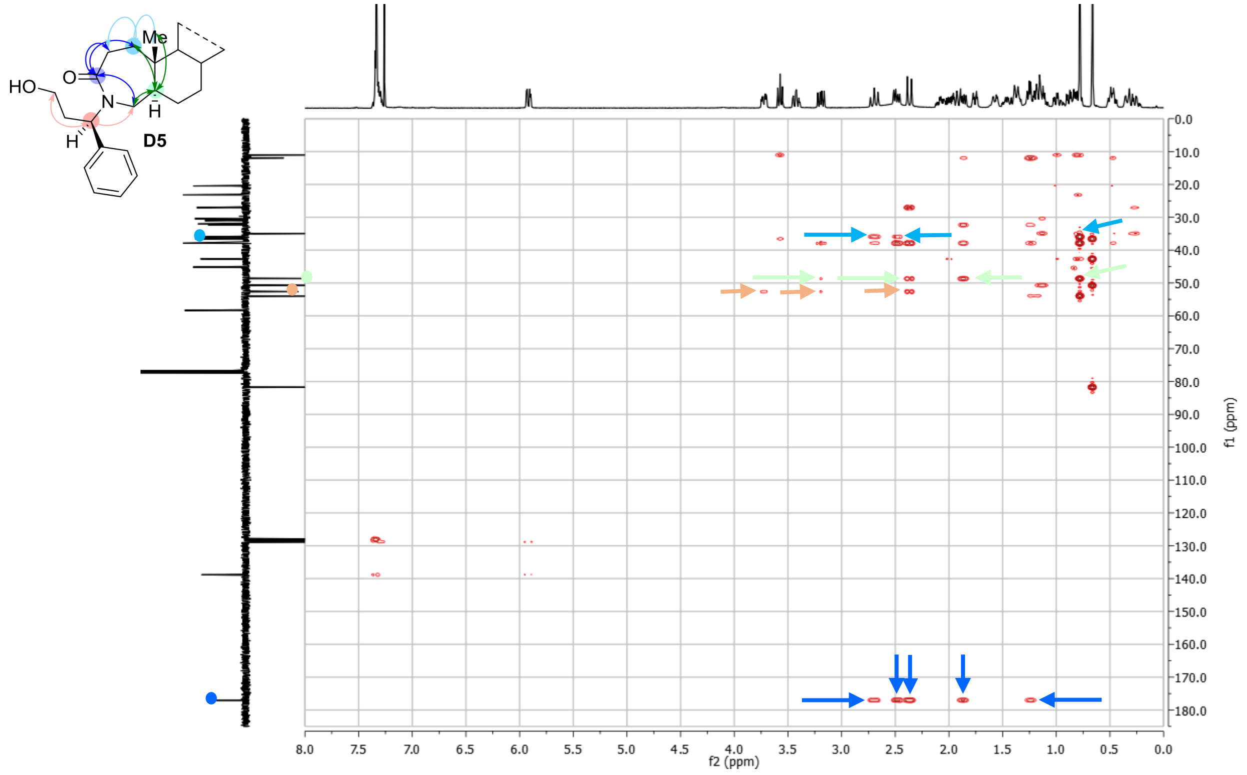

Supplementary Figure 206. ^{1}H NMR spectrum in $CDCl_3$, 400 MHz, of **D6**.

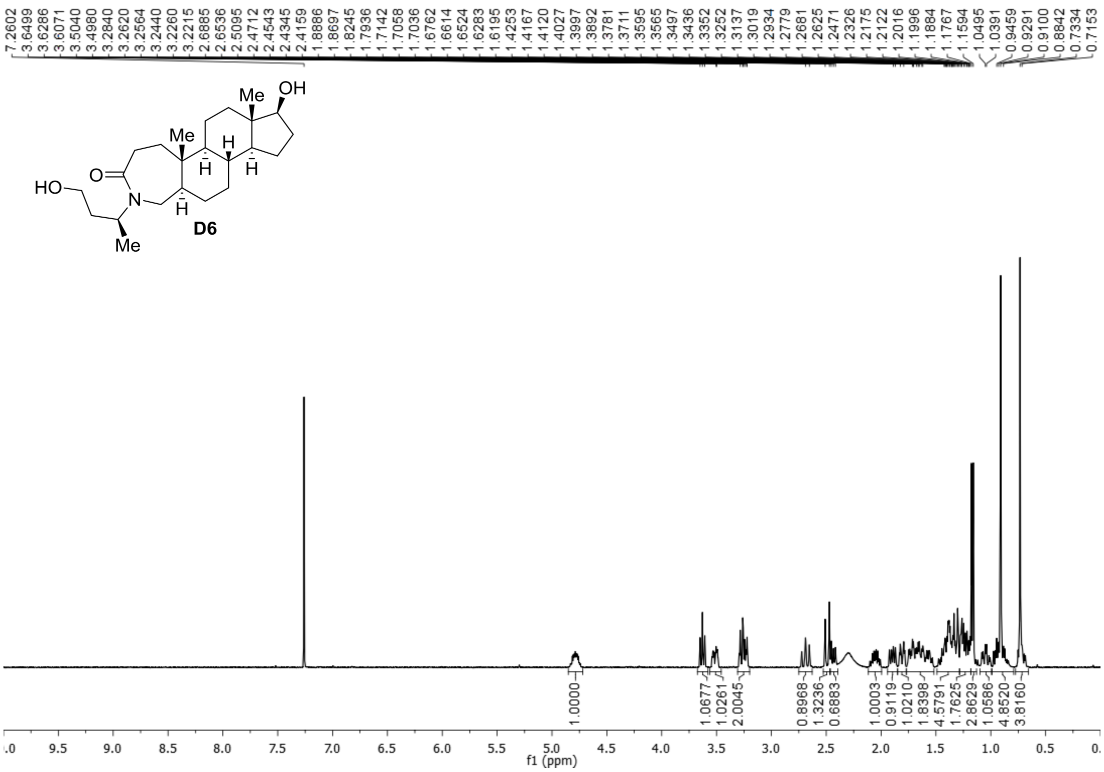

Supplementary Figure 207. ^{13}C NMR spectrum in $CDCl_3$, 101 MHz, of **D6**.

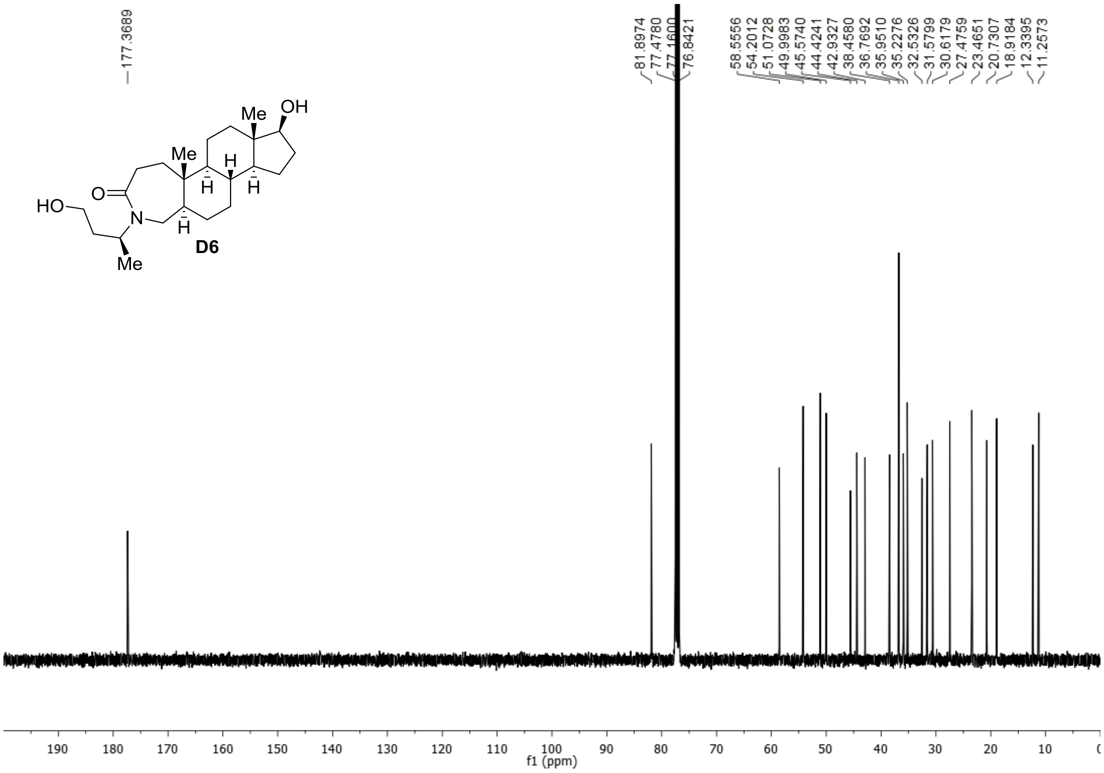

Supplementary Figure 208. ^{13}C NMR (APT) spectrum in $CDCl_3$, 101 MHz, of **D6**.

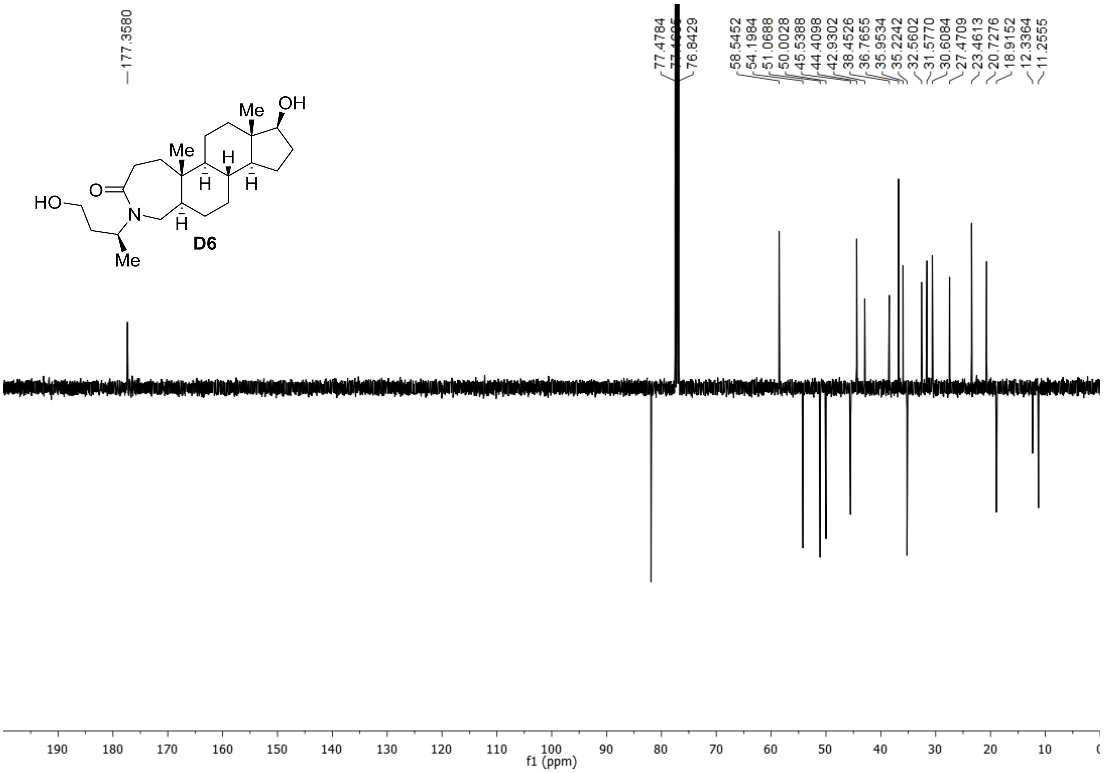

Supplementary Figure 209. 1D NOE spectrum in $CDCl_3$, 400 MHz, of **D6**.

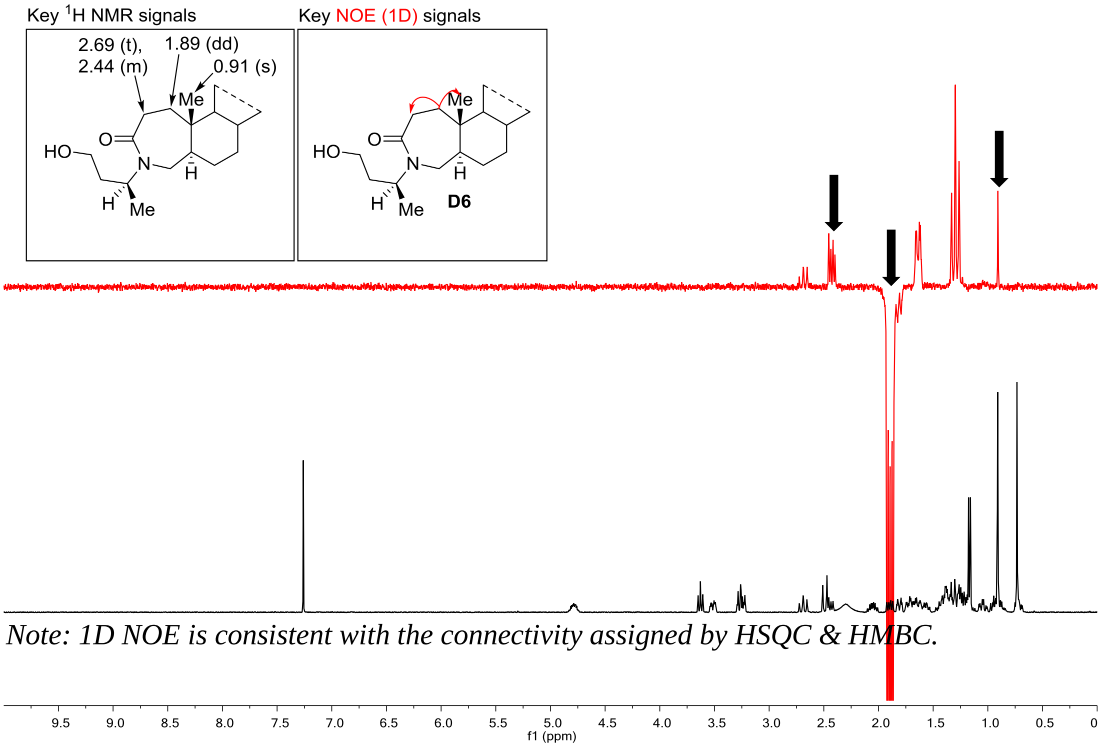

Supplementary Figure 210. 1D NOE spectrum in $CDCl_3$, 400 MHz, of **D6**.

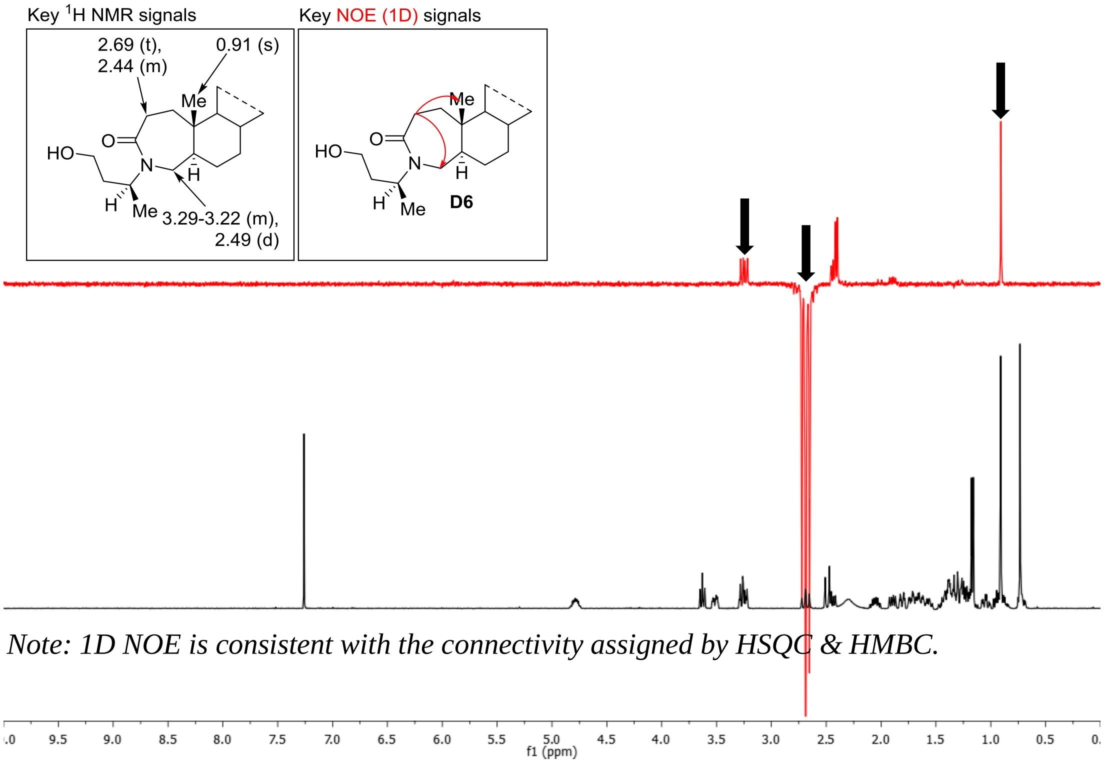

Supplementary Figure 210. HSQC spectrum of **D6**.

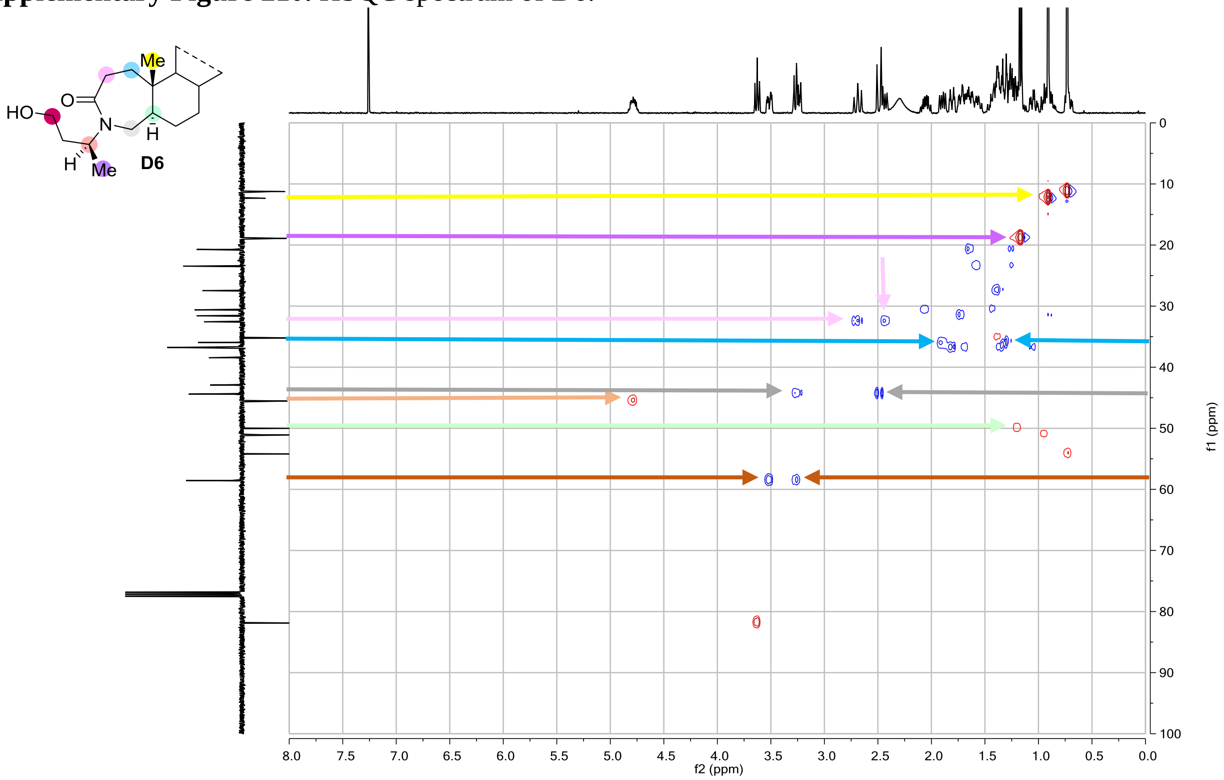

Supplementary Figure 211. HMBC spectrum of **D6**.

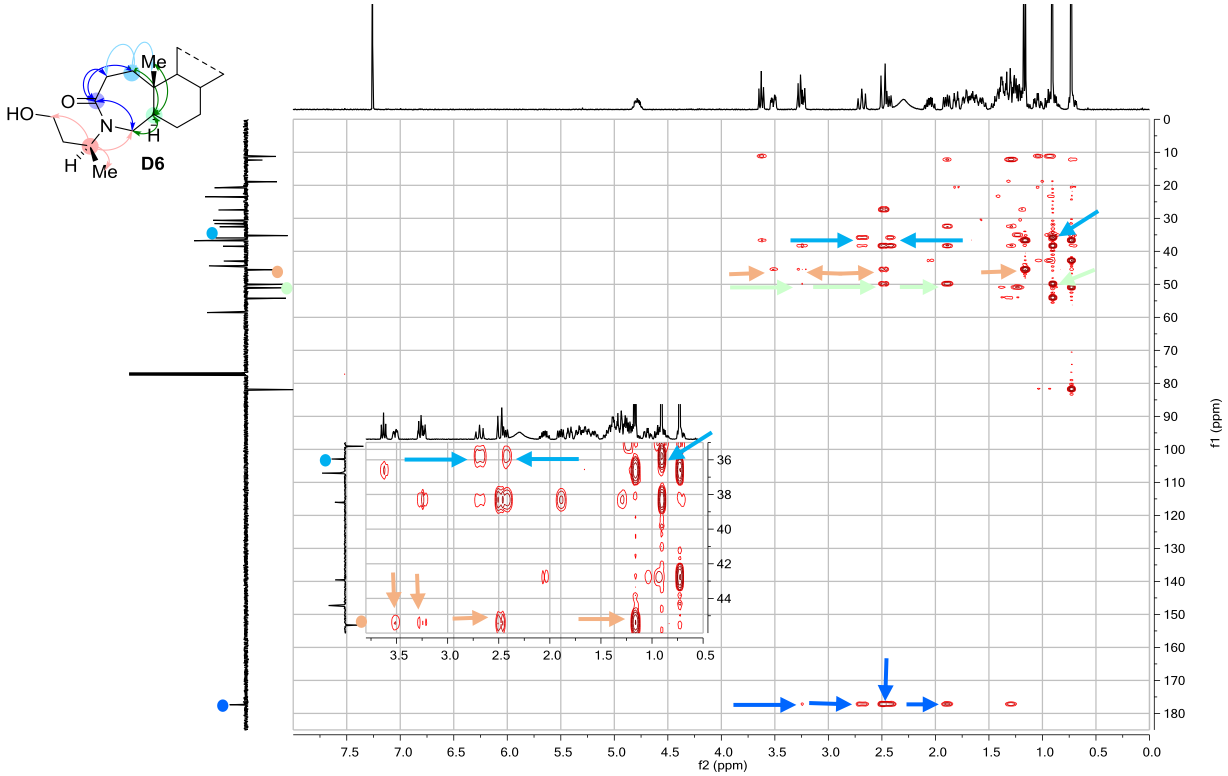

Supplementary Figure 212. ^{1}H NMR spectrum in $CDCl_3$, 400 MHz, of **D7**.

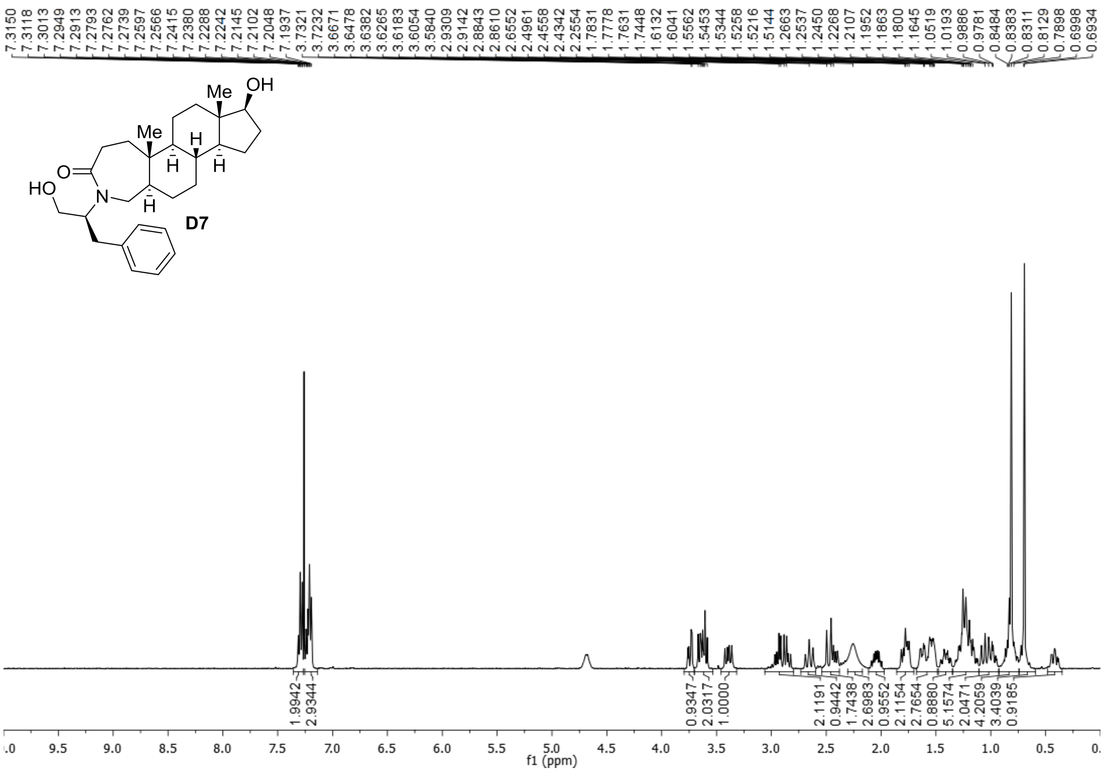

Supplementary Figure 213. ^{13}C NMR spectrum in $CDCl_3$, 151 MHz, of **D7**.

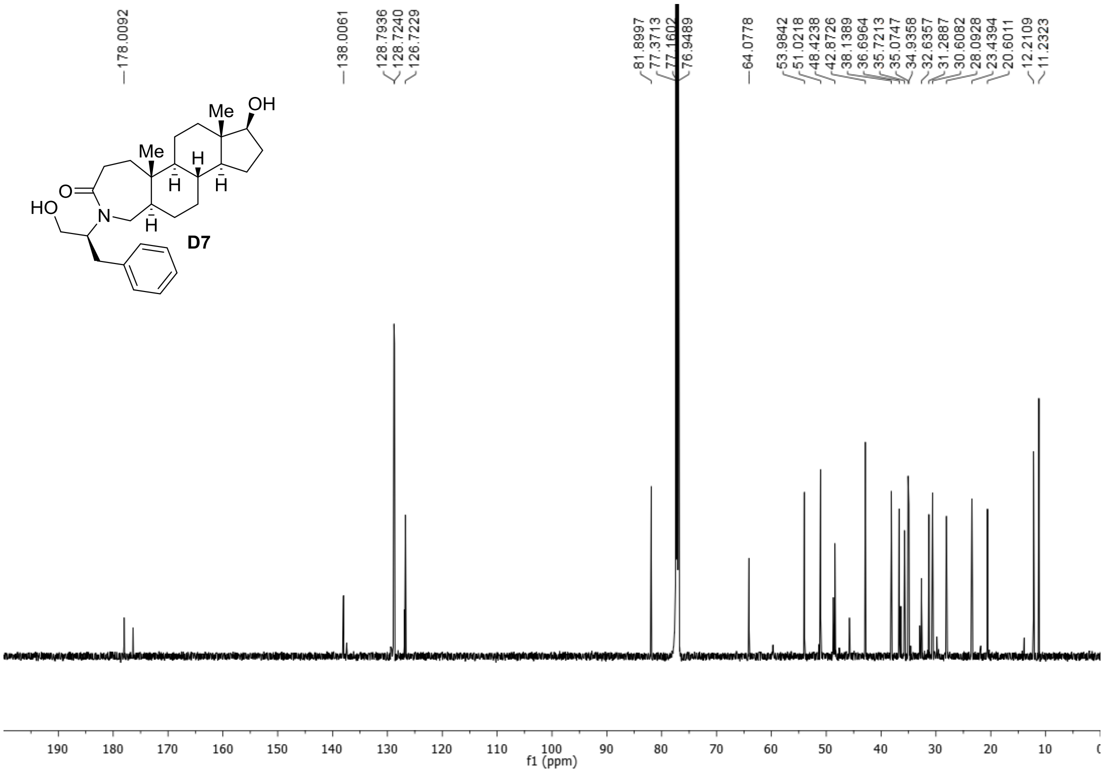

Supplementary Figure 214. ^{1}H NMR spectrum in $CDCl_3$, 400 MHz, of **D8**.

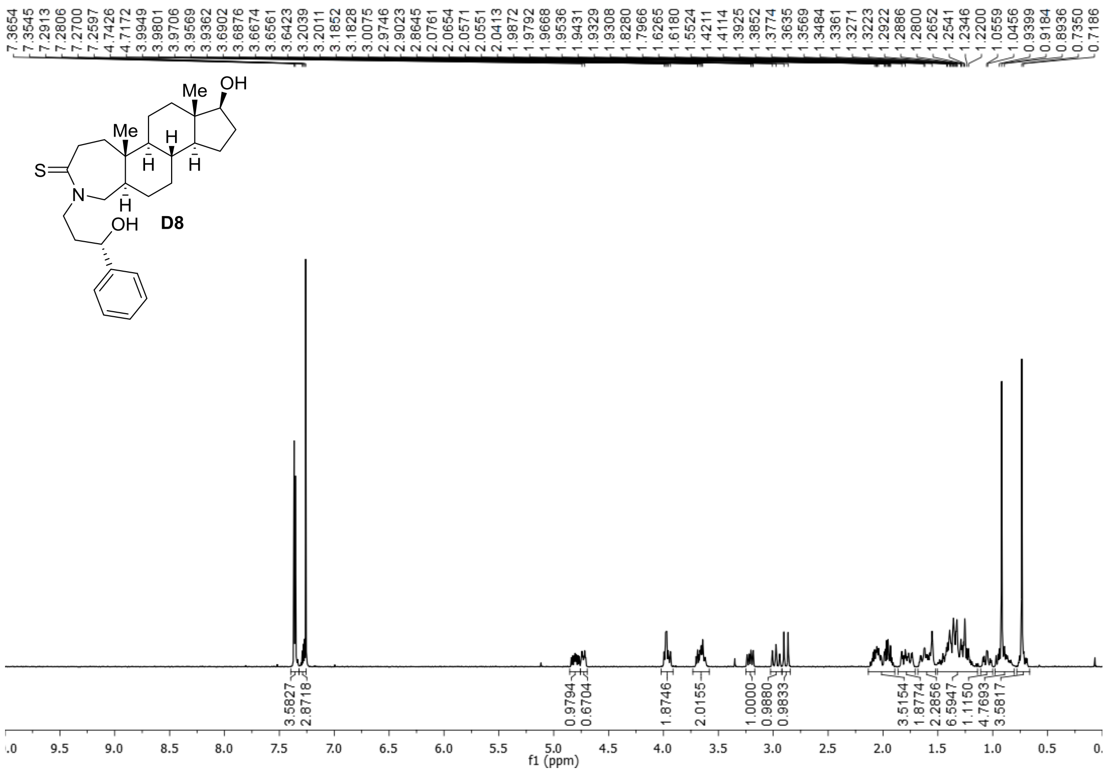

Supplementary Figure 215. ^{13}C NMR spectrum in $CDCl_3$, 151 MHz, of **D8**.

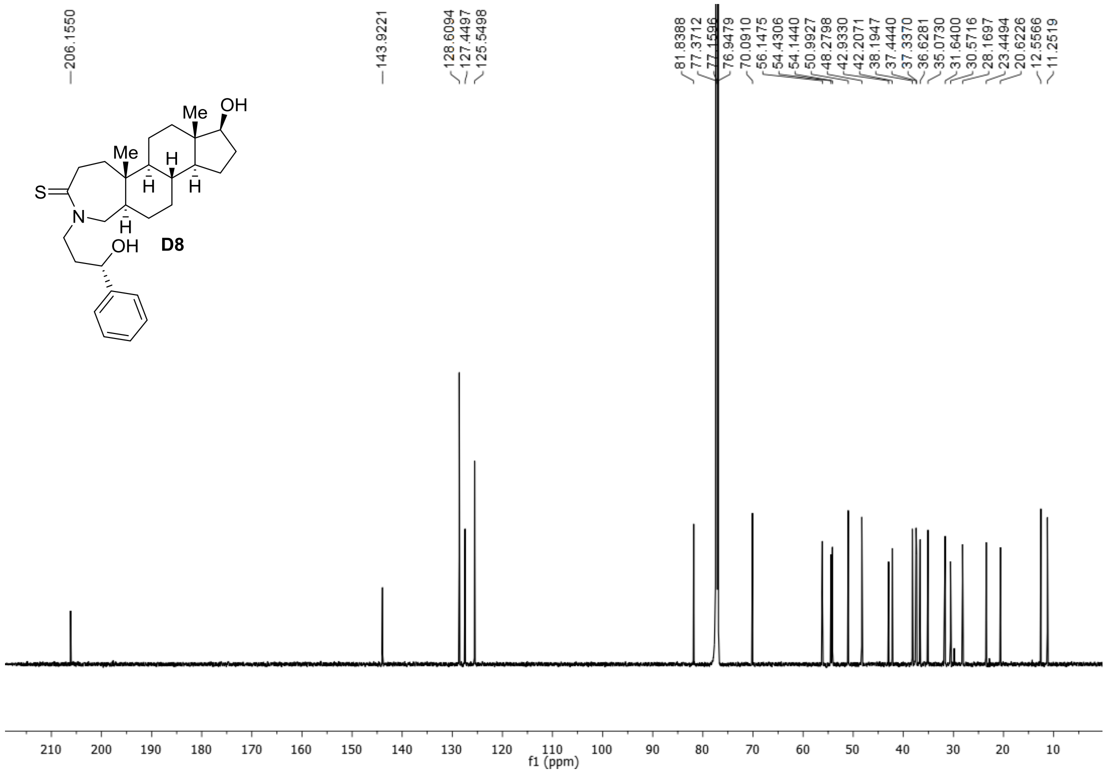

Supplementary Figure 216. ^{1}H NMR spectrum in $CDCl_3$, 400 MHz, of **D9**.

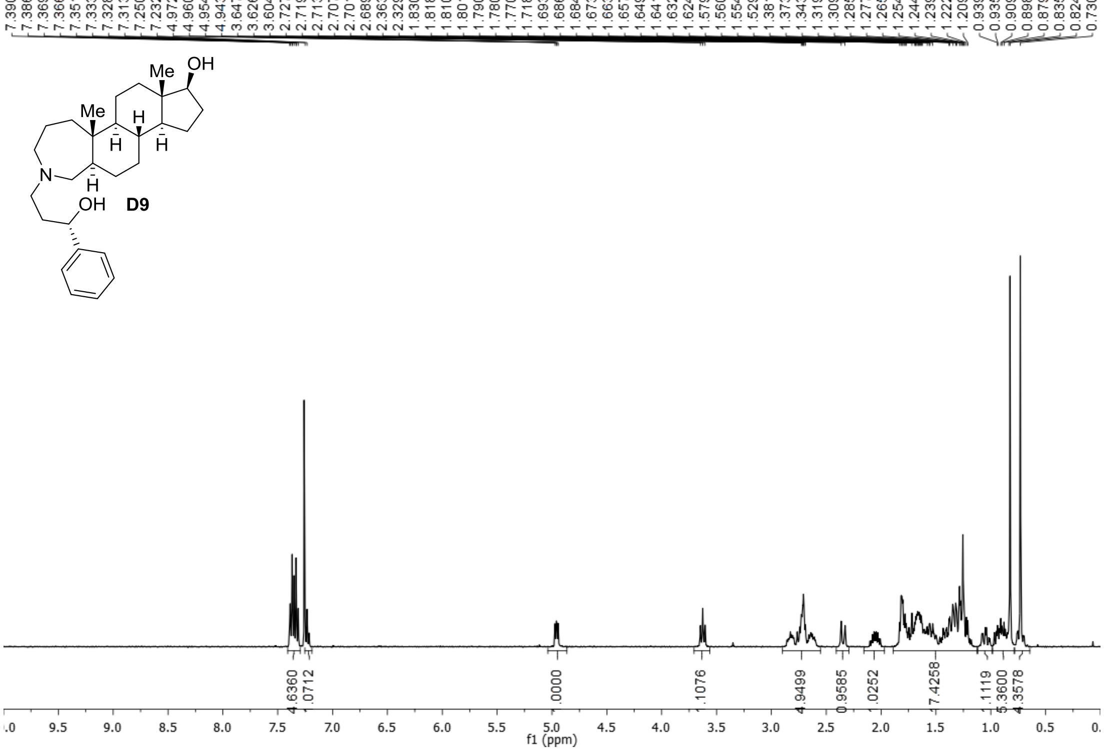

Supplementary Figure 217. ^{13}C NMR (APT) spectrum in $CDCl_3$, 101 MHz, of **D9**.

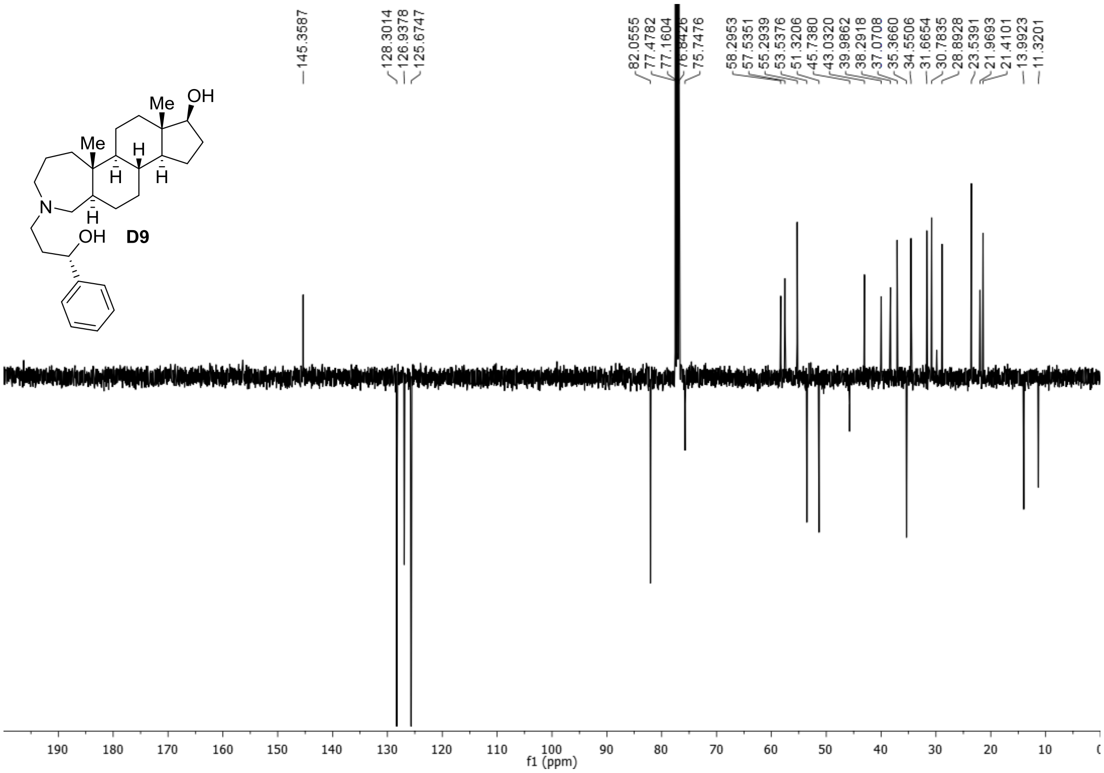

Supplementary Figure 218. ^{1}H NMR spectrum in $CDCl_3$, 400 MHz, of **D10**.

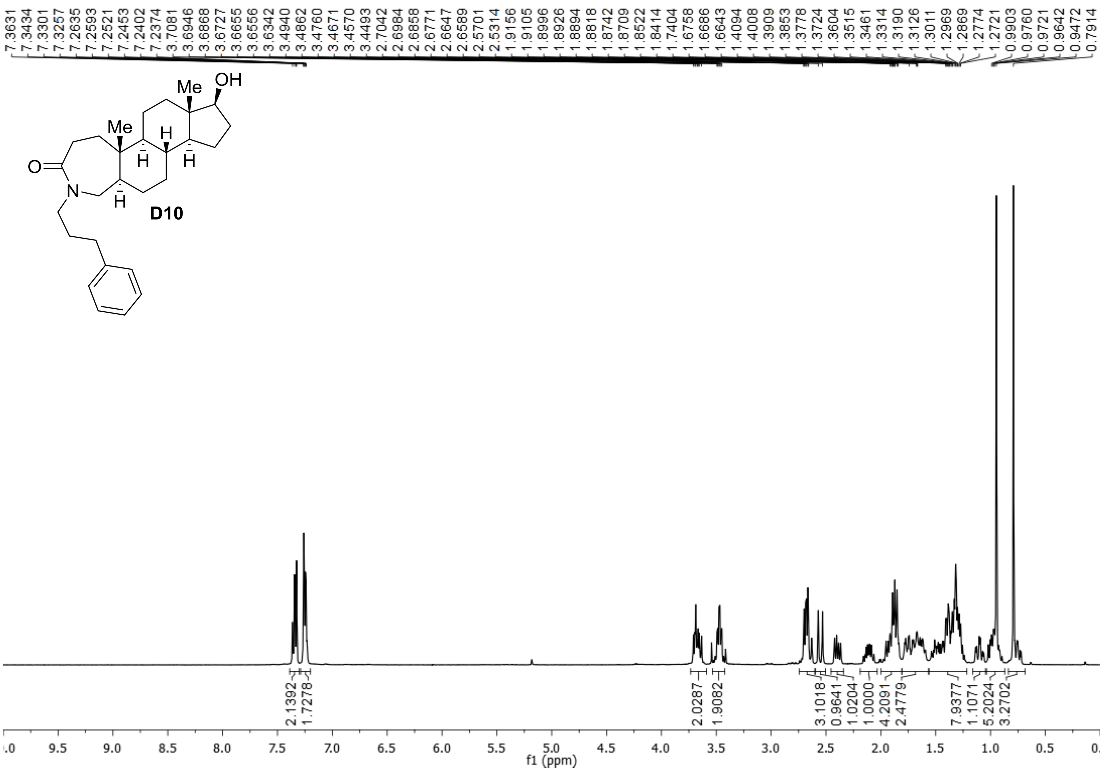

Supplementary Figure 219. ^{13}C NMR (APT) spectrum in $CDCl_3$, 101 MHz, of **D10**.

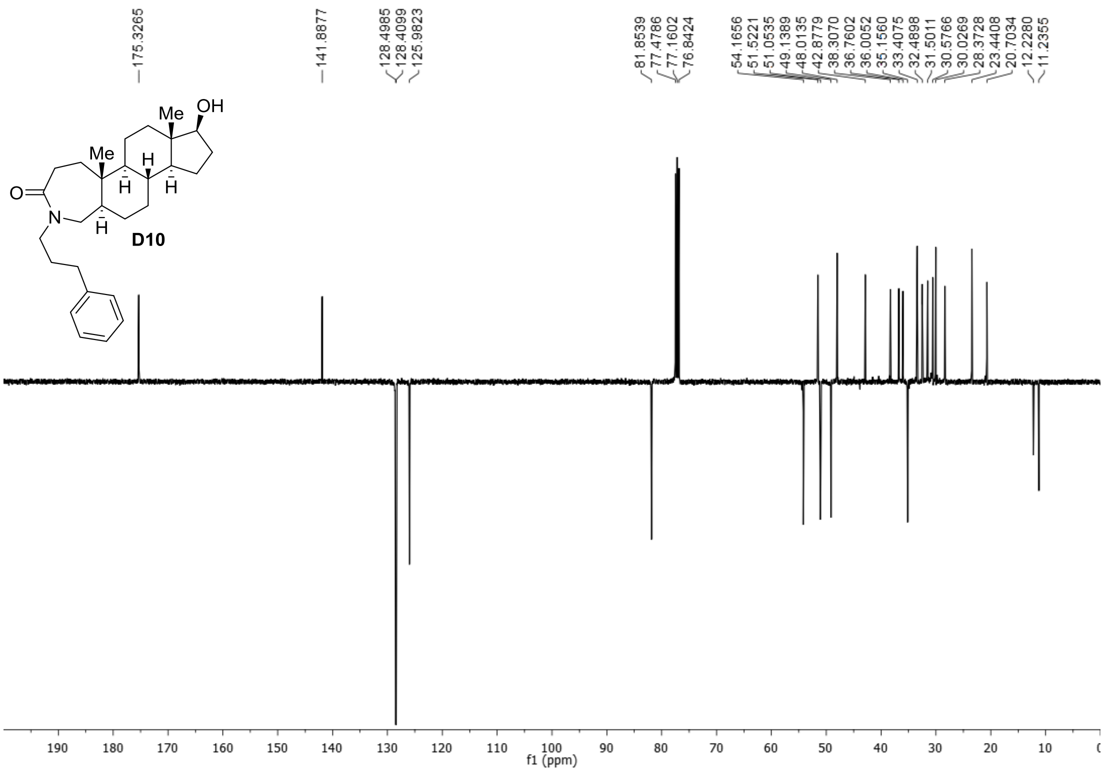

Supplementary Figure 220. ^{1}H NMR spectrum in $CDCl_3$, 400 MHz, of **D11**.

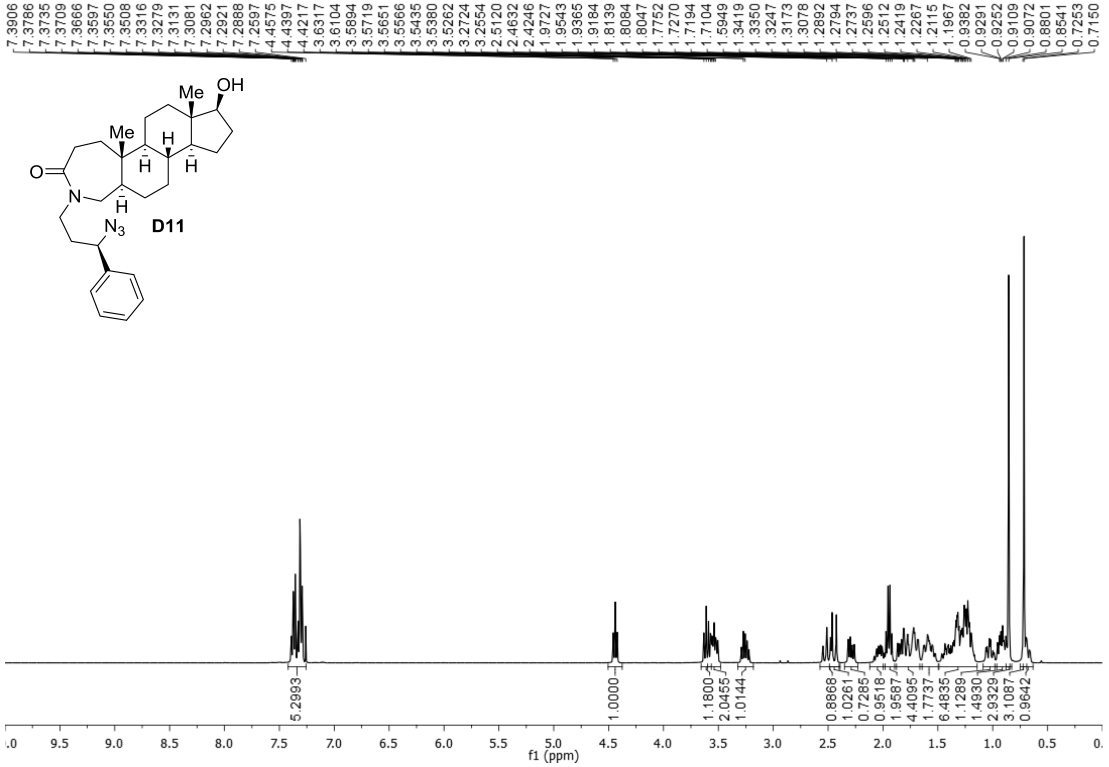

Supplementary Figure 221. ^{13}C NMR spectrum in $CDCl_3$, 101 MHz, of **D11**.

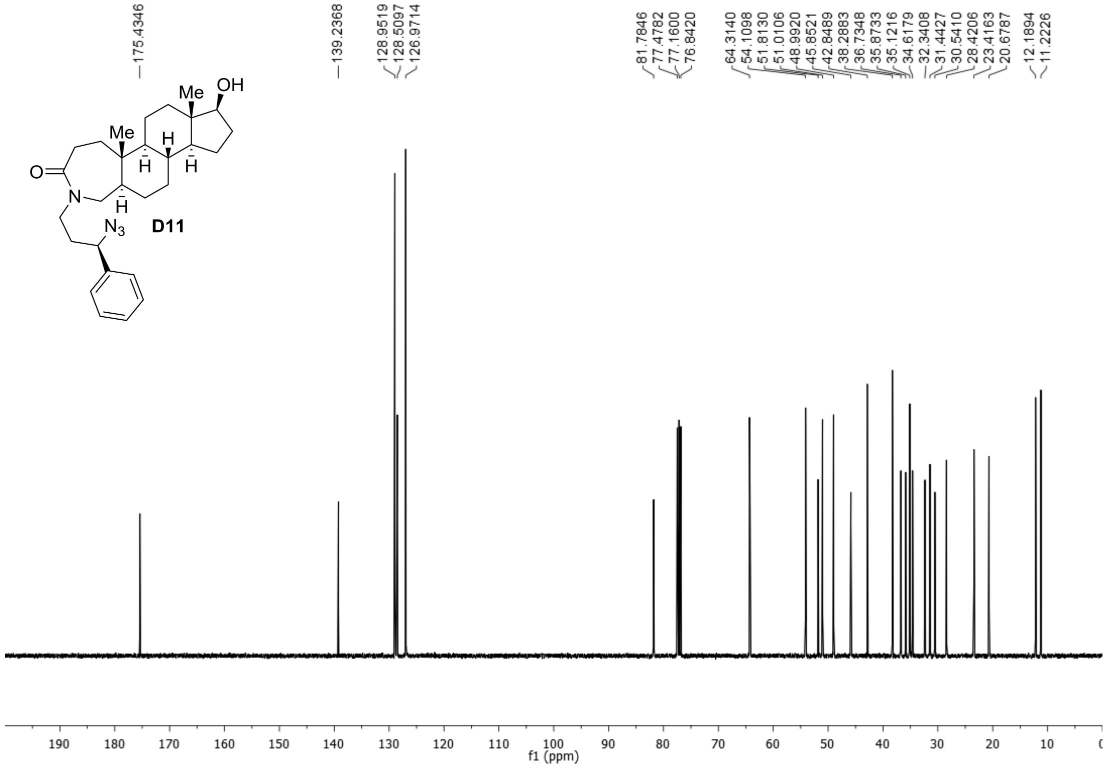

Supplementary Figure 222. ^{1}H NMR spectrum in $CDCl_3$, 400 MHz, of **D12**.

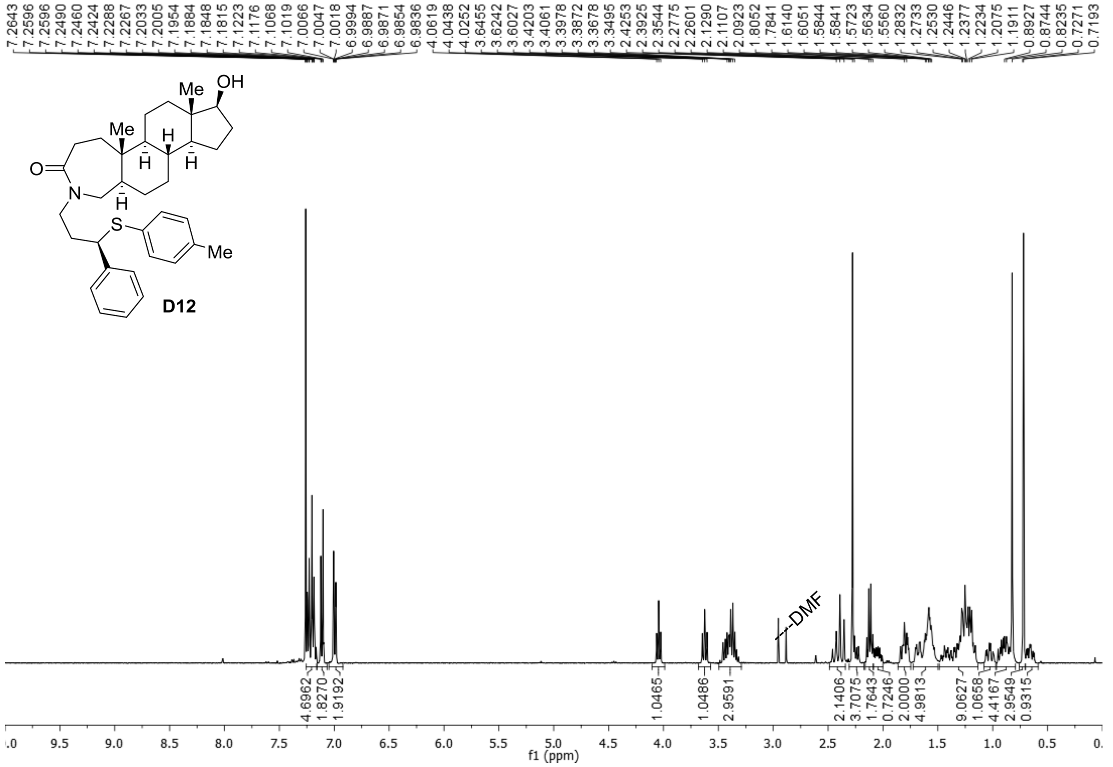

Supplementary Figure 223. ^{13}C NMR spectrum in $CDCl_3$, 101 MHz, of **D12**.

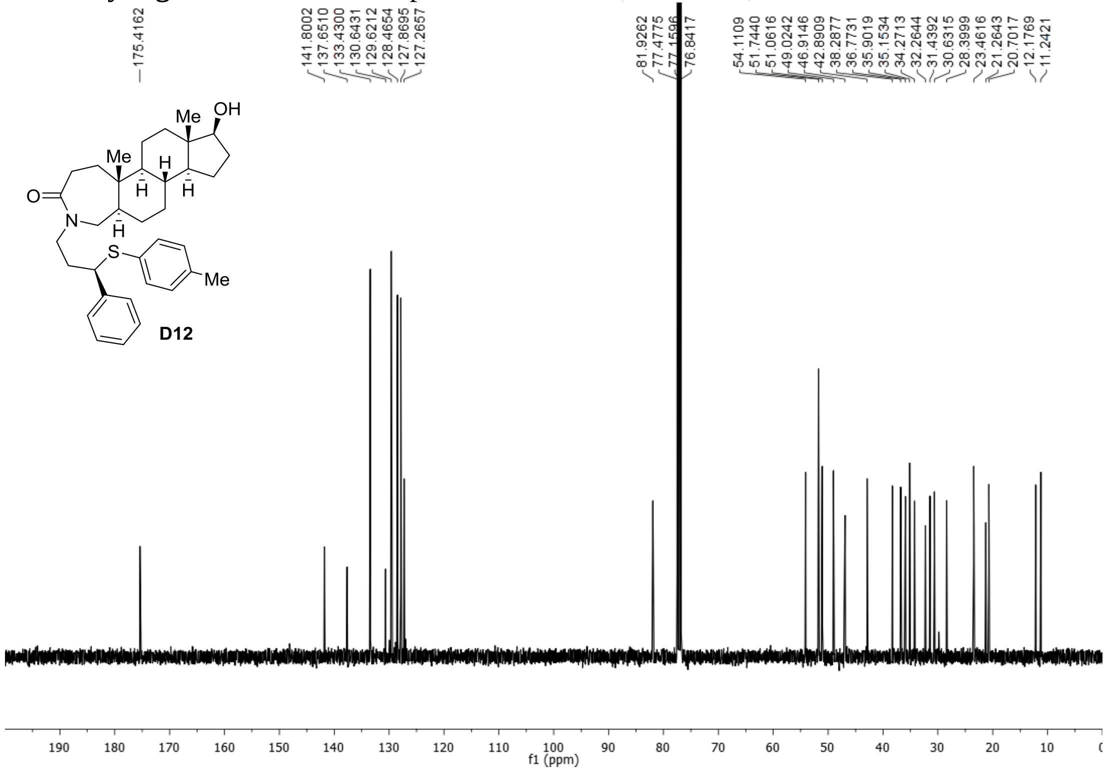

Supplementary Figure 224. ^{1}H NMR spectrum in $CDCl_3$, 400 MHz, of **D13**.

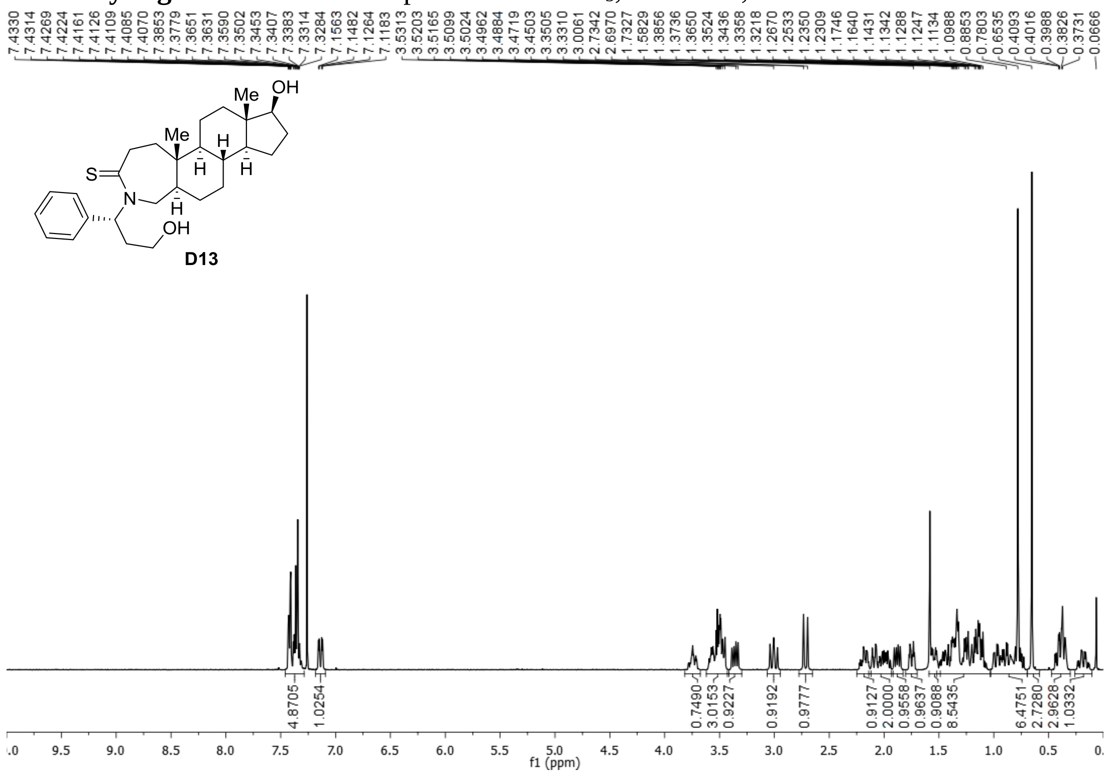

Supplementary Figure 225. ^{13}C NMR (APT) spectrum in $CDCl_3$, 151 MHz, of **D13**.

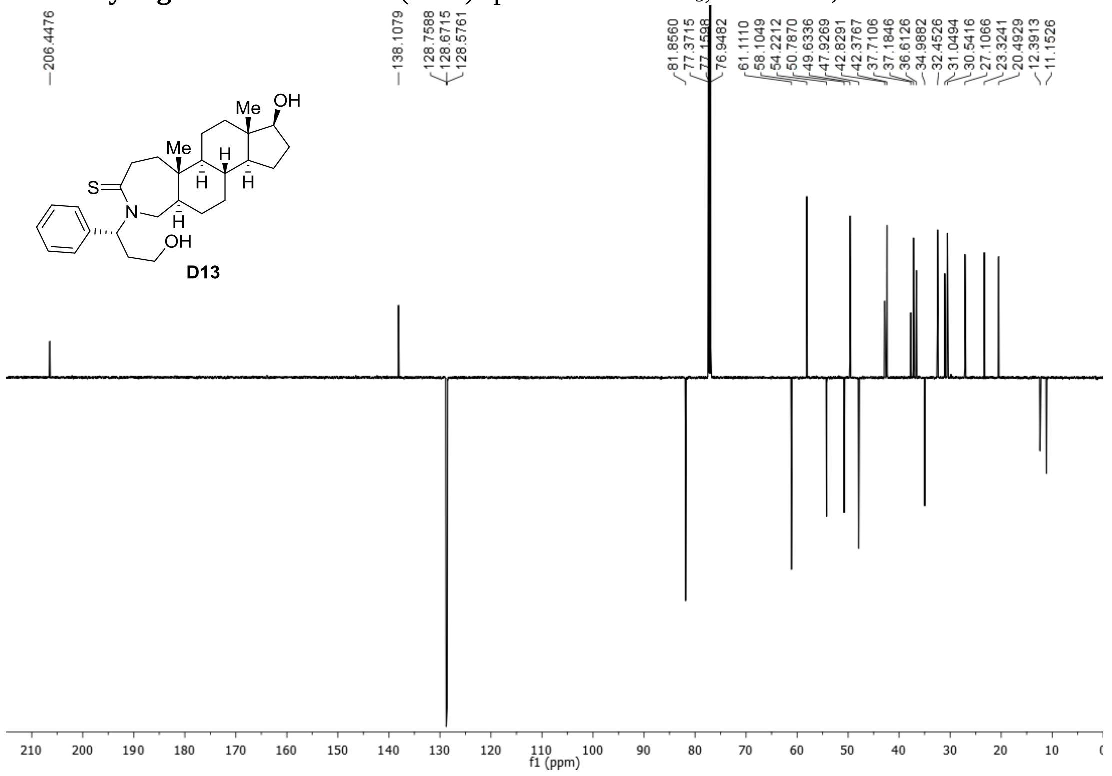

Supplementary Figure 226. ^{1}H NMR spectrum in $CDCl_3$, 400 MHz, of **D14**.

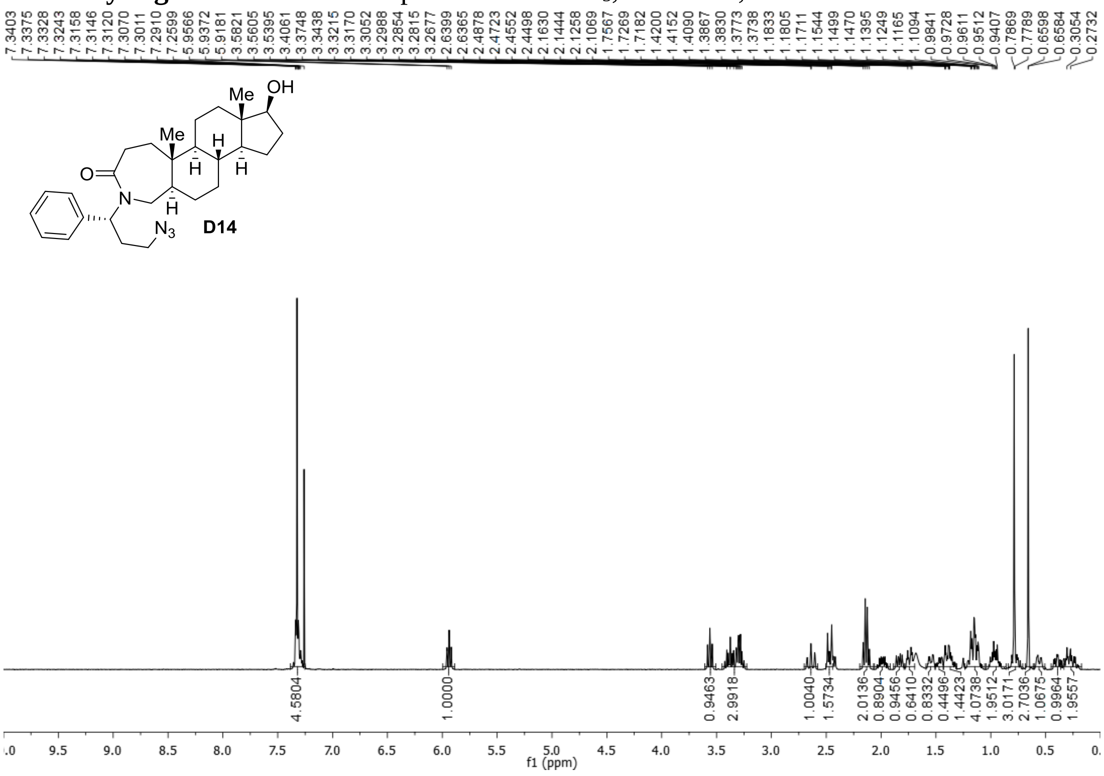

Supplementary Figure 227. ^{13}C NMR spectrum in $CDCl_3$, 101 MHz, of **D14**.

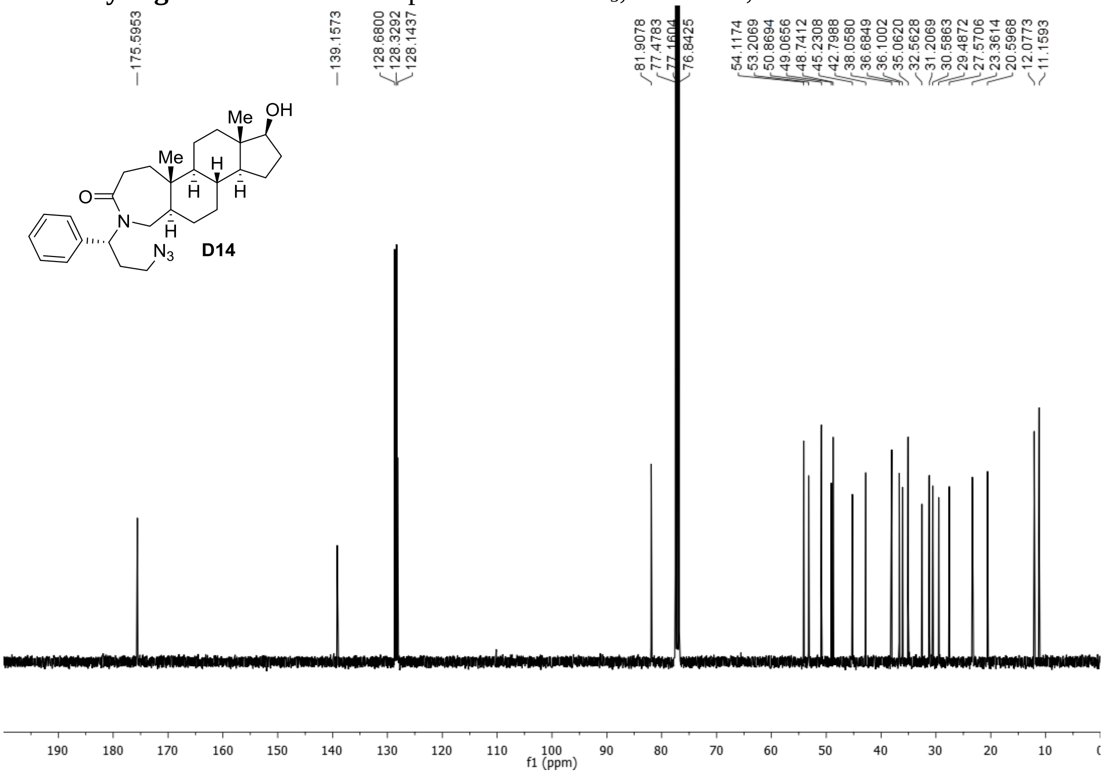

Supplementary Figure 228. ^{1}H NMR spectrum in $CDCl_3$, 400 MHz, of **D15**.

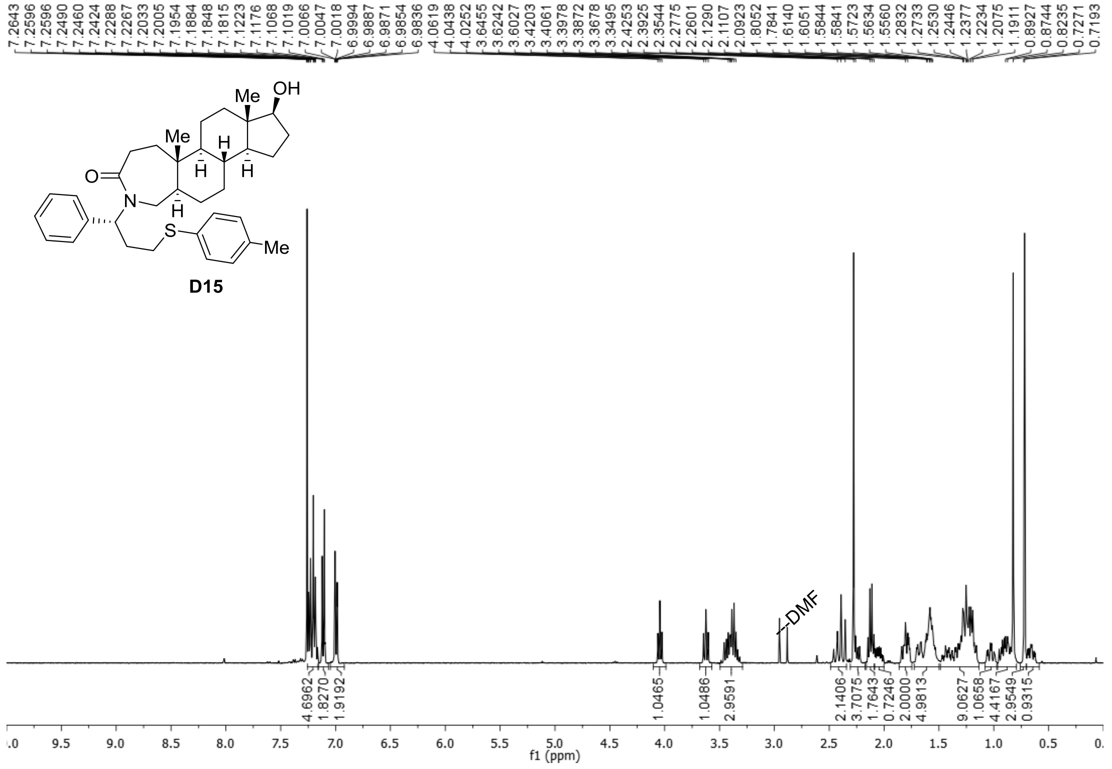

Supplementary Figure 229. ^{13}C NMR spectrum in $CDCl_3$, 101 MHz, of **D15**.

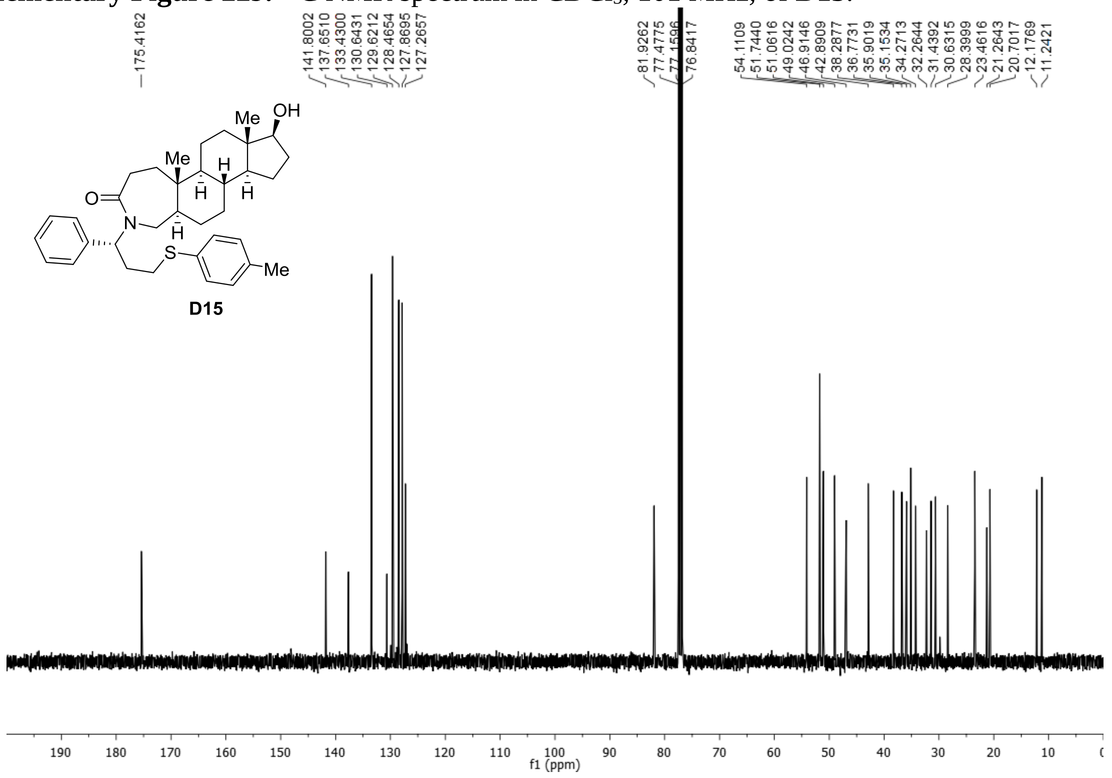

Supplementary Figure 230. ^{1}H NMR spectrum in $CDCl_3$, 400 MHz, of **D16**.

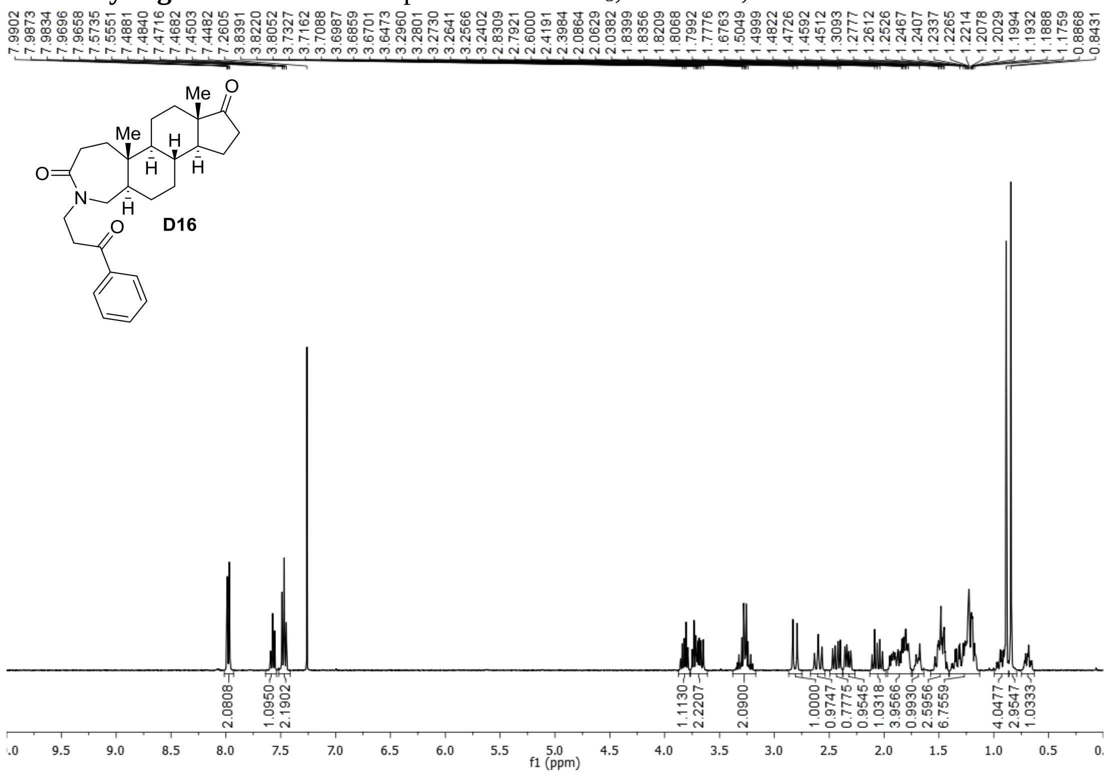

Supplementary Figure 231. ^{13}C NMR spectrum in $CDCl_3$, 101 MHz, of **D16**.

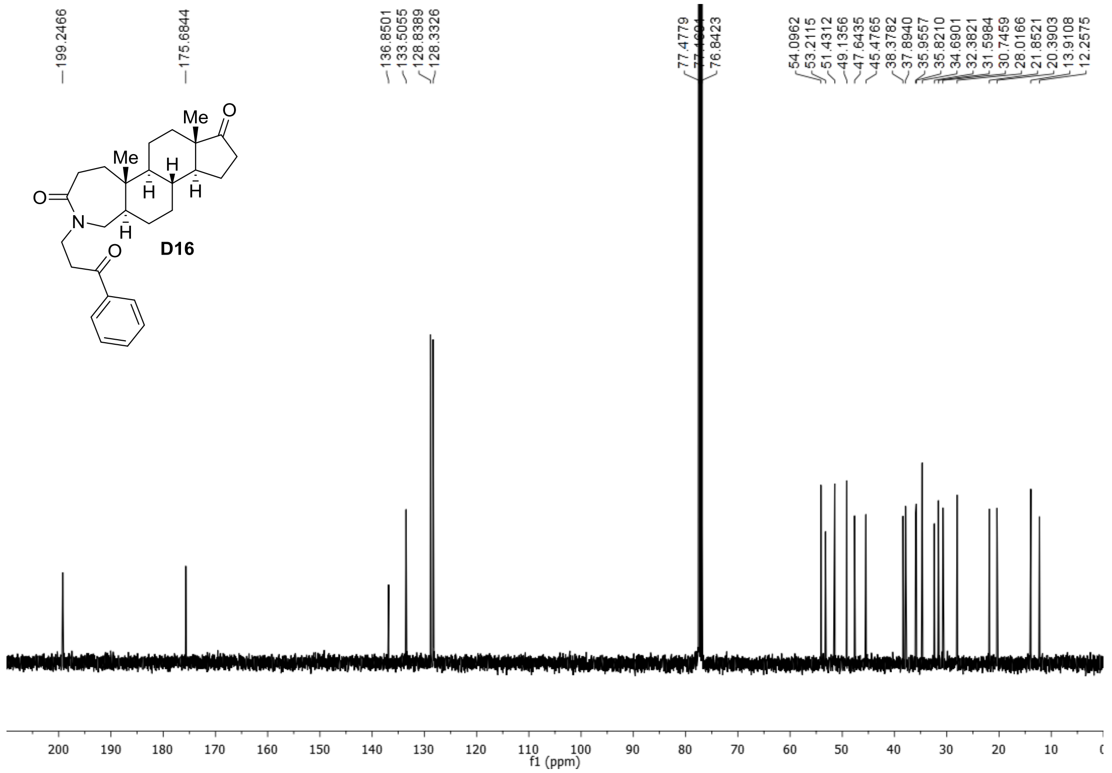

Supplementary Figure 232. ^{1}H NMR spectrum in $CDCl_3$, 400 MHz, of **D17**.

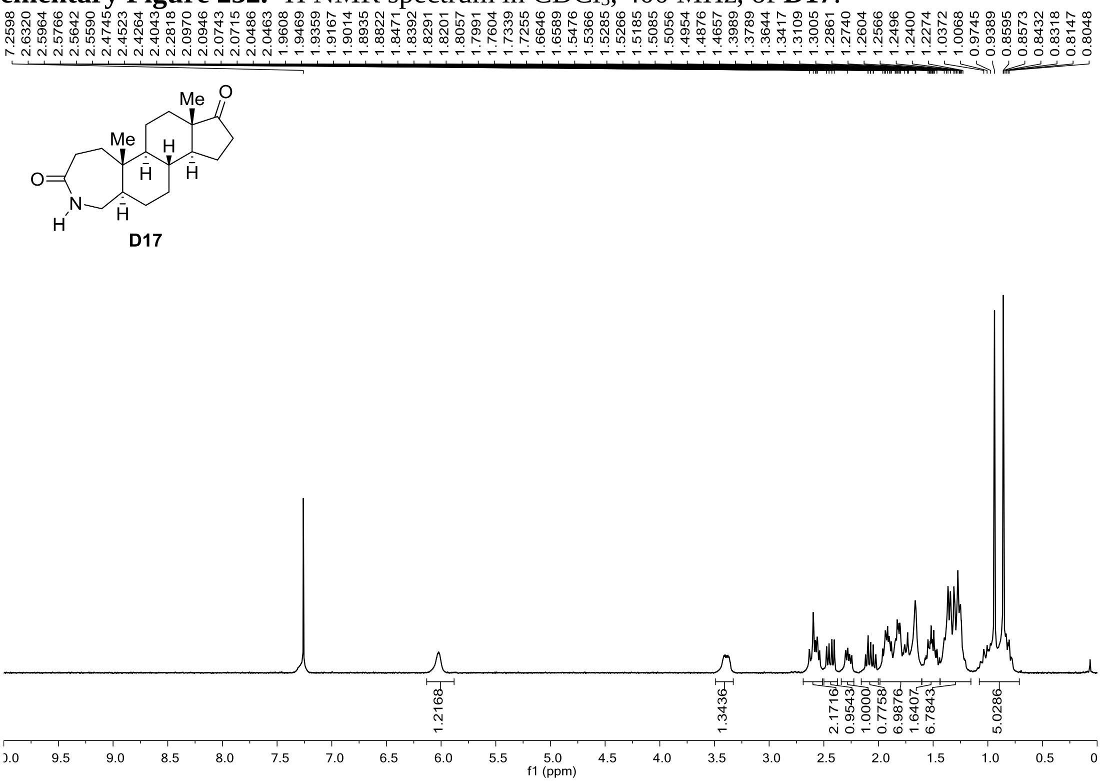

Supplementary Figure 233. ^{13}C NMR spectrum in $CDCl_3$, 151 MHz, of **D17**.

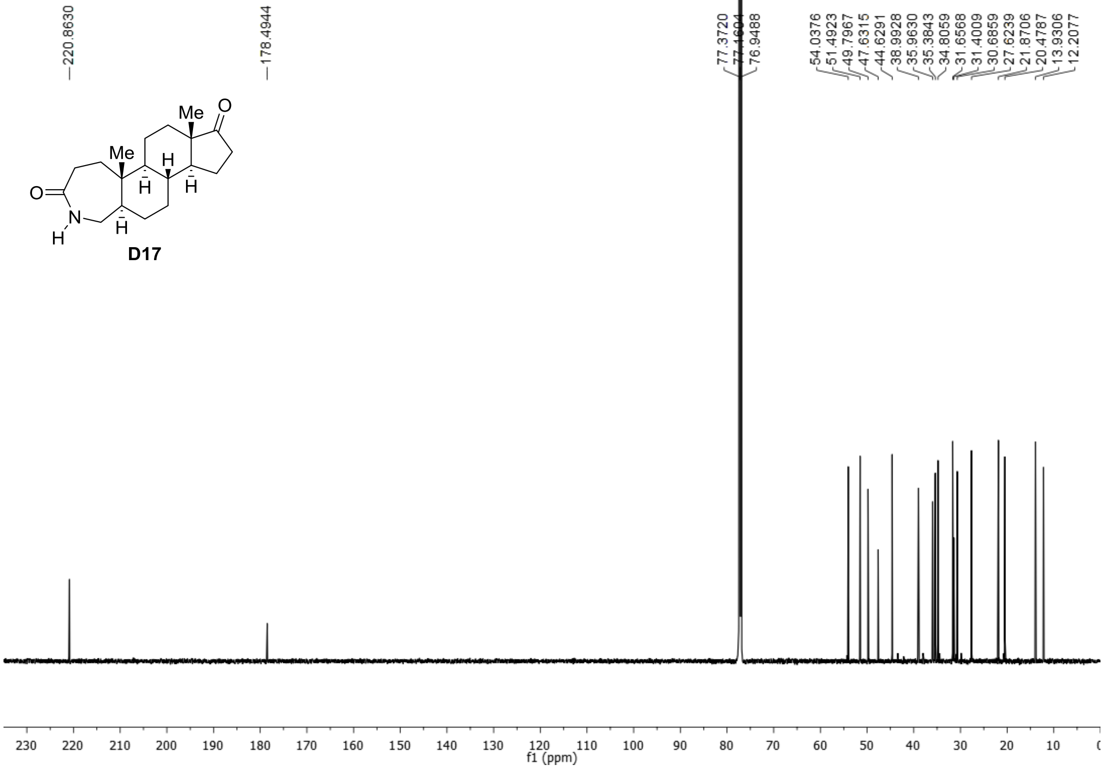

Supplementary Figure 234. ^{1}H NMR spectrum in $CDCl_3$, 400 MHz, of **E1**.

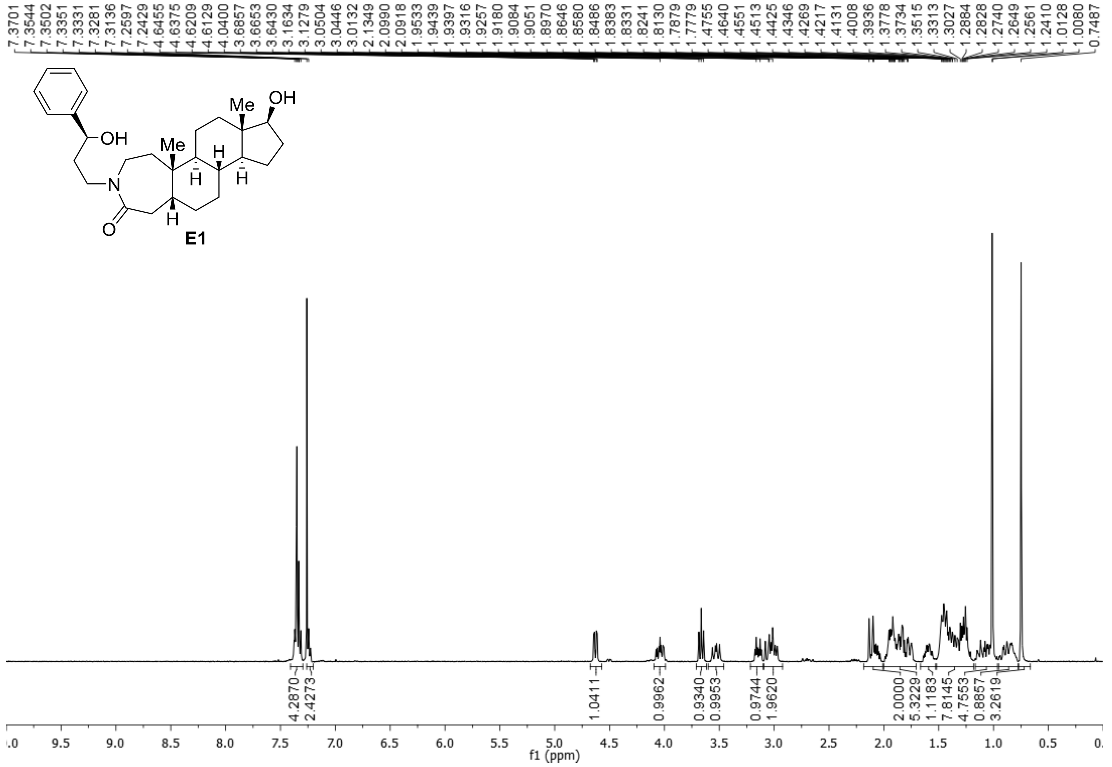

Supplementary Figure 235. ^{13}C NMR spectrum in $CDCl_3$, 151 MHz, of **E1**.

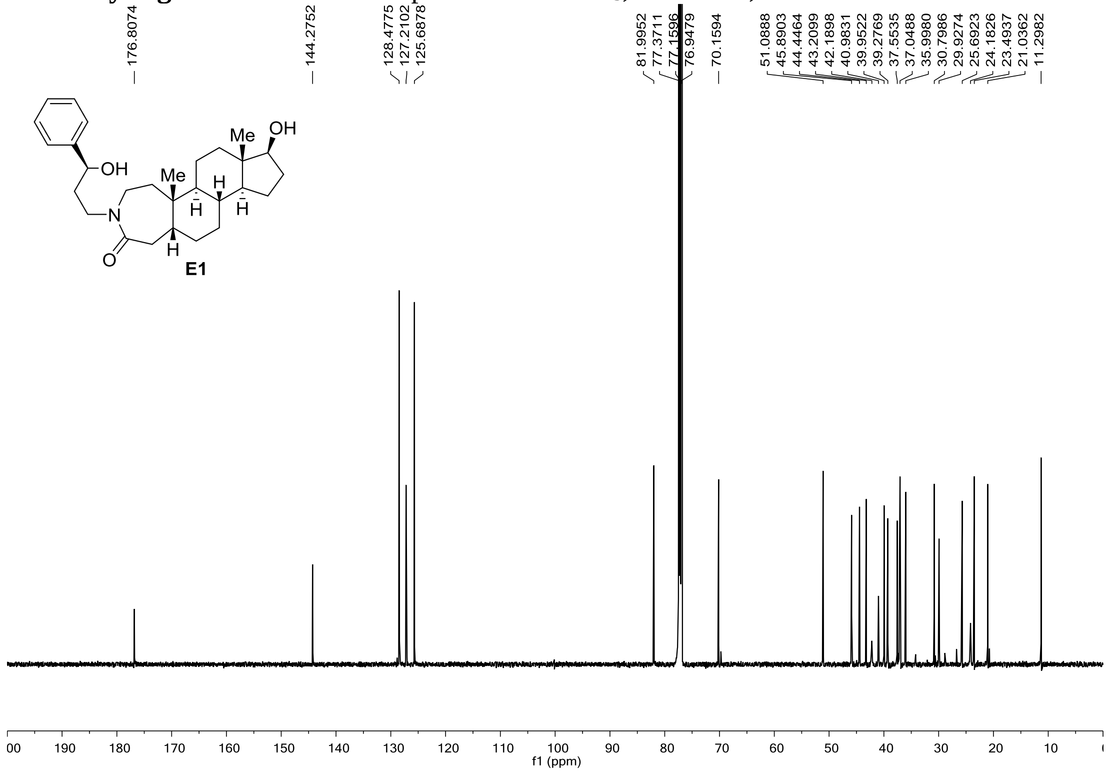

Supplementary Figure 236. HSQC spectrum of **E1**.

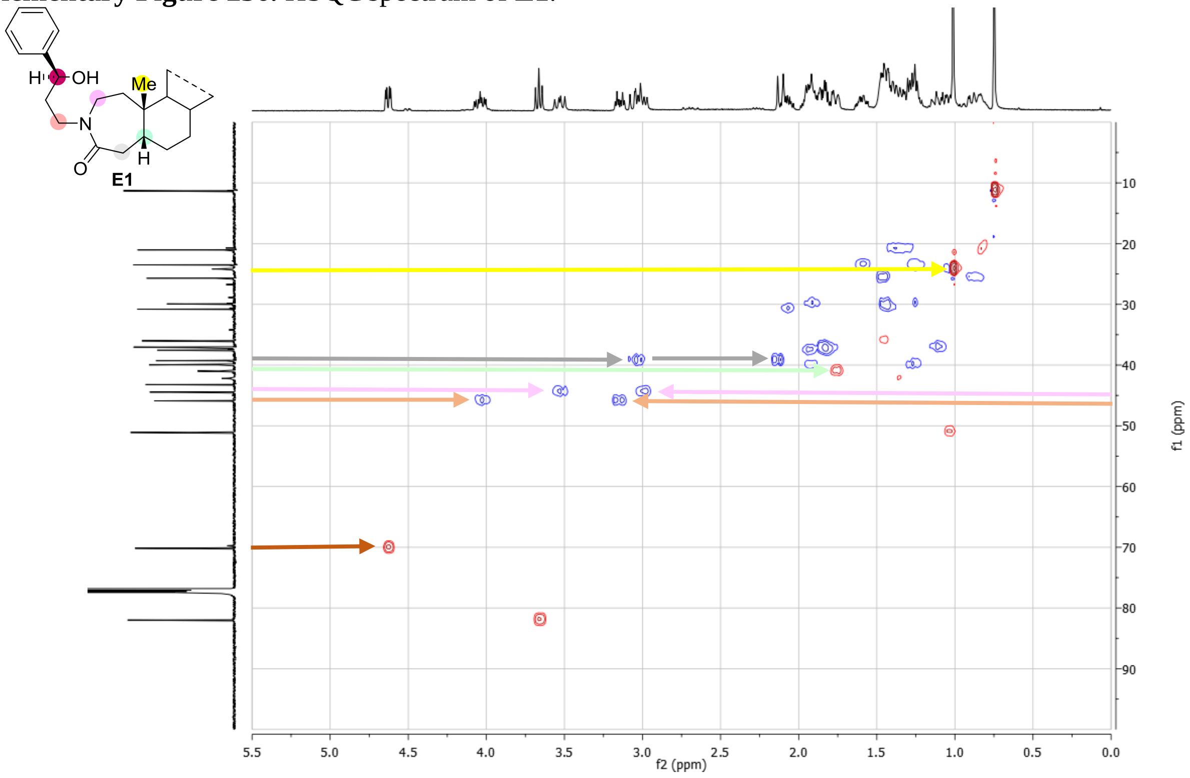

Supplementary Figure 237. HMBC spectrum of **E1**.

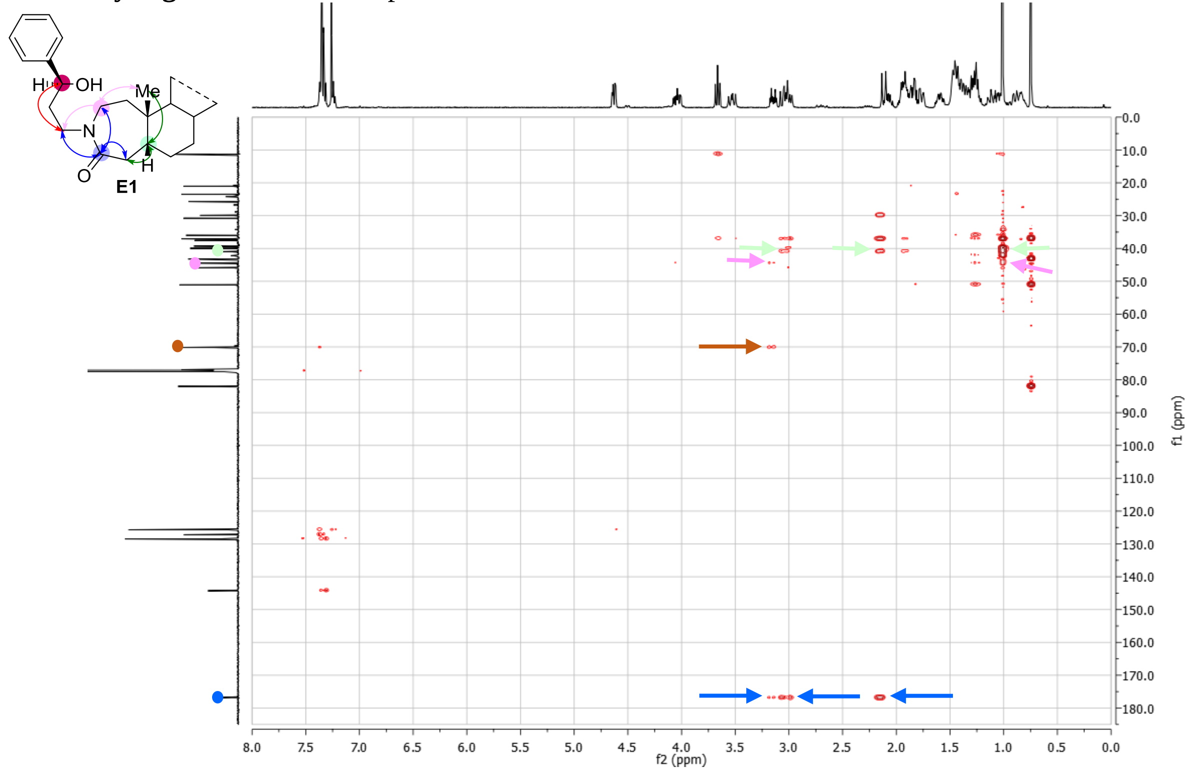

Supplementary Figure 238. ^{1}H NMR spectrum in $CDCl_3$, 400 MHz, of **E2**.

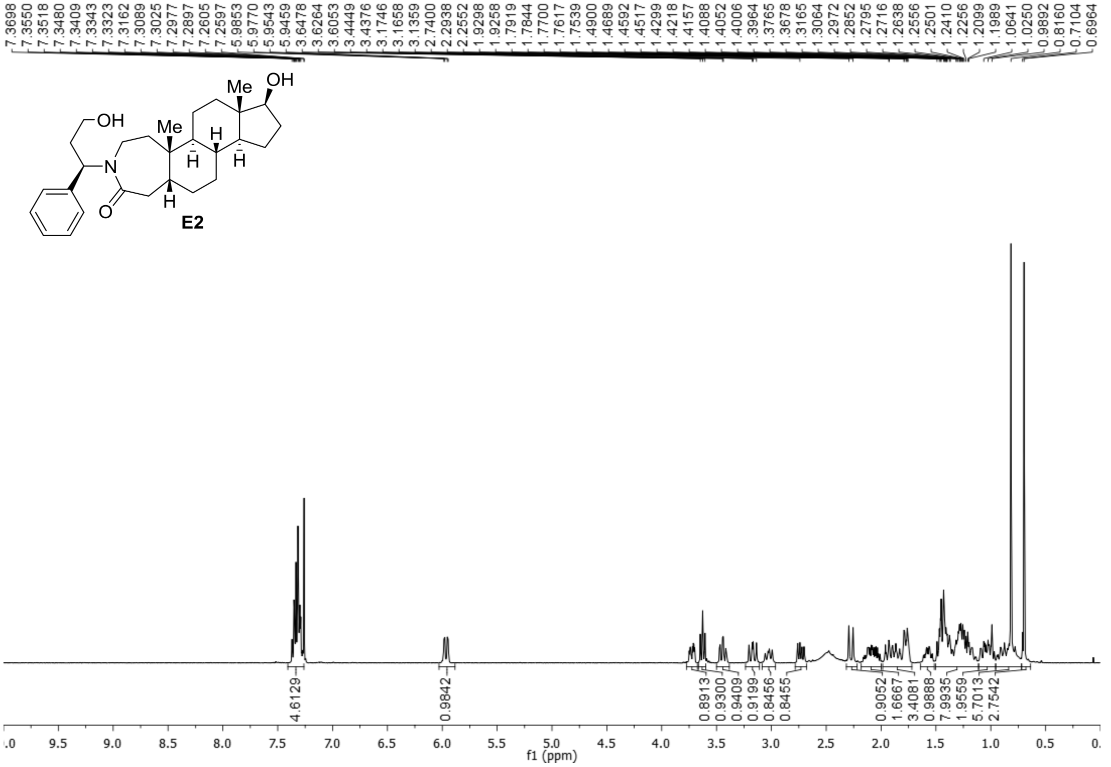

Supplementary Figure 239. ^{13}C NMR spectrum in $CDCl_3$, 151 MHz, of **E2**.

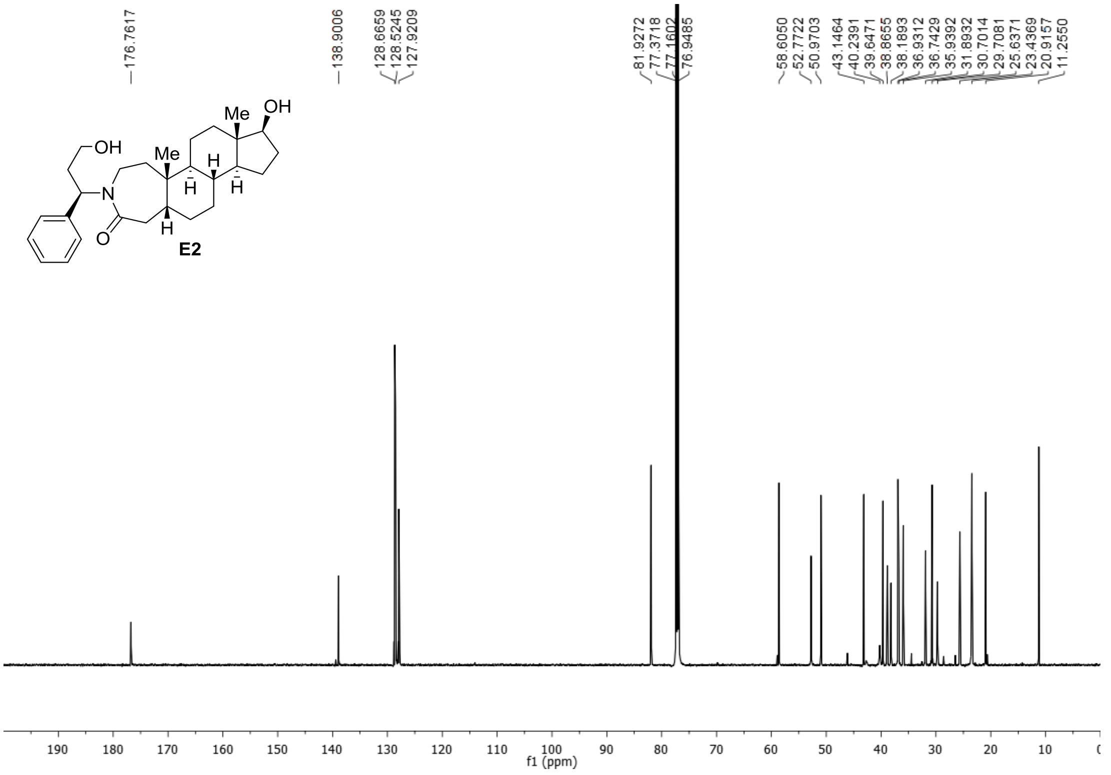

Supplementary Figure 240. 1D NOE spectrum in $CDCl_3$, 400 MHz, of **E2**.

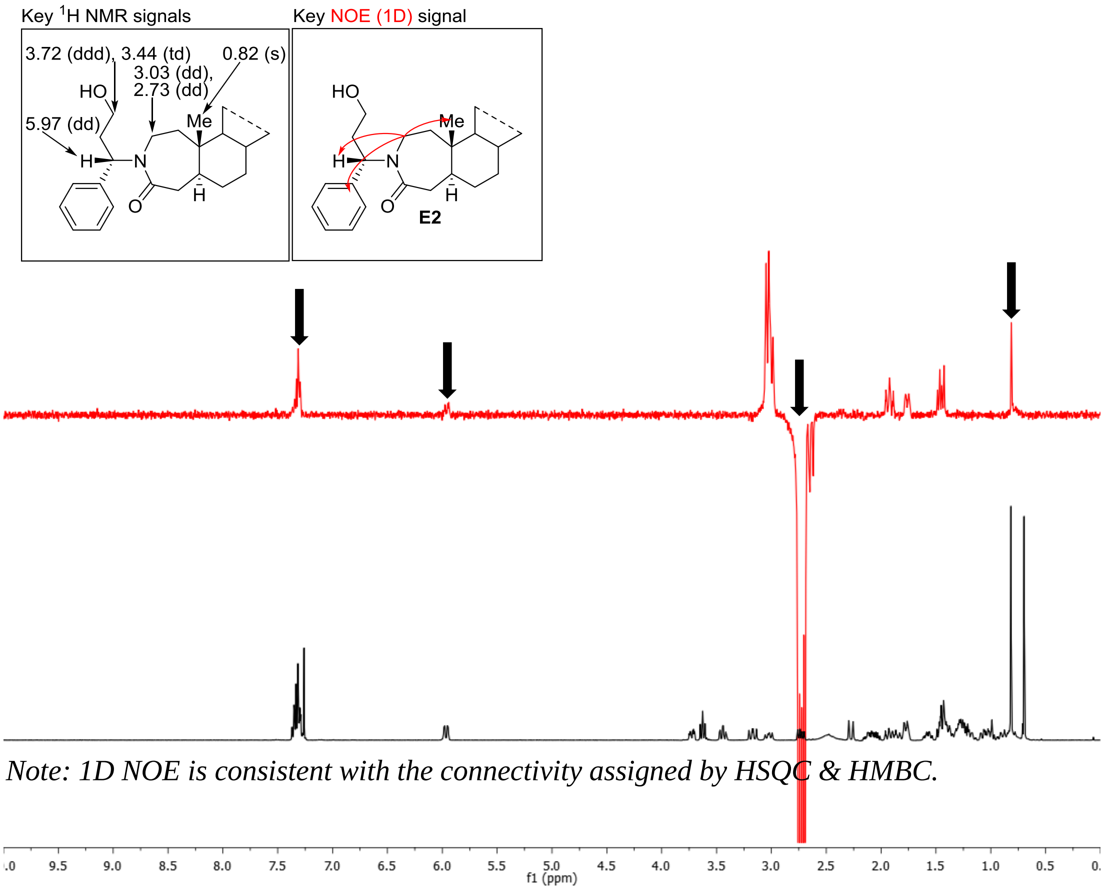

Supplementary Figure 241. HSQC spectrum of **E2**.

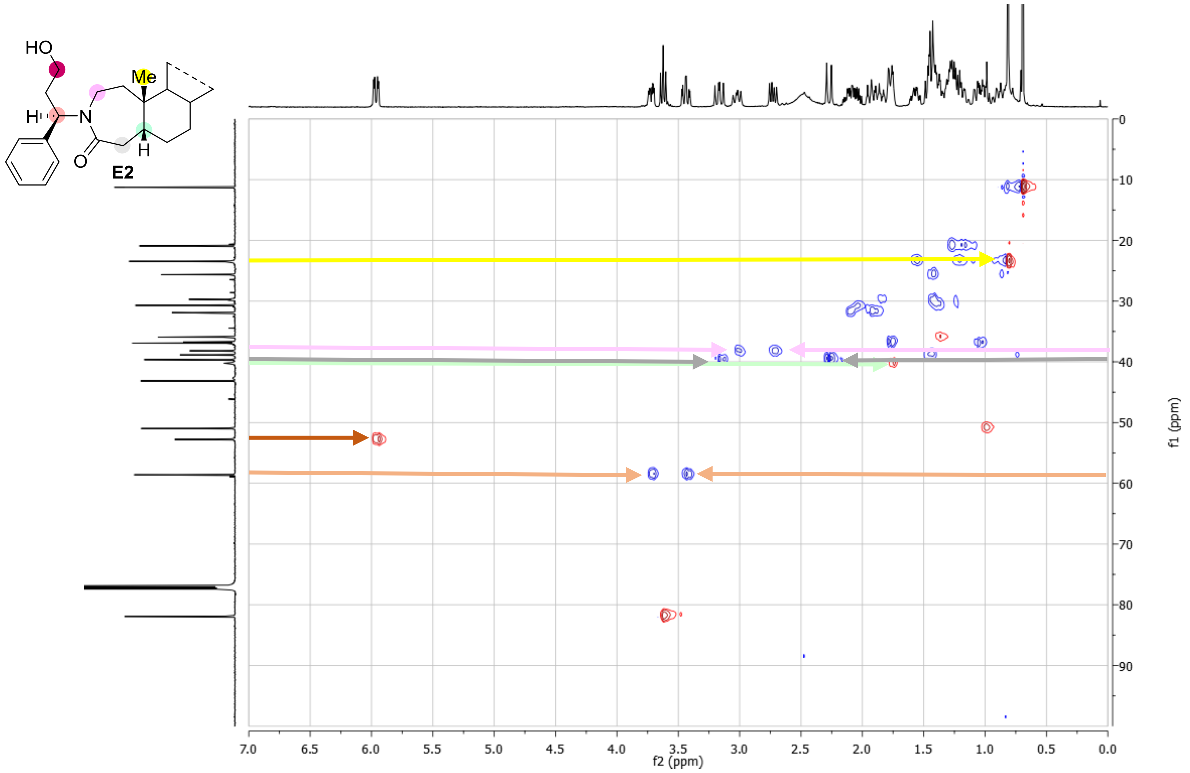

Supplementary Figure 242. HMBC spectrum of **E2**.

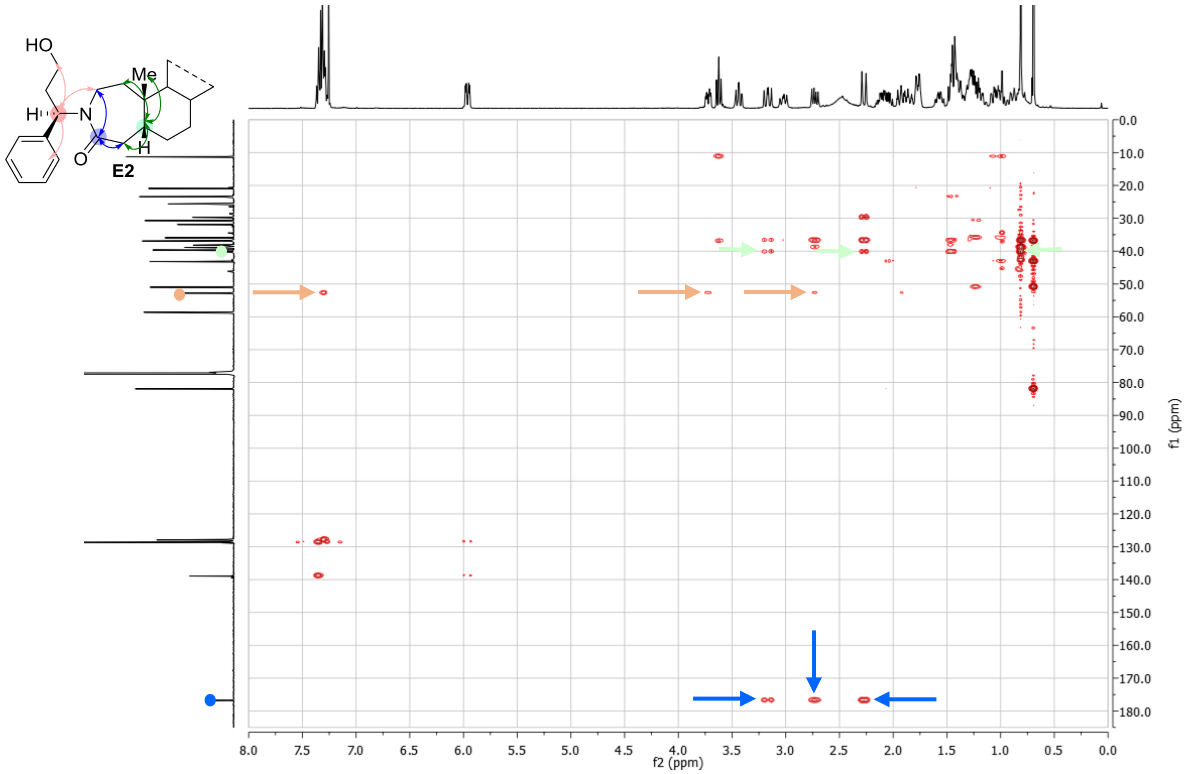

Supplementary Figure 243. ^{1}H NMR spectrum in $CDCl_3$, 400 MHz, of **E3**.

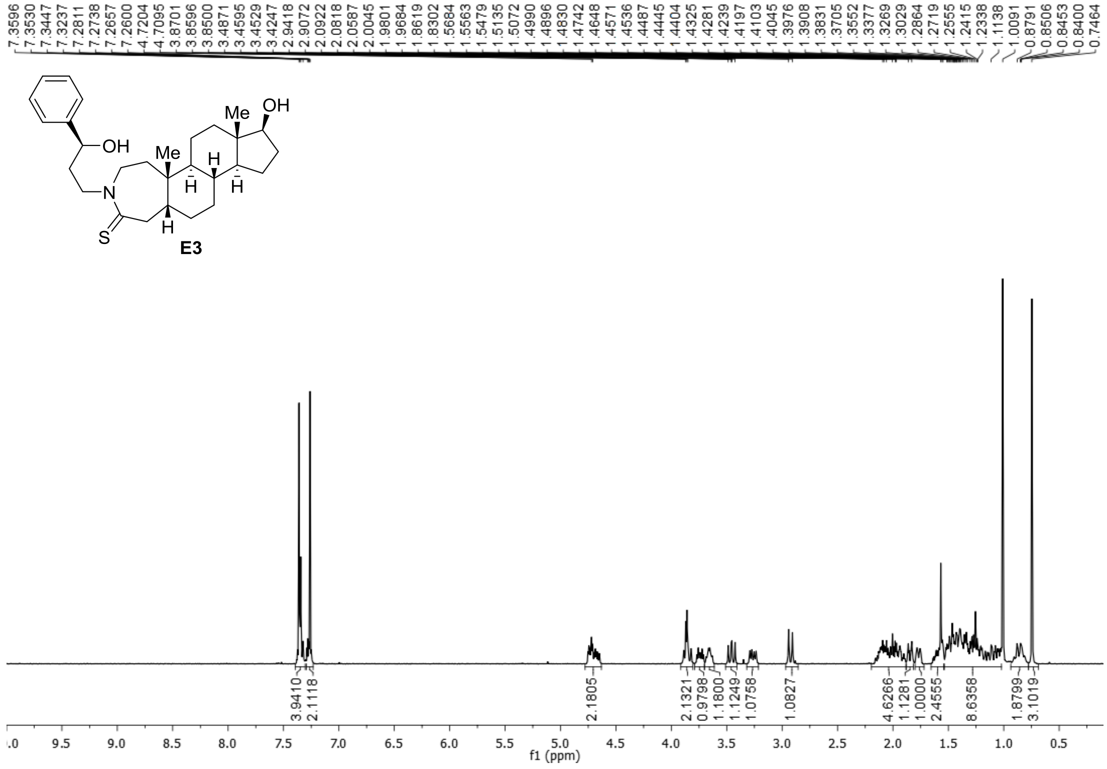

Supplementary Figure 244. ^{13}C NMR spectrum in $CDCl_3$, 151 MHz, of **E3**.

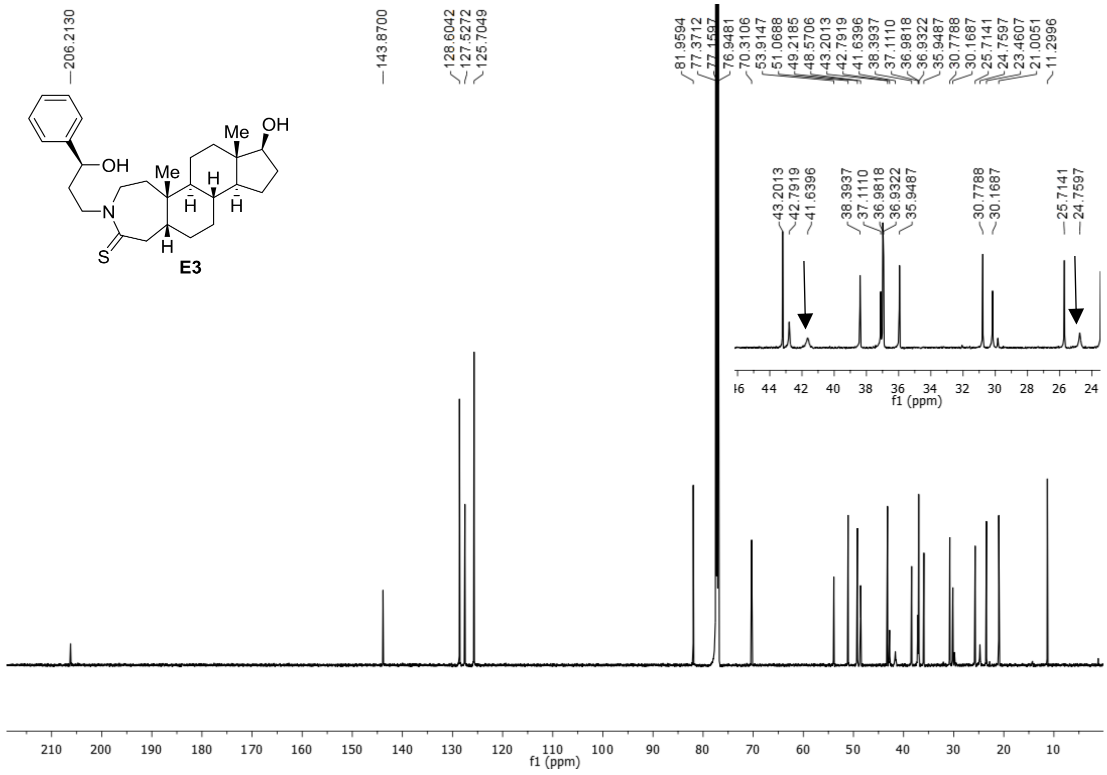

Supplementary Figure 245. ^{1}H NMR spectrum in $CDCl_3$, 400 MHz, of **E4**.

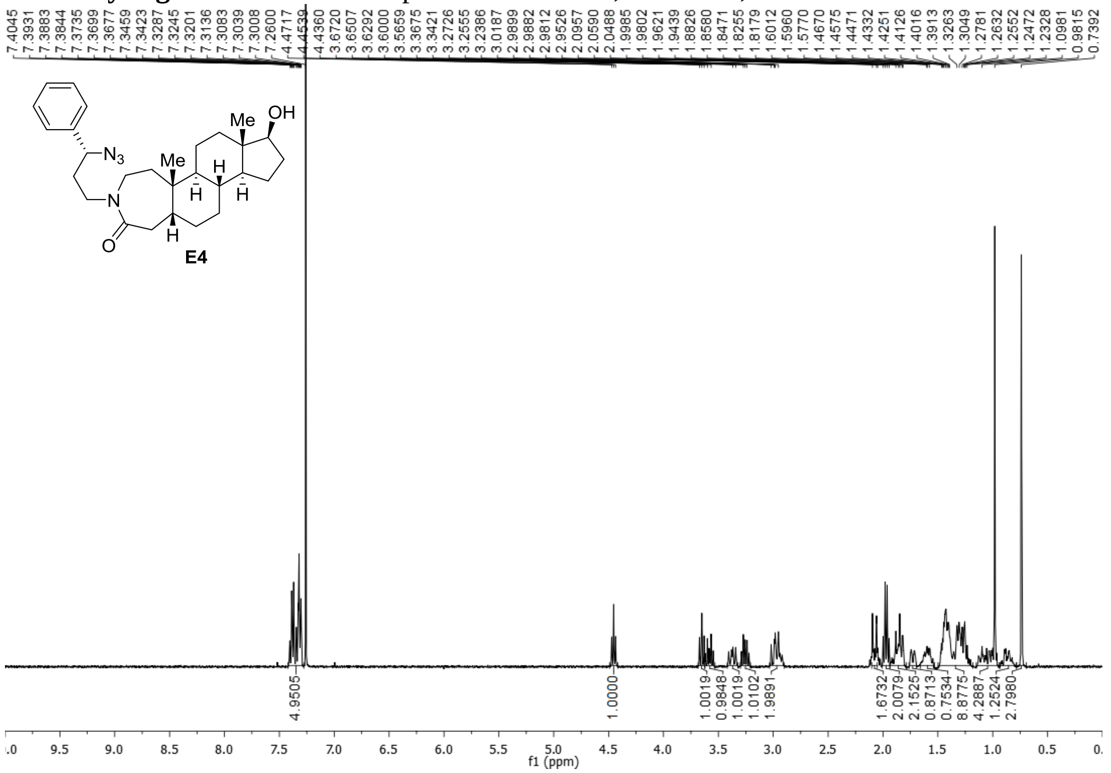

Supplementary Figure 246. ^{13}C NMR spectrum in $CDCl_3$, 151 MHz, of **E4**.

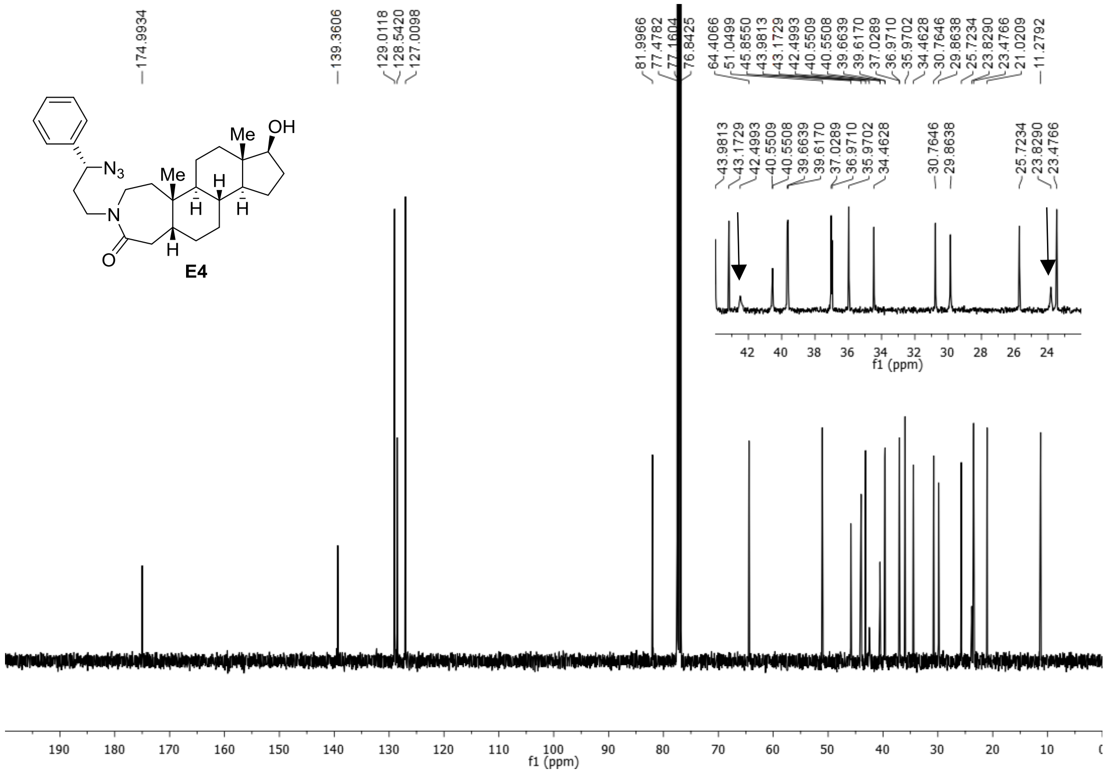

Supplementary Figure 247. ^{1}H NMR spectrum in $CDCl_3$, 400 MHz, of **E5**.

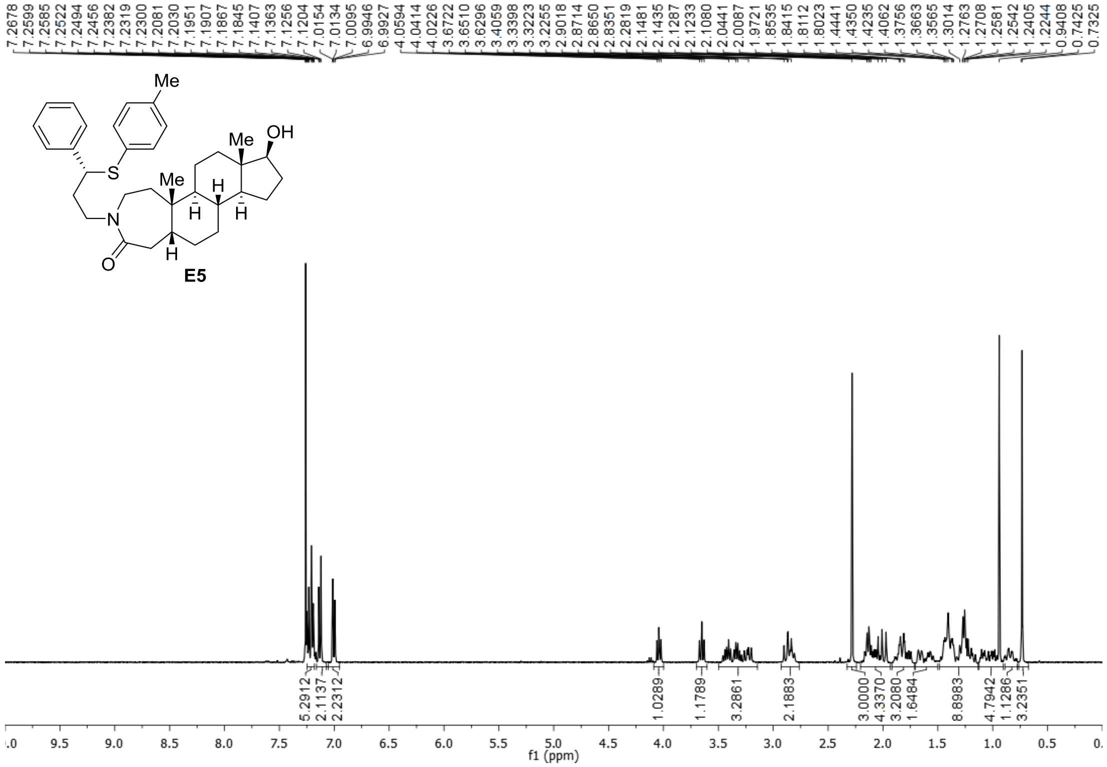

Supplementary Figure 248. ^{13}C NMR spectrum in $CDCl_3$, 101 MHz, of **E5**.

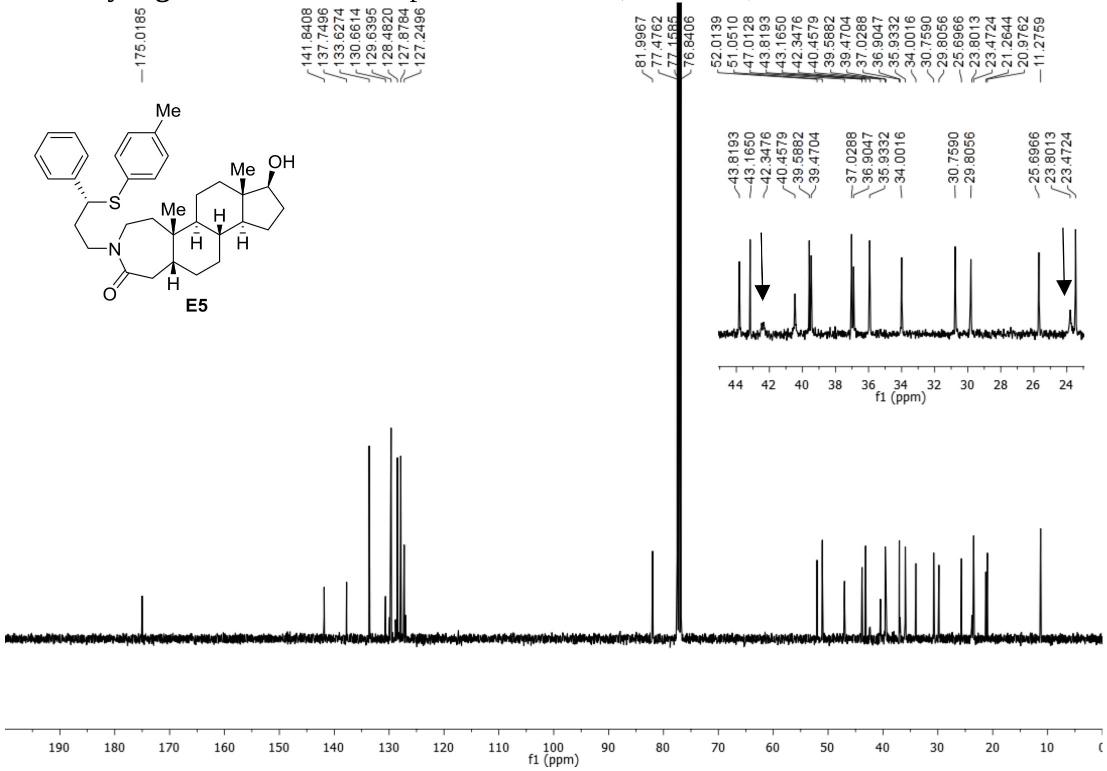

Supplementary Figure 249. ^{1}H NMR spectrum in $CDCl_3$, 400 MHz, of **F1**.

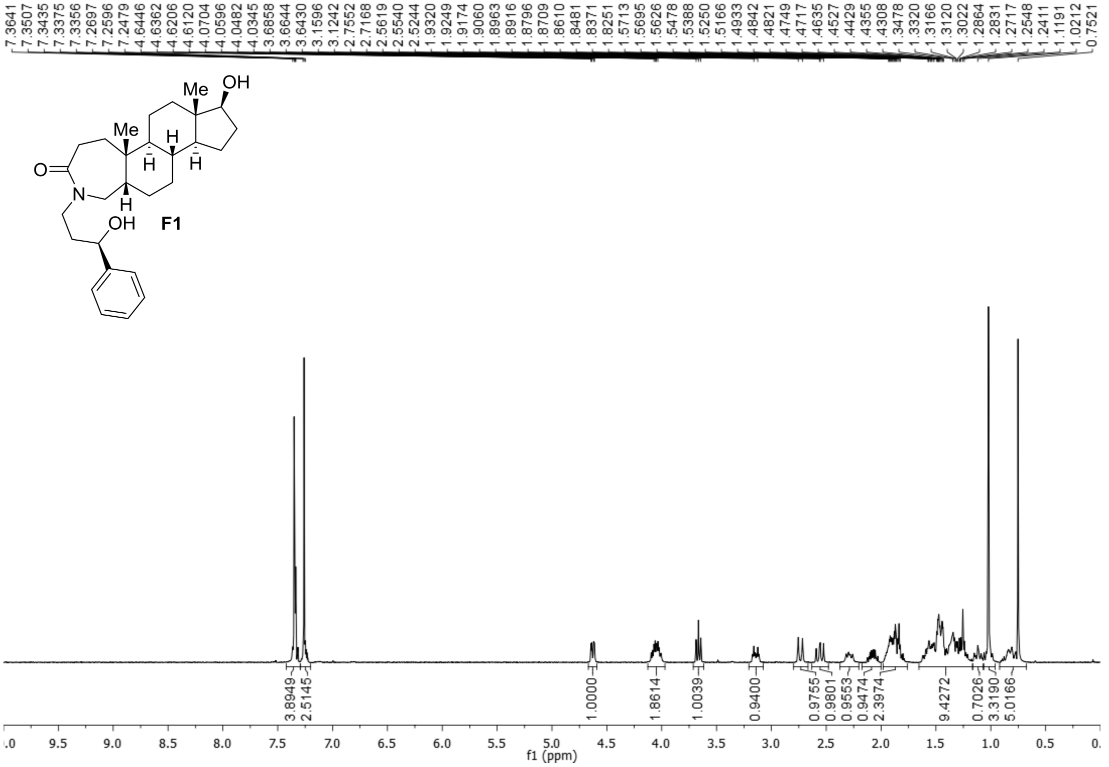

Supplementary Figure 250. ^{13}C NMR spectrum in $CDCl_3$, 151 MHz, of **F1**.

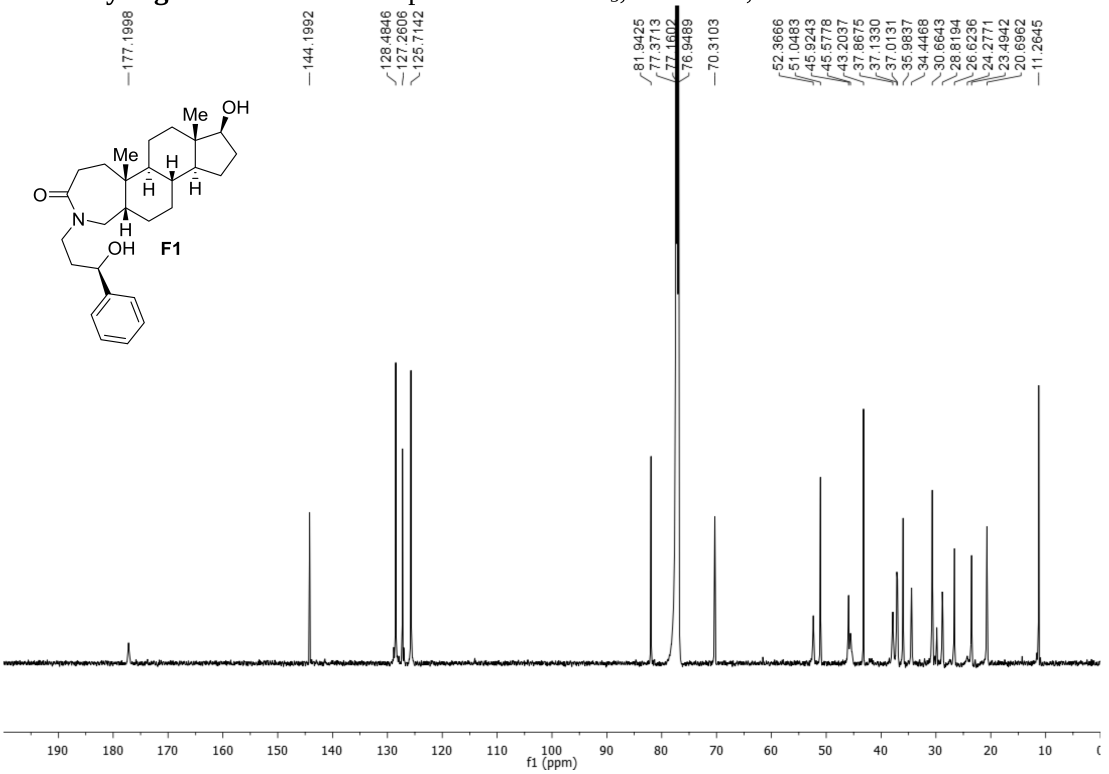

Supplementary Figure 251. COSY spectrum of **F1**.

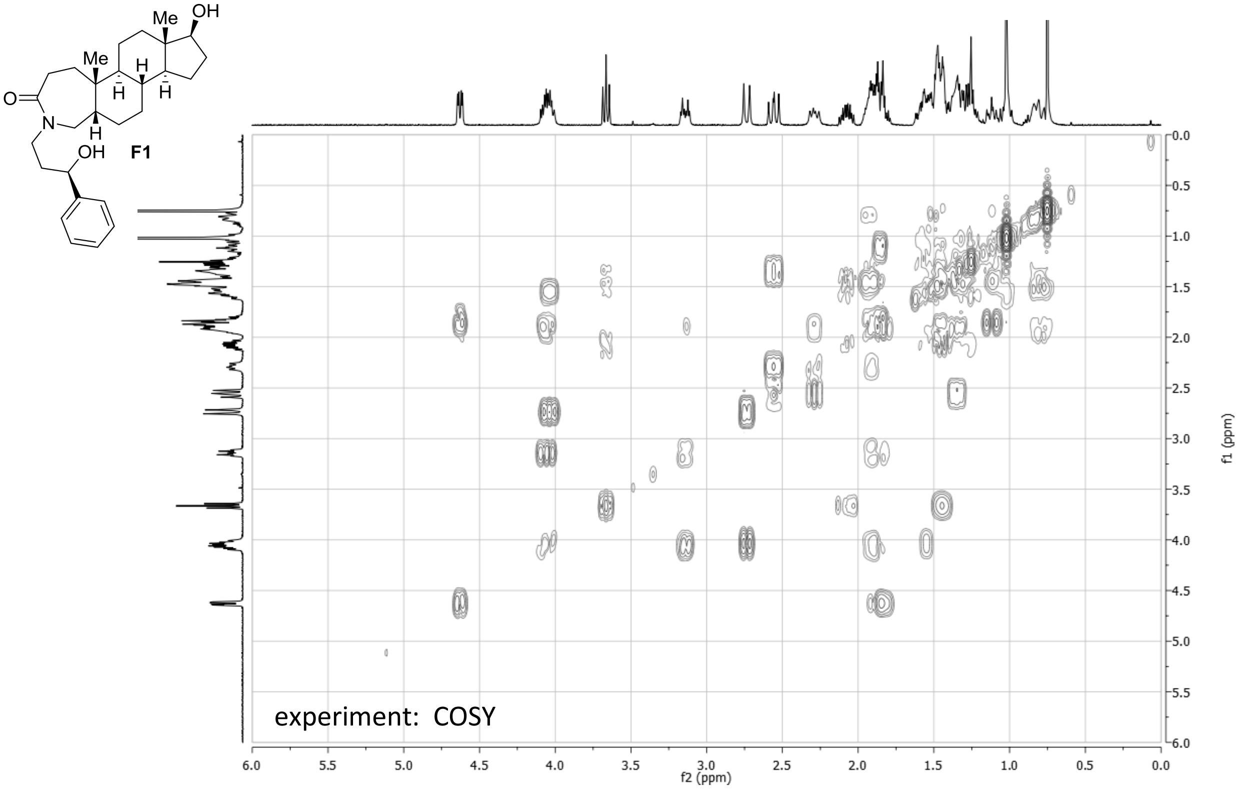

Supplementary Figure 252. 1D NOE spectrum in $CDCl_3$, 400 MHz, of **F1**.

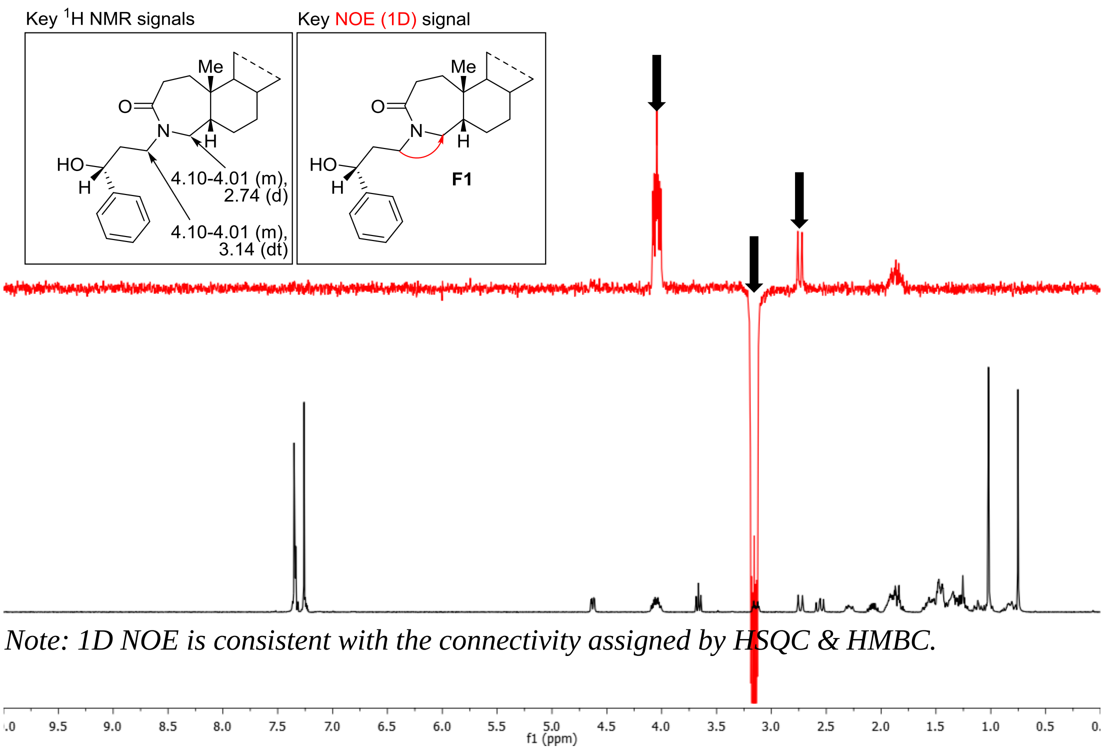

Supplementary Figure 253. HSQC spectrum of **F1**.

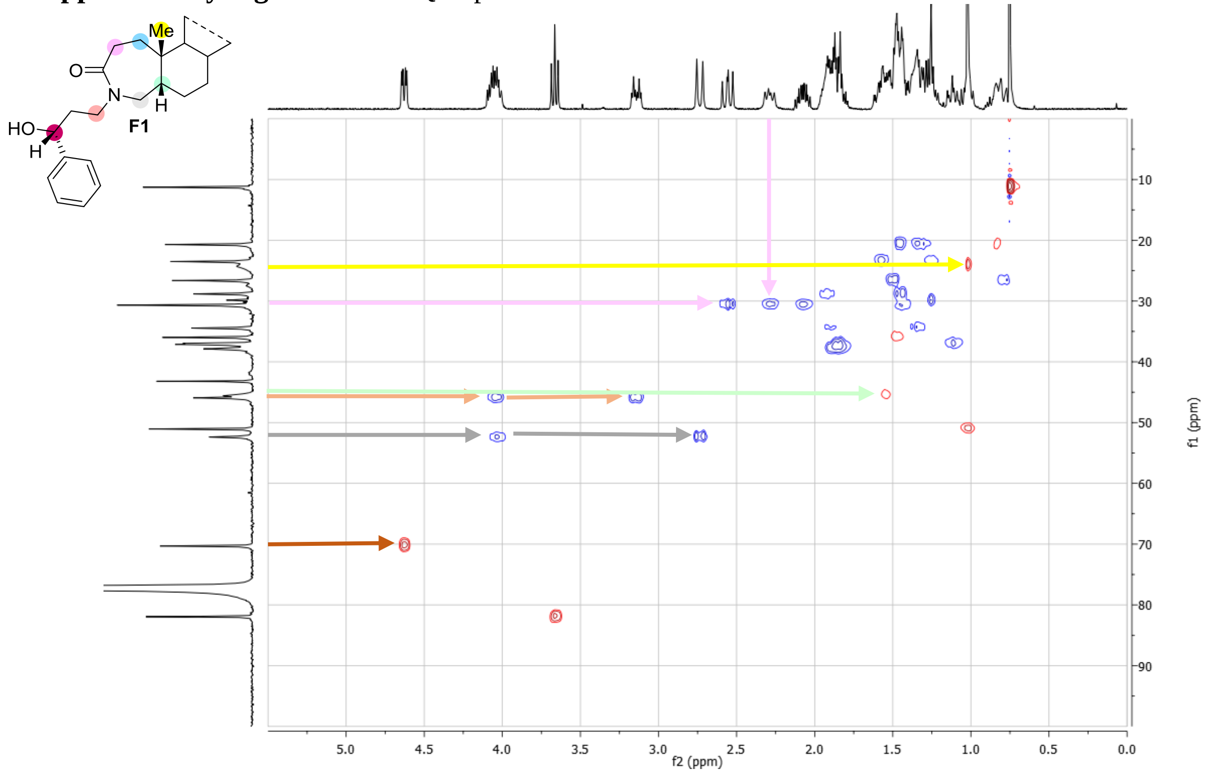

Supplementary Figure 254. HMBC spectrum of **F1**.

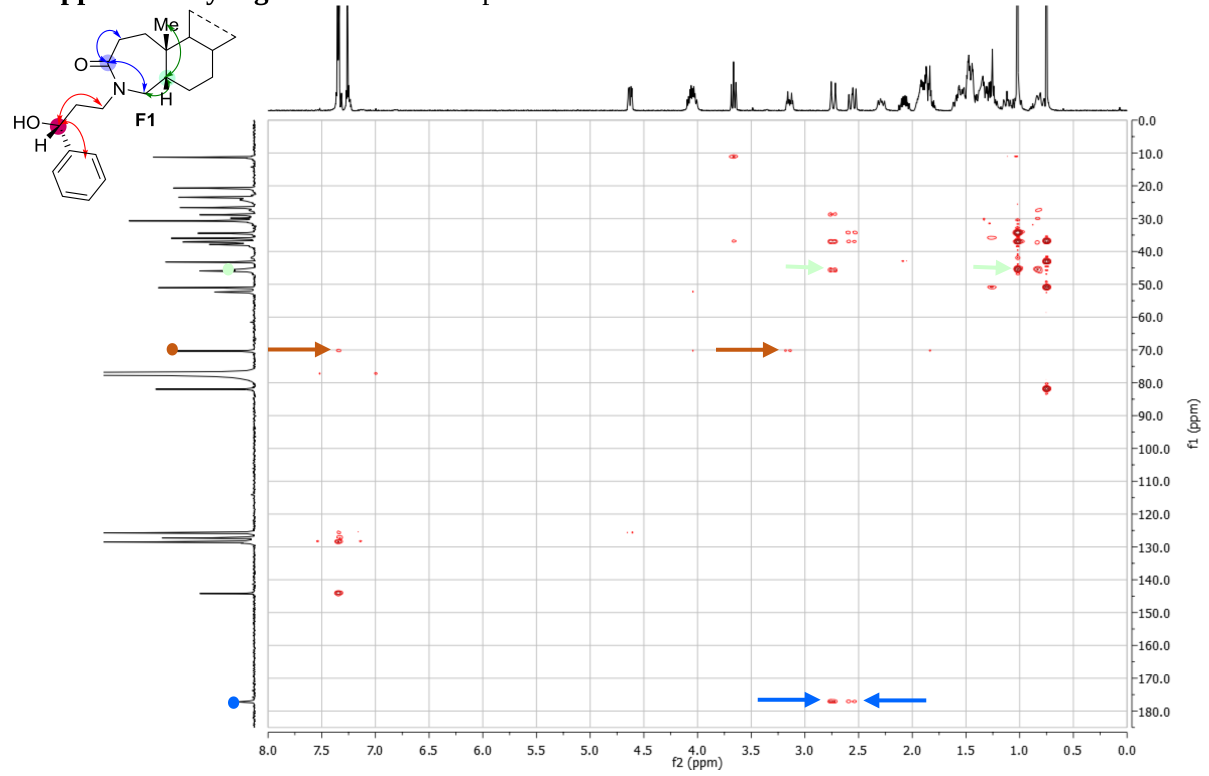

Supplementary Figure 255. ^{1}H NMR spectrum in $CDCl_3$, 400 MHz, of **F2**.

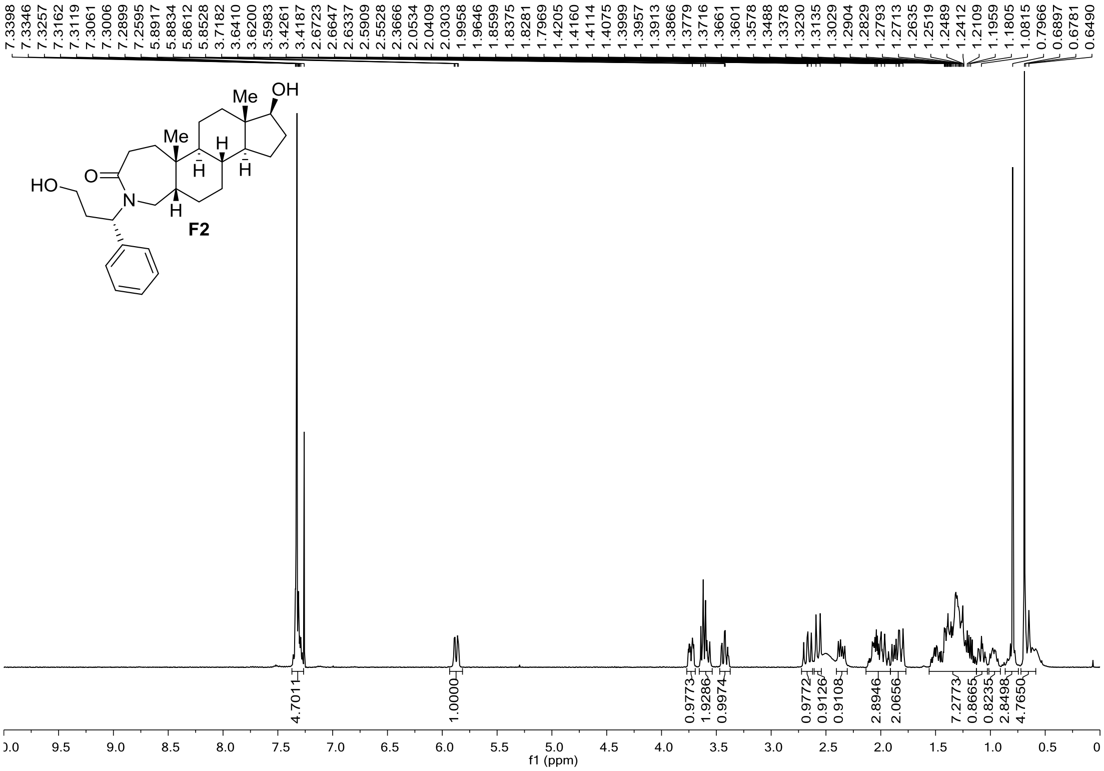

Supplementary Figure 256. ^{13}C NMR spectrum in $CDCl_3$, 151 MHz, of **F2**.

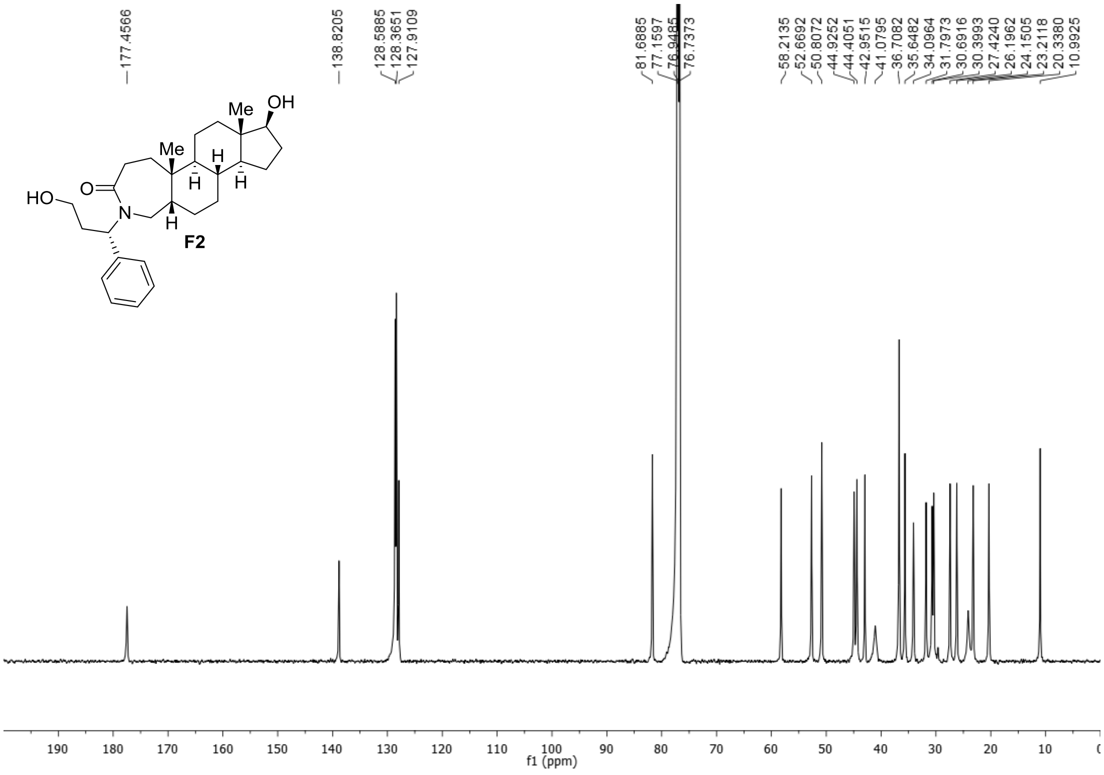

Supplementary Figure 257. 1D NOE spectrum in $CDCl_3$, 400 MHz, of **F2**.

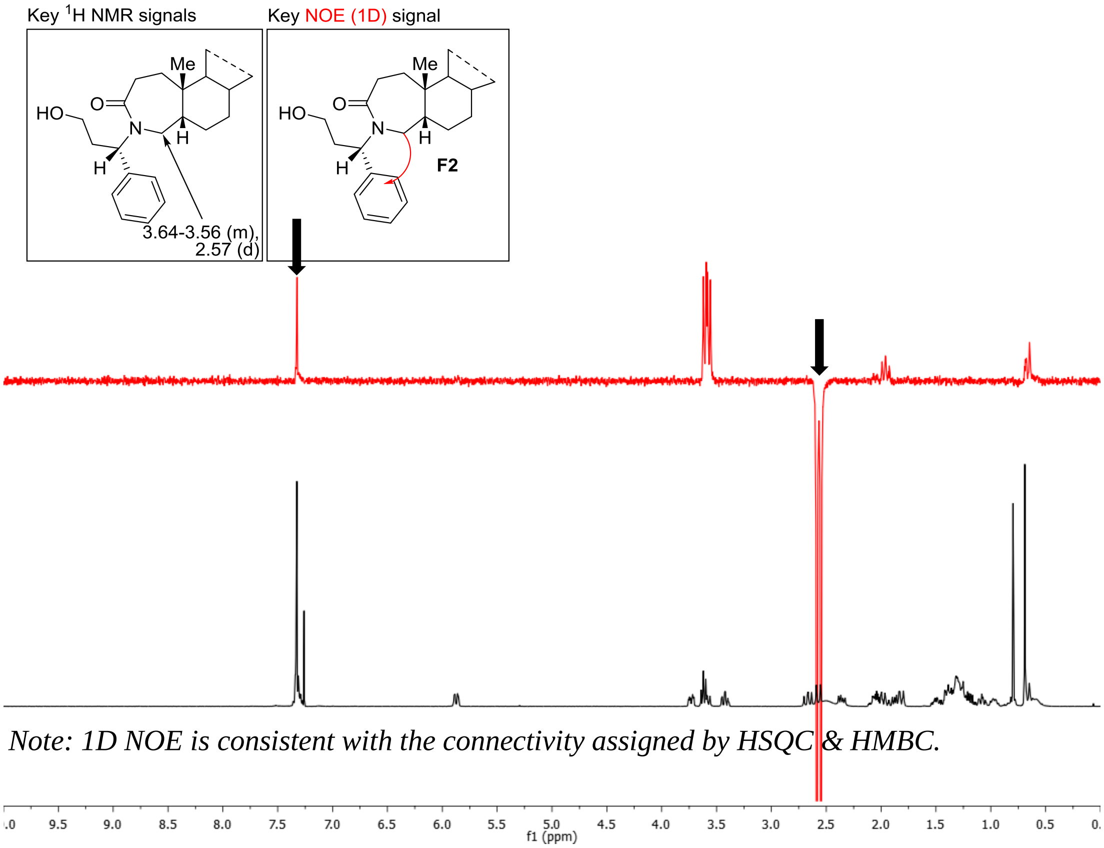

Supplementary Figure 258. 1D NOE spectrum in $CDCl_3$, 400 MHz, of **F2**.

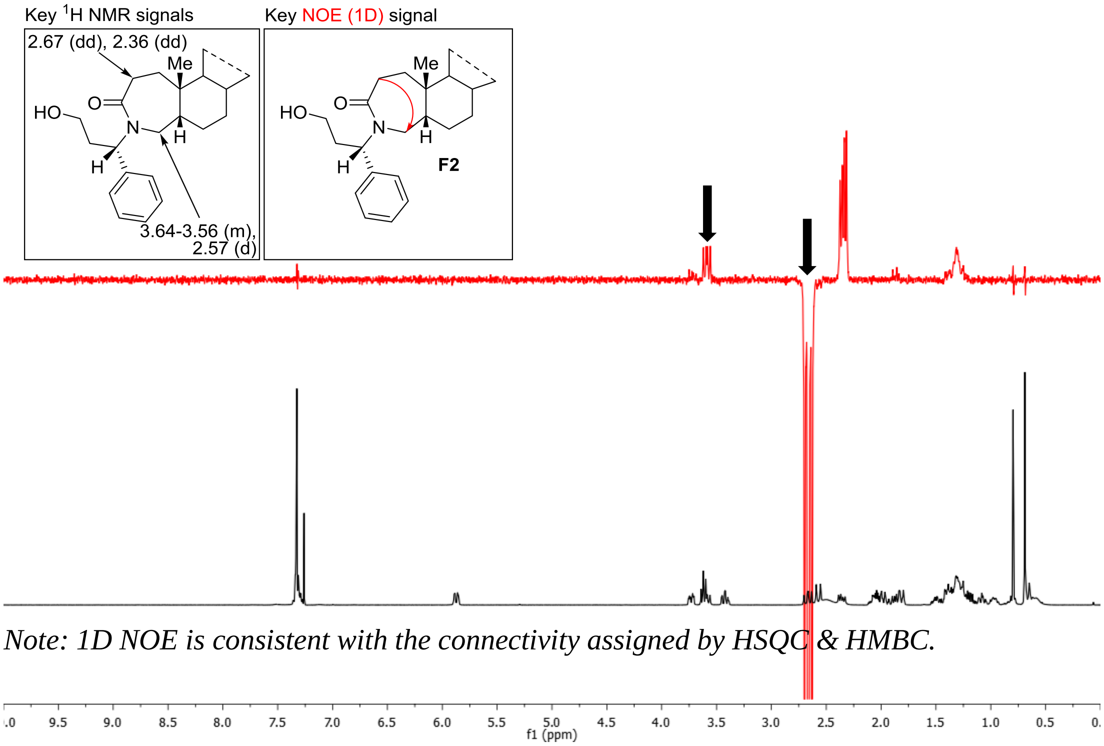

Supplementary Figure 259. HSQC spectrum of **F2**.

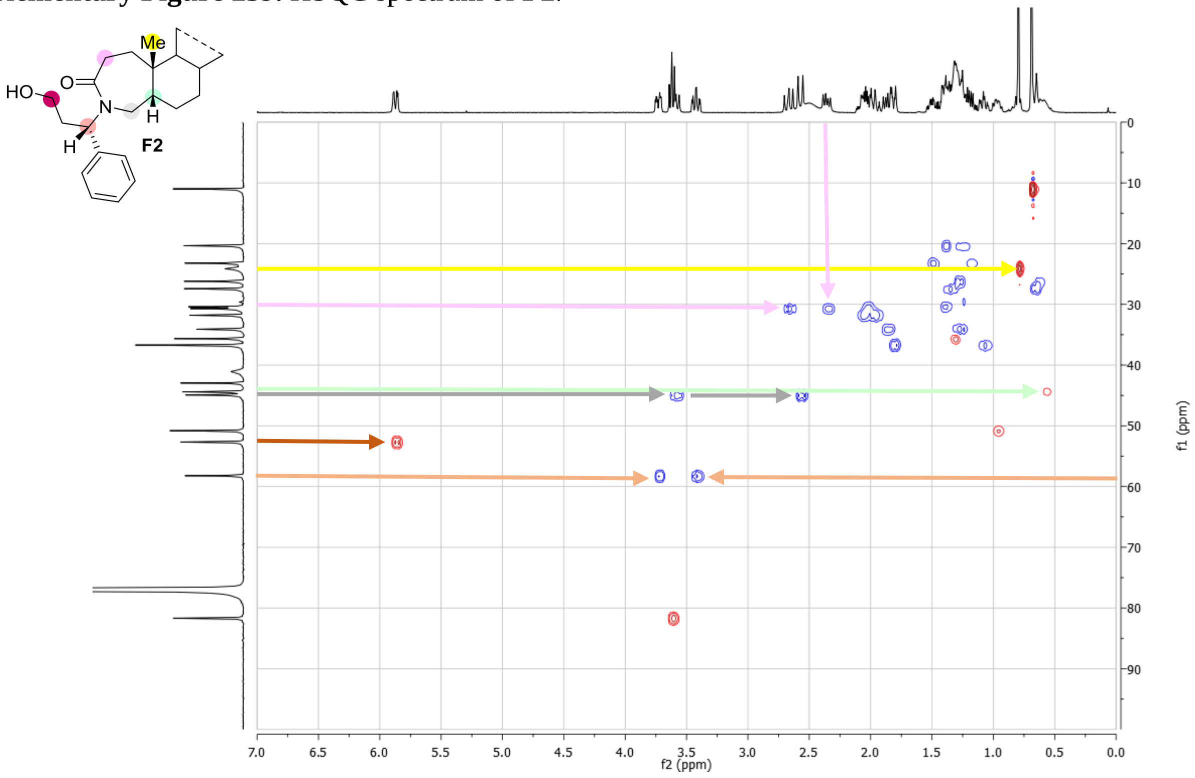

Supplementary Figure 260. HMBC spectrum of **F2**.

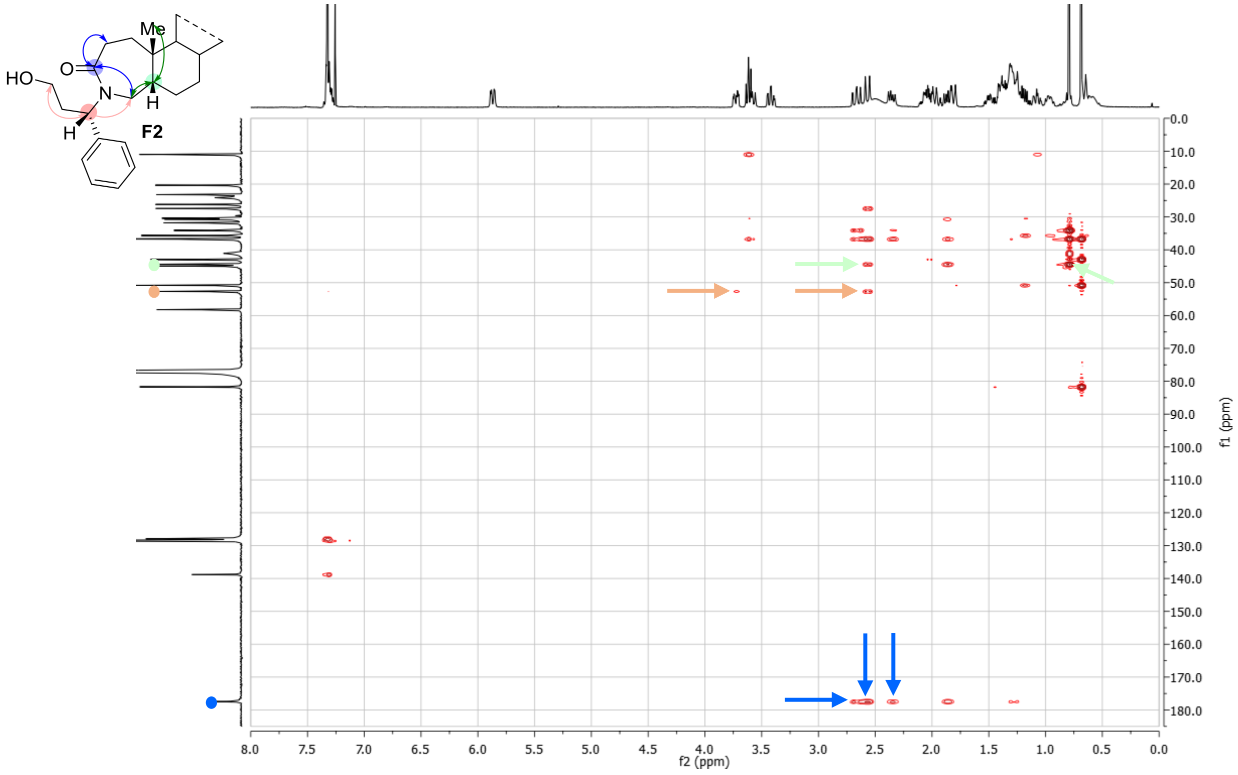

Supplementary Figure 261. ^{1}H NMR spectrum in $CDCl_3$, 400 MHz, of **F3**.

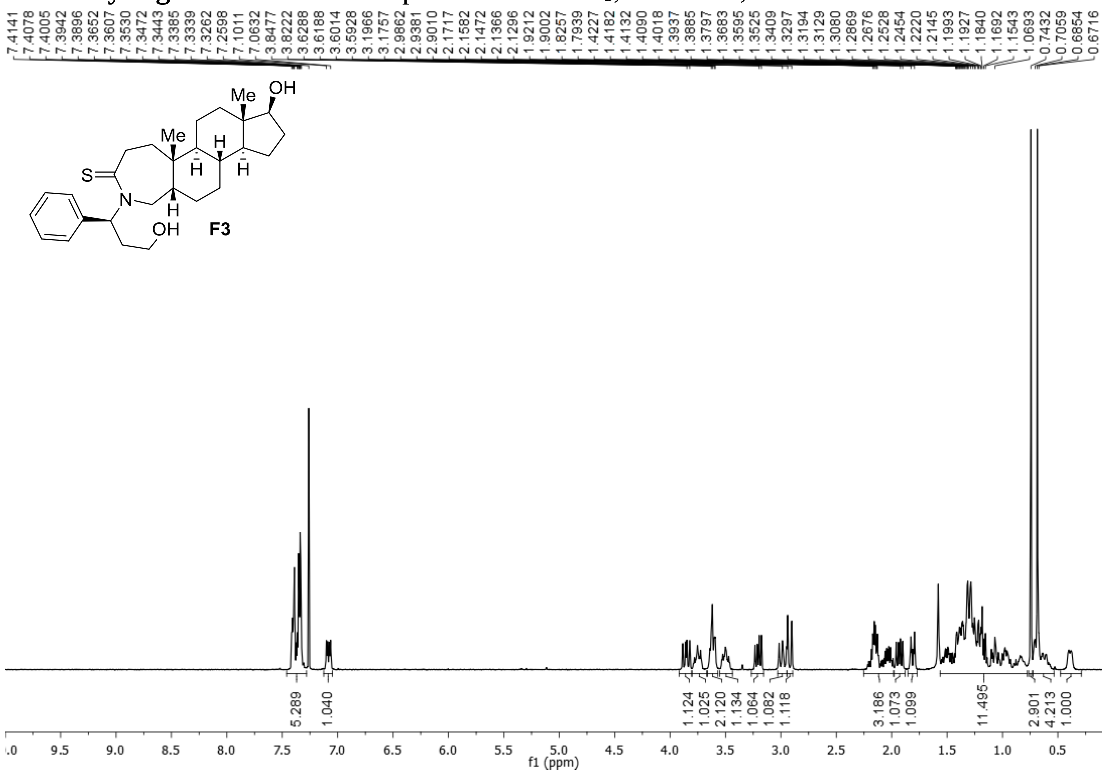

Supplementary Figure 262. ^{13}C NMR spectrum in $CDCl_3$, 151 MHz, of **F3**.

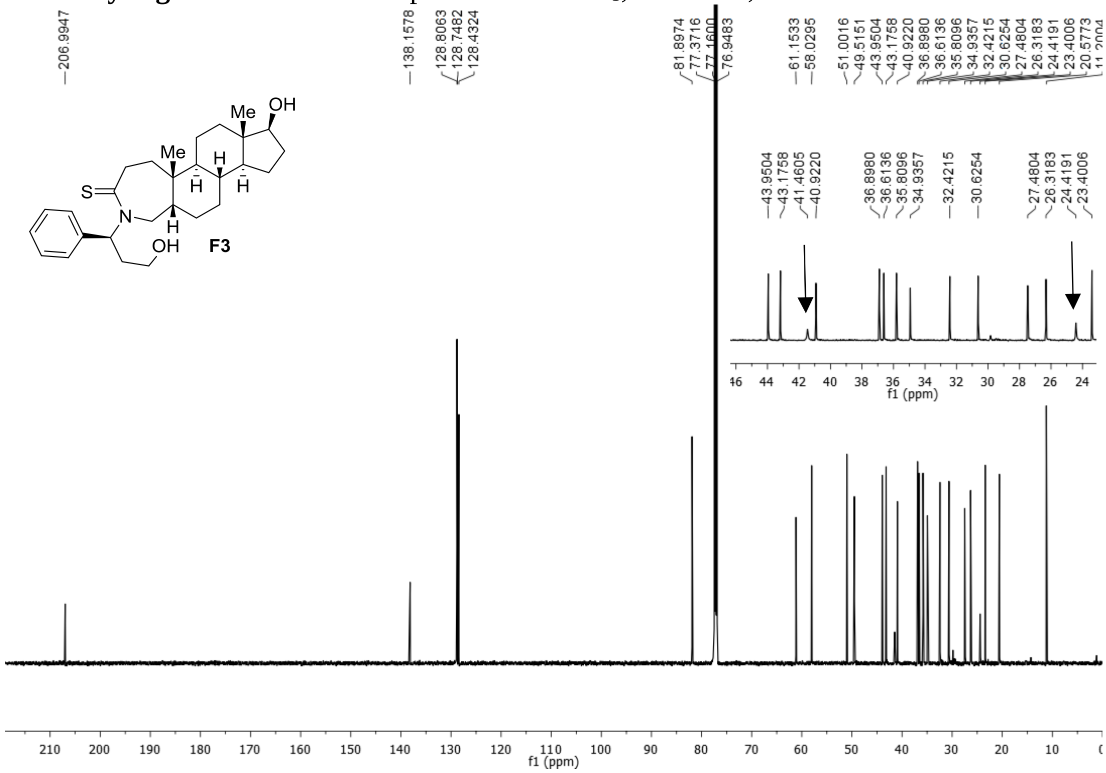

Supplementary Figure 263. ^{1}H NMR spectrum in $CDCl_3$, 400 MHz, of **F4**.

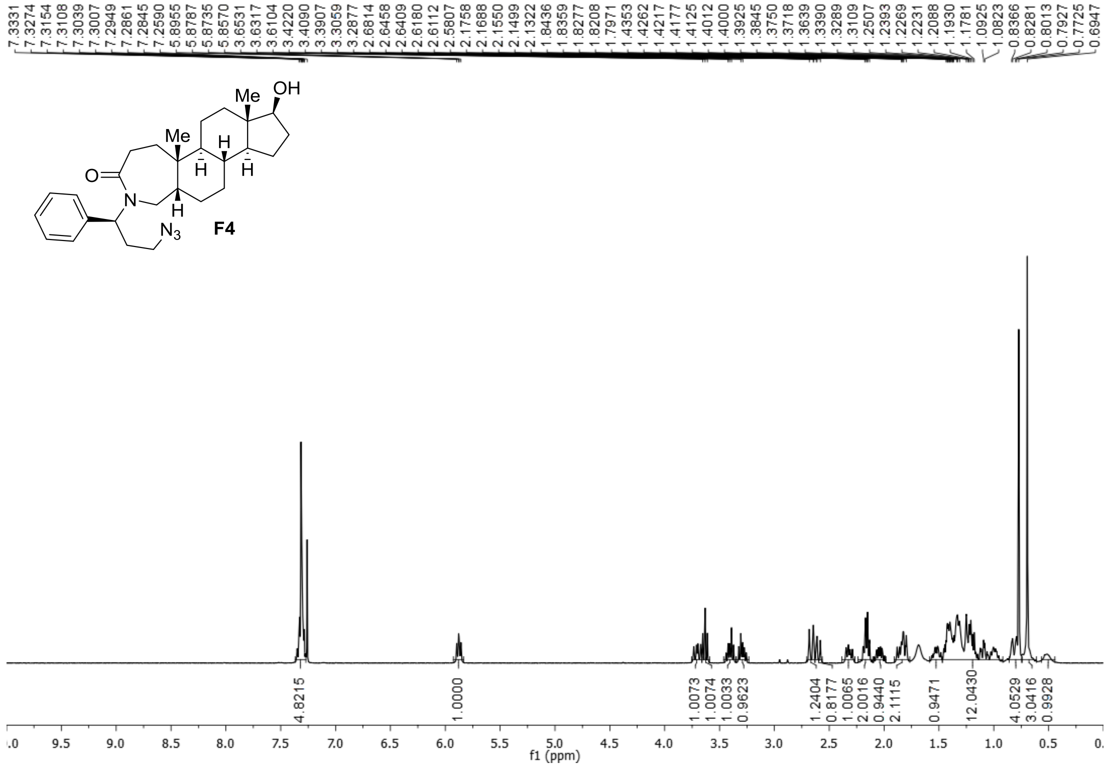

Supplementary Figure 264. ^{13}C NMR spectrum in $CDCl_3$, 101 MHz, of **F4**.

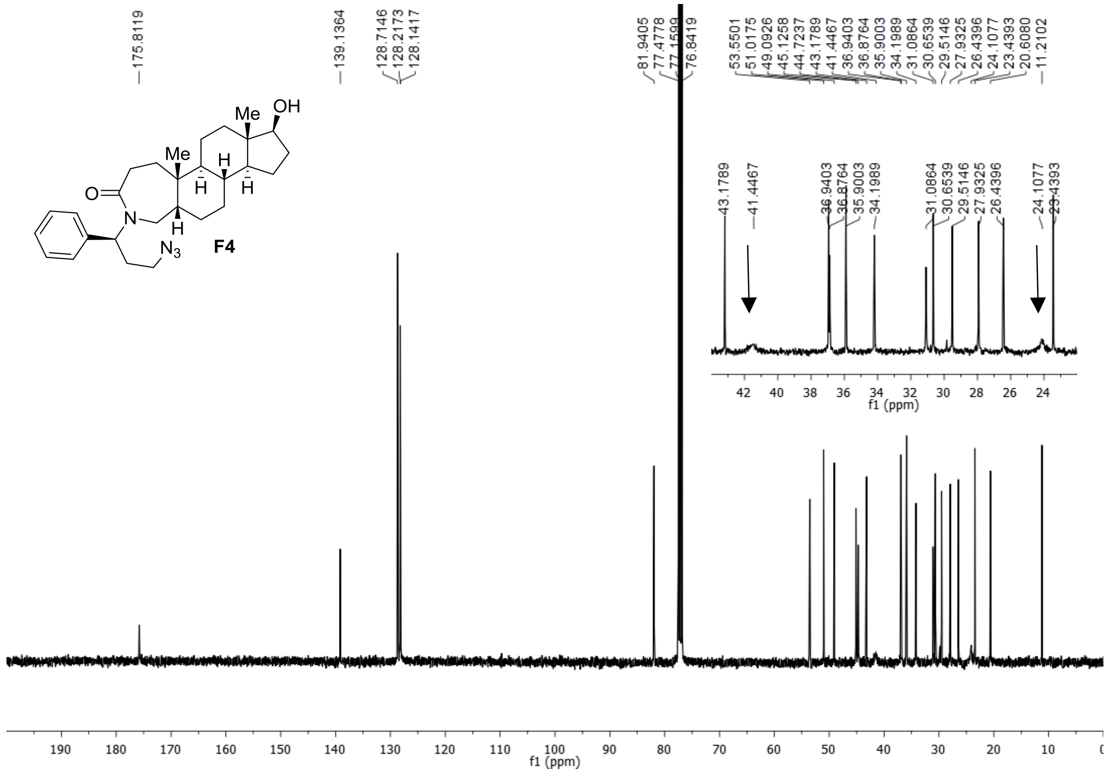

Supplementary Figure 265. ^{1}H NMR spectrum in $CDCl_3$, 400 MHz, of **F5**.

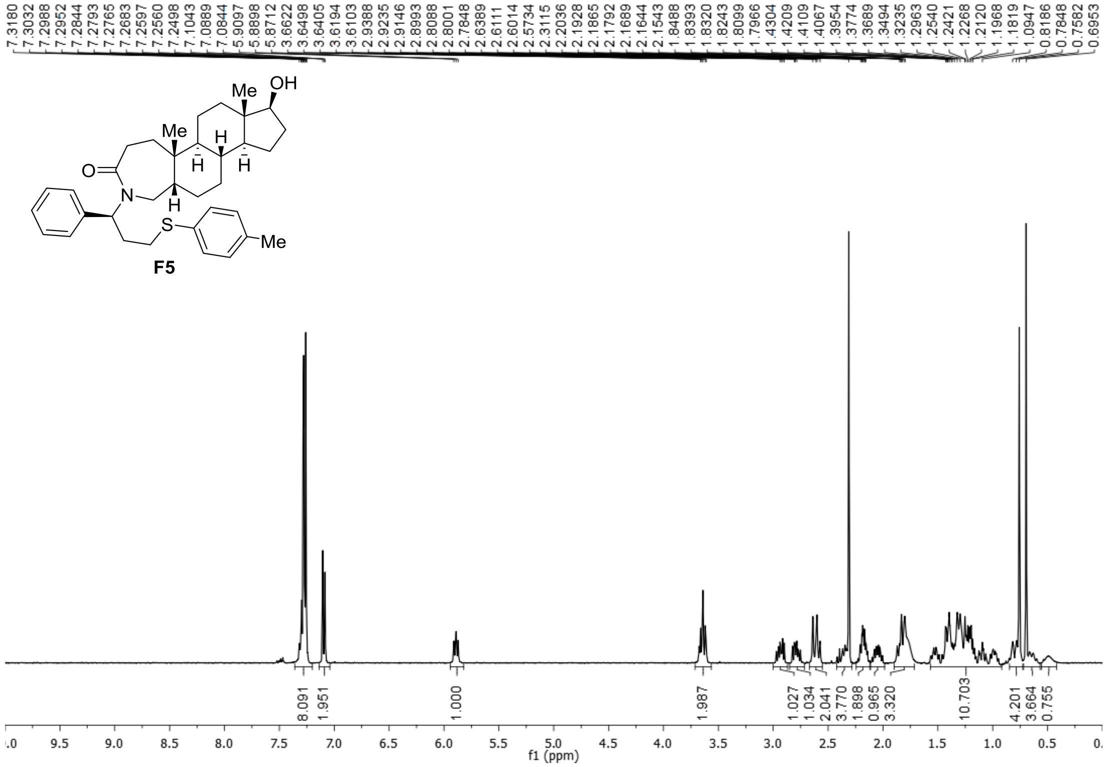

Supplementary Figure 266. ^{13}C NMR spectrum in $CDCl_3$, 101 MHz, of **F5**.

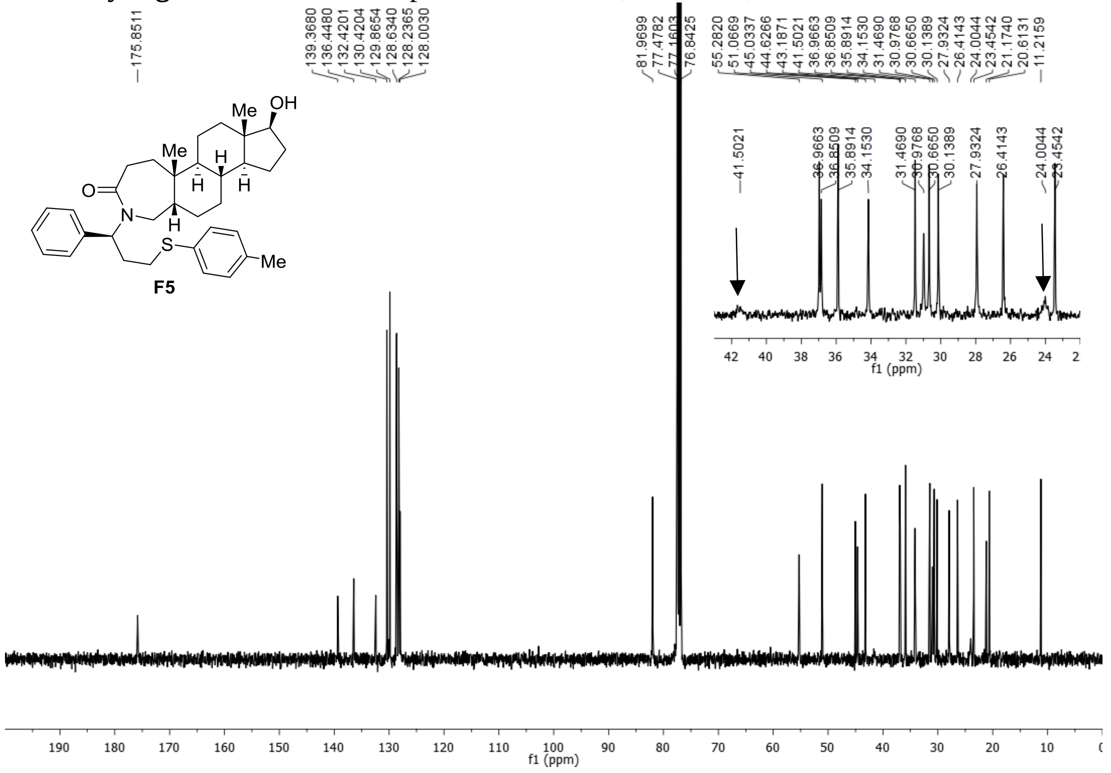

Supplementary Figure 267. ^{1}H NMR spectrum in $CDCl_3$, 400 MHz, of **S12**.

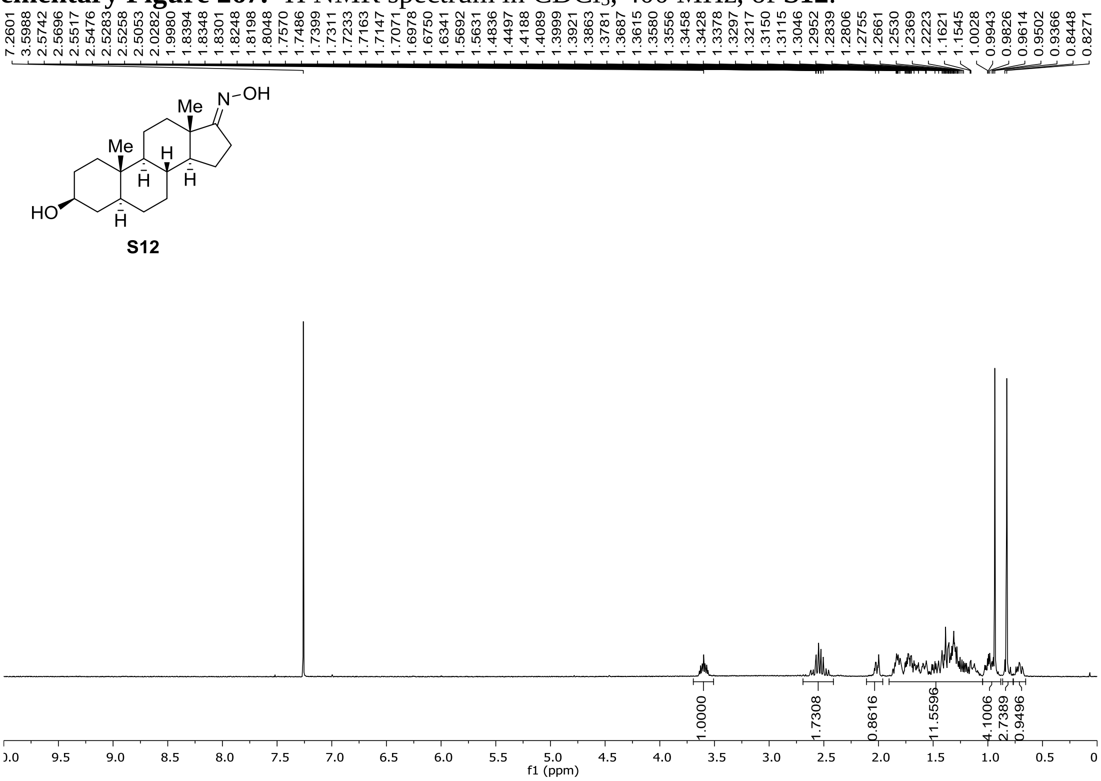

Supplementary Figure 268. ^{13}C NMR spectrum in $CDCl_3$, 101 MHz, of **S12**.

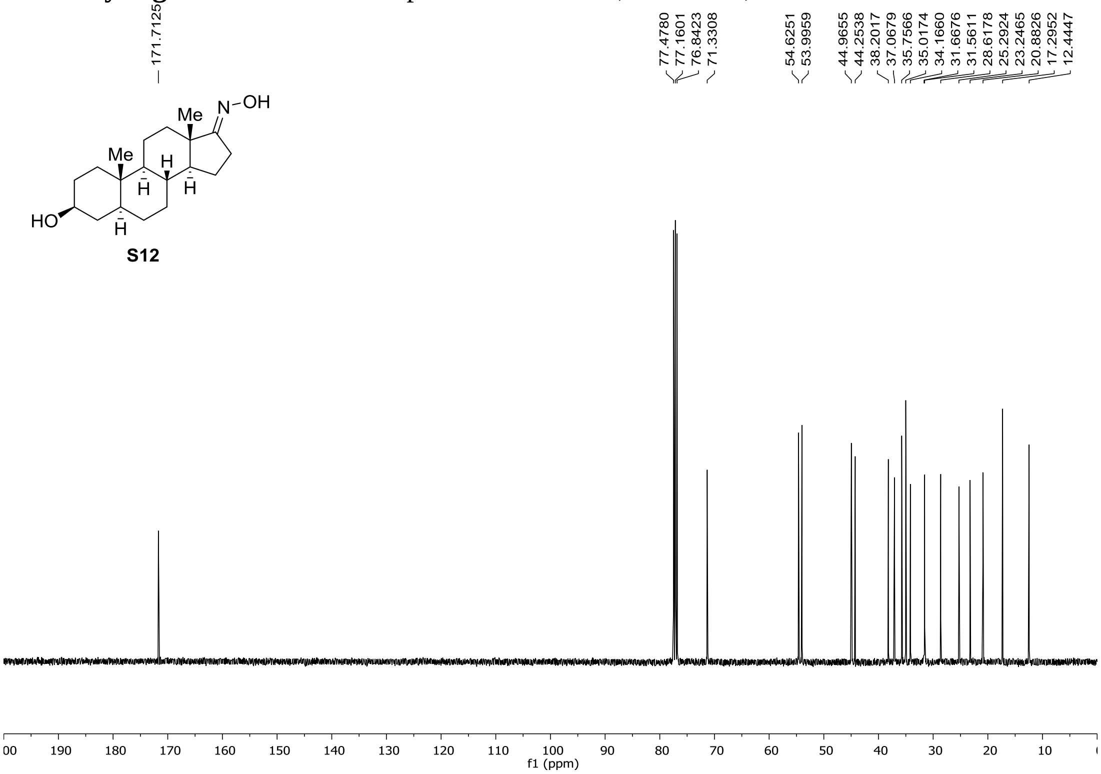

Supplementary Figure 269. ^{1}H NMR spectrum in $CDCl_3$, 400 MHz, of **Beck1**.

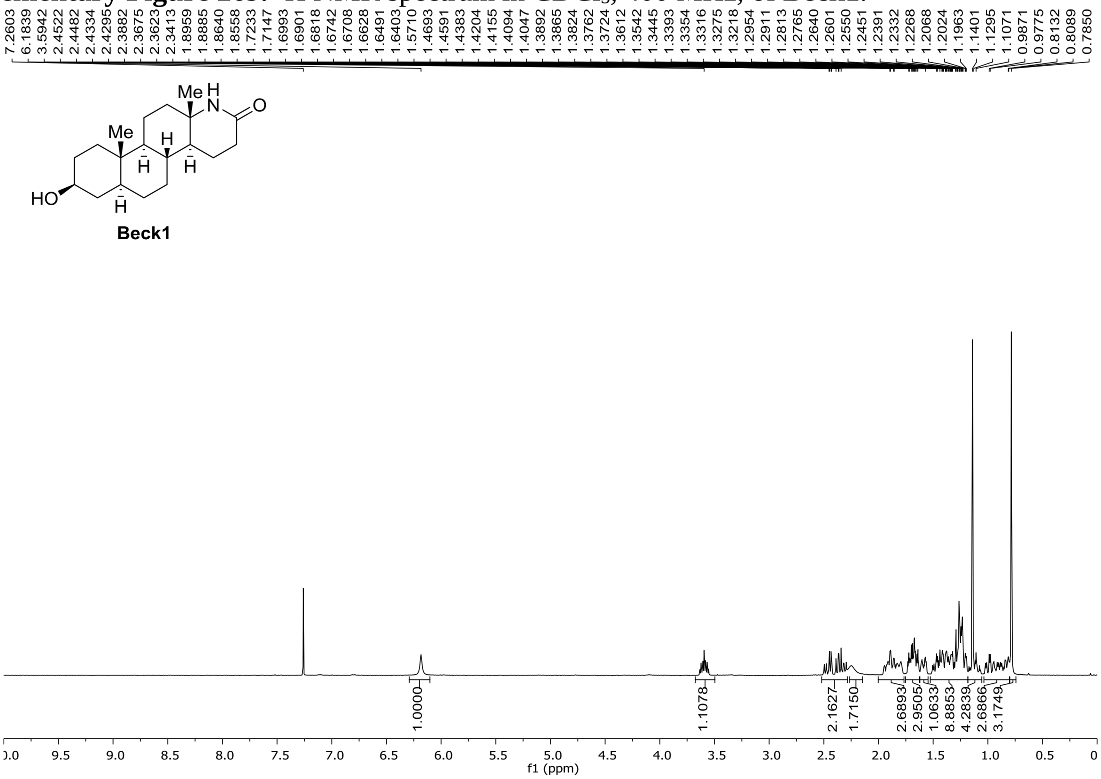

Supplementary Figure 270. ^{13}C NMR spectrum in $CDCl_3$, 101 MHz, of **Beck1**.

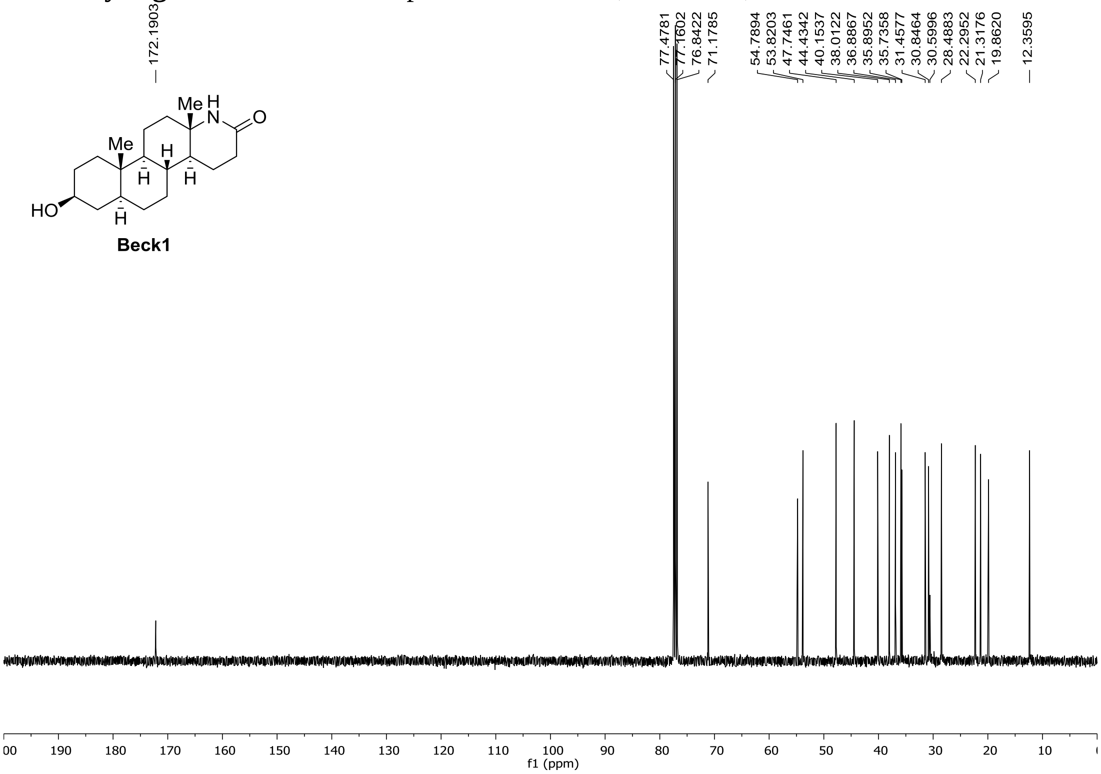

Supplementary Figure 271. ^{1}H NMR spectrum in $CDCl_3$, 400 MHz, of **S13**.

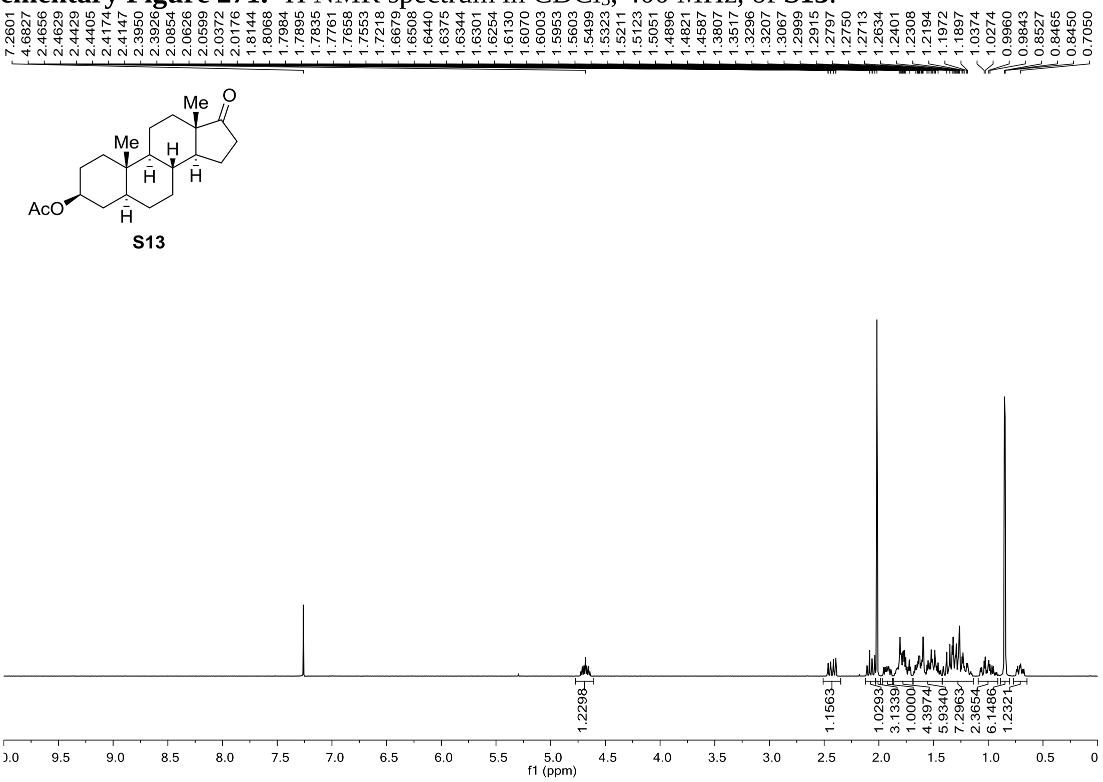

Supplementary Figure 272. ^{13}C NMR spectrum in $CDCl_3$, 101 MHz, of **S13**.

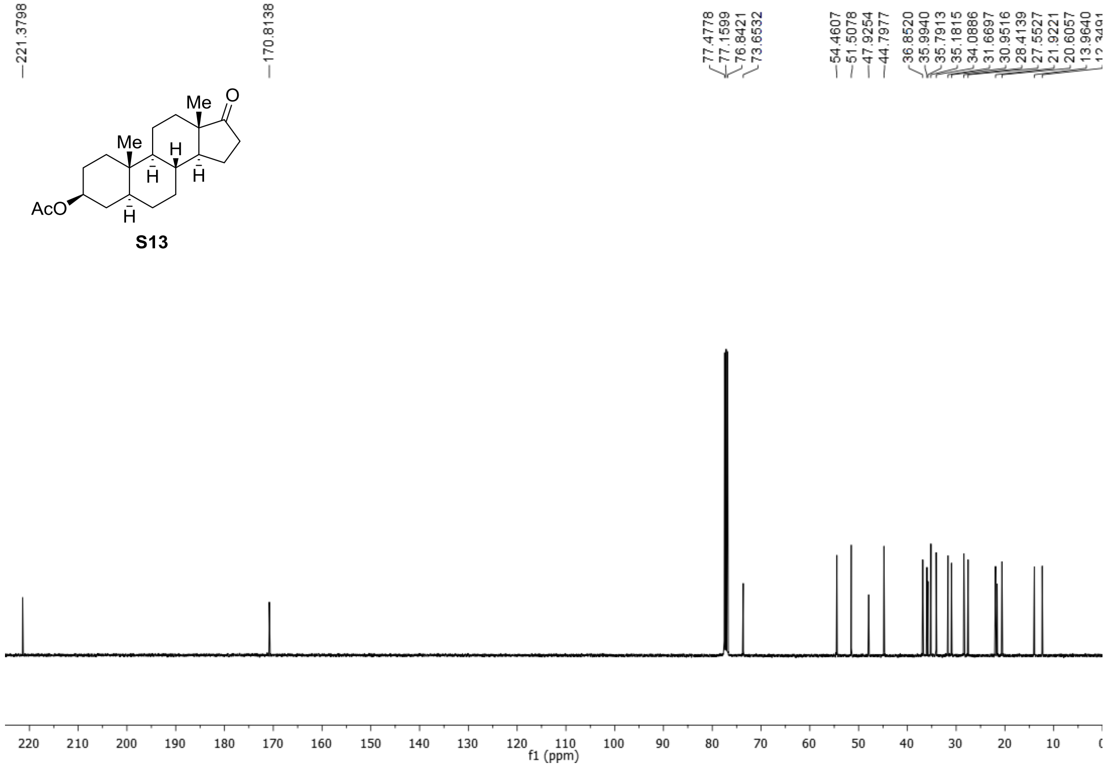

Supplementary Figure 273. ^{1}H NMR spectrum in $CDCl_3$, 400 MHz, of **S14**.

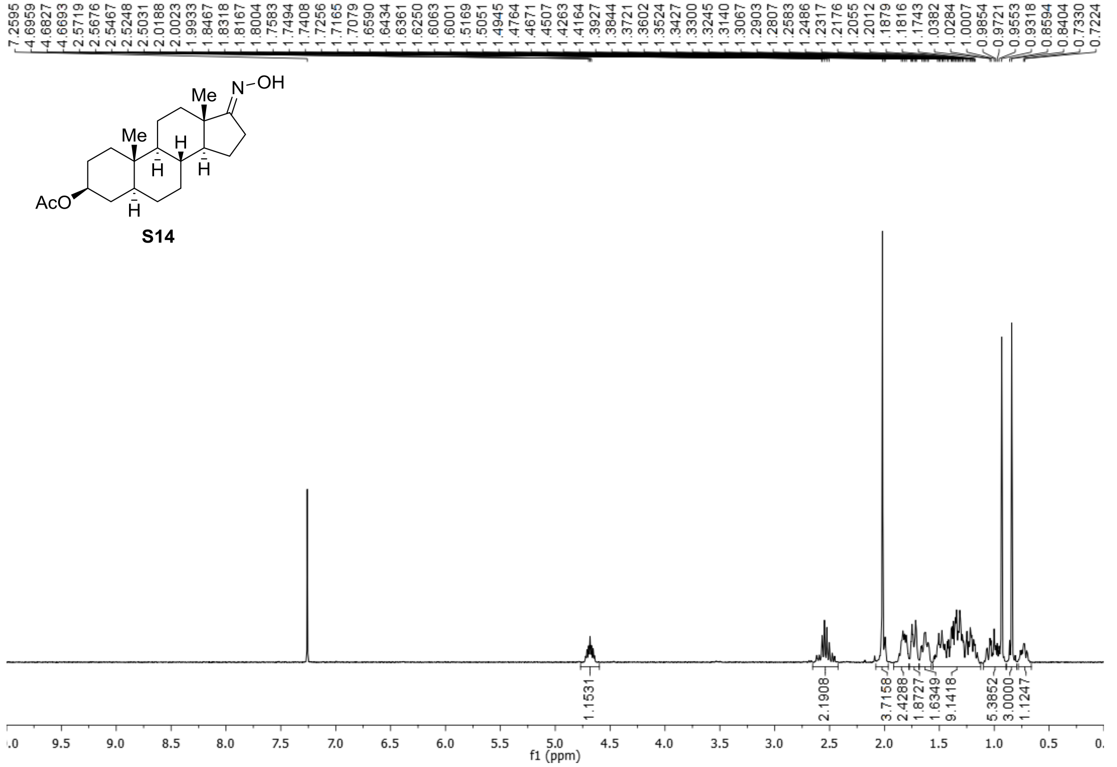

Supplementary Figure 274. ^{13}C NMR spectrum in $CDCl_3$, 101 MHz, of **S14**.

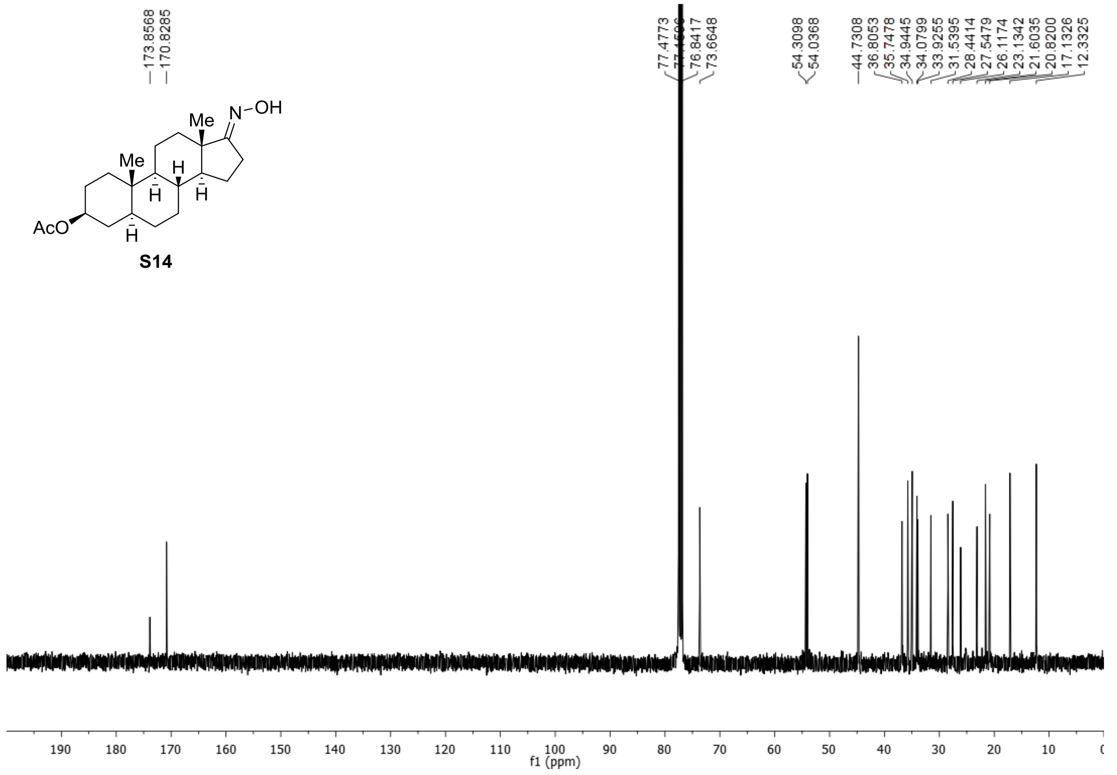

Supplementary Figure 275. ^{1}H NMR spectrum in $CDCl_3$, 400 MHz, of **Beck2**.

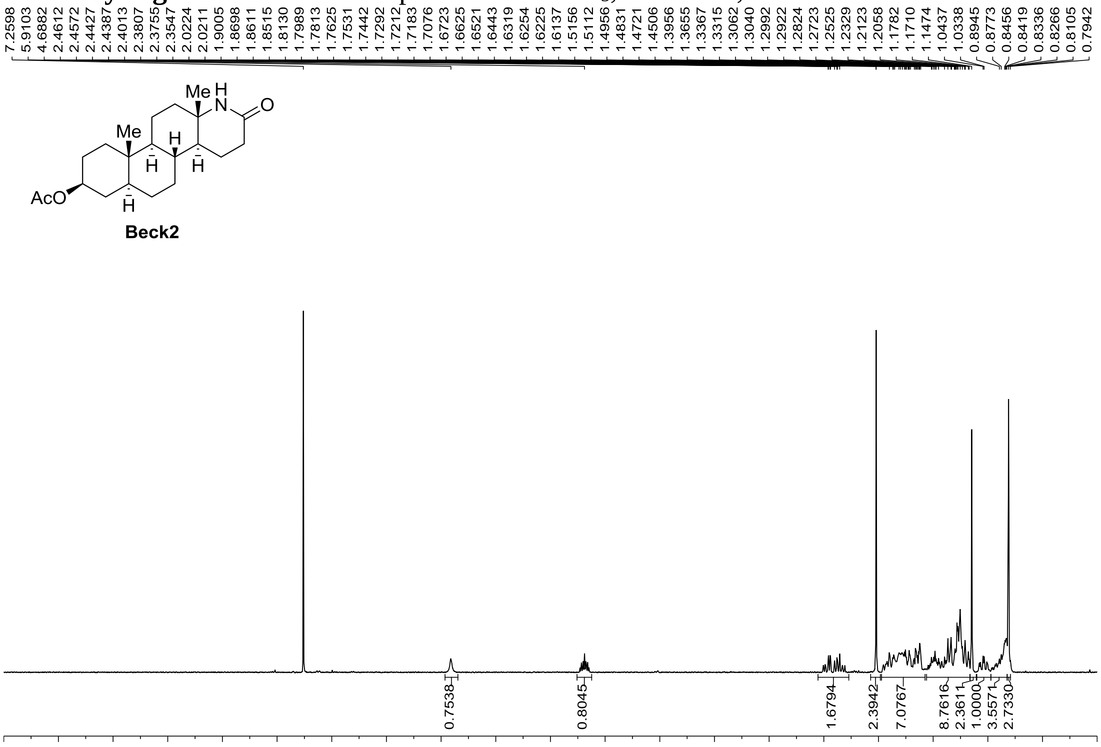

Supplementary Figure 276. ^{13}C NMR spectrum in $CDCl_3$, 101 MHz, of **Beck2**.

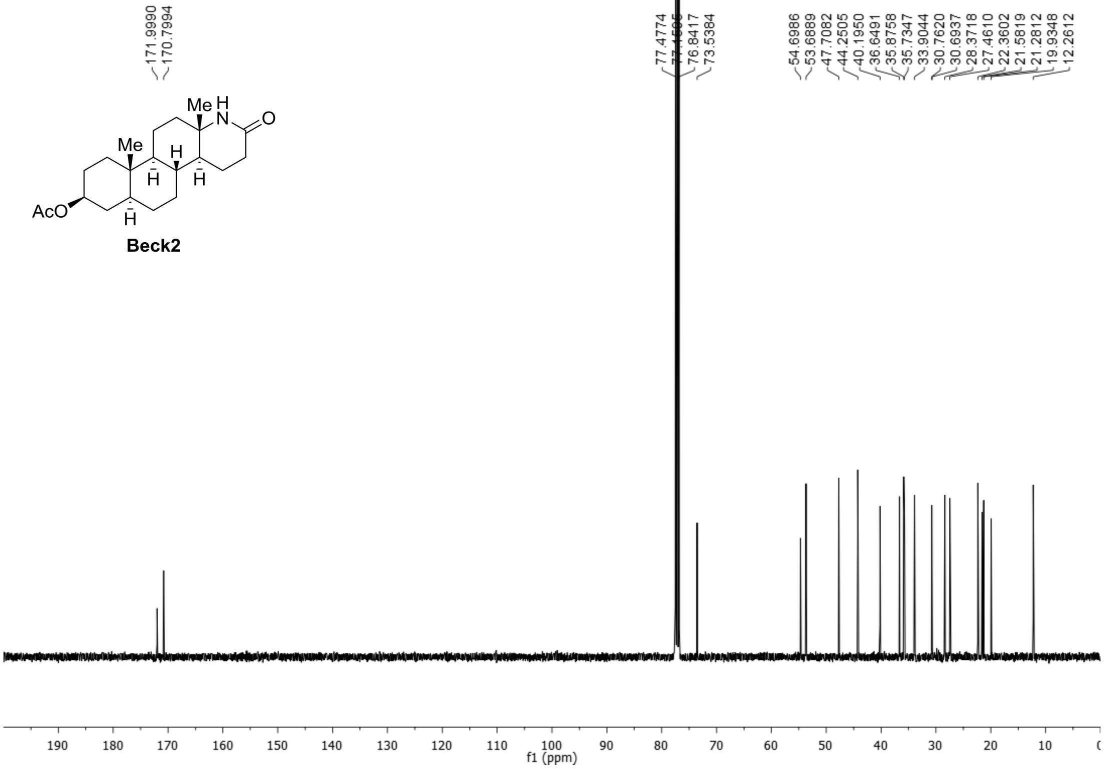

Supplementary Figure 277. ^{1}H NMR spectrum in $CDCl_3$, 400 MHz, of **S15**.

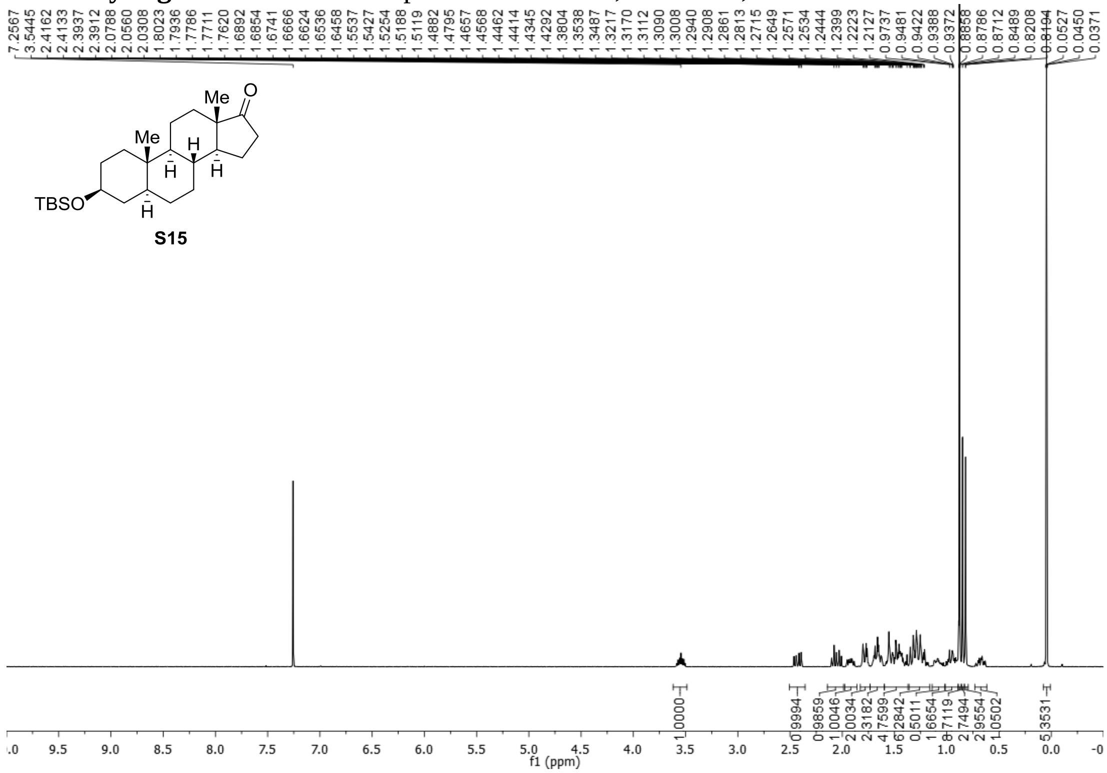

Supplementary Figure 278. ^{13}C NMR spectrum in $CDCl_3$, 101 MHz, of **S15**.

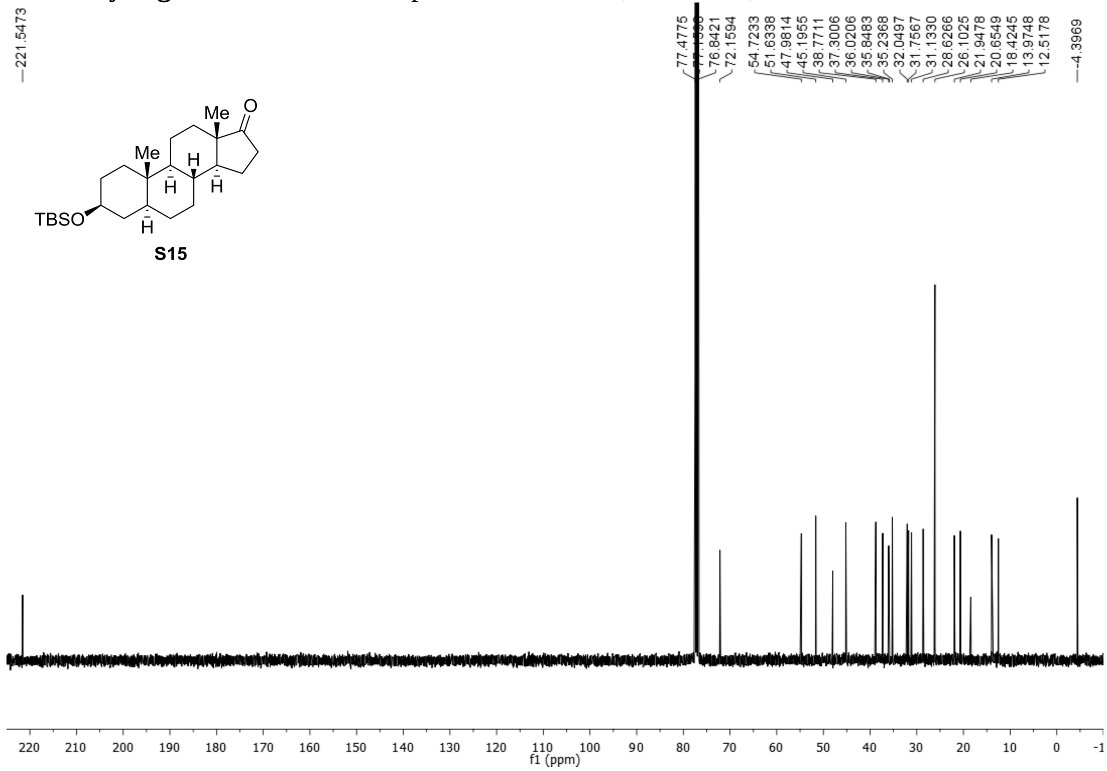

Supplementary Figure 279. ^{1}H NMR spectrum in $CDCl_3$, 400 MHz, of **S16**.

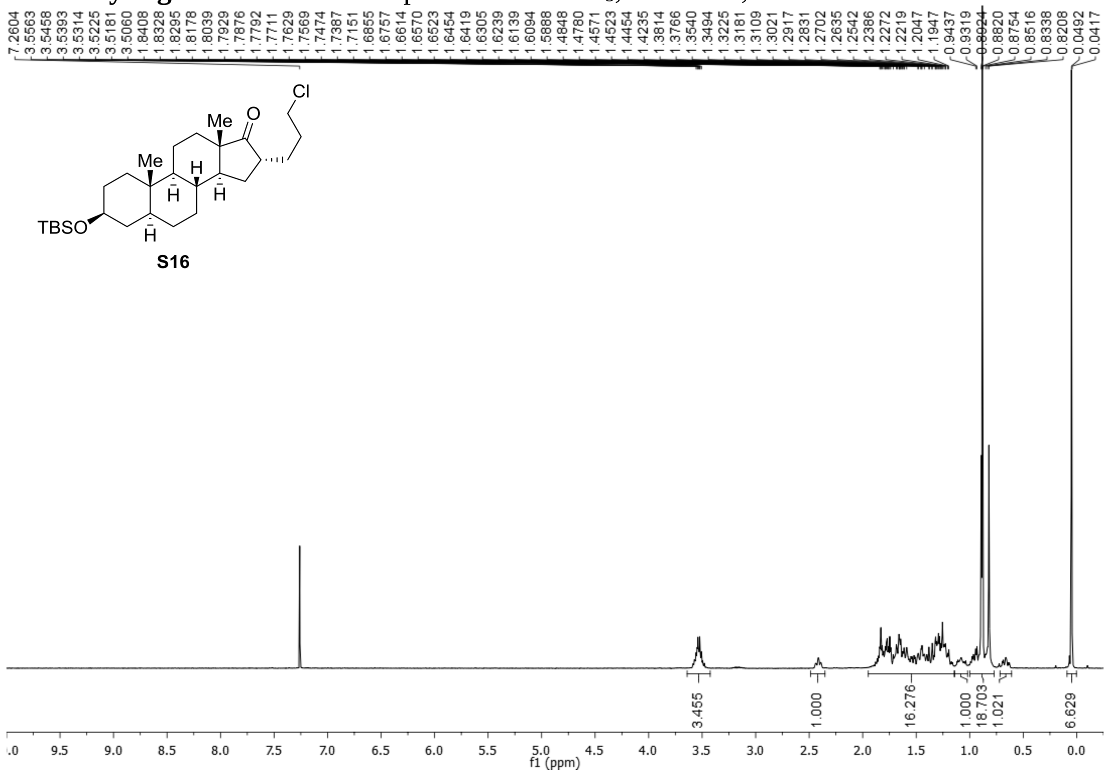

Supplementary Figure 280. ^{13}C NMR spectrum in $CDCl_3$, 101 MHz, of **S16**.

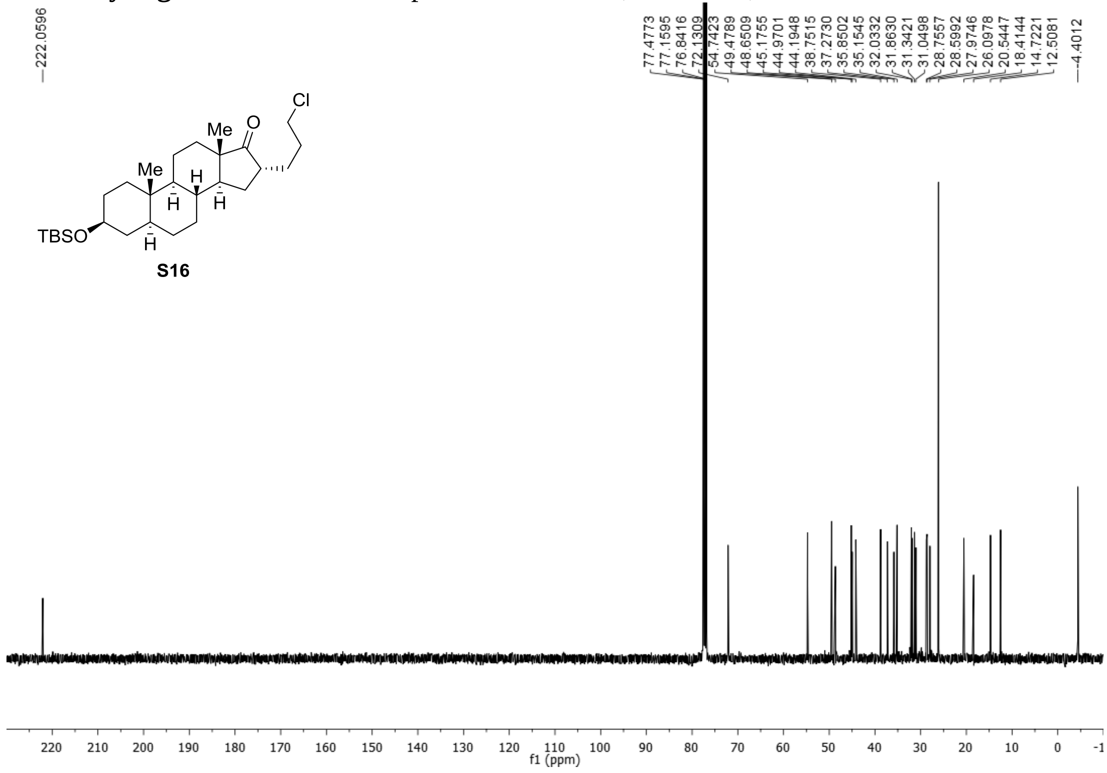

Supplementary Figure 281. HSQC spectrum of **S16**.

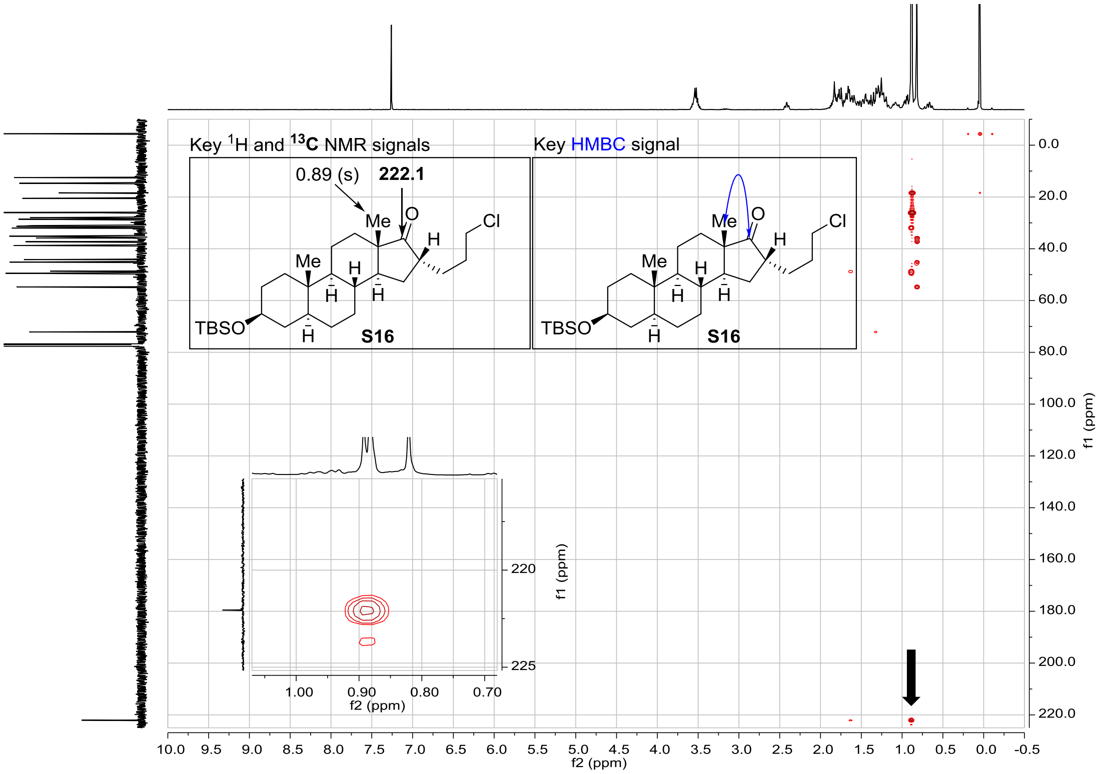

Supplementary Figure 282. 1D NOE spectrum in $CDCl_3$, 400 MHz, of **S16**.

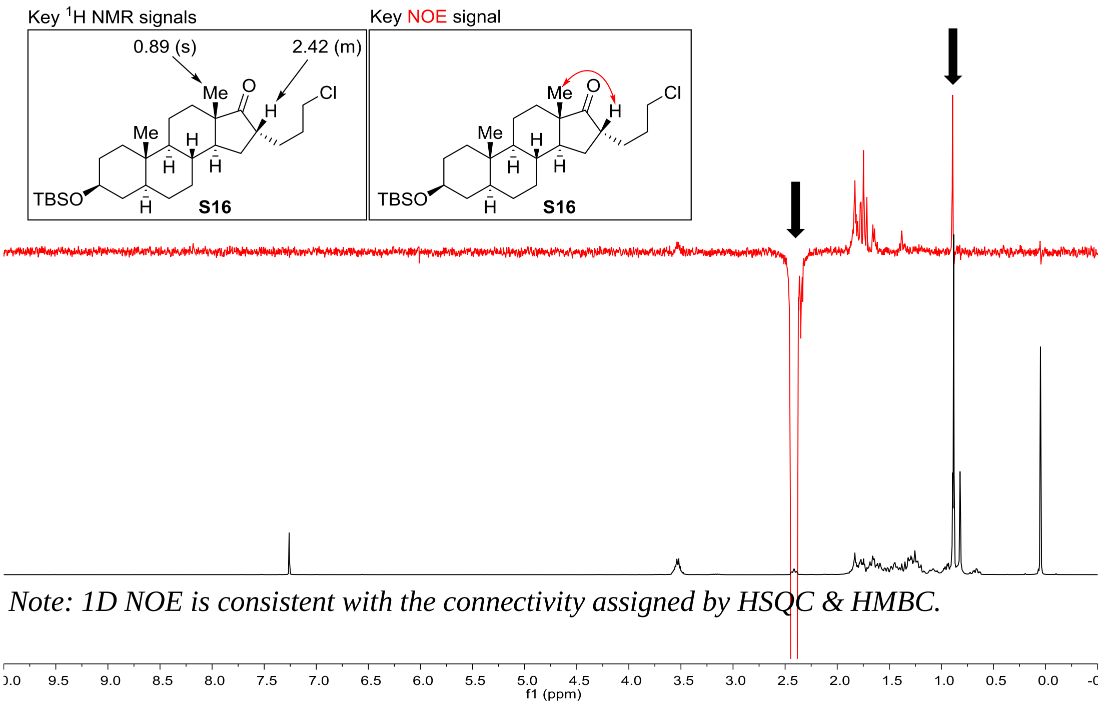

Note: 1D NOE is consistent with the connectivity assigned by HSQC & HMBC.

Supplementary Figure 283. ^{1}H NMR spectrum in $CDCl_3$, 400 MHz, of **S17**.

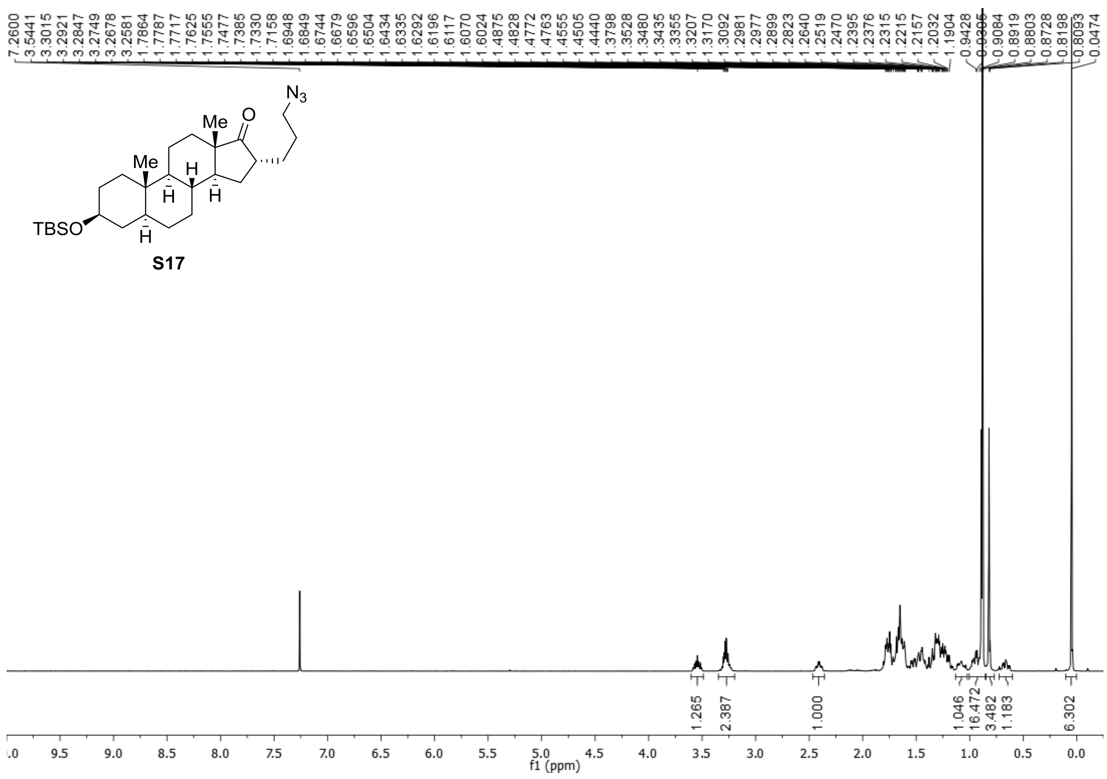

Supplementary Figure 284. ^{13}C NMR spectrum in $CDCl_3$, 101 MHz, of **S17**.

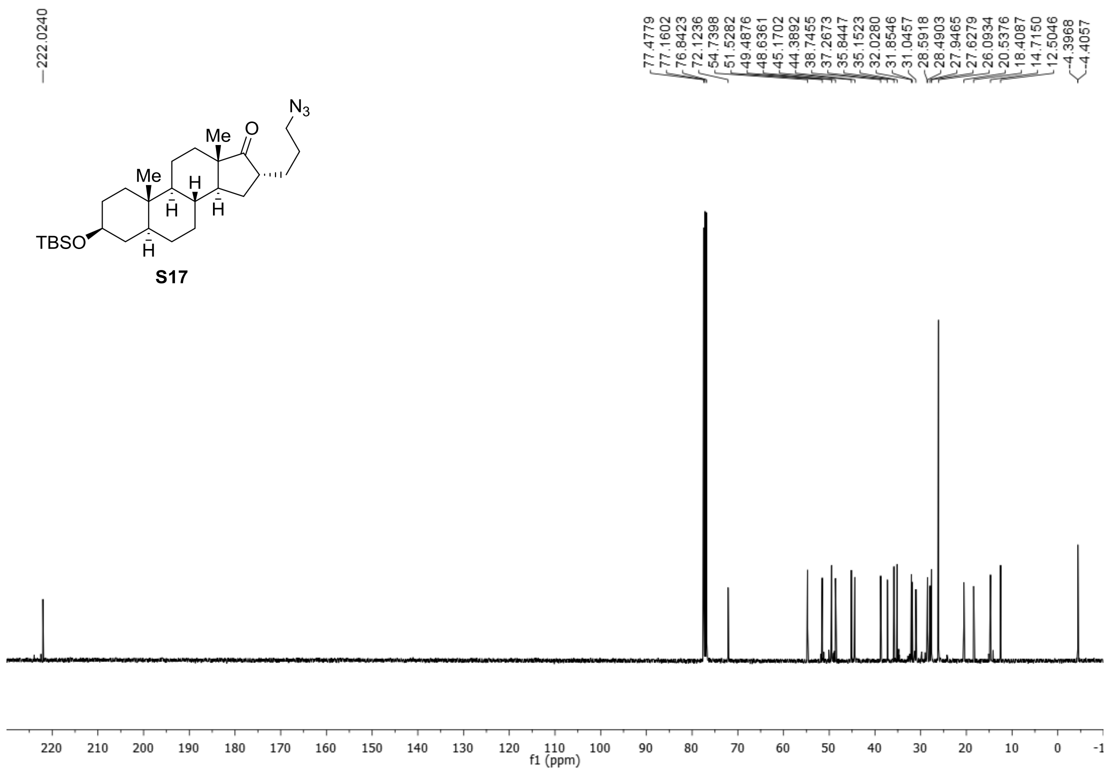

Supplementary Figure 285. ^{1}H NMR spectrum in $CDCl_3$, 400 MHz, of **Intra1**.

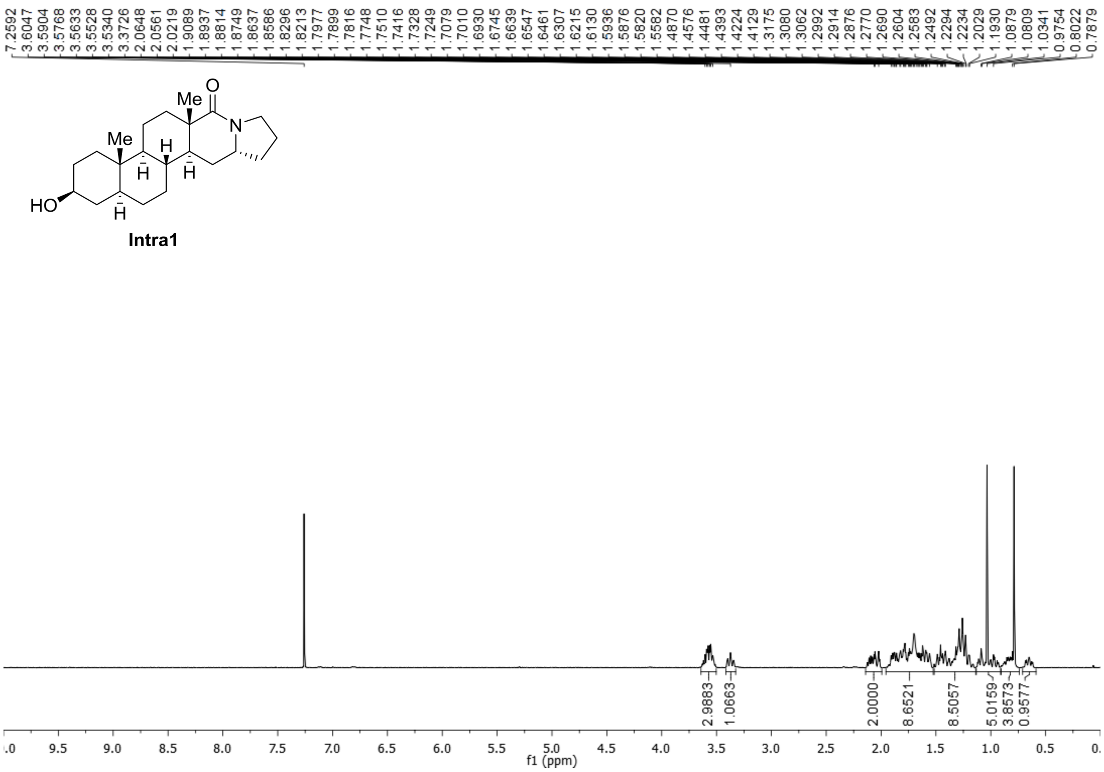

Supplementary Figure 286. ^{13}C NMR spectrum in $CDCl_3$, 101 MHz, of **Intra1**.

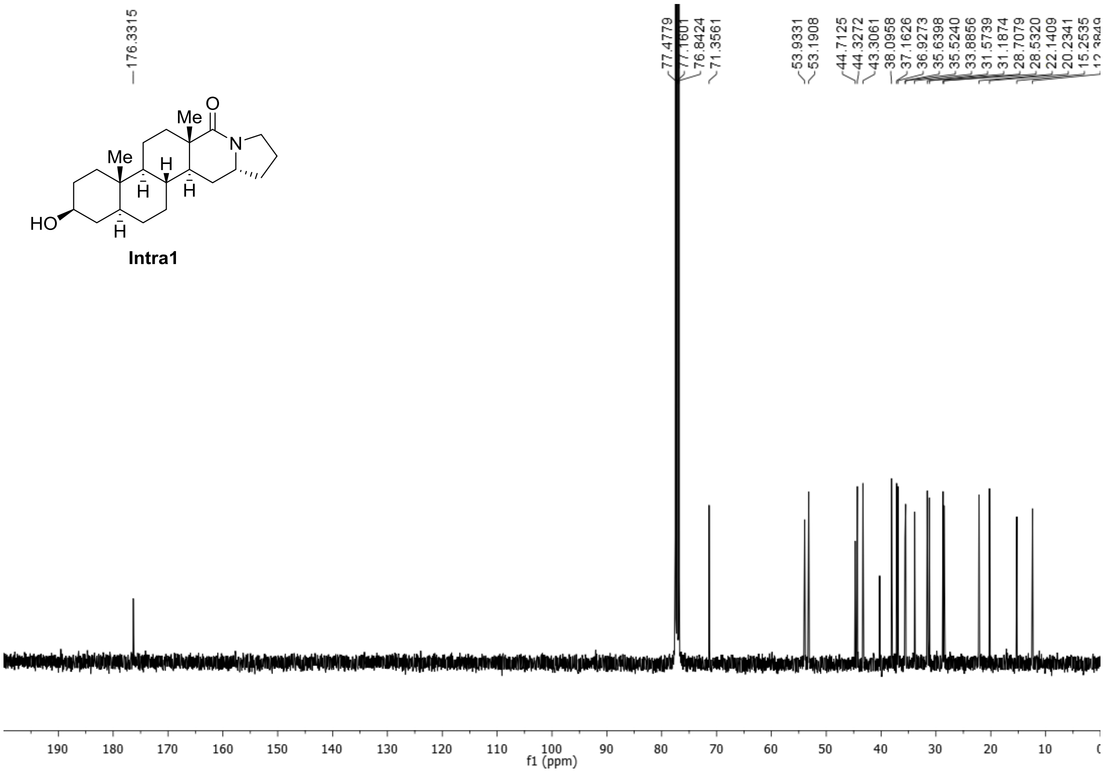

Supplementary Figure 287. ^{1}H NMR spectrum in $CDCl_3$, 400 MHz, of **5b**.

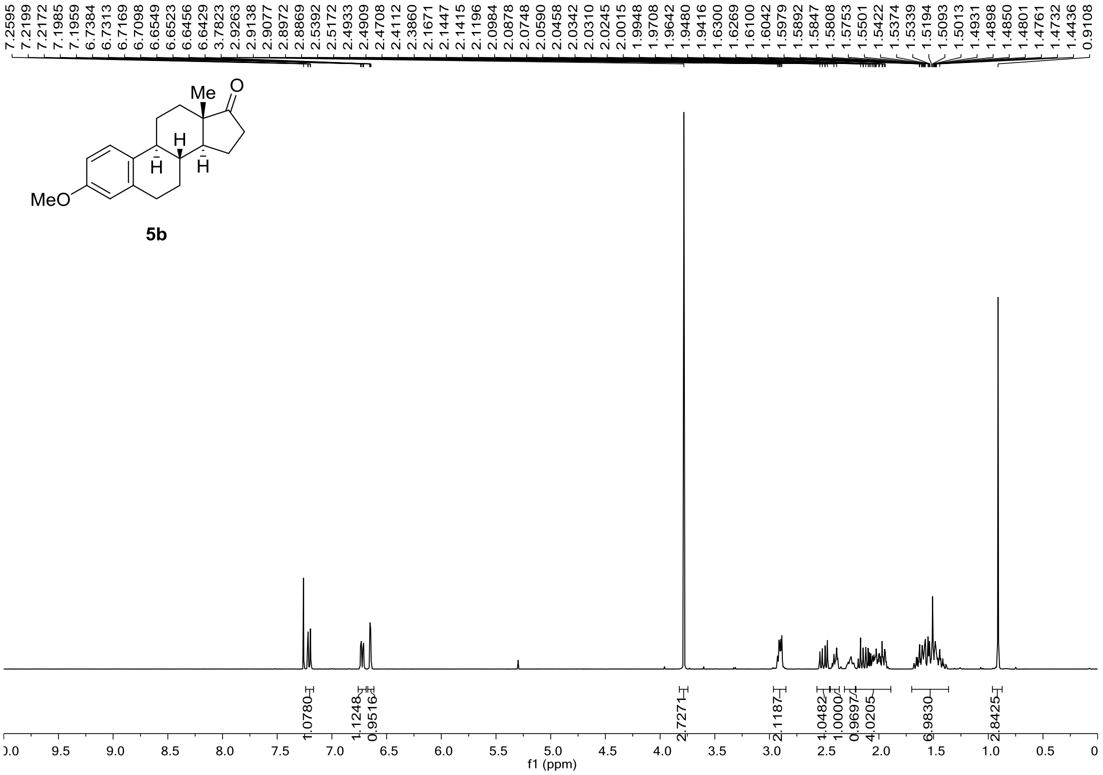

Supplementary Figure 288. ^{13}C NMR spectrum in $CDCl_3$, 101 MHz, of **5b**.

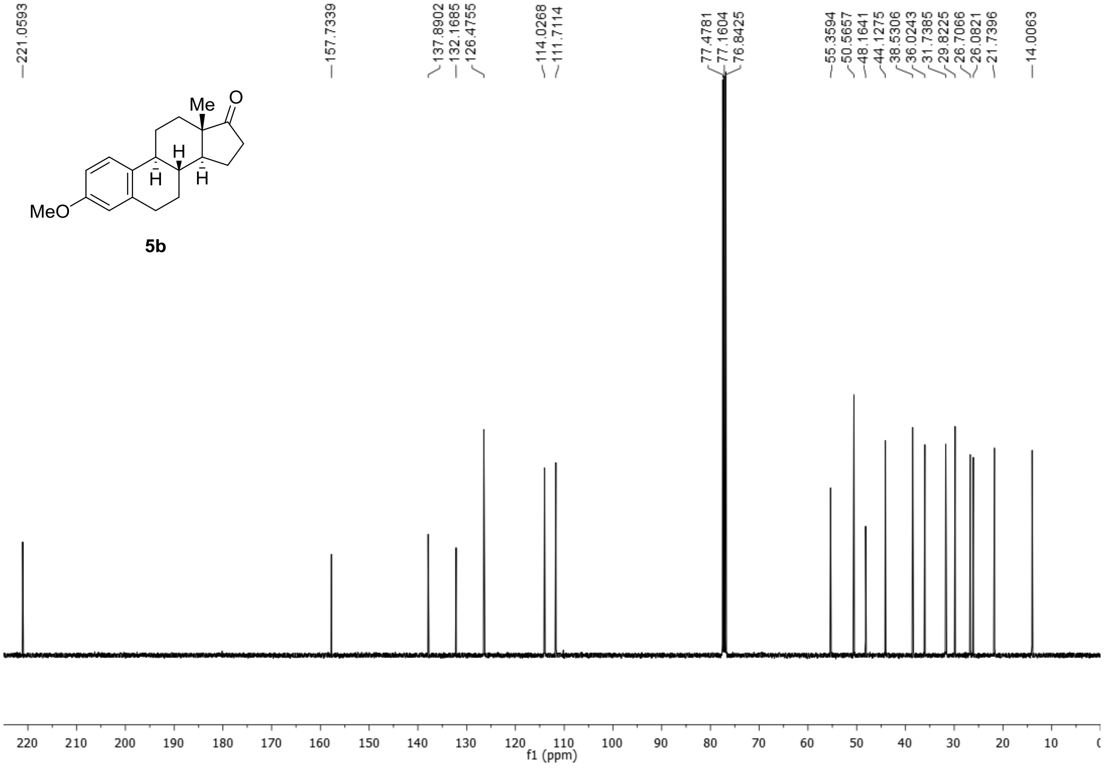

Supplementary Figure 289. ^{1}H NMR spectrum in $CDCl_3$, 400 MHz, of **S18**.

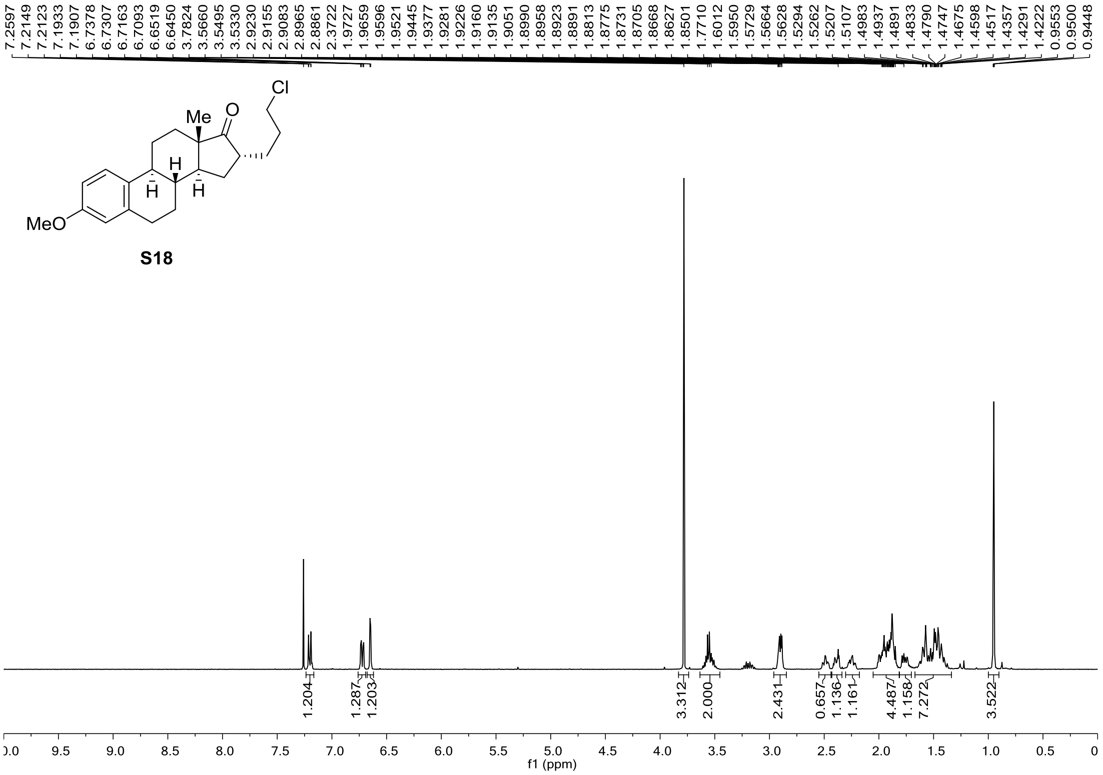

Supplementary Figure 290. ^{13}C NMR spectrum in $CDCl_3$, 101 MHz, of **S18**.

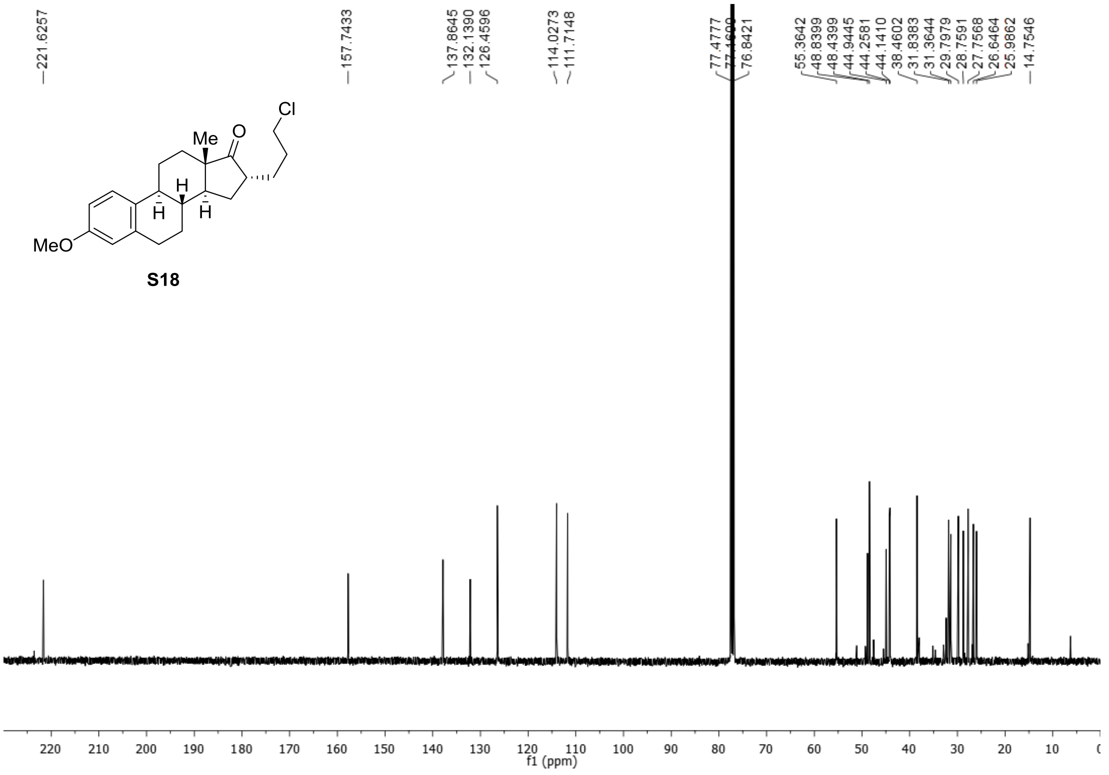

Supplementary Figure 291. HSQC spectrum of **S18**.

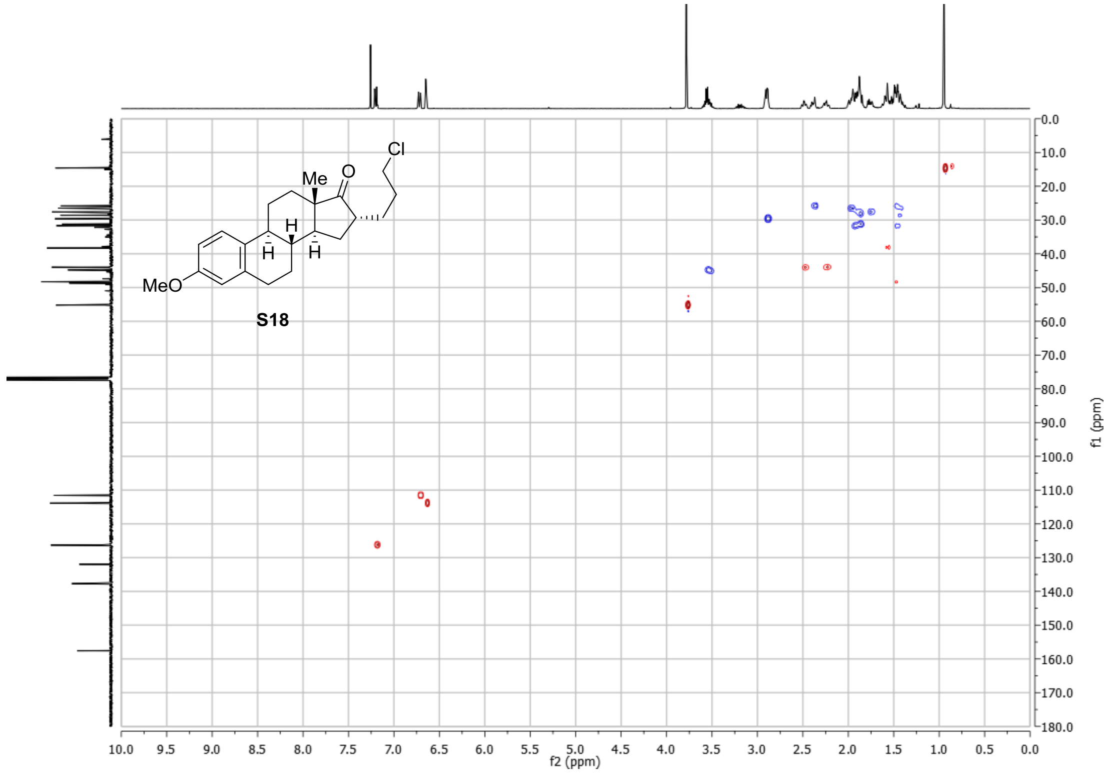

Supplementary Figure 292. ^{1}H NMR spectrum in $CDCl_3$, 400 MHz, of **S19**.

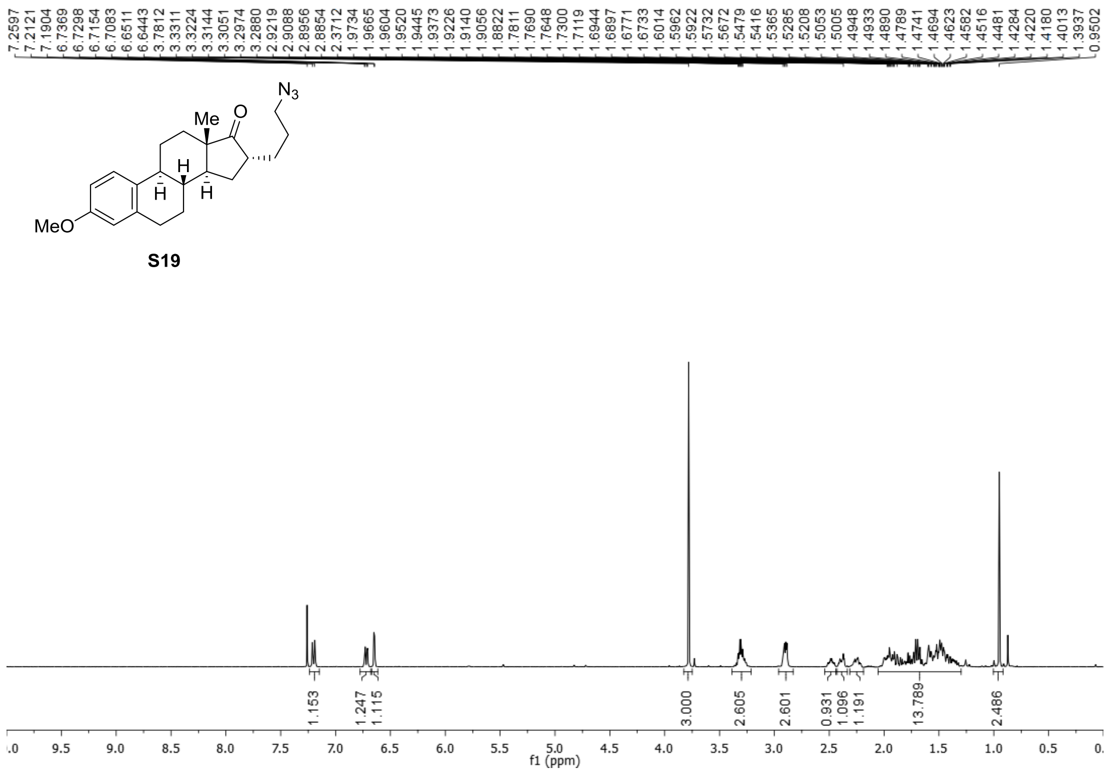

Supplementary Figure 293. ^{13}C NMR spectrum in $CDCl_3$, 101 MHz, of **S19**.

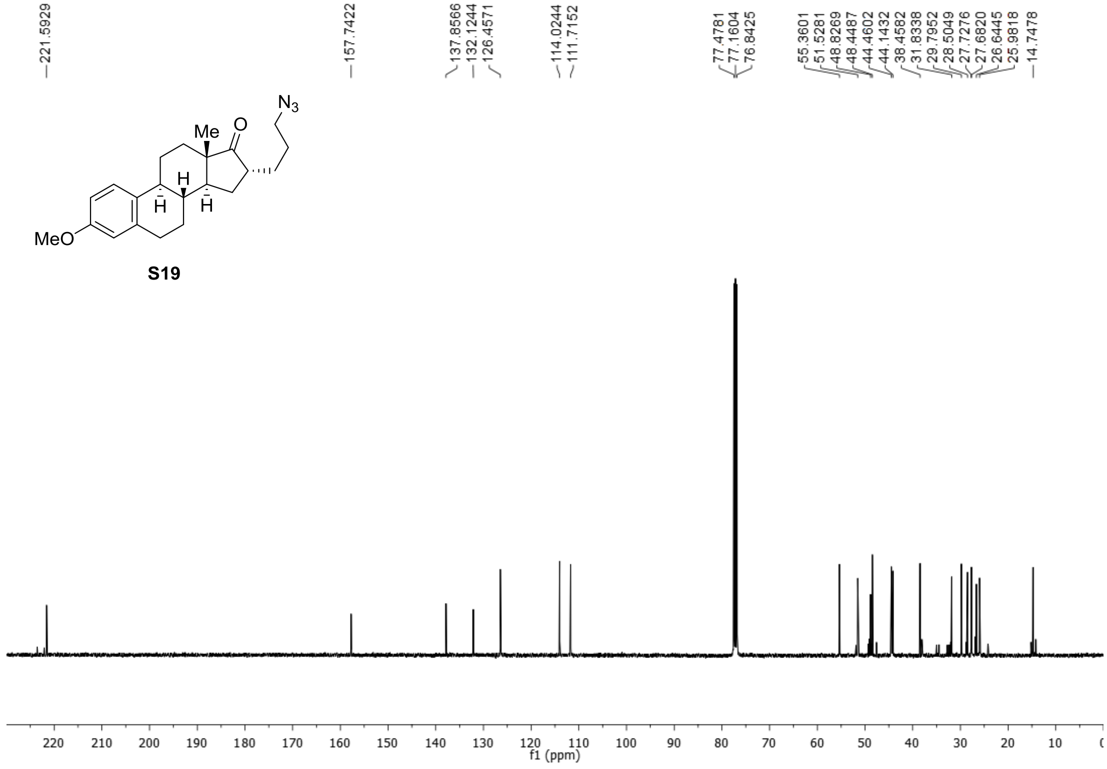

Supplementary Figure 294. HSQC spectrum of **S19**.

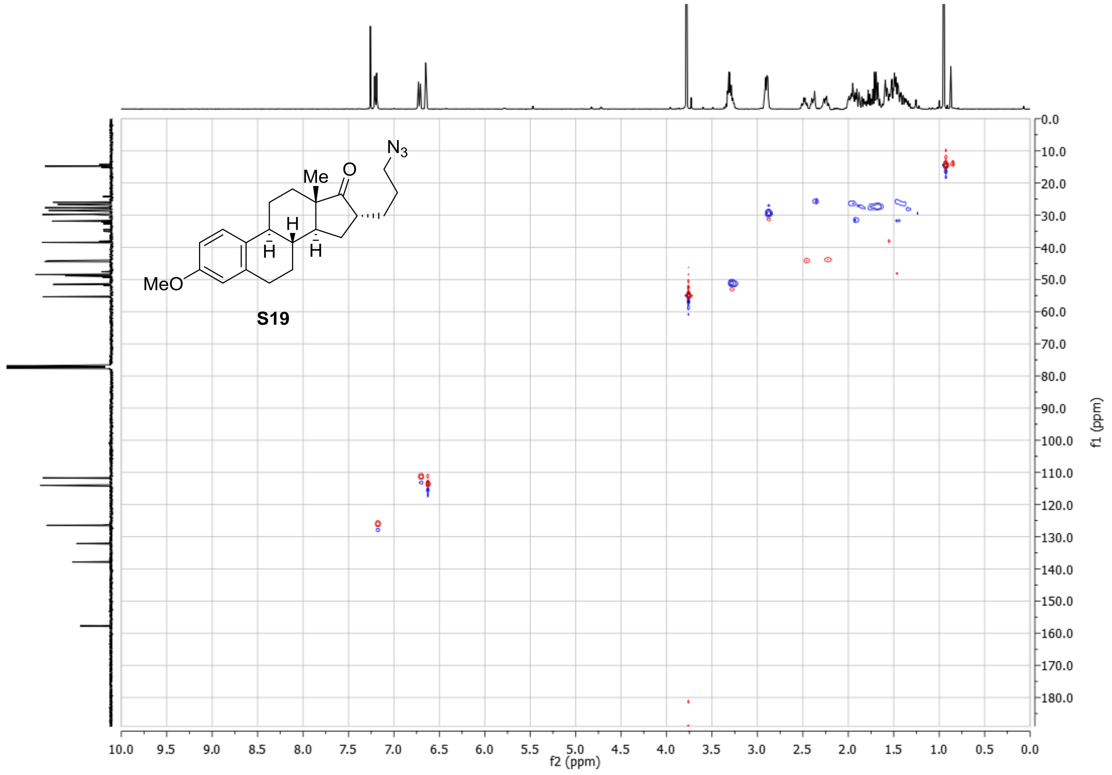

Supplementary Figure 295. 1D NOE spectrum in $CDCl_3$, 400 MHz, of **S19**.

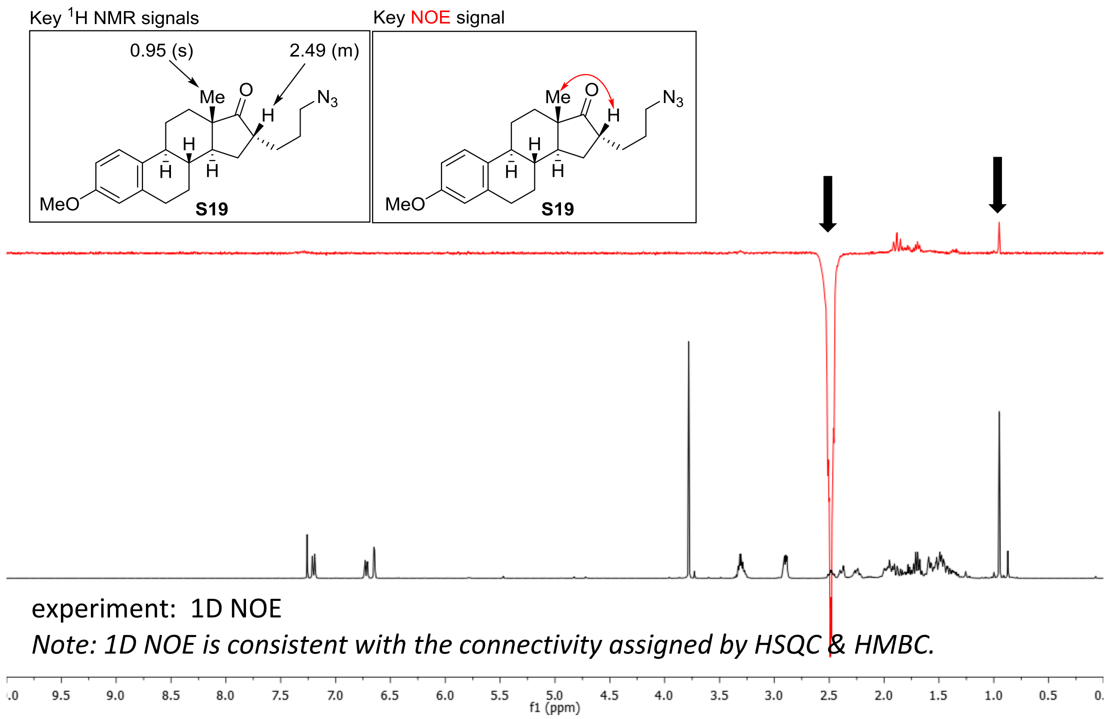

Supplementary Figure 296. ^{1}H NMR spectrum in $CDCl_3$, 400 MHz, of **Intra2**.

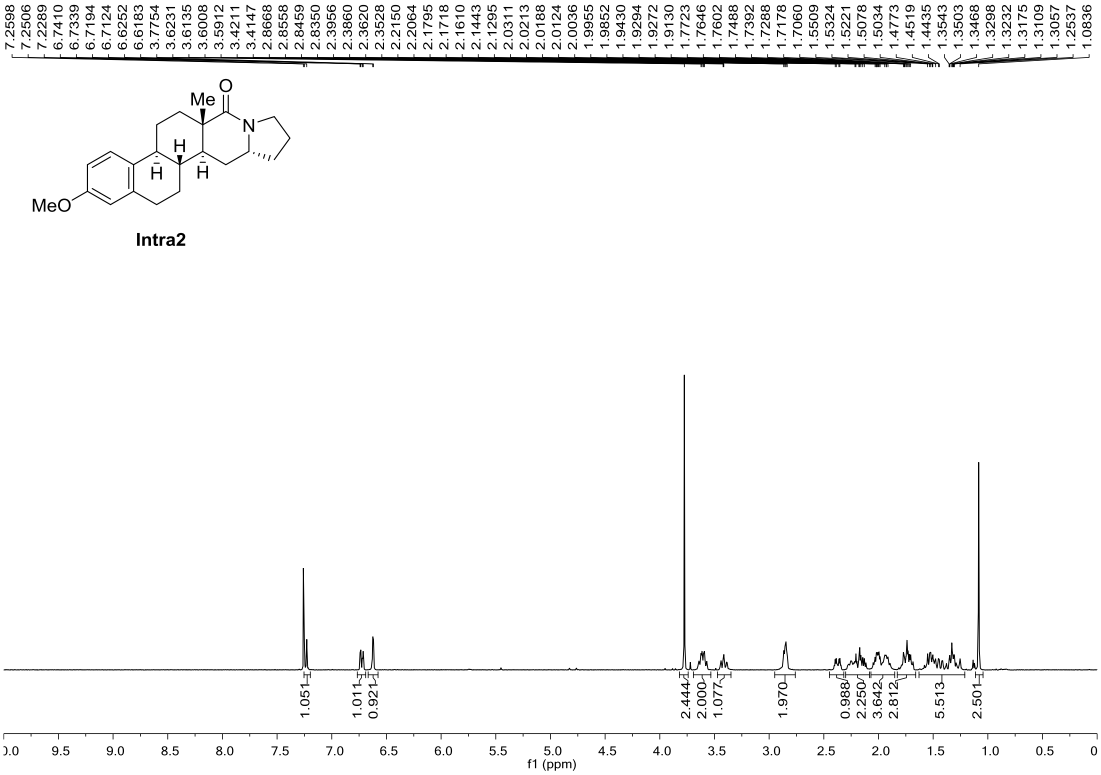

Supplementary Figure 297. ^{13}C NMR spectrum in $CDCl_3$, 101 MHz, of **Intra2**.

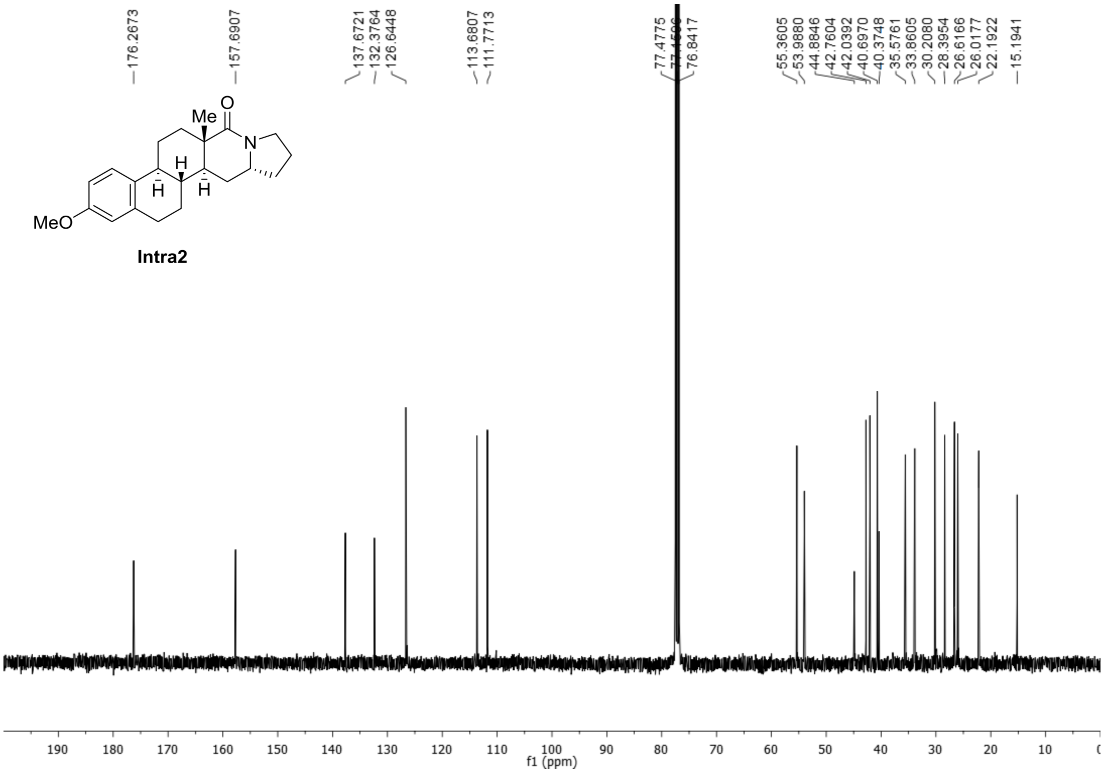

Supplementary Figure 298. ^{1}H NMR spectrum in DMSO-d_6, 600 MHz, of **Intra3**.

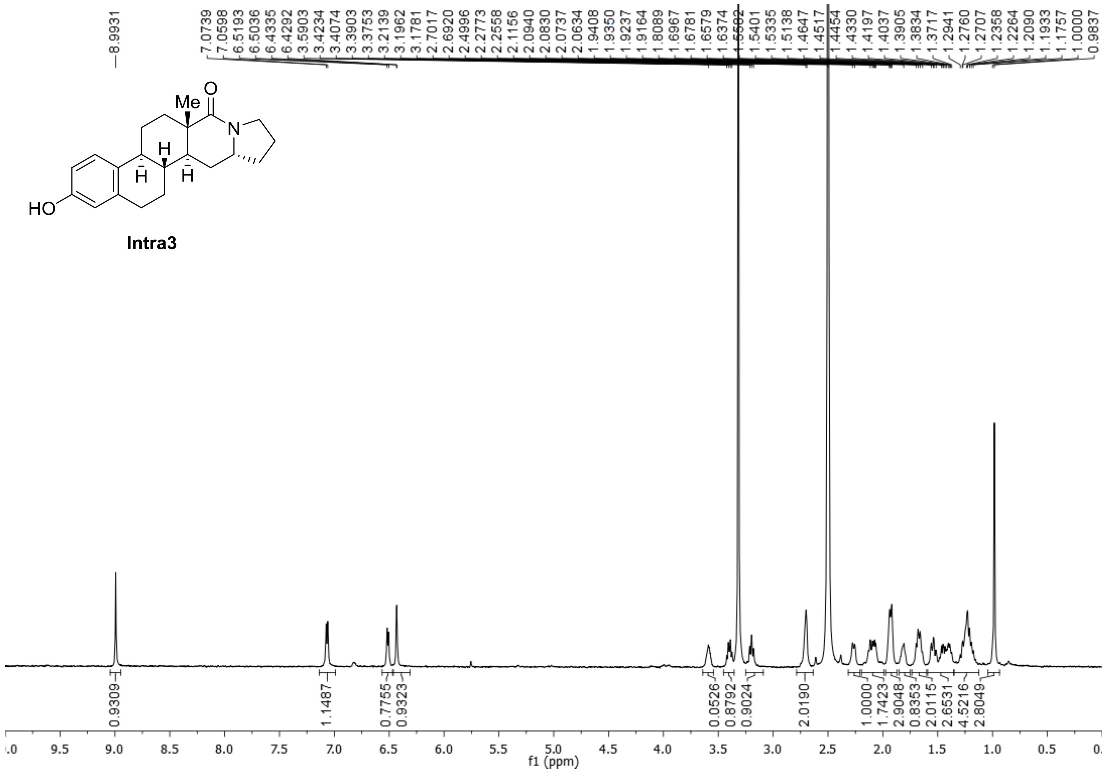

Supplementary Figure 299. ^{13}C NMR spectrum in DMSO-d_6, 151 MHz, of **Intra3**.

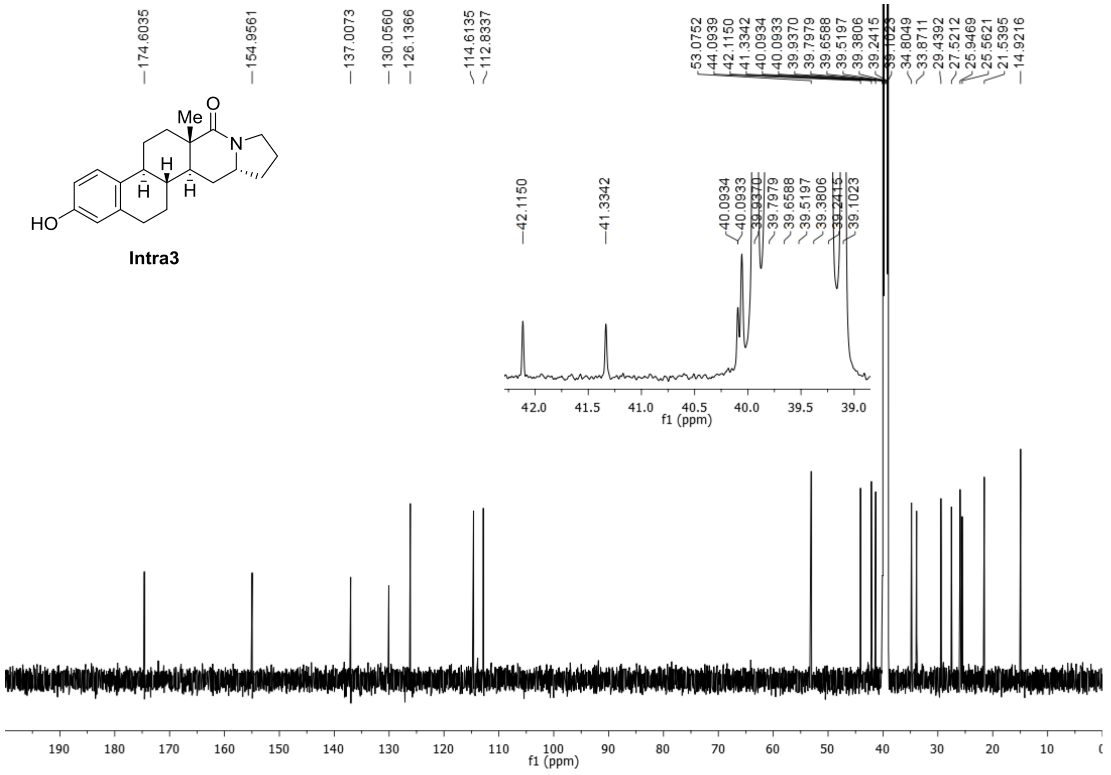

Supplementary Figure 300. ^{1}H NMR spectrum in $CDCl_3$, 400 MHz, of **G1**.

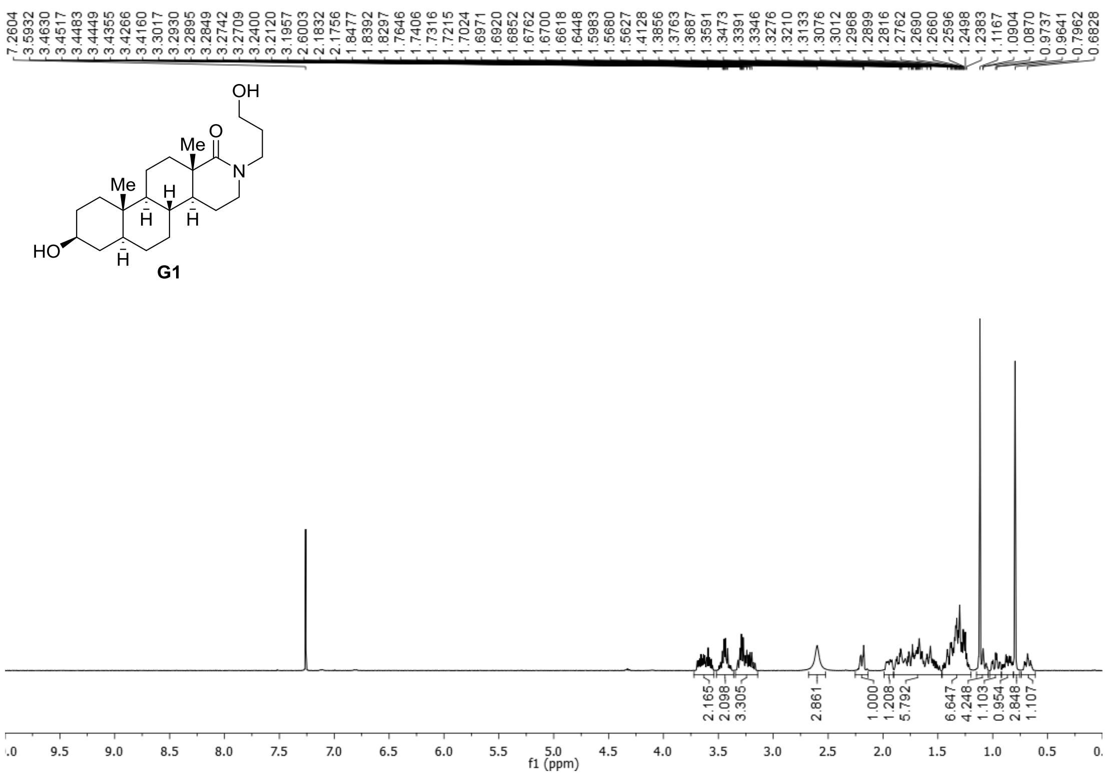

Supplementary Figure 301. ^{13}C NMR spectrum in $CDCl_3$, 101 MHz, of **G1**.

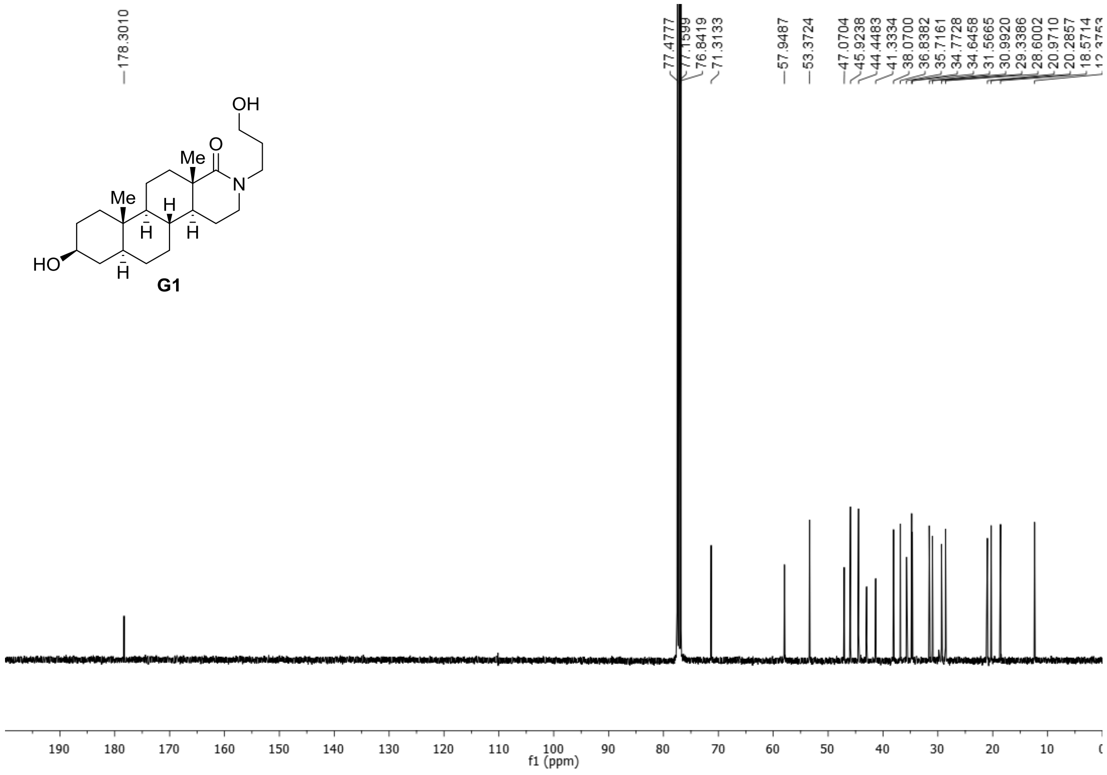

Supplementary Figure 302. ^{13}C NMR (APT) spectrum in $CDCl_3$, 101 MHz, of **G1**.

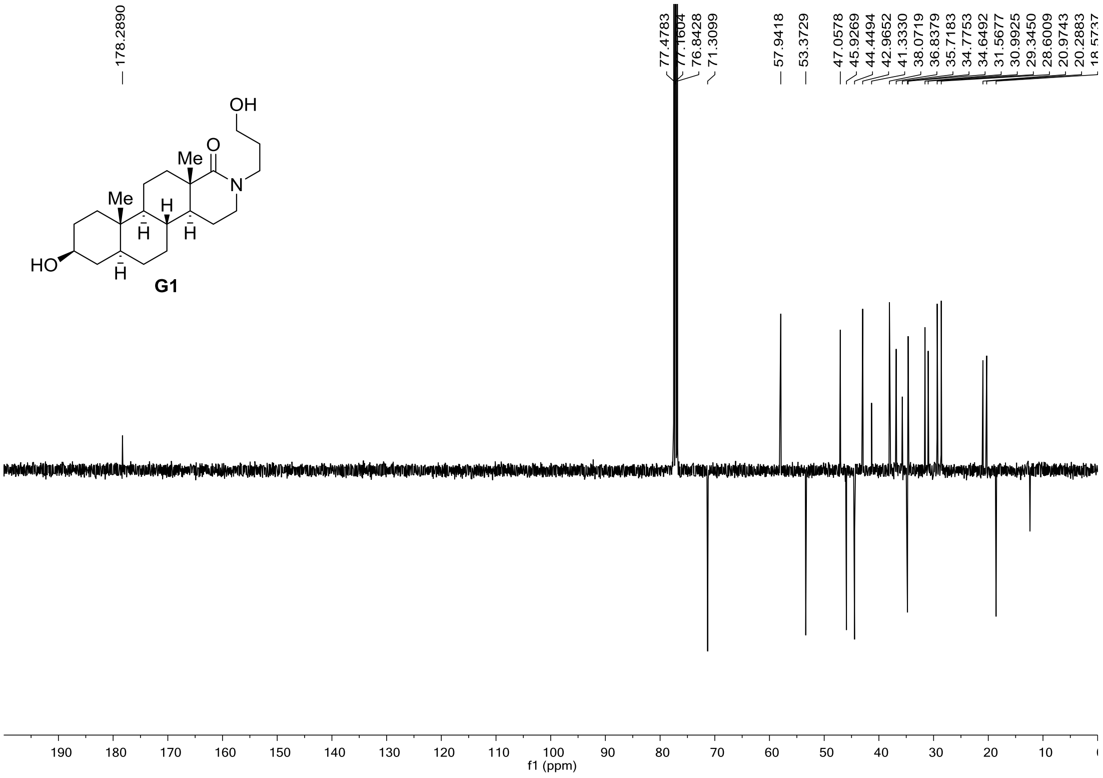

Supplementary Figure 303. HSQC spectrum of **G1**.

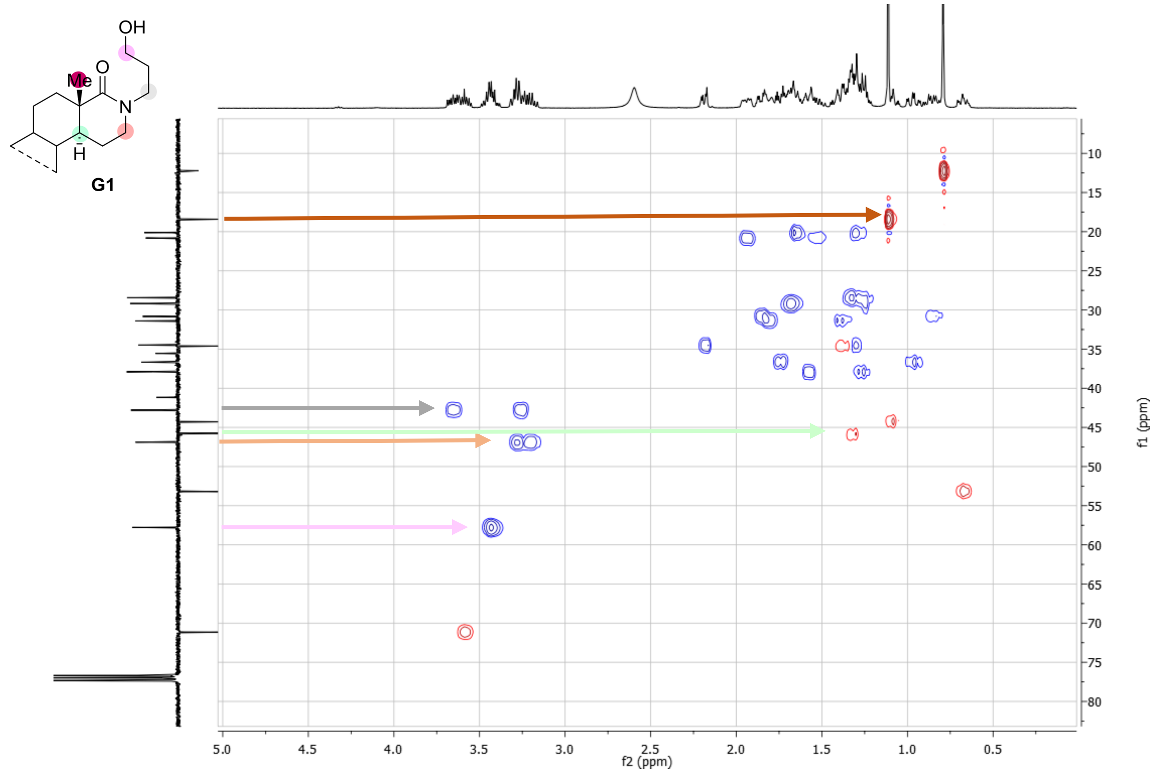

Supplementary Figure 304. HMBC spectrum of **G1**.

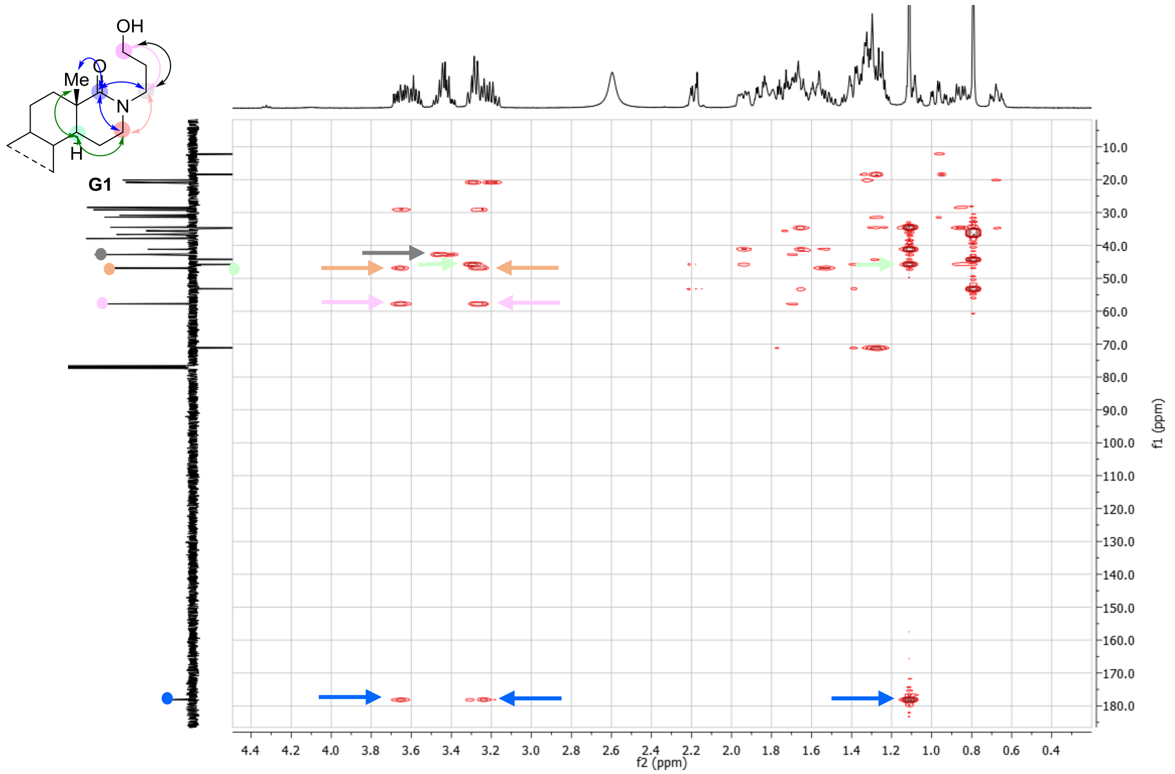

Supplementary Figure 305. ^{1}H NMR spectrum in $CDCl_3$, 400 MHz, of **G2**.

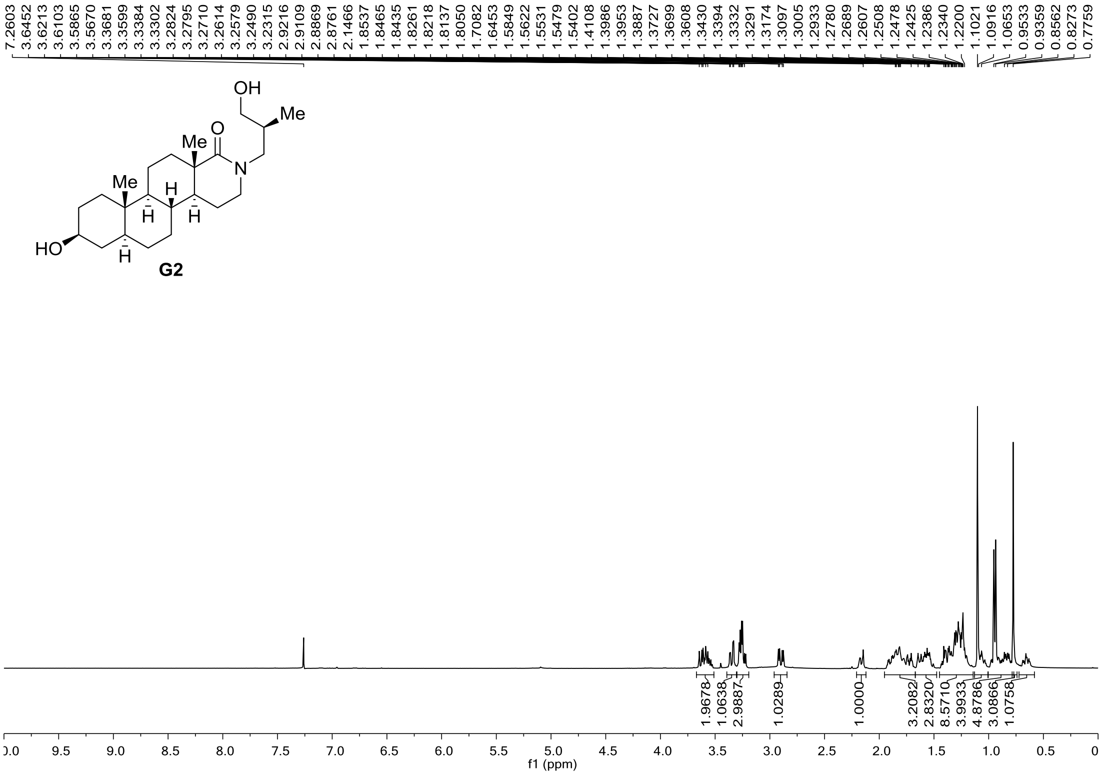

Supplementary Figure 306. ^{13}C NMR spectrum in $CDCl_3$, 101 MHz, of **G2**.

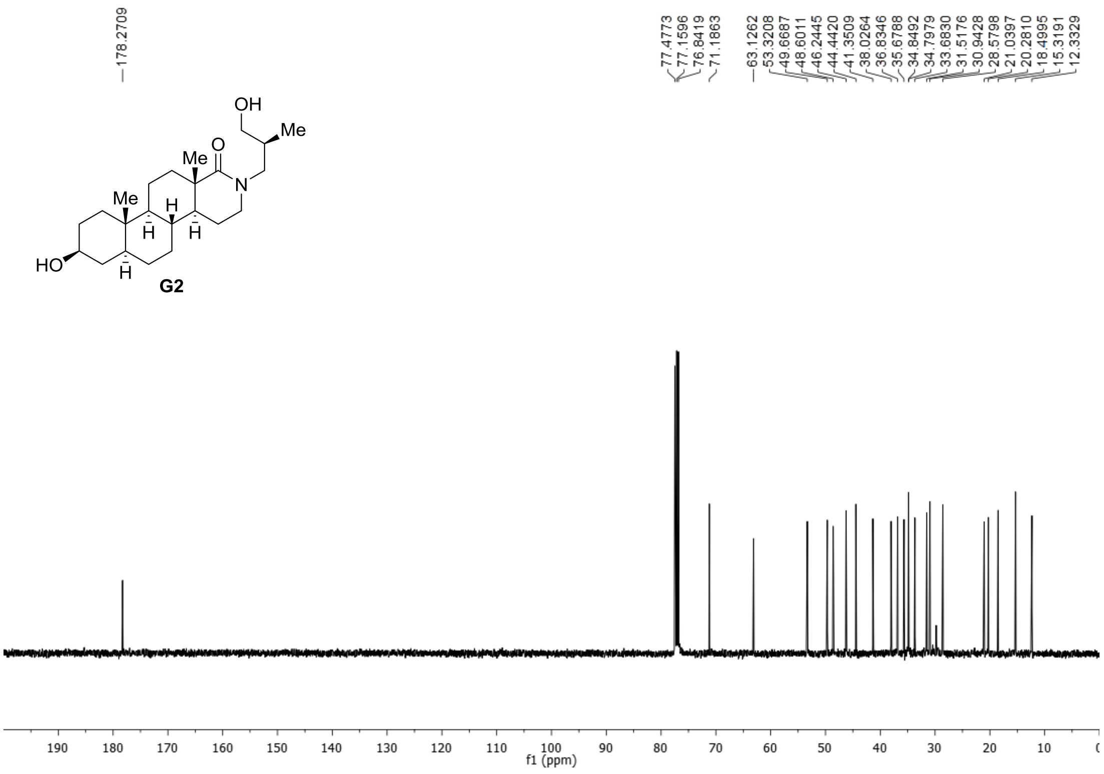

Supplementary Figure 307. ^{1}H NMR spectrum in $CDCl_3$, 400 MHz, of **H1**.

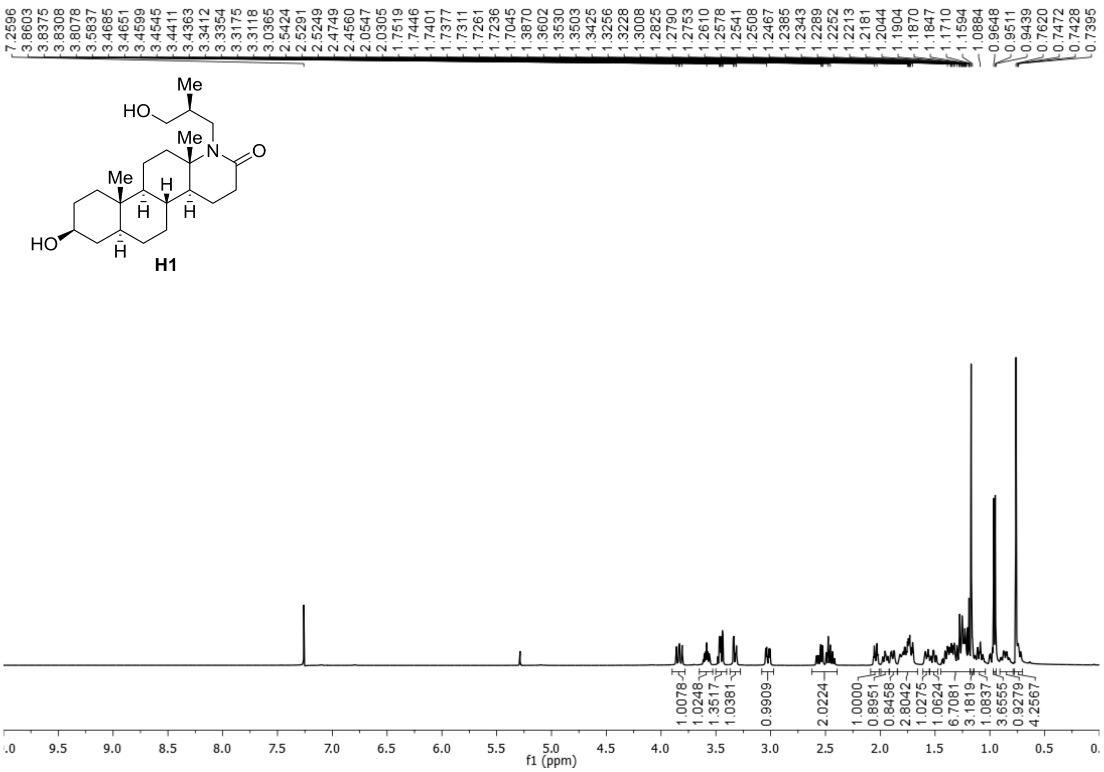

Supplementary Figure 308. ^{13}C NMR spectrum in $CDCl_3$, 126 MHz, of **H1**.

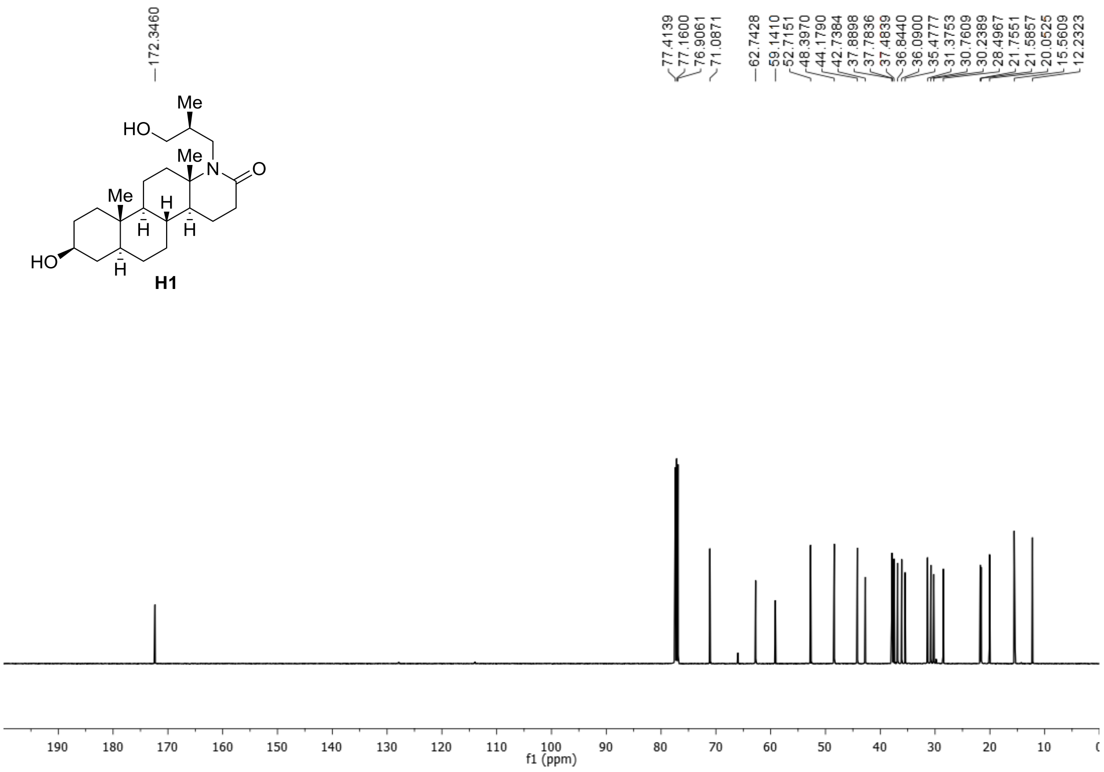

Supplementary Figure 309. ^{1}H NMR spectrum in $CDCl_3$, 400 MHz, of **G3**.

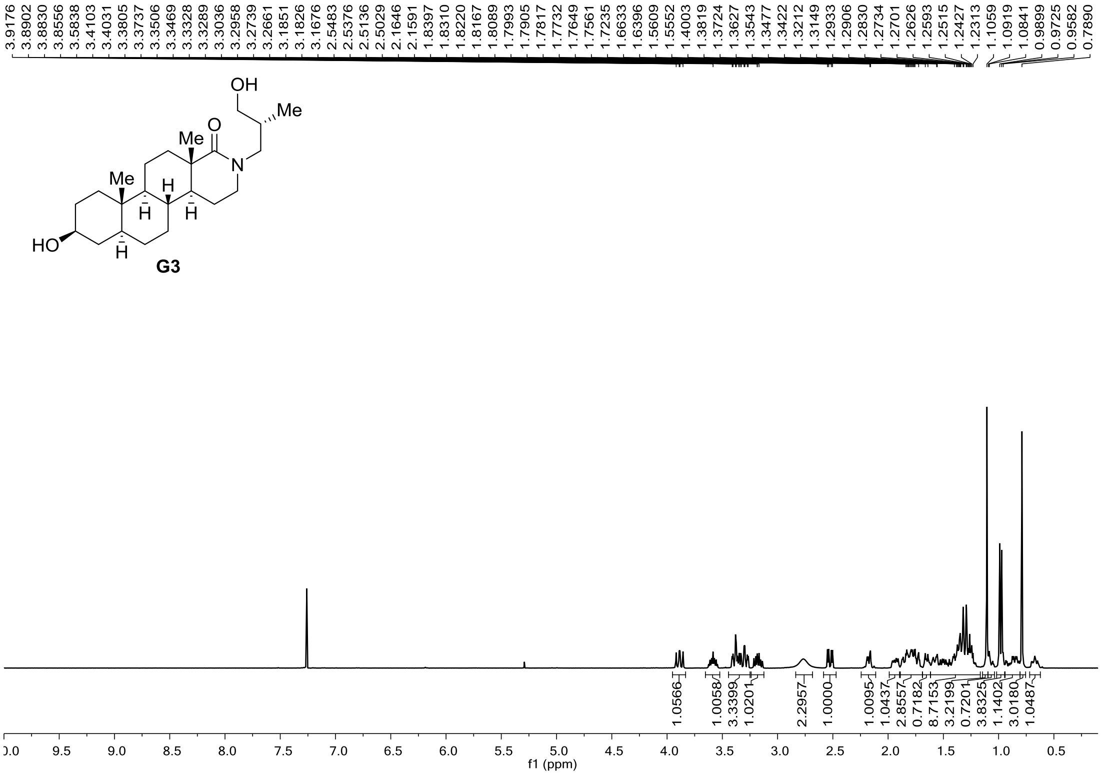

Supplementary Figure 310. ^{13}C NMR spectrum in $CDCl_3$, 101 MHz, of **G3**.

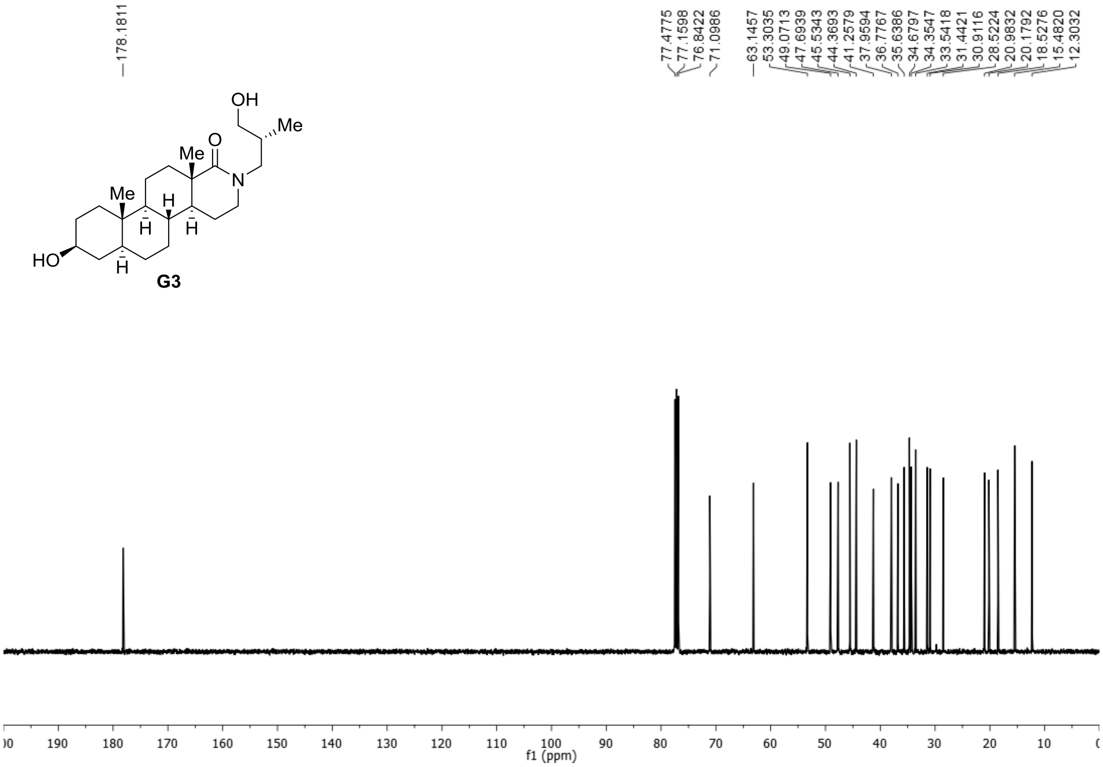

Supplementary Figure 311. ^{1}H NMR spectrum in $CDCl_3$, 400 MHz, of **G4**.

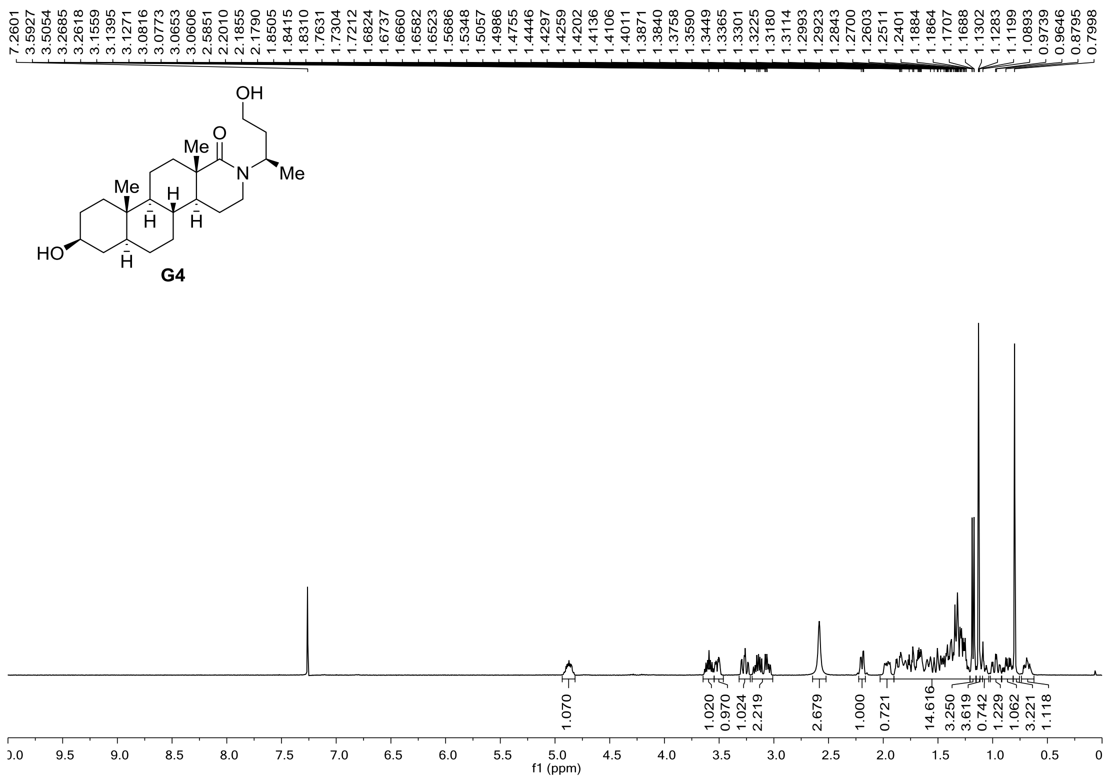

Supplementary Figure 312. ^{13}C NMR spectrum in $CDCl_3$, 101 MHz, of **G4**.

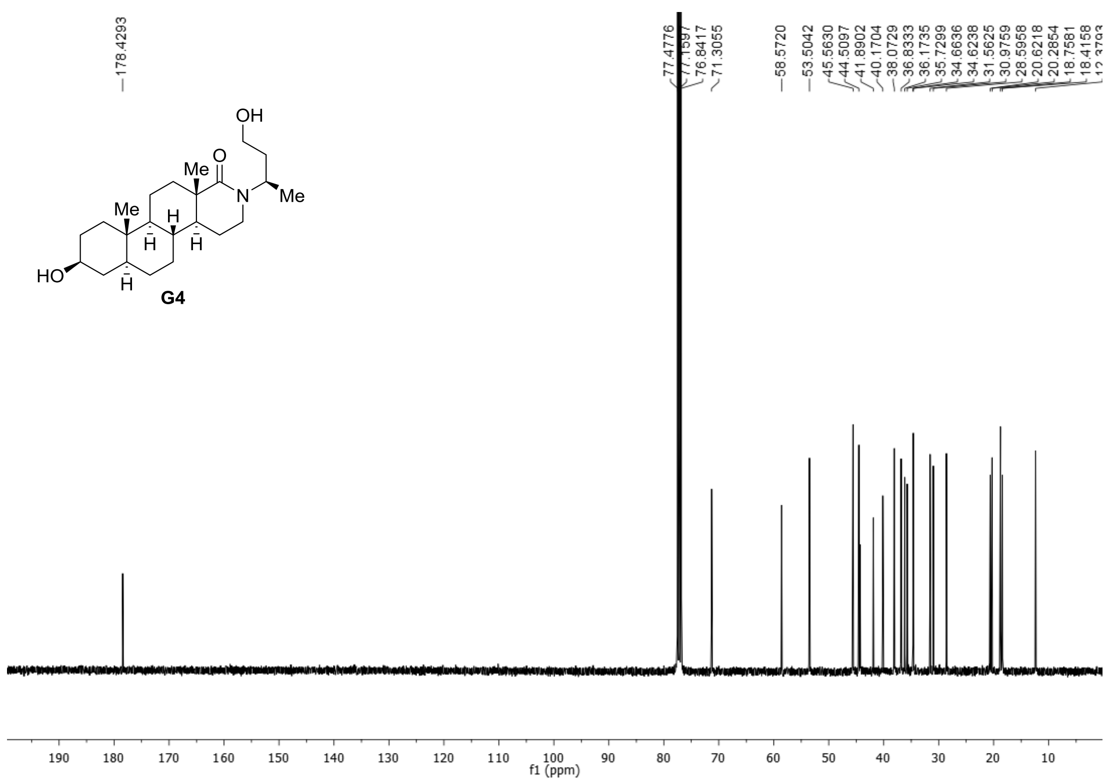

Supplementary Figure 313. ^{13}C NMR (APT) spectrum in $CDCl_3$, 101 MHz, of **G4**.

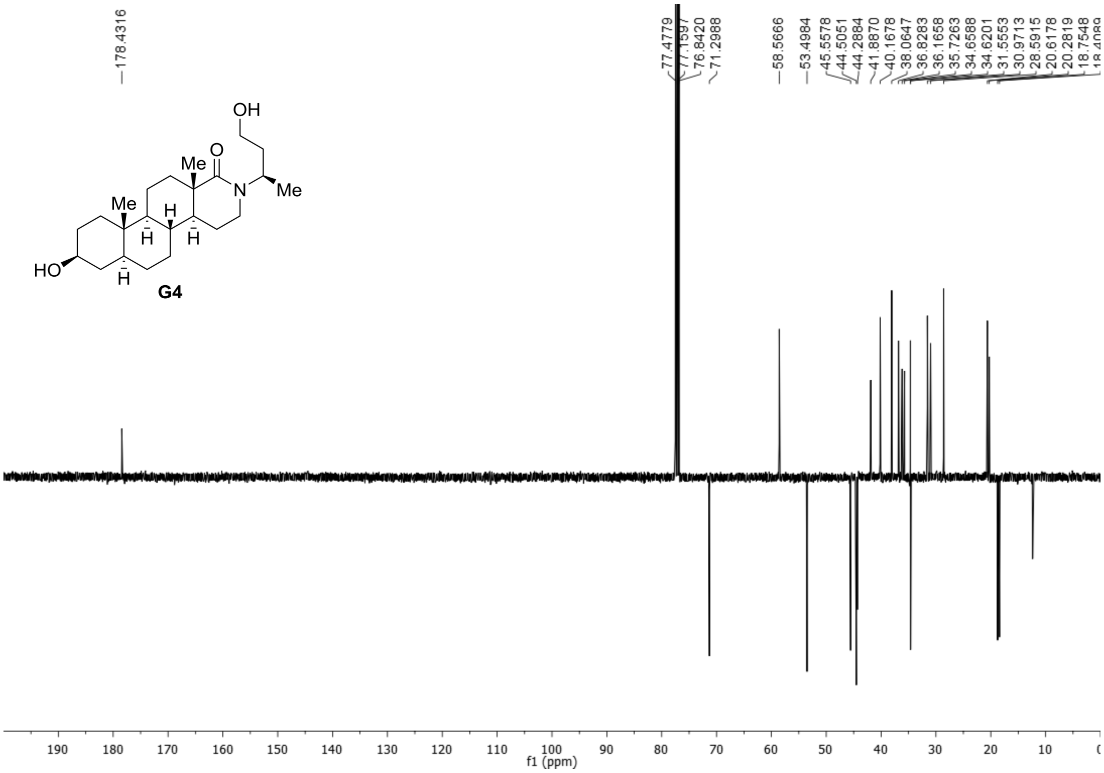

Supplementary Figure 314. 1D NOE spectrum in $CDCl_3$, 400 MHz, of **G4**.

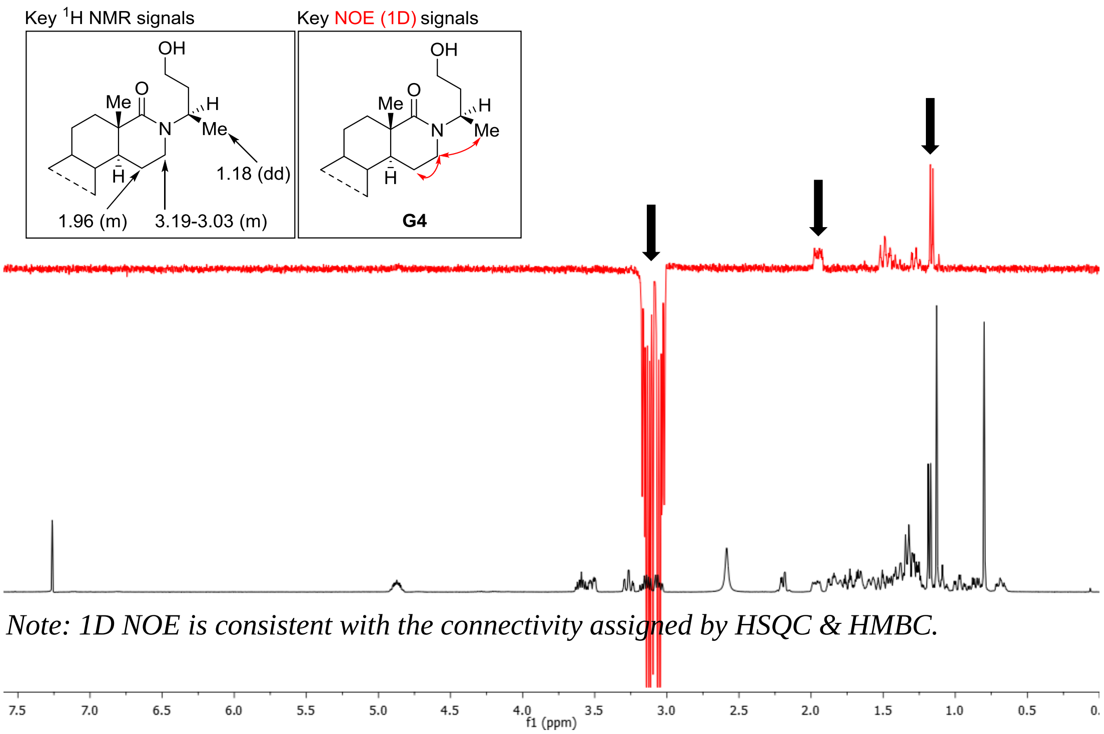

Supplementary Figure 315. HSQC of **G4**.

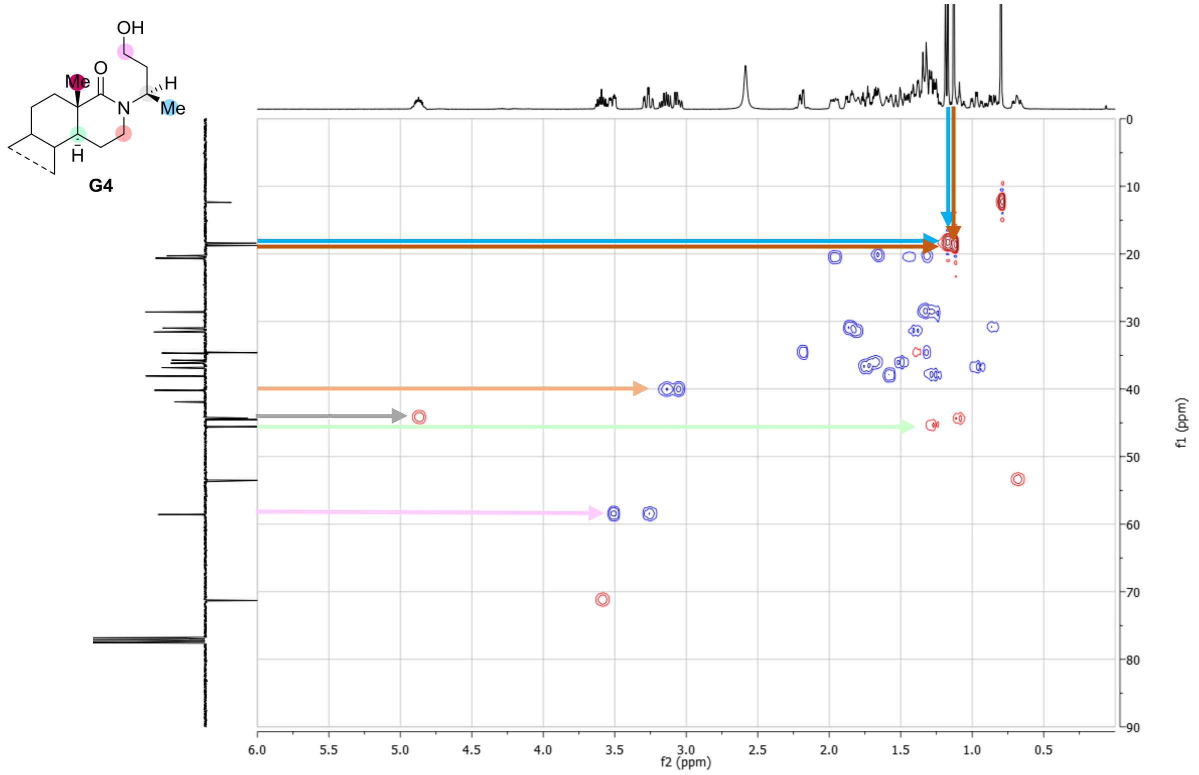

Supplementary Figure 316. HSQC of **G4**.

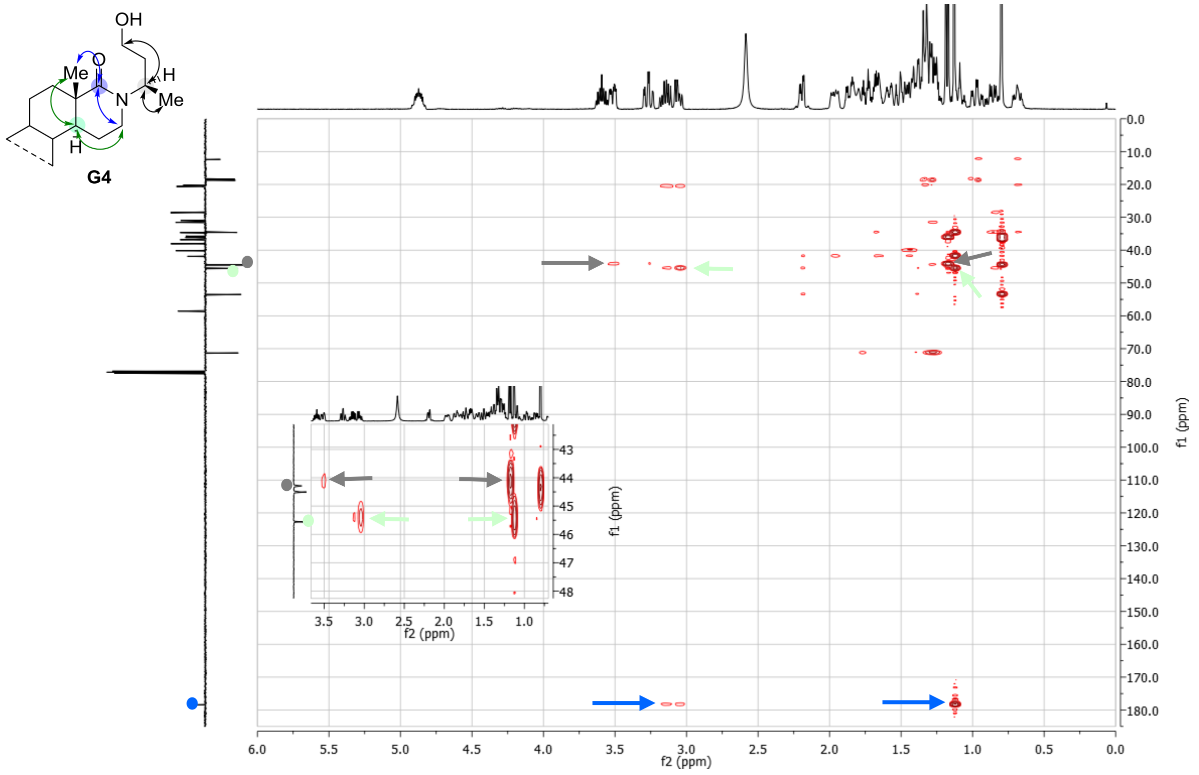

Supplementary Figure 317. ^{1}H NMR spectrum in $CDCl_3$, 400 MHz, of **G6**.

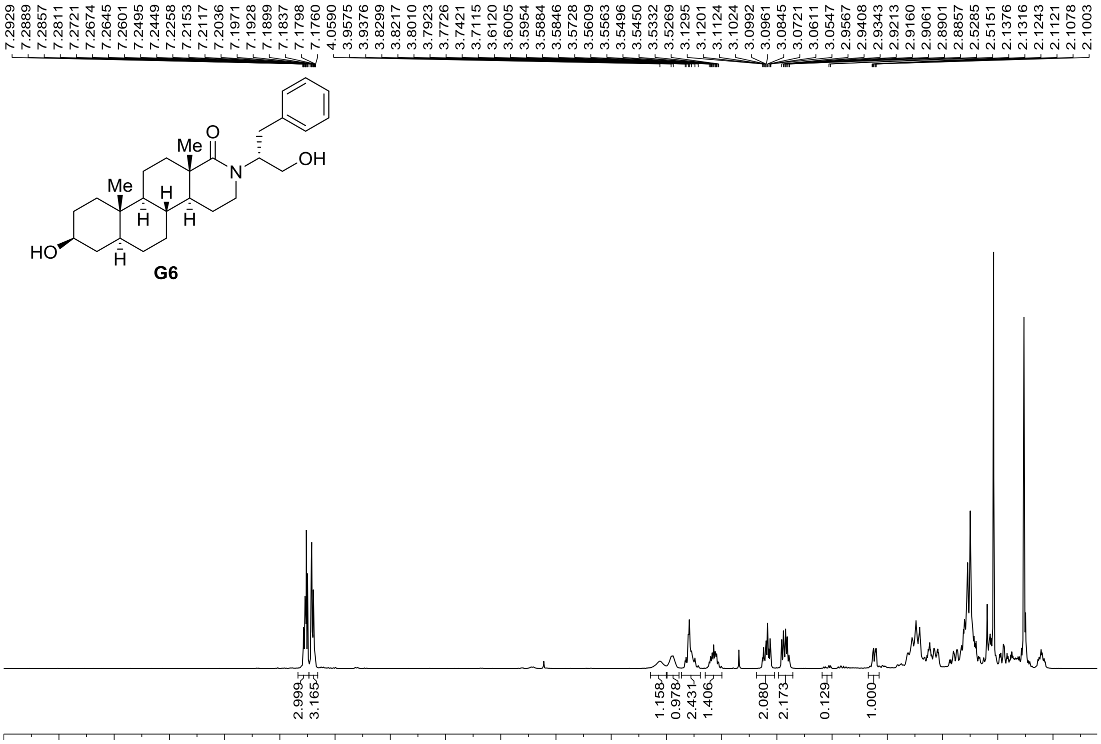

Supplementary Figure 318. ^{13}C NMR spectrum in $CDCl_3$, 101 MHz, of **G6**.

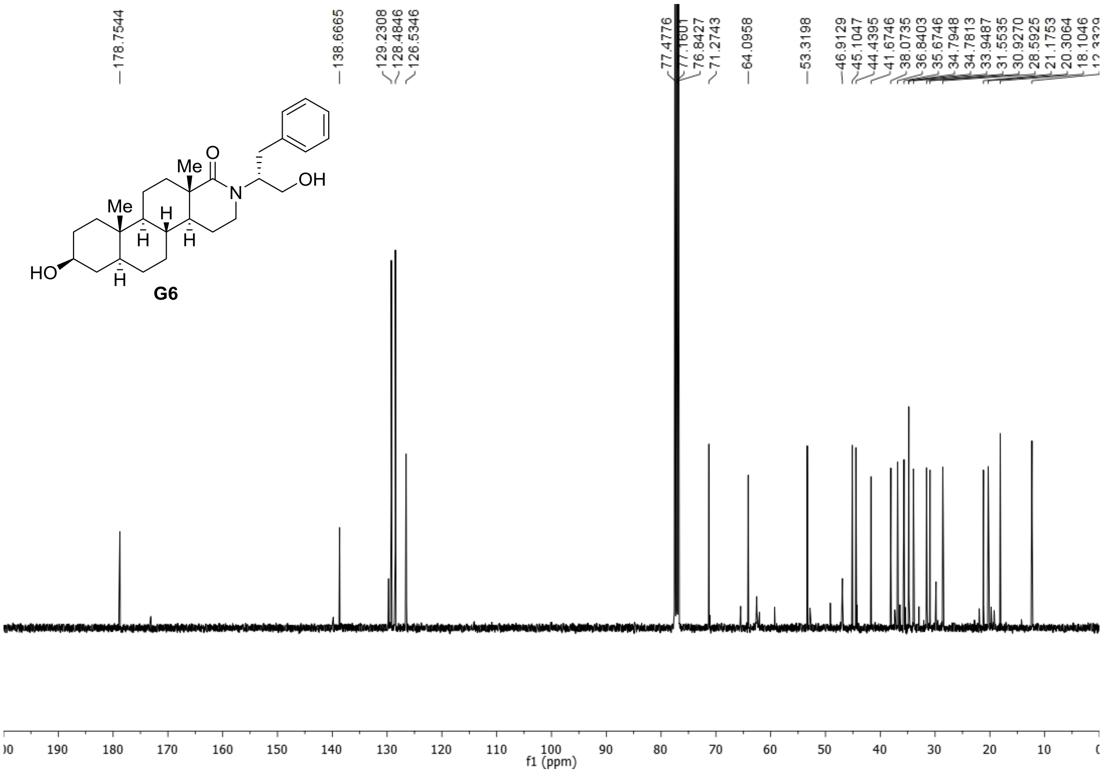

Supplementary Figure 319. ^{1}H NMR spectrum in $CDCl_3$, 400 MHz, of **G7**.

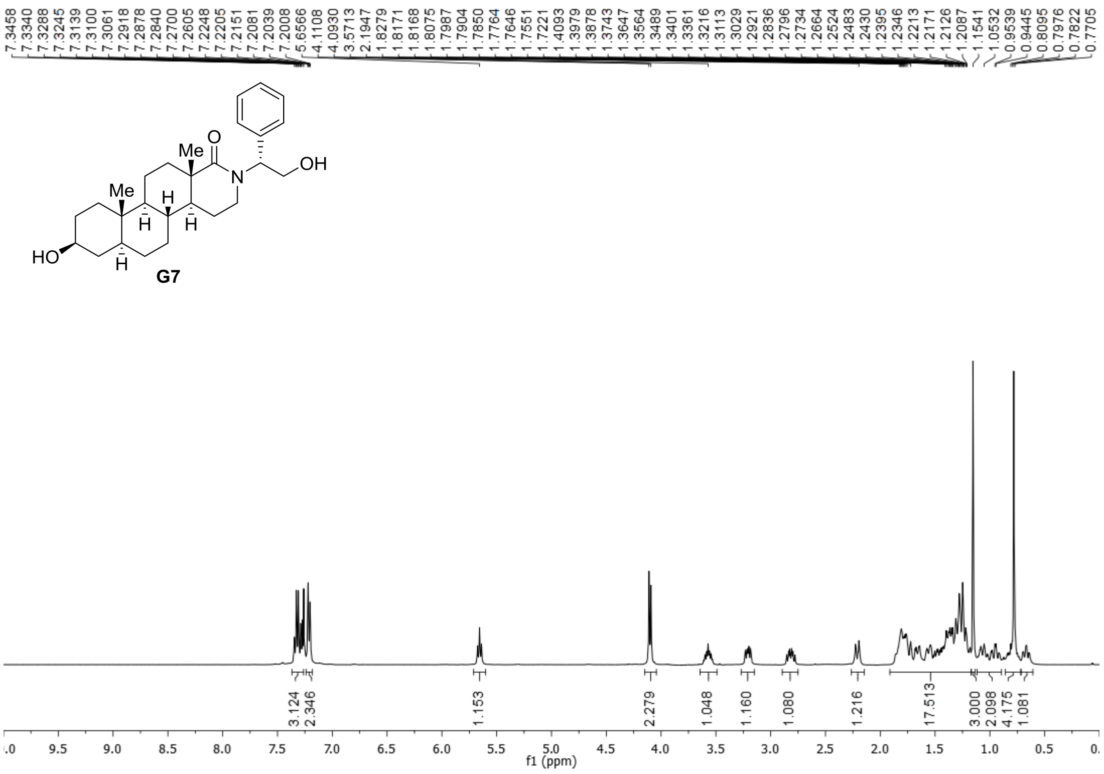

Supplementary Figure 320. ^{13}C NMR spectrum in $CDCl_3$, 126 MHz, of **G7**.

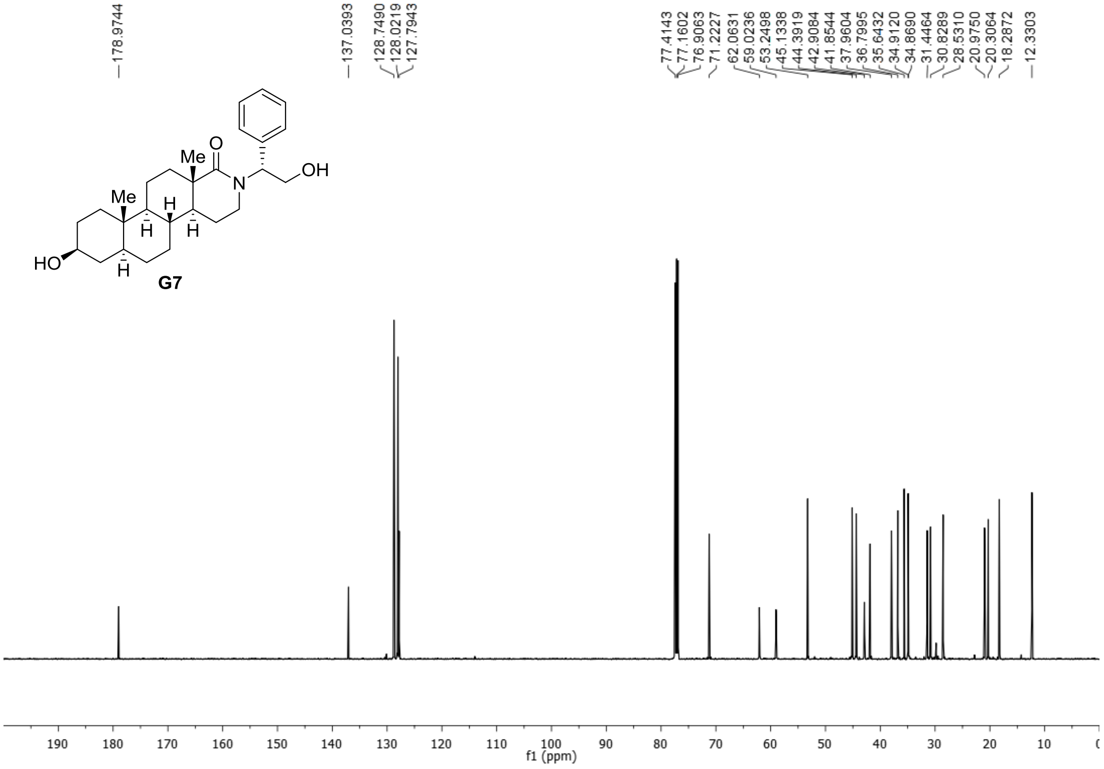

Supplementary Figure 321. ^{1}H NMR spectrum in $CDCl_3$, 400 MHz, of **G8**.

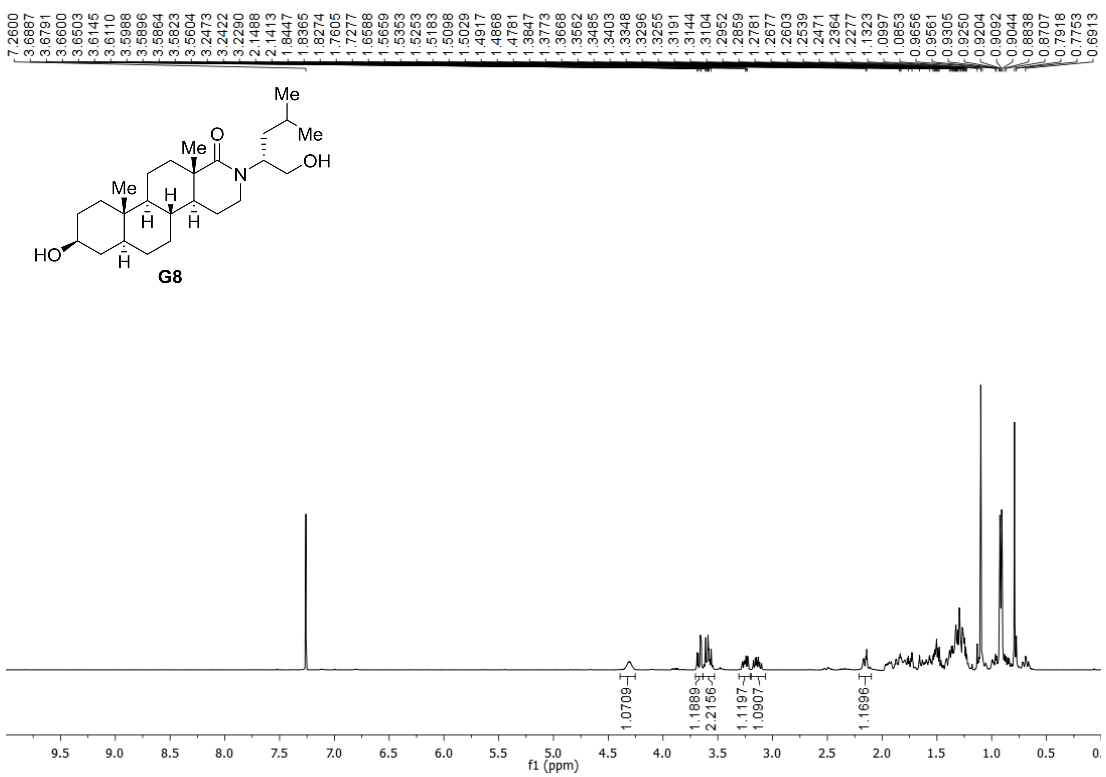

Supplementary Figure 322. ^{13}C NMR (APT) spectrum in $CDCl_3$, 101 MHz, of **G8**.

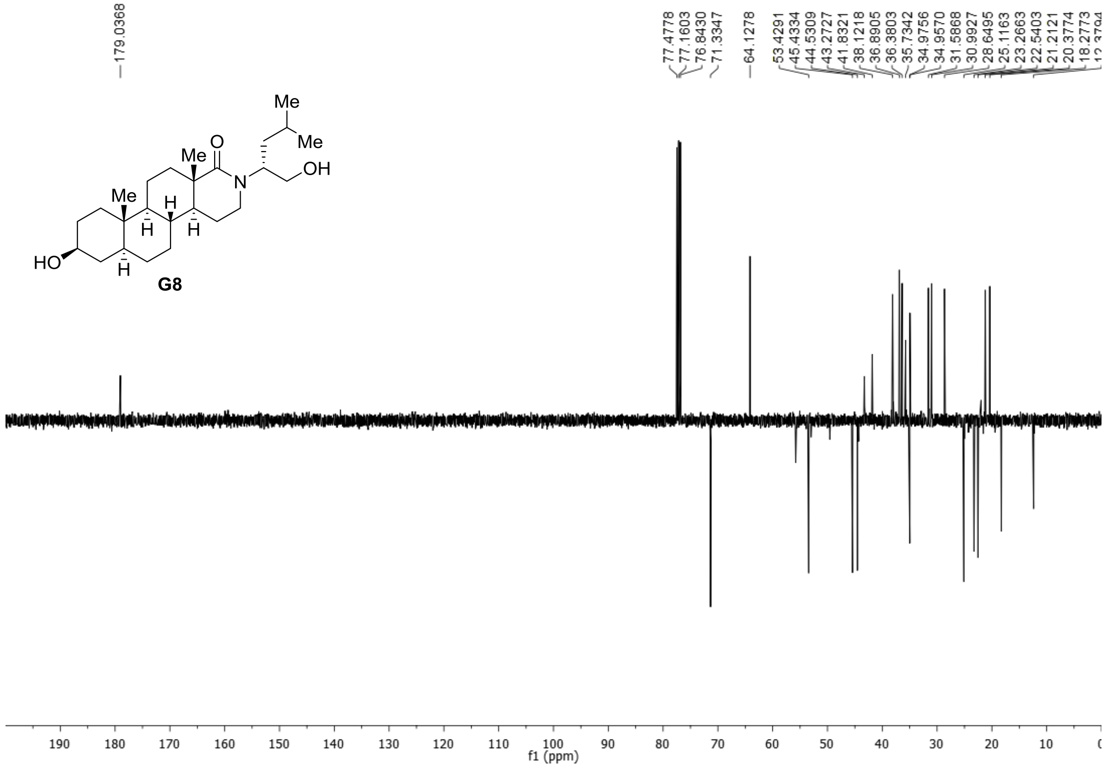

Supplementary Figure 323. ^{1}H NMR spectrum in $CDCl_3$, 400 MHz, of **G9**.

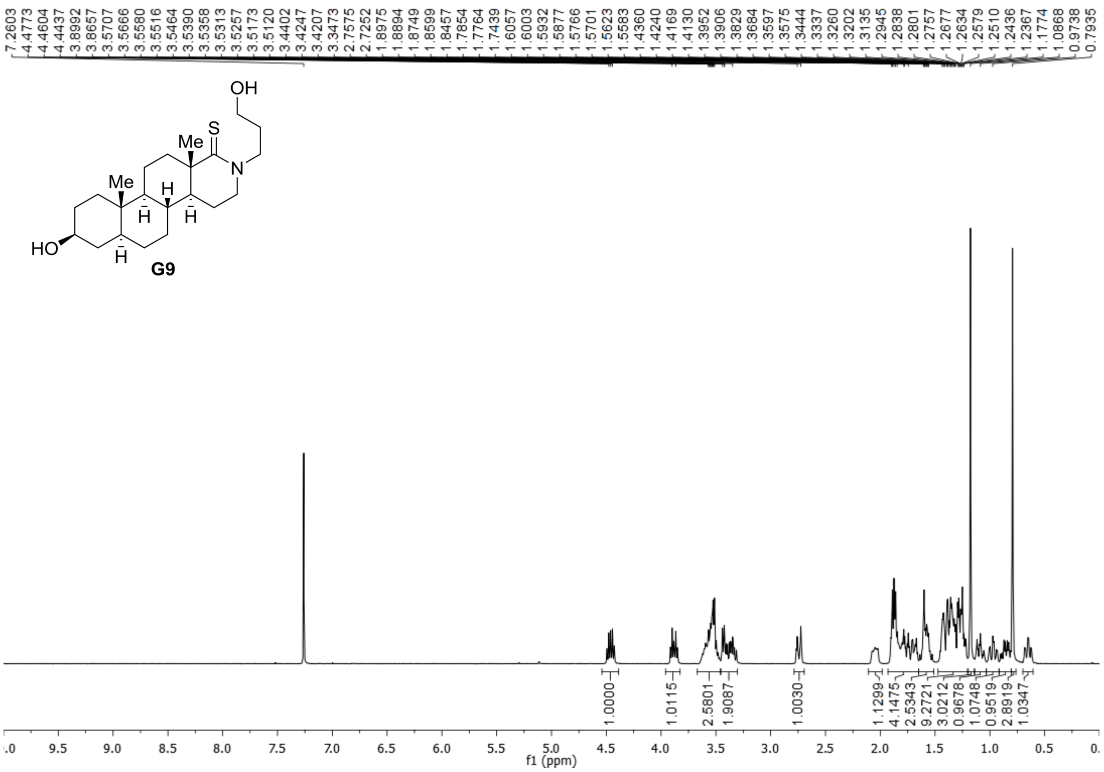

Supplementary Figure 324. ^{13}C NMR spectrum in $CDCl_3$, 101 MHz, **G9**.

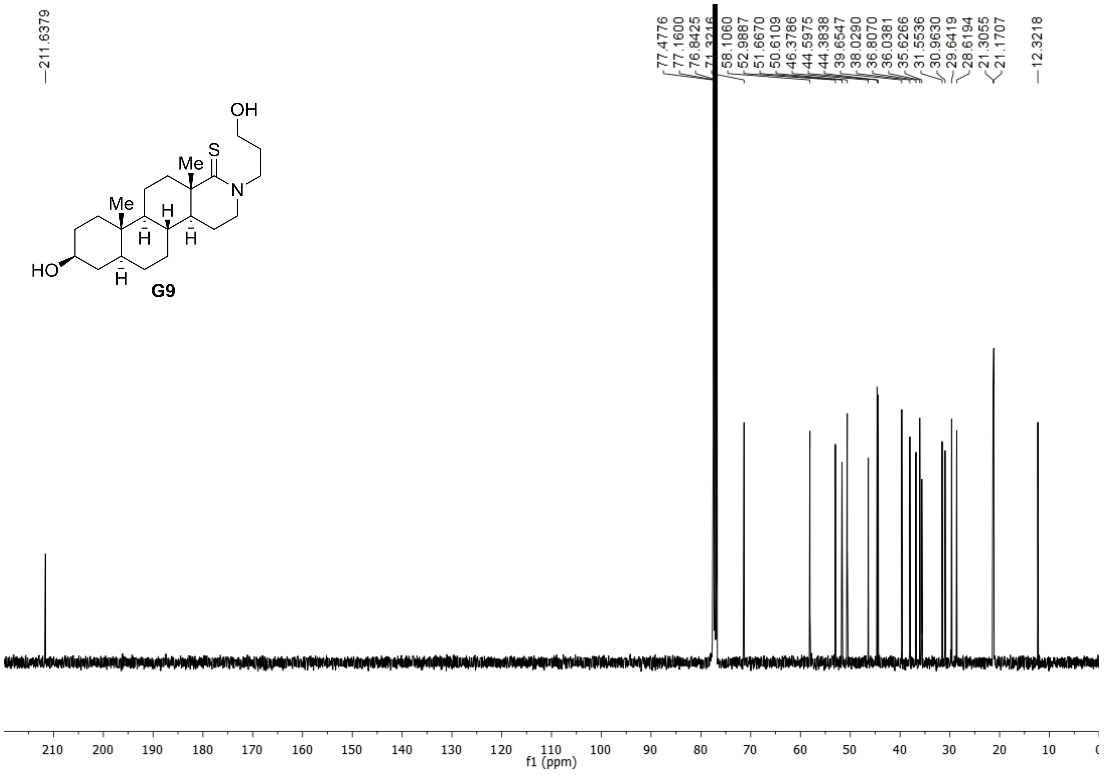

Supplementary Figure 325. ^{1}H NMR spectrum in $CDCl_3$, 400 MHz, of **G10**.

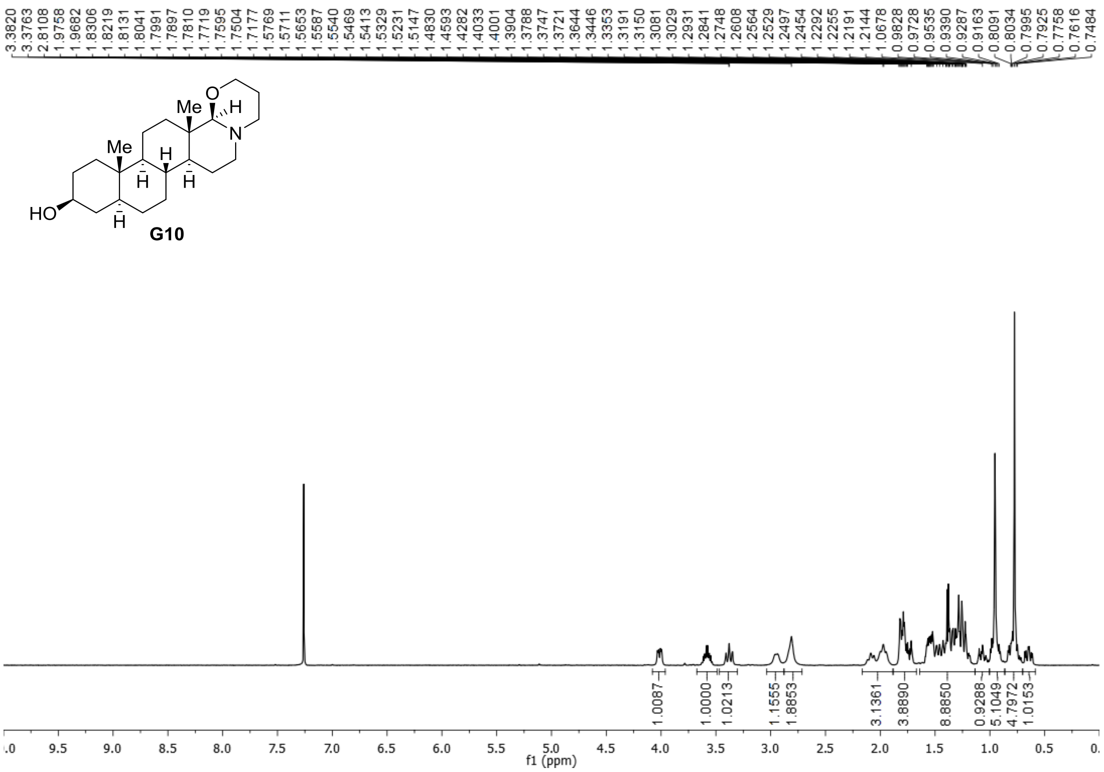

Supplementary Figure 326. ^{13}C NMR (APT) spectrum in $CDCl_3$, 101 MHz, of **G10**.

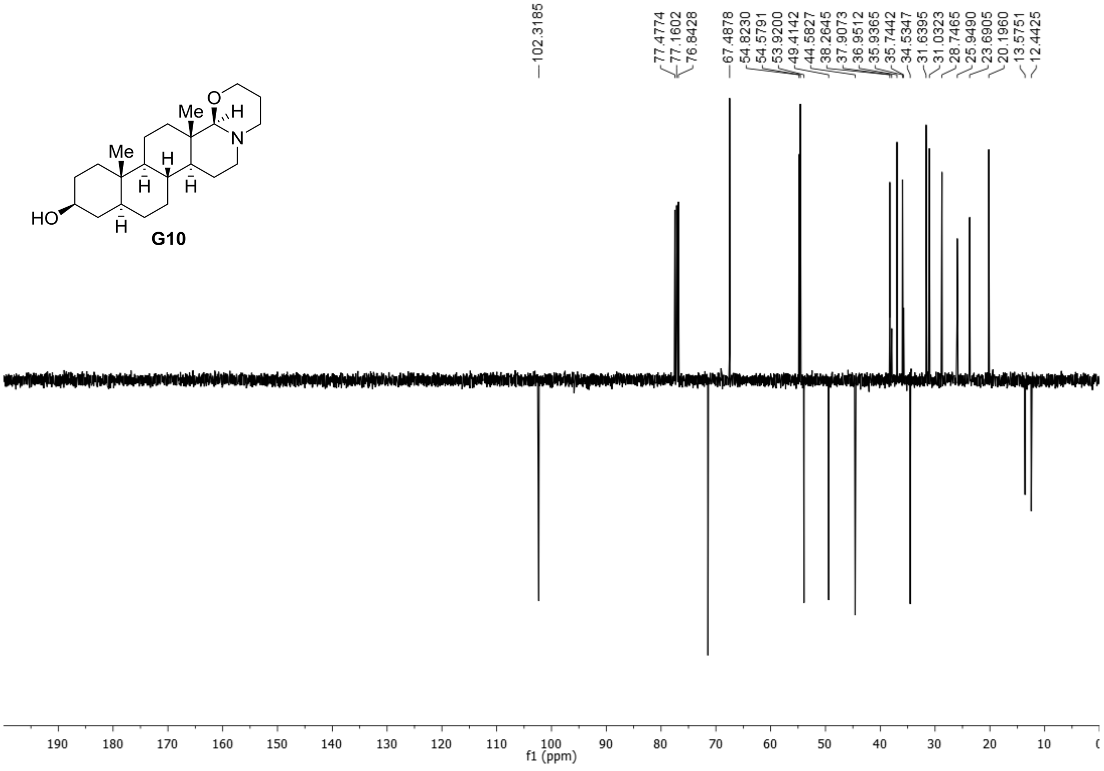

Supplementary Figure 327. ^{1}H NMR spectrum in $CDCl_3$, 400 MHz, of **G11**.

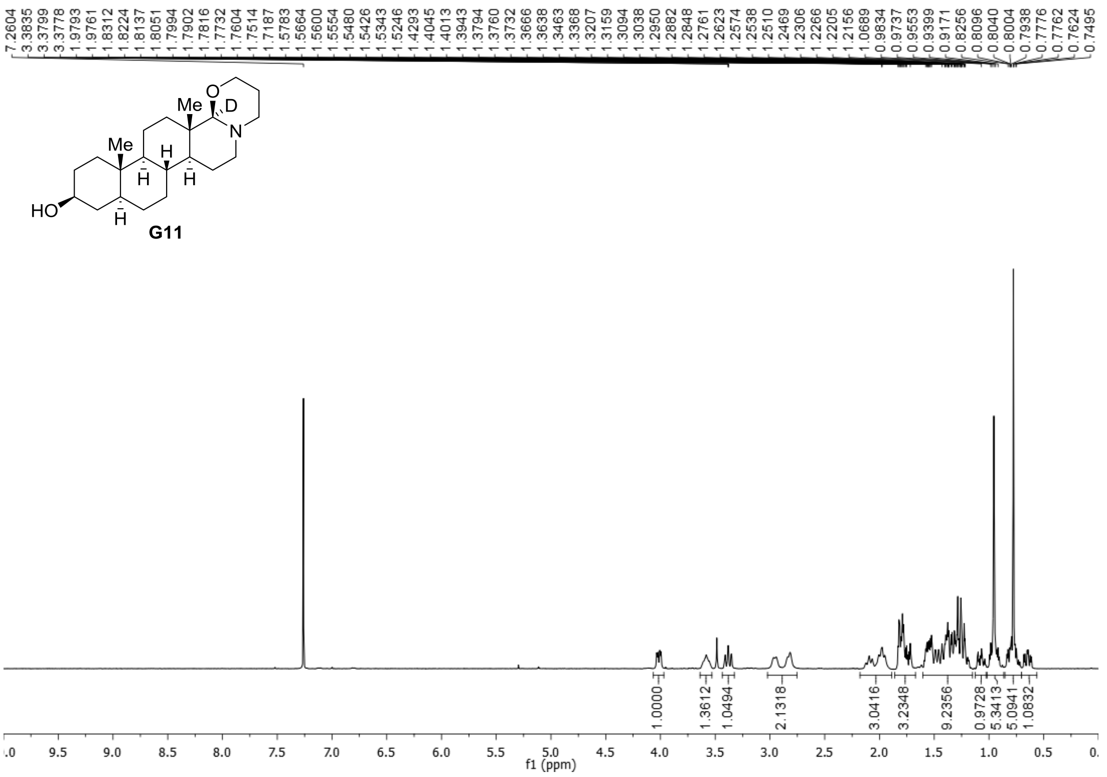

Supplementary Figure 328. ^{13}C NMR spectrum in $CDCl_3$, 101 MHz, **G11**.

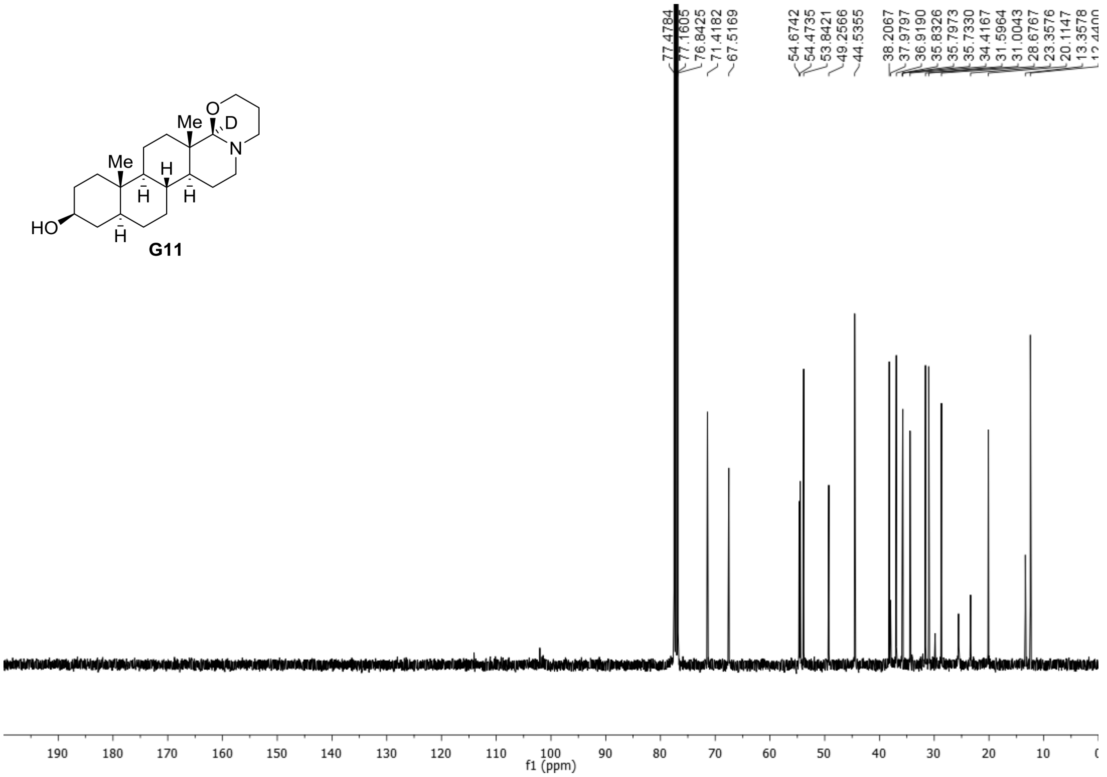

Supplementary Figure 329. ^{1}H NMR spectrum in $CDCl_3$, 400 MHz, of **G12**.

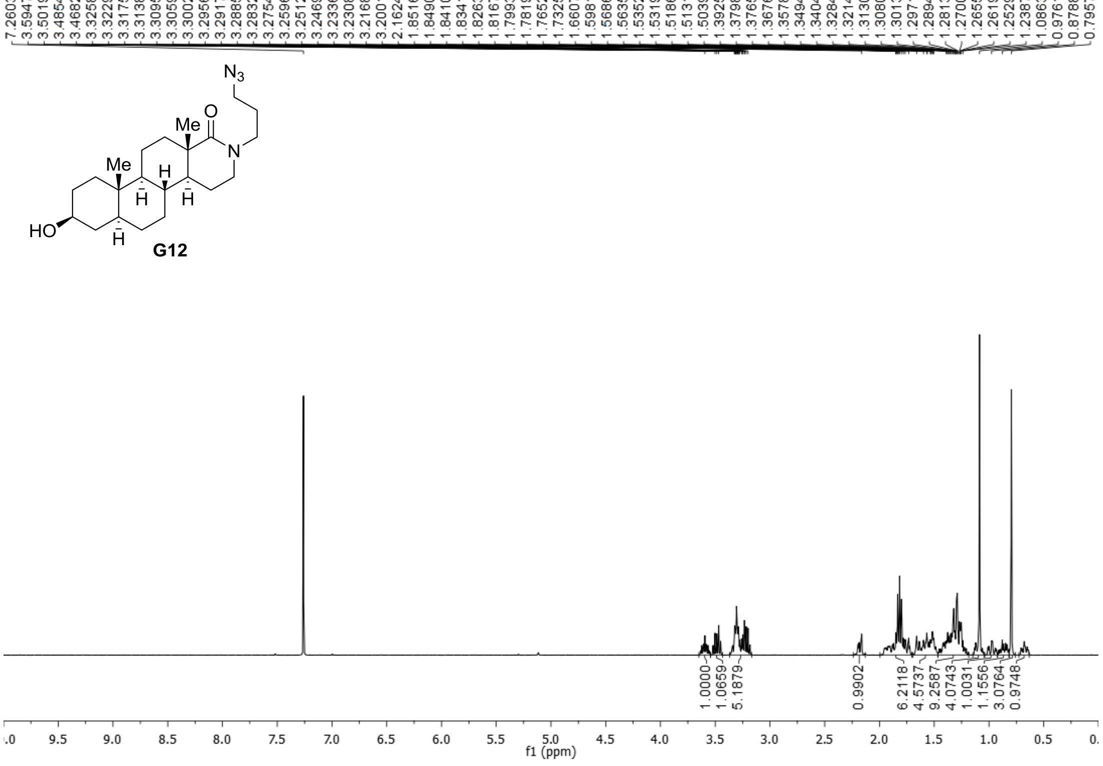

Supplementary Figure 330. ^{13}C NMR spectrum in $CDCl_3$, 101 MHz, of **G12**.

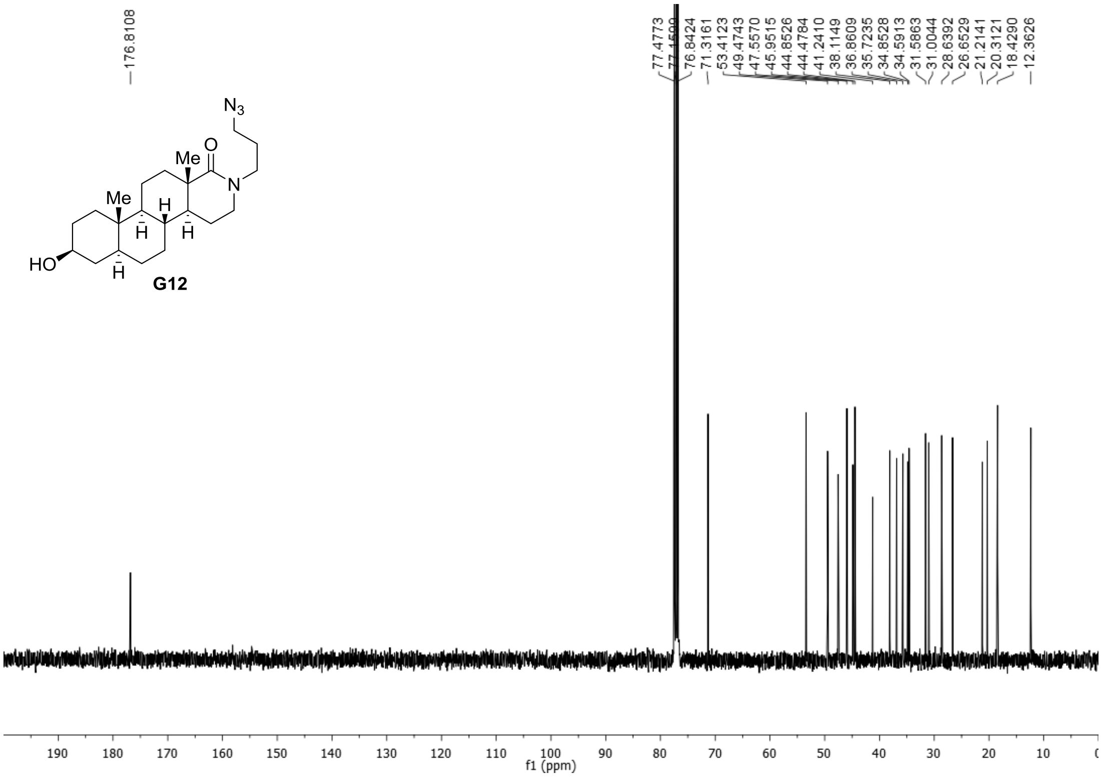

Supplementary Figure 331. ^{1}H NMR spectrum in $CDCl_3$, 400 MHz, of **G13**.

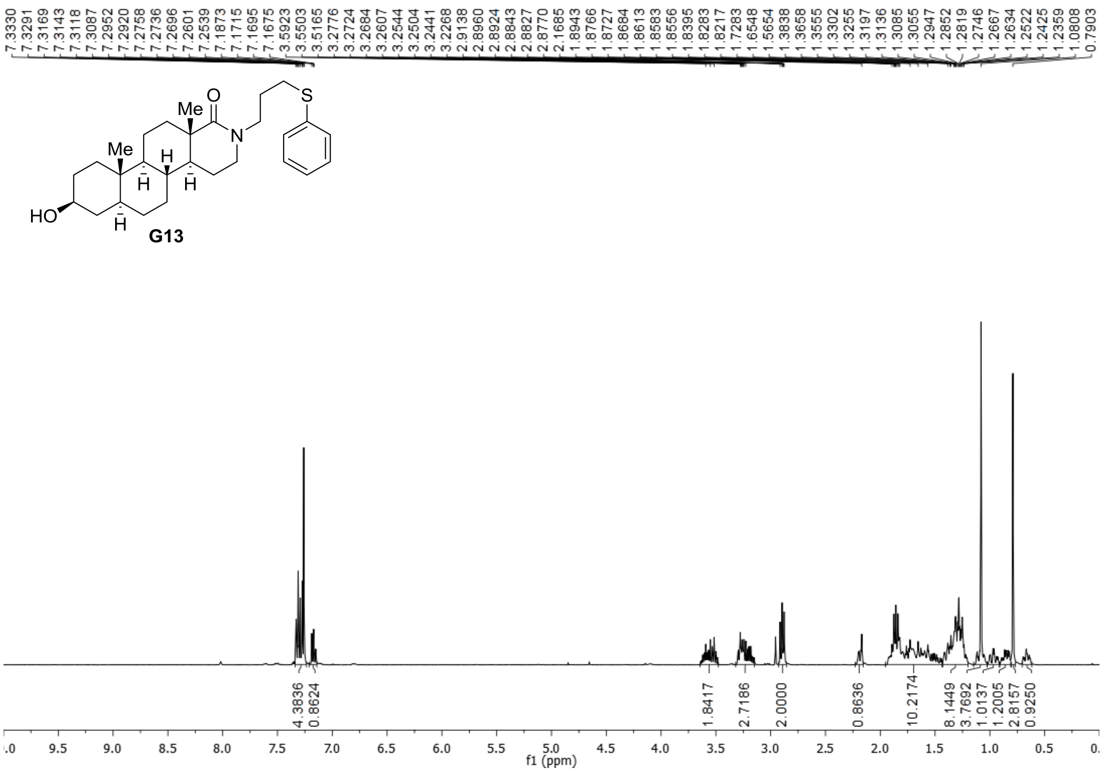

Supplementary Figure 332. ^{13}C NMR spectrum in $CDCl_3$, 101 MHz, of **G13**.

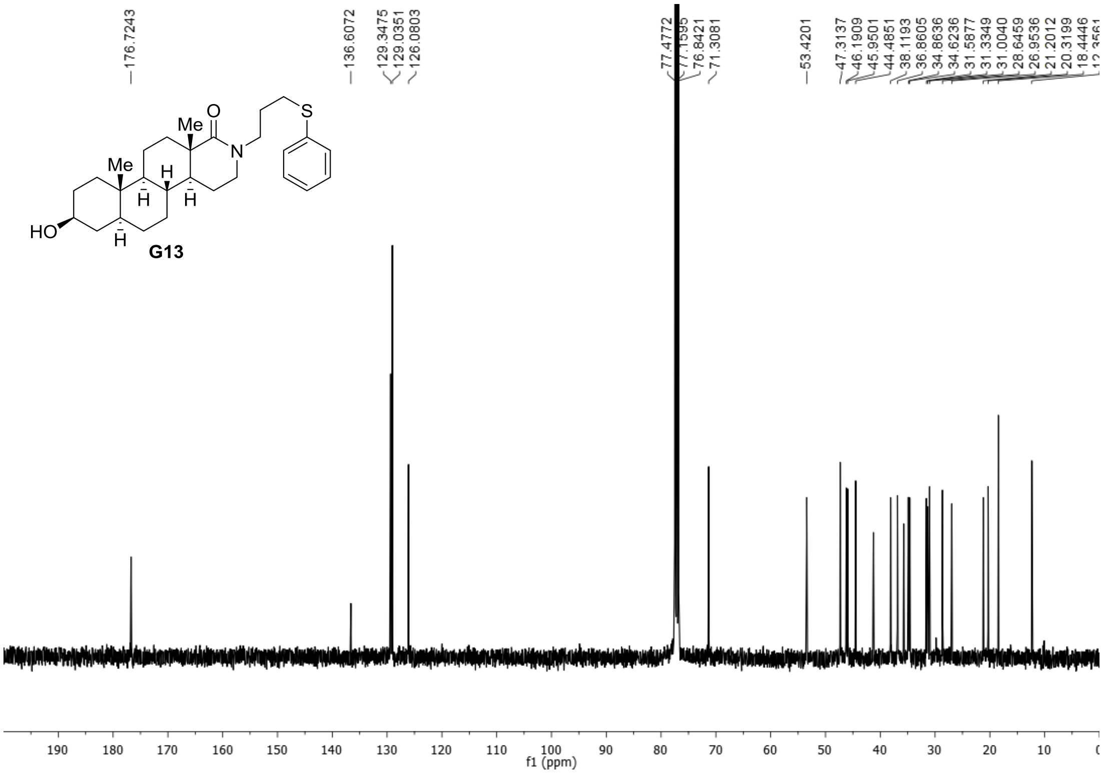

Supplementary Figure 333. ^{1}H NMR spectrum in $CDCl_3$, 400 MHz, of **G14**.

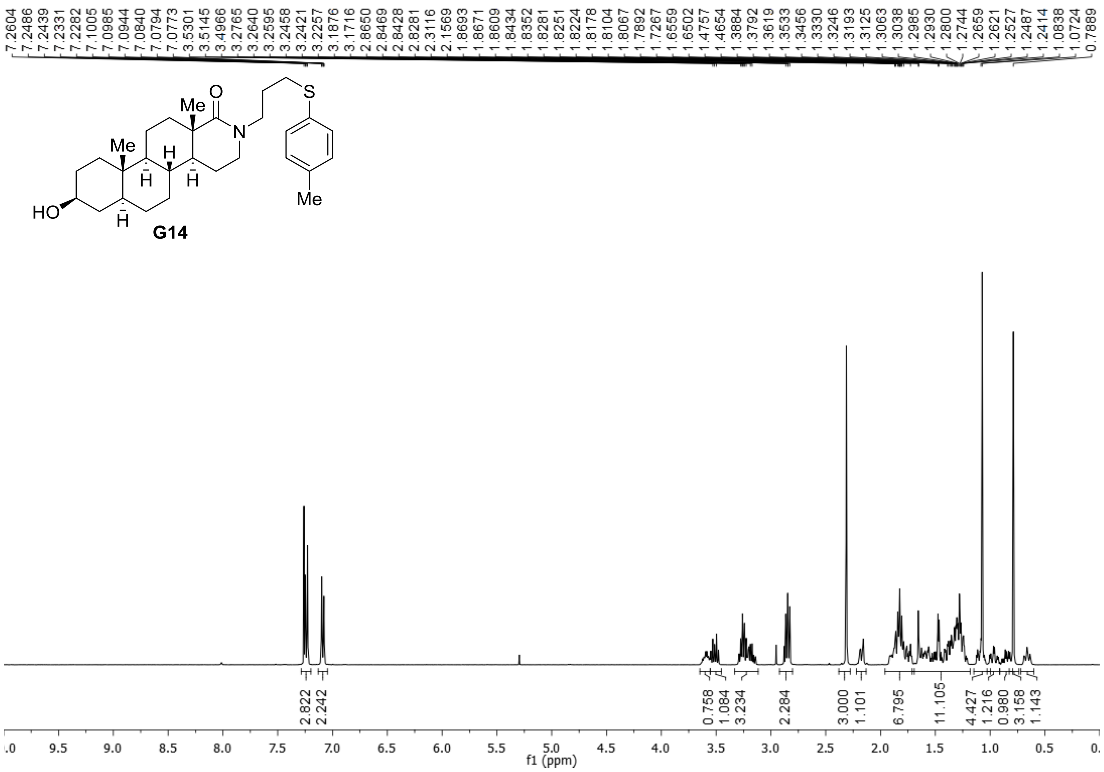

Supplementary Figure 334. ^{13}C NMR spectrum in $CDCl_3$, 101 MHz, of **G14**.

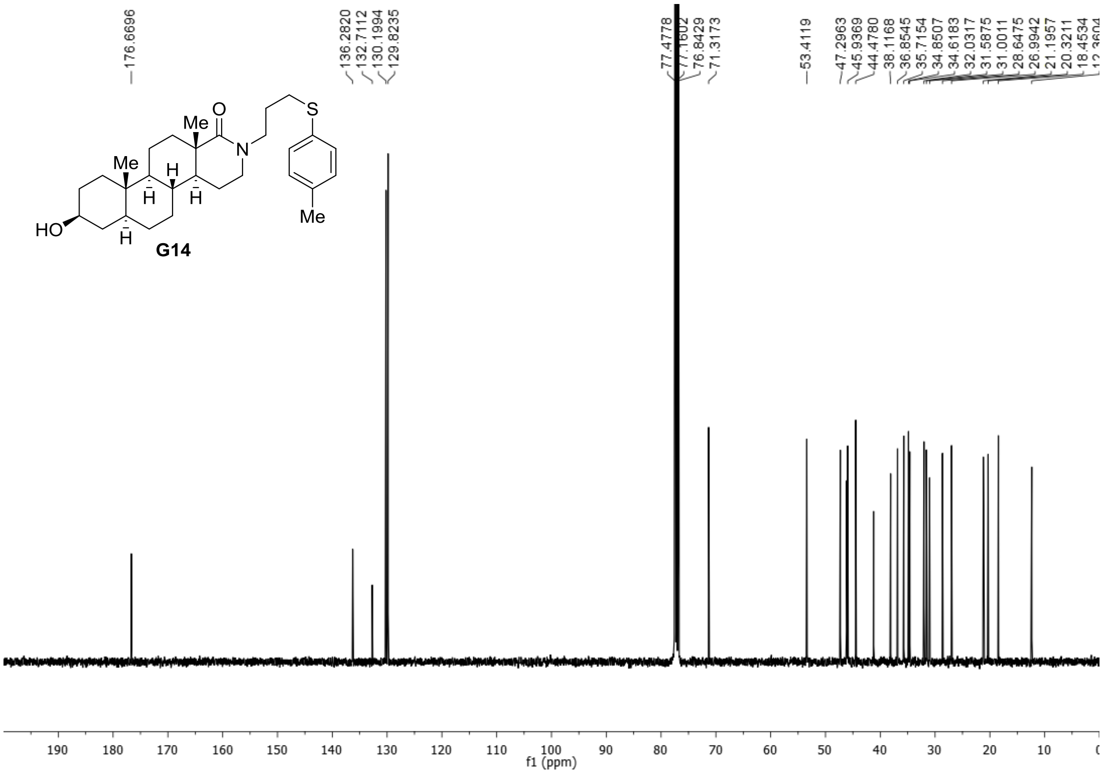

Supplementary Figure 335. ^{1}H NMR spectrum in $CDCl_3$, 400 MHz, of **G15**.

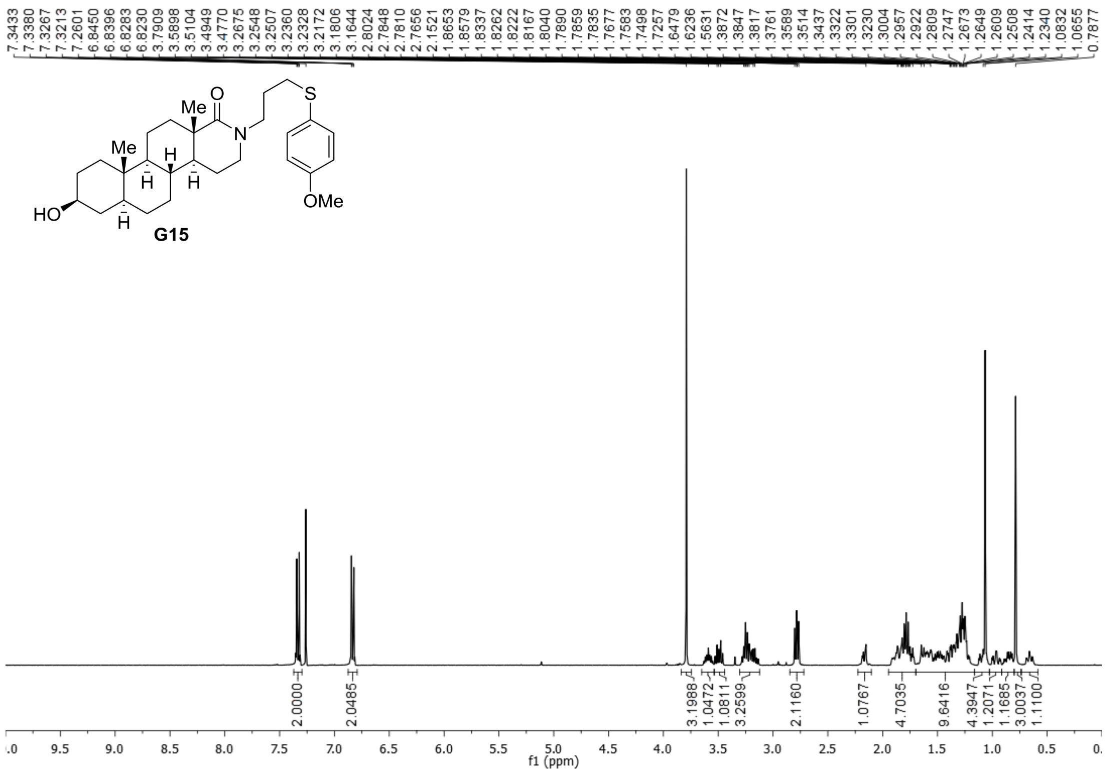

Supplementary Figure 336. ^{13}C NMR spectrum in $CDCl_3$, 101 MHz, of **G15**.

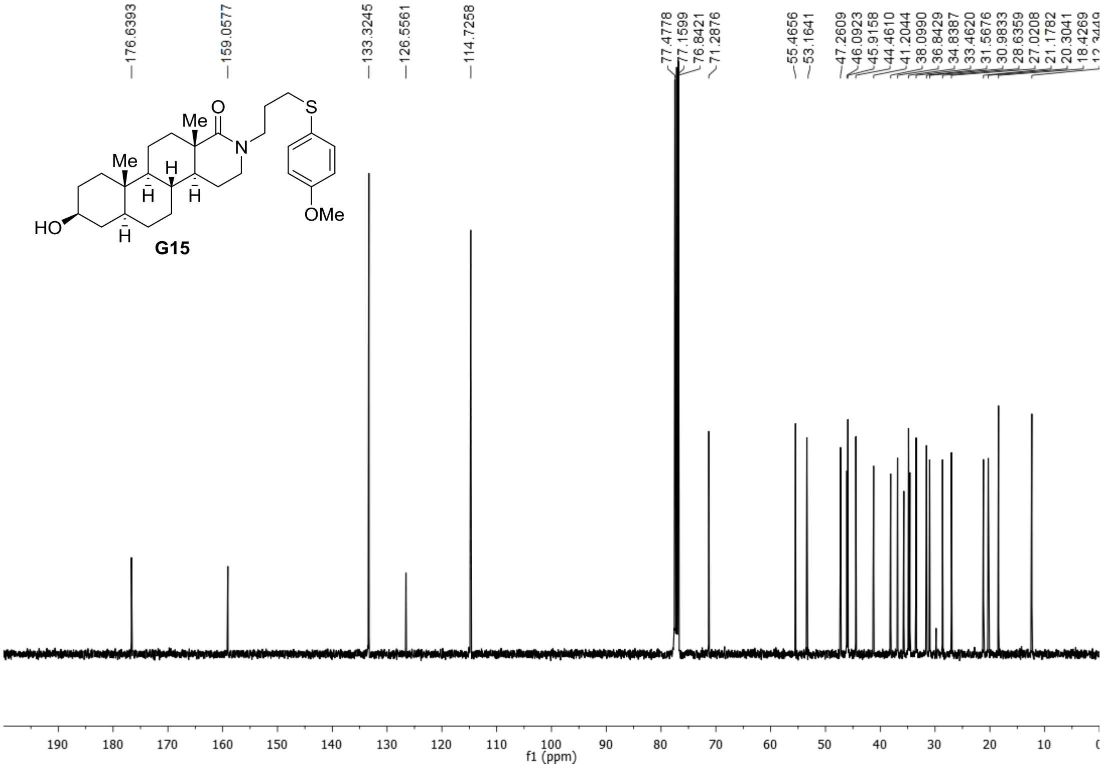

Supplementary Figure 337. ^{1}H NMR spectrum in $CDCl_3$, 400 MHz, of **G16**.

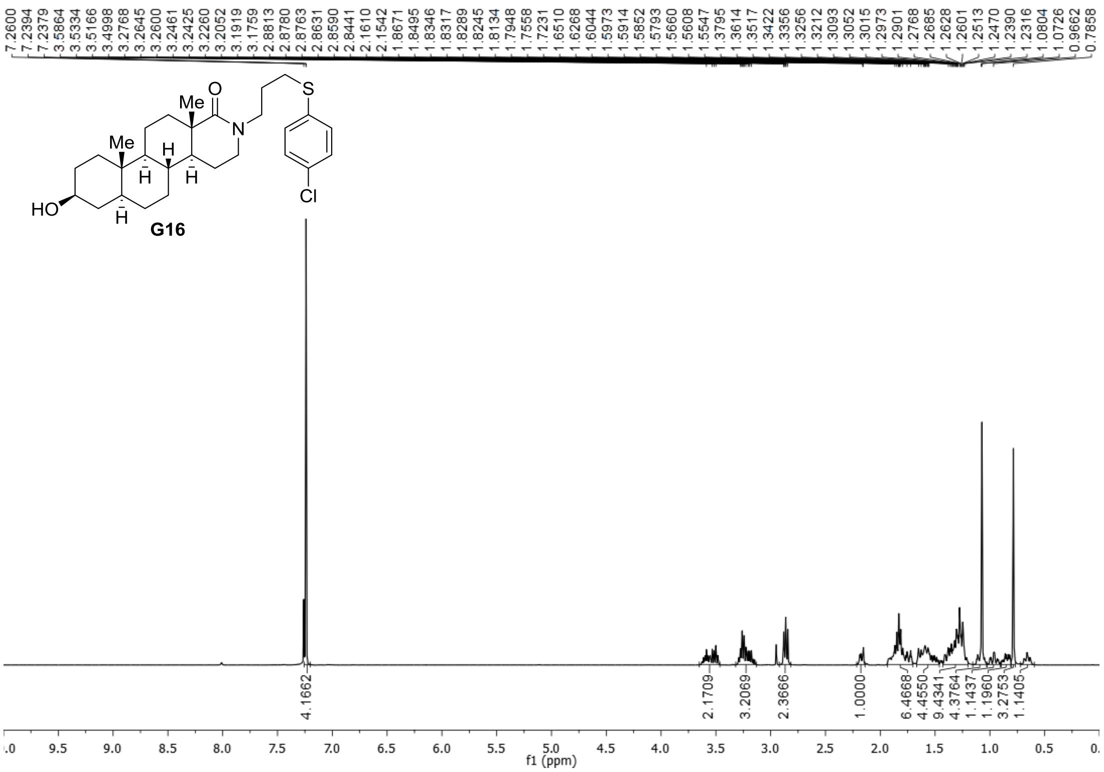

Supplementary Figure 338. ^{13}C NMR spectrum in $CDCl_3$, 101 MHz, of **G16**.

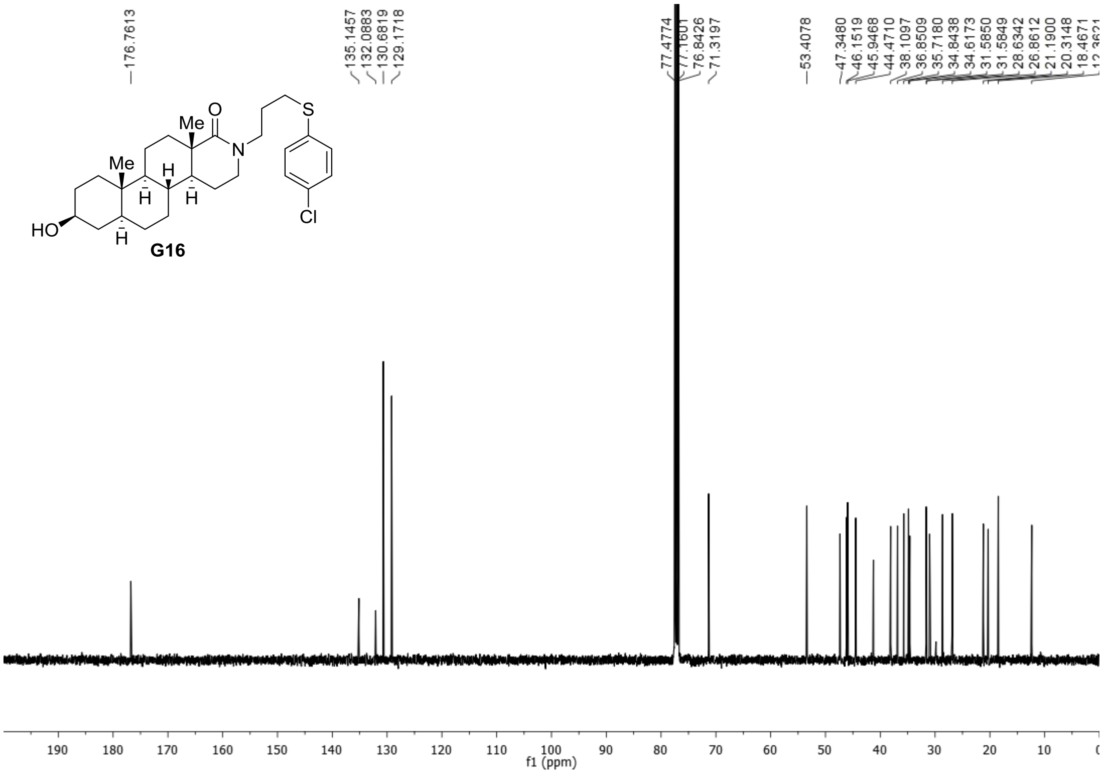

Supplementary Figure 339. ^{1}H NMR spectrum in $CDCl_3$, 400 MHz, of **G17**.

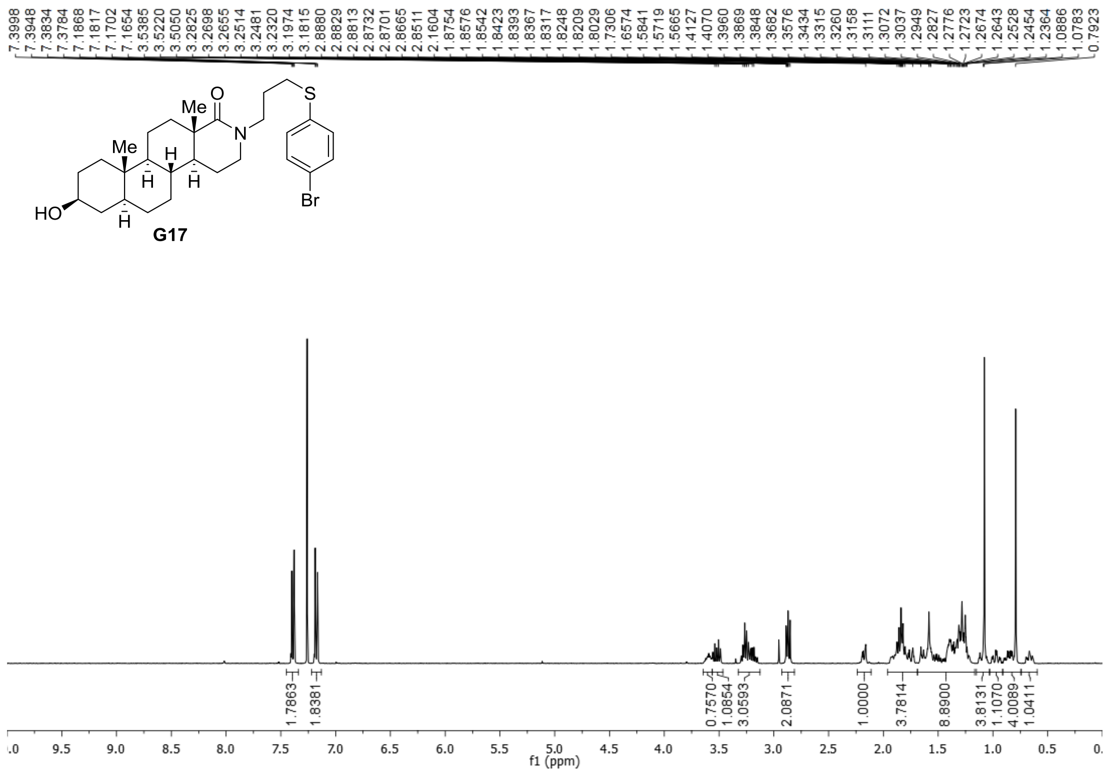

Supplementary Figure 340. ^{13}C NMR spectrum in $CDCl_3$, 101 MHz, of **G17**.

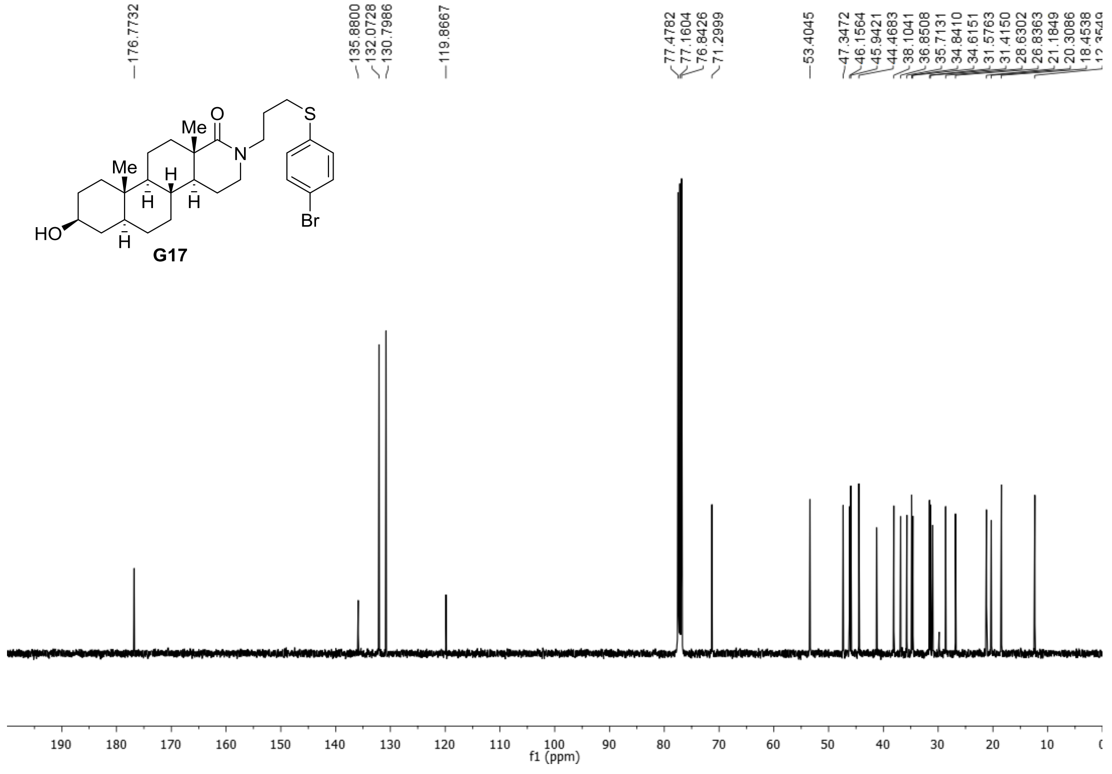

Supplementary Figure 341. ^{1}H NMR spectrum in $CDCl_3$, 400 MHz, of **G18**.

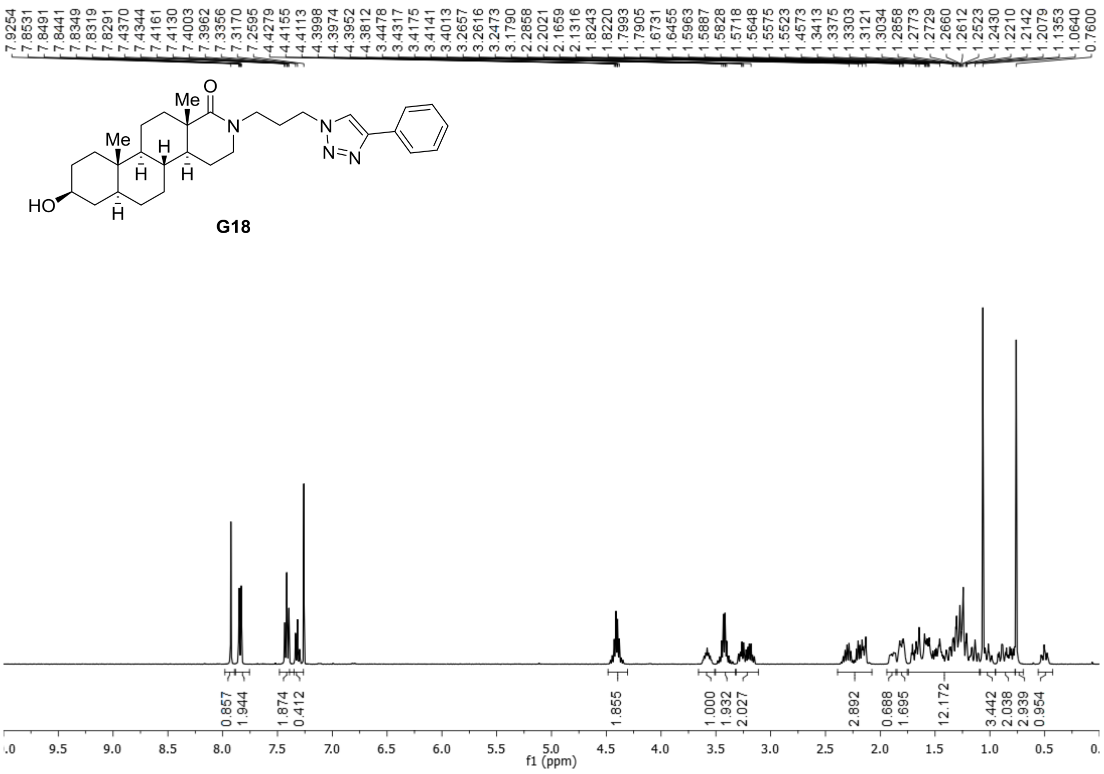

Supplementary Figure 342. ^{13}C NMR spectrum in $CDCl_3$, 101 MHz, of **G18**.

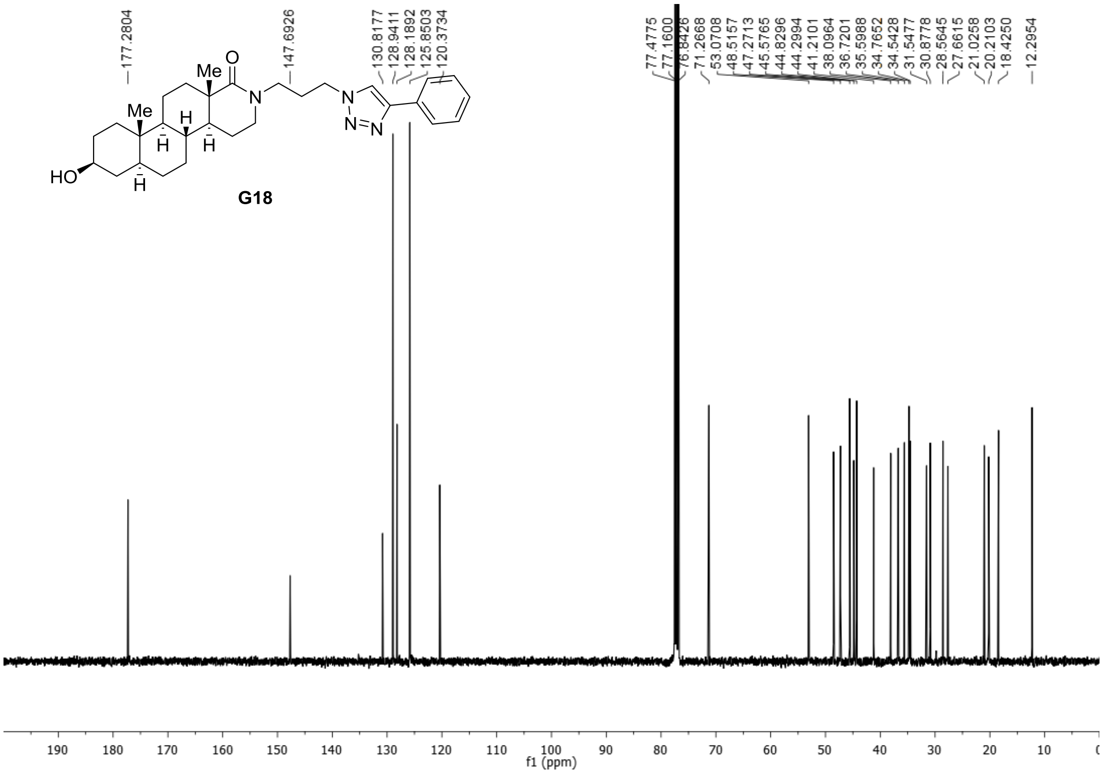

Supplementary Figure 343. ^{1}H NMR spectrum in $CDCl_3$, 400 MHz, of **G19**.

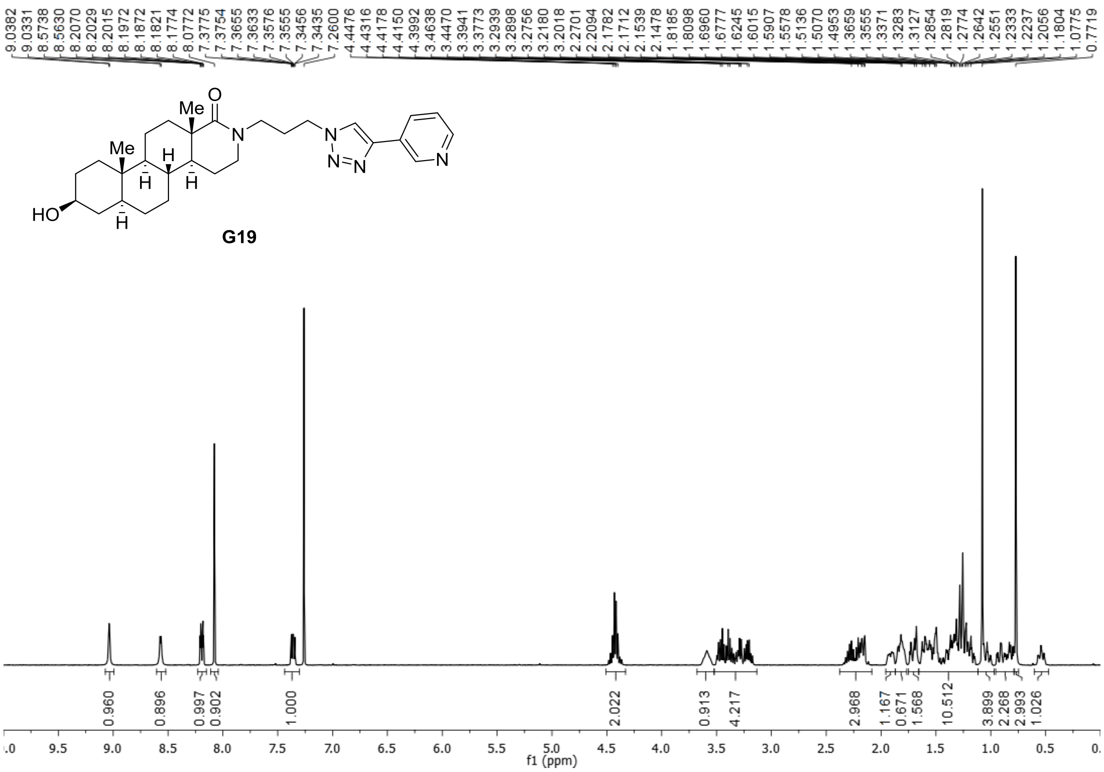

Supplementary Figure 344. ^{13}C NMR spectrum in $CDCl_3$, 101 MHz, of **G19**.

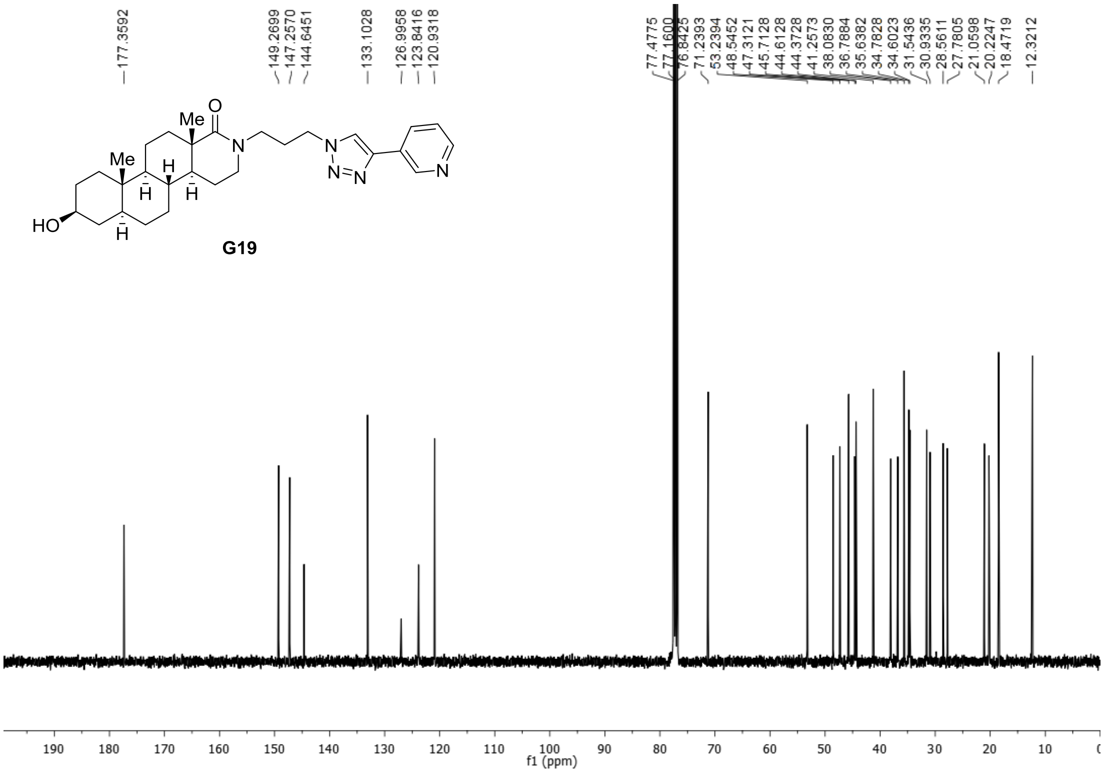

Supplementary Figure 345. ^{1}H NMR spectrum in $CDCl_3$, 400 MHz, of **G20**.

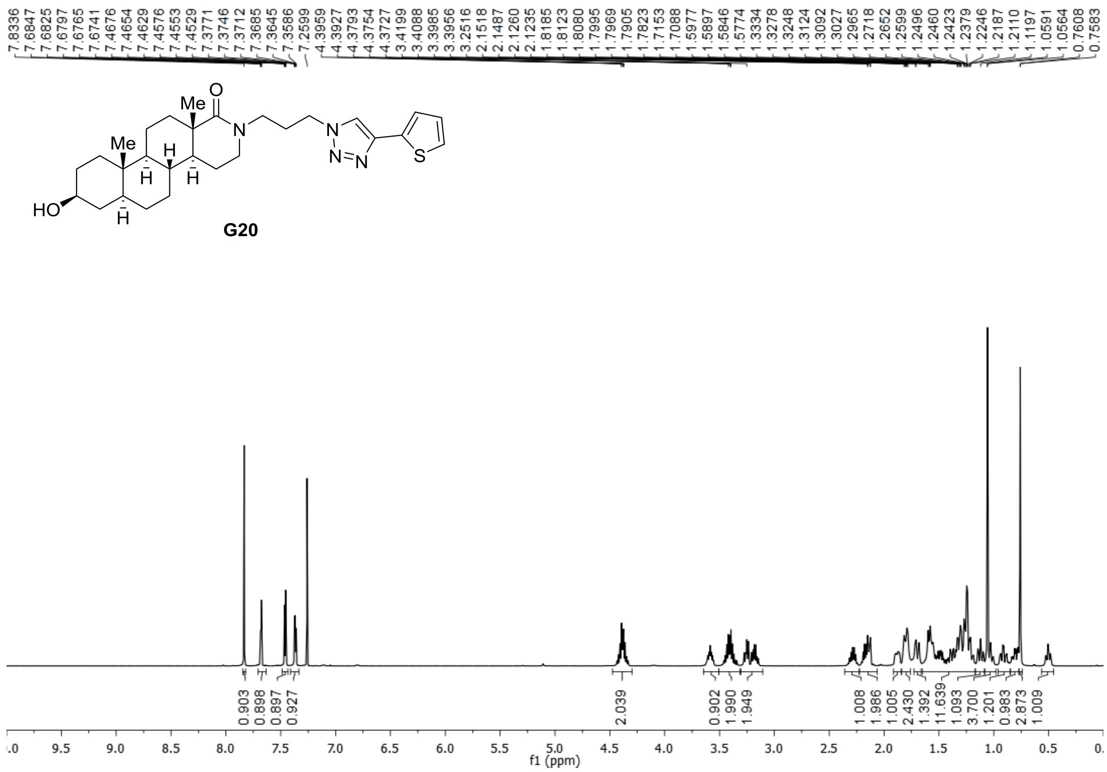

Supplementary Figure 346. ^{13}C NMR spectrum in $CDCl_3$, 126 MHz, of **G20**.

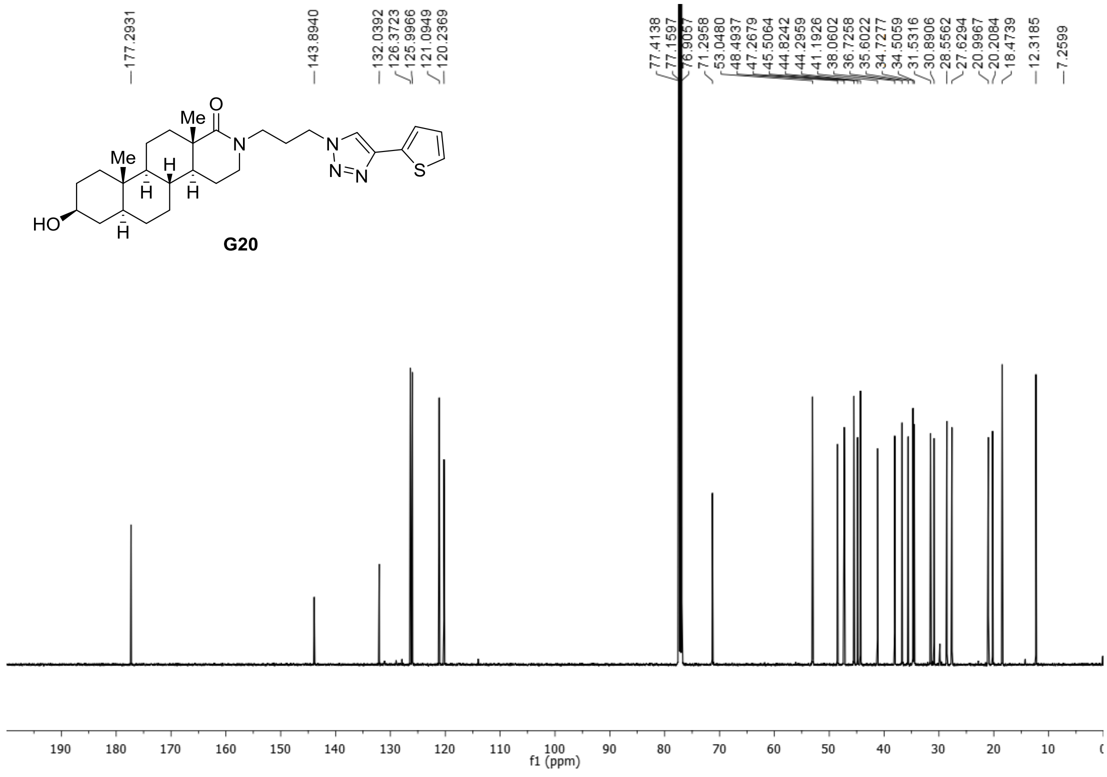

Supplementary Figure 347. ^{1}H NMR spectrum in $CDCl_3$, 400 MHz, of **G21**.

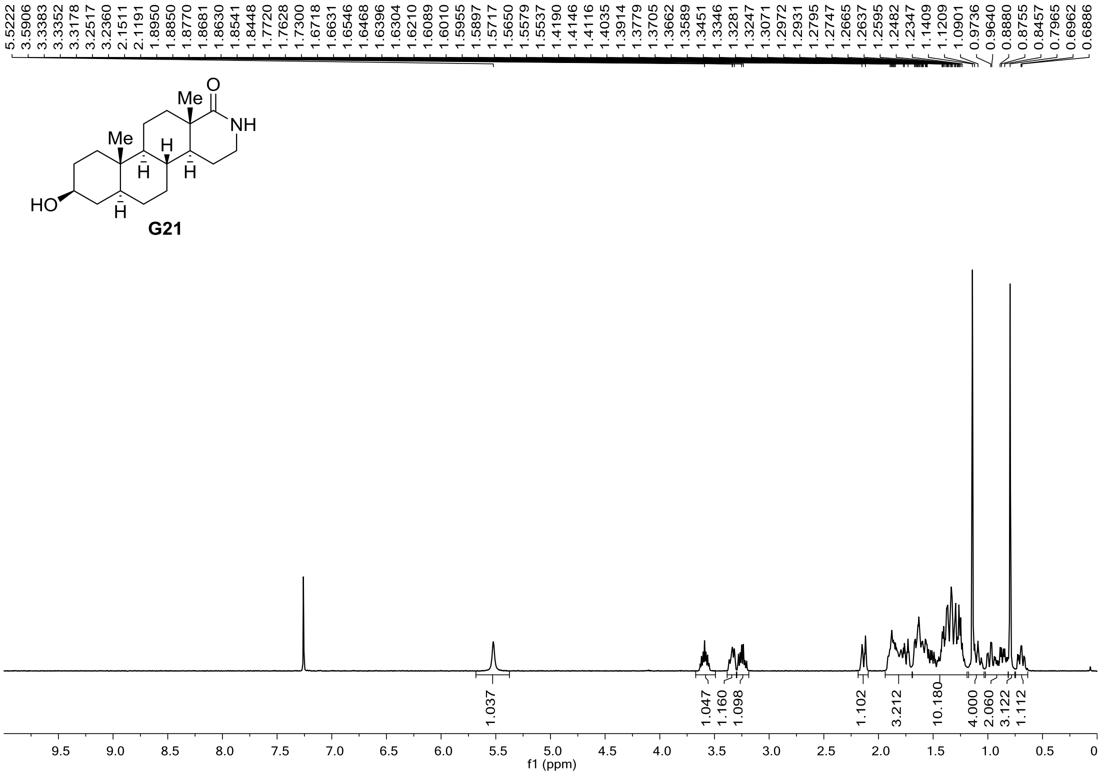

Supplementary Figure 348. ^{13}C NMR (APT) spectrum in $CDCl_3$, 101 MHz, of **G21**.

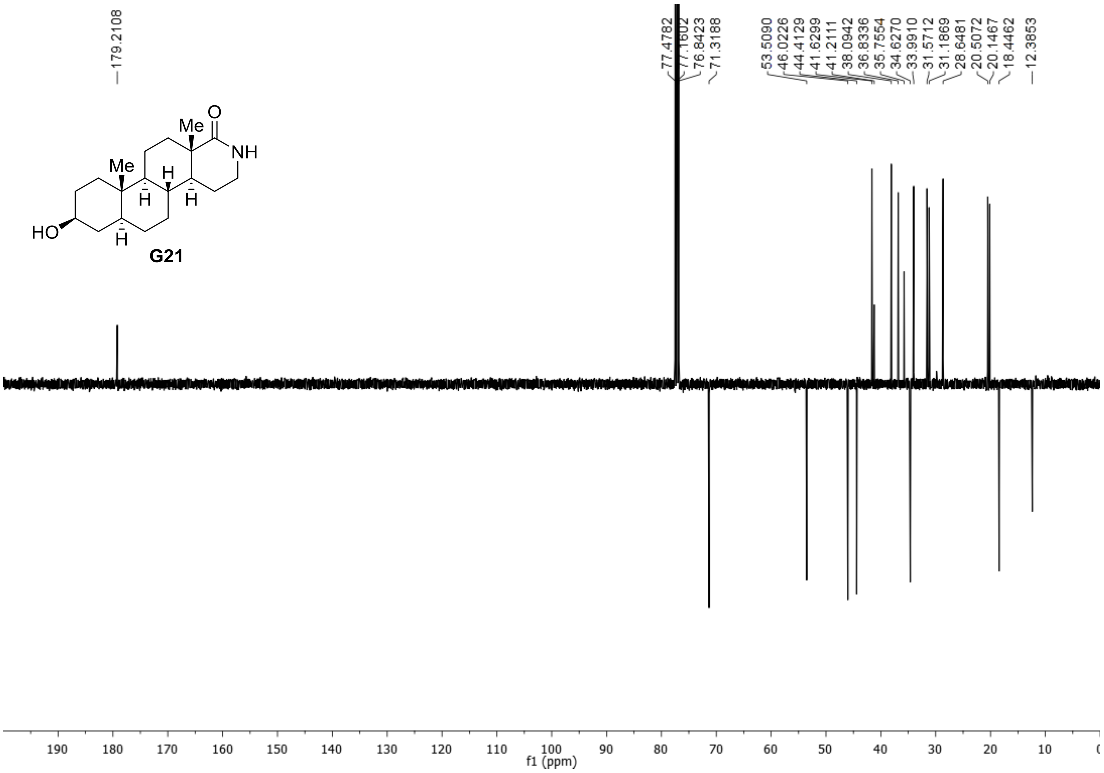

Supplementary Figure 349. ^{1}H NMR spectrum in $CDCl_3$, 400 MHz, of **H2**.

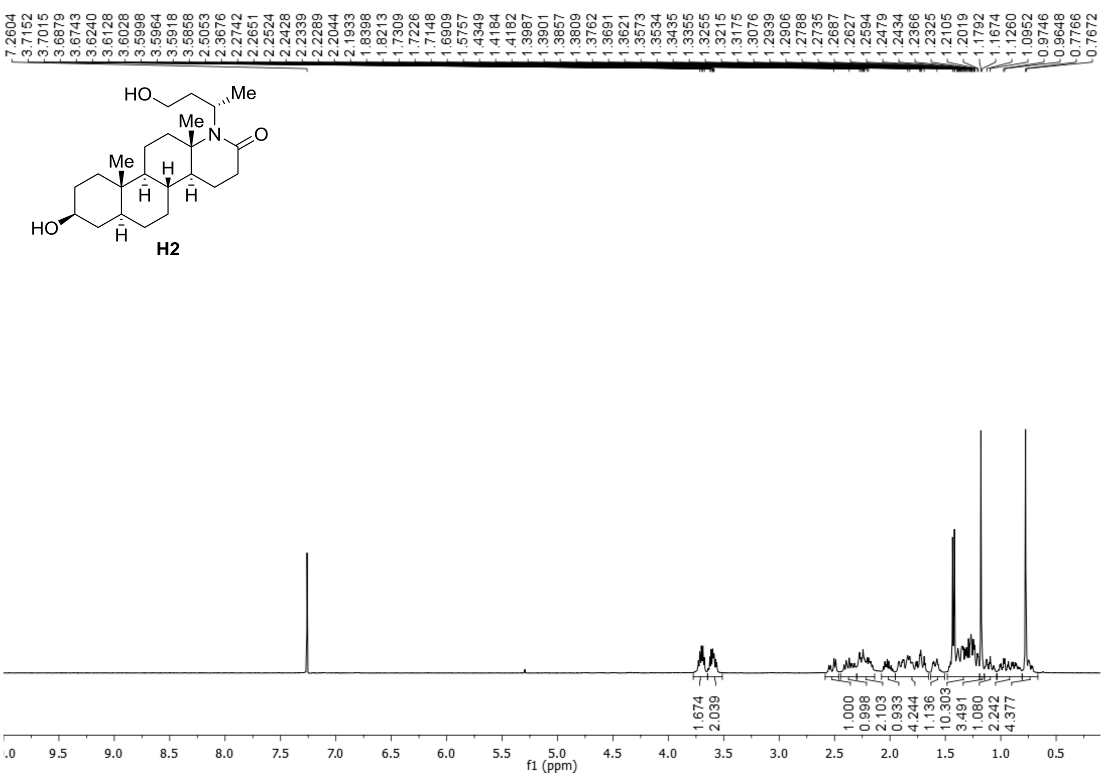

Supplementary Figure 350. ^{13}C NMR spectrum in $CDCl_3$, 101 MHz, of **H2**.

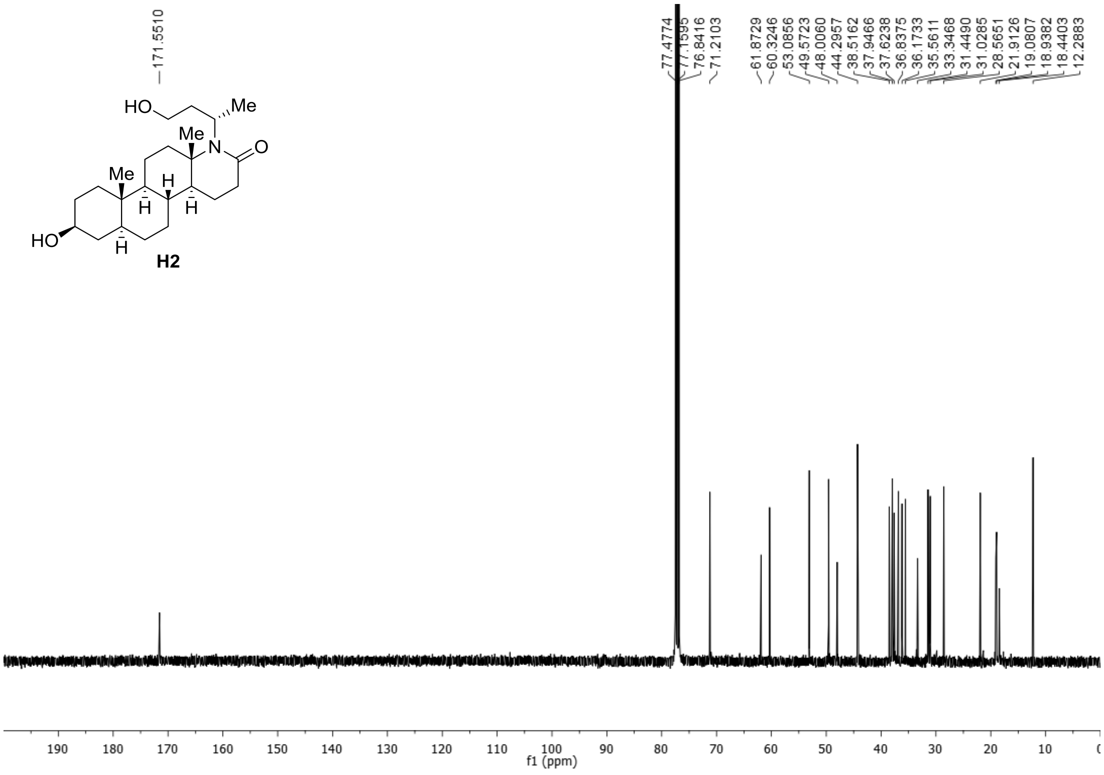

Supplementary Figure 351. ^{13}C NMR (APT) spectrum in $CDCl_3$, 101 MHz, of **H2**.

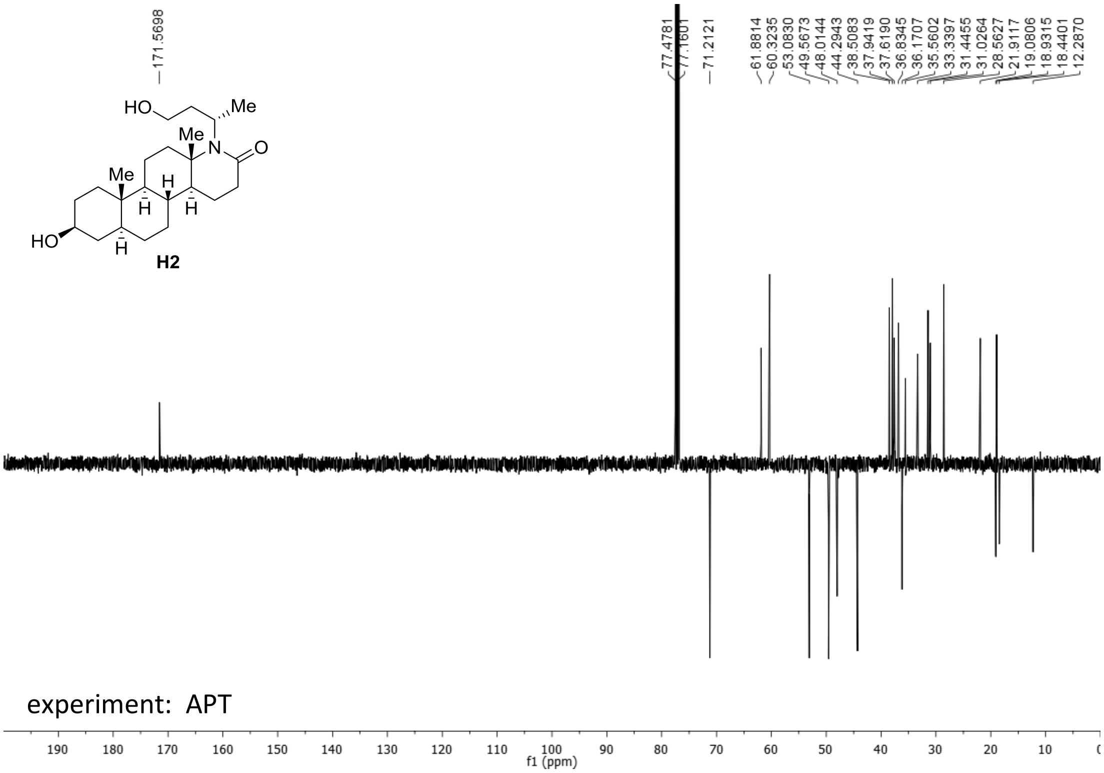

Supplementary Figure 352. 1D NOE spectrum in $CDCl_3$, 400 MHz, of **H2**.

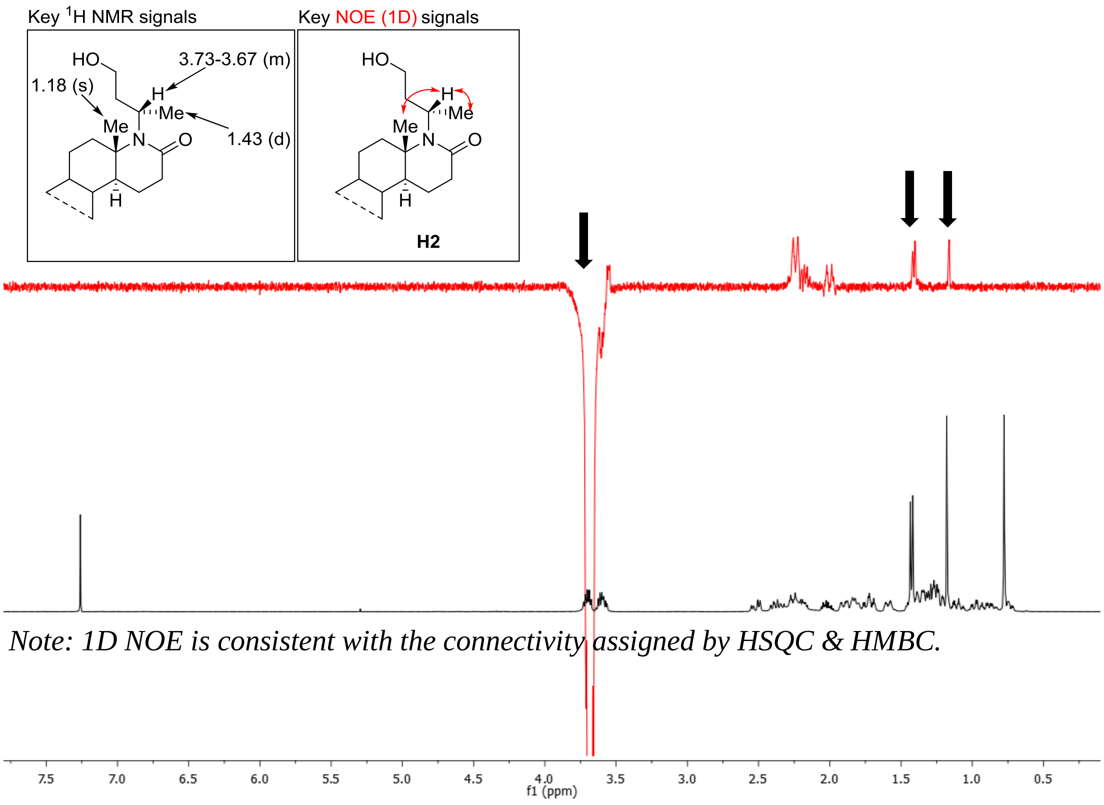

Supplementary Figure 353. HSQC spectrum of **H2**.

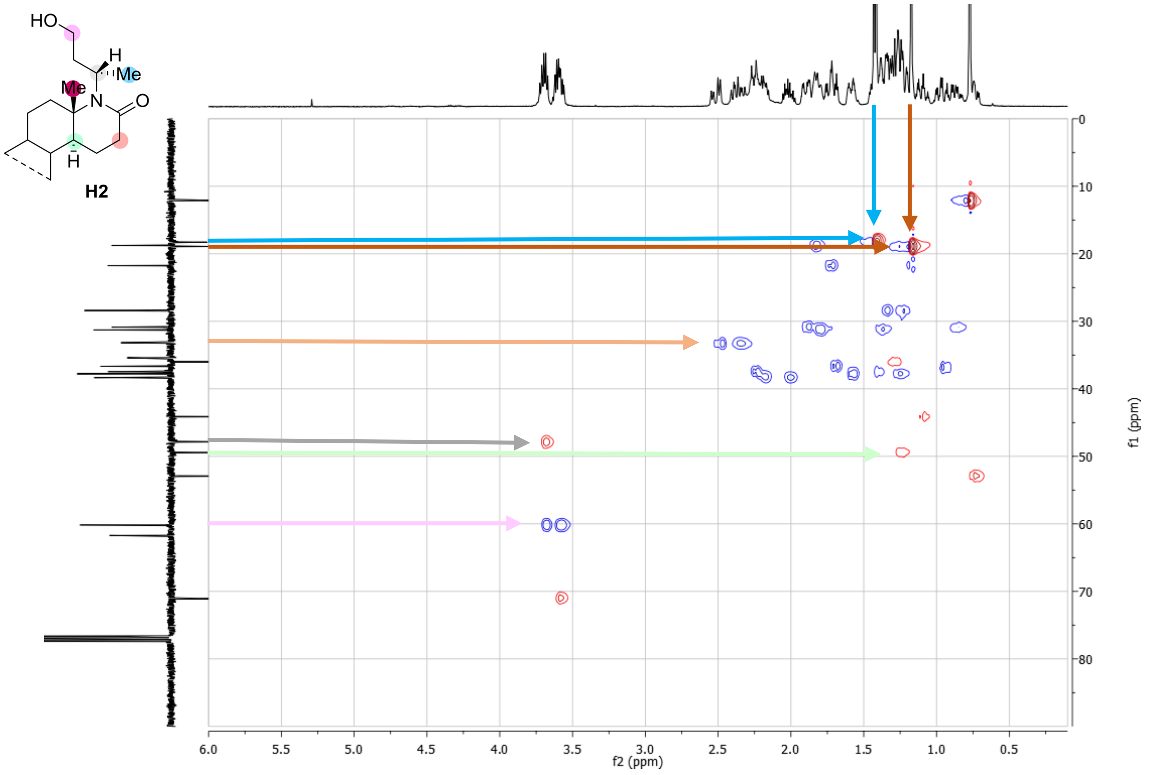

Supplementary Figure 354. HMBC spectrum of **H2**.

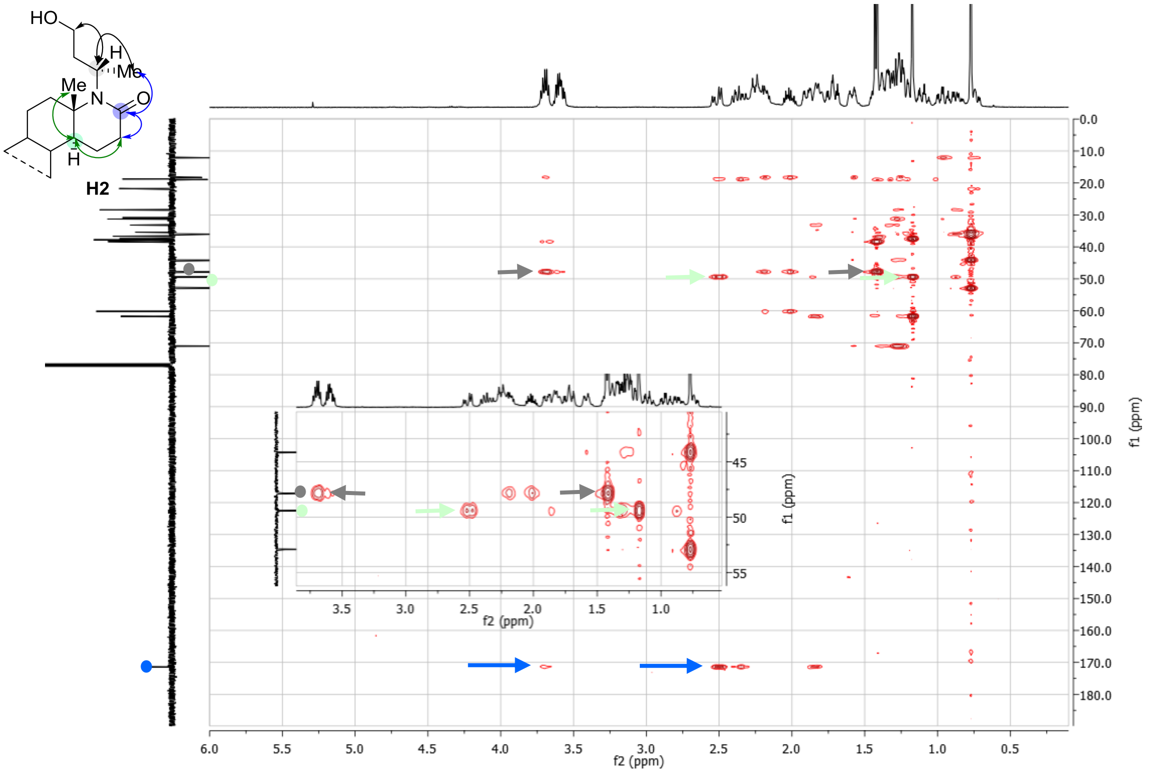

Supplementary Figure 355. ^{1}H NMR spectrum in $CDCl_3$, 400 MHz, of **G5** and **H3**.

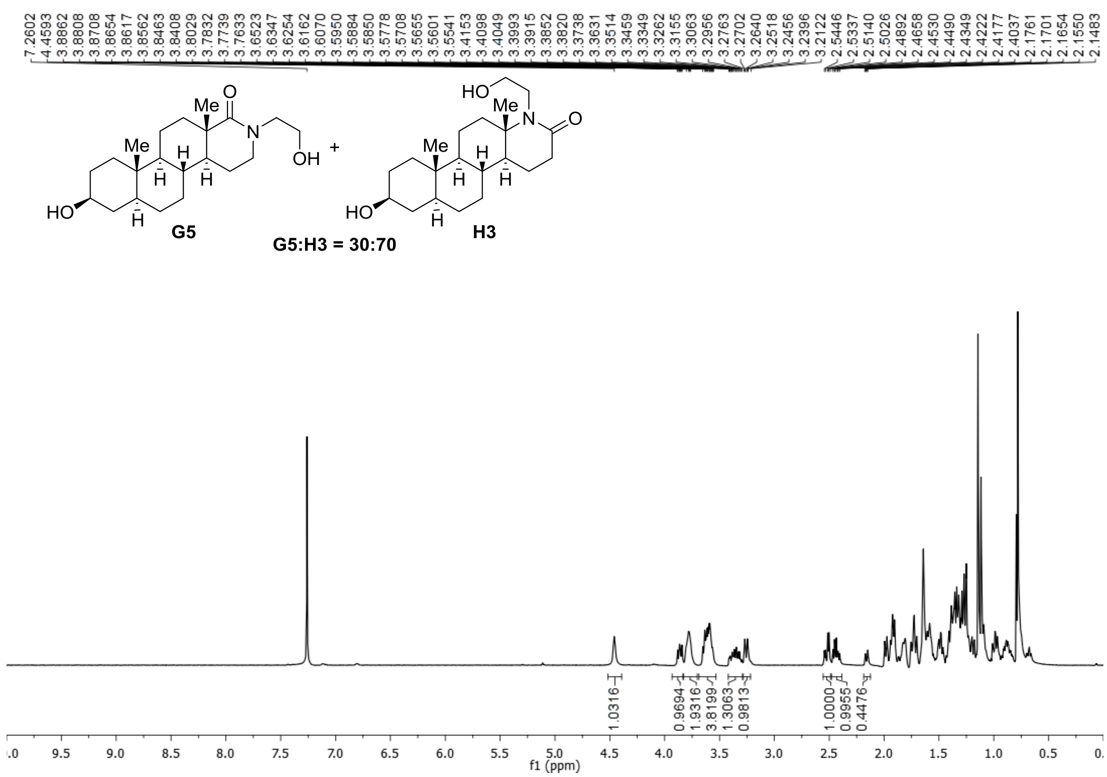

Supplementary Figure 356. ^{13}C NMR spectrum in $CDCl_3$, 151 MHz, of **G5** and **H3**.

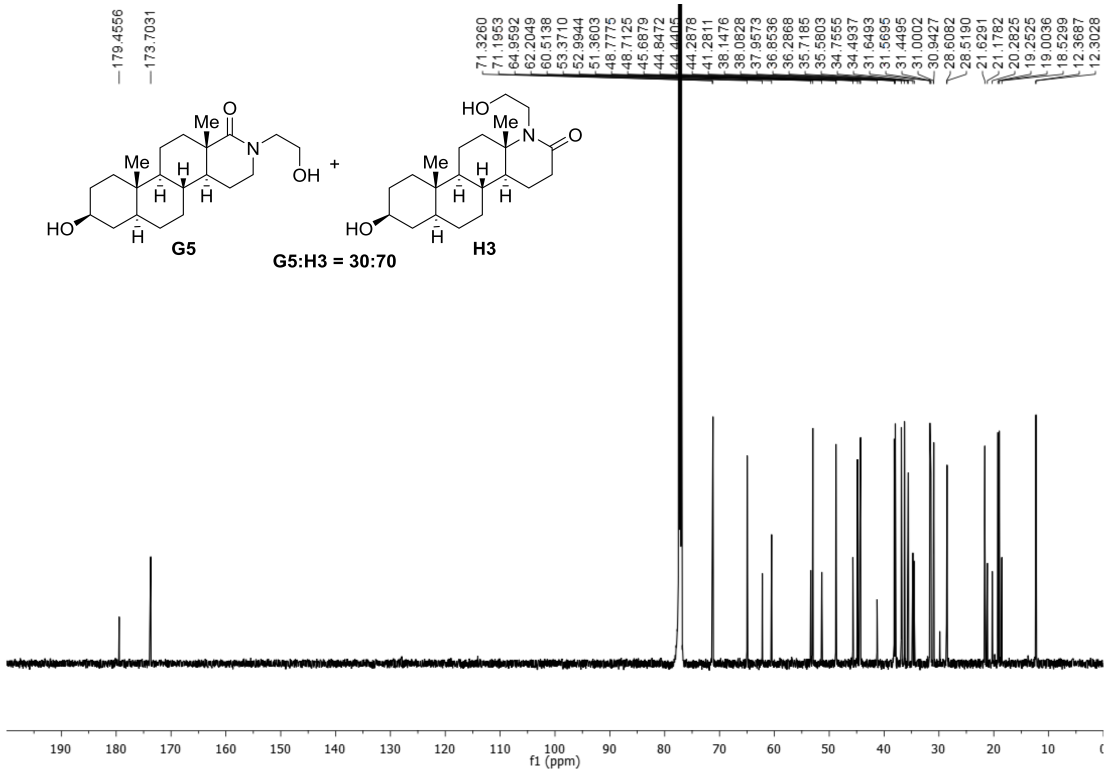

Supplementary Figure 357. ^{1}H NMR spectrum in $CDCl_3$, 400 MHz, of **H4**.

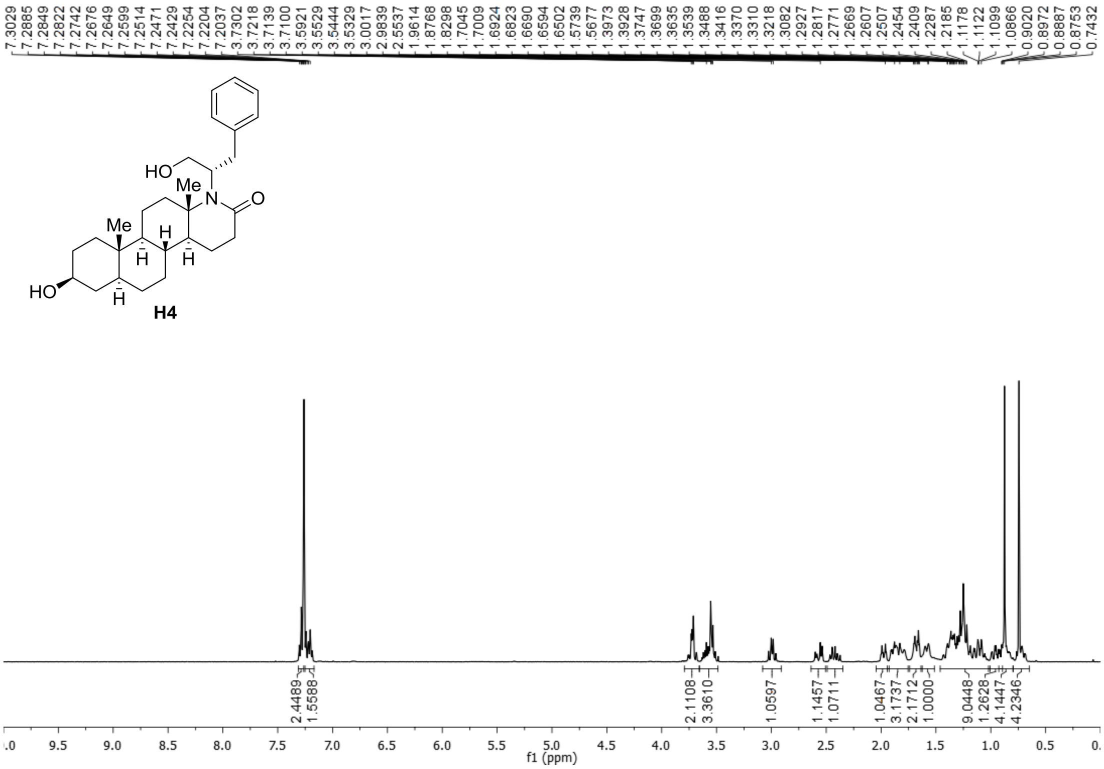

Supplementary Figure 358. ^{13}C NMR spectrum in $CDCl_3$, 101 MHz, of **H4**.

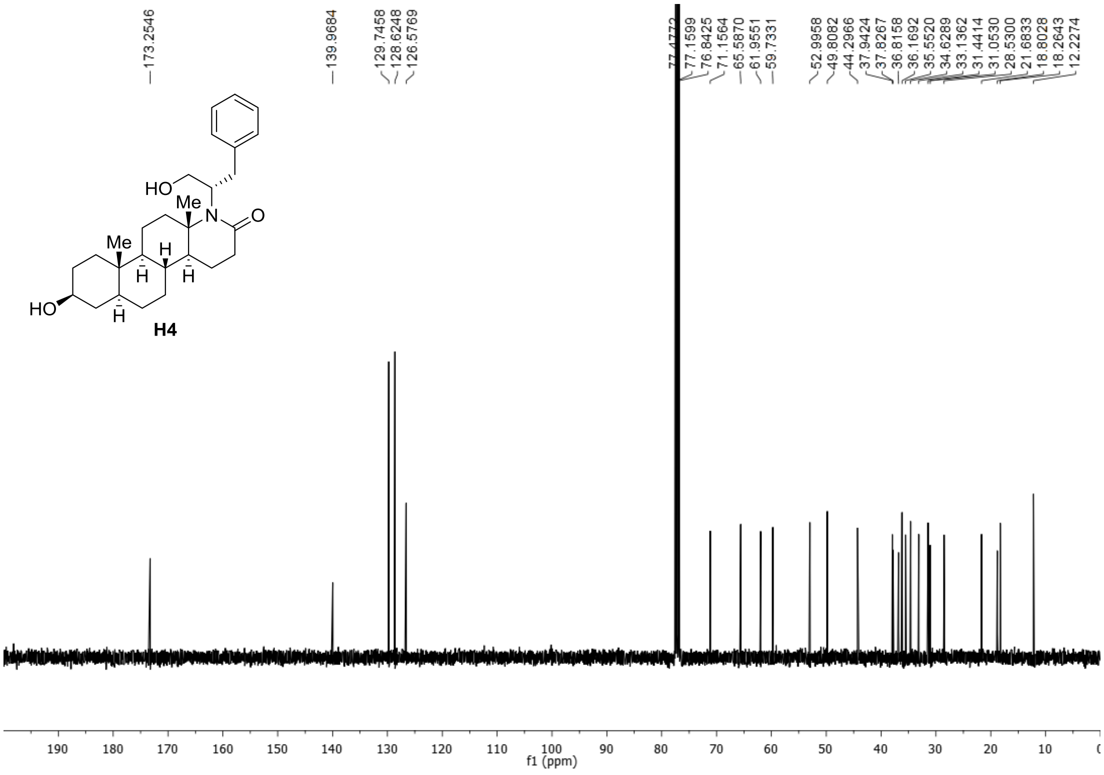

Supplementary Figure 359. ^{1}H NMR spectrum in $CDCl_3$, 400 MHz, of **H5**.

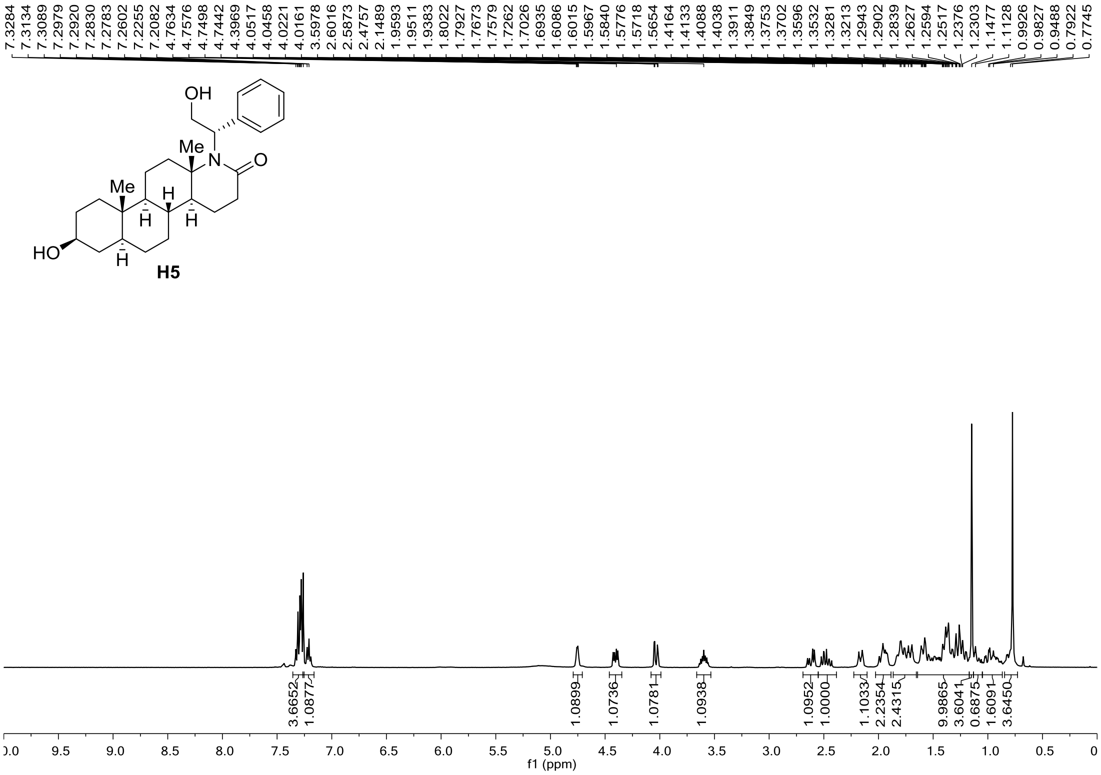

Supplementary Figure 360. ^{13}C NMR spectrum in $CDCl_3$, 101 MHz, of **H5**.

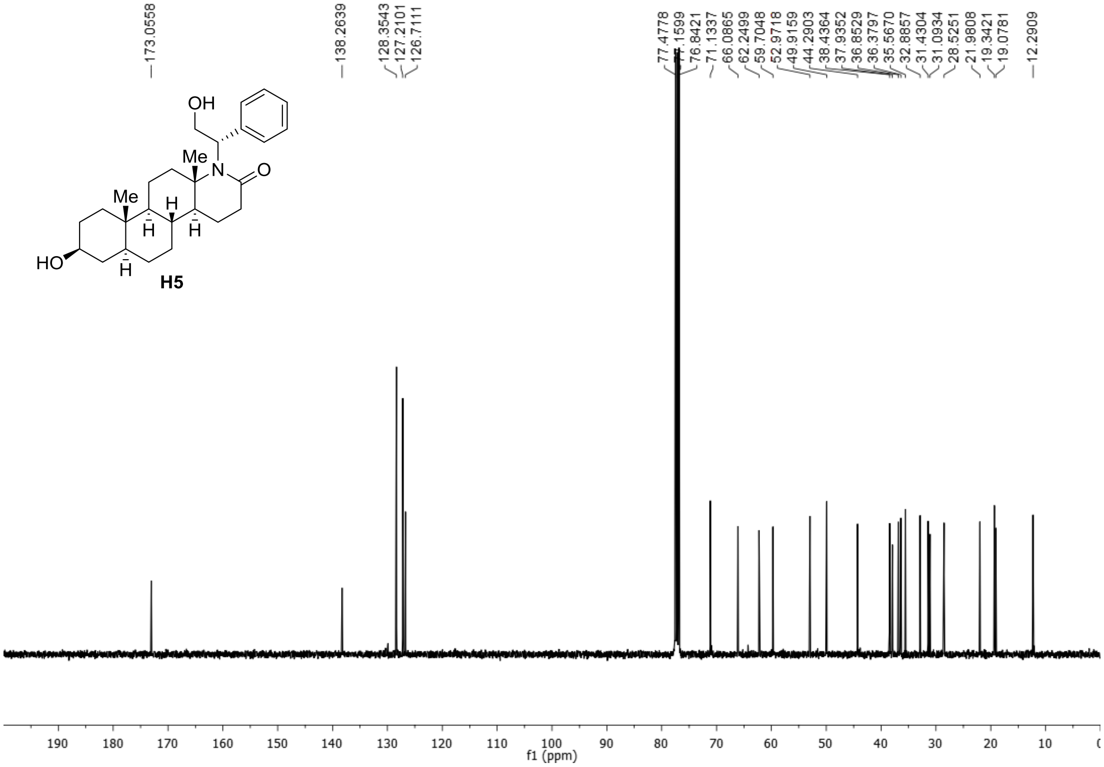

Supplementary Figure 361. ^{1}H NMR spectrum in $CDCl_3$, 400 MHz, of **H5**.

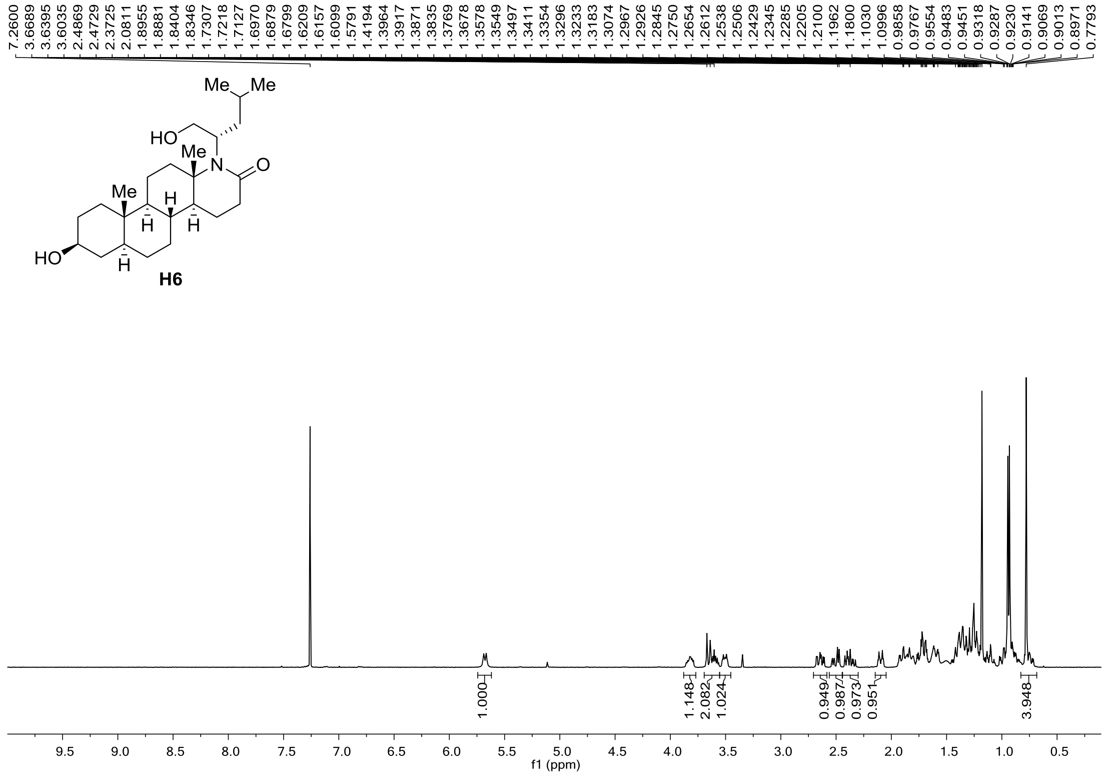

Supplementary Figure 362. ^{13}C NMR spectrum in $CDCl_3$, 101 MHz, of **H5**.

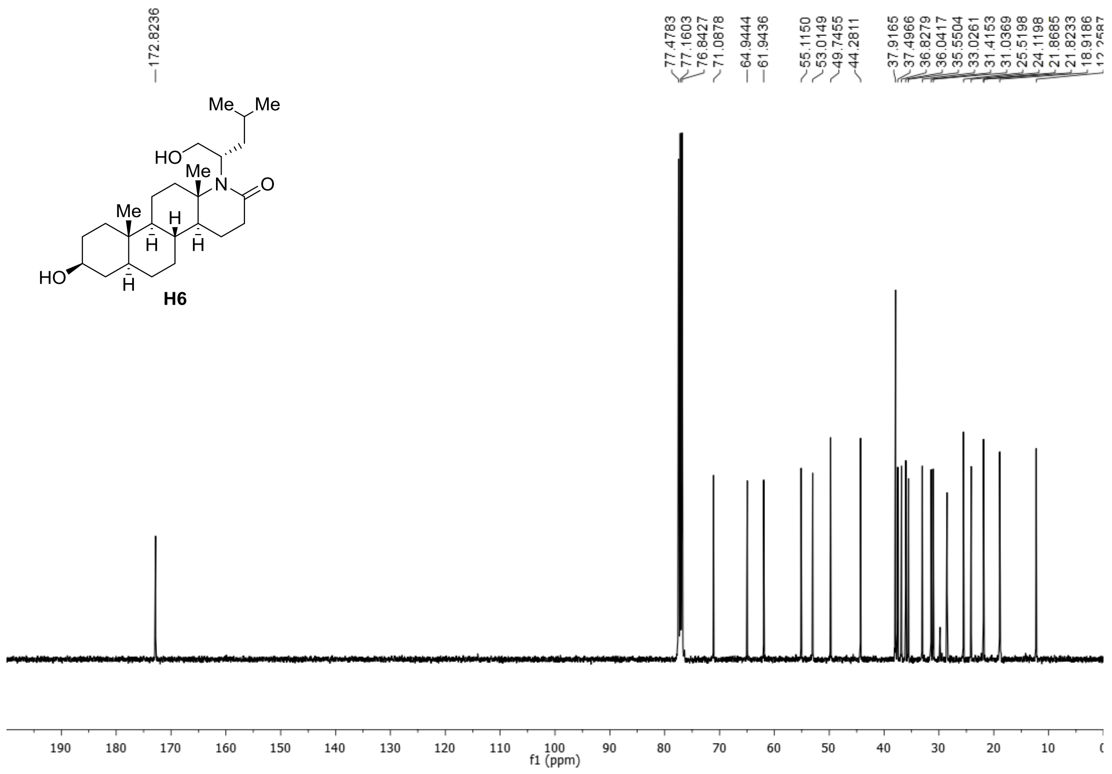

Supplementary Figure 363. ^{1}H NMR spectrum in $CDCl_3$, 400 MHz, of **Beck1**.

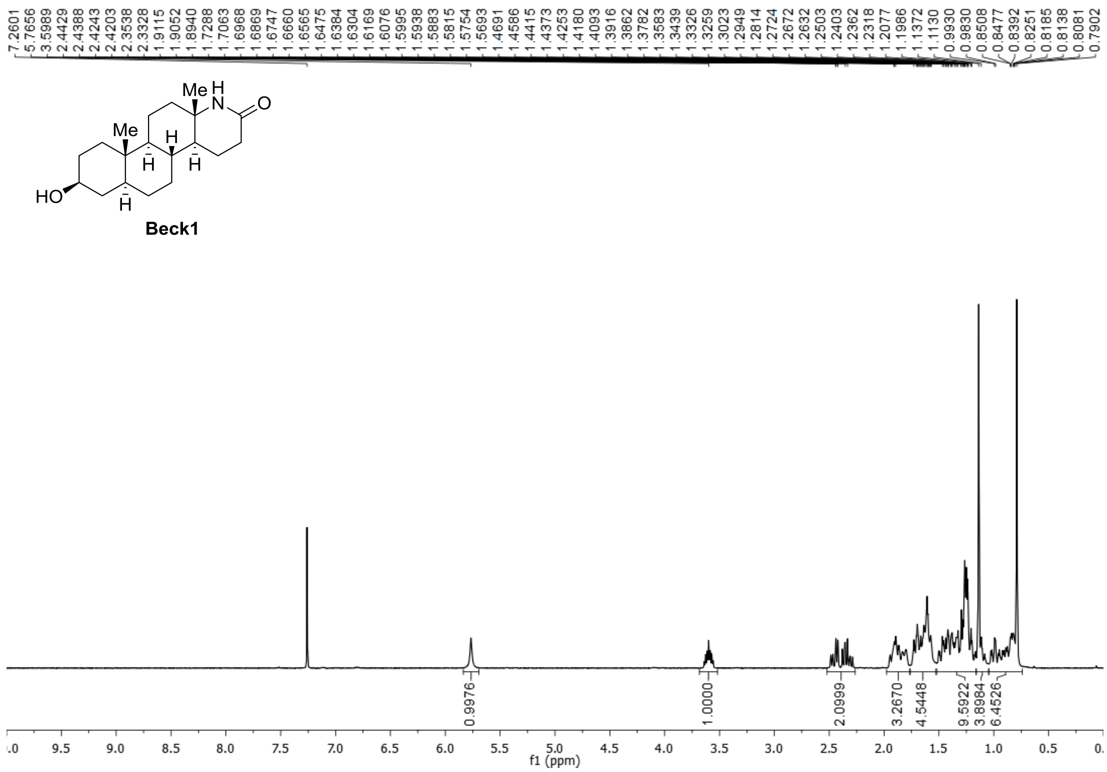

Supplementary Figure 364. ^{13}C NMR (APT) spectrum in $CDCl_3$, 101 MHz, of **Beck1**.

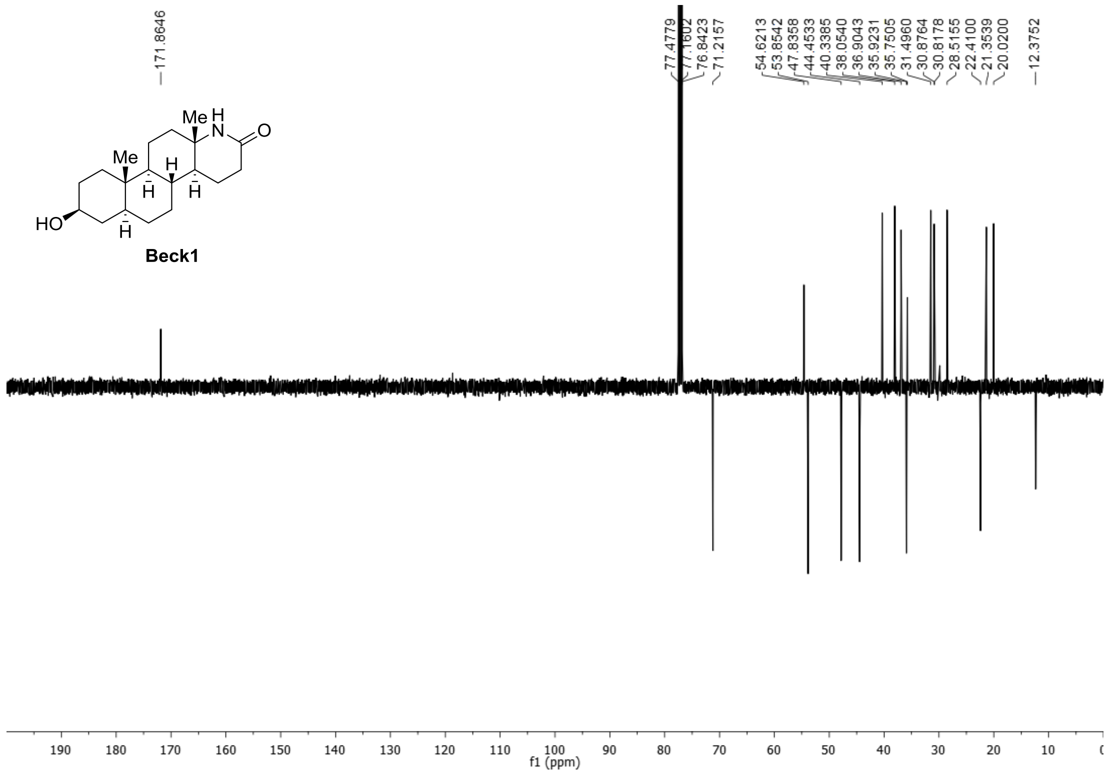

Supplementary Figure 365. ^{1}H NMR spectrum in DMSO-d_6, 400 MHz, of **I1**.

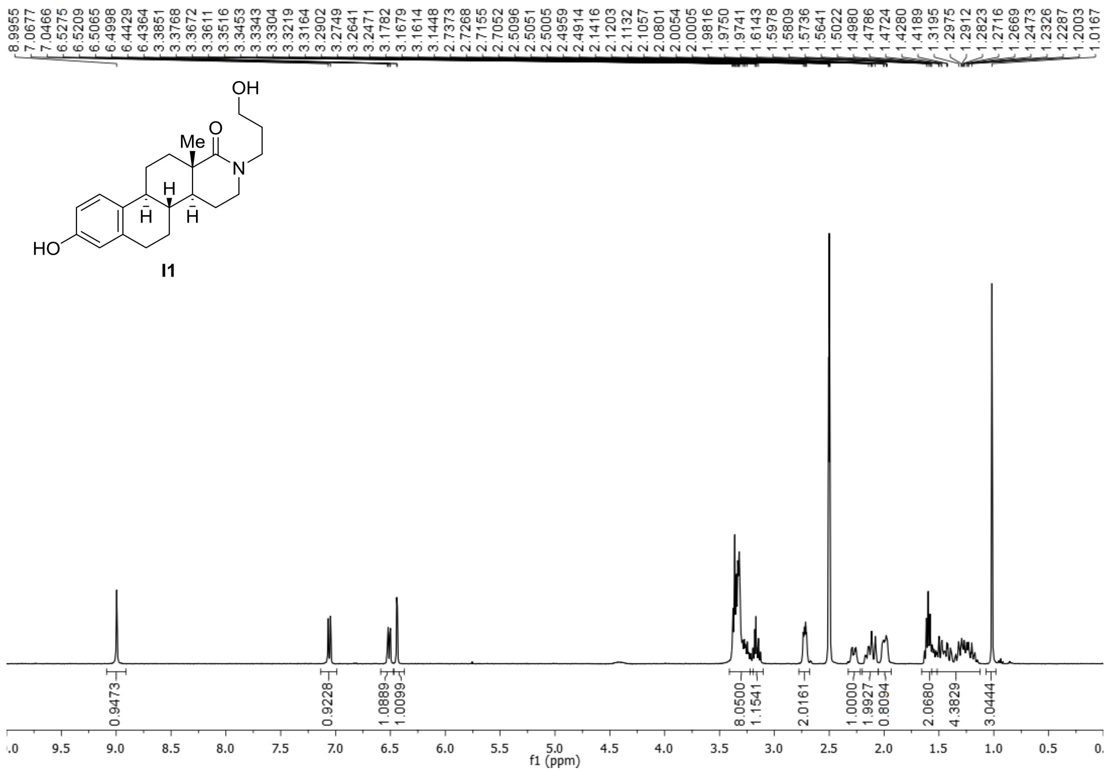

Supplementary Figure 366. ^{13}C NMR spectrum in DMSO-d_6, 126 MHz, of **I1**.

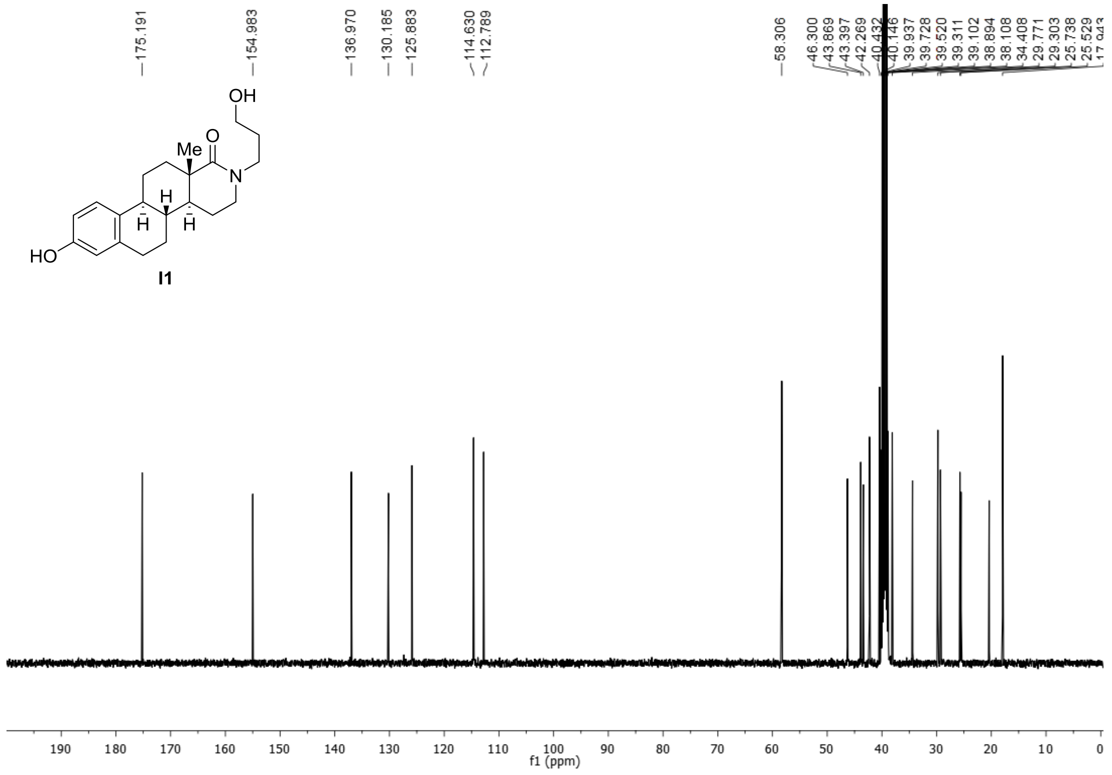

Supplementary Figure 367. ^{1}H NMR spectrum in DMSO-d_6, 400 MHz, of **I2**.

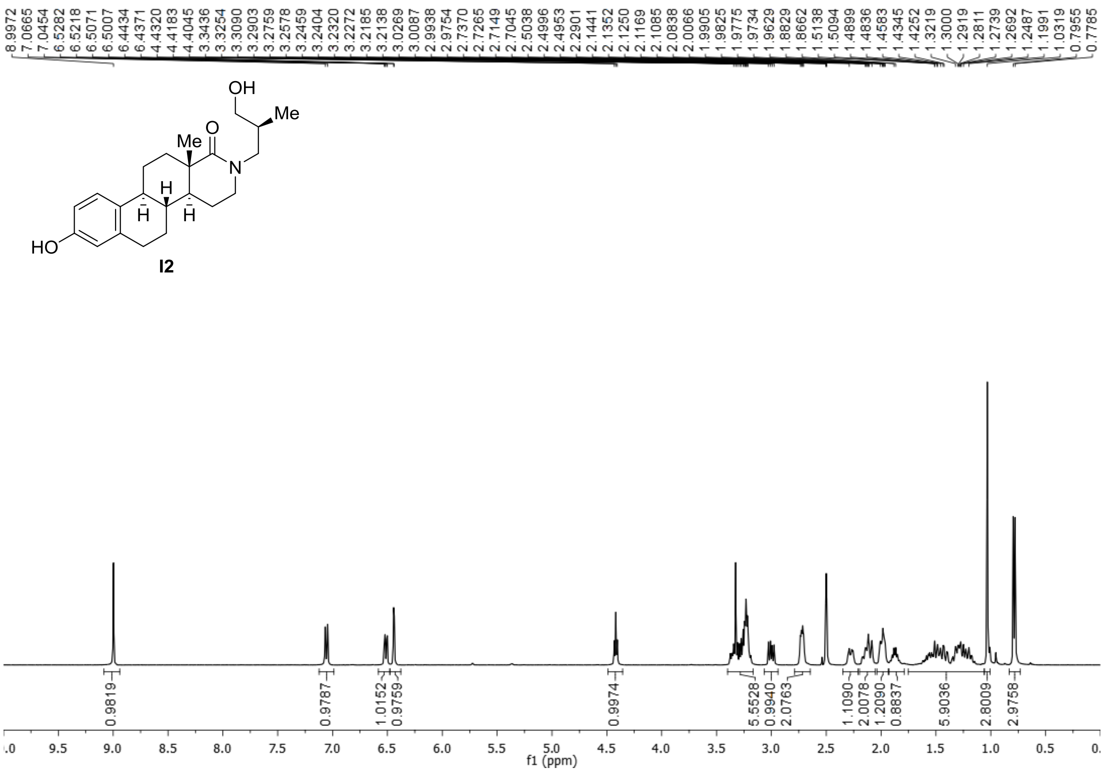

Supplementary Figure 368. ^{13}C NMR spectrum in DMSO-d_6, 126 MHz, of **I2**.

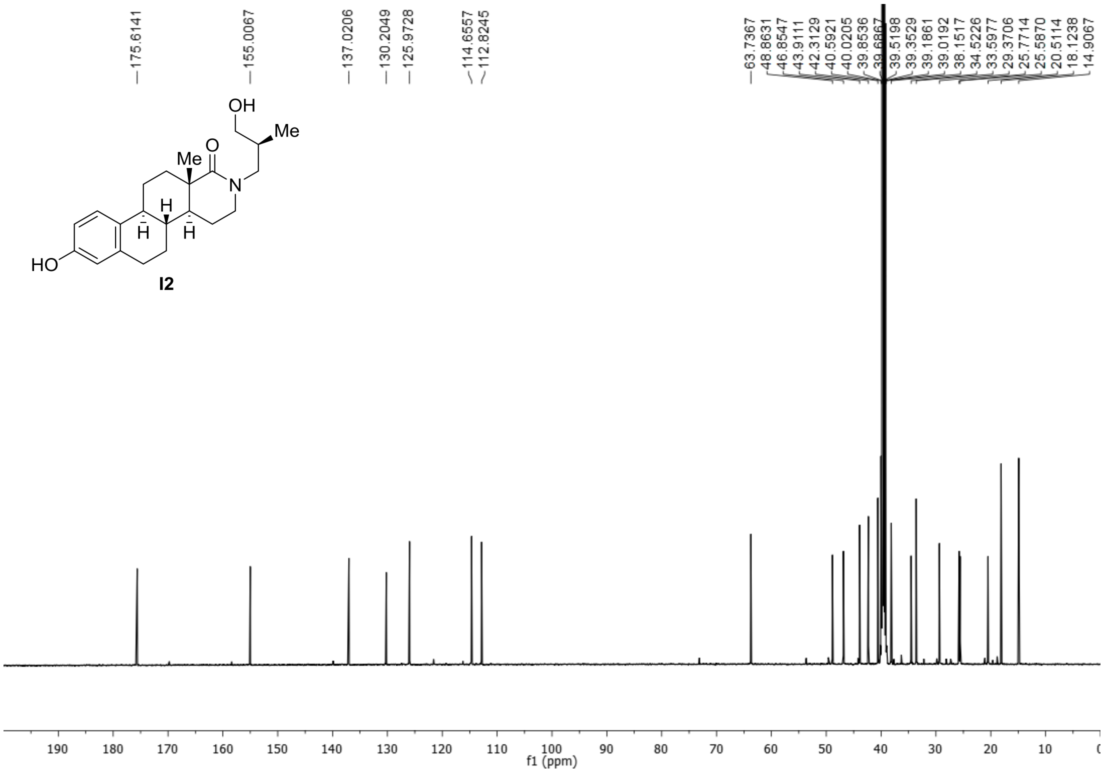

Supplementary Figure 369. ^{1}H NMR spectrum in DMSO-d_6, 400 MHz, of **J1**.

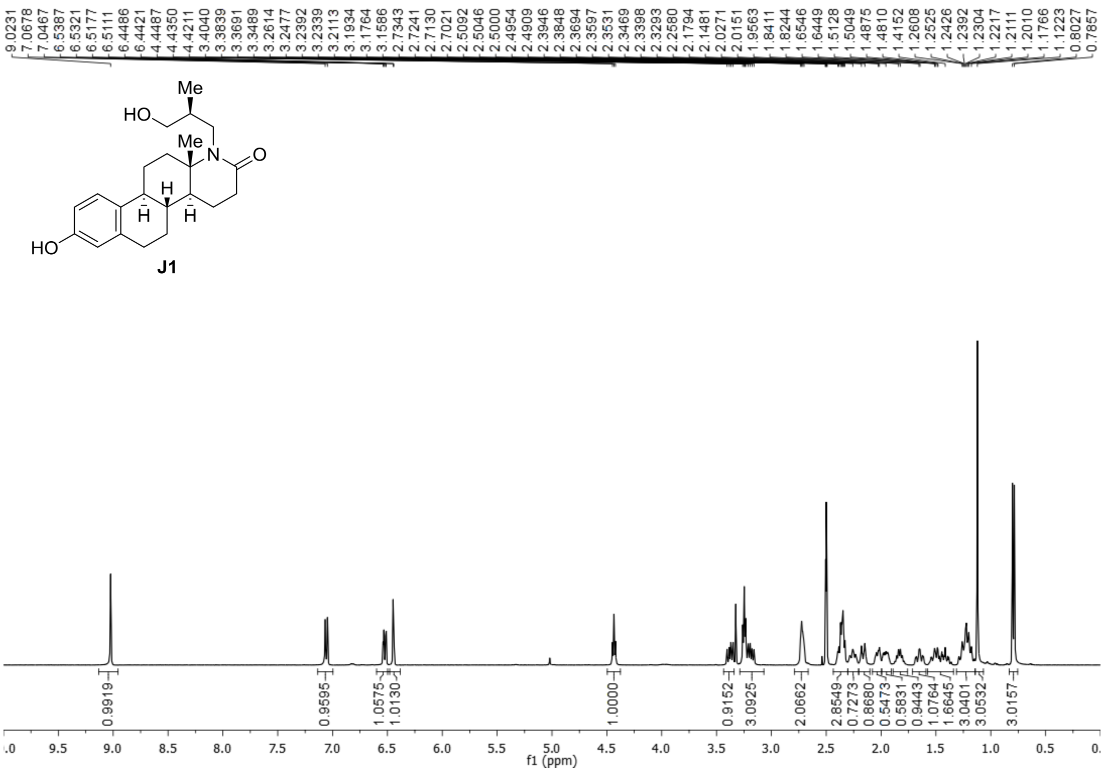

Supplementary Figure 370. ^{13}C NMR spectrum in DMSO-d_6, 126 MHz, of **J1**.

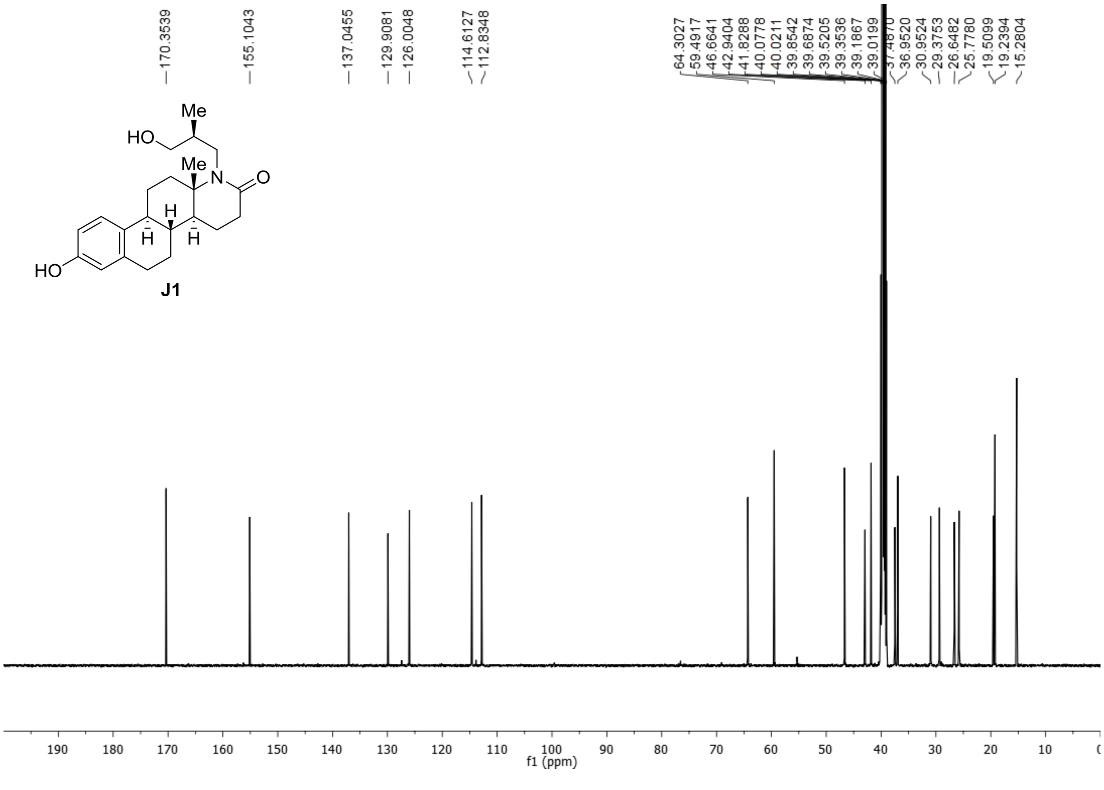

Supplementary Figure 371. ^{1}H NMR spectrum in DMSO-d_6, 400 MHz, of **I3**.

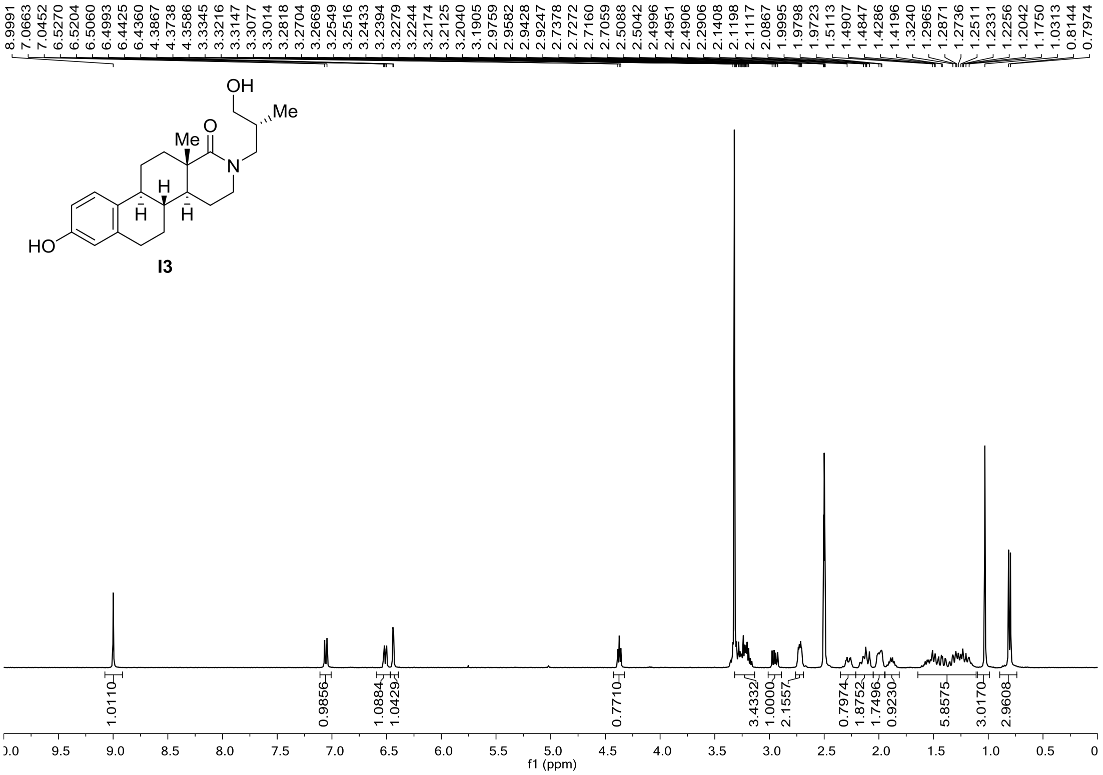

Supplementary Figure 372. ^{13}C NMR spectrum in DMSO-d_6, 151 MHz, of **I3**.

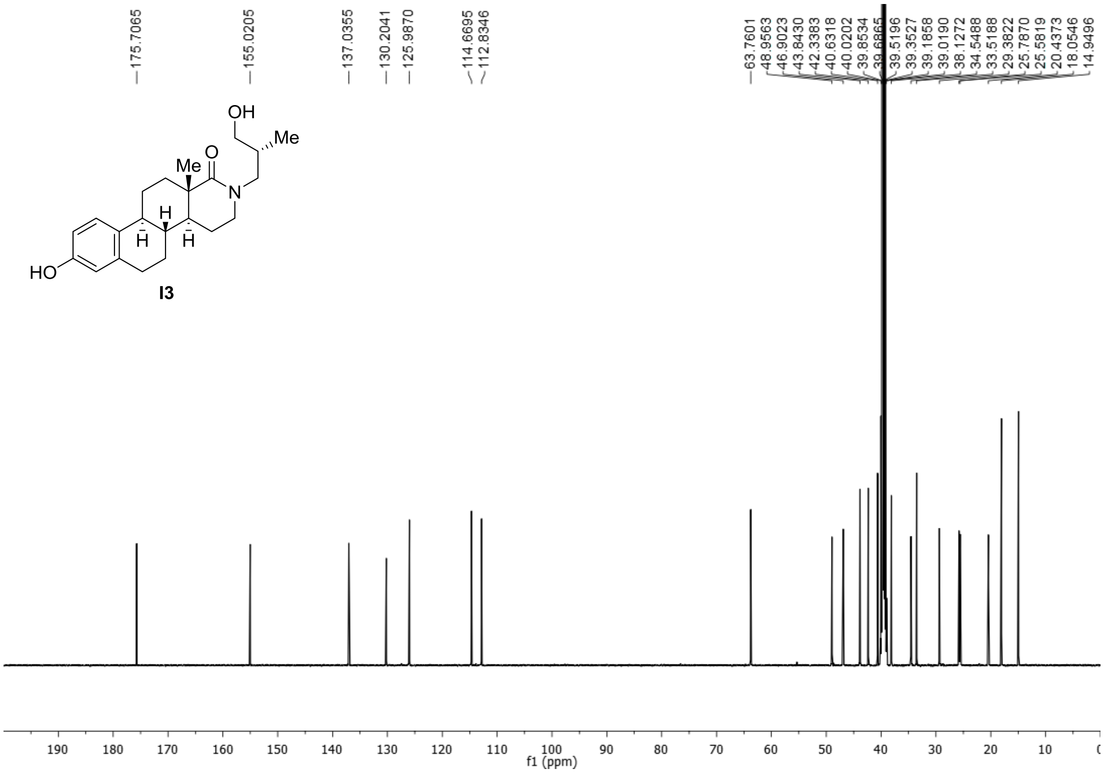

Supplementary Figure 373. ^{1}H NMR spectrum in DMSO-d_6, 600 MHz, of **I4**.

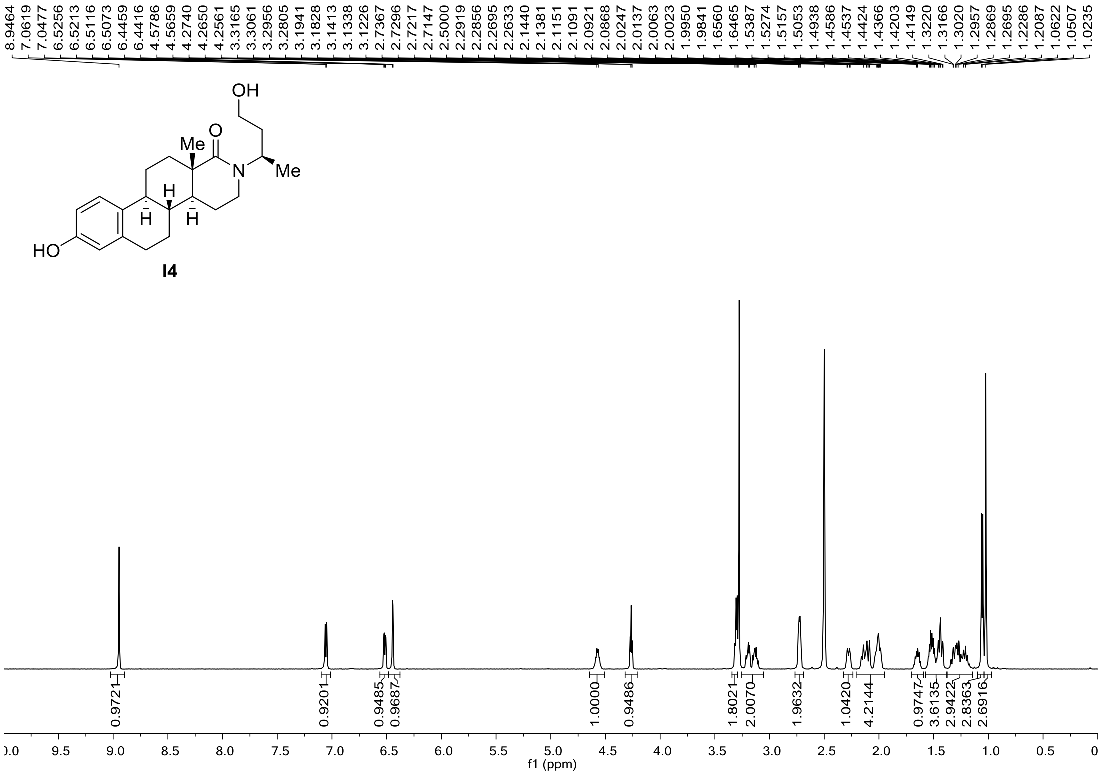

Supplementary Figure 374. ^{13}C NMR spectrum in DMSO-d_6, 151 MHz, of **I4**.

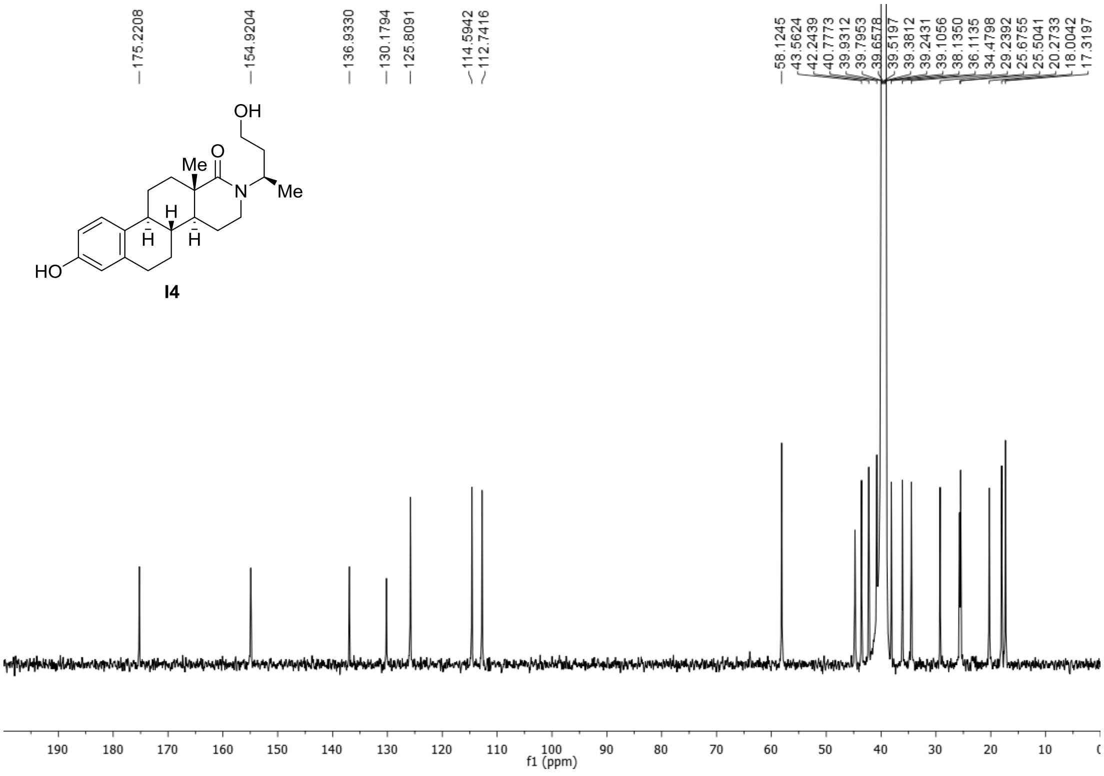

Supplementary Figure 375. ^{13}C NMR spectrum in DMSO-d_6, 151 MHz, of **I4**.

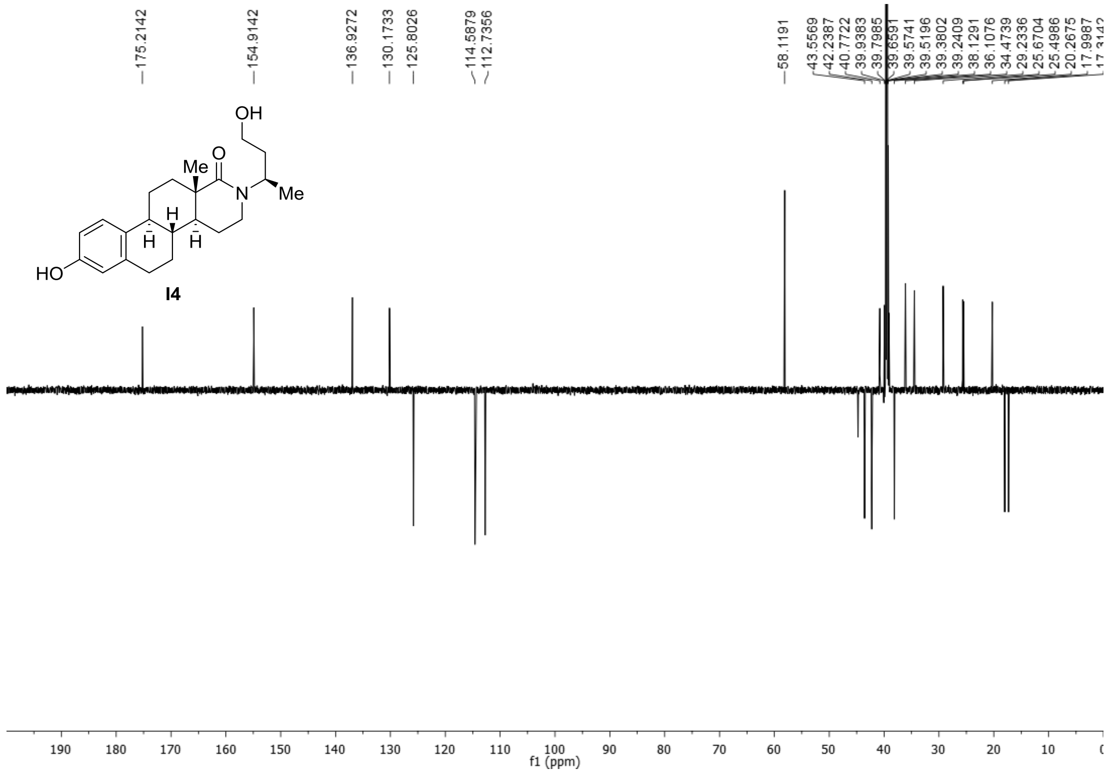

Supplementary Figure 376. ^{1}H NMR spectrum in $CDCl_3$, 400 MHz, of **I5**.

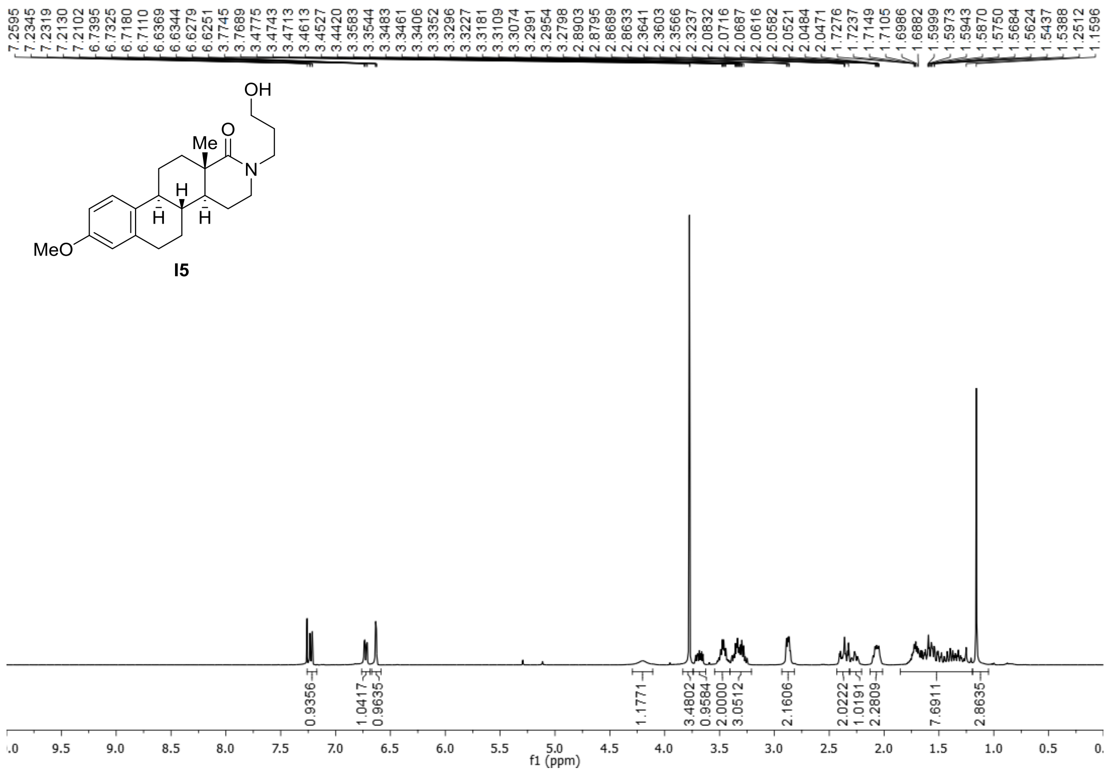

Supplementary Figure 377. ^{13}C NMR spectrum in $CDCl_3$, 101 MHz, of **I5**.

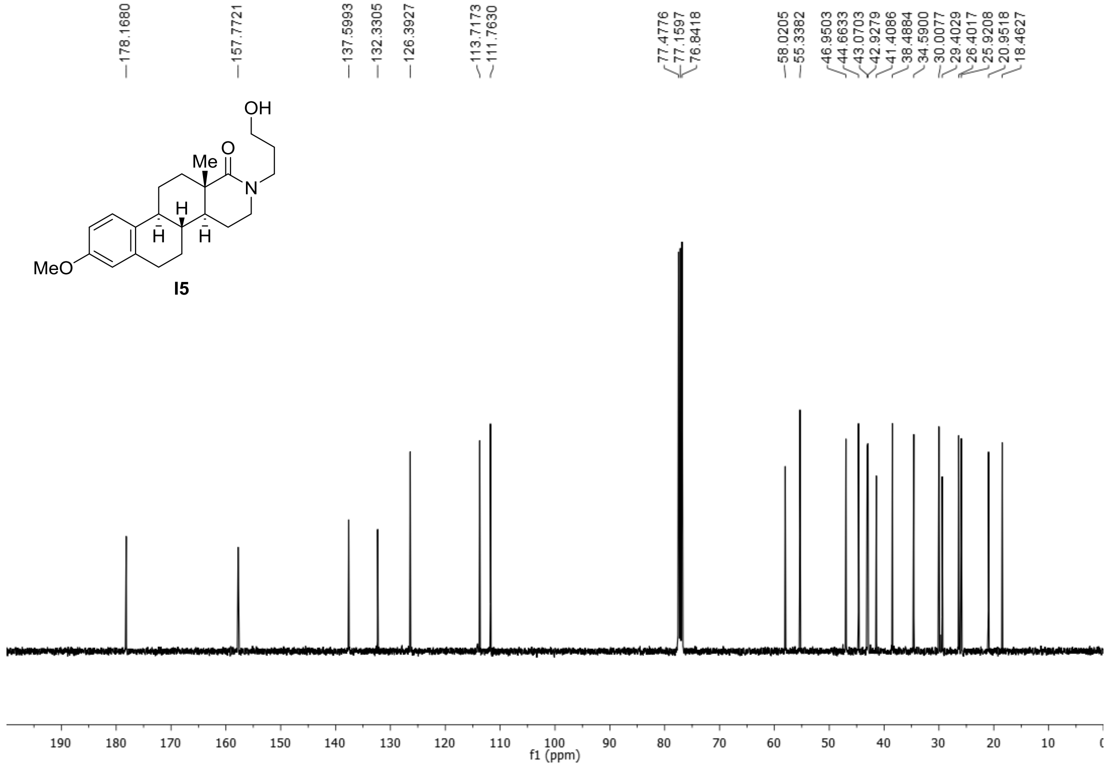

Supplementary Figure 378. ^{1}H NMR spectrum in DMSO-d_6, 400 MHz, of **I7**.

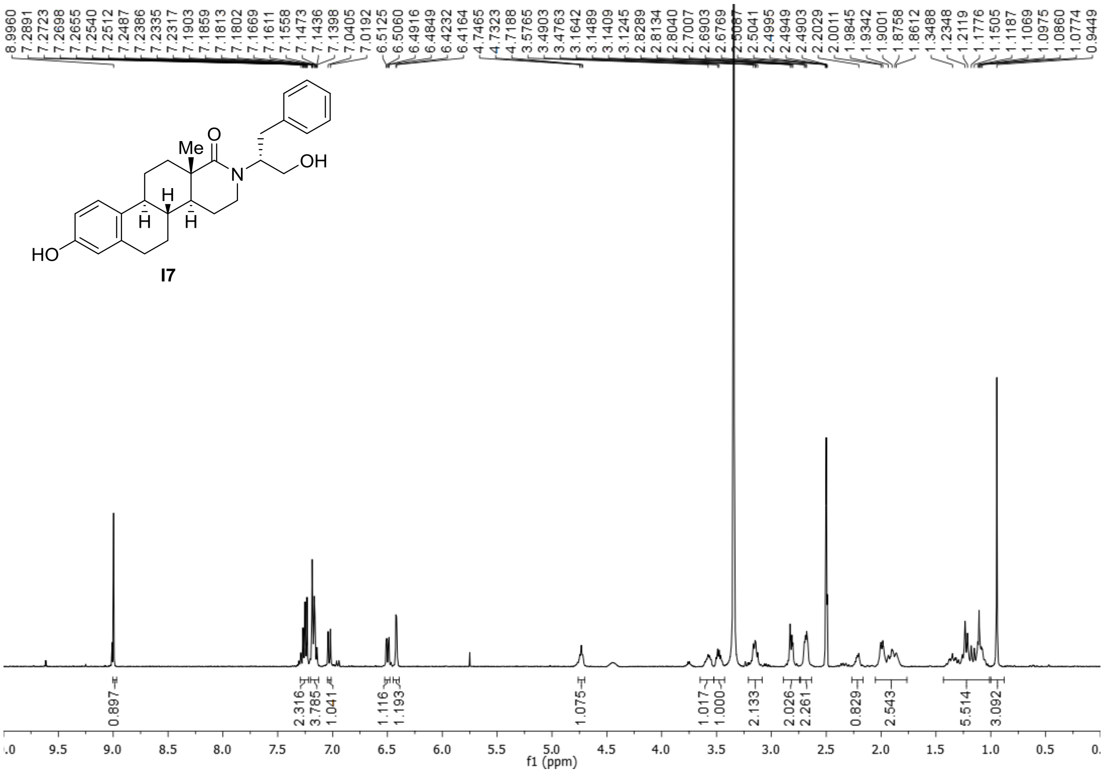

Supplementary Figure 379. ^{13}C NMR (APT) spectrum in DMSO-d_6, 151 MHz, of **I7**.

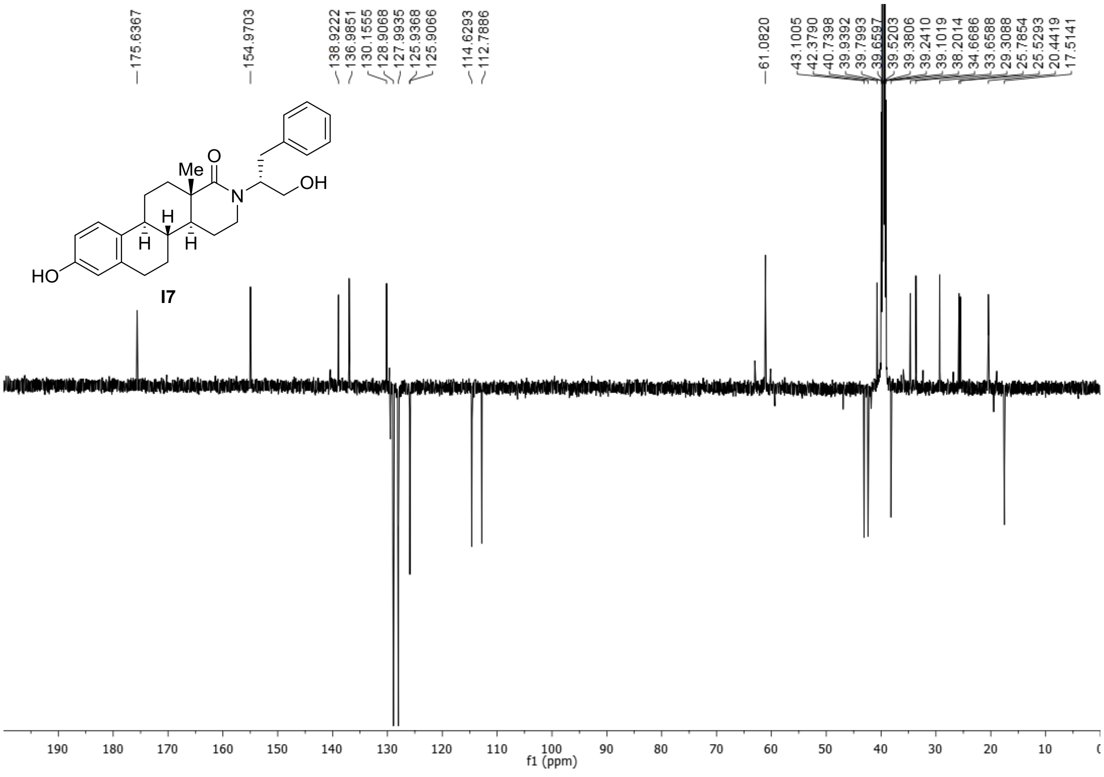

Supplementary Figure 380. ^{1}H NMR spectrum in DMSO-d_6, 400 MHz, of **I8**.

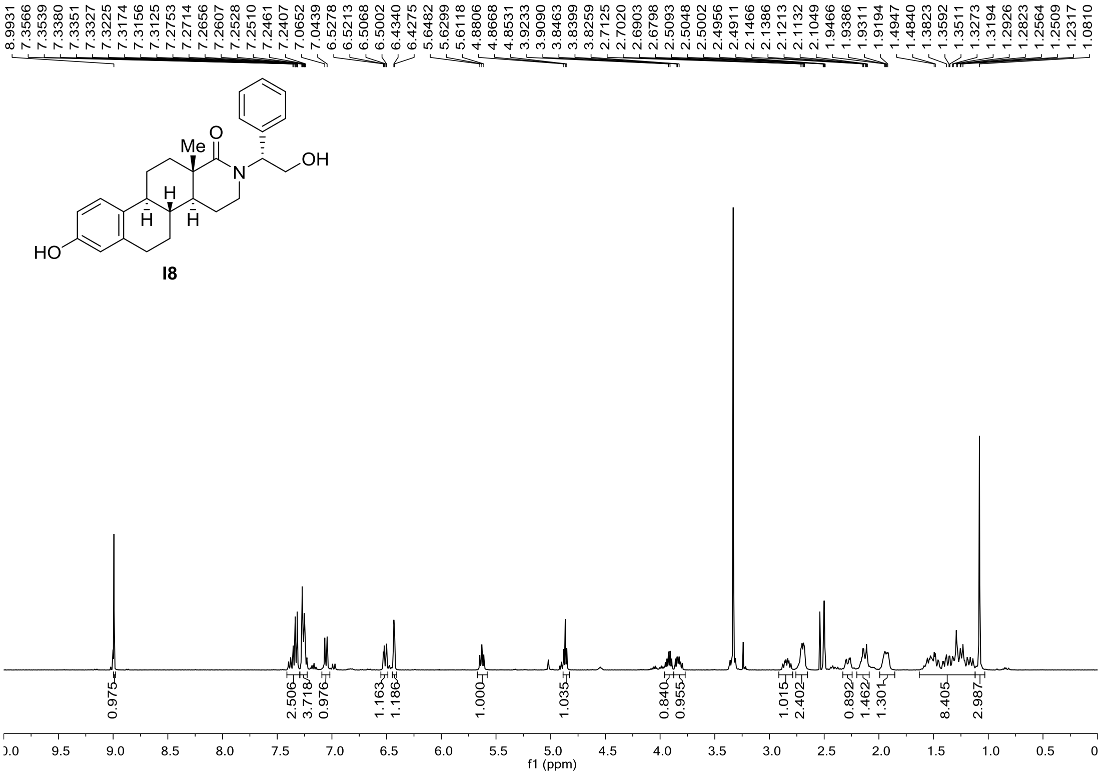

Supplementary Figure 381. ^{13}C NMR spectrum in DMSO-d_6, 101 MHz, of **I8**.

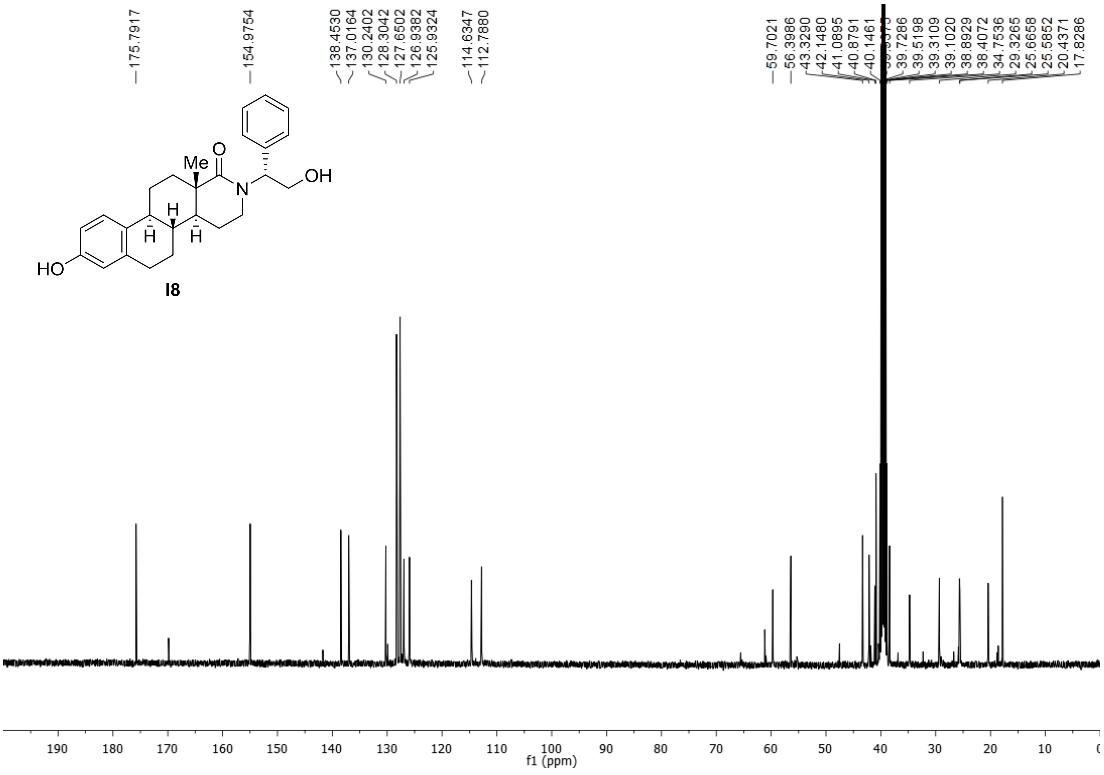

Supplementary Figure 382. ^{1}H NMR spectrum in DMSO-d_6, 400 MHz, of **I9**.

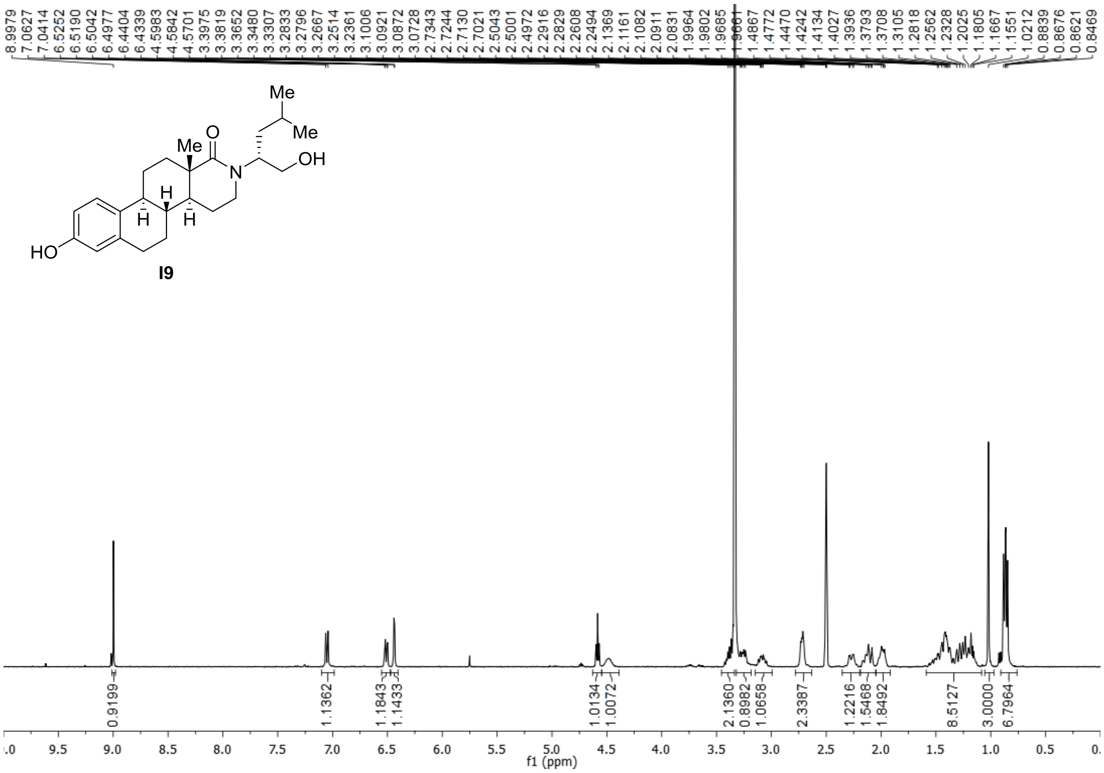

Supplementary Figure 383. ^{13}C NMR spectrum in DMSO-d_6, 151 MHz, of **I9**.

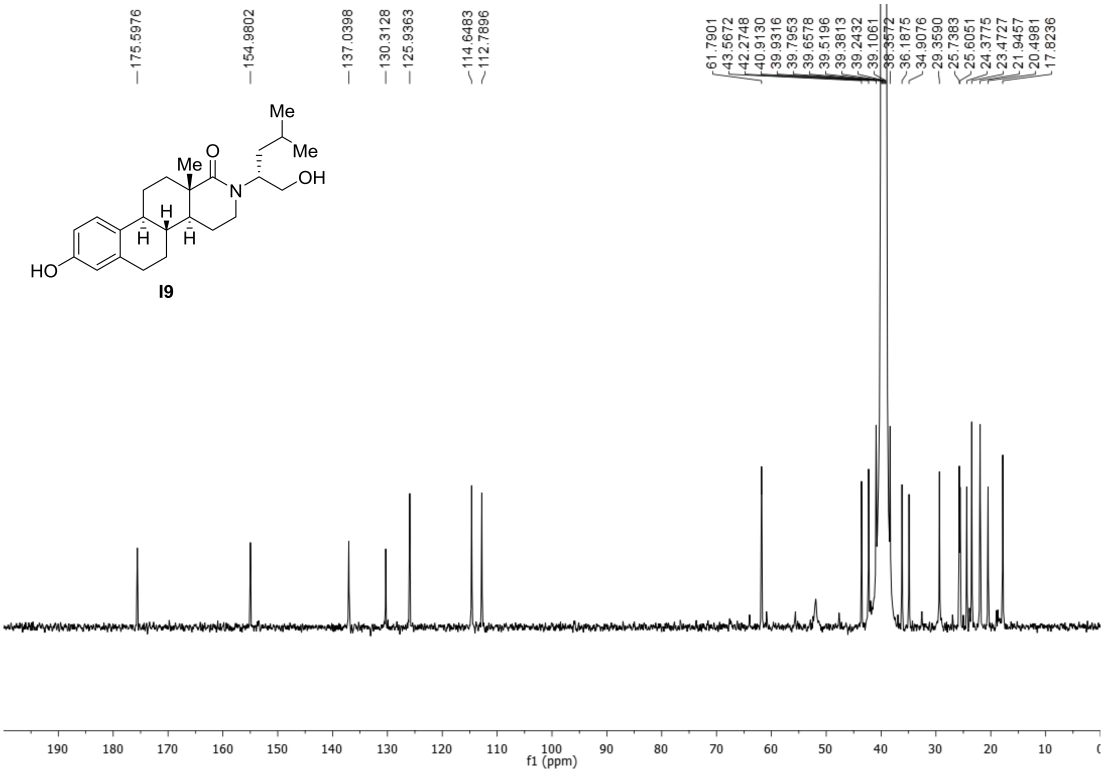

Supplementary Figure 384. ^{1}H NMR spectrum in DMSO-d_6, 400 MHz, of **I10**.

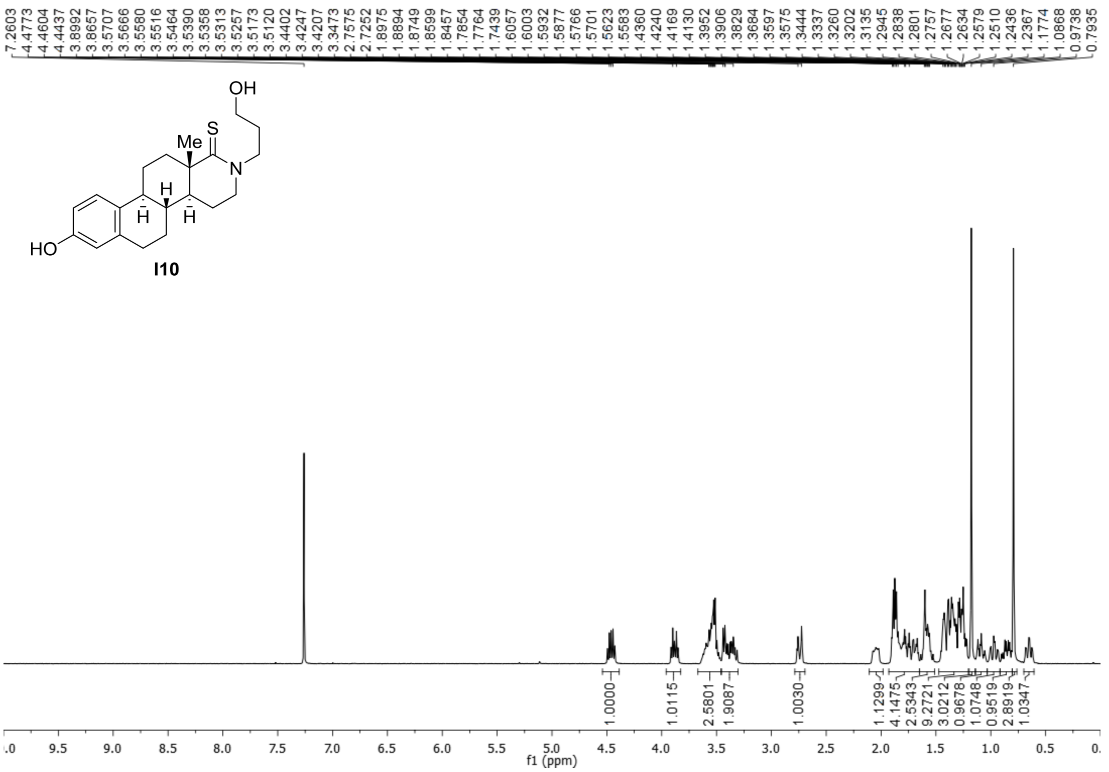

Supplementary Figure 385. ^{13}C NMR spectrum in DMSO-d_6, 126 MHz, of **I10**.

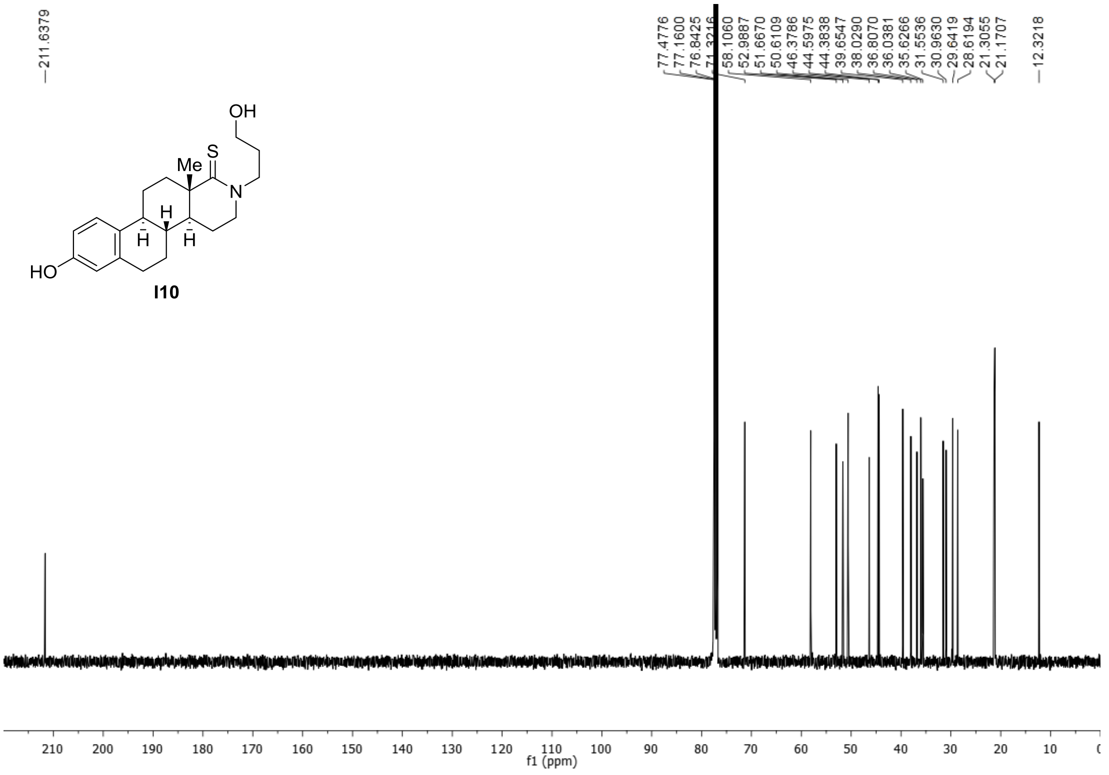

Supplementary Figure 386. ^{1}H NMR spectrum in DMSO-d_6, 400 MHz, of **I11**.

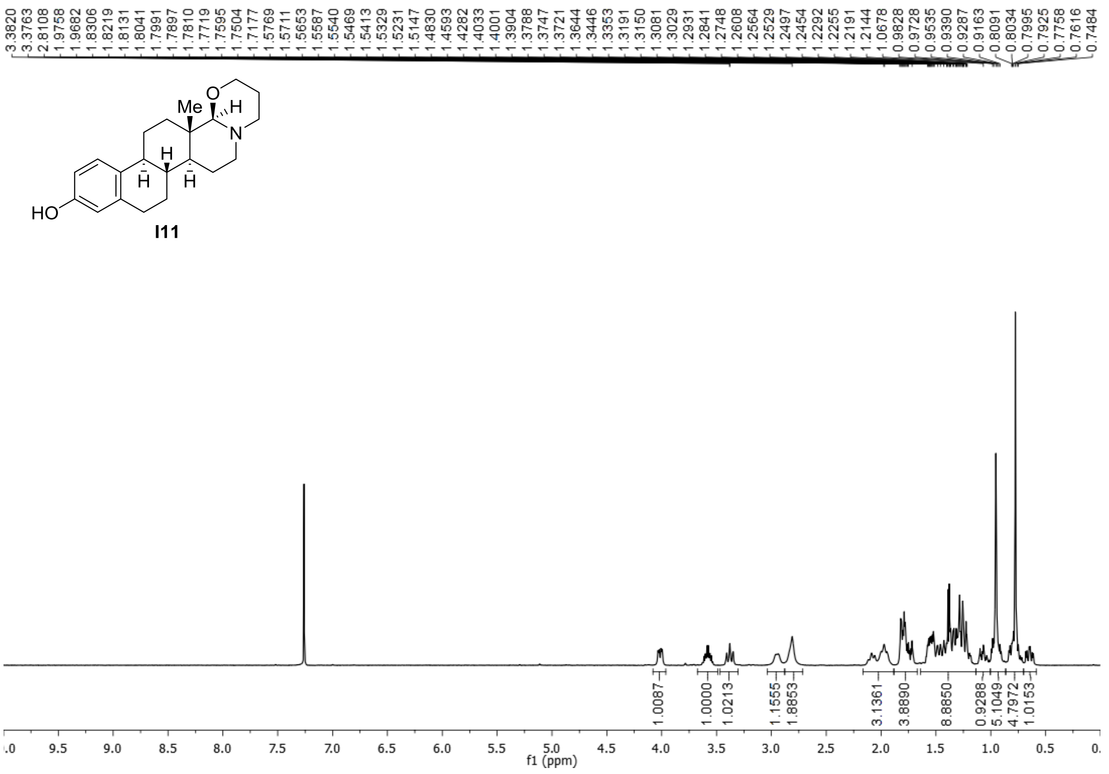

Supplementary Figure 387. ^{13}C NMR (APT) spectrum in DMSO-d_6, 126 MHz, of **I11**.

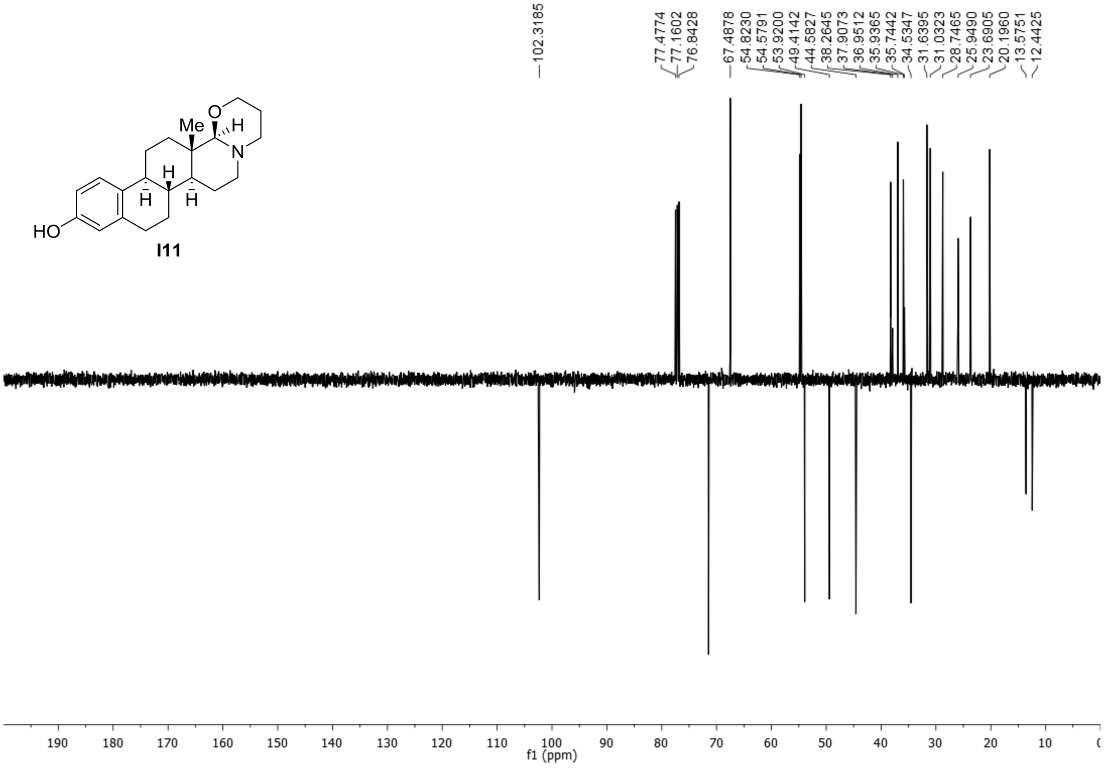

Supplementary Figure 388. ^{1}H NMR spectrum in DMSO-d_6, 400 MHz, of **I12**.

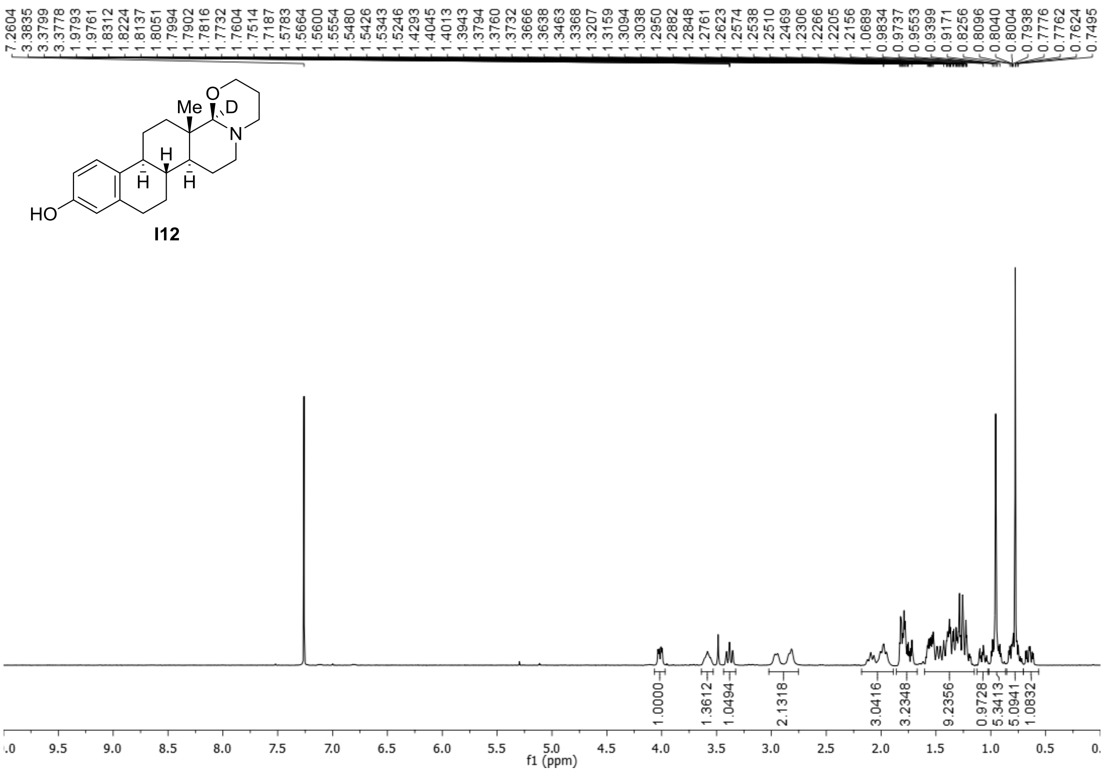

Supplementary Figure 389. ^{13}C NMR spectrum in DMSO-d_6, 126 MHz, of **I12**.

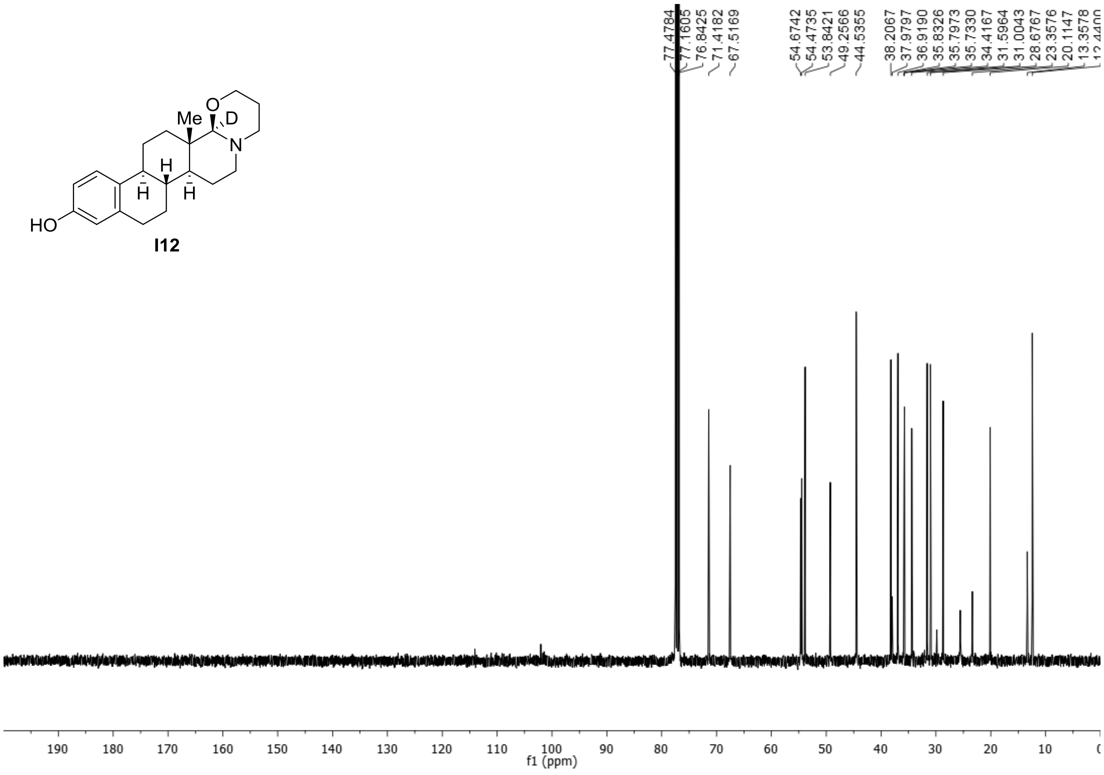

Supplementary Figure 390. ^{1}H NMR spectrum in DMSO-d_6, 400 MHz, of **I13**.

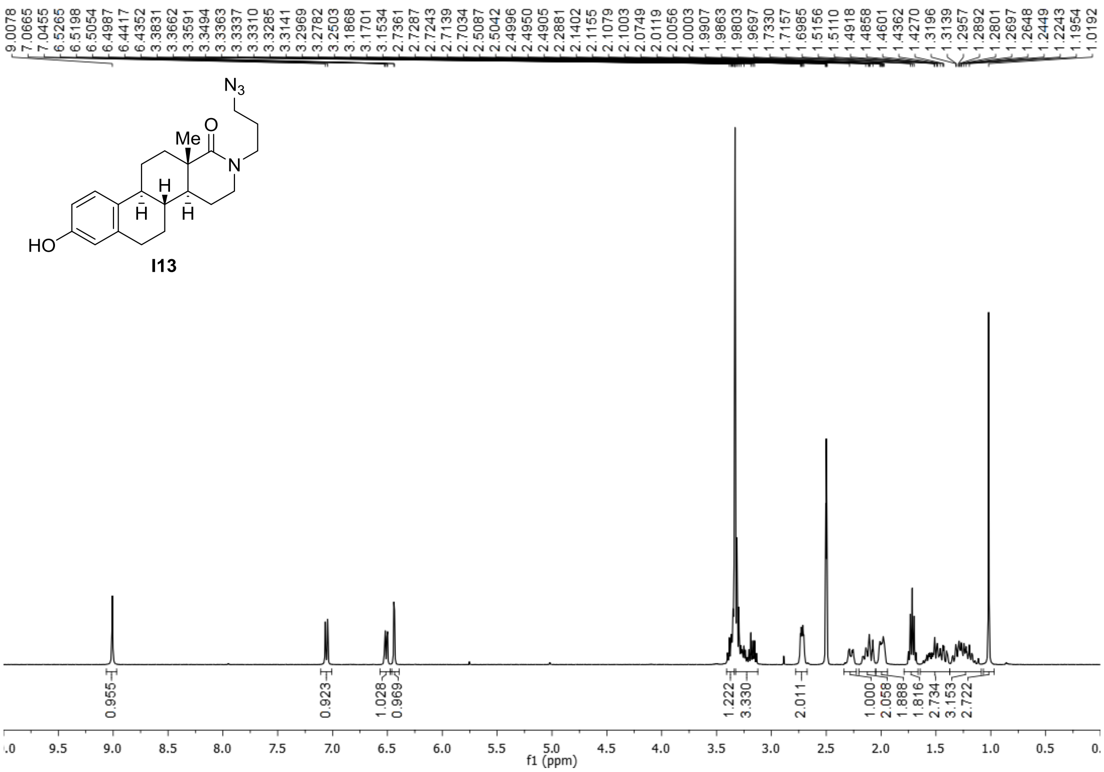

Supplementary Figure 391. ^{13}C NMR spectrum in DMSO-d_6, 101 MHz, of **I13**.

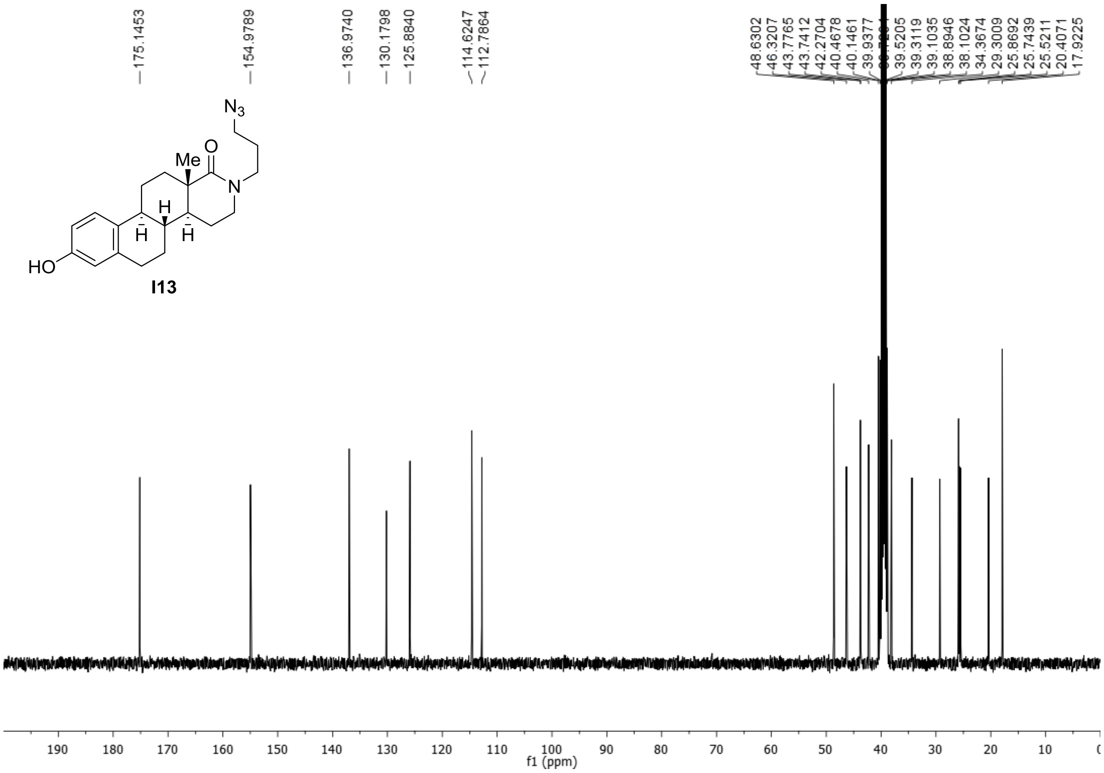

Supplementary Figure 392. ^{1}H NMR spectrum in DMSO-d_6, 400 MHz, of **I14**.

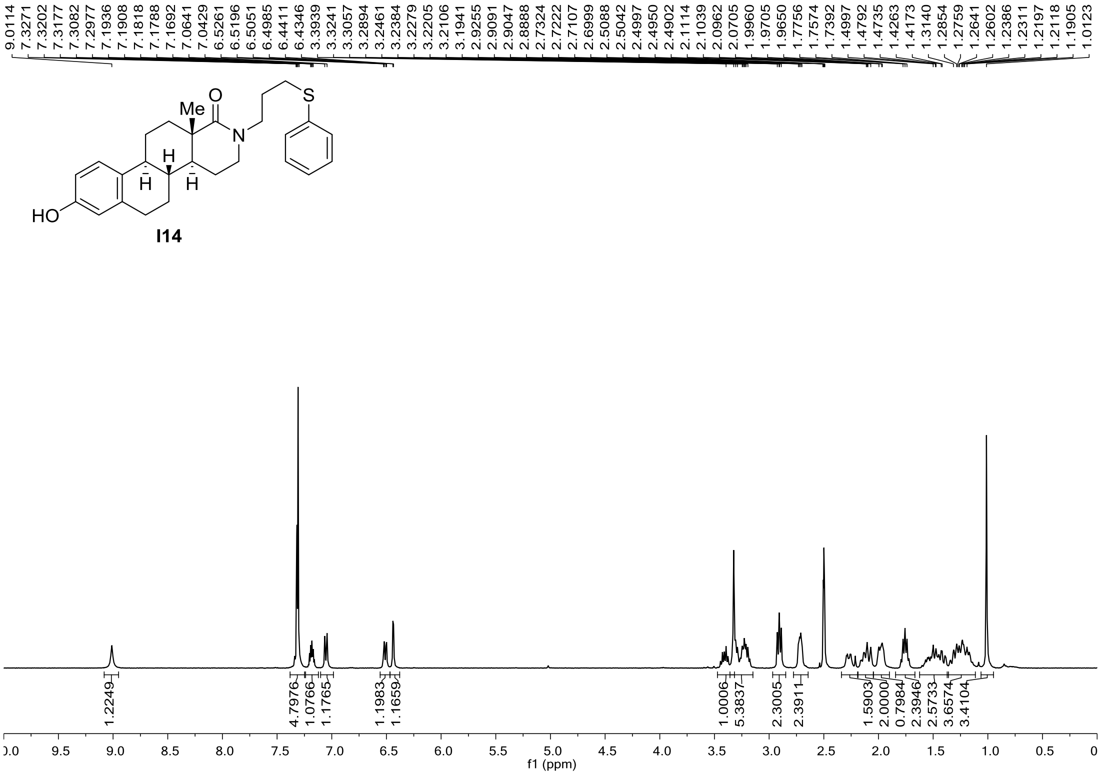

Supplementary Figure 393. ^{13}C NMR spectrum in DMSO-d_6, 126 MHz, of **I14**.

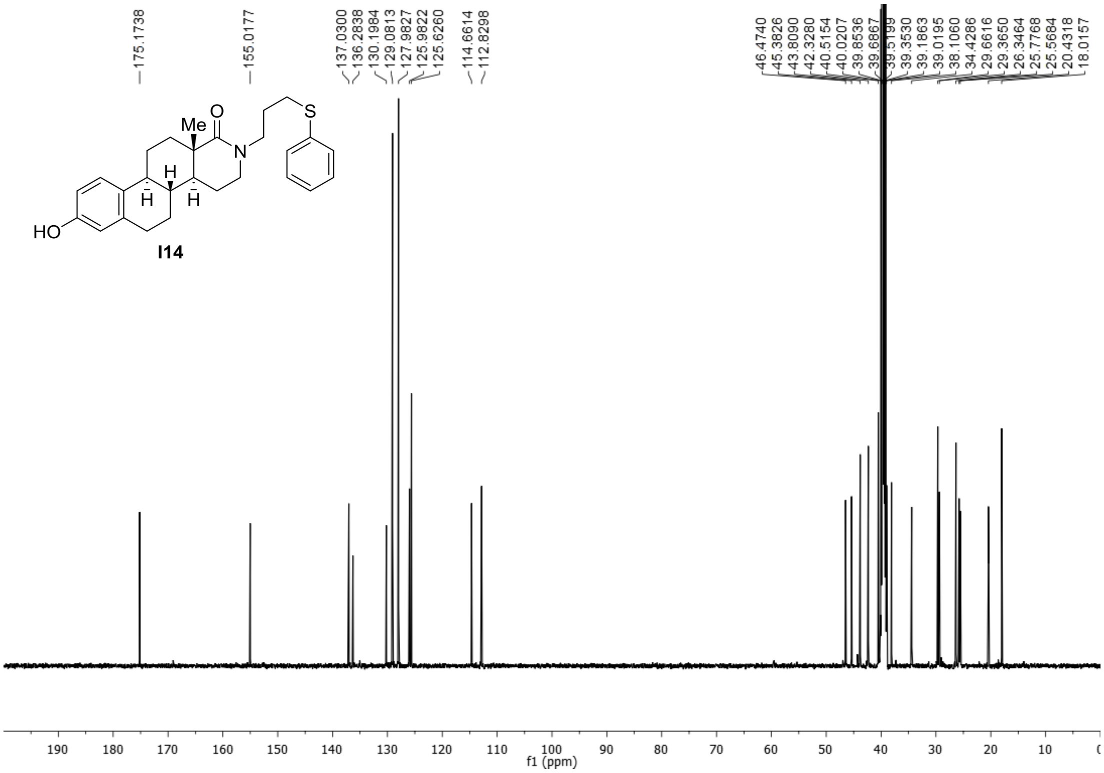

Supplementary Figure 394. ^{1}H NMR spectrum in DMSO-d_6, 400 MHz, of **I15**.

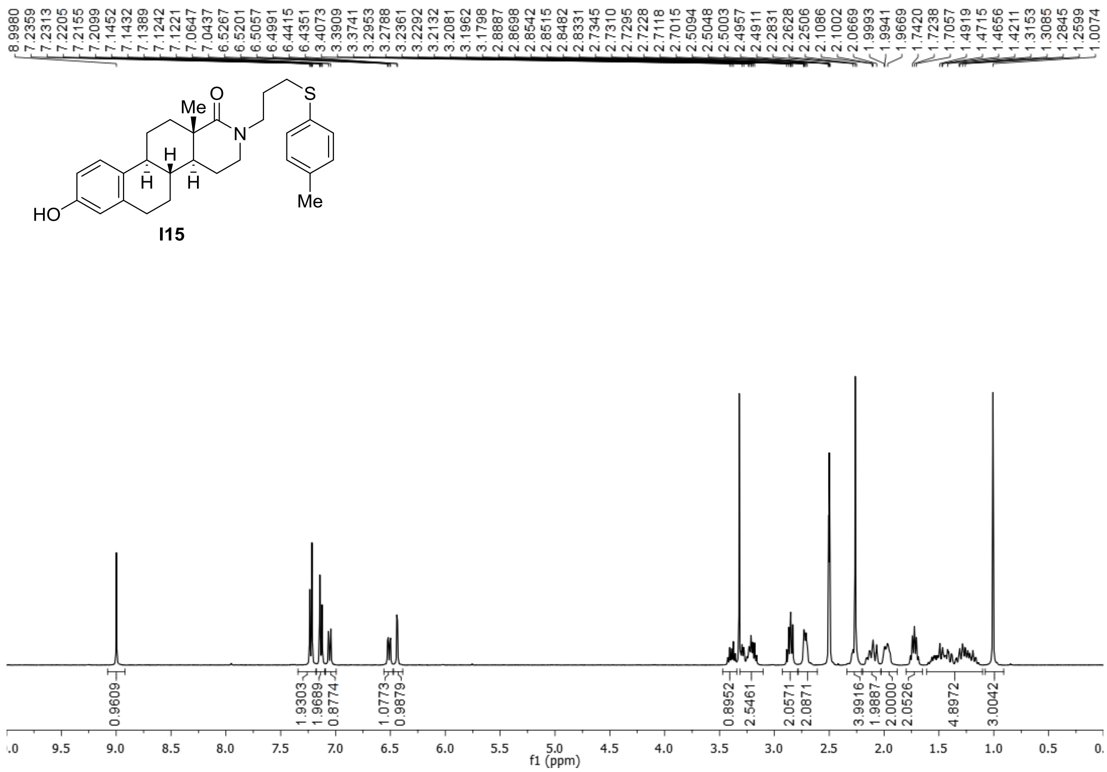

Supplementary Figure 395. ^{13}C NMR spectrum in DMSO-d_6, 126 MHz, of **I15**.

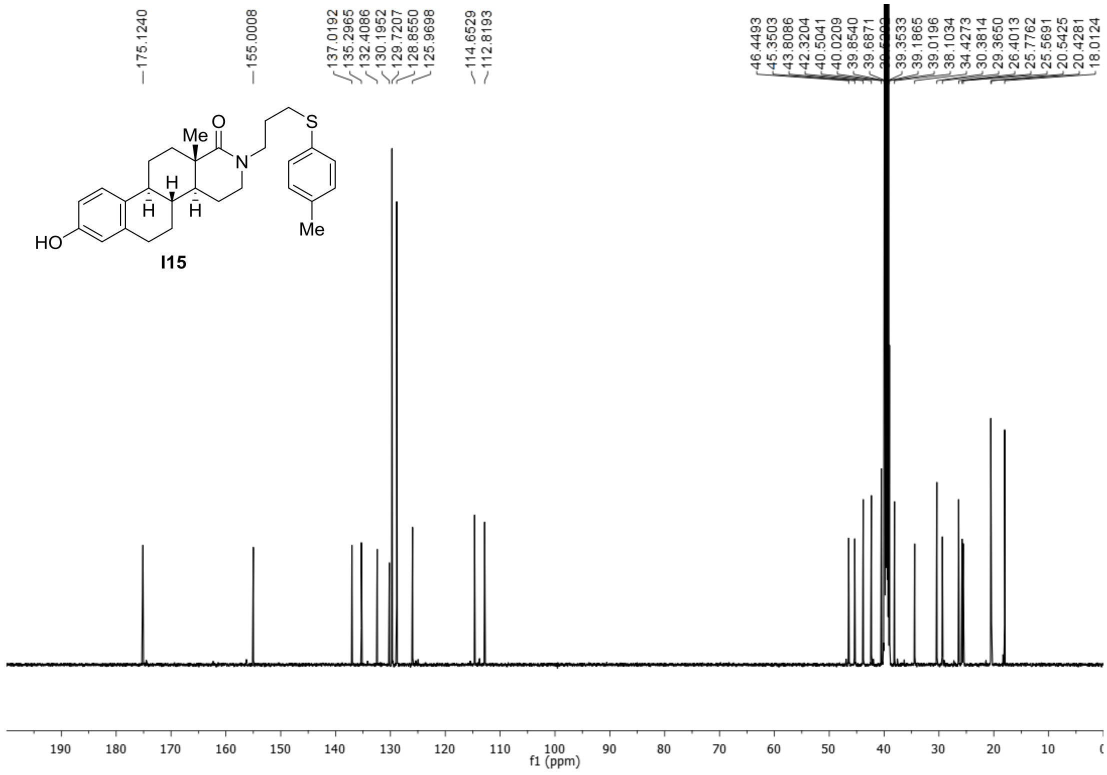

Supplementary Figure 396. ^{1}H NMR spectrum in DMSO-d_6, 400 MHz, of **I16**.

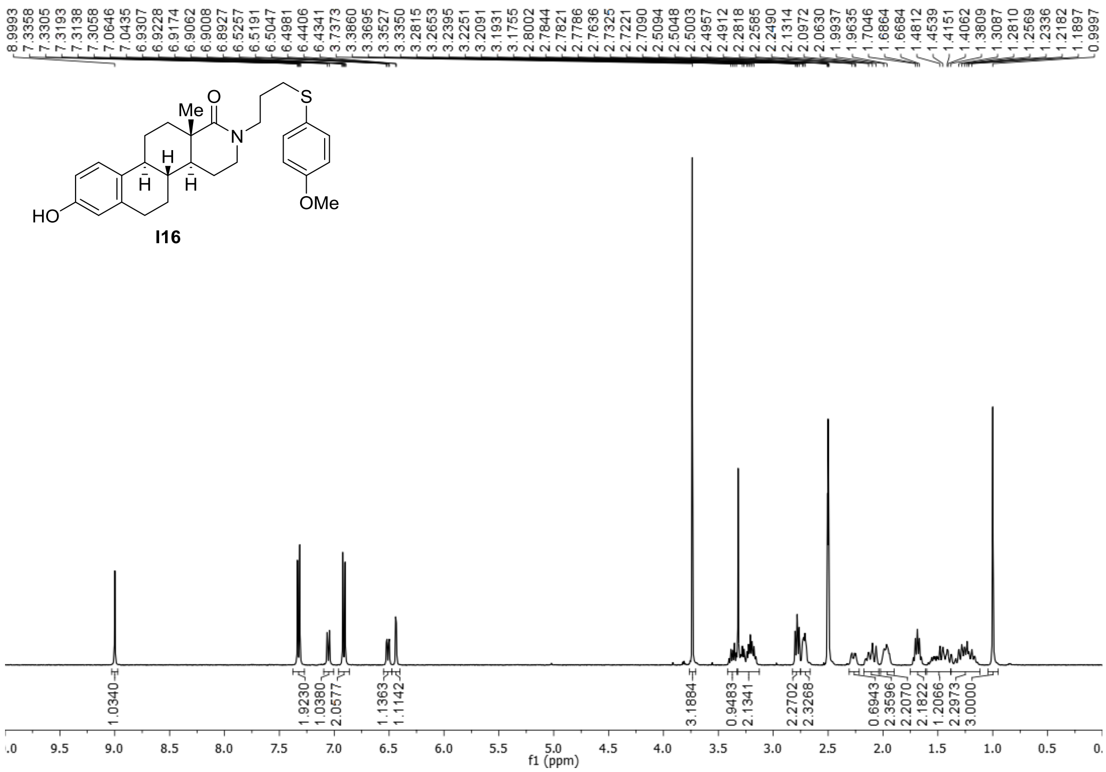

Supplementary Figure 397. ^{13}C NMR spectrum in DMSO-d_6, 126 MHz, of **I16**.

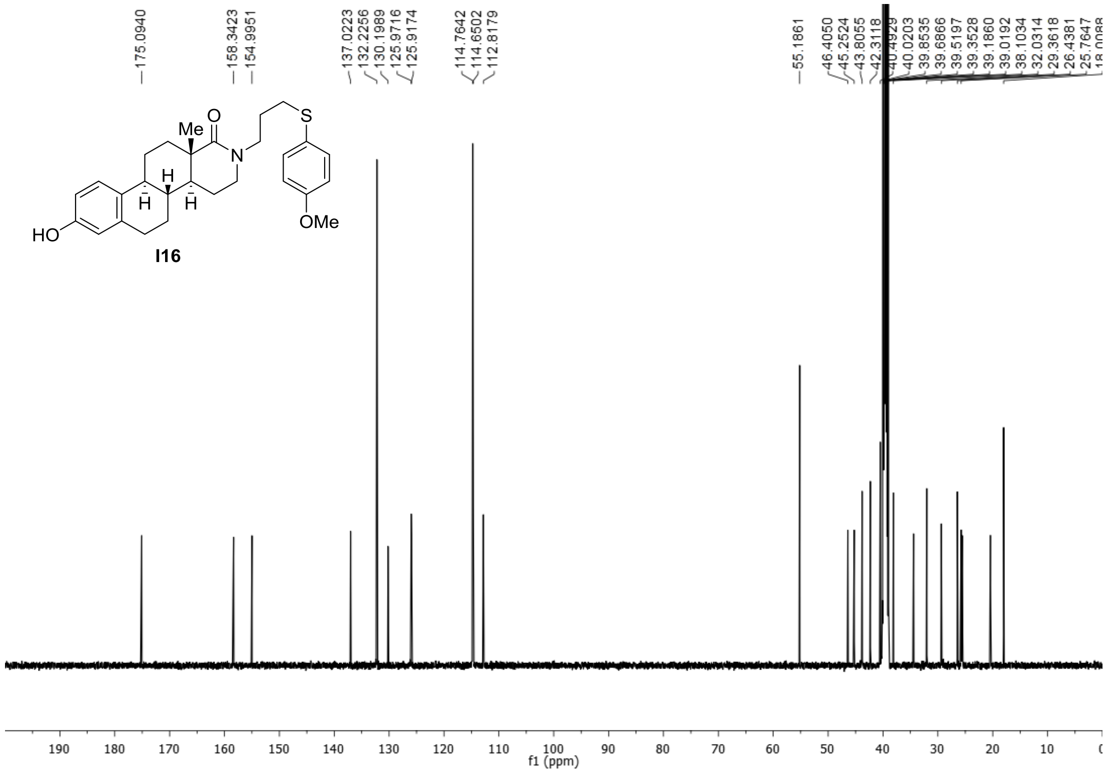

Supplementary Figure 398. ^{1}H NMR spectrum in DMSO-d_6, 400 MHz, of **I17**.

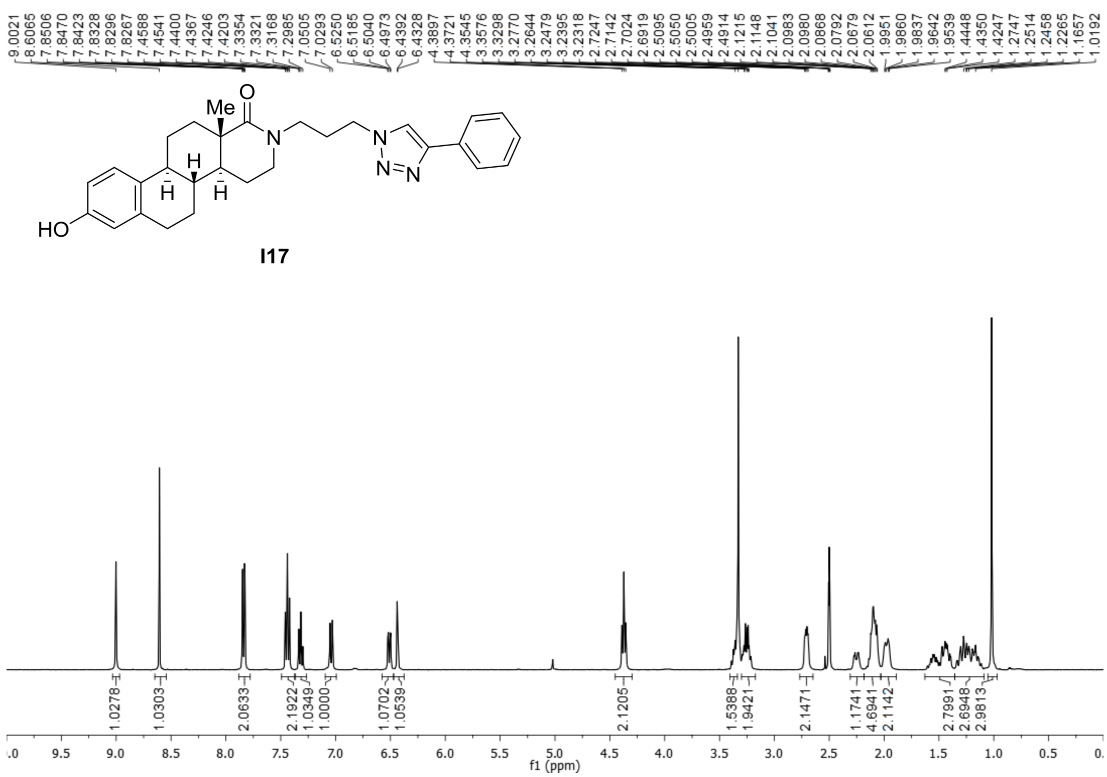

Supplementary Figure 399. ^{13}C NMR spectrum in DMSO-d_6, 126 MHz, of **I17**.

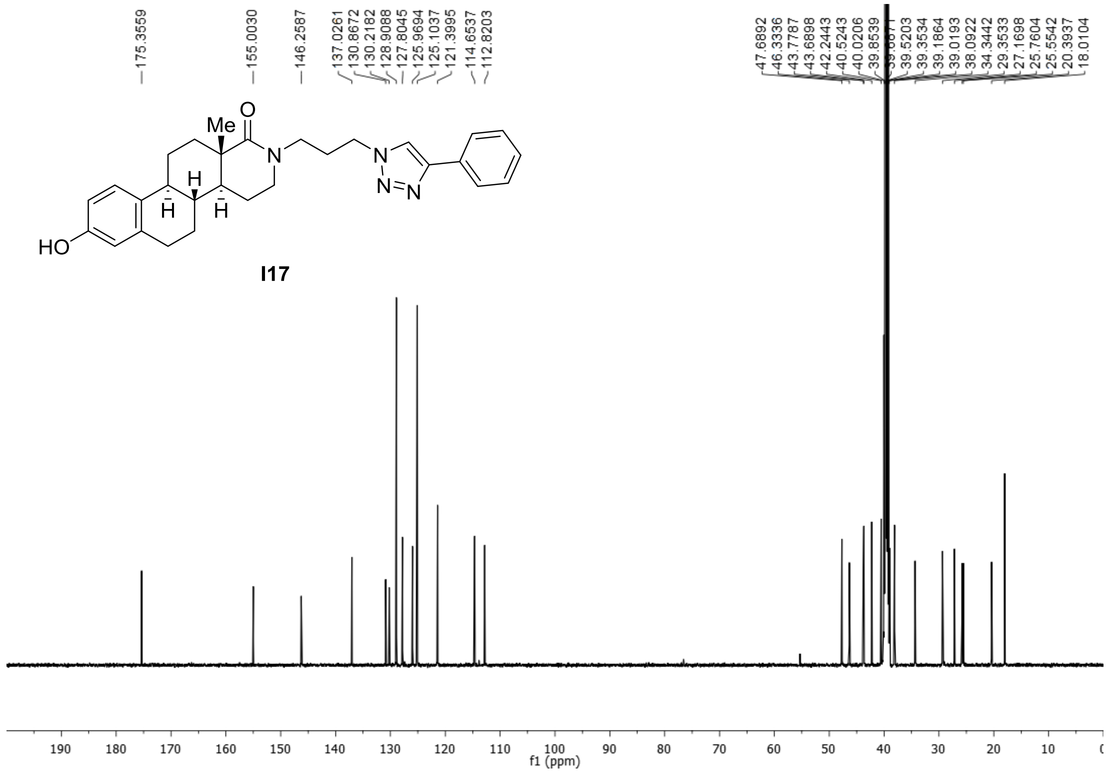

Supplementary Figure 400. ^{1}H NMR spectrum in DMSO-d_6, 600 MHz, of **J2**.

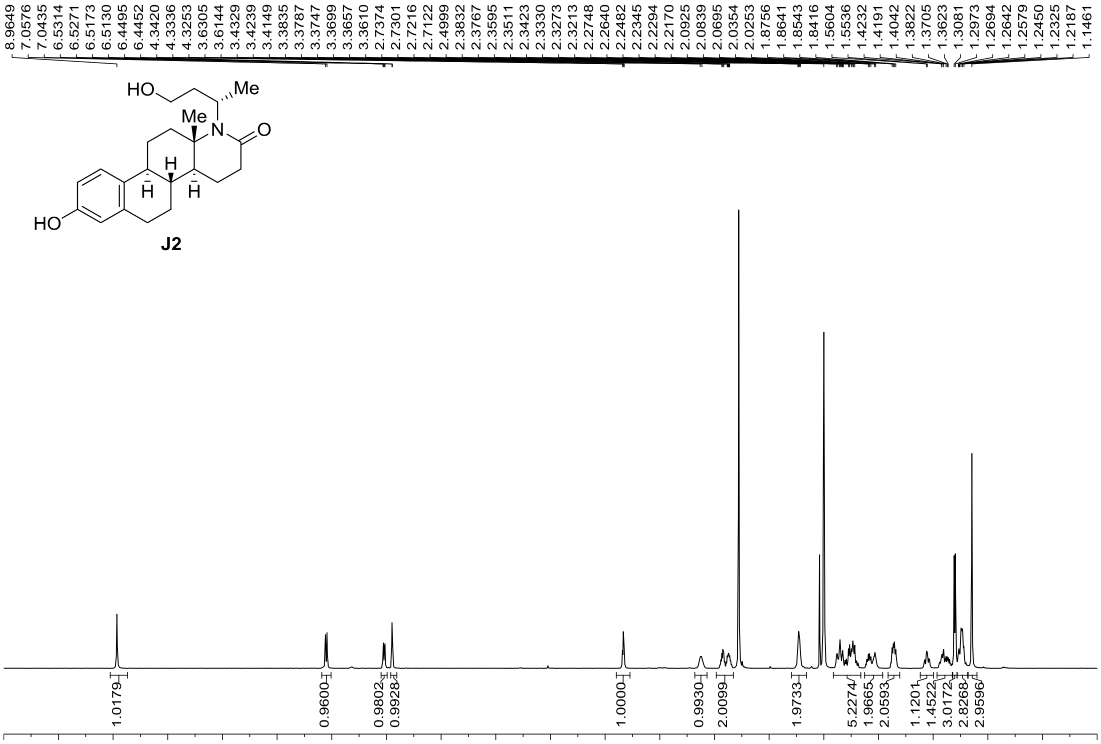

Supplementary Figure 401. ^{13}C NMR spectrum in DMSO-d_6, 151 MHz, of **J2**.

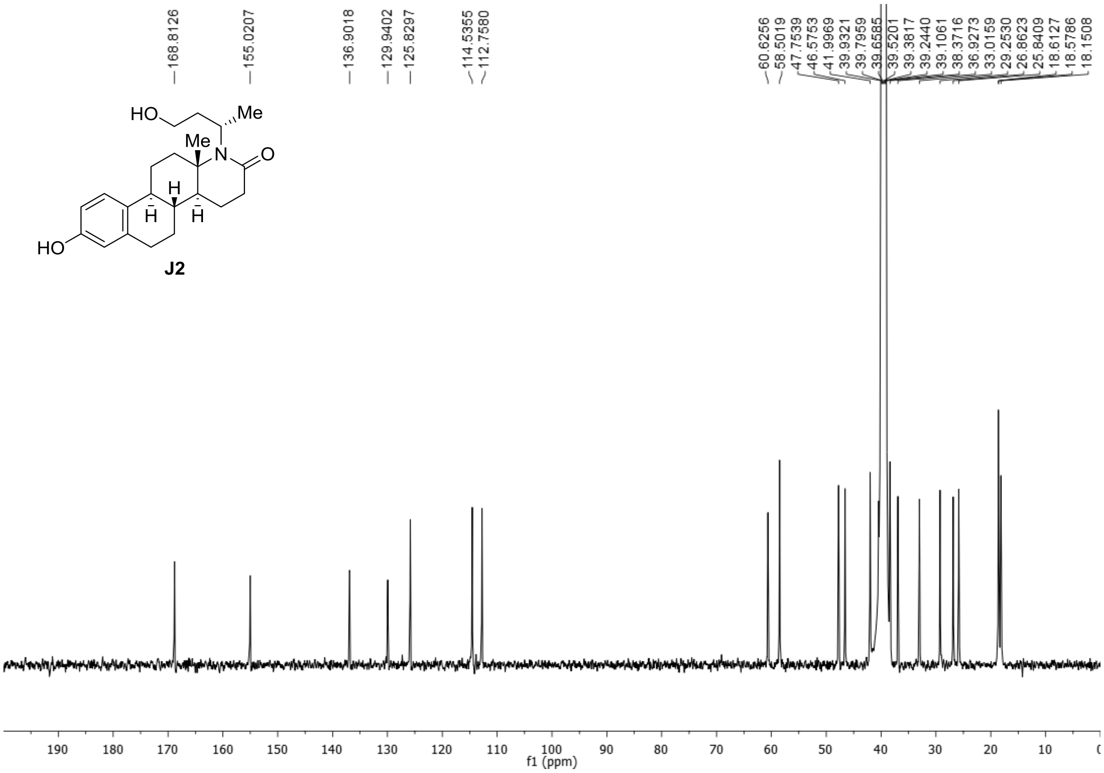

Supplementary Figure 402. ^{13}C NMR (APT) spectrum in DMSO-d_6, 151 MHz, of **J2**.

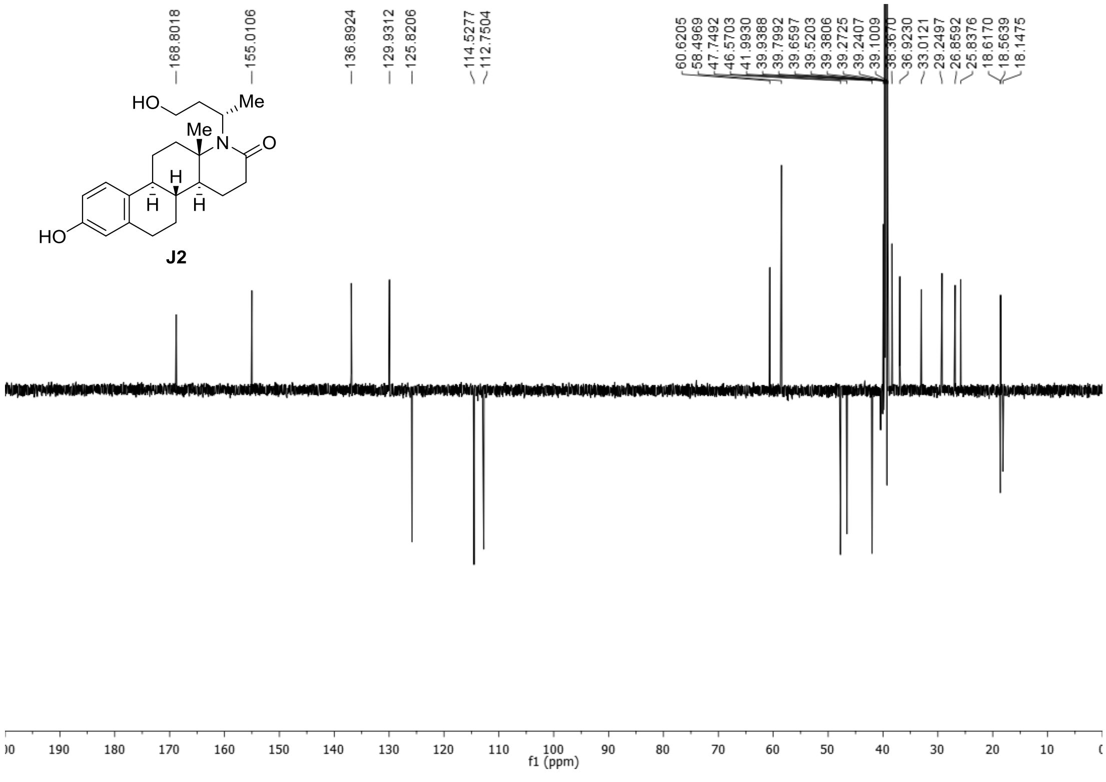

Supplementary Figure 403. ^{1}H NMR spectrum in DMSO-d_6, 600 MHz, of **I6**.

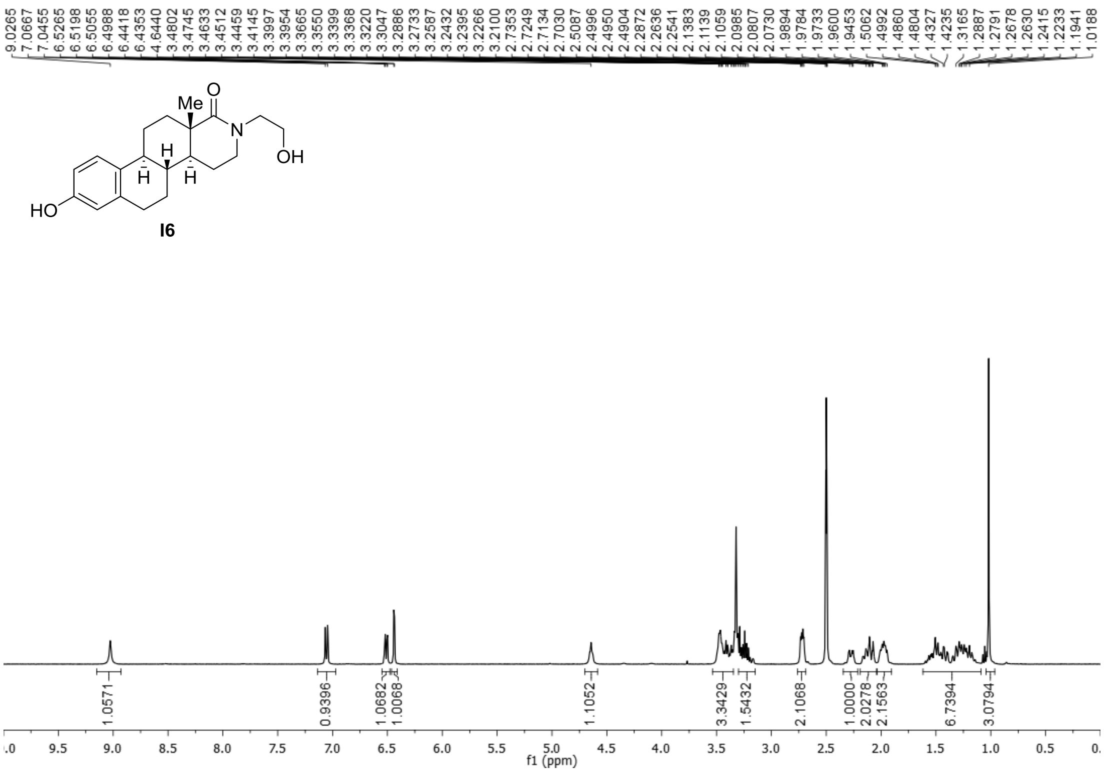

Supplementary Figure 404. ^{13}C NMR spectrum in DMSO-d_6, 126 MHz, of **I6**.

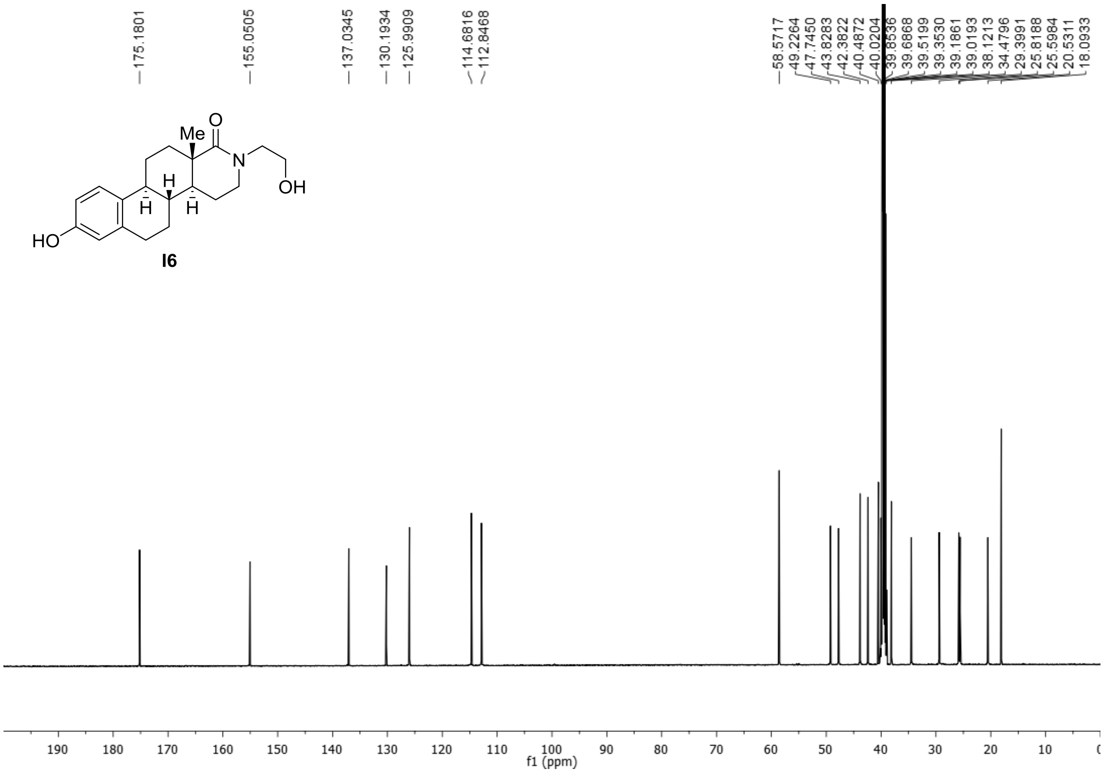

Supplementary Figure 405. ^{1}H NMR spectrum in DMSO-d_6, 400 MHz, of **J3**.

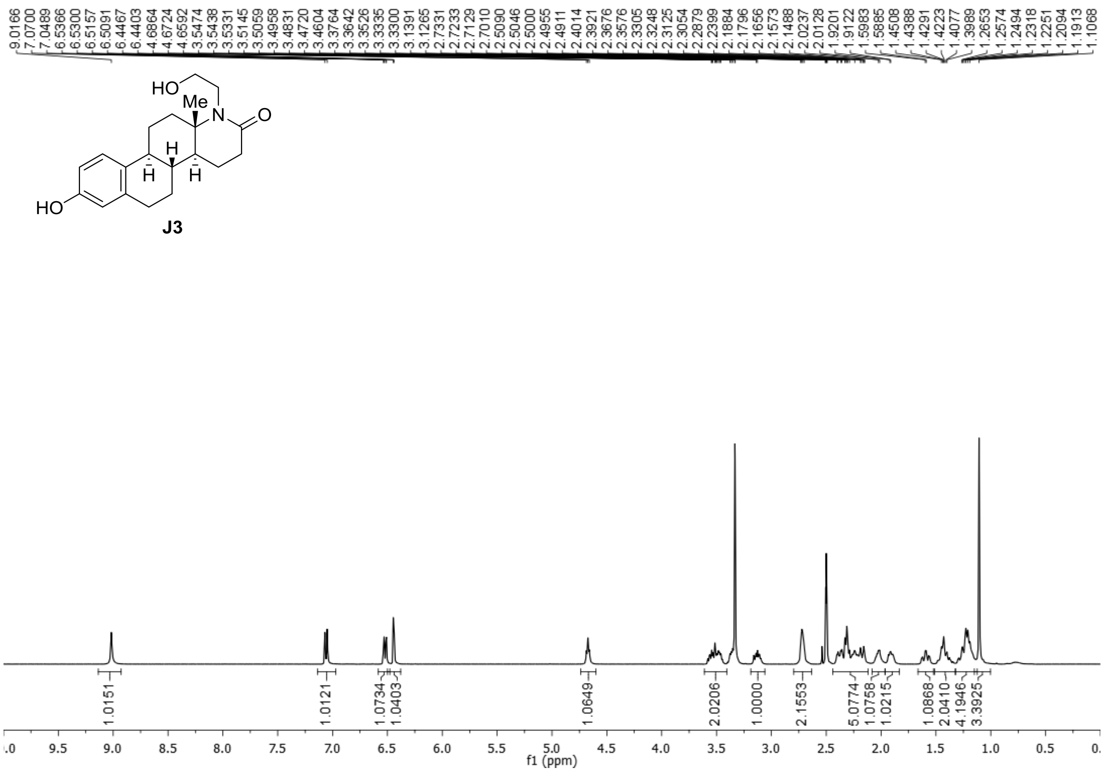

Supplementary Figure 406. ^{13}C NMR spectrum in DMSO-d_6, 126 MHz, of **J3**.

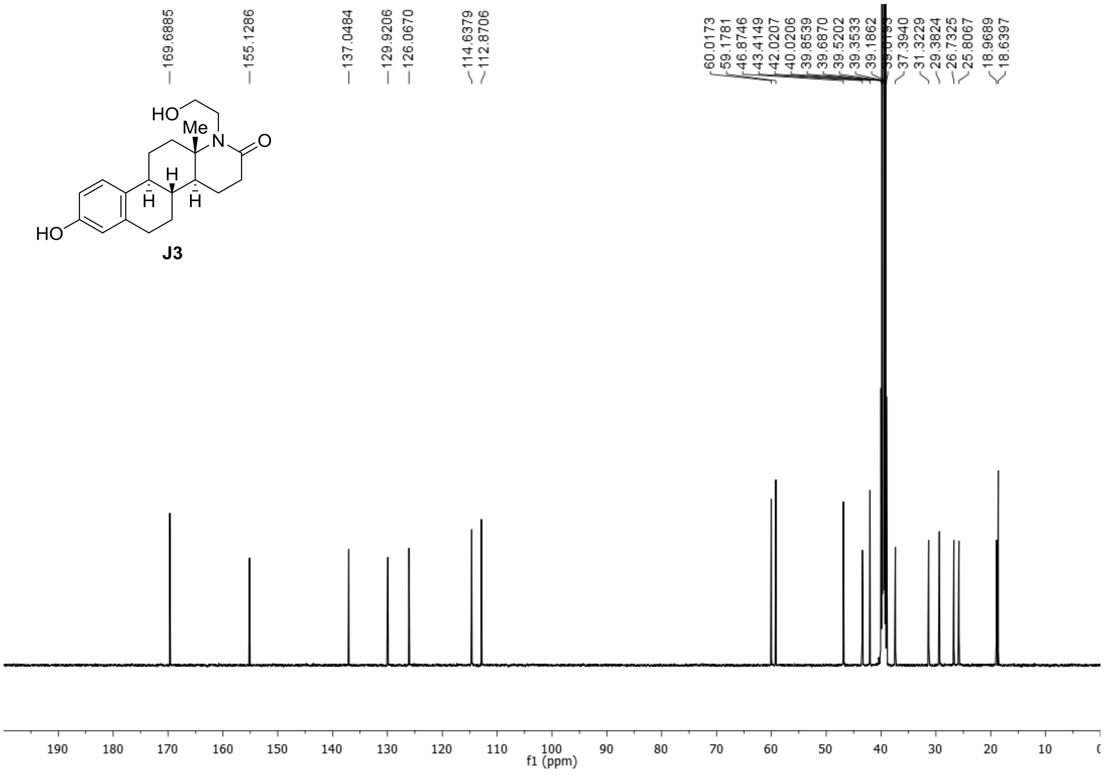

Supplementary Figure 407. ^{1}H NMR spectrum in DMSO-d_6, 400 MHz, of **J4**.

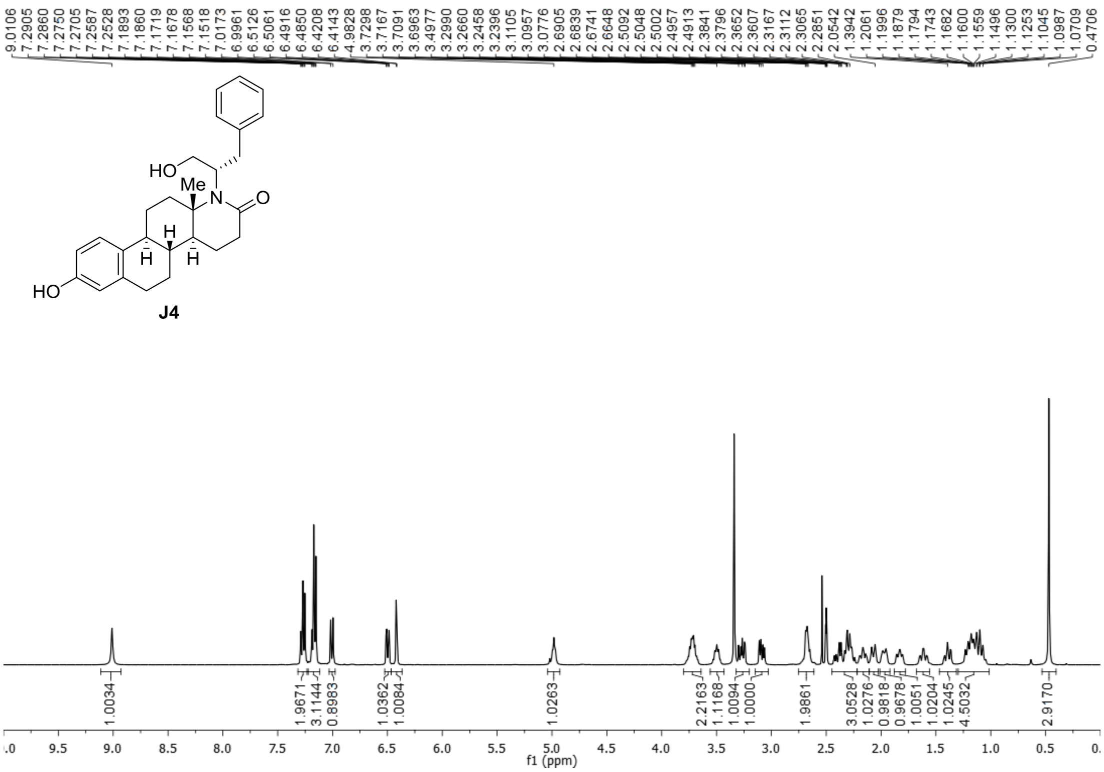

Supplementary Figure 408. ^{13}C NMR spectrum in DMSO-d_6, 126 MHz, of **J4**.

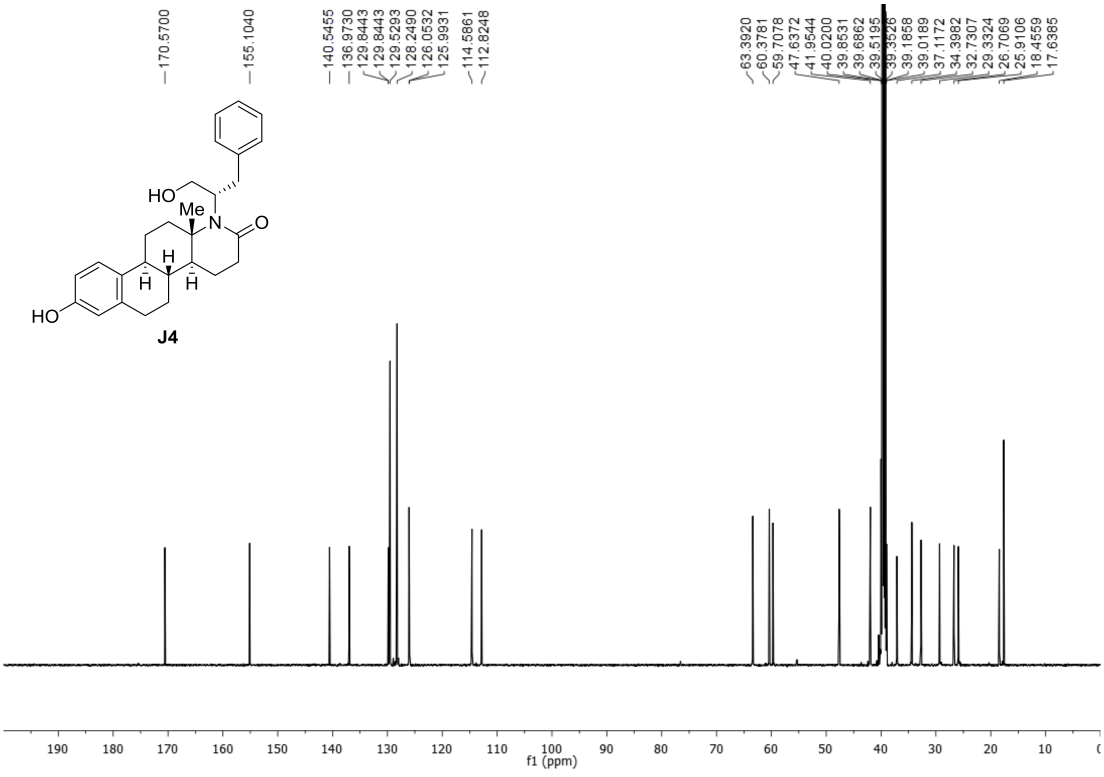

Supplementary Figure 409. ^{1}H NMR spectrum in DMSO-d_6, 400 MHz, of **J5**.

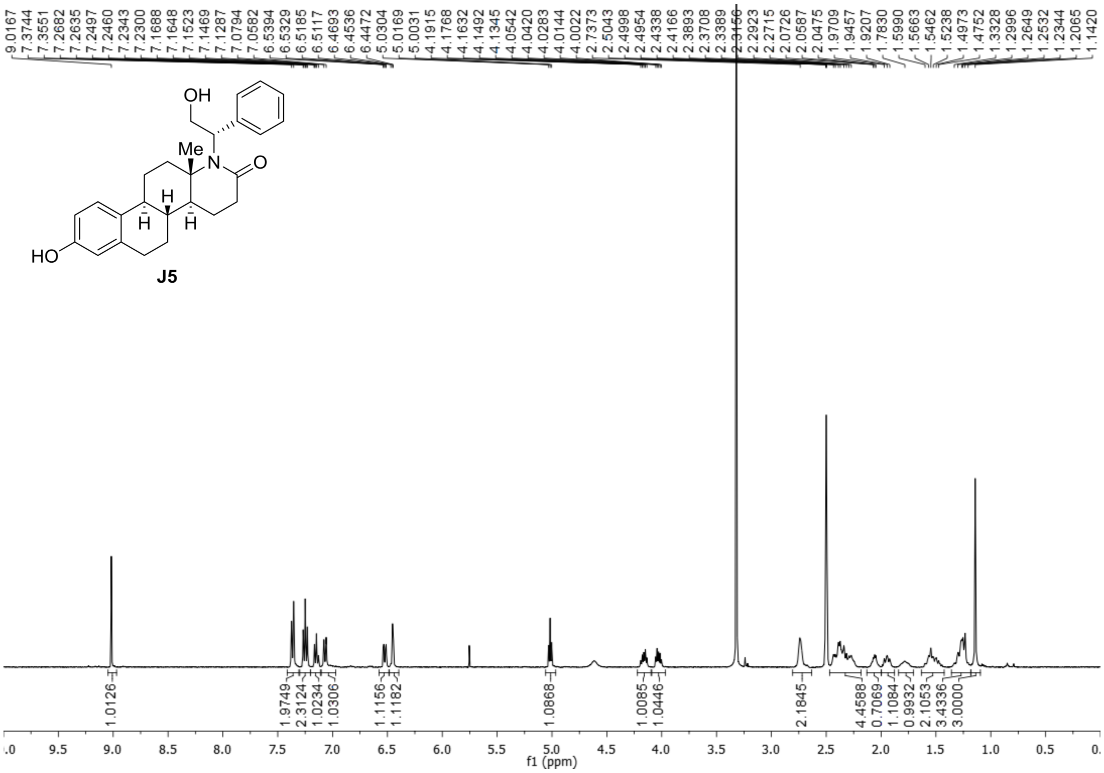

Supplementary Figure 410. ^{13}C NMR spectrum in DMSO-d_6, 101 MHz, of **J5**.

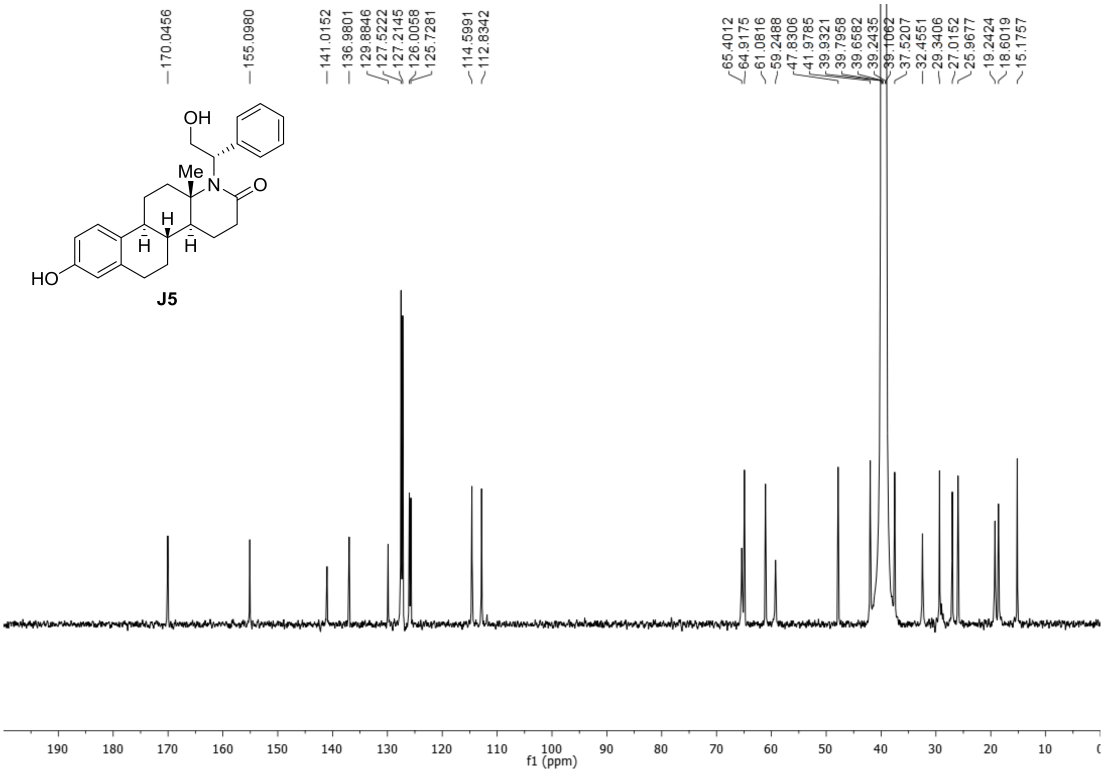

Supplementary Figure 411. ^{13}C NMR (APT) spectrum in DMSO-d_6, 101 MHz, of **J5**.

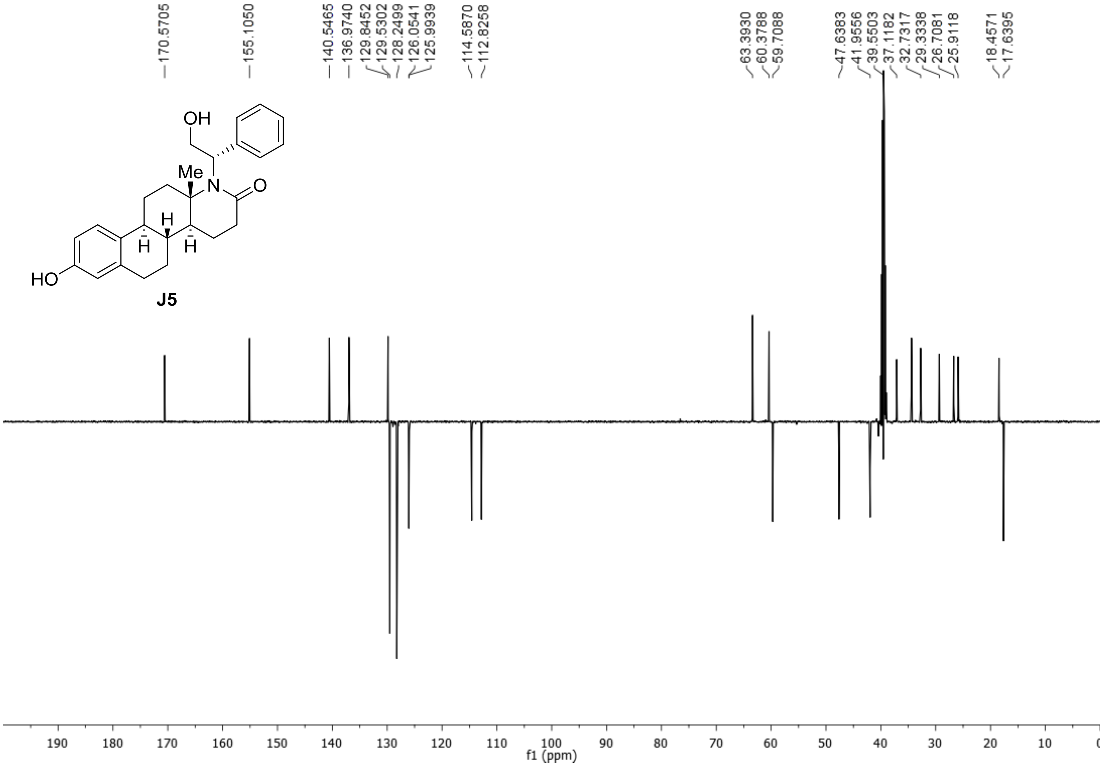

Supplementary Figure 412. ^{1}H NMR spectrum in DMSO-d_6, 400 MHz, of **J6**.

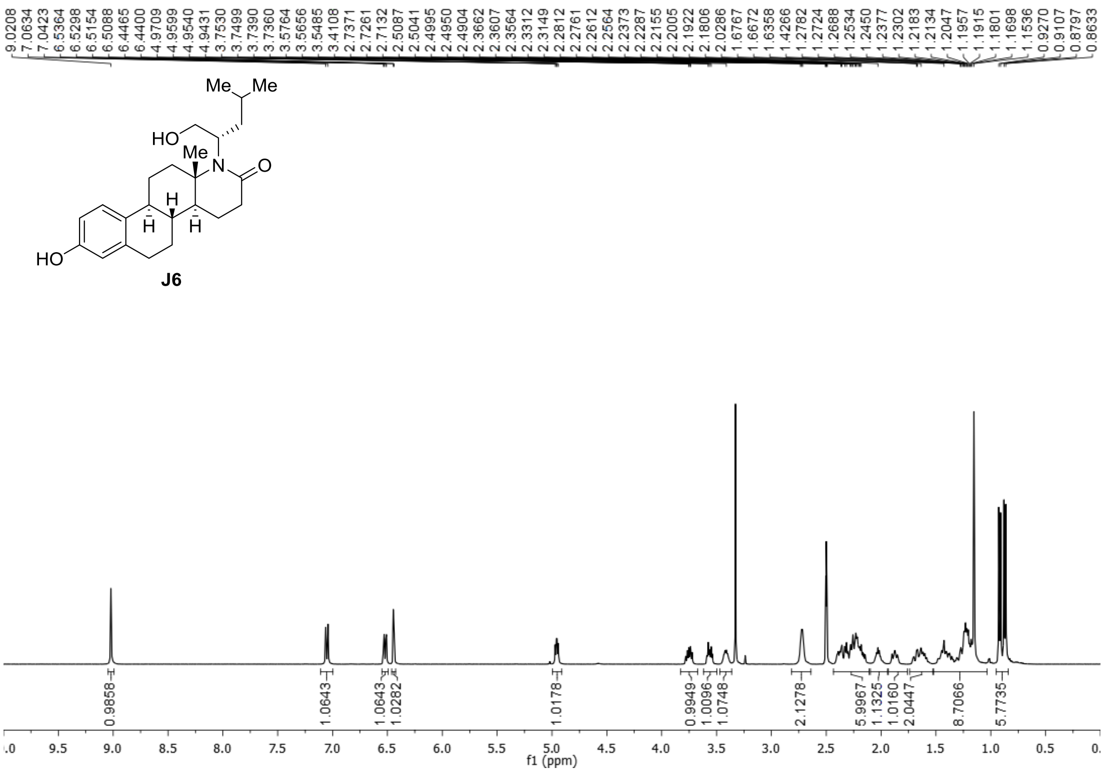

Supplementary Figure 413. ^{13}C NMR spectrum in DMSO-d_6, 126 MHz, of **J6**.

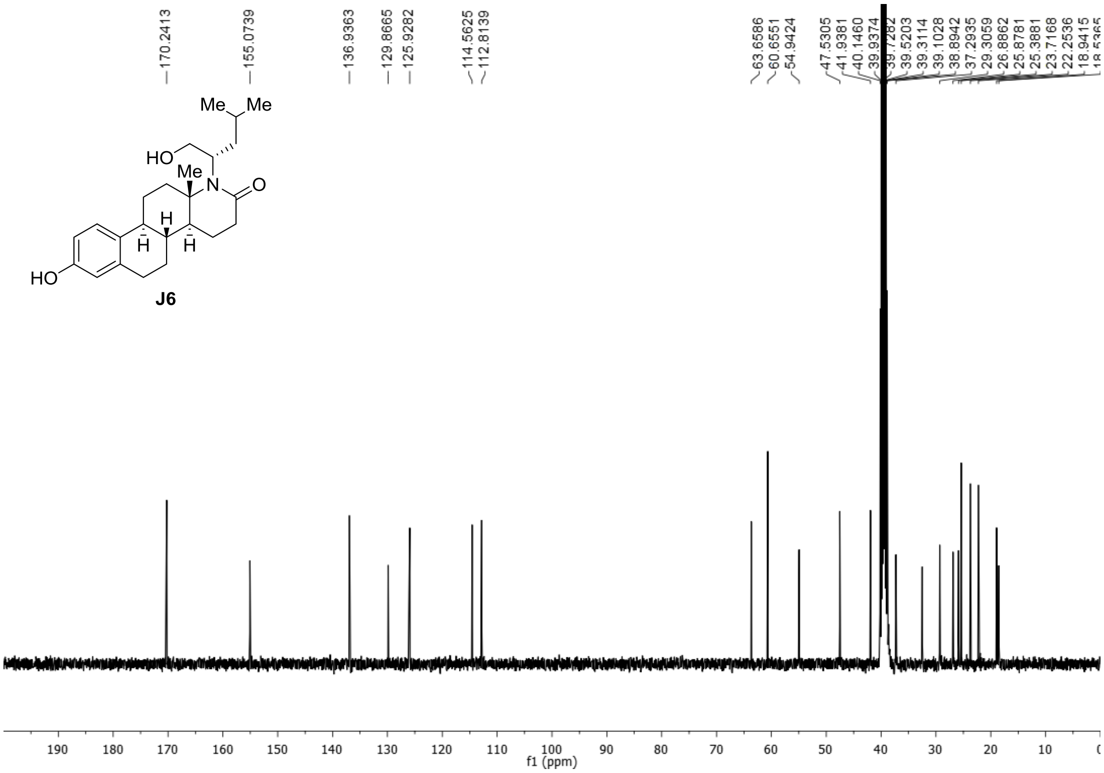

Supplementary Figure 414. ^{13}C NMR (APT) spectrum in DMSO-d_6, 126 MHz, of **J6**.

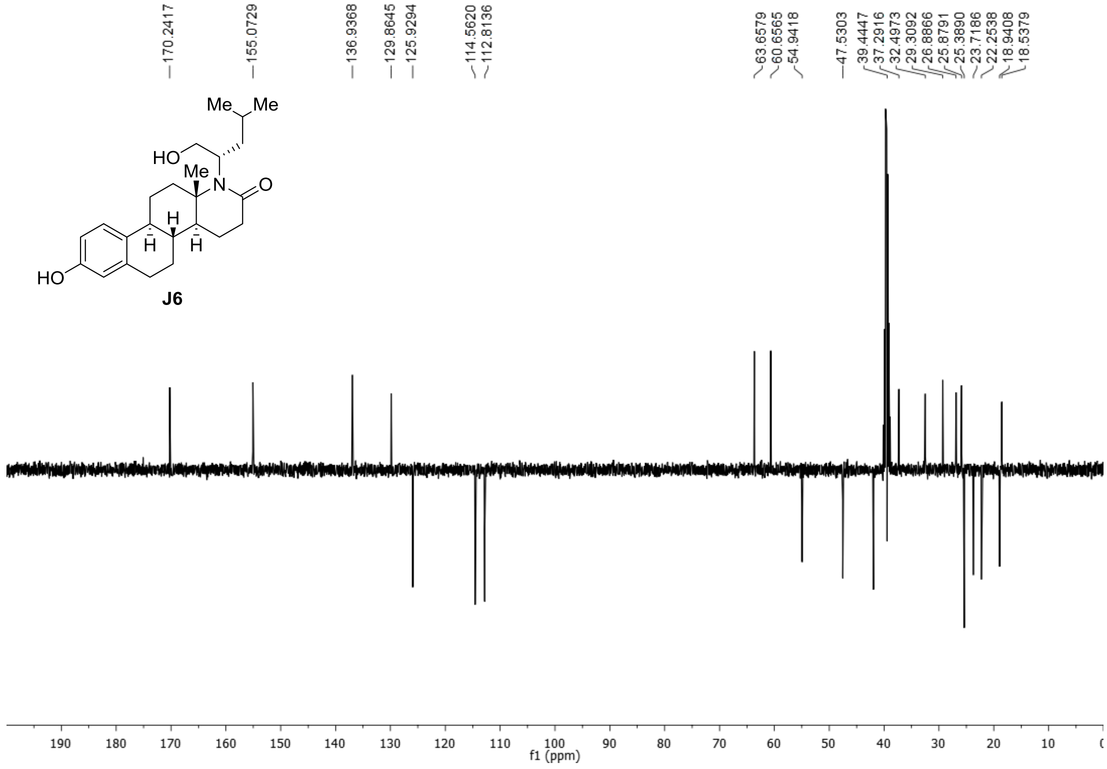

Supplementary References

1 Wrobleski, A., Coombs, T. C., Huh, C. W., Li, S. W. & Aubé, J. The Schmidt reaction. *Org. Reactions* **78**, 1-37 (2012).

2 Gracias, V., Milligan, G. L. & Aube, J. Efficient nitrogen ring-expansion process facilitated by in situ hemiketal formation. An asymmetric Schmidt reaction. . *J. Am. Chem. Soc.* **117**, 8047-8048, doi:10.1021/ja00135a036 (1995).

3 Gracias, V., Frank, K. E., Milligan, G. L. & Aubé, J. Ring expansion by in situ tethering of hydroxy azides to ketones: The boyer reaction. *Tetrahedron* **53**, 16241-16252 (1997).

4 Hewlett, N. D., Aubé, J. & Radkiewicz-Poutsma, J. L. Ab initio approach to understanding the stereoselectivity of reactions between hydroxyalkyl azides and ketones. *J. Org. Chem.* **69**, 3439-3446, doi:10.1021/jo035632t (2004).

5 Gawley, R. E. The Beckmann reactions: rearrangements, elimination–additions, fragmentations, and rearrangement–cyclizations. *Organic reactions* (1988).

6 Gutierrez, O., Aubé, J. & Tantillo, D. J. Mechanism of the Acid-Promoted Intramolecular Schmidt Reaction: Theoretical Assessment of the Importance of Lone Pair-Cation, Cation-π, and Steric Effects in Controlling Regioselectivity. *J. Org. Chem.* **ASAP**, doi:10.1021/jo202338m (2012).

7 Mukherjee, S., Maji, B., Tlahuext-Aca, A. & Glorius, F. Visible-light-promoted activation of unactivated C(sp3)–H bonds and their selective trifluoromethylthiolation. *J. Am. Chem. Soc.* **138**, 16200-16203, doi:10.1021/jacs.6b09970 (2016).

8 Mo, F., Lim, H. N. & Dong, G. Bifunctional ligand-assisted catalytic ketone α-alkenylation with internal alkynes: Controlled synthesis of enones and mechanistic studies. *J. Am. Chem. Soc.* **137**, 15518-15527, doi:10.1021/jacs.5b10466 (2015).

9 Purushottamachar, P. & Njar, V. C. O. A new simple and high-yield synthesis of 5α-dihydrotestosterone (DHT), a potent androgen receptor agonist. *Steroids* **77**, 1530-1534 (2012).

10 Singh, C. *et al.* Bile acid-based 1,2,4-trioxanes: Synthesis and antimalarial assessment. *J. Med. Chem.* **55**, 10662-10673, doi:10.1021/jm301323k (2012).

11 Hamilton, N. M. *et al.* Novel steroid inhibitors of glucose 6-phosphate dehydrogenase. *J. Med. Chem.* **55**, 4431-4445, doi:10.1021/jm300317k (2012).

12 Marcano, D., De Méndez, J., Ortiz, A. C. & Salinas, M. Carbon-13 nuclear magnetic resonance spectra of some heterocyclic D-homoandrostanes. *Organic Magnetic Resonance* **22**, 736-738, doi:10.1002/mrc.1270221116 (1984).

13 Moriyama, K., Nakamura, Y. & Togo, H. Oxidative debenzylation of *N*-benzyl amides and O-benzyl ethers using alkali metal bromide. *Org. Lett.* **16**, 3812-3815, doi:10.1021/ol501703y (2014).

14 Hilton, P. J. *et al.* Selective inhibition of the cellular sodium pump by emicymarin and 14ß anhydroxy bufadienolides. *Steroids* **75**, 1137-1145 (2010).

15 Rodríguez-Molina, B., Pérez-Estrada, S. & Garcia-Garibay, M. A. Amphidynamic crystals of a steroidal bicyclo[2.2.2]octane rotor: A high symmetry group that rotates faster than smaller methyl and methoxy groups. *J. Am. Chem. Soc.* **135**, 10388-10395, doi:10.1021/ja4024463 (2013).

16 Badiang, J. G. & Aubé, J. One-step conversion of aldehydes to oxazolines and 5,6-dihydro-4H-1,3-oxazines using 1,2- and 1,3-azido alcohols. *J. Org. Chem.* **61**, 2484-2487, doi:10.1021/jo9521256 (1996).

17 Sahasrabudhe, K. *et al.* Asymmetric Schmidt reaction of hydroxyalkyl azides with ketones. *J. Am. Chem. Soc.* **125**, 7914-7922, doi:10.1021/ja0348896 (2003).

18 Ramanathan, S. K. *et al.* Modular synthesis of cyclic peptidomimetics inspired by γ-turns. *Org. Lett.* **7**, 1059-1062, doi:10.1021/ol047323a (2005).

19 Chaudhry, P., Schoenen, F., Neuenswander, B., Lushington, G. H. & Aubé, J. One-step synthesis of oxazoline and dihydrooxazine libraries. *J. Comb. Chem.* **9**, 473-476, doi:10.1021/cc060159t (2007).

20 Schmidt, A. W. *et al.* Regio- and stereospecific synthesis of cholesterol derivatives and their hormonal activity in Caenorhabditis elegans. *Eur. J. Org. Chem.* **2006**, 3687-3706, doi:10.1002/ejoc.200600394 (2006).

21 Park, J.-Y. *et al.* Simplified heterocyclic analogues of fluoxetine inhibit inducible nitric oxide production in lipopolysaccharide-induced BV2 cells. *Biol. Pharm. Bull.* **34**, 538-544, doi:10.1248/bpb.34.538 (2011).

22 Werner, T. & Barrett, A. G. M. Simple method for the preparation of esters from grignard reagents and alkyl 1-imidazolecarboxylates. *J. Org. Chem.* **71**, 4302-4304, doi:10.1021/jo060562m (2006).

23 Salunkhe, A. M. & Burkhardt, E. R. Highly enantioselective reduction of prochiral ketones with N,N-diethylaniline·borane (DEANB) in oxazaborolidine-catalyzed reductions. *Tetrahedron Lett.* **38**, 1523-1526 (1997).

24 De Angelis, S., De Renzo, M., Carlucci, C., Degennaro, L. & Luisi, R. A convenient enantioselective CBS-reduction of arylketones in flow-microreactor systems. *Org. Biomol. Chem.* **14**, 4304-4311, doi:10.1039/C6OB00336B (2016).

25 Touge, T. *et al.* Oxo-tethered ruthenium(II) complex as a bifunctional catalyst for asymmetric transfer hydrogenation and H2 hydrogenation. *J. Am. Chem. Soc.* **133**, 14960-14963, doi:10.1021/ja207283t (2011).

26 Klein, M., Krainz, K., Redwan, I. N., Dinér, P. & Grøtli, M. Synthesis of chiral 1,4-disubstituted-1,2,3-triazole derivatives from amino acids. *Molecules* **14**, 5124 (2009).

27 Wang, C., Wang, S., Xu, Y., Hu, Y. & Hu, H. Preparation of (5α,13α)-D-azasteroids as key precursors of a new family of potential GABAA receptor modulators. *Steroids* **68**, 677-683 (2003).

28 Regan, B. M. & Hayes, F. N. 17- and 17a-aza-D-homosteroids. *J. Am. Chem. Soc.* **78**, 639-643, doi:10.1021/ja01584a033 (1956).

29 Huang, Y. *et al.* Synthesis and cytotoxicity of 17a-aza-d-homo-androster-17-one derivatives. *Bioorganic Med. Chem. Lett.* **21**, 3641-3643 (2011).

30 Czajkowska-Szczykowska, D. *et al.* Macrocyclic molecular rotors with bridged steroidal frameworks. *J. Org. Chem.* **77**, 9970-9978, doi:10.1021/jo3020402 (2012).

31 Motiwala, H. F. *et al.* Overcoming product inhibition in catalysis of the intramolecular Schmidt reaction. *J. Am. Chem. Soc.* **135**, 9000-9009, doi:10.1021/ja402848c (2013).

32 Smith, A. B., Kürti, L. & Davulcu, A. H. A new modular indole synthesis. Construction of the highly strained CDEF parent tetracycle of nodulisporic acids A and B. *Org. Lett.* **8**, 2167-2170, doi:10.1021/ol0606536 (2006).
